# Supplementary material for: NFATc1 drives Orai3 transcription and proteolysis by harnessing epigenome differences in the MARCH8 promoter
Source: EMBO J. 2025 Sep 29;44(21):6137–67. doi: 10.1038/s44318-025-00572-4 (PMC12583688; doi:10.1038/s44318-025-00572-4)
Supplement: Supplementary file 1 — Appendix [file 44318_2025_572_MOESM1_ESM.pdf]

Table of contents:

Title	Page no.
Appendix Table S1	2
Appendix Table S2	2
Appendix Table S3	2
Appendix Table S4	2
Appendix Table S5	3
Appendix Table S6	3
Appendix Table S7	4

Appendix Table S1: qRT-PCR Primers

Gene	Forward Primer	Reverse Primer
NFATc1	CACCGCATCACAGGGAAGAG	GCACAGTCAATGACGGCTC
Orai3	TCCCCATCAGTCTGTCCCTT	GAAGGTCCCACAAGCTCTCC
MARCH8	GTGATCTCGAGAGAGACCTGCG	GAGATGGCATCCTGGGATGG
GAPDH	AACTGCTTAGCACCCCTGGC	ATGACCTTGCCCACAGCCTT

Appendix Table S2: Cloning Primers

Promoter	Forward Primer	Reverse Primer
Orai3 WT	GCGGGTACCTTCATCGTCATGG AAACTTGTATCC	AATATAAAGCTTGCTCGCCCGCG TC
Orai3 Truncation	CATGGTACCTCTTAGTTGCCATG GGAAC	AAGAAGCTTACCAGGCGGTCAC TA
MARCH8 WT	TATGGTACCCTGGCAGTGGTTG AAACT	ATATGCTAGCCGCCTGTTTACAC CCTA

Appendix Table S3: Orai3 Promoter site-directed Mutagenesis Primers

Orai3 deletion mutants	Forward Primer	Reverse Primer
Δ1017	TATCCCATTTGCCCAGGGAAC	CGATGAAGGTACCGGCCAG
Δ990	AGCCAACGGACACCTCCCG	ACCAGGAGTGGCAGTTCCC
Δ920	CCCCTCTTAGTTGCCATGG	GCCTTAGCACTGGCATCGG

Appendix Table S4: ChIP Primers

Primer	Sequence
Orai3 Promoter Forward Primer Set 1	GCCCAAGAATGGGGACCATGGG

Orai3 Promoter Reverse Primer Set 1	CCAGGAGTGGCAGTTCCCTG
Orai3 Promoter Forward Primer Set 2	CAGGGAACTGCCACTCCTGG
Orai3 Promoter Reverse Primer Set 2	CCCATGGCAACTAAGAGGGGGCC
MARCH8 Promoter Forward Primer	GCCTCAGGTAAGAAAG
MARCH8 Promoter Reverse Primer	GCCCTGACTACATGTT

Appendix Table S5: siRNAs

siRNA	Catalog No.	Target Sequence
siNT (Human)	D-001810-10-20	UGGUUUACAUGUCGACUAA, UGGUUUACAUGUUGUGUGA, UGGUUUACAUGUUUUCUGA, UGGUUUACAUGUUUUCCUA
siNFATc1 (Human)	Custom synthesized from Eurogentec	UCAGAAACUCCGACAUUGA UUUCGGAAUCAGAGGAUAA GGACAGCUAUCCGGUCGUG AGGAAGAACACACGGGUAC
siMARCH8 (Human)	L-007161-00-0005	GAAUGGCCCUUUUGGACUA GAGCAGAAAUCAUUCACGU GGAAGAGACUCAAGGCCUA UAAAGUGUAUGUGCAAUUG

Appendix Table S6: Orai3 over-expression site directed mutagenesis primers

K2R FP	AGCTATGCCGGGCGGCGAGG
K2R RP	CCGCCCGGCATAGCTCGAGATCT
K274R FP	GGCACACCCGACAGACCGCTACAA

K274R RP	TCTGTCGGGTGTGCCACCAA
K279R FP	CCGCTACCCGCAGGAACTAGAGGAA
K279R RP	TCCTGCGGGTAGCGGTCTGTCTT
ΔLMVXXXL FP	TCTTTGCACTGCCCCACATTGAA
ΔLMVXXXL RP	GGGGCAGTGCAAAGAGGTGCACA
ΔGXXXG FP	GTGGTGGTCCAGGCTGGCAAGC
ΔGXXXG RP	AGCCTGGACCACCACAGGCTT

Appendix Table S7: MeDIP Primers

Primer	Sequence
Forward Primer	GCCAGCTCAGAGCCCACCT
Reverse Primer	CCCTGGGCGCTGACCGTTT
