# Supplementary figures and images for: NFATc1 drives Orai3 transcription and proteolysis by harnessing epigenome differences in the MARCH8 promoter (part 1 of 3)
Source: EMBO J. 2025 Sep 29;44(21):6137–67. doi: 10.1038/s44318-025-00572-4 (PMC12583688; doi:10.1038/s44318-025-00572-4)

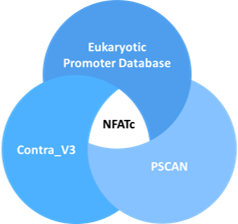

Supplement: Supplementary file 3 — Source data Fig. 1 [file 44318_2025_572_MOESM3_ESM.zip › Figure 1/Figure 1A/Figure 1A.png]

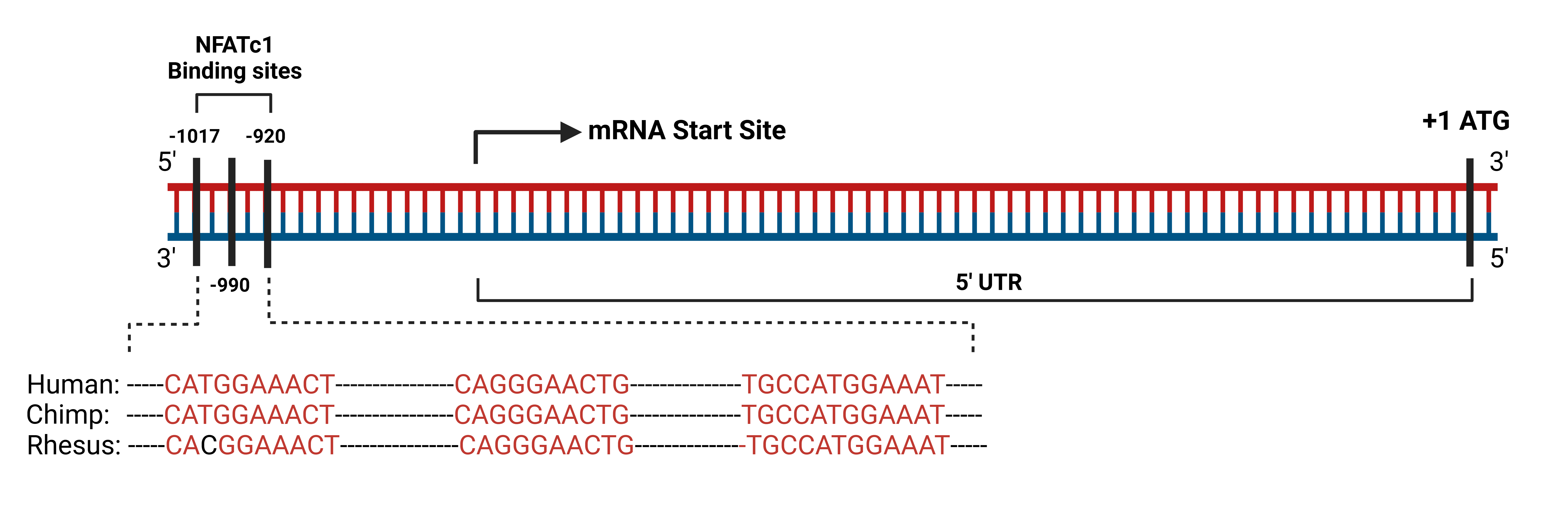

Supplement: Supplementary file 3 — Source data Fig. 1 [file 44318_2025_572_MOESM3_ESM.zip › Figure 1/Figure 1B/Orai3 Promoter.png]

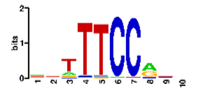

Supplement: Supplementary file 3 — Source data Fig. 1 [file 44318_2025_572_MOESM3_ESM.zip › Figure 1/Figure 1C/Figure 1C.png]

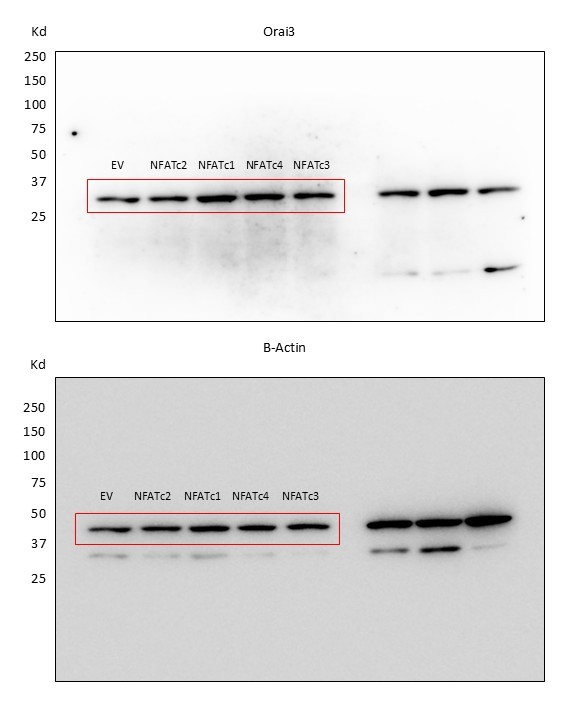

Supplement: Supplementary file 3 — Source data Fig. 1 [file 44318_2025_572_MOESM3_ESM.zip › Figure 1/Figure 1D/Figure 1D.jpg]

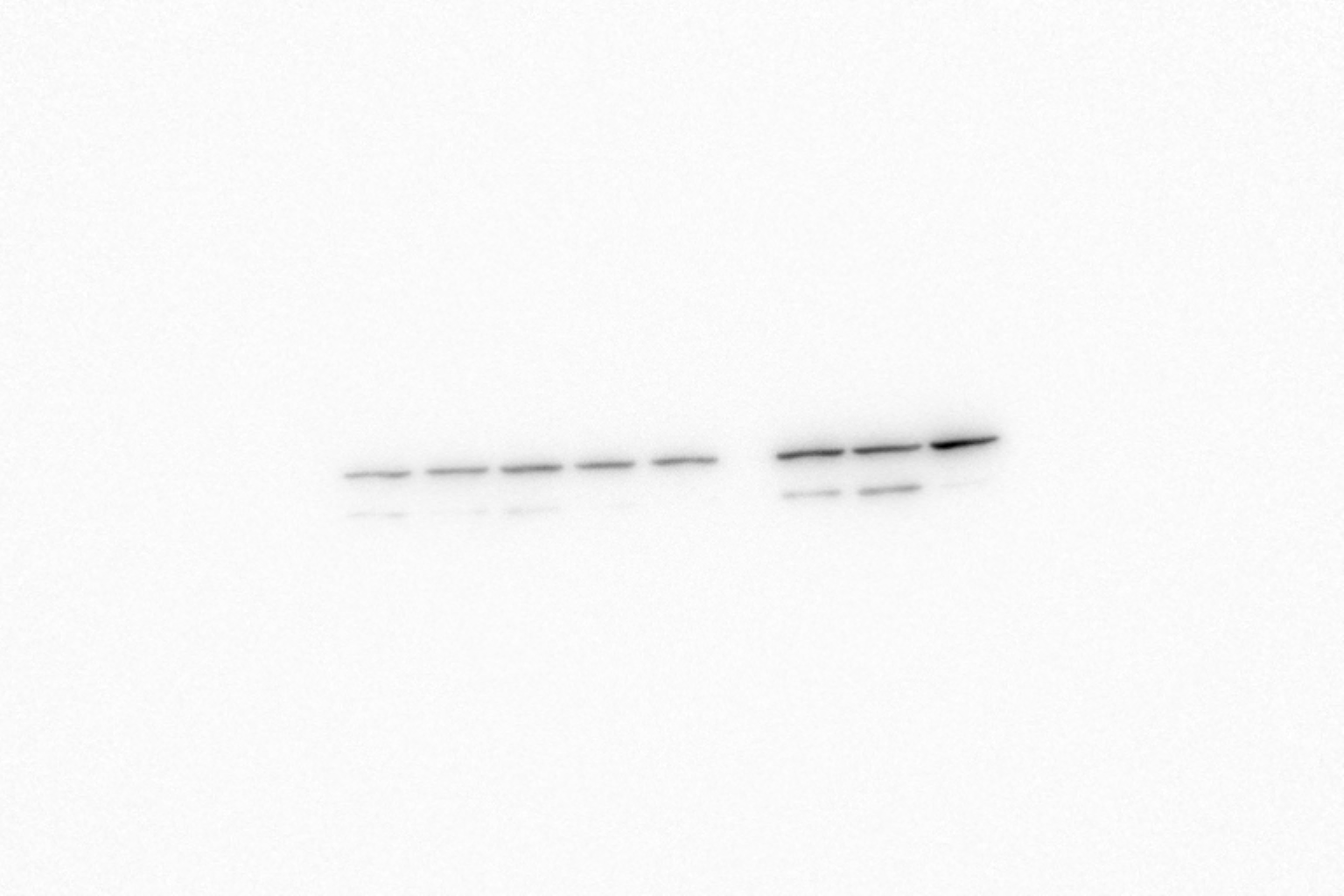

Supplement: Supplementary file 3 — Source data Fig. 1 [file 44318_2025_572_MOESM3_ESM.zip › Figure 1/Figure 1D/HEK B ACTIN 0.5 SEC.gel]

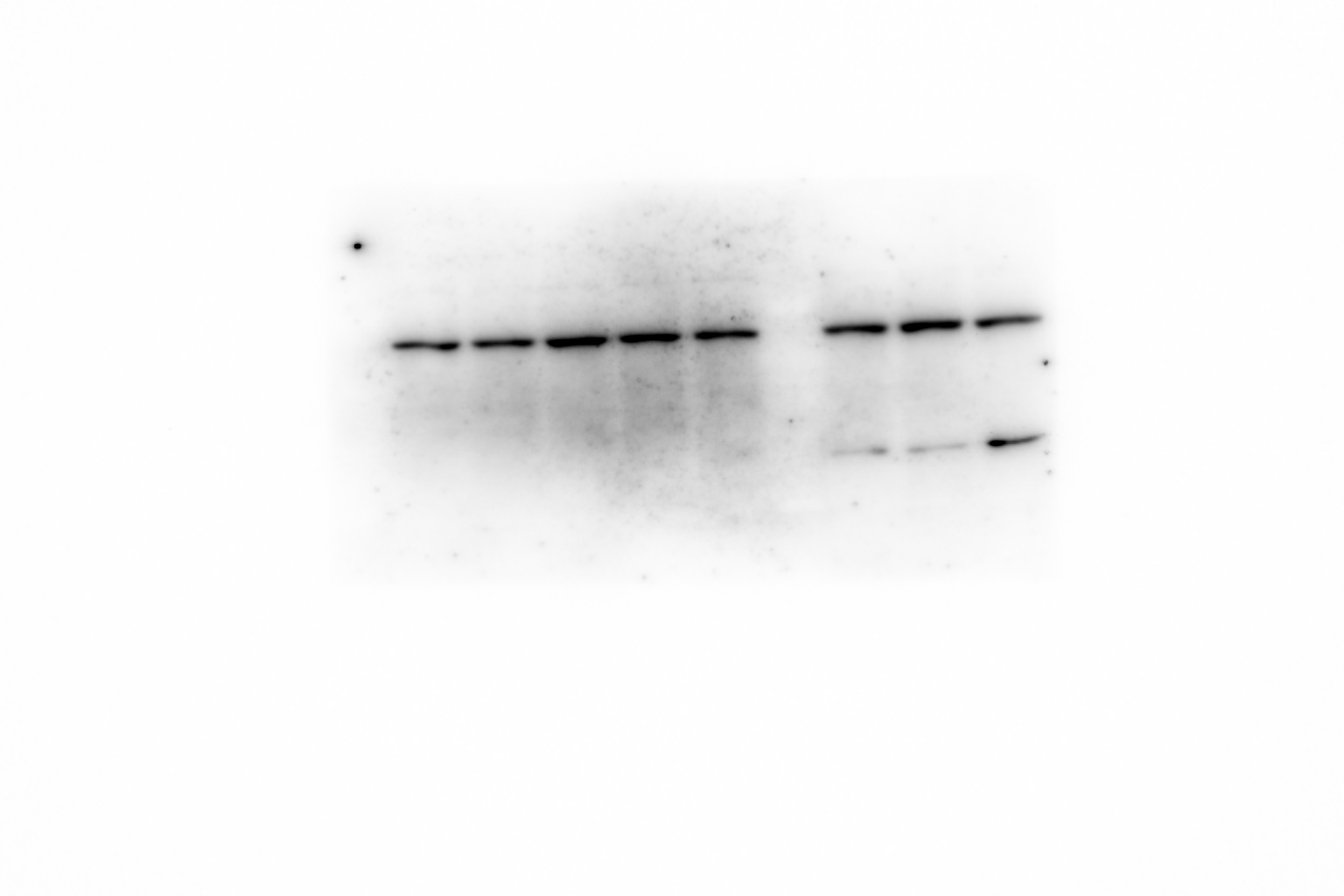

Supplement: Supplementary file 3 — Source data Fig. 1 [file 44318_2025_572_MOESM3_ESM.zip › Figure 1/Figure 1D/HEK ORAI3 240 SEC.gel]

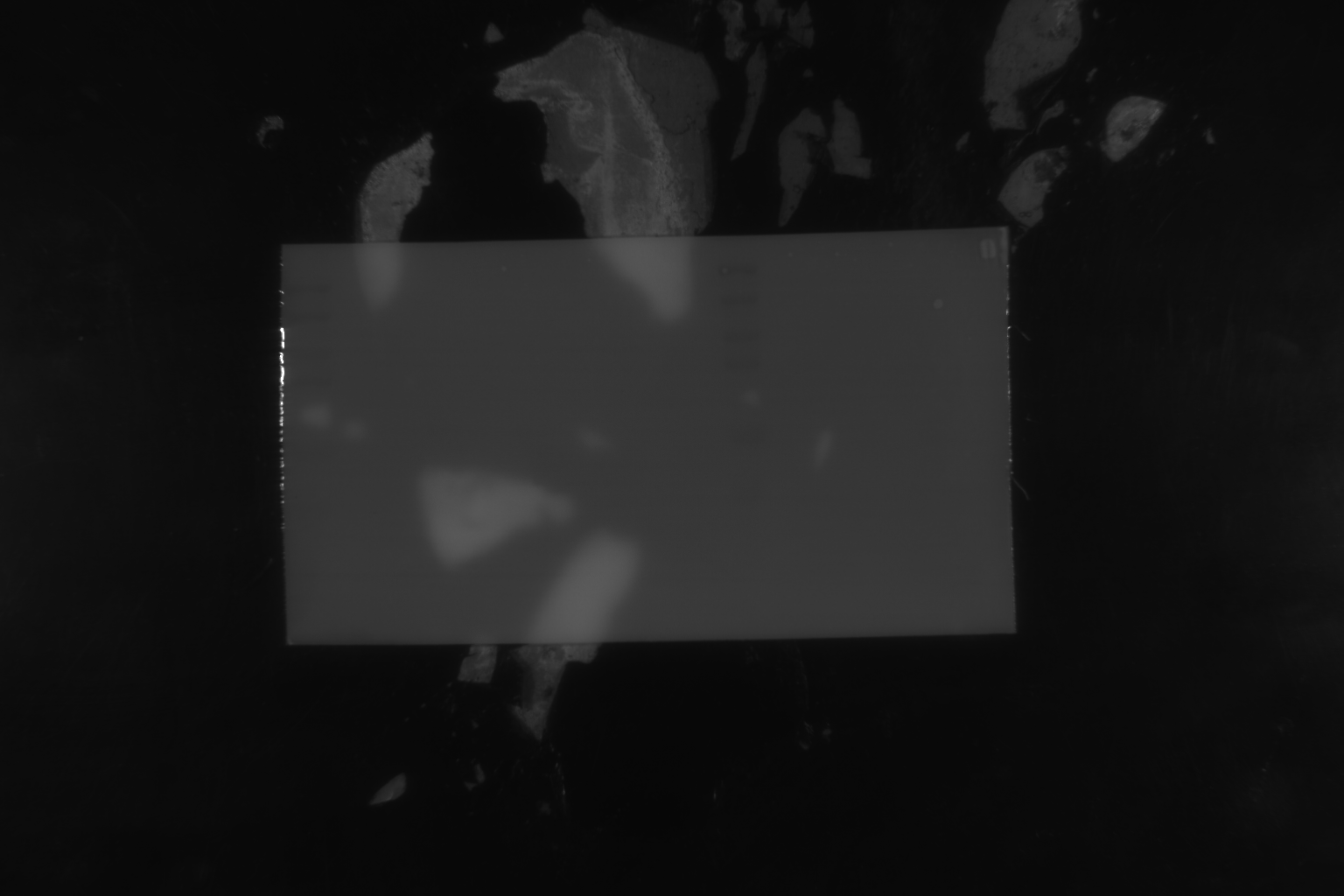

Supplement: Supplementary file 3 — Source data Fig. 1 [file 44318_2025_572_MOESM3_ESM.zip › Figure 1/Figure 1D/V_HEK B ACTIN 0.5 SEC.gel]

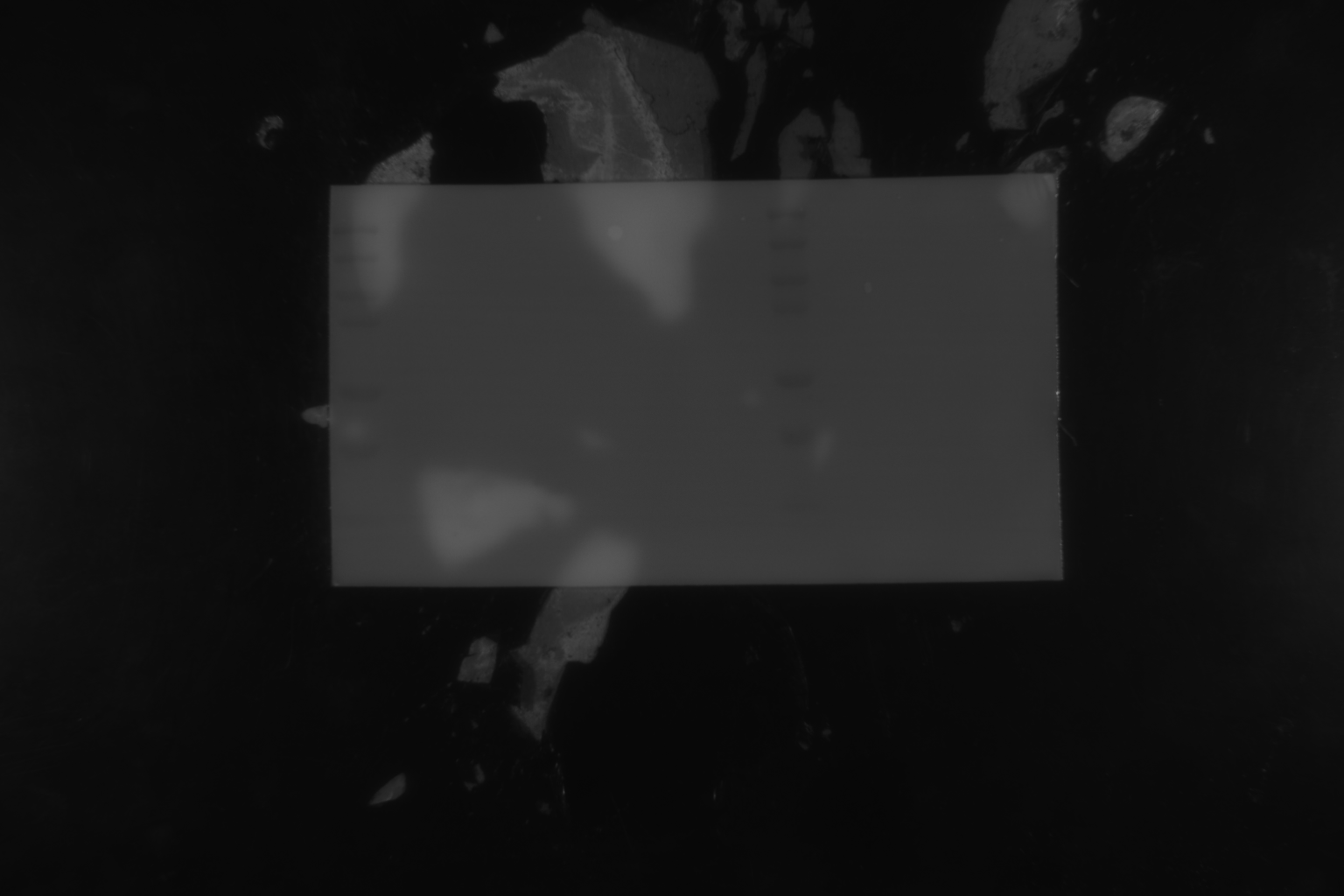

Supplement: Supplementary file 3 — Source data Fig. 1 [file 44318_2025_572_MOESM3_ESM.zip › Figure 1/Figure 1D/V_HEK ORAI3 240 SEC.gel]

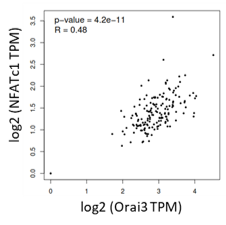

Supplement: Supplementary file 3 — Source data Fig. 1 [file 44318_2025_572_MOESM3_ESM.zip › Figure 1/Figure 1G/Figure 1G.png]

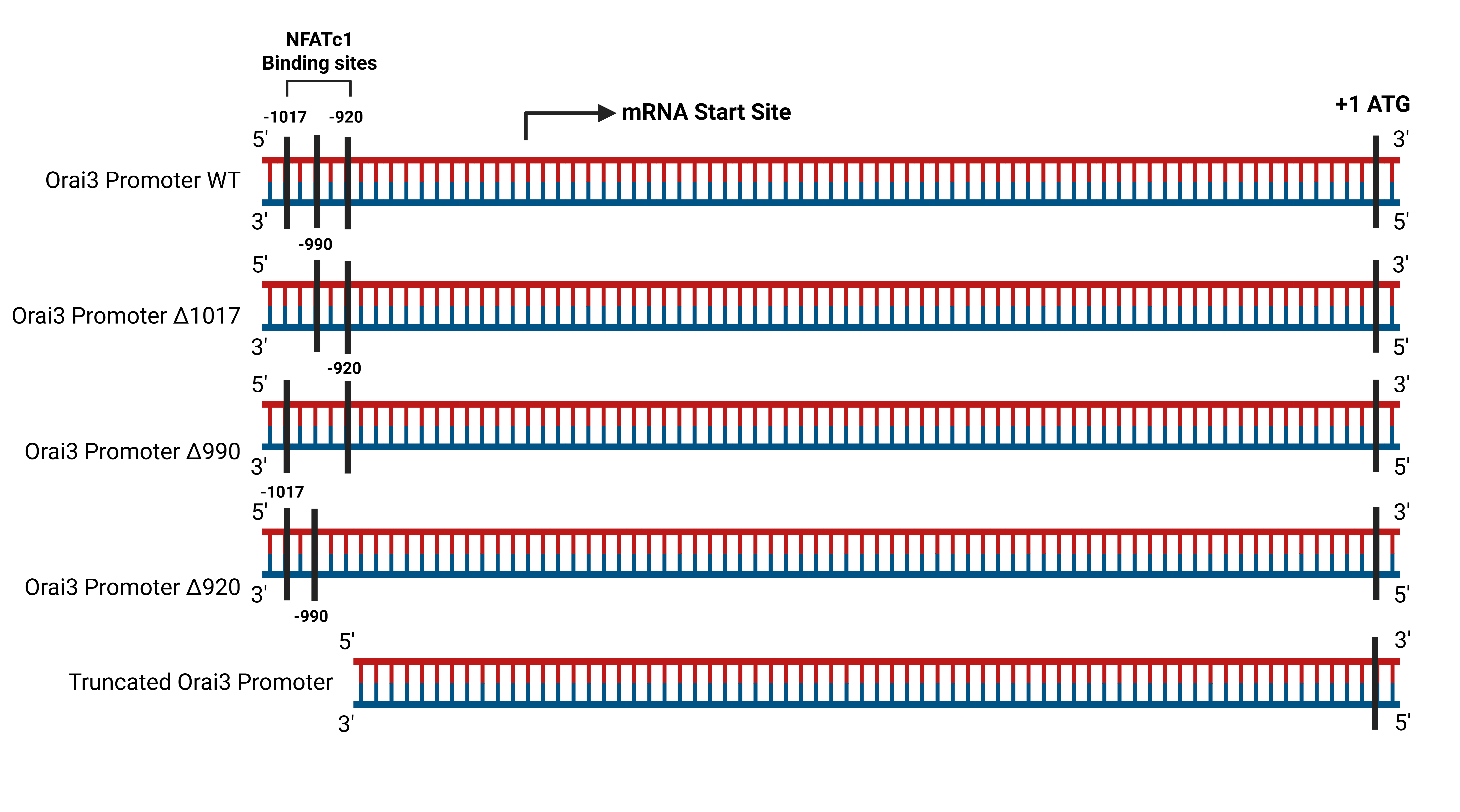

Supplement: Supplementary file 3 — Source data Fig. 1 [file 44318_2025_572_MOESM3_ESM.zip › Figure 1/Figure 1H/Orai3 Promoter SDM.png]

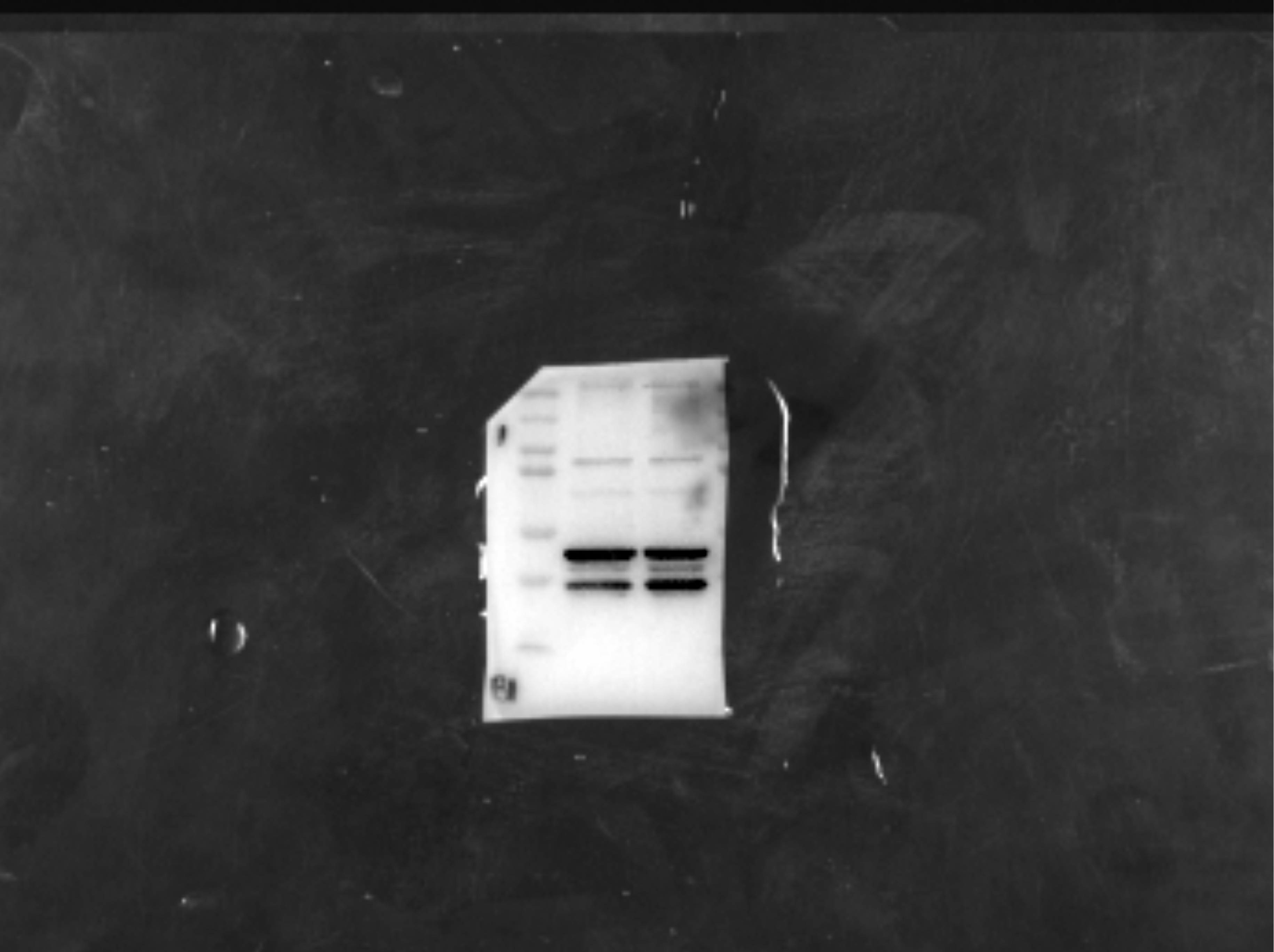

Supplement: Supplementary file 4 — Source data Fig. 2 [file 44318_2025_572_MOESM4_ESM.zip › Figure 2/Figure 2B/B Actin.png]

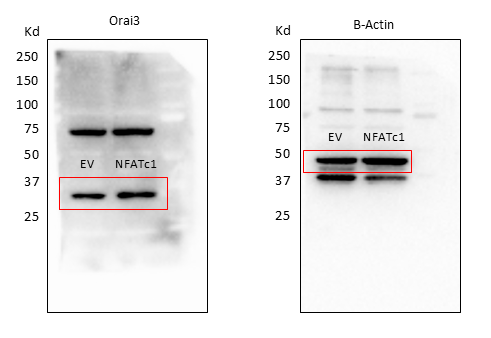

Supplement: Supplementary file 4 — Source data Fig. 2 [file 44318_2025_572_MOESM4_ESM.zip › Figure 2/Figure 2B/Figure 2B.png]

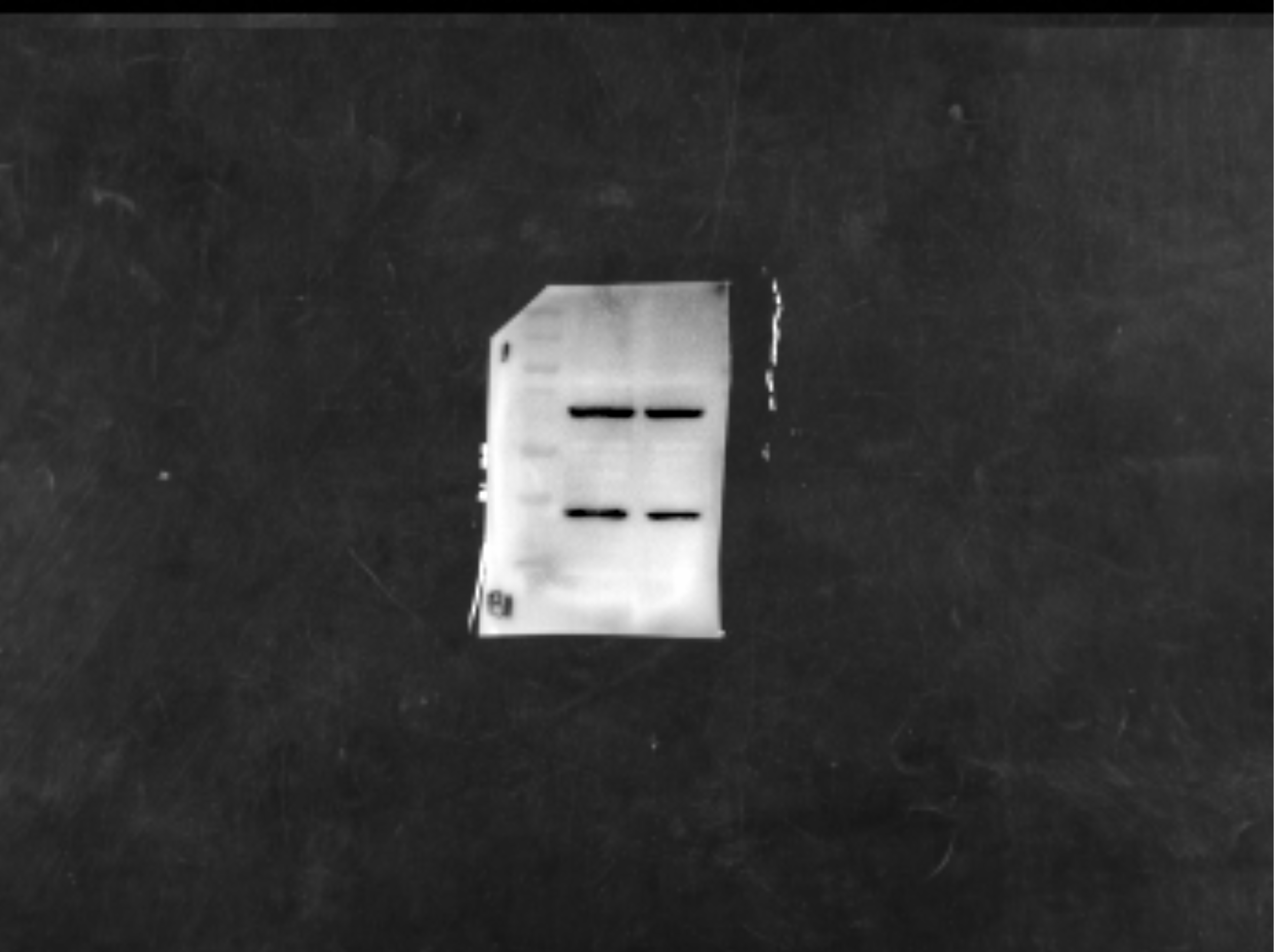

Supplement: Supplementary file 4 — Source data Fig. 2 [file 44318_2025_572_MOESM4_ESM.zip › Figure 2/Figure 2B/Orai3.png]

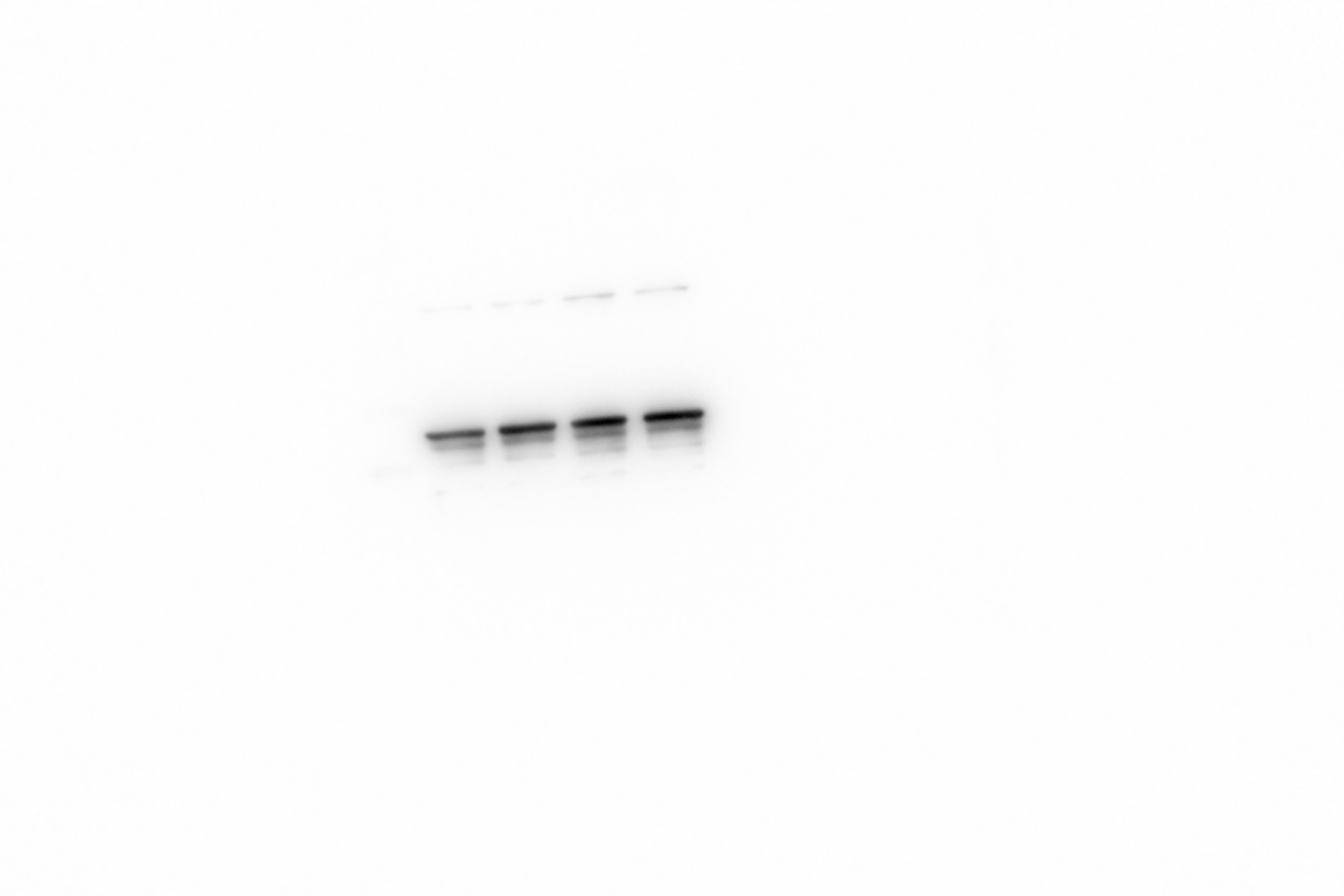

Supplement: Supplementary file 4 — Source data Fig. 2 [file 44318_2025_572_MOESM4_ESM.zip › Figure 2/Figure 2G/B ACTIN 1 SEC.gel]

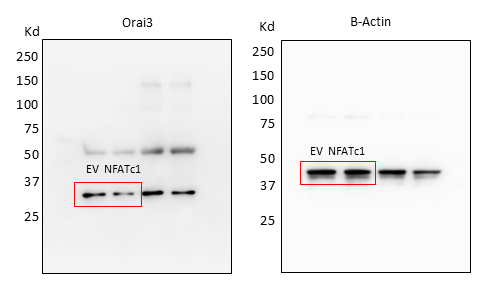

Supplement: Supplementary file 4 — Source data Fig. 2 [file 44318_2025_572_MOESM4_ESM.zip › Figure 2/Figure 2G/Figure 2G.png]

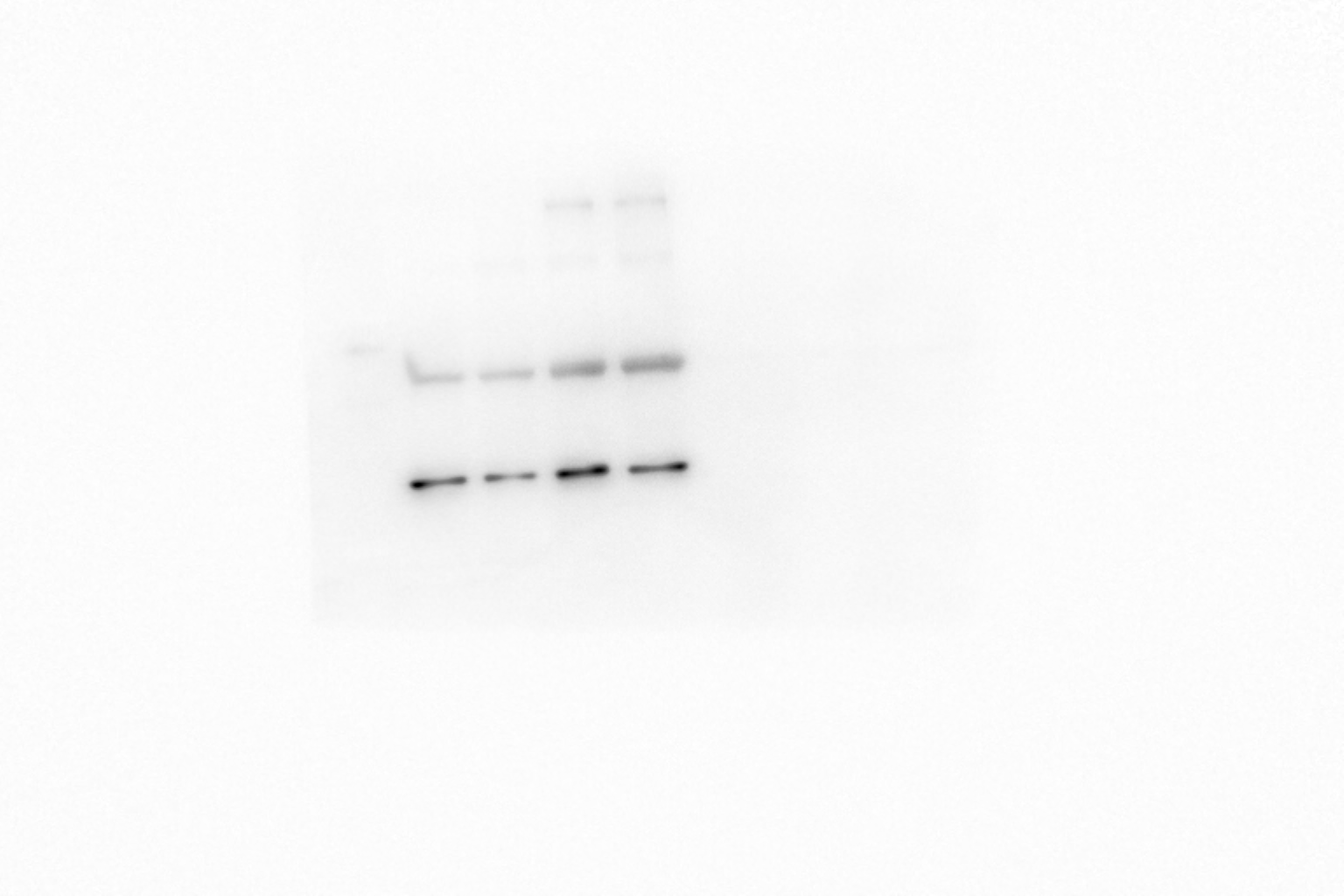

Supplement: Supplementary file 4 — Source data Fig. 2 [file 44318_2025_572_MOESM4_ESM.zip › Figure 2/Figure 2G/ORAI 3 2 SEC.gel]

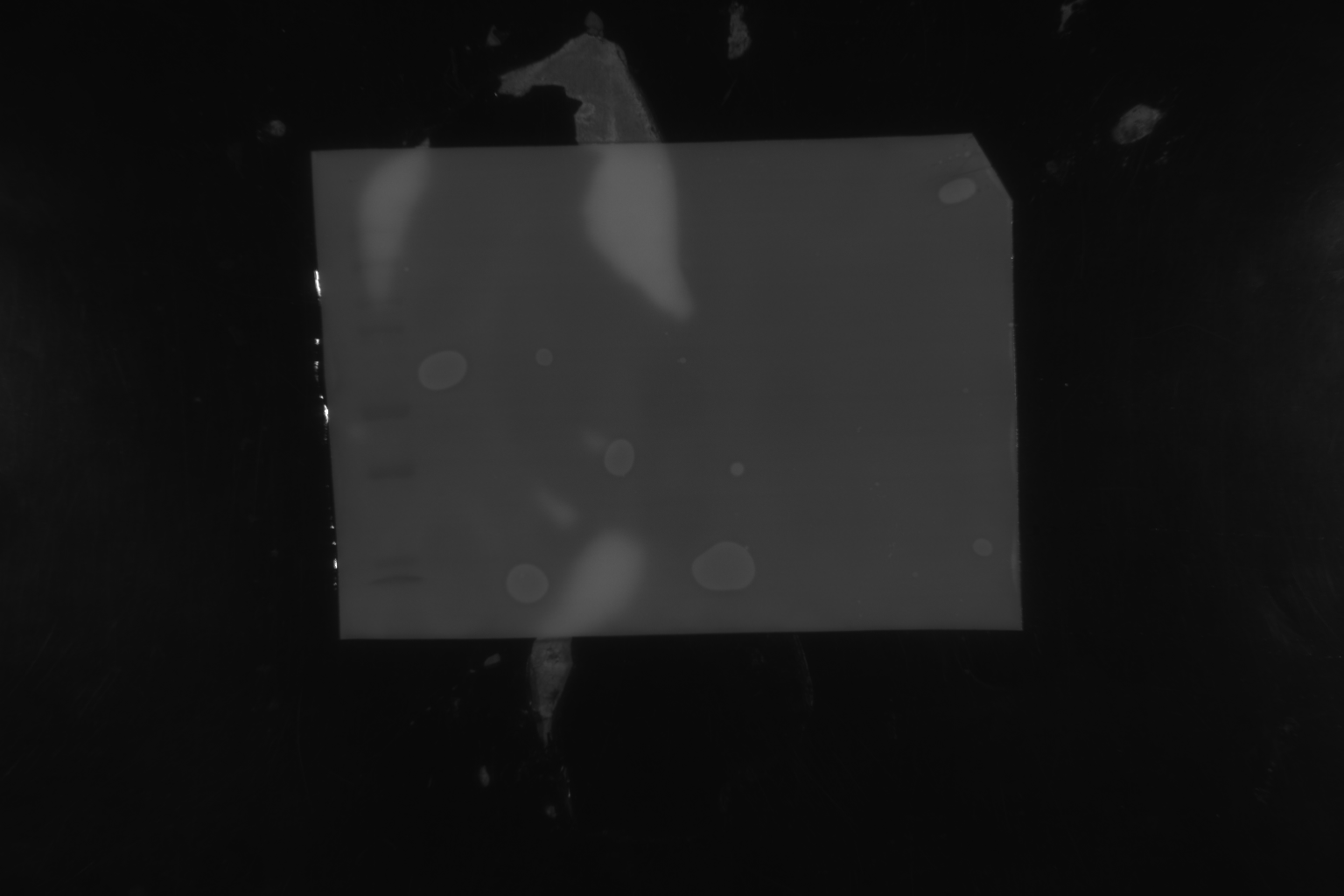

Supplement: Supplementary file 4 — Source data Fig. 2 [file 44318_2025_572_MOESM4_ESM.zip › Figure 2/Figure 2G/V_B ACTIN 1 SEC.gel]

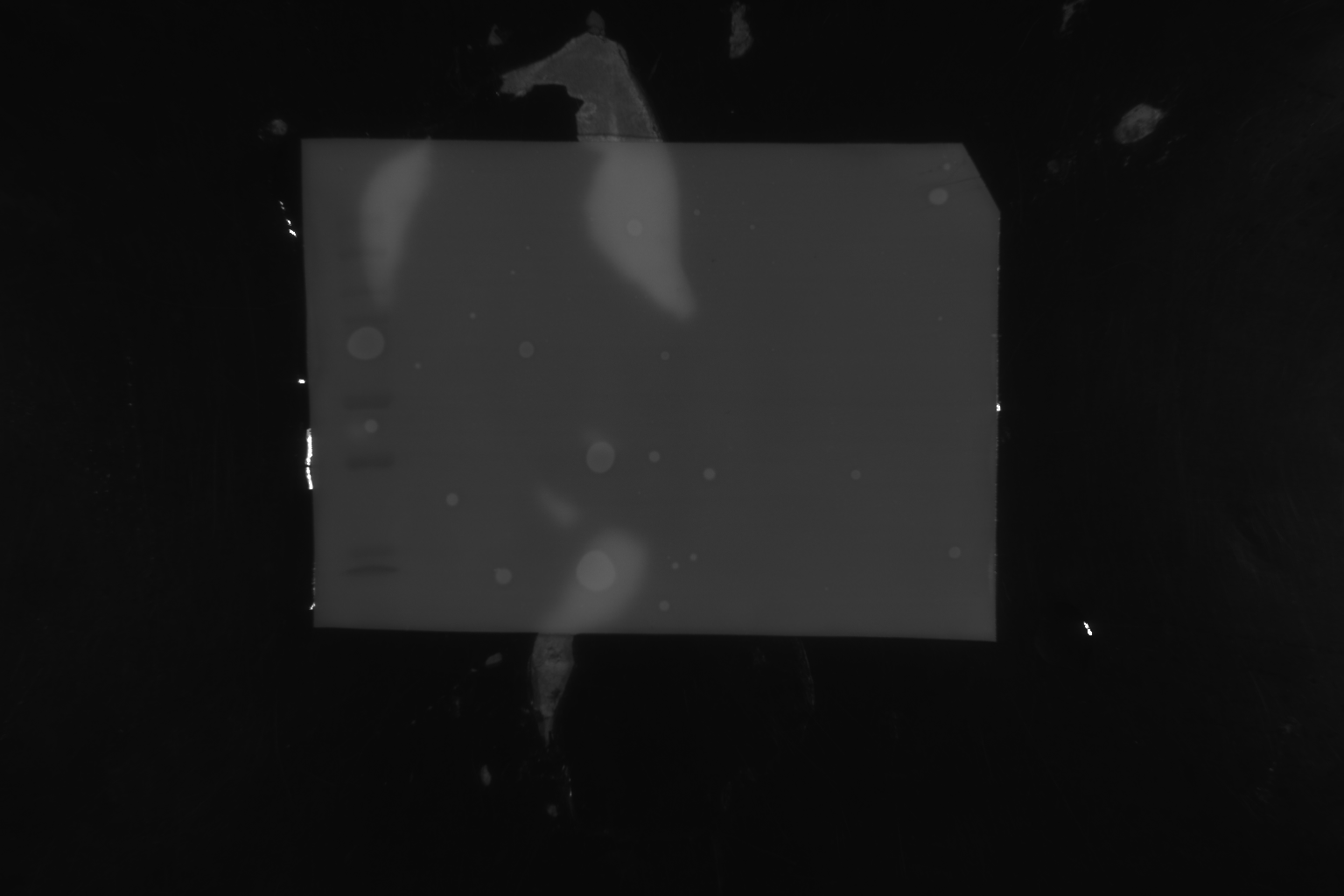

Supplement: Supplementary file 4 — Source data Fig. 2 [file 44318_2025_572_MOESM4_ESM.zip › Figure 2/Figure 2G/V_ORAI 3 2 SEC.gel]

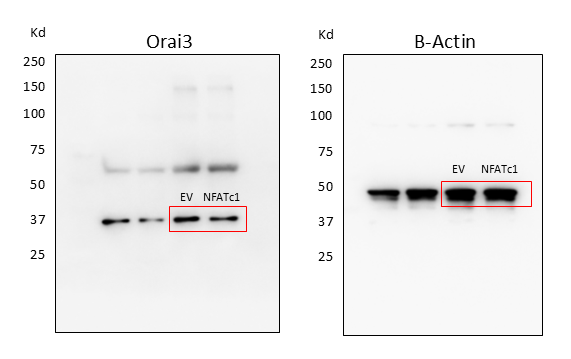

Supplement: Supplementary file 4 — Source data Fig. 2 [file 44318_2025_572_MOESM4_ESM.zip › Figure 2/Figure 2L/Figure 2L.png]

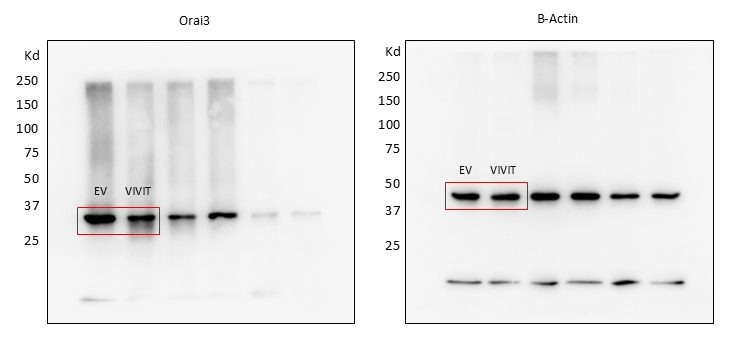

Supplement: Supplementary file 5 — Source data Fig. 3 [file 44318_2025_572_MOESM5_ESM.zip › Figure 3/Figure 3B/Figure 3B.png]

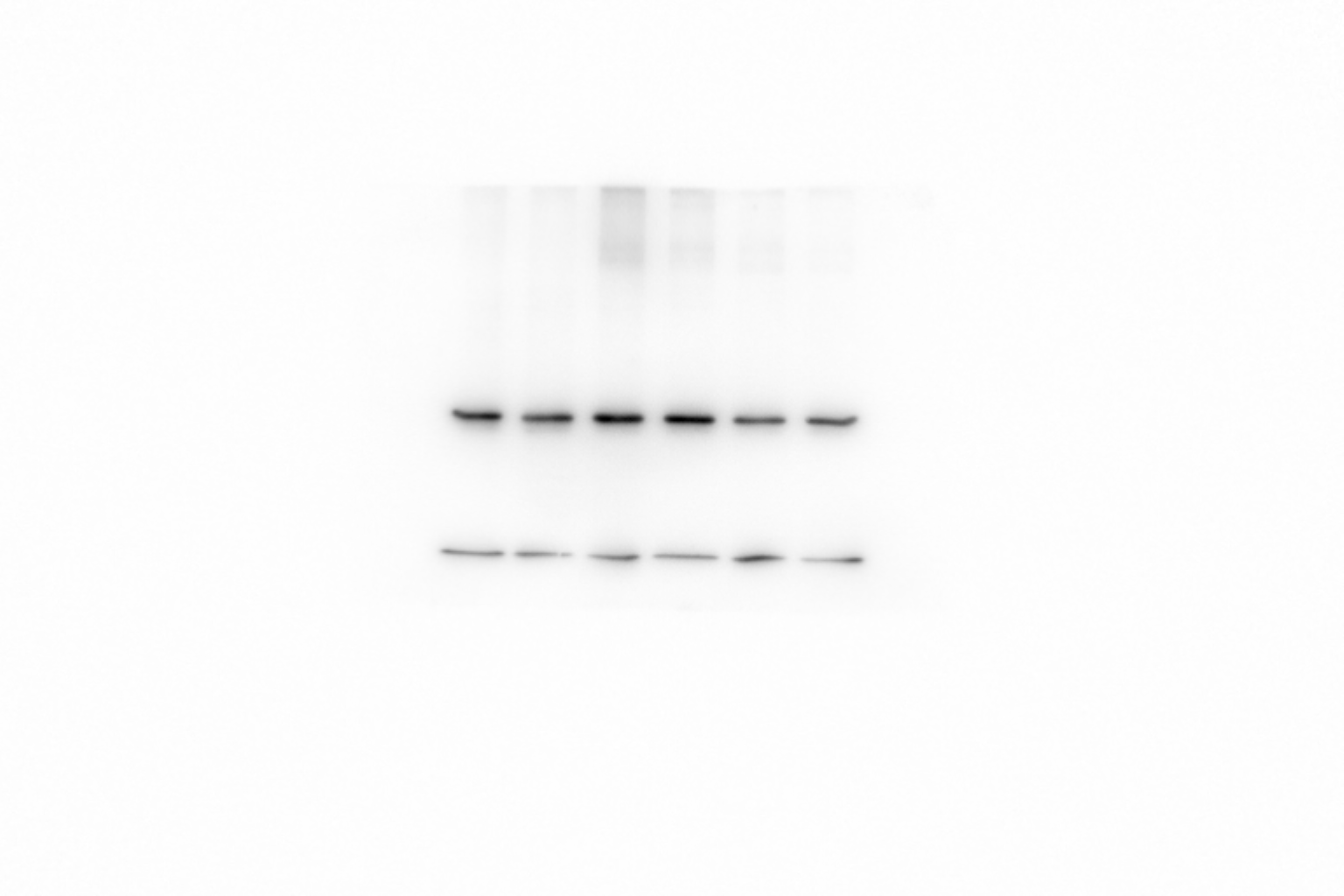

Supplement: Supplementary file 5 — Source data Fig. 3 [file 44318_2025_572_MOESM5_ESM.zip › Figure 3/Figure 3B/MIAPACA2 B ACTIN 1 SEC.gel]

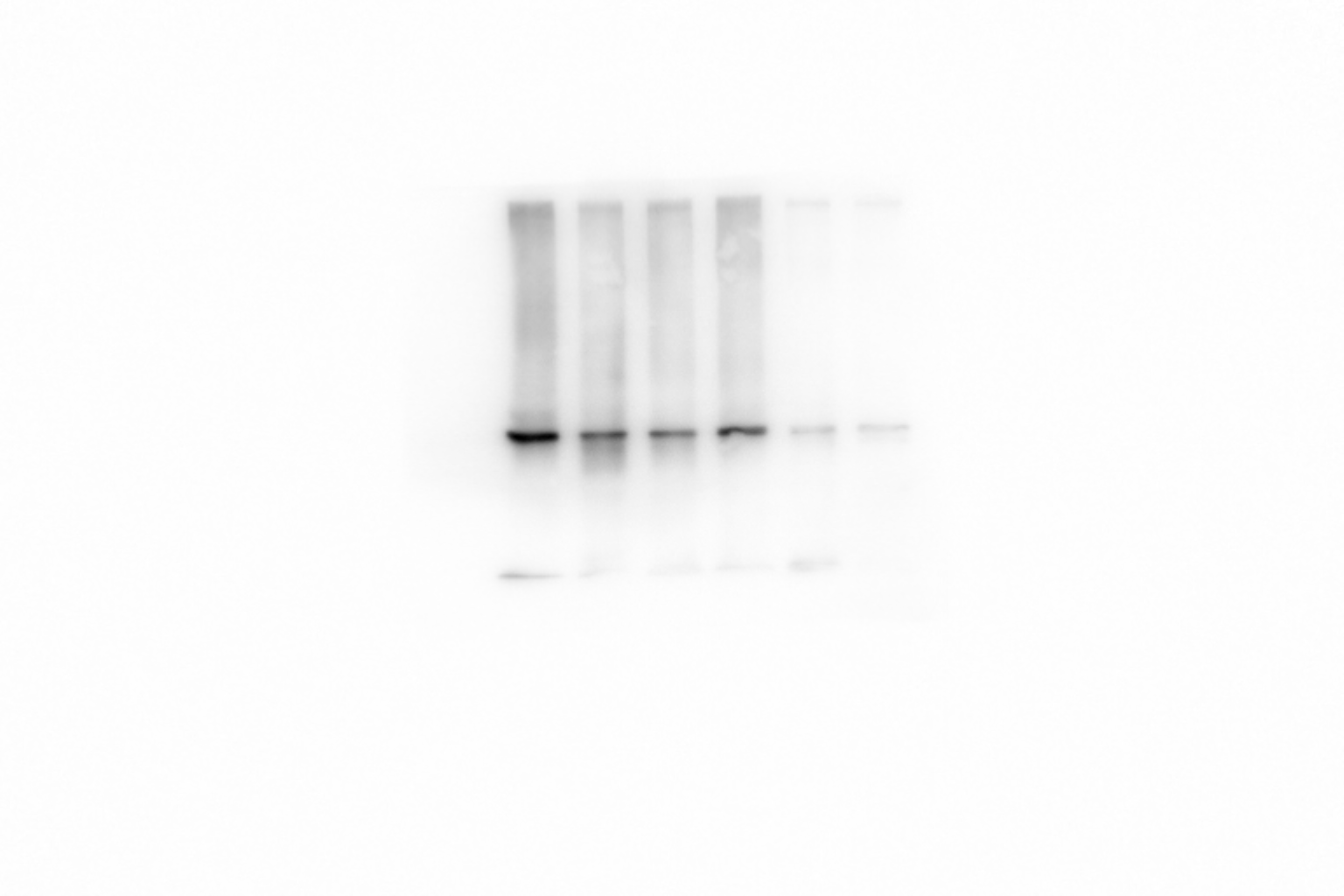

Supplement: Supplementary file 5 — Source data Fig. 3 [file 44318_2025_572_MOESM5_ESM.zip › Figure 3/Figure 3B/MIAPACA2 VIVIT 1 SEC.gel]

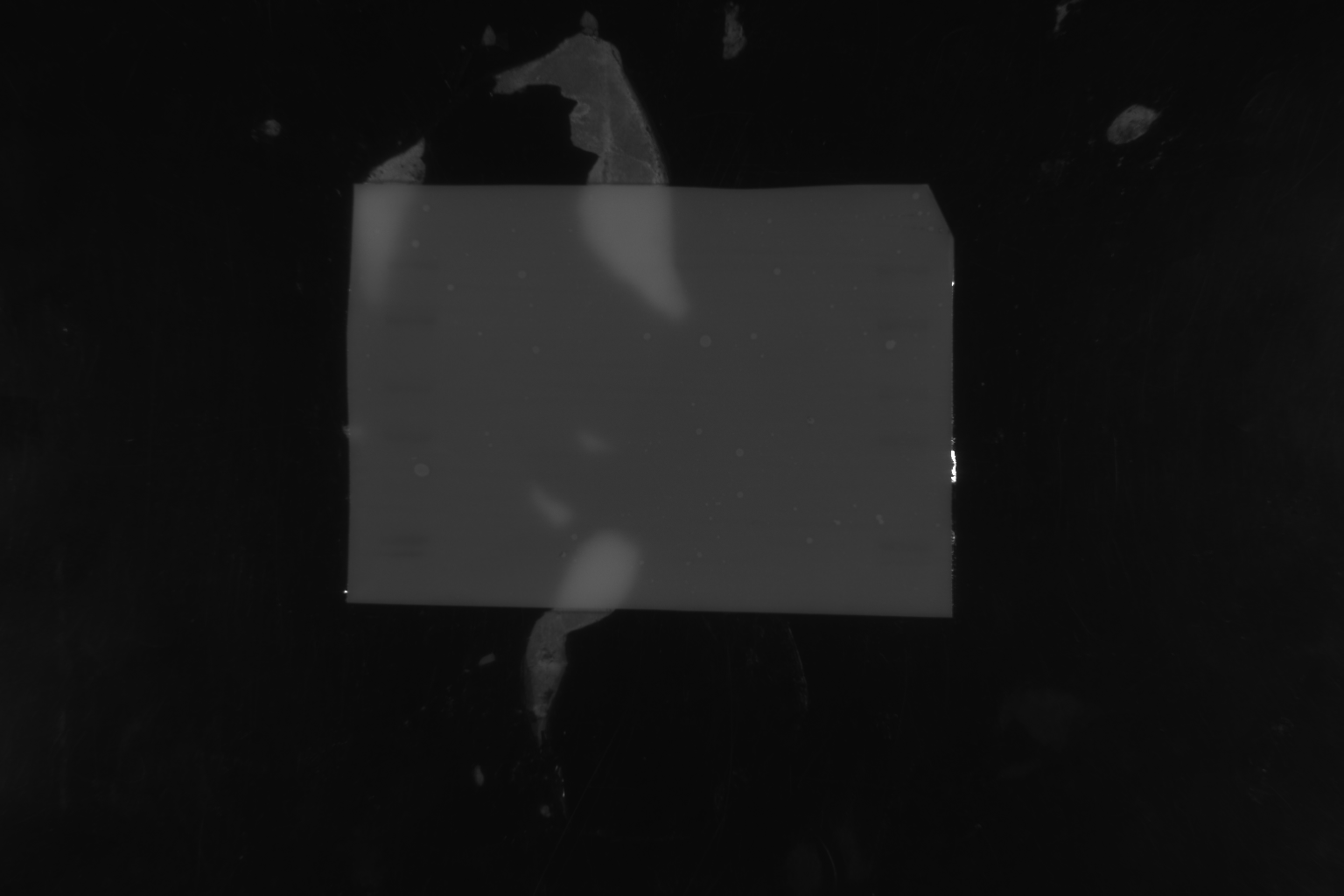

Supplement: Supplementary file 5 — Source data Fig. 3 [file 44318_2025_572_MOESM5_ESM.zip › Figure 3/Figure 3B/V_MIAPACA2 B ACTIN 1 SEC.gel]

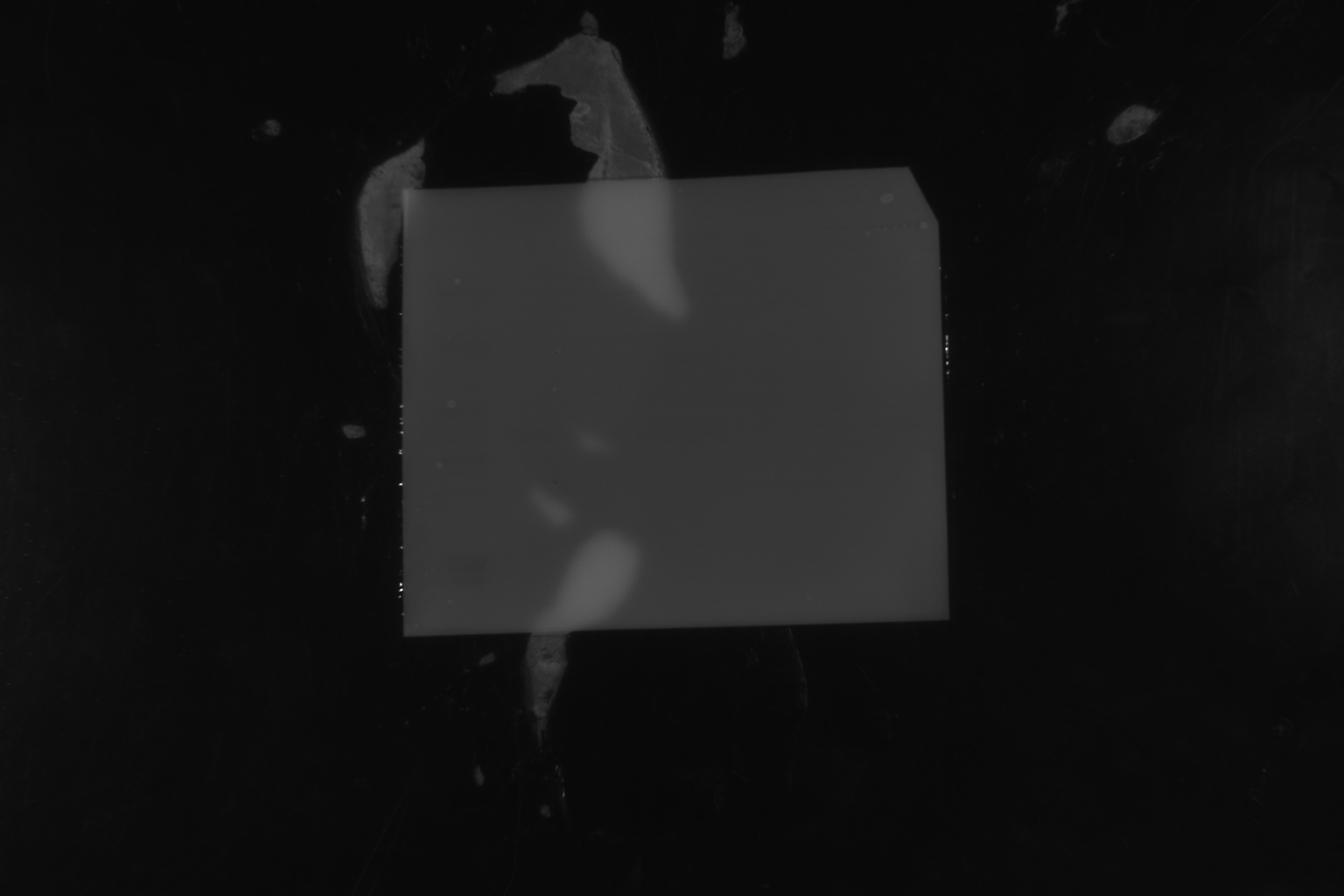

Supplement: Supplementary file 5 — Source data Fig. 3 [file 44318_2025_572_MOESM5_ESM.zip › Figure 3/Figure 3B/V_MIAPACA2 VIVIT 1 SEC.gel]

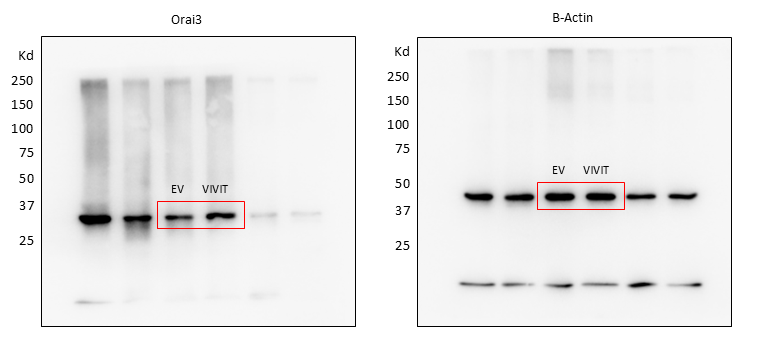

Supplement: Supplementary file 5 — Source data Fig. 3 [file 44318_2025_572_MOESM5_ESM.zip › Figure 3/Figure 3G/Figure 3G.png]

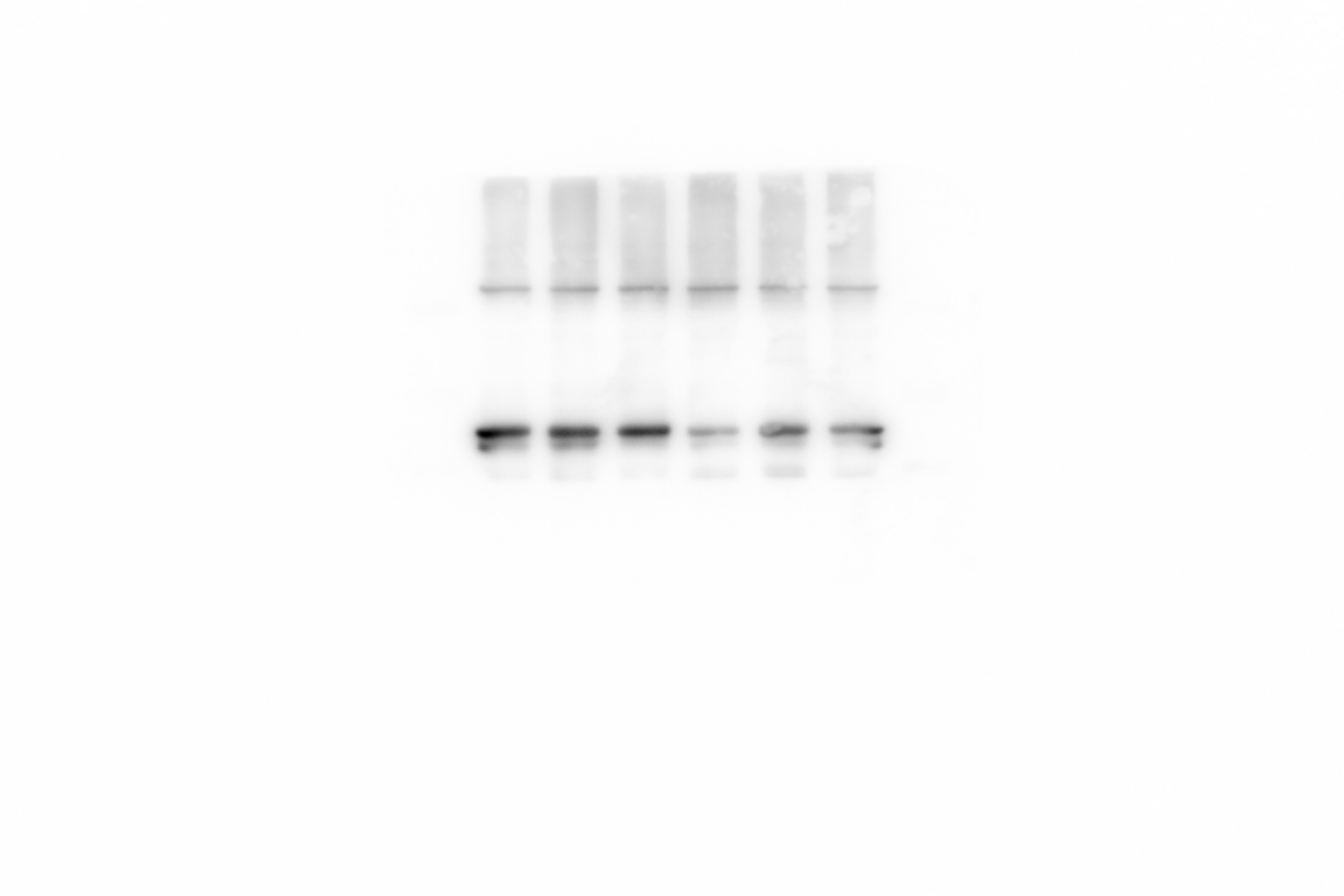

Supplement: Supplementary file 5 — Source data Fig. 3 [file 44318_2025_572_MOESM5_ESM.zip › Figure 3/Figure 3L/B ACTIN 2 SEC.gel]

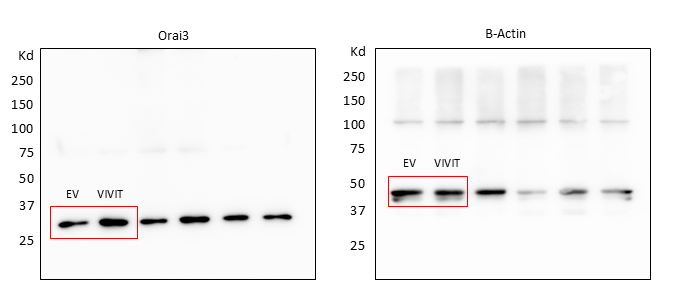

Supplement: Supplementary file 5 — Source data Fig. 3 [file 44318_2025_572_MOESM5_ESM.zip › Figure 3/Figure 3L/Figure 3L.png]

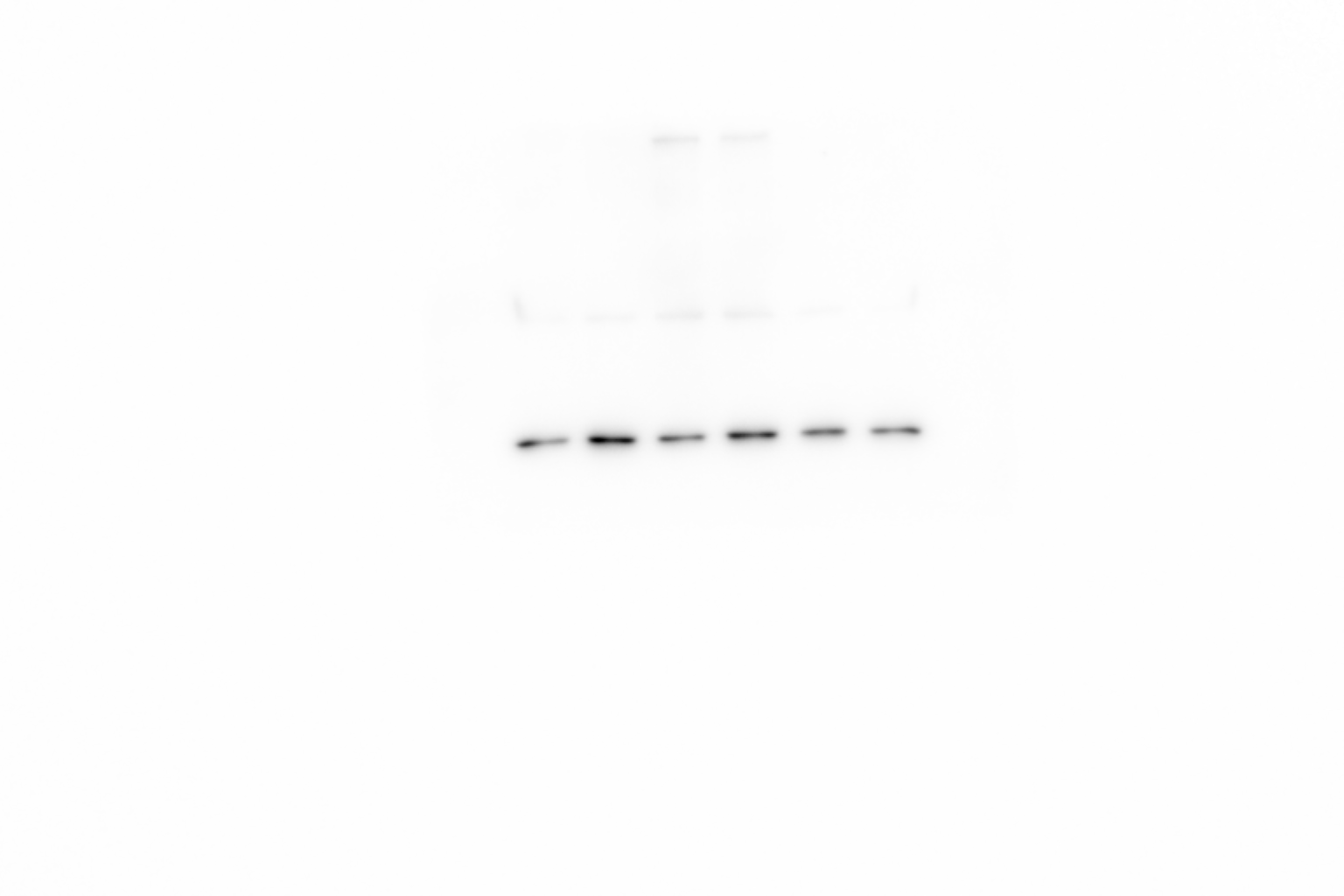

Supplement: Supplementary file 5 — Source data Fig. 3 [file 44318_2025_572_MOESM5_ESM.zip › Figure 3/Figure 3L/ORAI 3 8 SEC.gel]

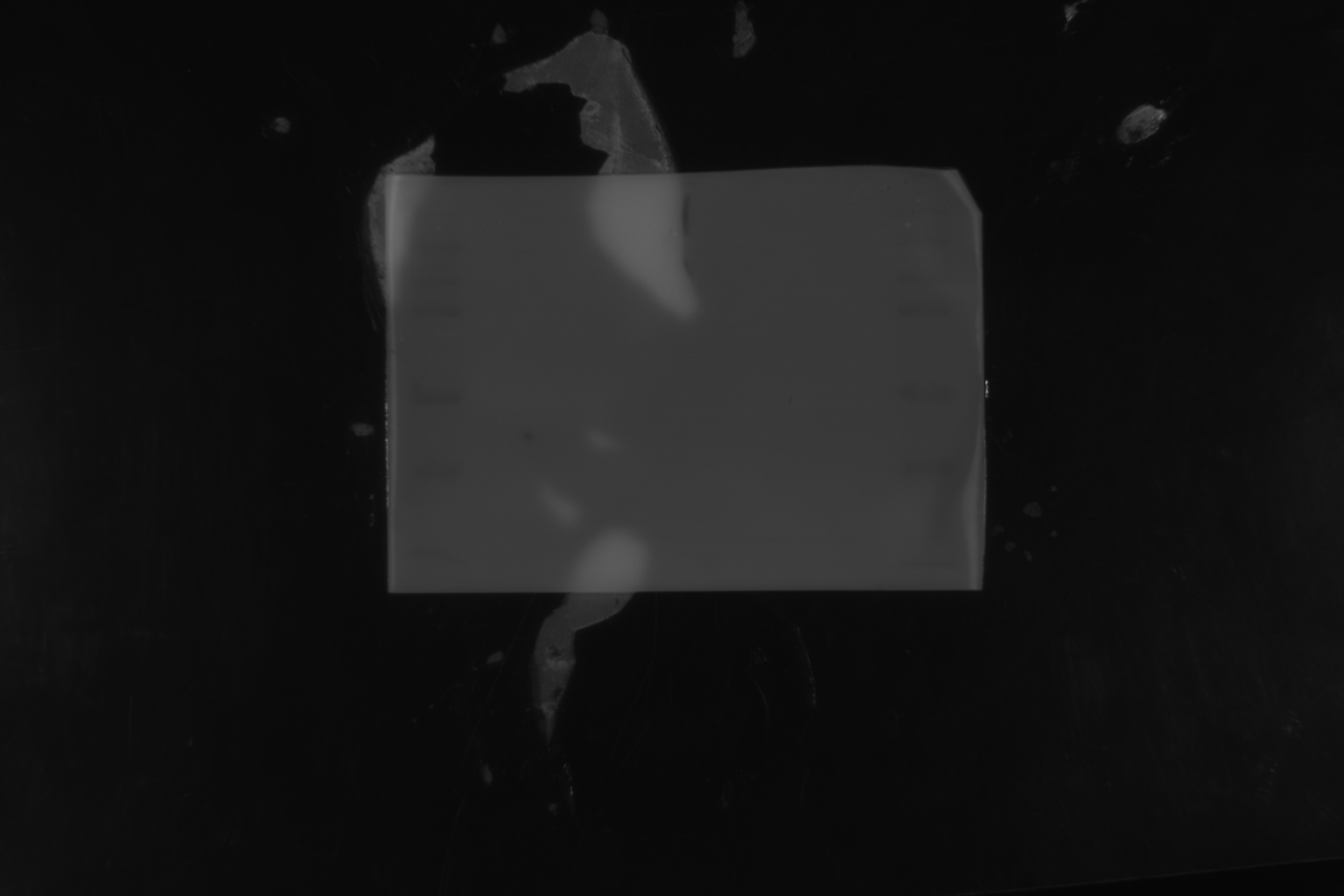

Supplement: Supplementary file 5 — Source data Fig. 3 [file 44318_2025_572_MOESM5_ESM.zip › Figure 3/Figure 3L/V_B ACTIN 2 SEC.gel]

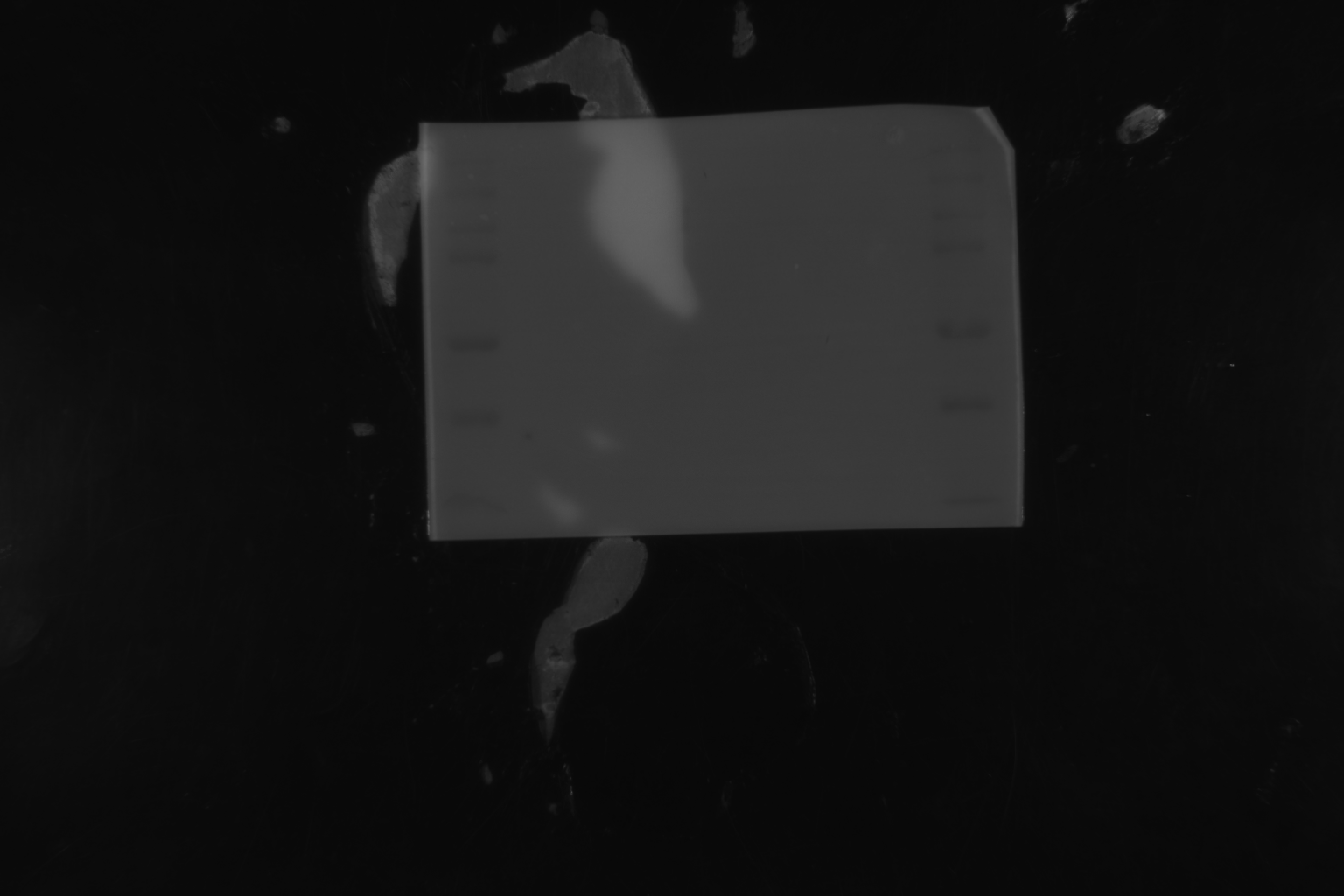

Supplement: Supplementary file 5 — Source data Fig. 3 [file 44318_2025_572_MOESM5_ESM.zip › Figure 3/Figure 3L/V_ORAI 3 8 SEC.gel]

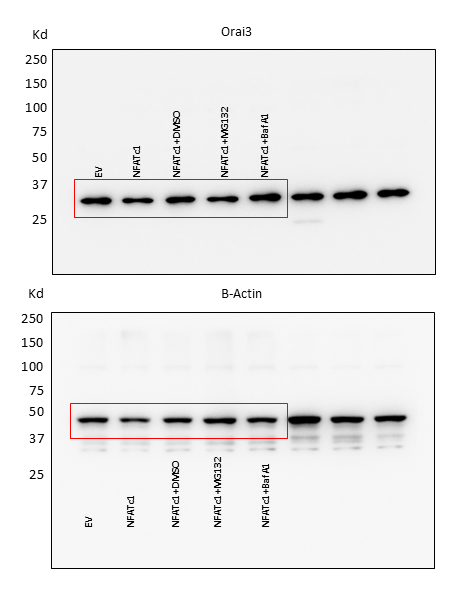

Supplement: Supplementary file 6 — Source data Fig. 4 [file 44318_2025_572_MOESM6_ESM.zip › Figure 4/Figure 4A/Figure 4A.png]

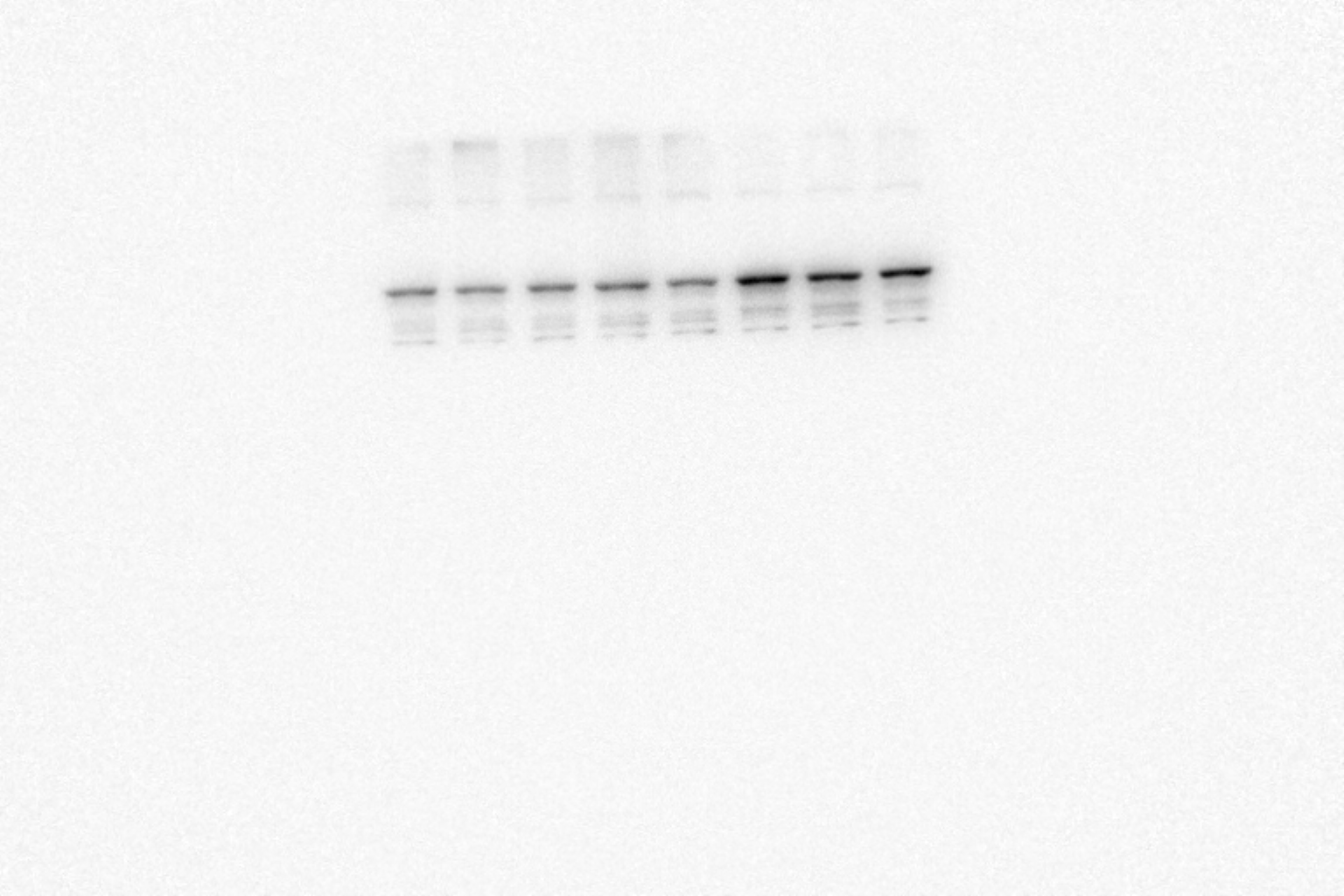

Supplement: Supplementary file 6 — Source data Fig. 4 [file 44318_2025_572_MOESM6_ESM.zip › Figure 4/Figure 4A/PANC1 B ACTIN 0.5 SEC.gel]

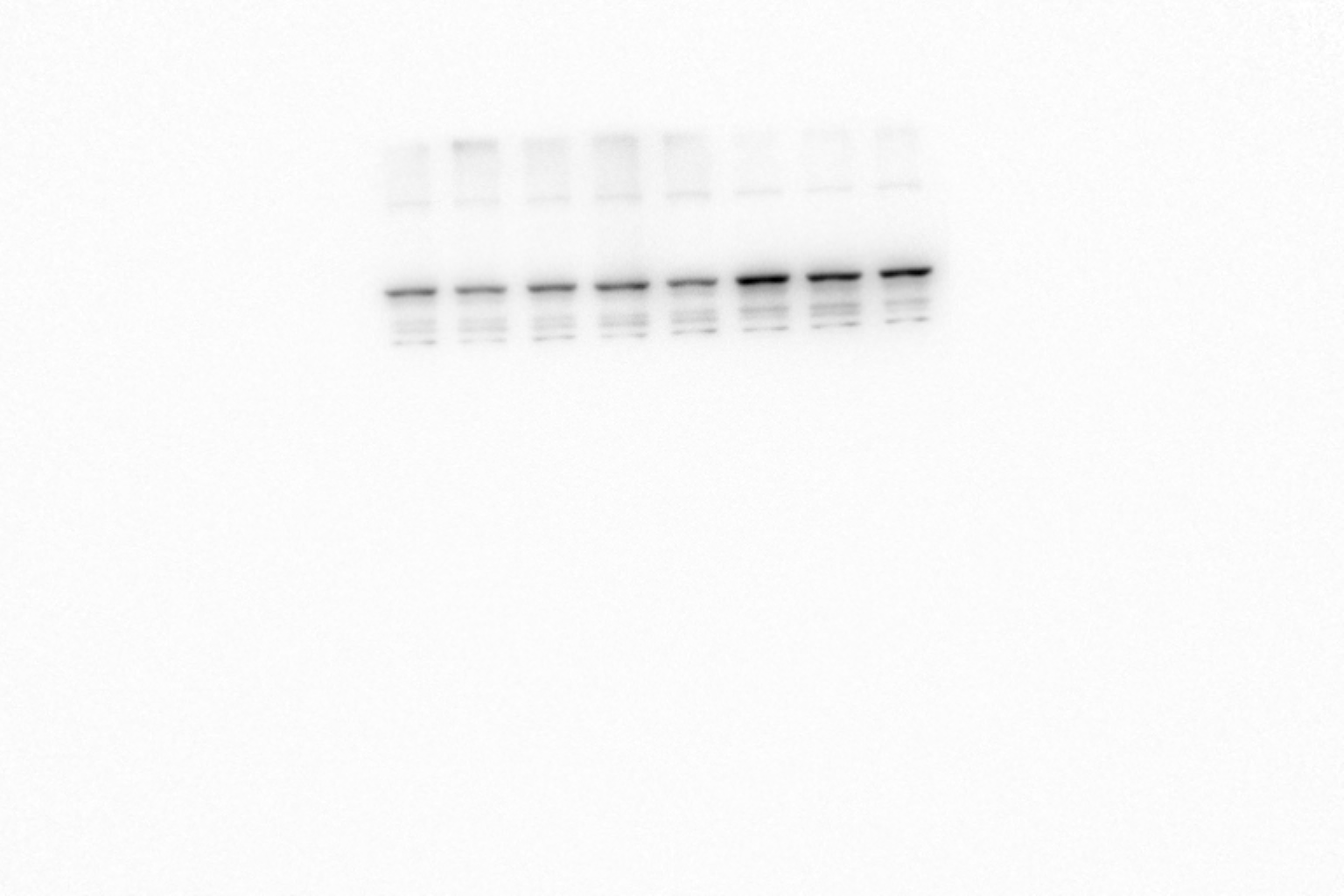

Supplement: Supplementary file 6 — Source data Fig. 4 [file 44318_2025_572_MOESM6_ESM.zip › Figure 4/Figure 4A/PANC1 B ACTIN 1 SEC.gel]

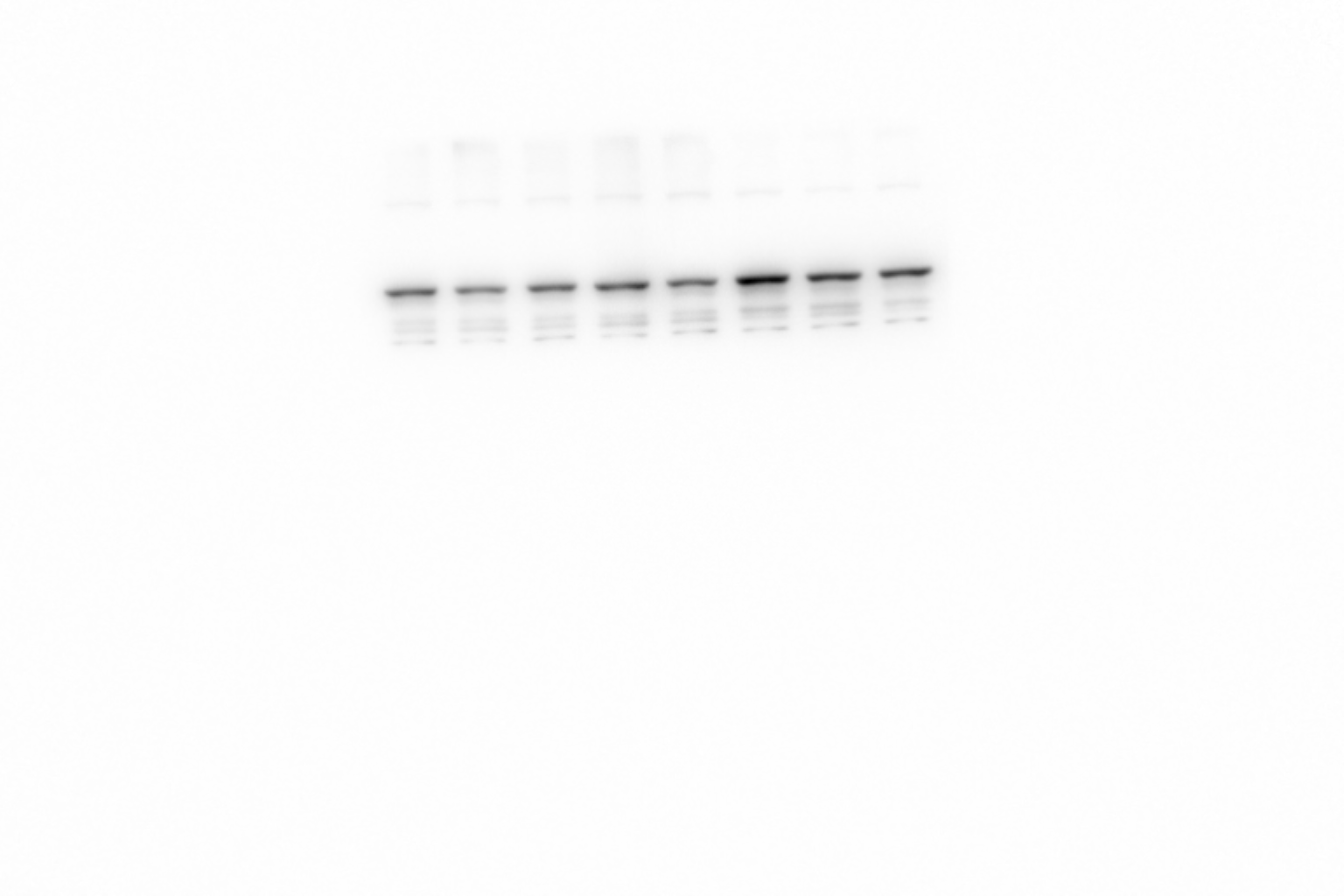

Supplement: Supplementary file 6 — Source data Fig. 4 [file 44318_2025_572_MOESM6_ESM.zip › Figure 4/Figure 4A/PANC1 B ACTIN 2 SEC.gel]

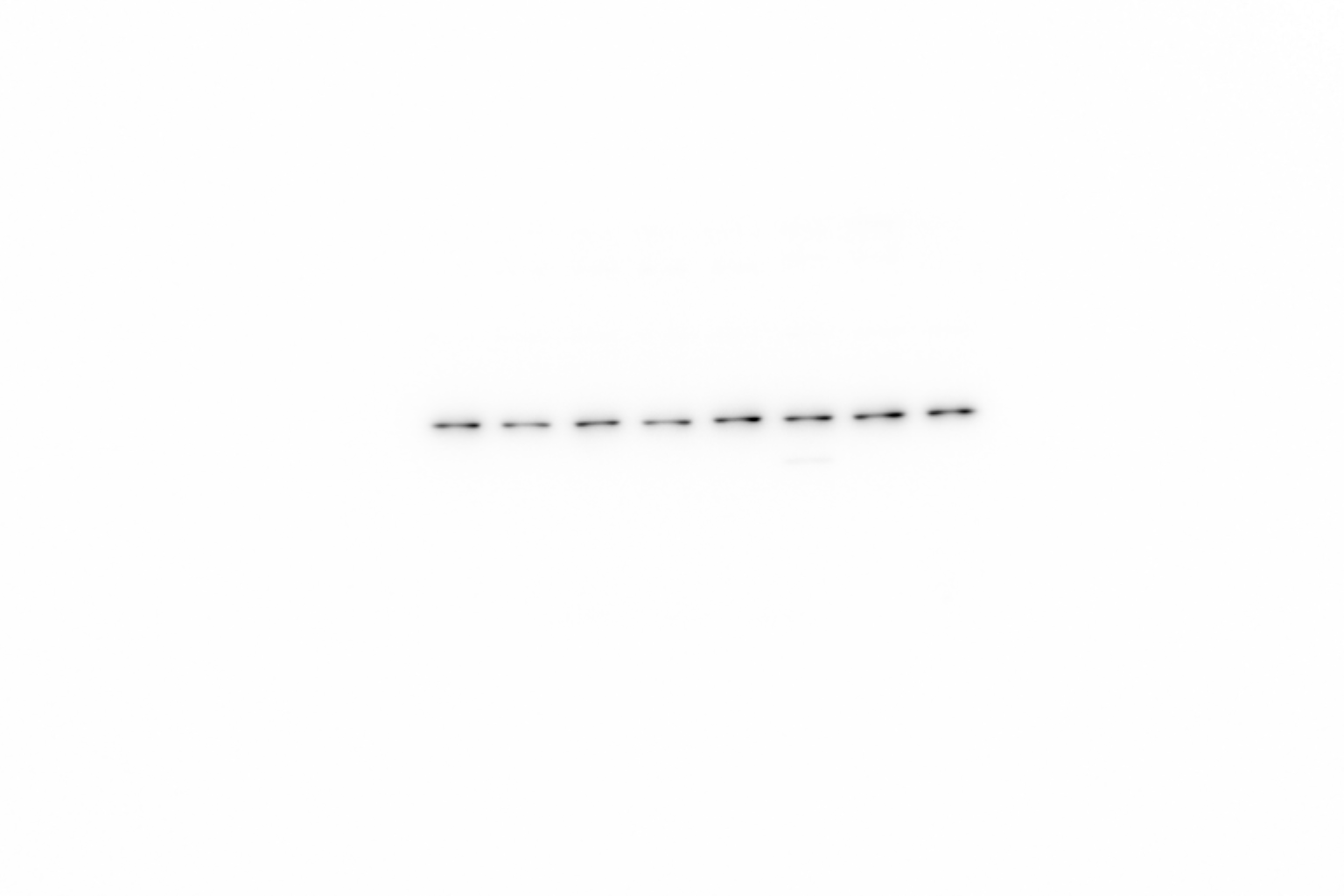

Supplement: Supplementary file 6 — Source data Fig. 4 [file 44318_2025_572_MOESM6_ESM.zip › Figure 4/Figure 4A/PANC1 ORAI 3 4 SEC.gel]

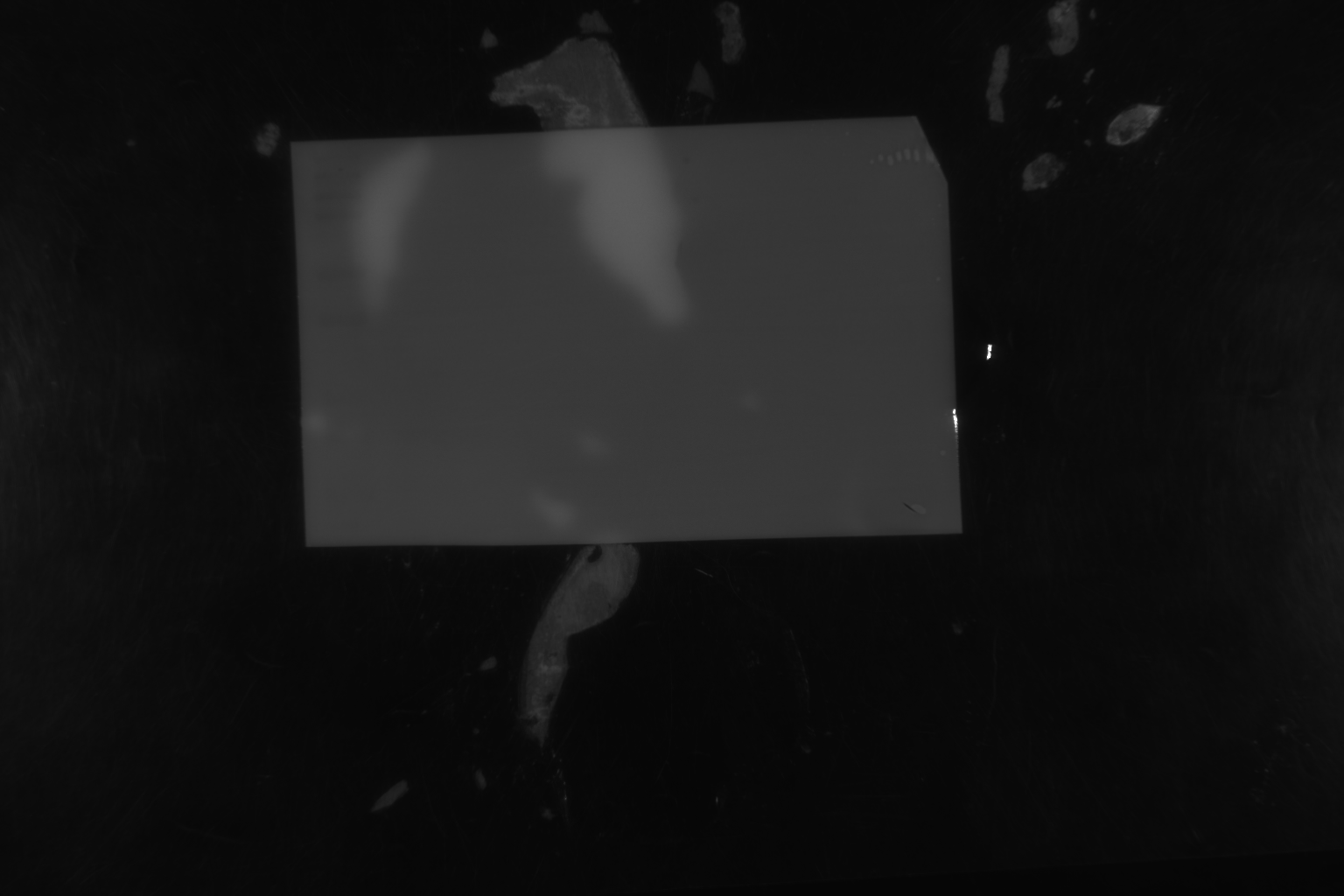

Supplement: Supplementary file 6 — Source data Fig. 4 [file 44318_2025_572_MOESM6_ESM.zip › Figure 4/Figure 4A/V_PANC1 B ACTIN 0.5 SEC.gel]

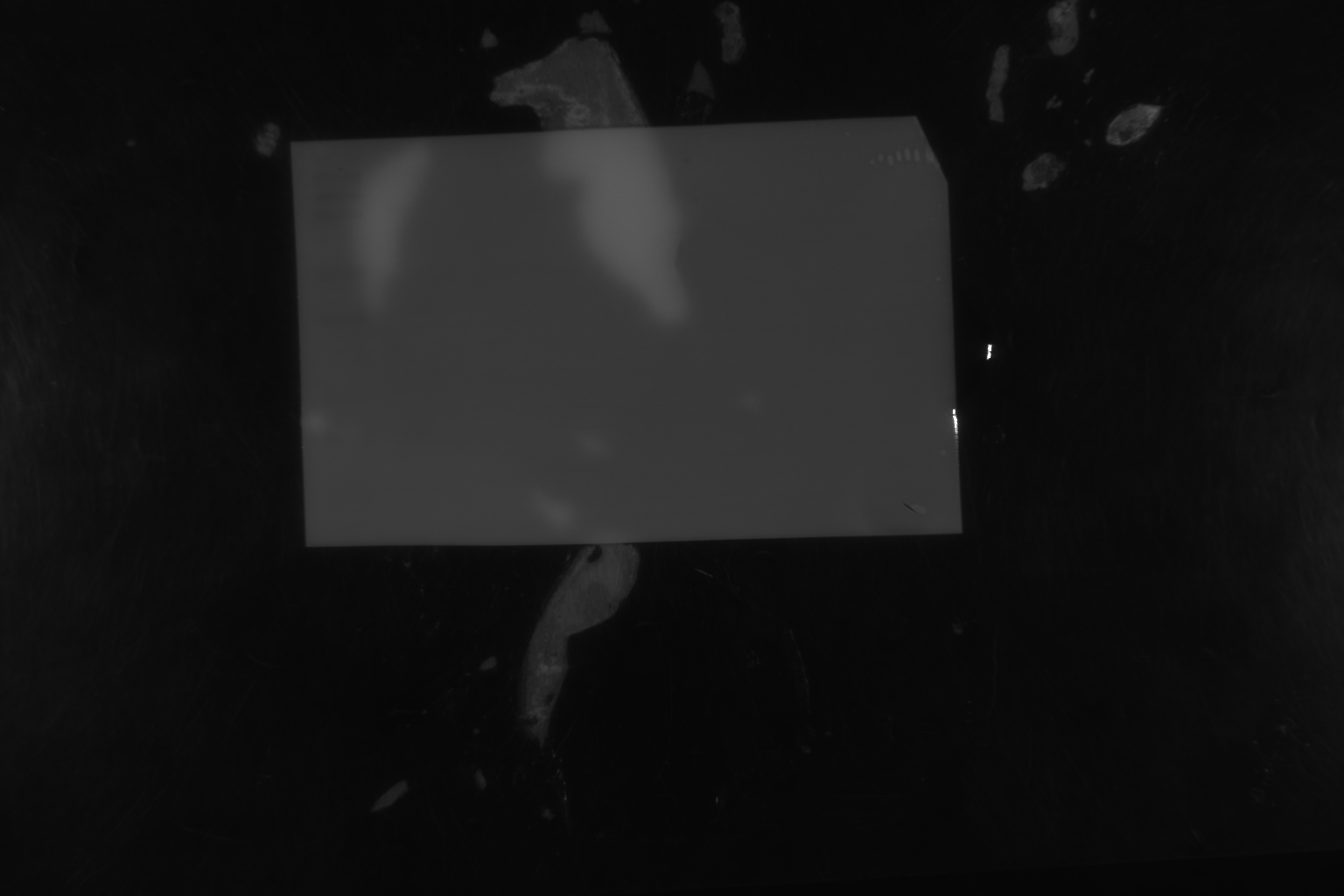

Supplement: Supplementary file 6 — Source data Fig. 4 [file 44318_2025_572_MOESM6_ESM.zip › Figure 4/Figure 4A/V_PANC1 B ACTIN 1 SEC.gel]

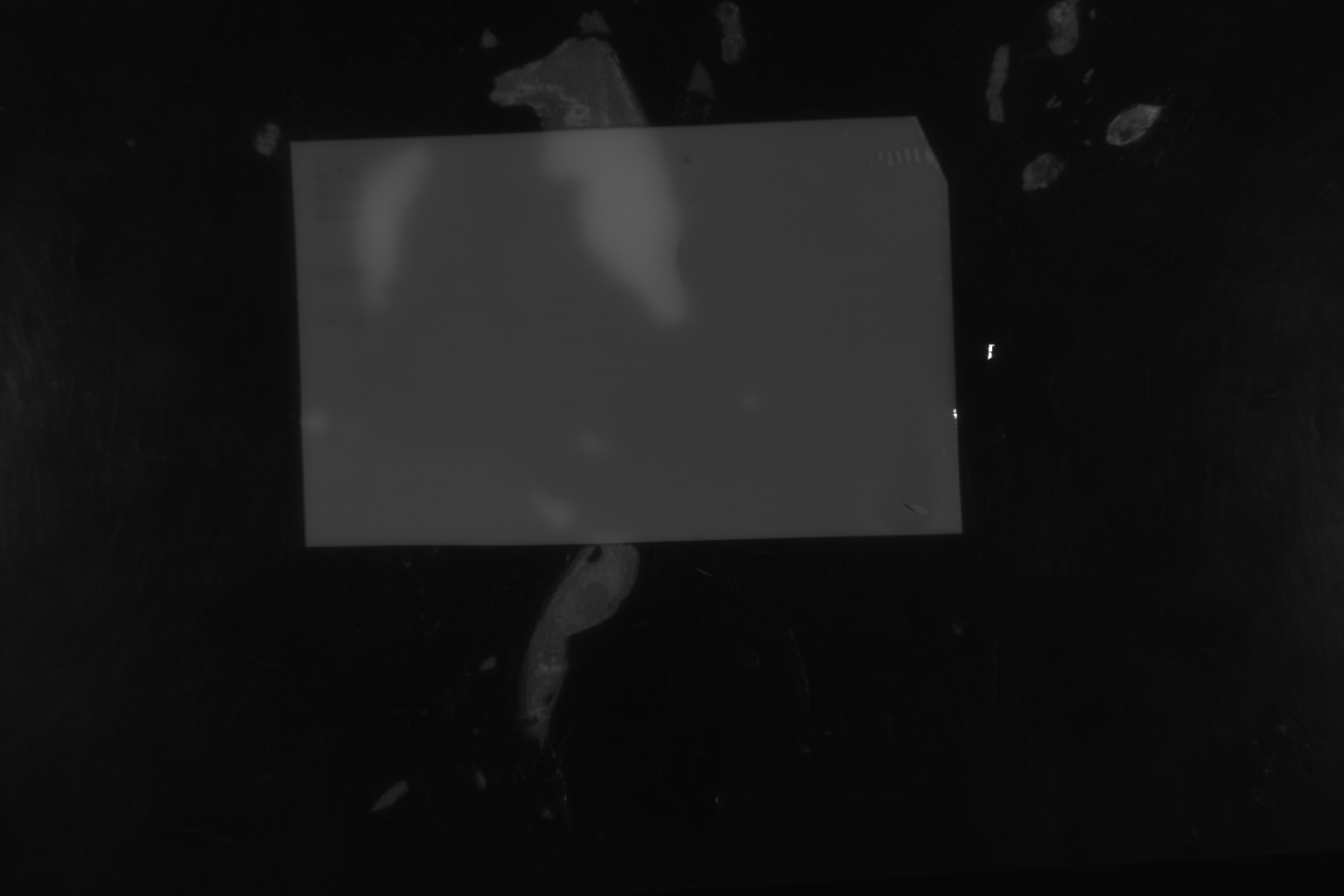

Supplement: Supplementary file 6 — Source data Fig. 4 [file 44318_2025_572_MOESM6_ESM.zip › Figure 4/Figure 4A/V_PANC1 B ACTIN 2 SEC.gel]

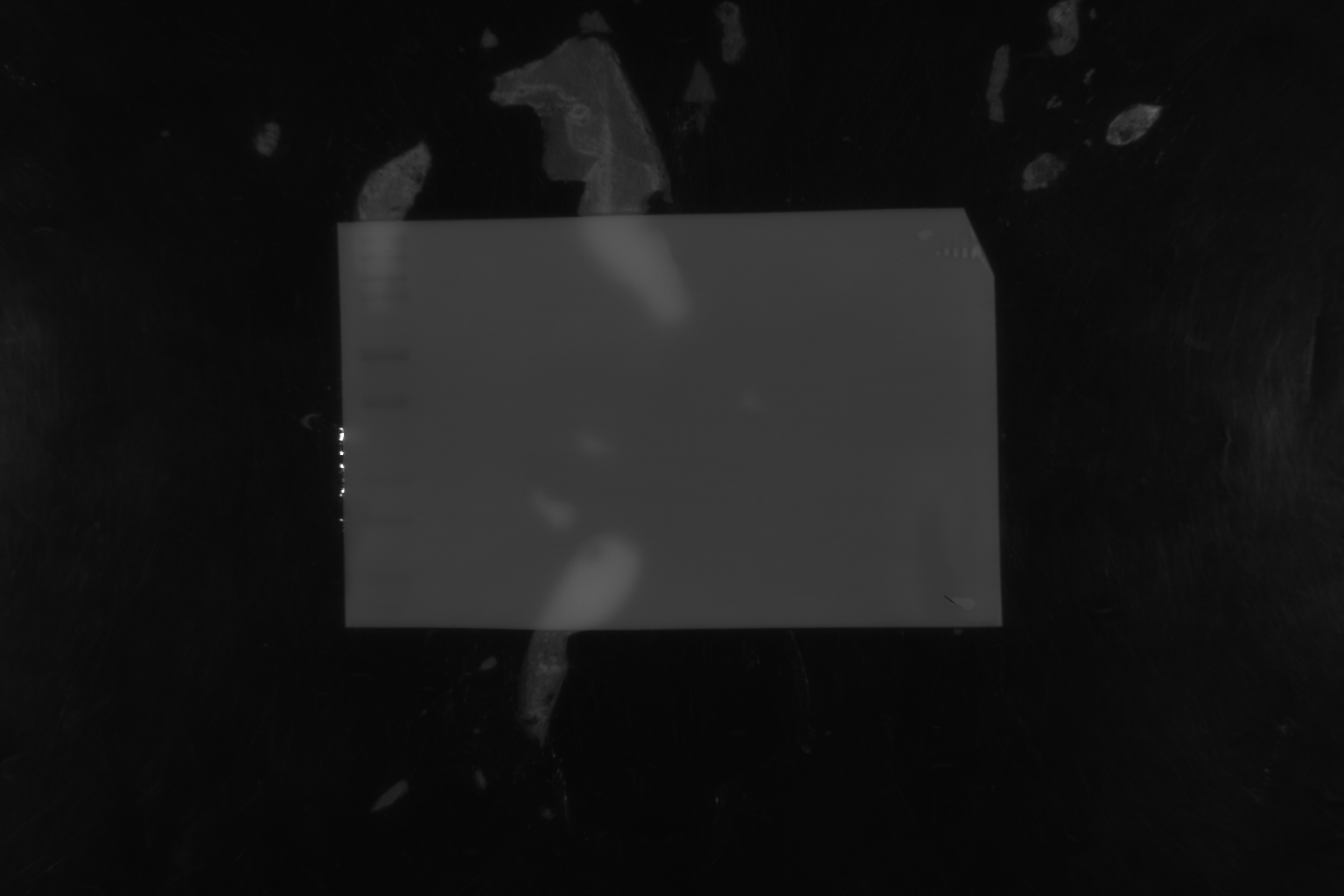

Supplement: Supplementary file 6 — Source data Fig. 4 [file 44318_2025_572_MOESM6_ESM.zip › Figure 4/Figure 4A/V_PANC1 ORAI 3 4 SEC.gel]

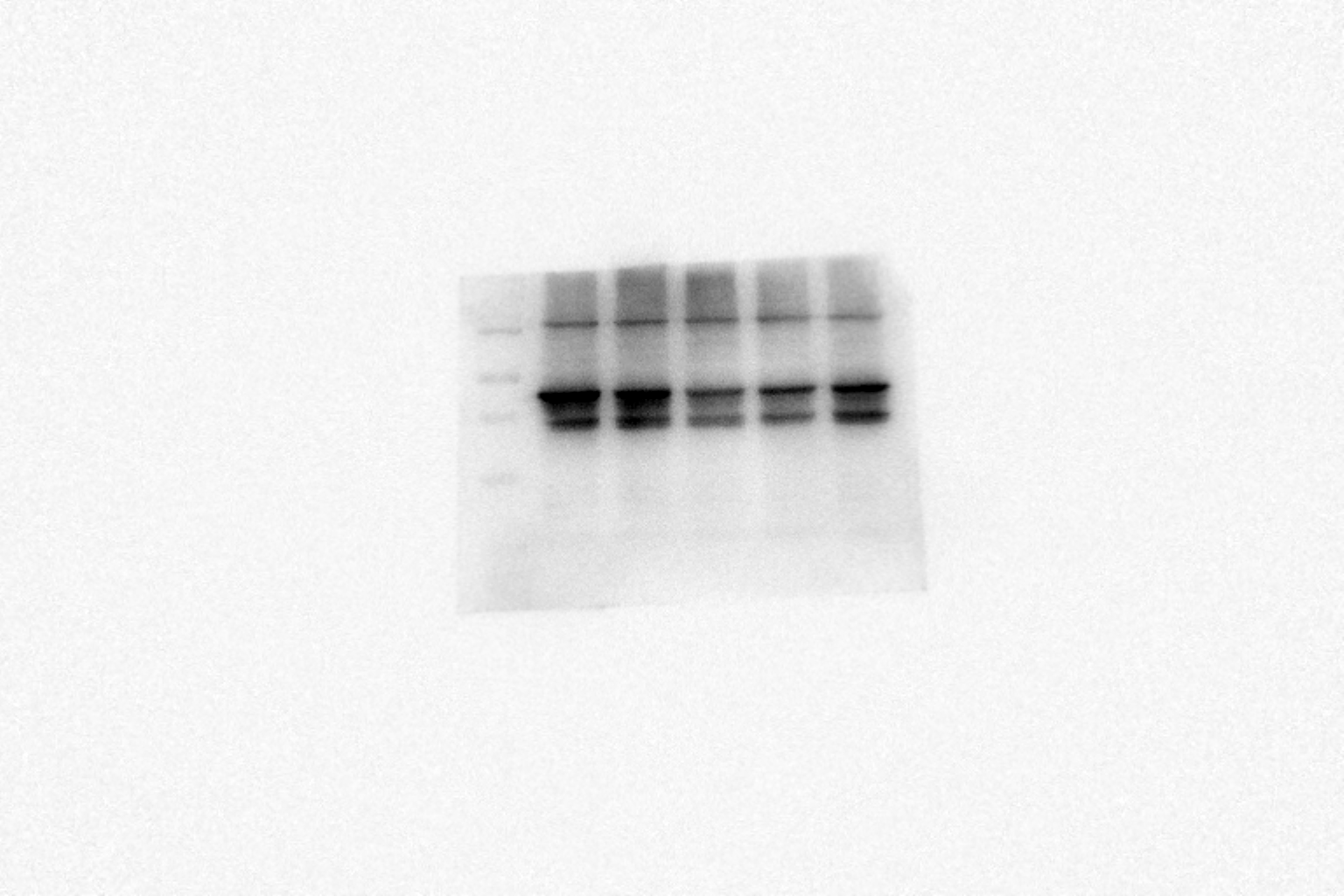

Supplement: Supplementary file 6 — Source data Fig. 4 [file 44318_2025_572_MOESM6_ESM.zip › Figure 4/Figure 4C/C B ACTIN 0.125 SEC.gel]

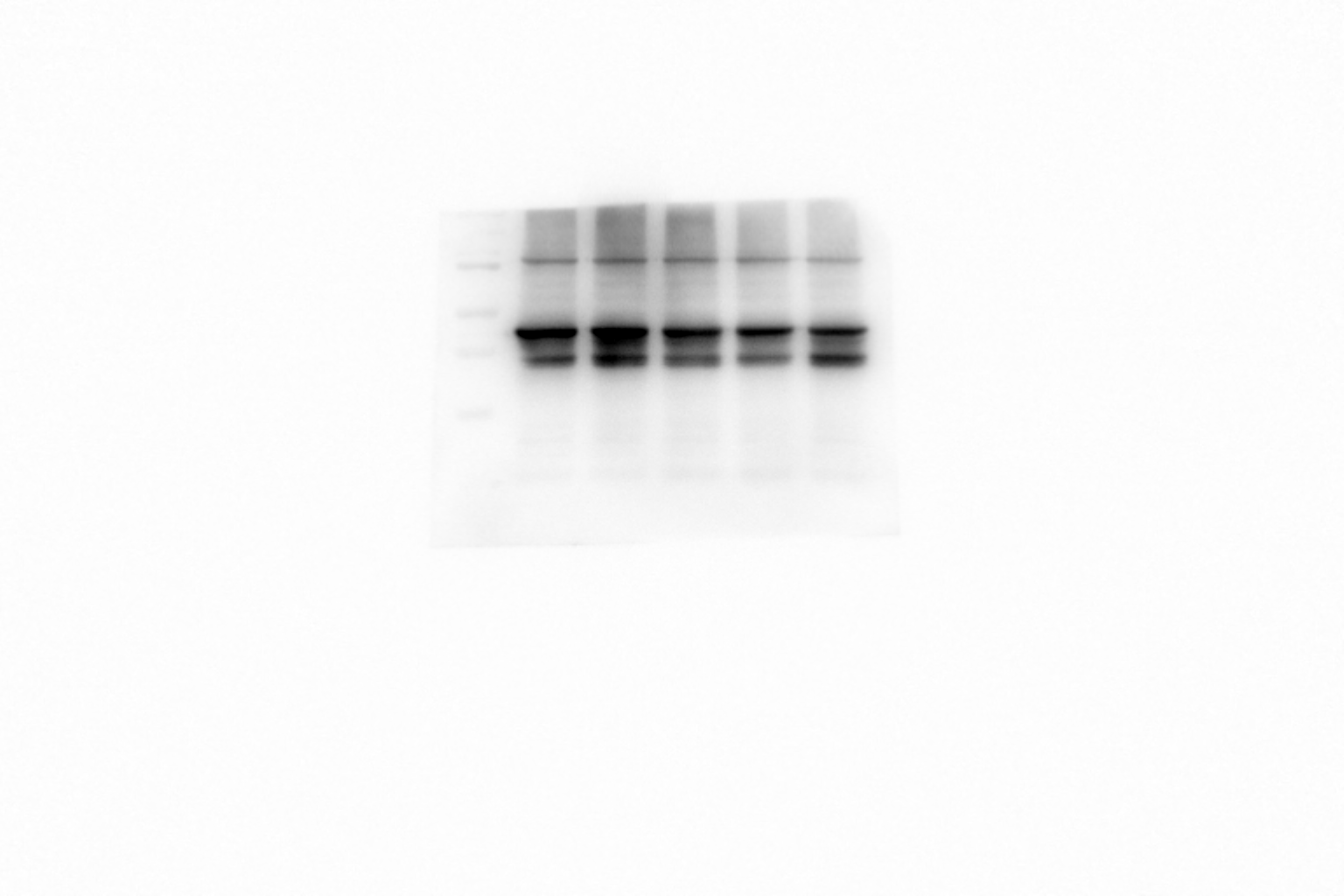

Supplement: Supplementary file 6 — Source data Fig. 4 [file 44318_2025_572_MOESM6_ESM.zip › Figure 4/Figure 4C/C B ACTIN 0.25 SEC.gel]

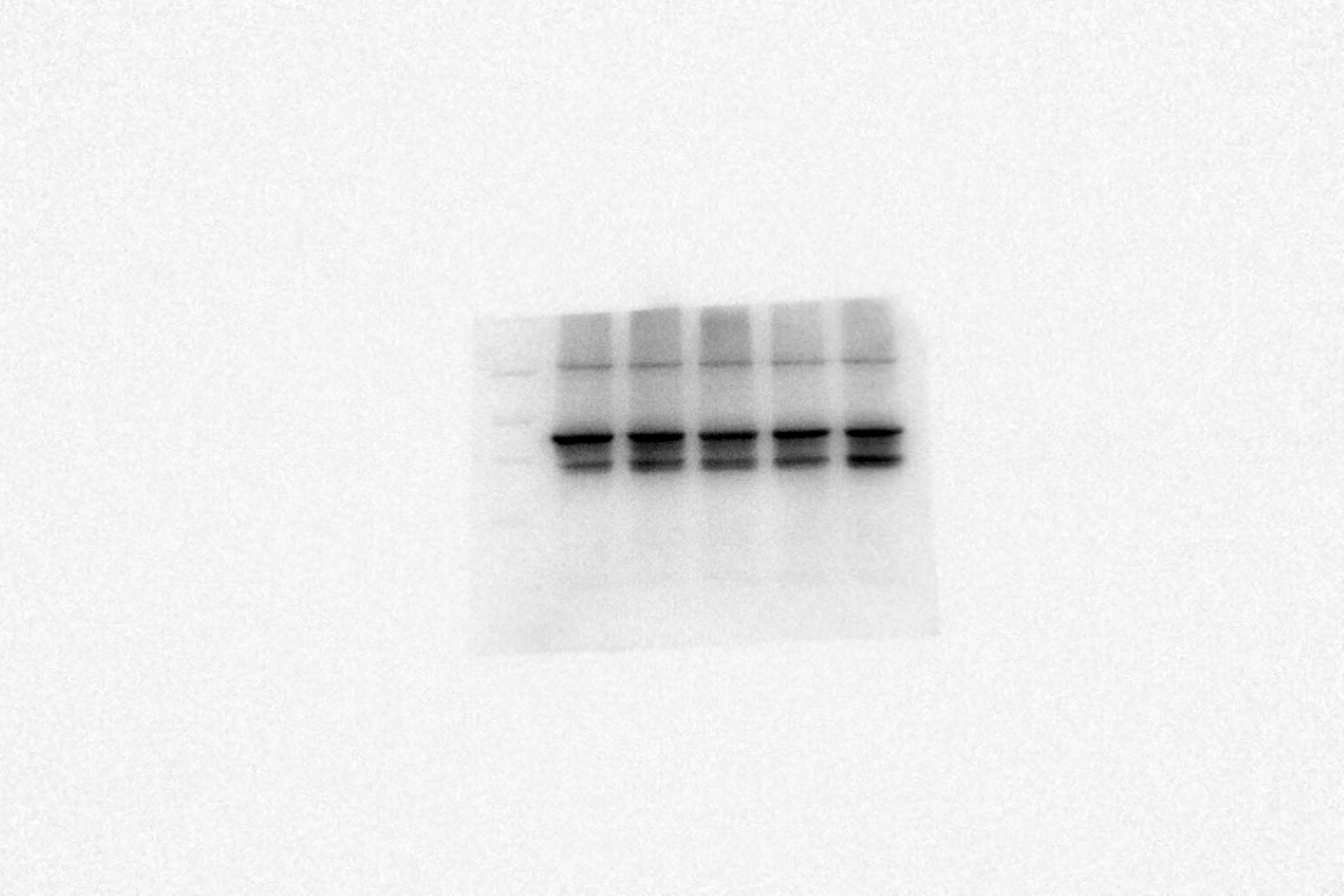

Supplement: Supplementary file 6 — Source data Fig. 4 [file 44318_2025_572_MOESM6_ESM.zip › Figure 4/Figure 4C/C B ACTIN 0.5 SEC.gel]

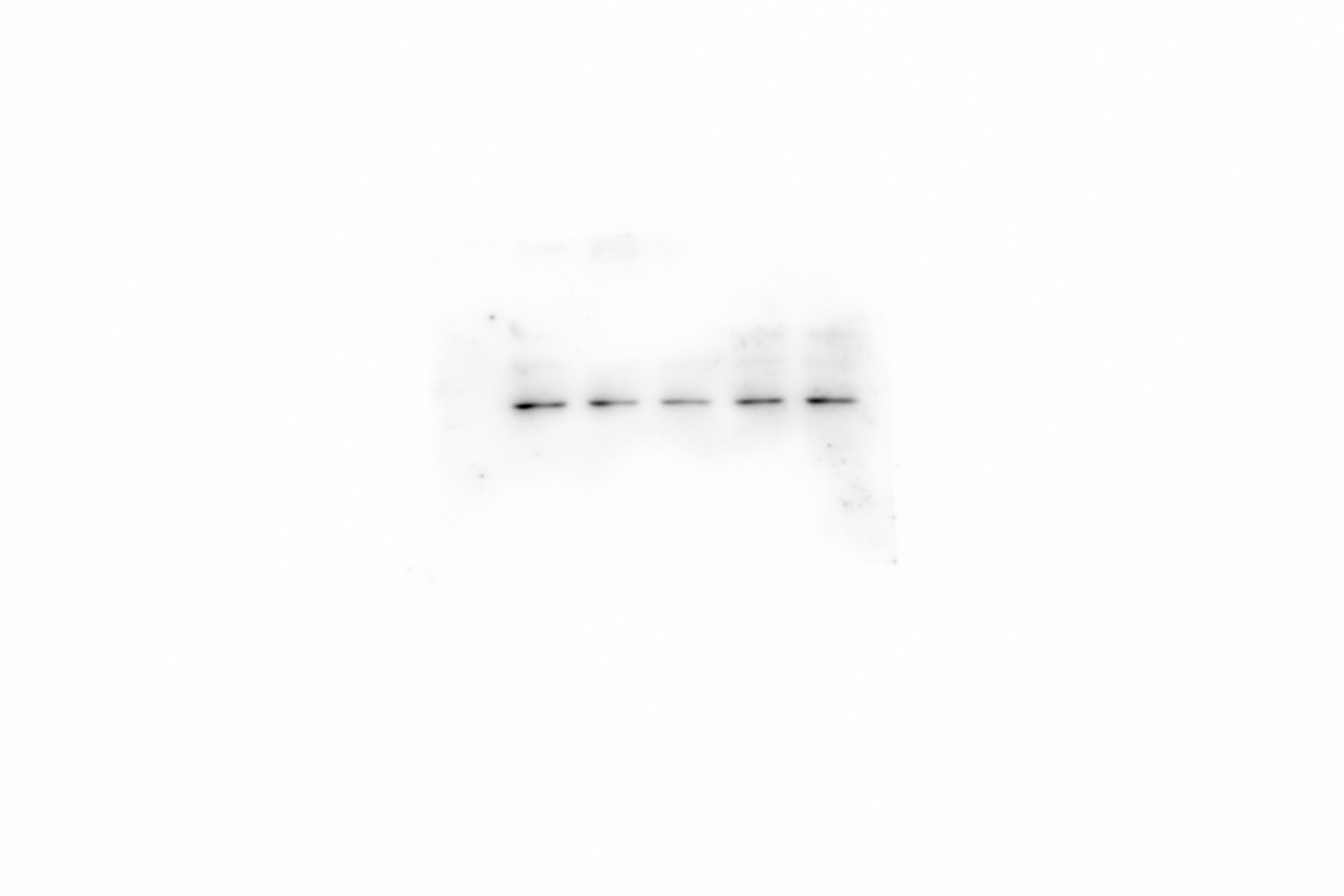

Supplement: Supplementary file 6 — Source data Fig. 4 [file 44318_2025_572_MOESM6_ESM.zip › Figure 4/Figure 4C/C ORAI 3 120 SEC.gel]

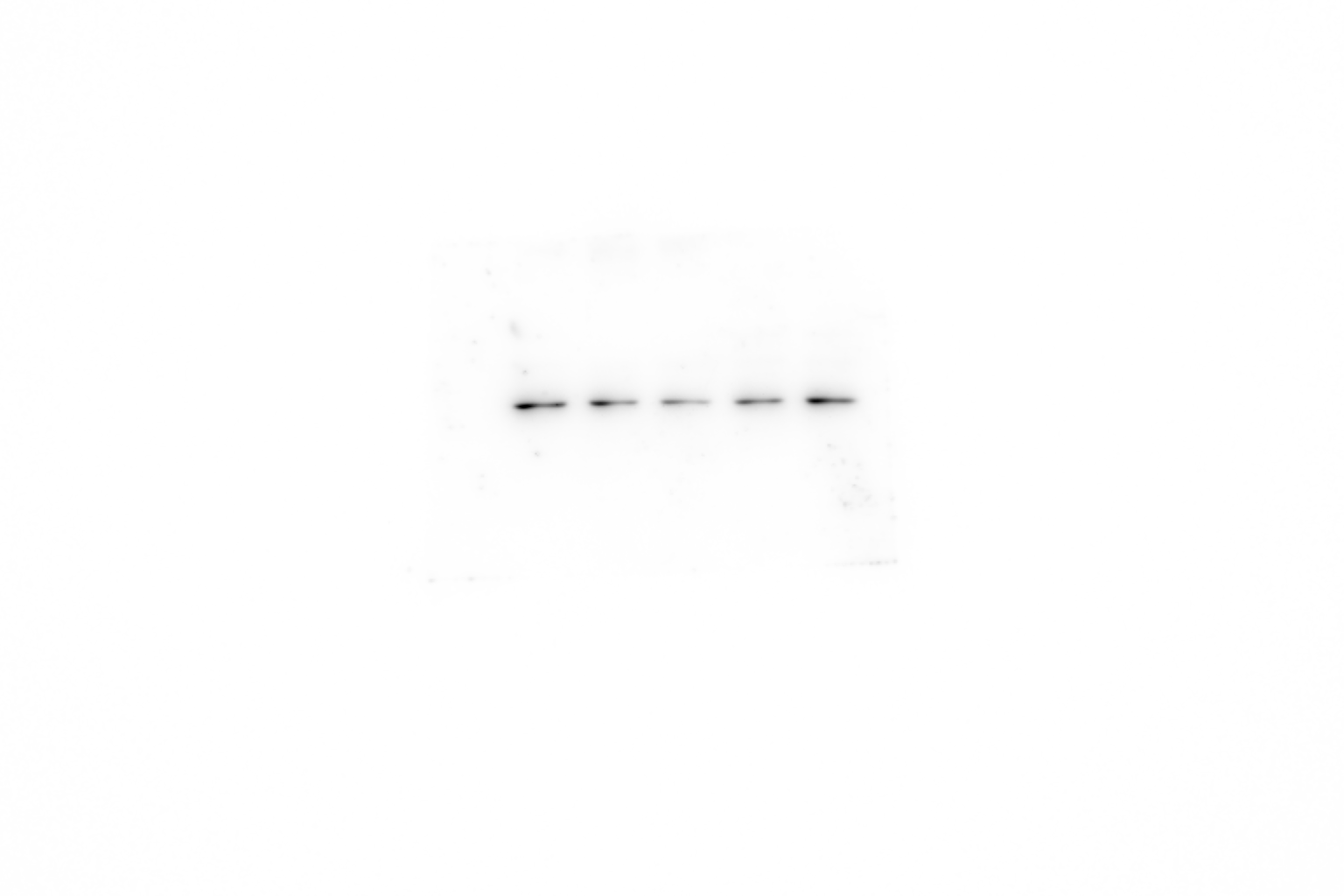

Supplement: Supplementary file 6 — Source data Fig. 4 [file 44318_2025_572_MOESM6_ESM.zip › Figure 4/Figure 4C/C ORAI 3 240 SEC.gel]

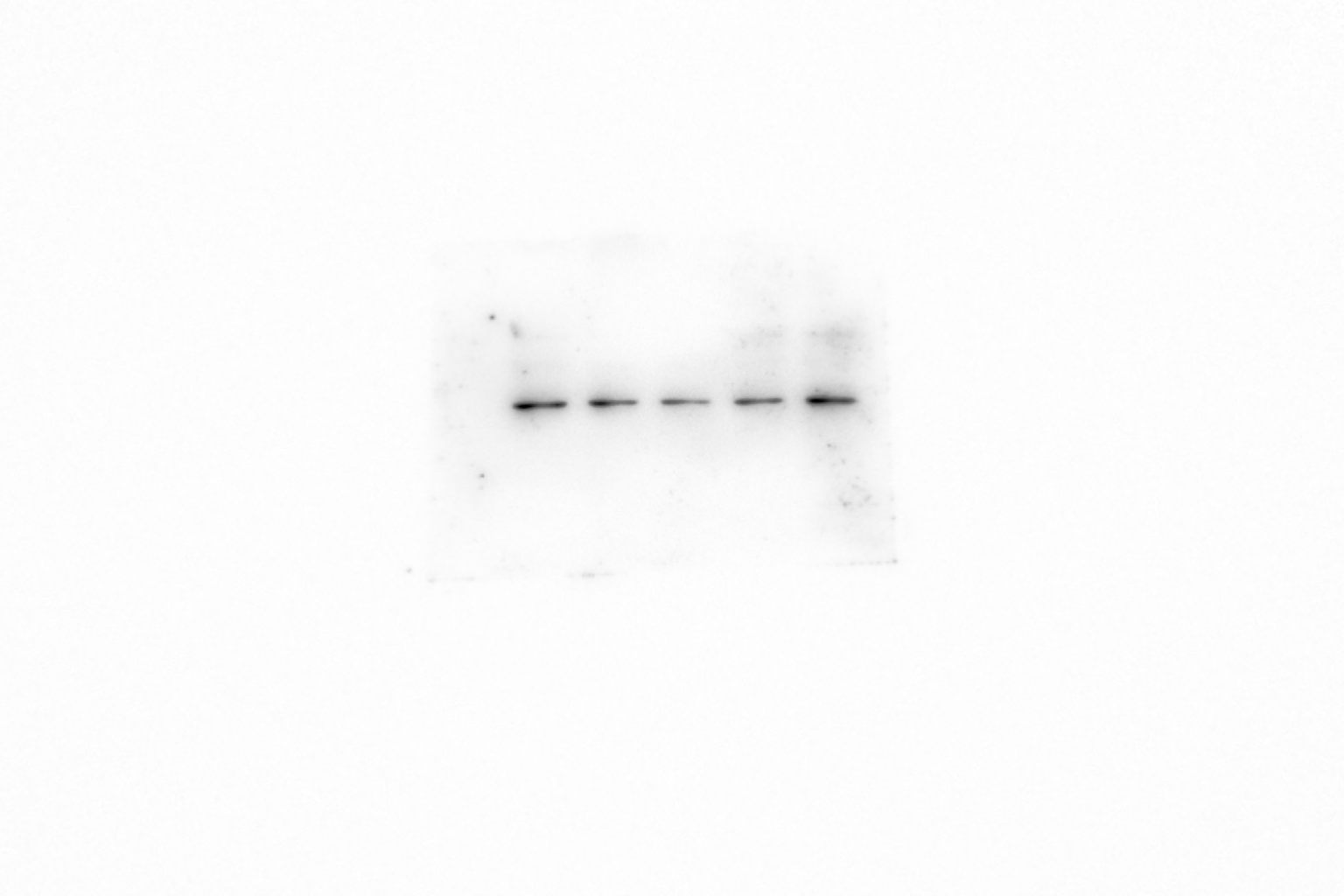

Supplement: Supplementary file 6 — Source data Fig. 4 [file 44318_2025_572_MOESM6_ESM.zip › Figure 4/Figure 4C/C ORAI 3 60 SEC.gel]

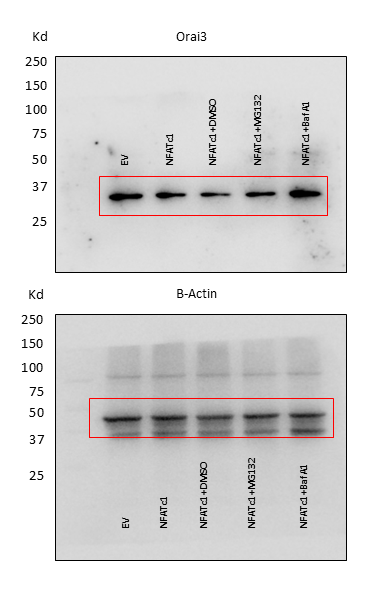

Supplement: Supplementary file 6 — Source data Fig. 4 [file 44318_2025_572_MOESM6_ESM.zip › Figure 4/Figure 4C/Figure 4C.png]

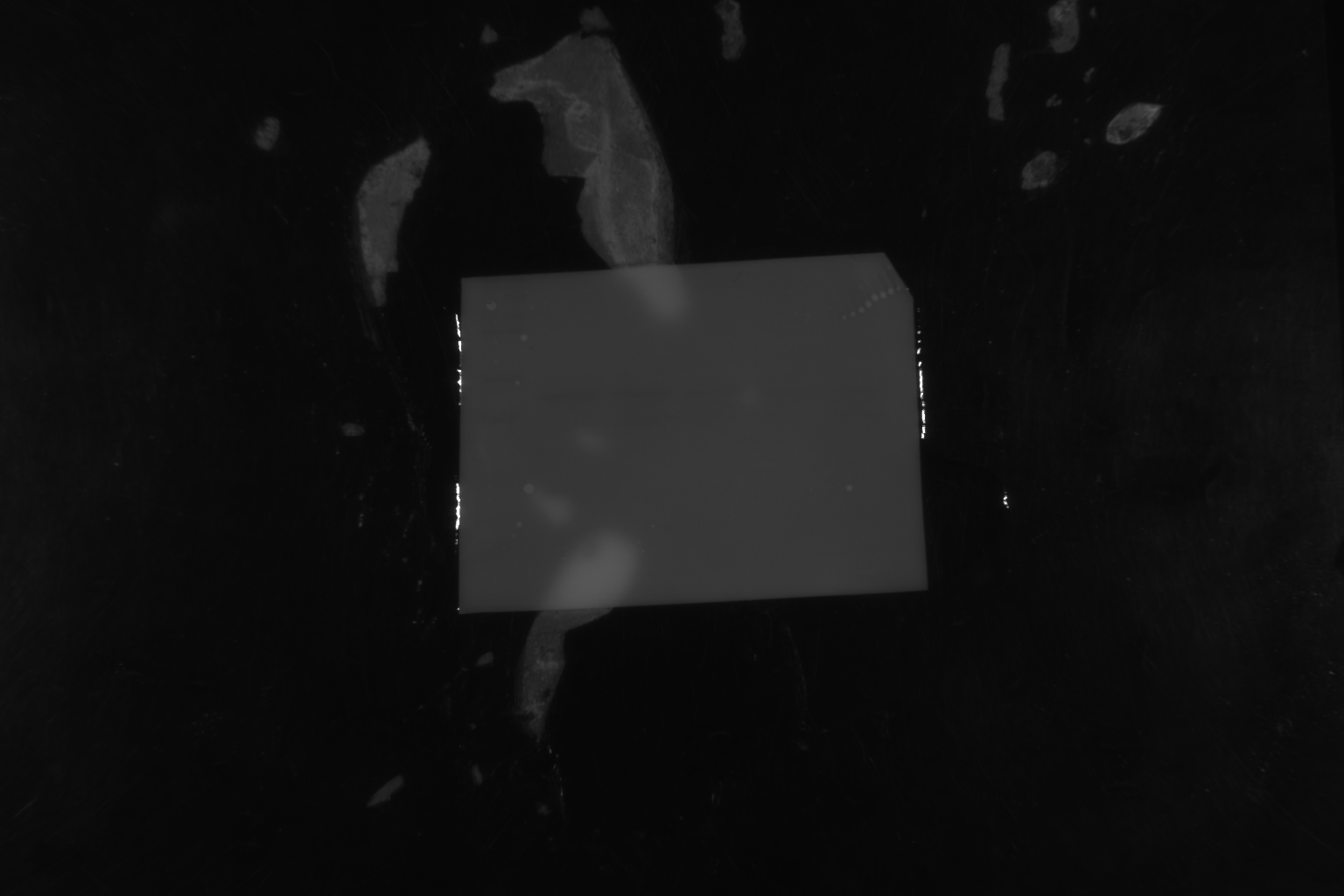

Supplement: Supplementary file 6 — Source data Fig. 4 [file 44318_2025_572_MOESM6_ESM.zip › Figure 4/Figure 4C/V_C B ACTIN 0.125 SEC.gel]

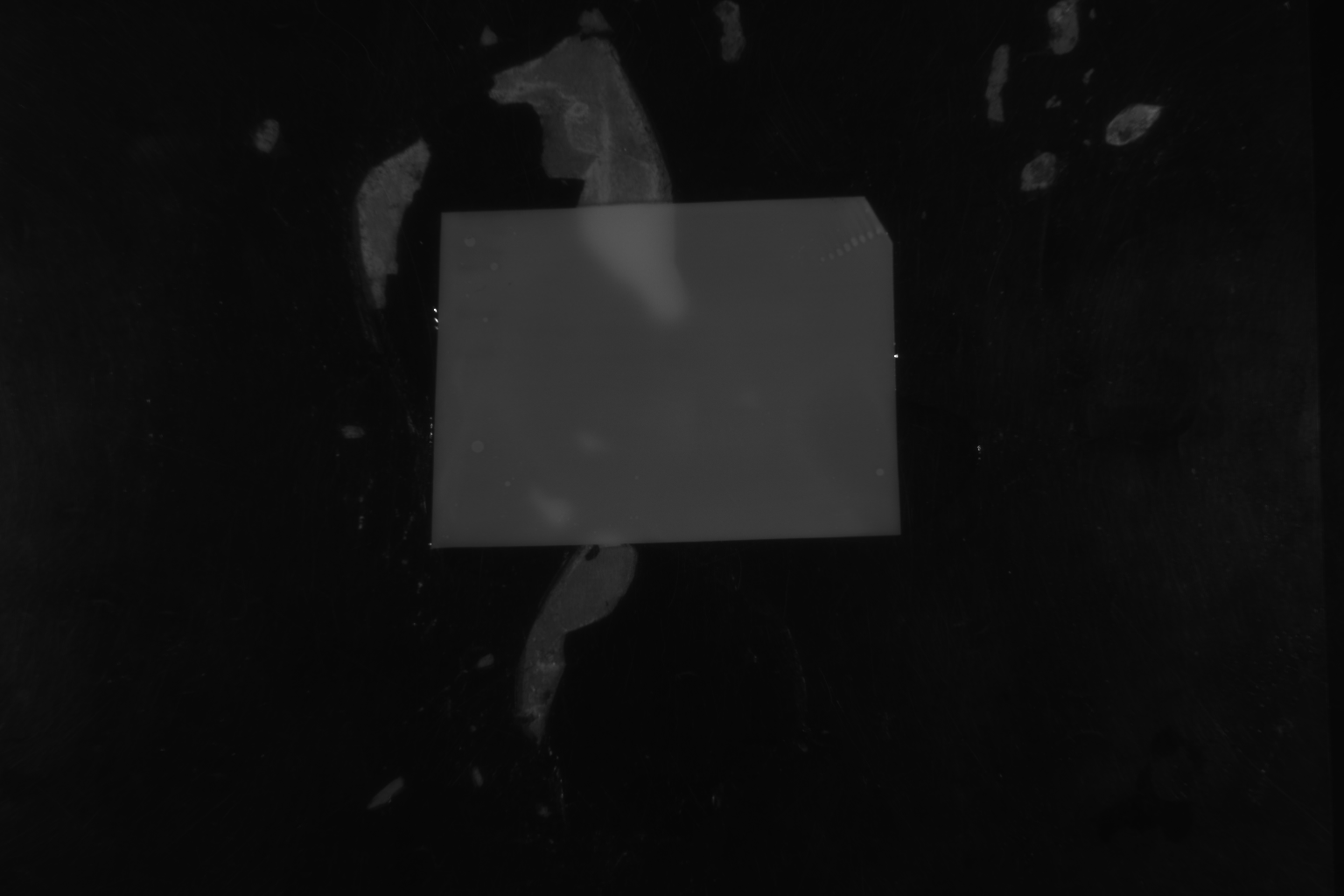

Supplement: Supplementary file 6 — Source data Fig. 4 [file 44318_2025_572_MOESM6_ESM.zip › Figure 4/Figure 4C/V_C B ACTIN 0.25 SEC.gel]

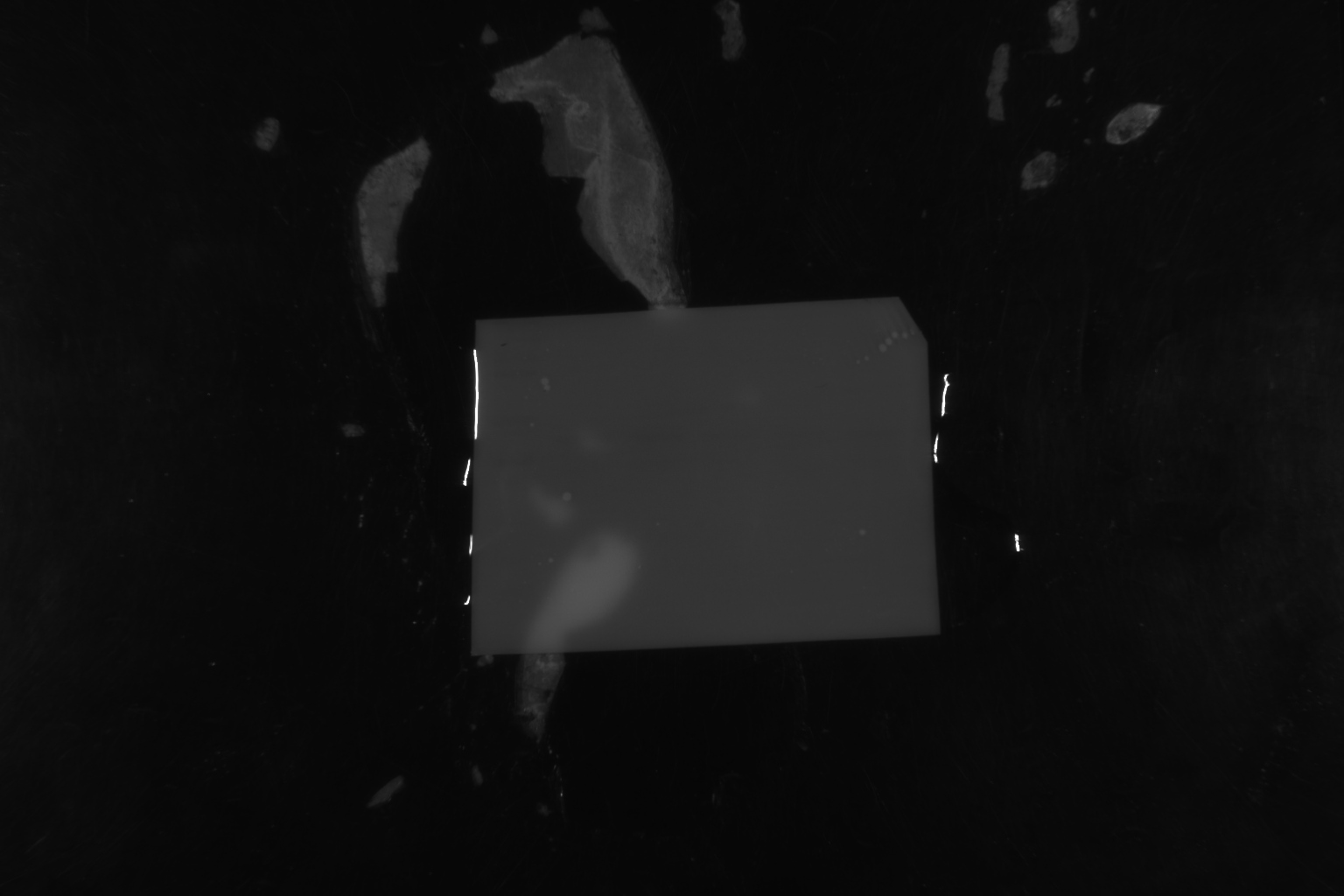

Supplement: Supplementary file 6 — Source data Fig. 4 [file 44318_2025_572_MOESM6_ESM.zip › Figure 4/Figure 4C/V_C B ACTIN 0.5 SEC.gel]

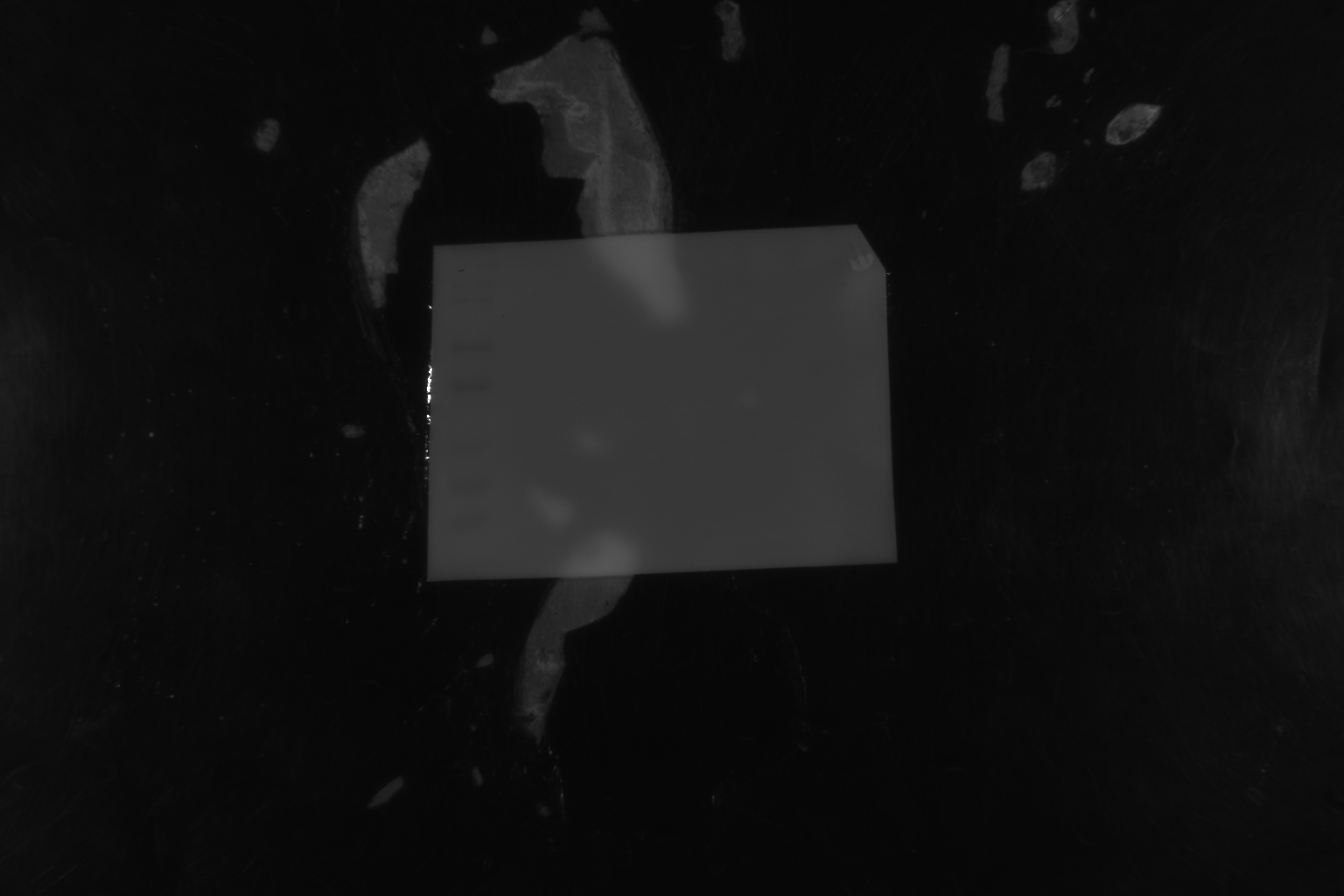

Supplement: Supplementary file 6 — Source data Fig. 4 [file 44318_2025_572_MOESM6_ESM.zip › Figure 4/Figure 4C/V_C ORAI 3 120 SEC.gel]

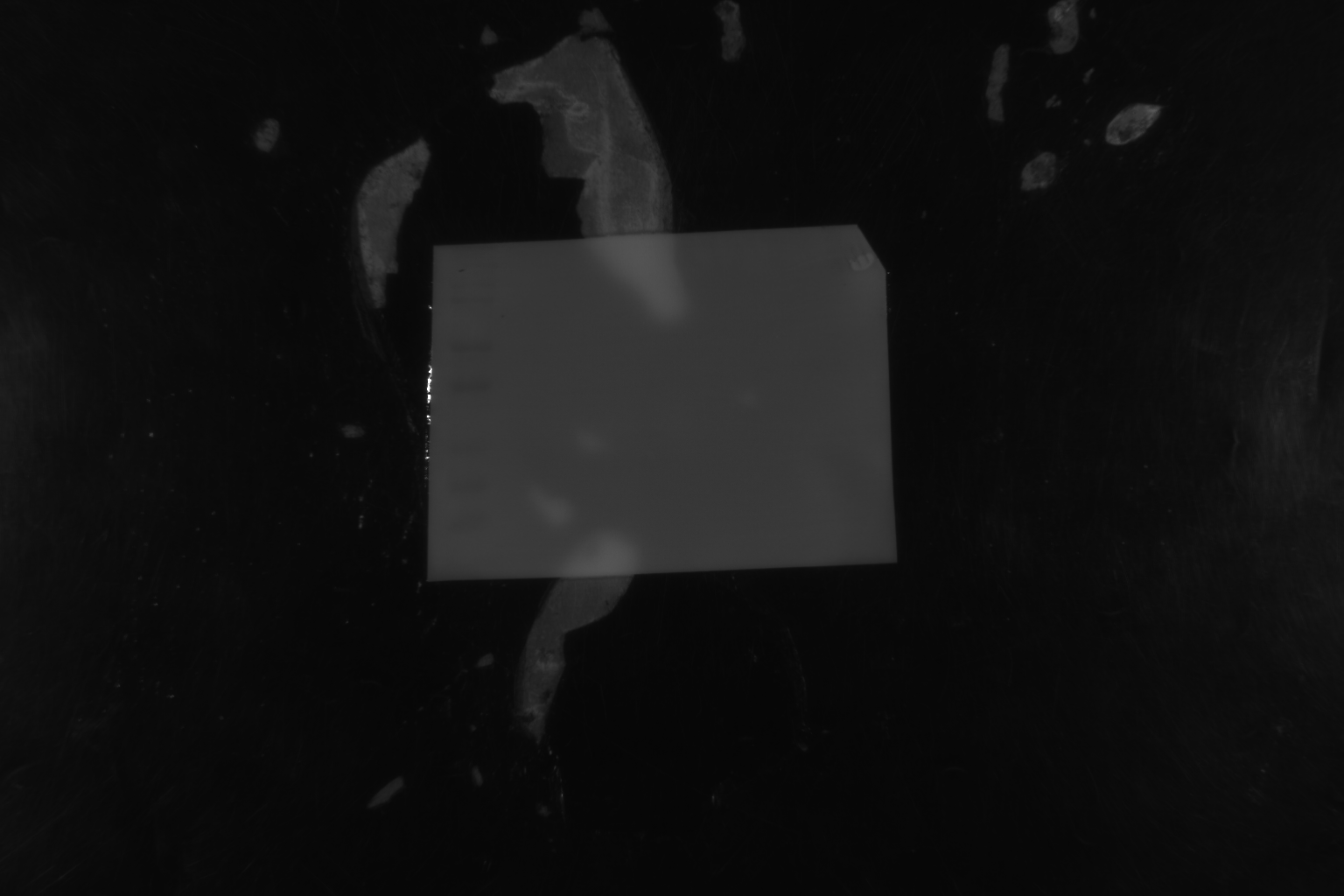

Supplement: Supplementary file 6 — Source data Fig. 4 [file 44318_2025_572_MOESM6_ESM.zip › Figure 4/Figure 4C/V_C ORAI 3 240 SEC.gel]

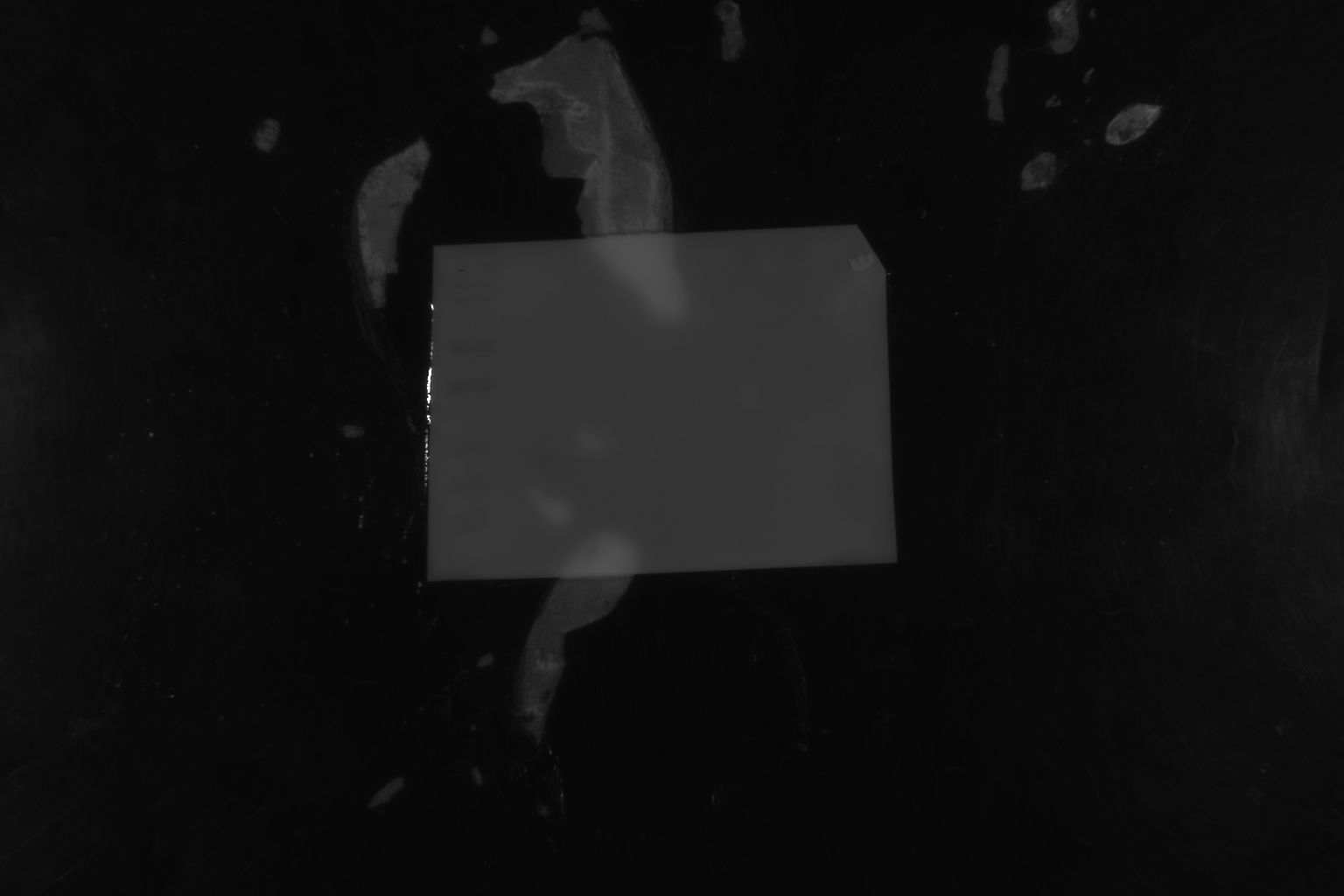

Supplement: Supplementary file 6 — Source data Fig. 4 [file 44318_2025_572_MOESM6_ESM.zip › Figure 4/Figure 4C/V_C ORAI 3 60 SEC.gel]

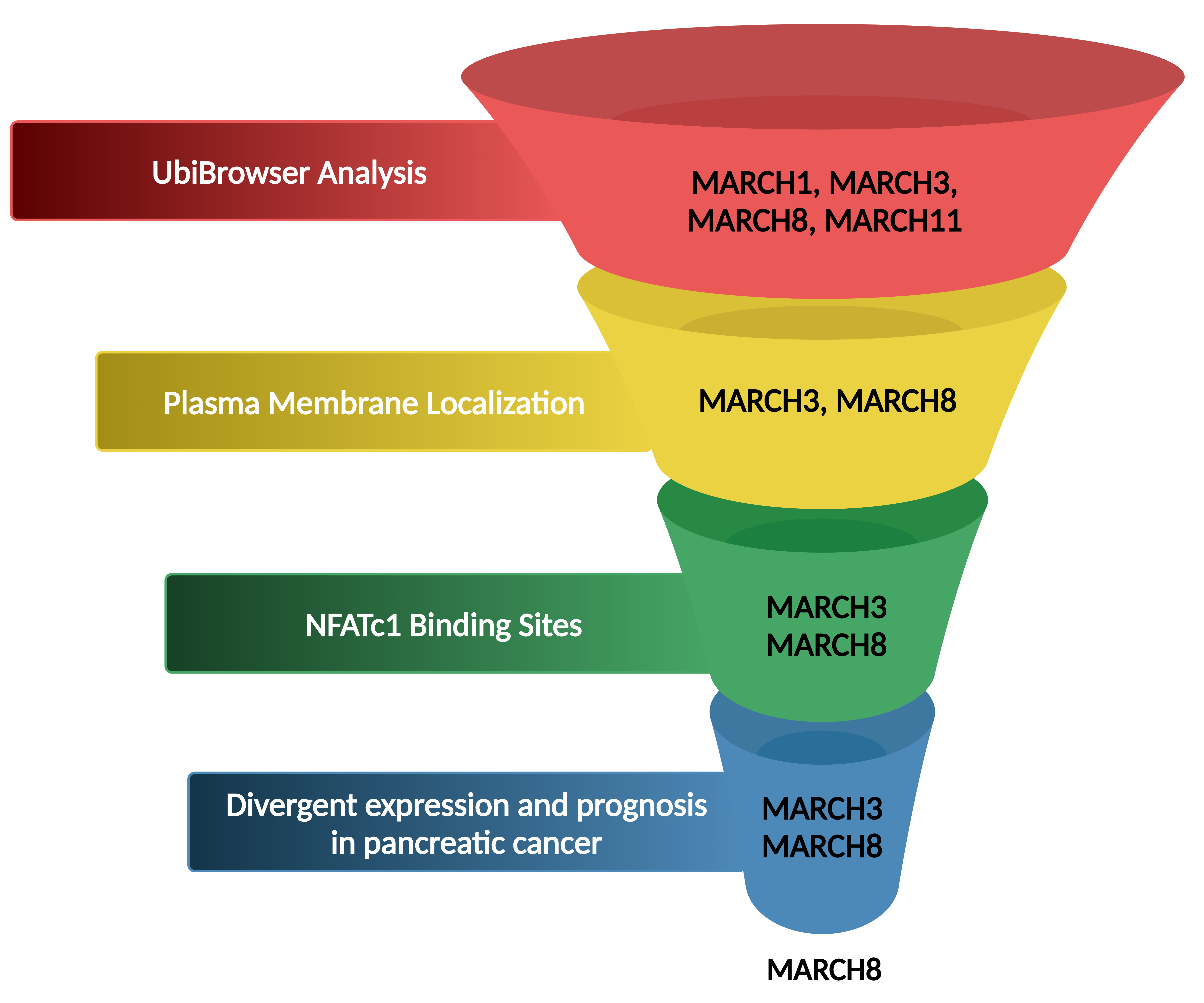

Supplement: Supplementary file 6 — Source data Fig. 4 [file 44318_2025_572_MOESM6_ESM.zip › Figure 4/Figure 4E/E3 Ubiquitin Ligase.png]

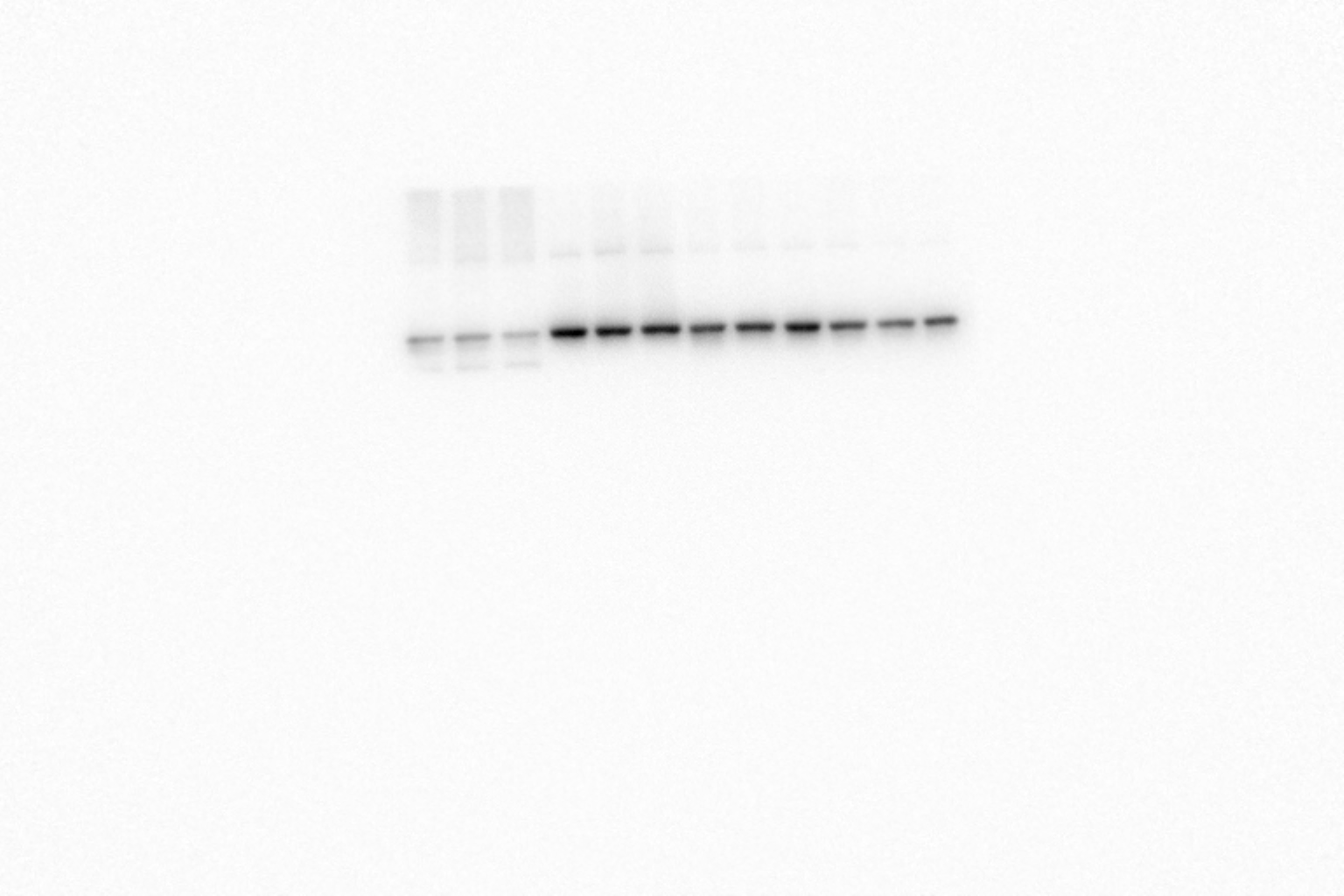

Supplement: Supplementary file 6 — Source data Fig. 4 [file 44318_2025_572_MOESM6_ESM.zip › Figure 4/Figure 4F/B ACTIN 0.5 SEC.gel]

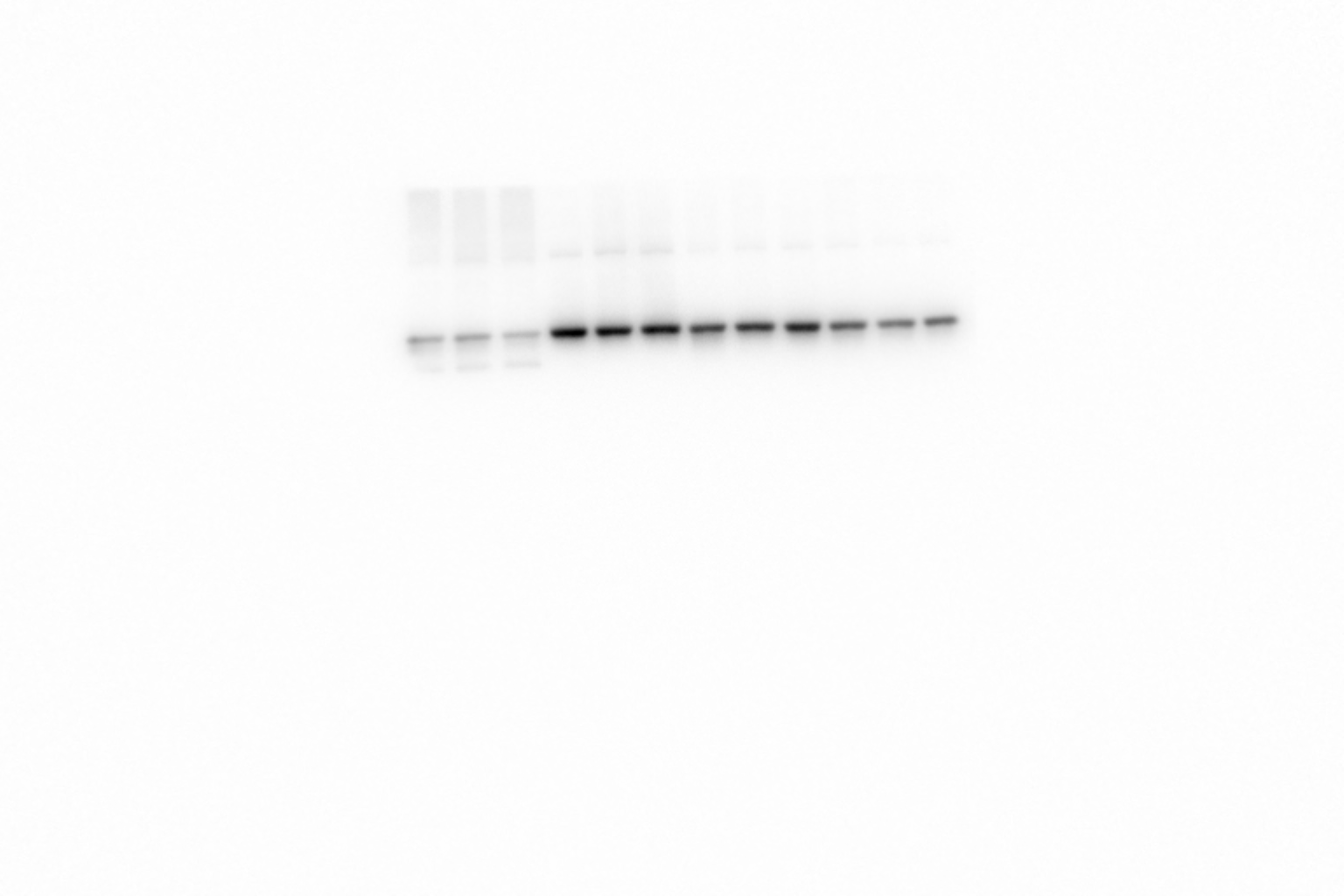

Supplement: Supplementary file 6 — Source data Fig. 4 [file 44318_2025_572_MOESM6_ESM.zip › Figure 4/Figure 4F/B ACTIN 1 SEC.gel]

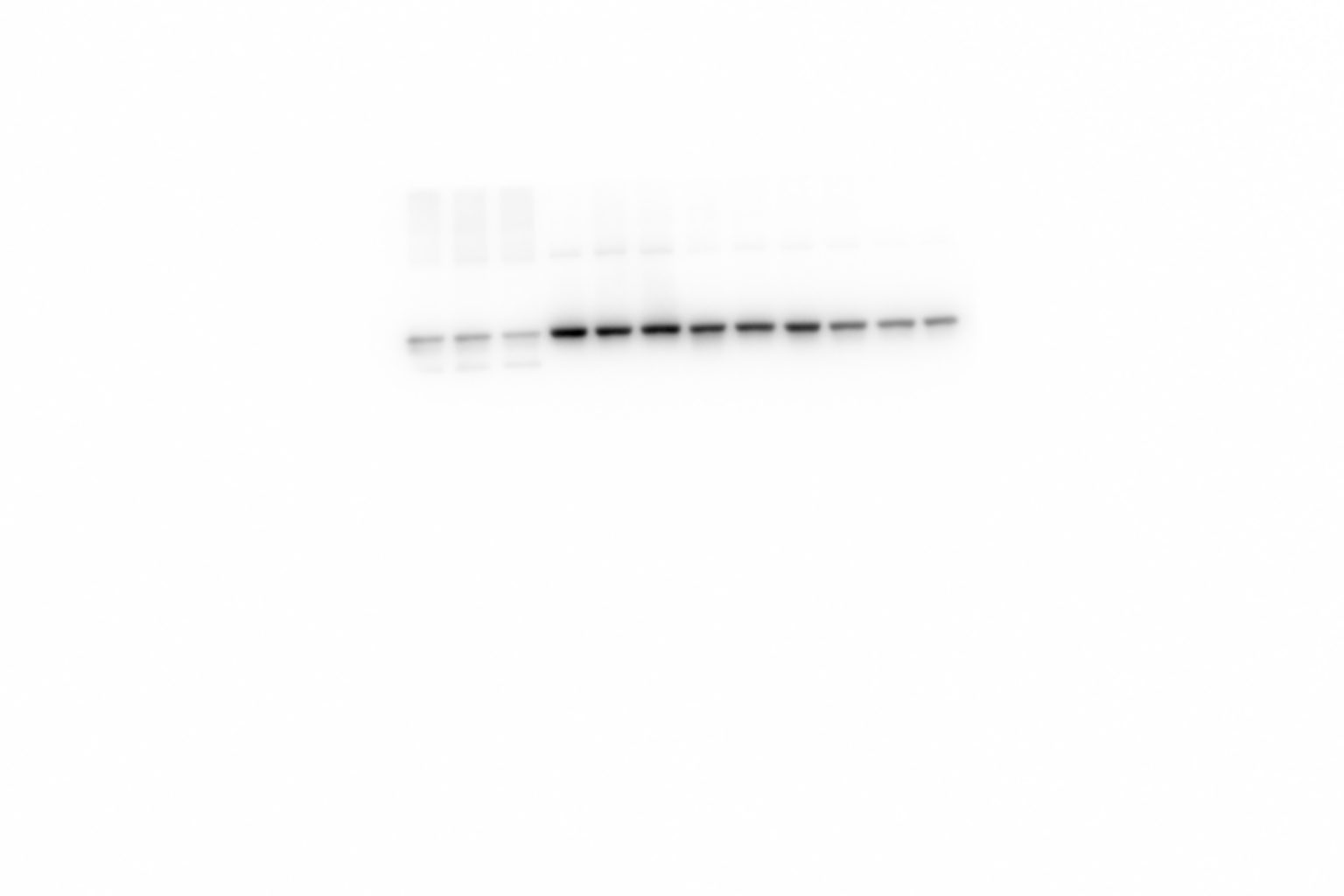

Supplement: Supplementary file 6 — Source data Fig. 4 [file 44318_2025_572_MOESM6_ESM.zip › Figure 4/Figure 4F/B ACTIN 2 SEC.gel]

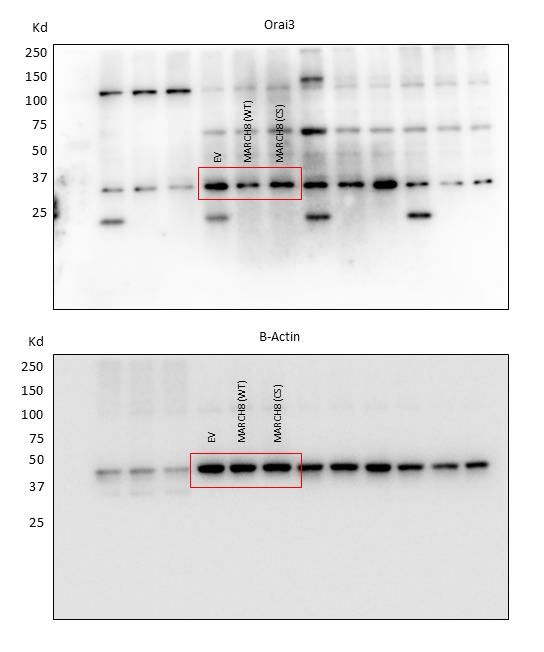

Supplement: Supplementary file 6 — Source data Fig. 4 [file 44318_2025_572_MOESM6_ESM.zip › Figure 4/Figure 4F/Figure 4F.png]

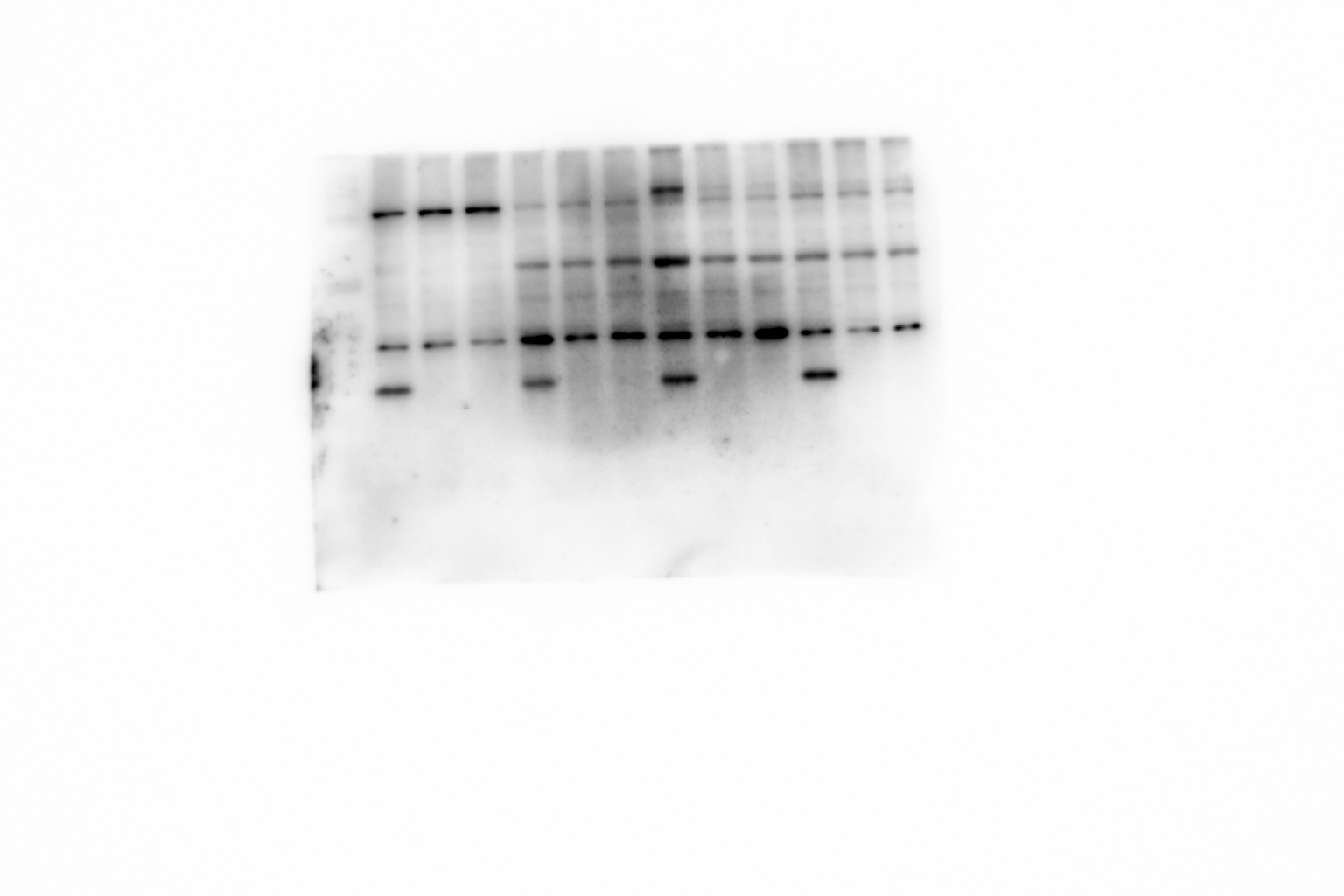

Supplement: Supplementary file 6 — Source data Fig. 4 [file 44318_2025_572_MOESM6_ESM.zip › Figure 4/Figure 4F/ORAI 3 120 SEC.gel]

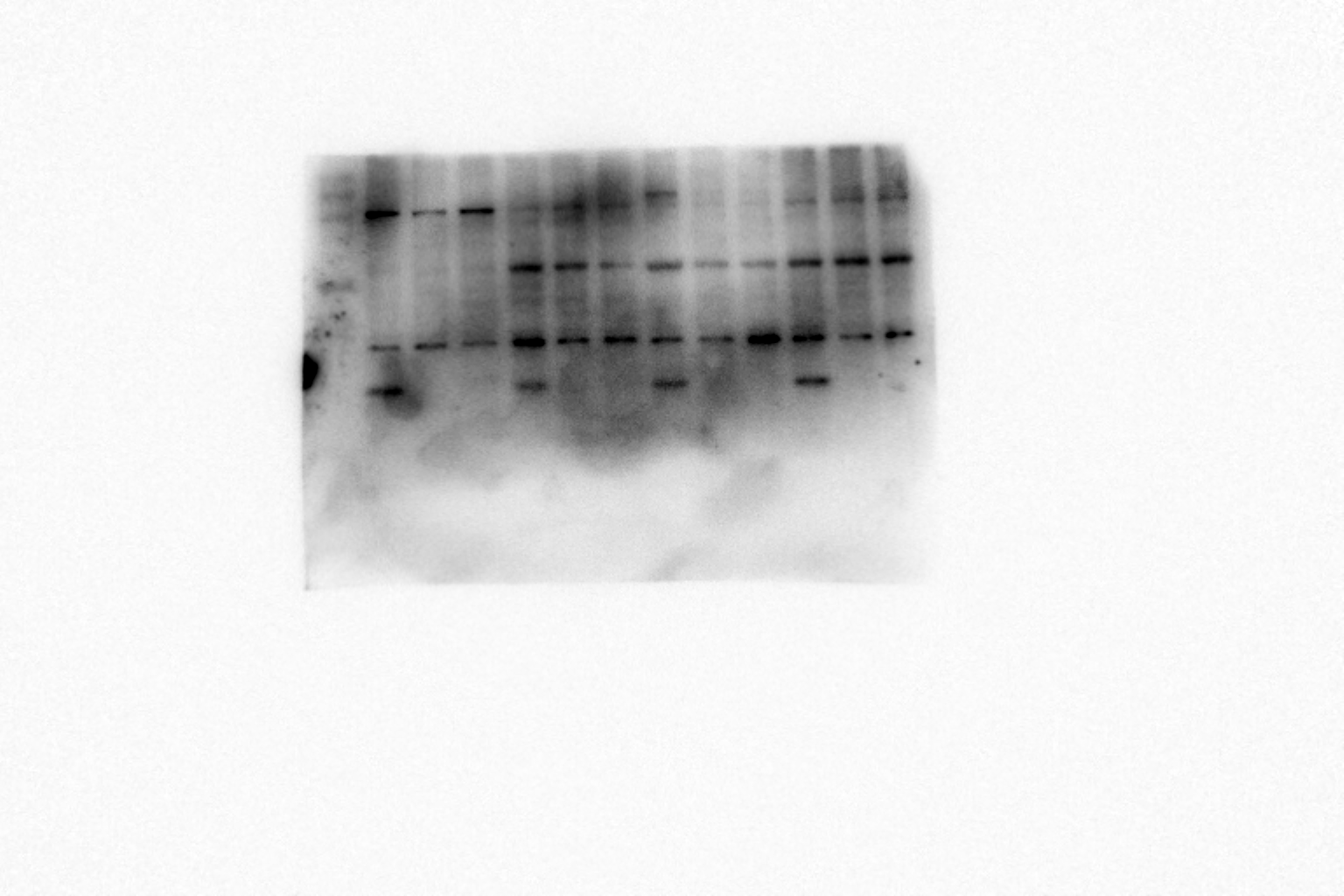

Supplement: Supplementary file 6 — Source data Fig. 4 [file 44318_2025_572_MOESM6_ESM.zip › Figure 4/Figure 4F/ORAI 3 15 SEC.gel]

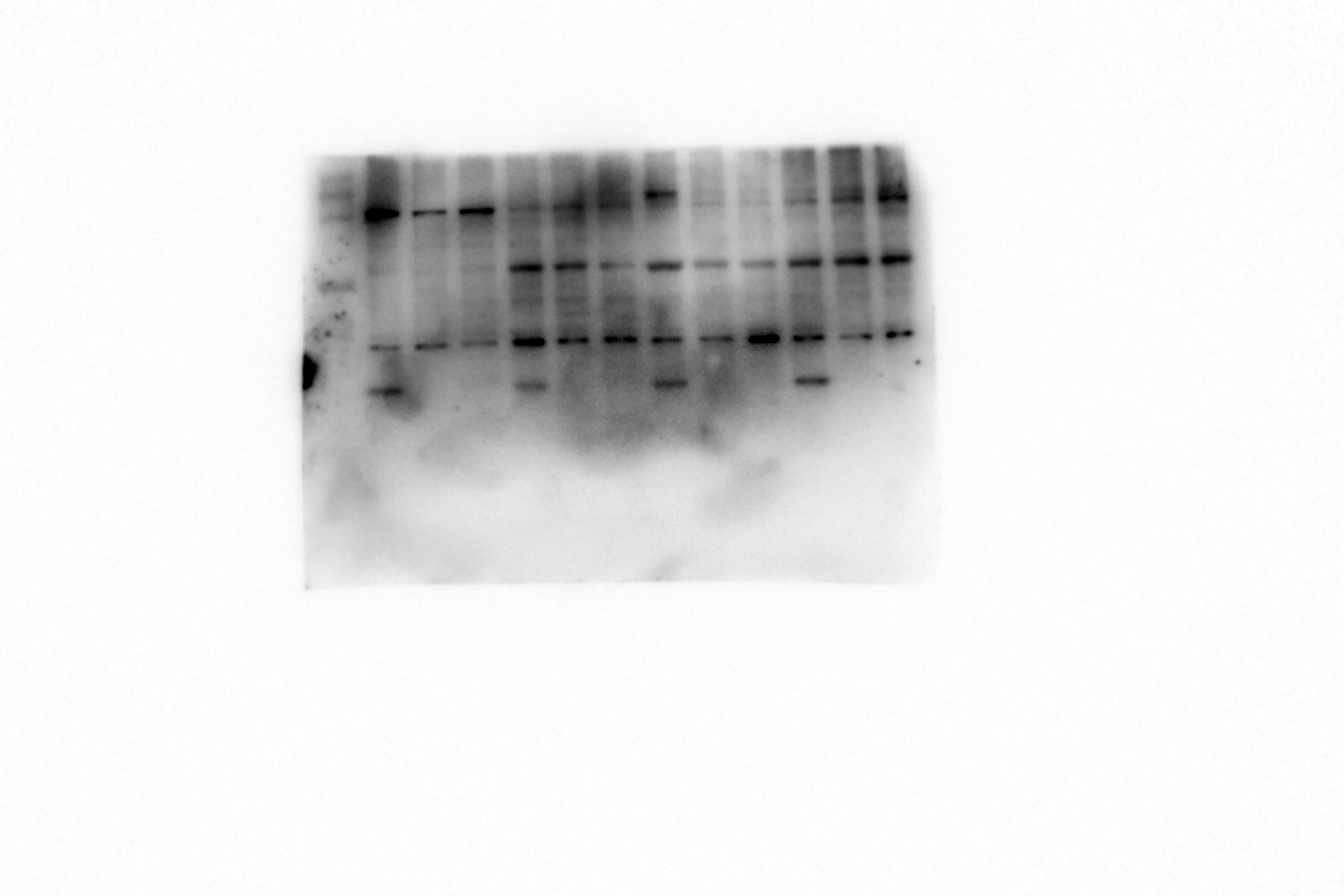

Supplement: Supplementary file 6 — Source data Fig. 4 [file 44318_2025_572_MOESM6_ESM.zip › Figure 4/Figure 4F/ORAI 3 30 SEC.gel]

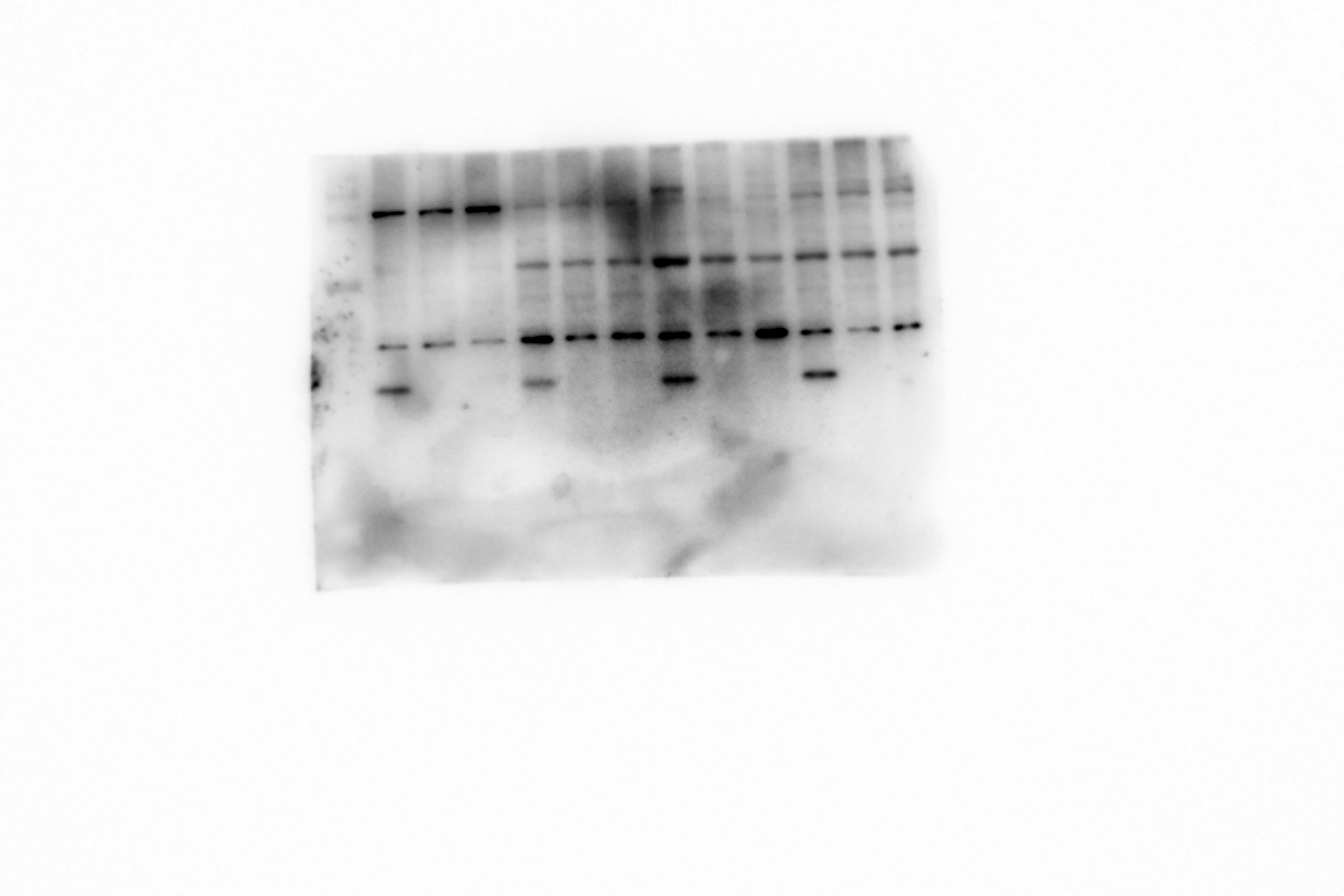

Supplement: Supplementary file 6 — Source data Fig. 4 [file 44318_2025_572_MOESM6_ESM.zip › Figure 4/Figure 4F/ORAI 3 60 SEC.gel]

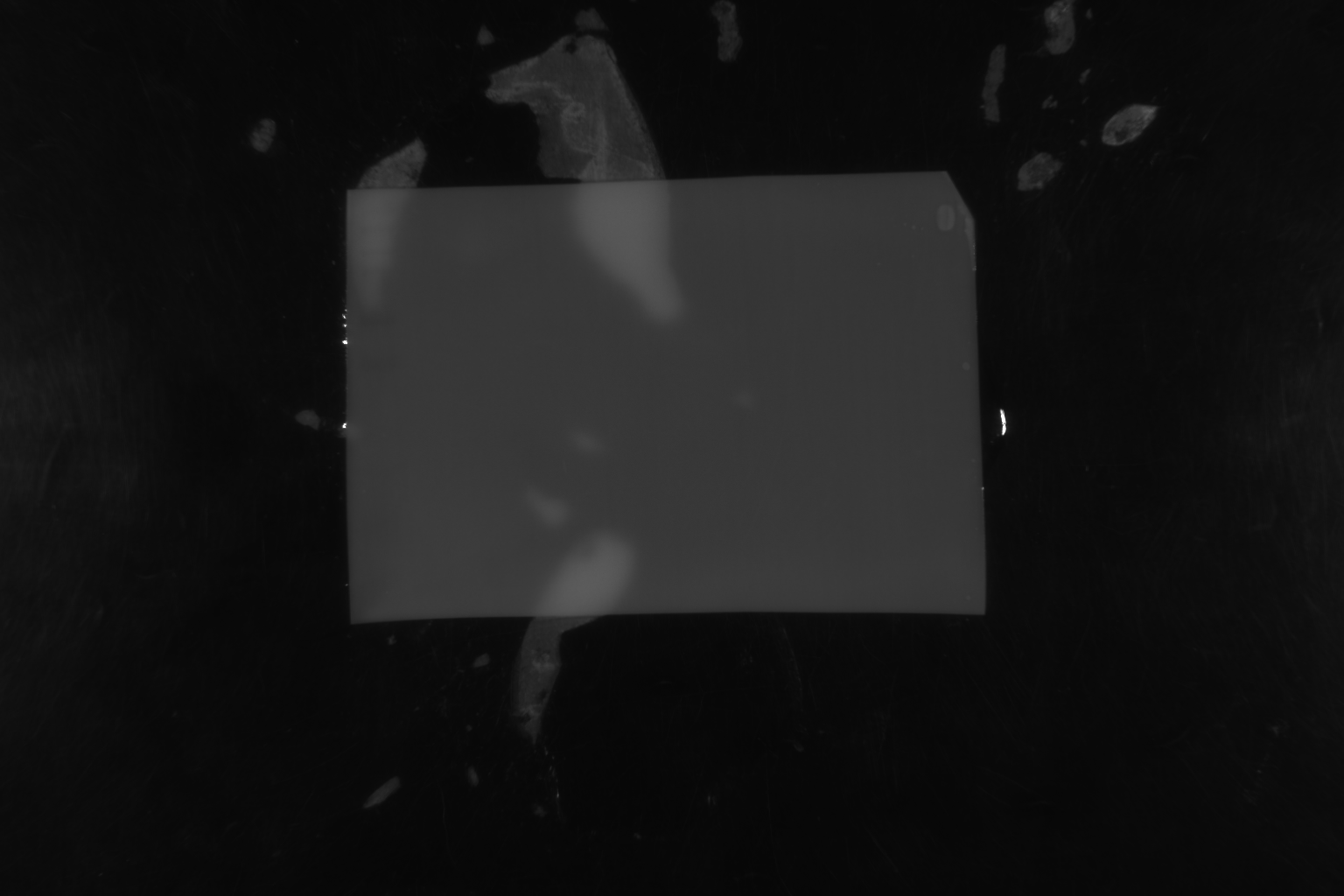

Supplement: Supplementary file 6 — Source data Fig. 4 [file 44318_2025_572_MOESM6_ESM.zip › Figure 4/Figure 4F/V_B ACTIN 0.5 SEC.gel]

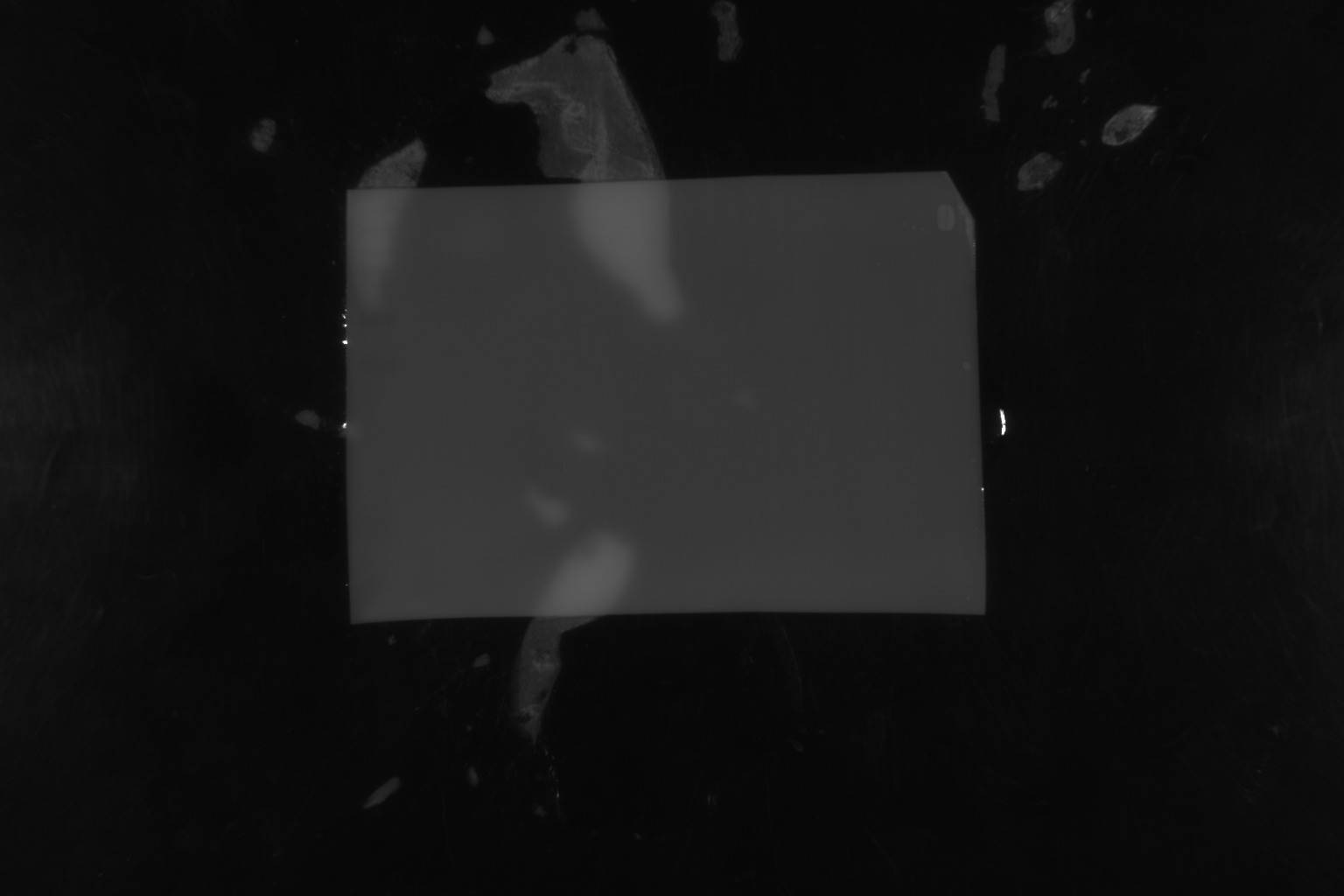

Supplement: Supplementary file 6 — Source data Fig. 4 [file 44318_2025_572_MOESM6_ESM.zip › Figure 4/Figure 4F/V_B ACTIN 1 SEC.gel]

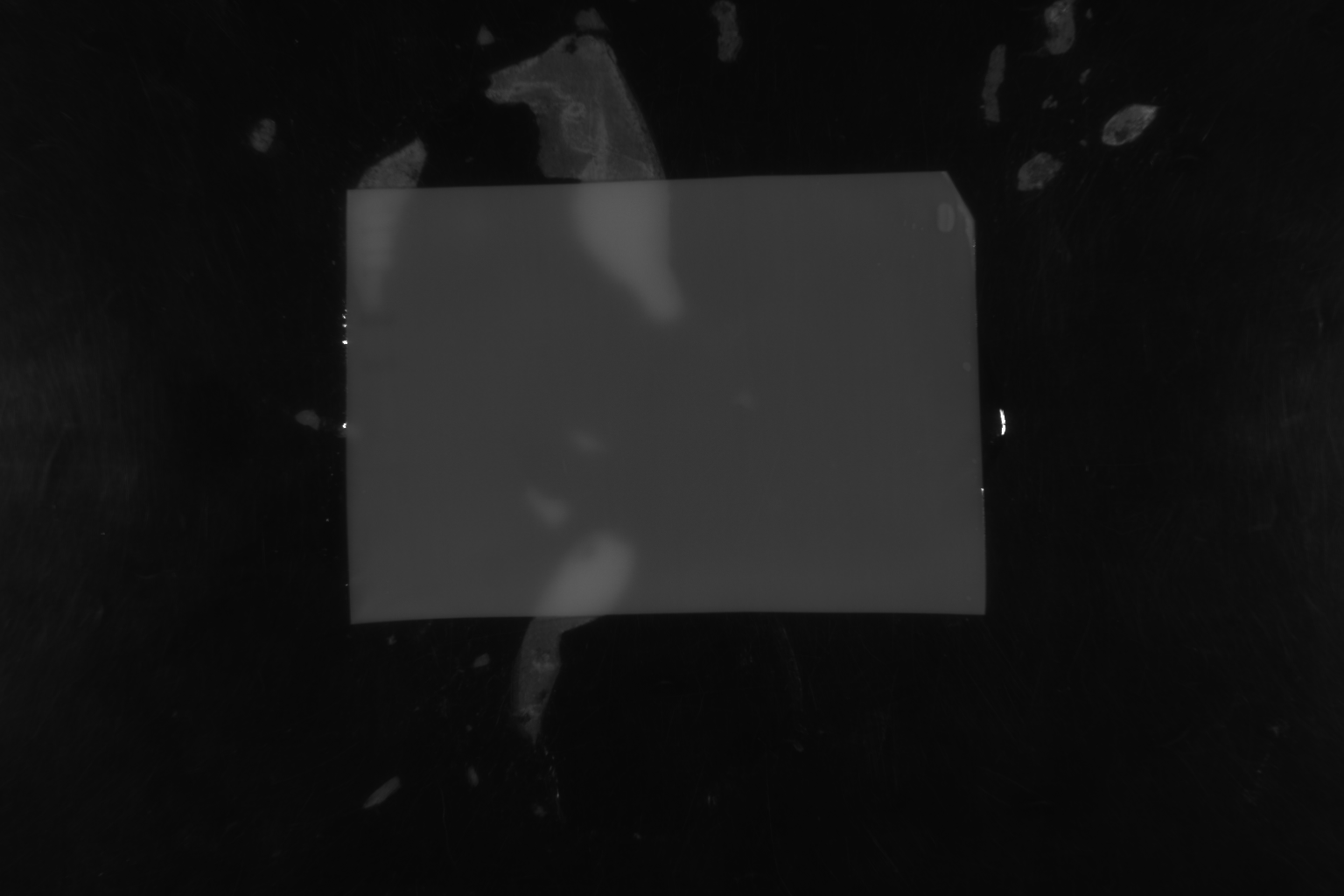

Supplement: Supplementary file 6 — Source data Fig. 4 [file 44318_2025_572_MOESM6_ESM.zip › Figure 4/Figure 4F/V_B ACTIN 2 SEC.gel]

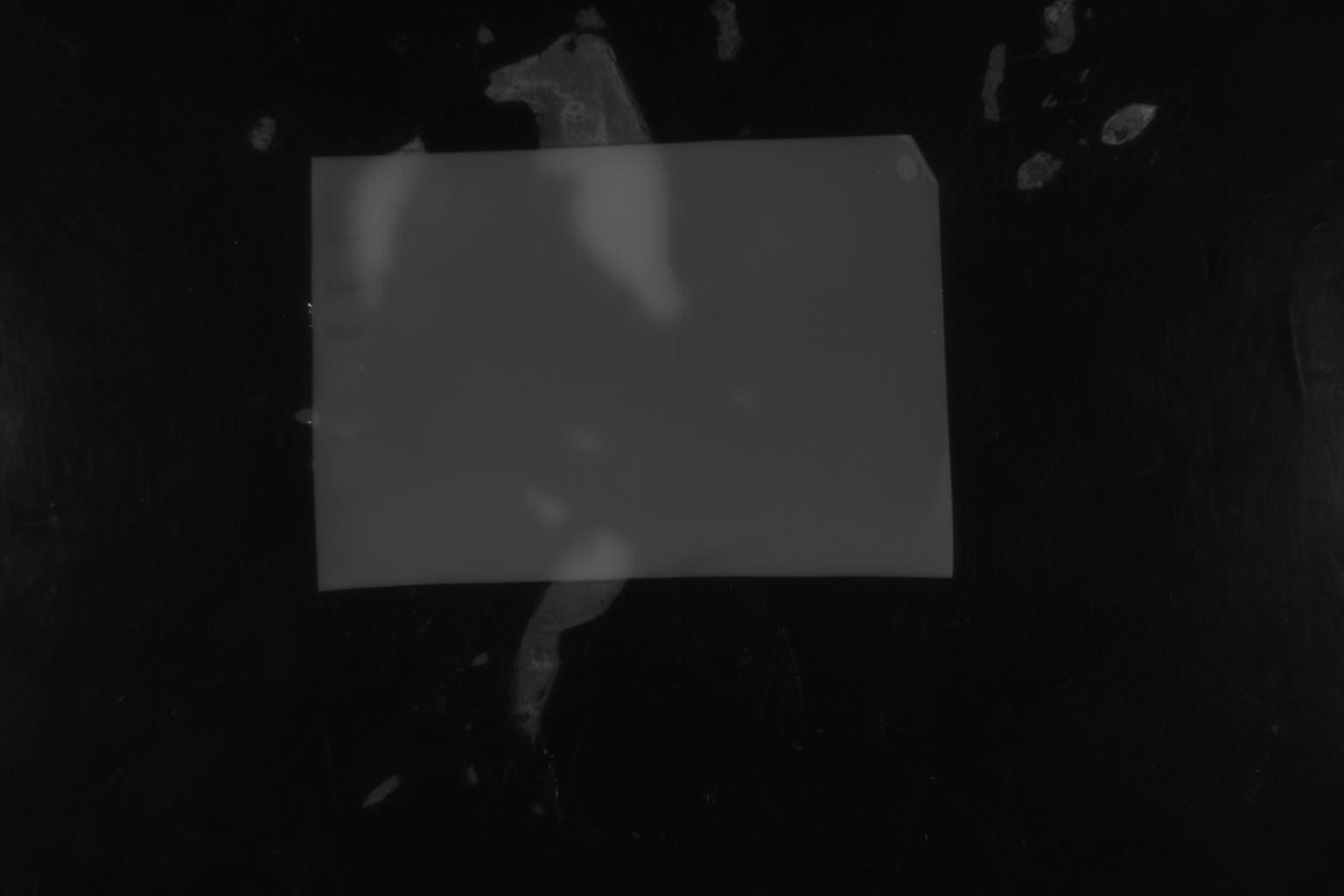

Supplement: Supplementary file 6 — Source data Fig. 4 [file 44318_2025_572_MOESM6_ESM.zip › Figure 4/Figure 4F/V_ORAI 3 120 SEC.gel]

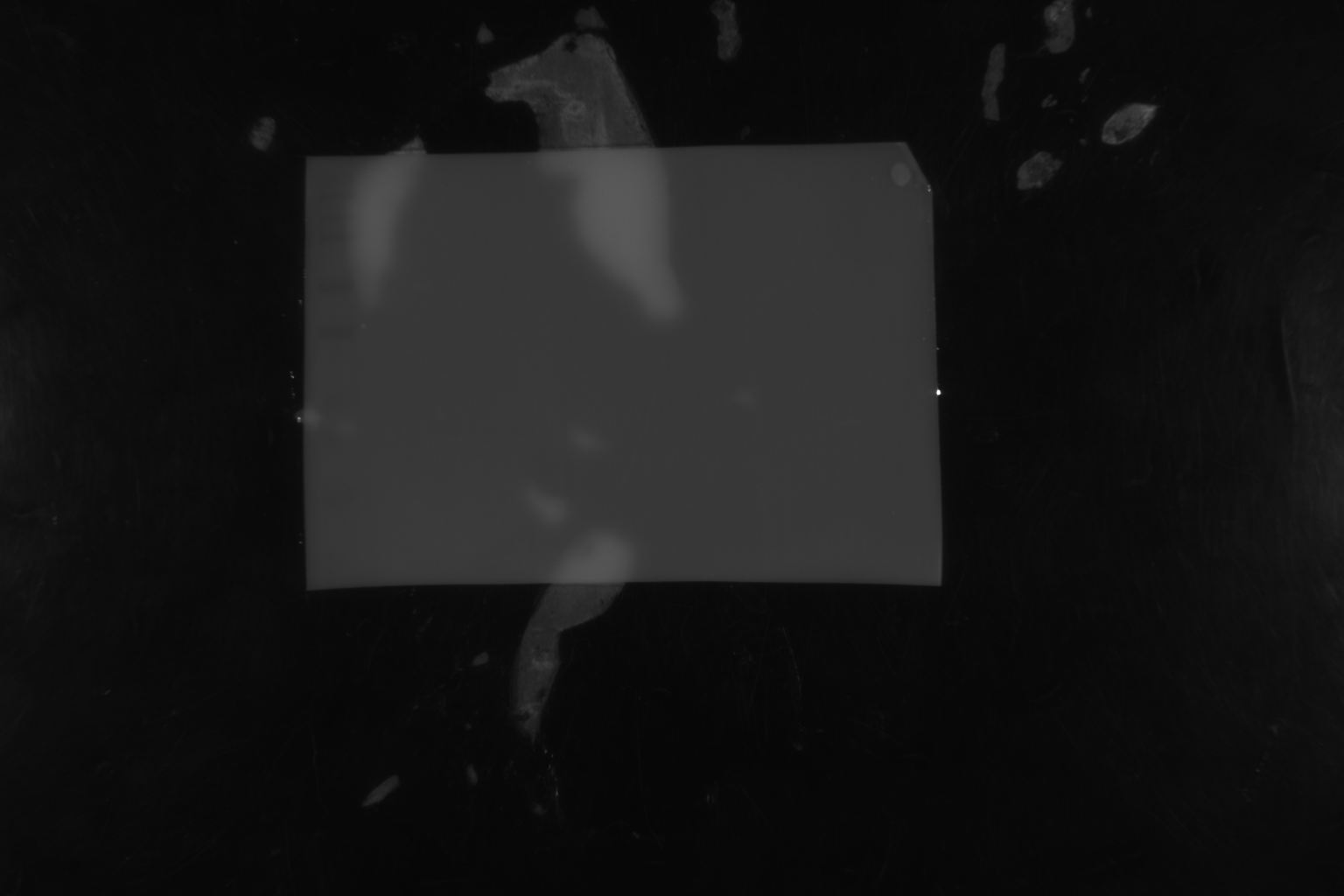

Supplement: Supplementary file 6 — Source data Fig. 4 [file 44318_2025_572_MOESM6_ESM.zip › Figure 4/Figure 4F/V_ORAI 3 15 SEC.gel]

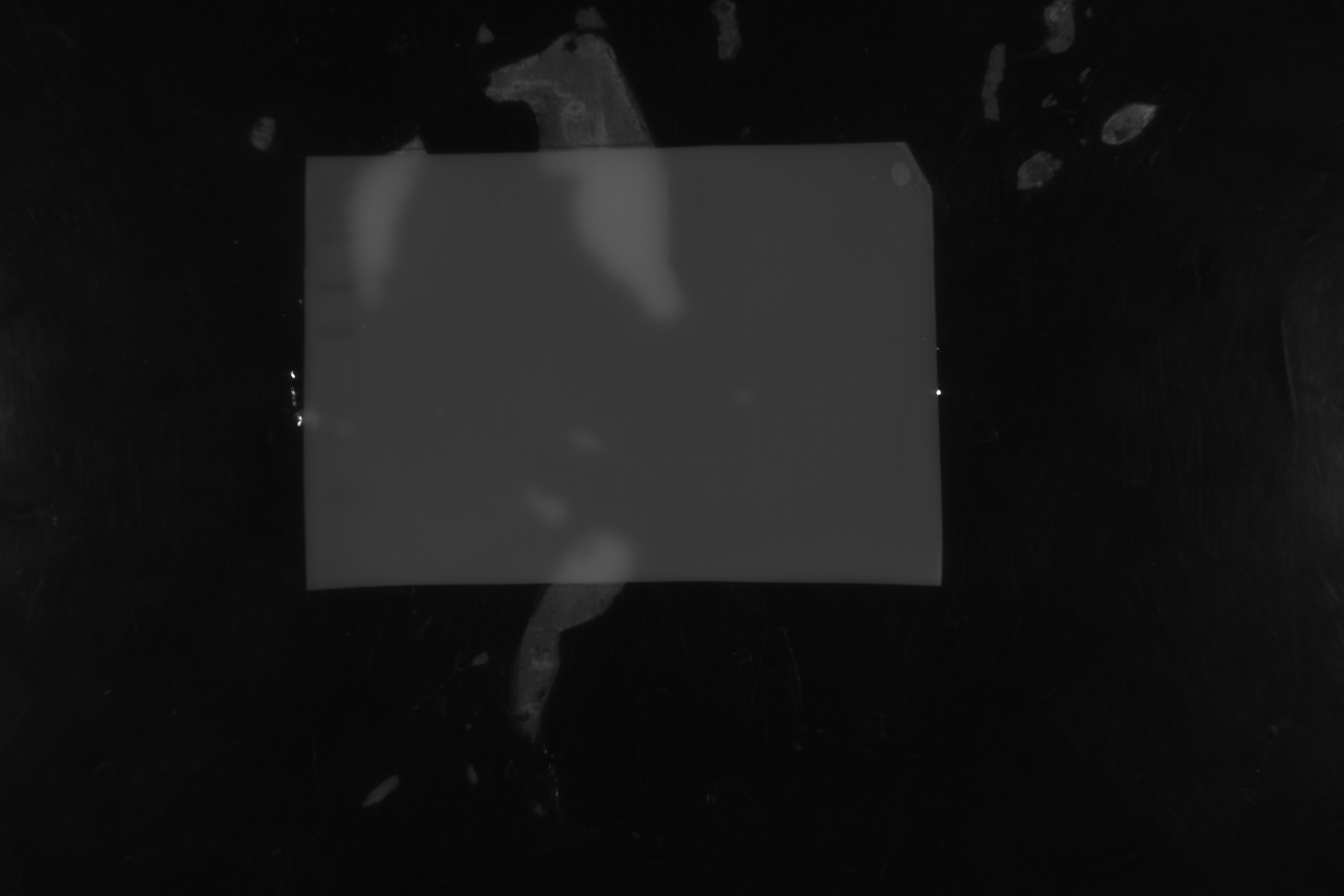

Supplement: Supplementary file 6 — Source data Fig. 4 [file 44318_2025_572_MOESM6_ESM.zip › Figure 4/Figure 4F/V_ORAI 3 30 SEC.gel]

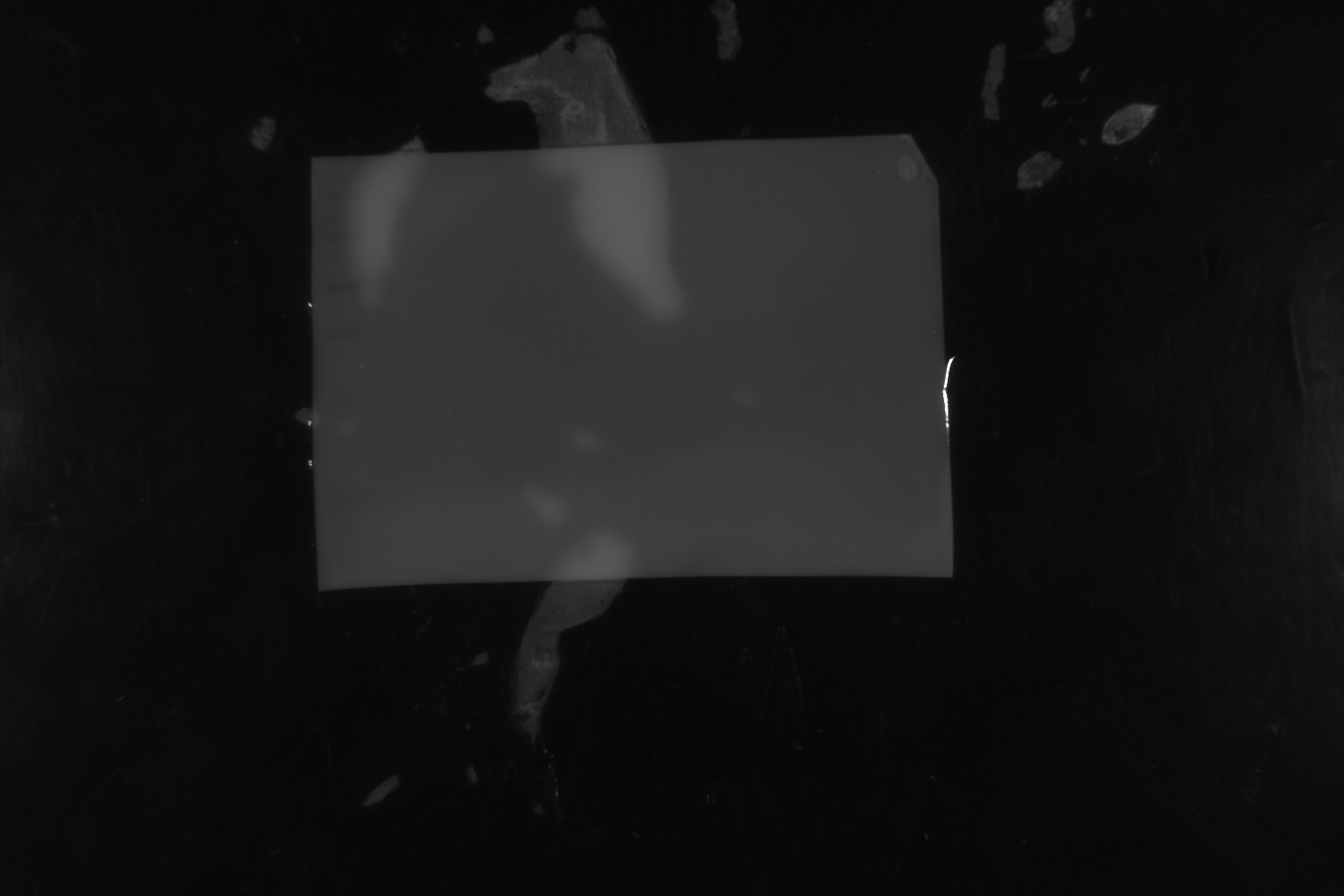

Supplement: Supplementary file 6 — Source data Fig. 4 [file 44318_2025_572_MOESM6_ESM.zip › Figure 4/Figure 4F/V_ORAI 3 60 SEC.gel]

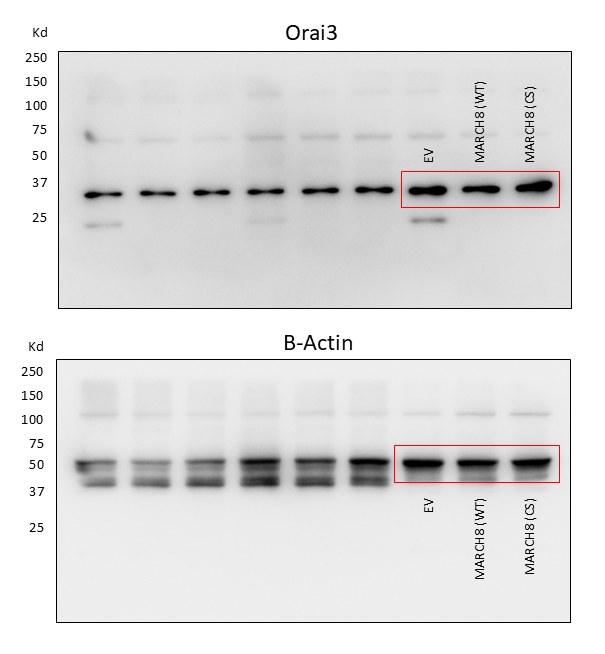

Supplement: Supplementary file 6 — Source data Fig. 4 [file 44318_2025_572_MOESM6_ESM.zip › Figure 4/Figure 4J/Figure 4J.png]

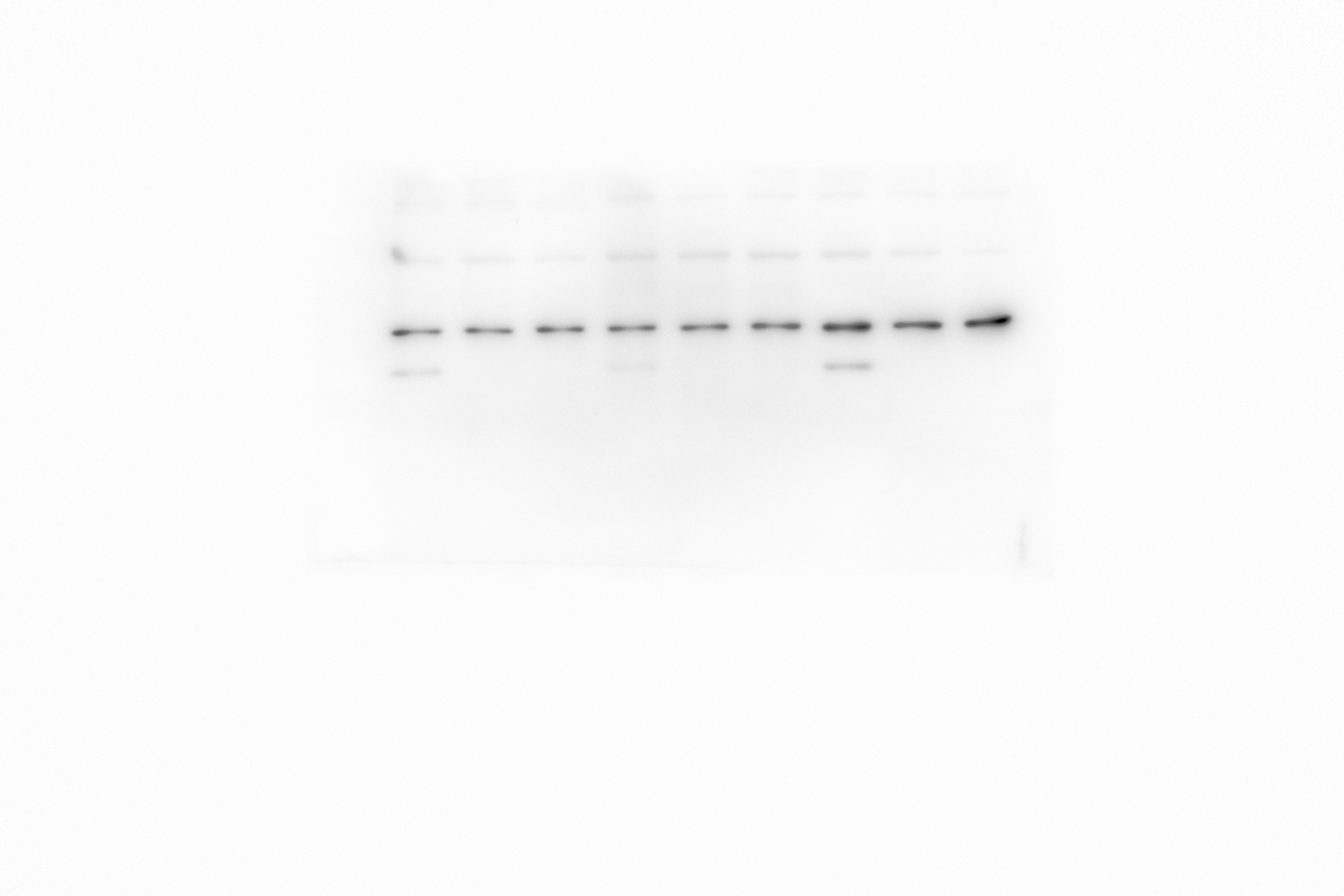

Supplement: Supplementary file 6 — Source data Fig. 4 [file 44318_2025_572_MOESM6_ESM.zip › Figure 4/Figure 4J/ORAI 3 8 SEC.gel]

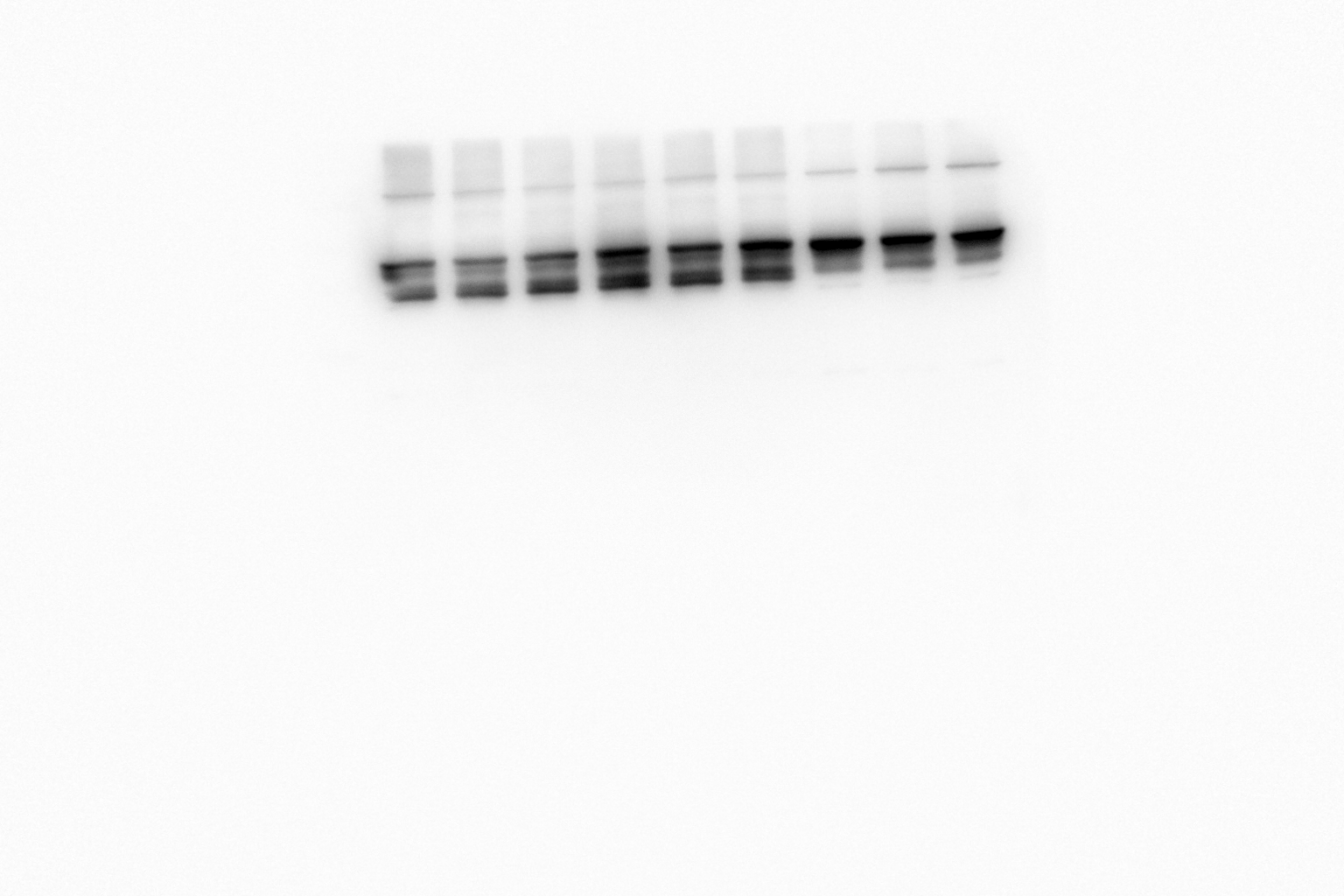

Supplement: Supplementary file 6 — Source data Fig. 4 [file 44318_2025_572_MOESM6_ESM.zip › Figure 4/Figure 4J/P B ACTIN 0.5 SEC.gel]

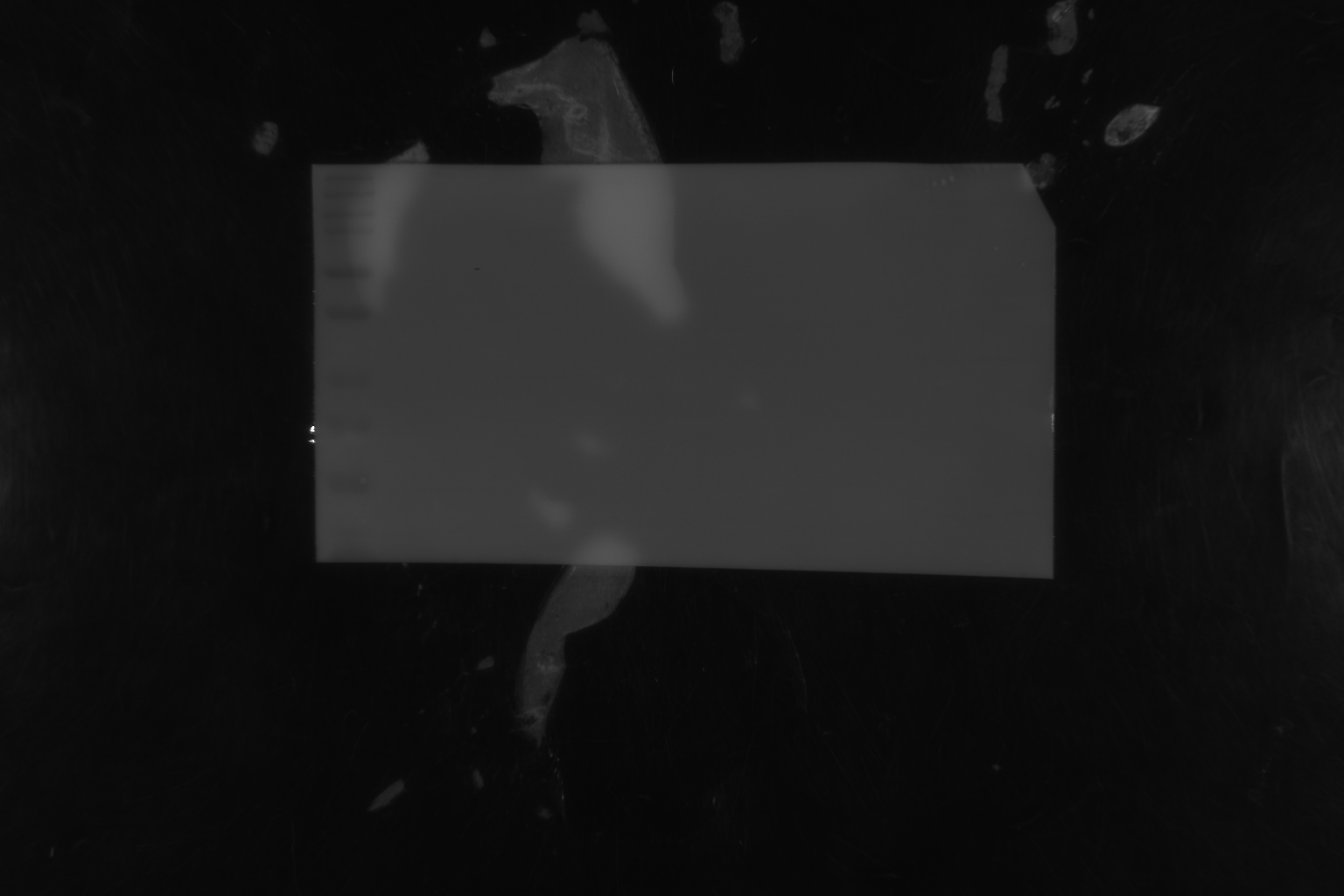

Supplement: Supplementary file 6 — Source data Fig. 4 [file 44318_2025_572_MOESM6_ESM.zip › Figure 4/Figure 4J/V_ORAI 3 8 SEC.gel]

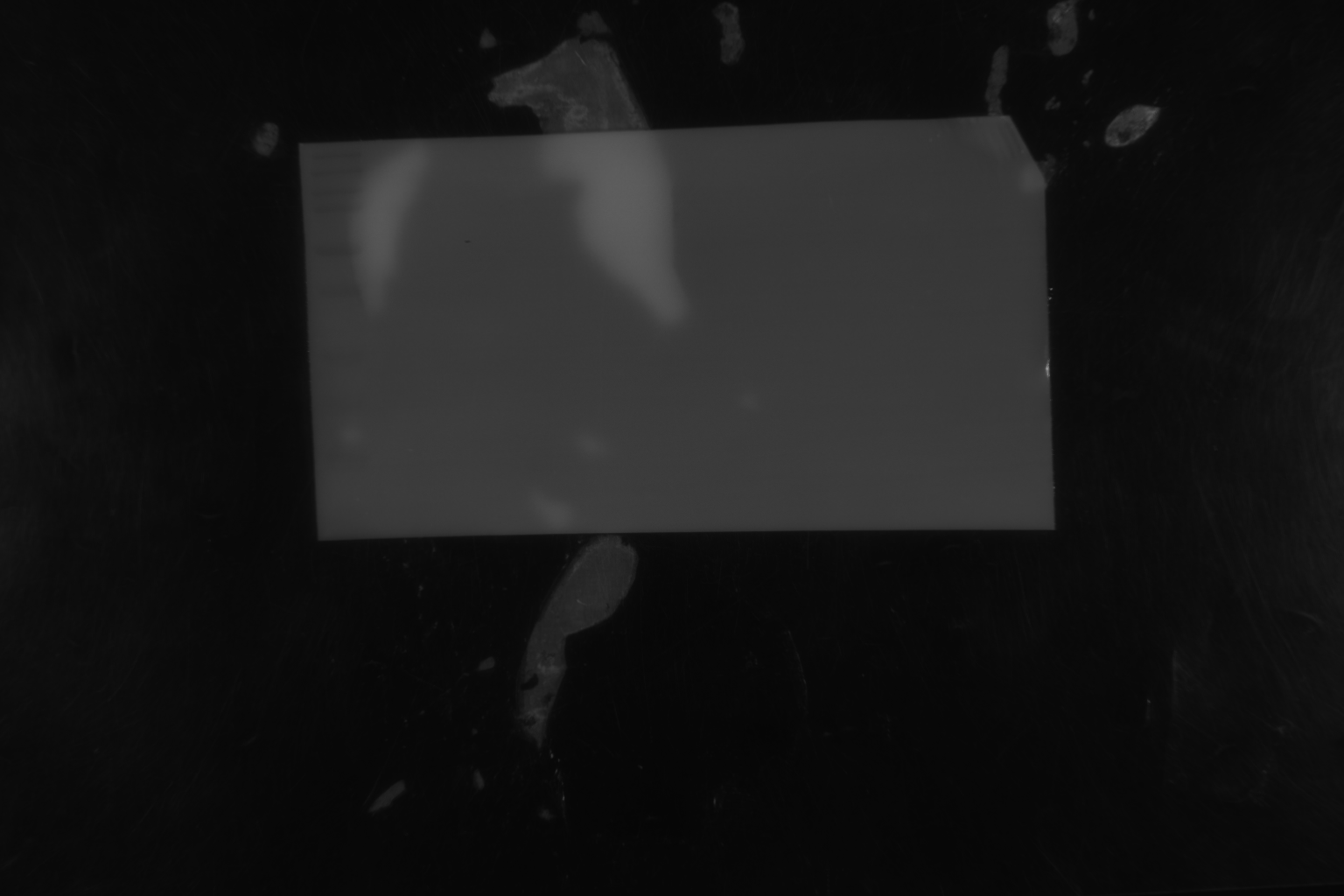

Supplement: Supplementary file 6 — Source data Fig. 4 [file 44318_2025_572_MOESM6_ESM.zip › Figure 4/Figure 4J/V_P B ACTIN 0.5 SEC.gel]

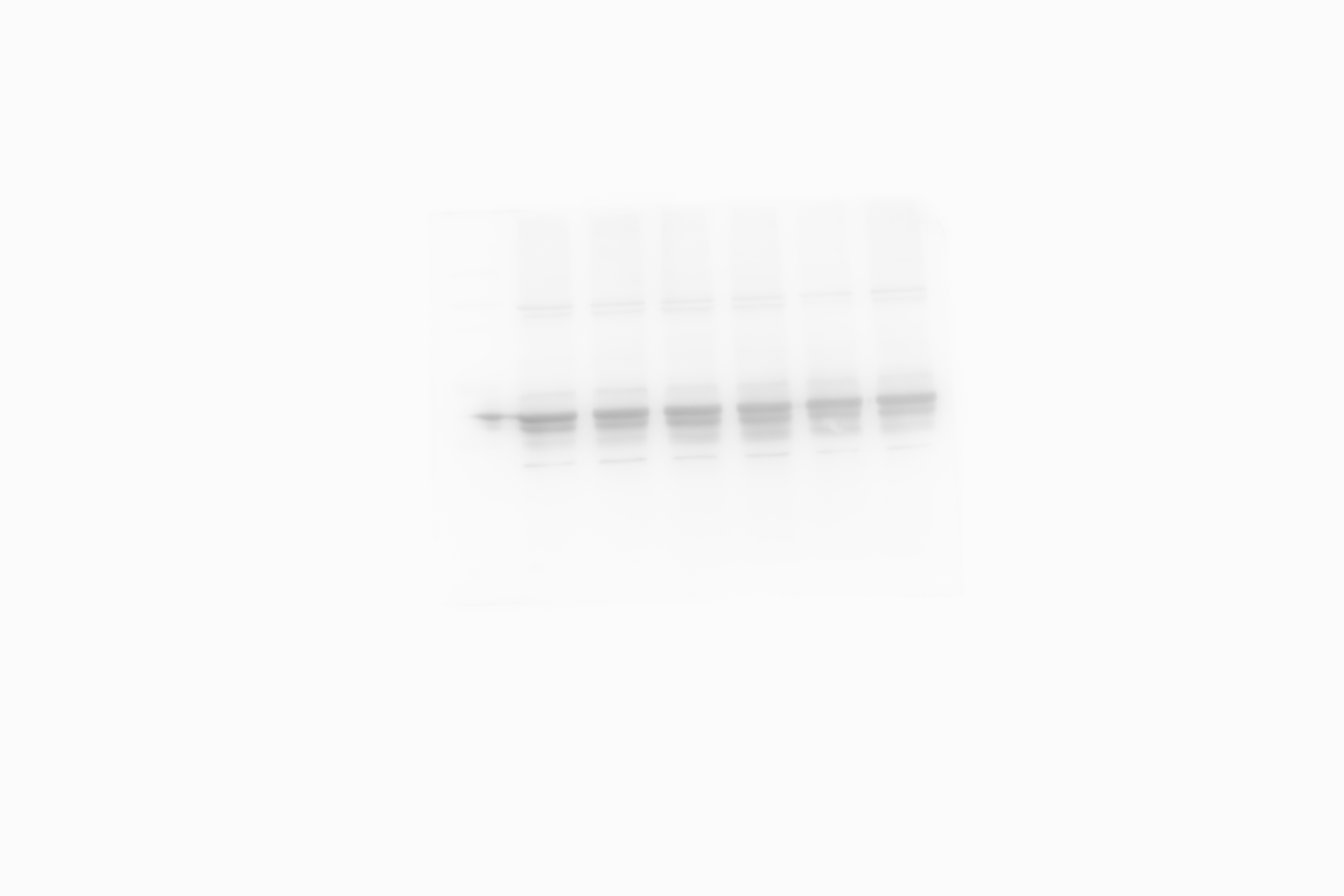

Supplement: Supplementary file 6 — Source data Fig. 4 [file 44318_2025_572_MOESM6_ESM.zip › Figure 4/Figure 4N/B ACTIN 2 SEC.gel]

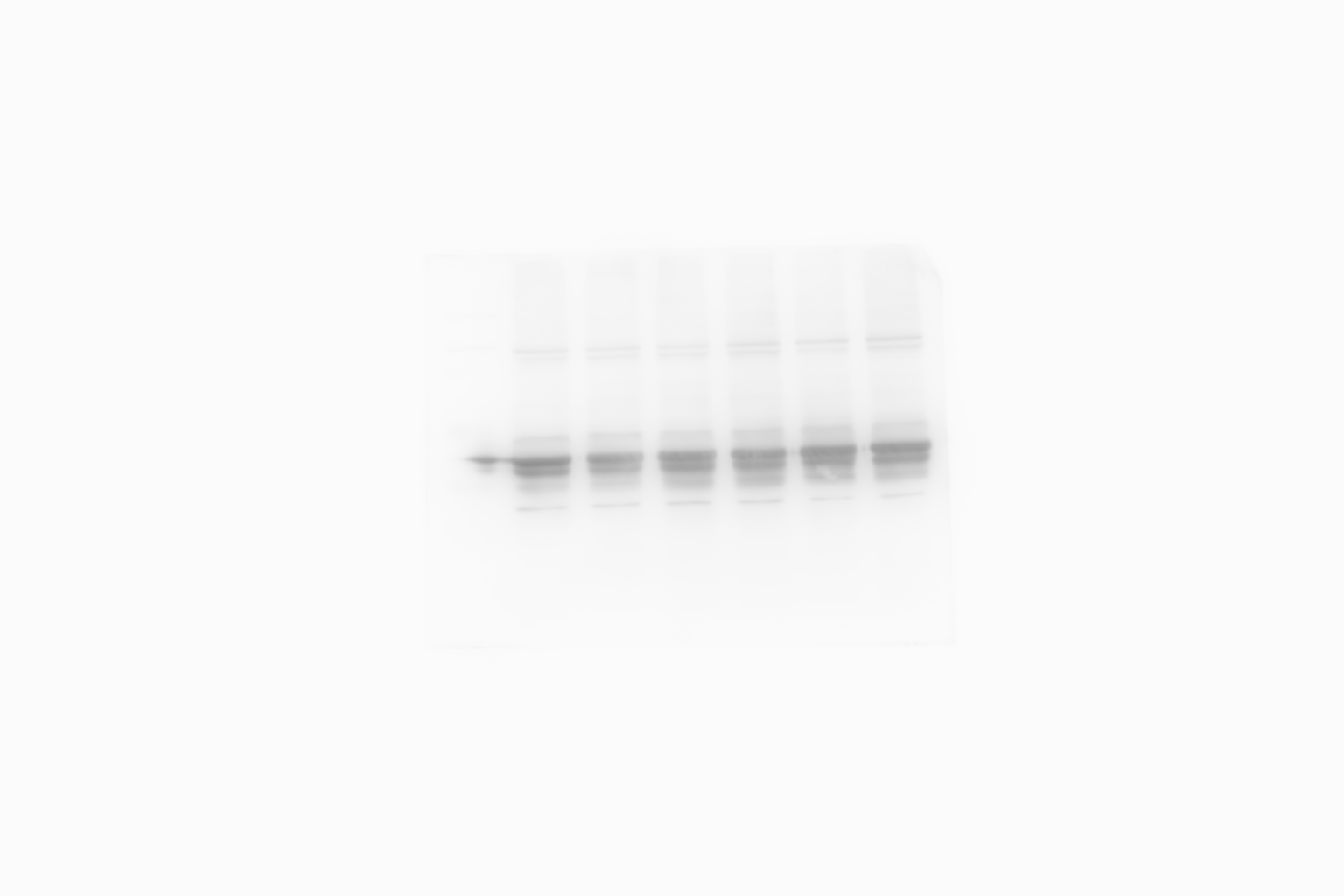

Supplement: Supplementary file 6 — Source data Fig. 4 [file 44318_2025_572_MOESM6_ESM.zip › Figure 4/Figure 4N/B ACTIN 3 SEC.gel]

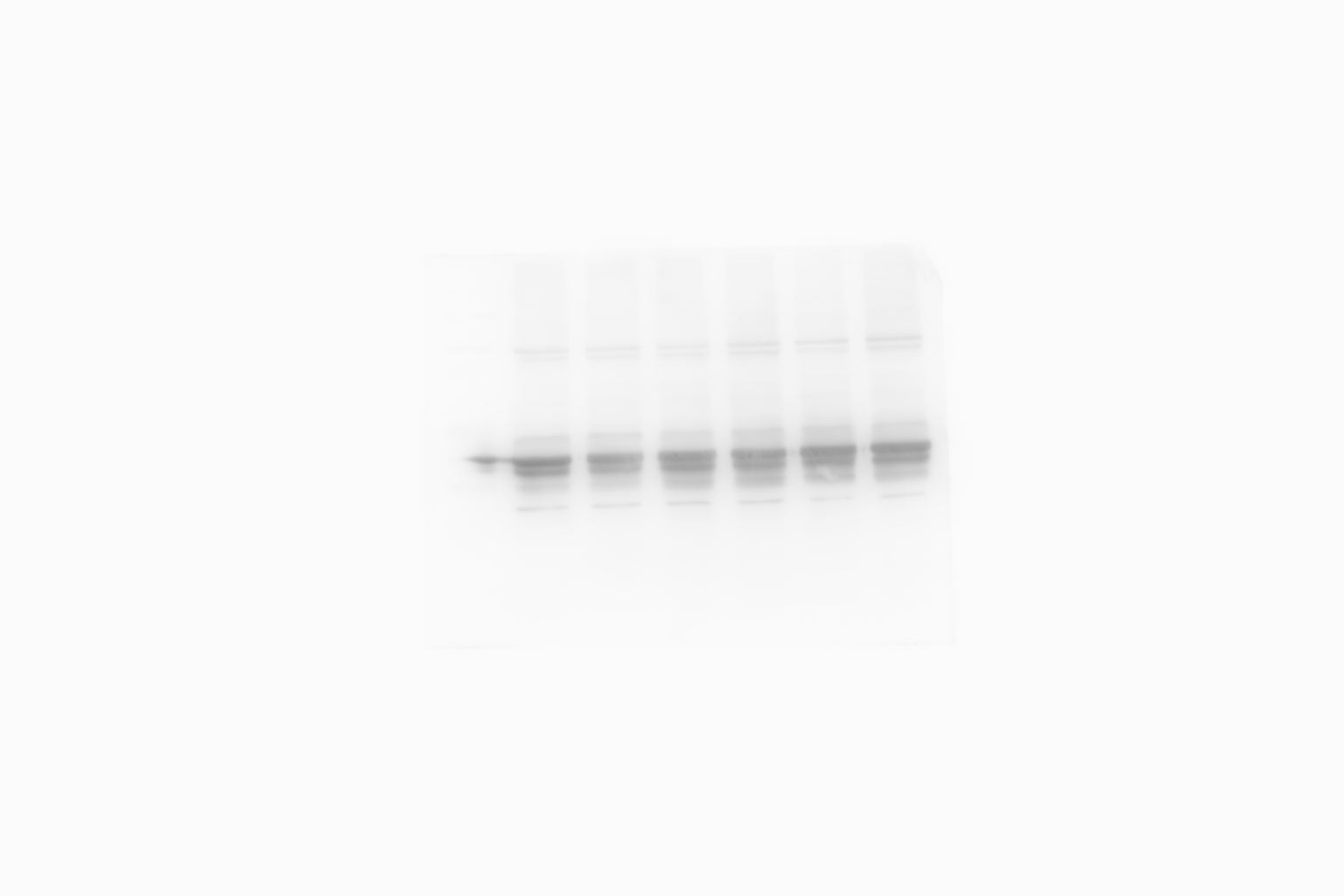

Supplement: Supplementary file 6 — Source data Fig. 4 [file 44318_2025_572_MOESM6_ESM.zip › Figure 4/Figure 4N/B ACTIN 3 SEC.gel.tif]

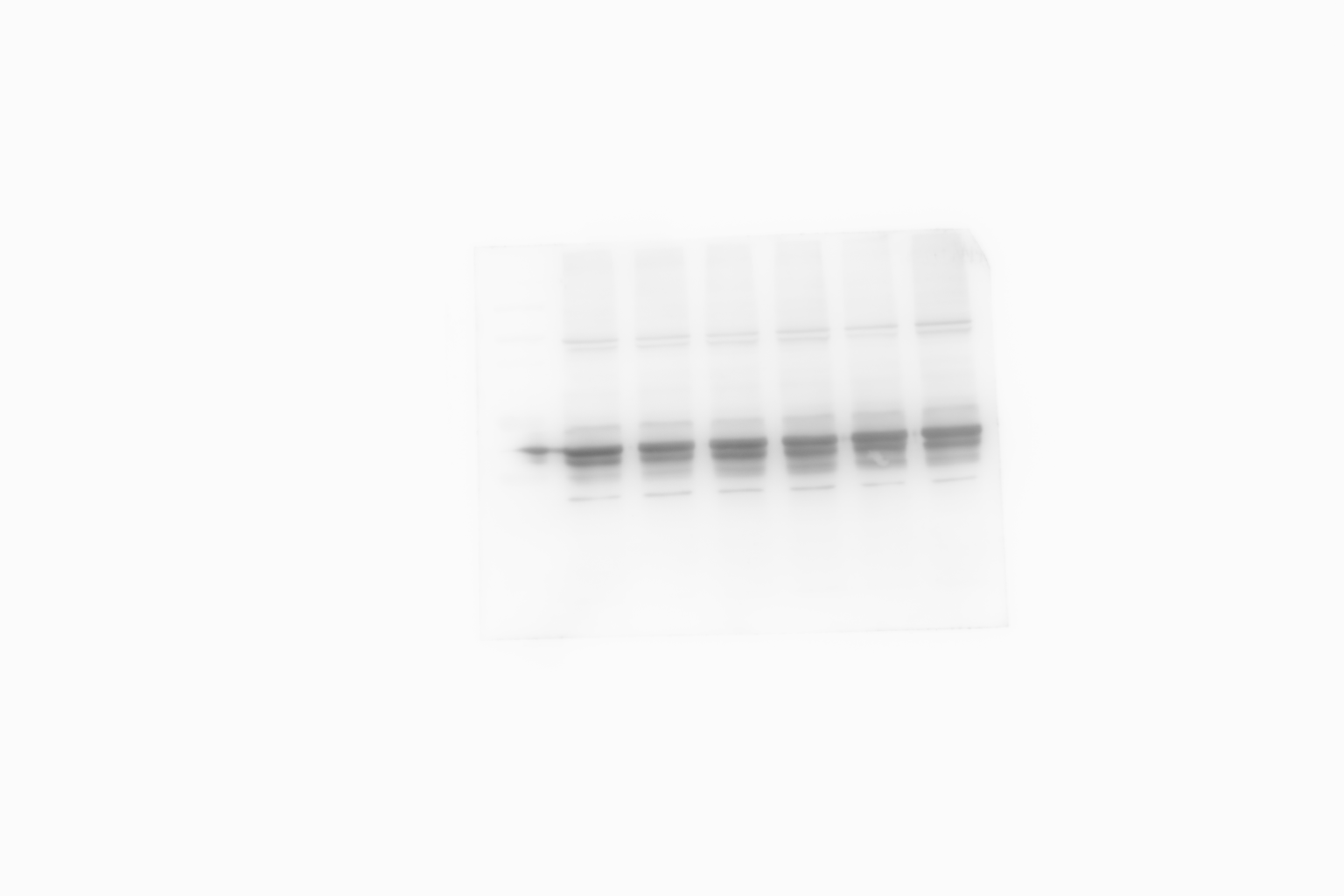

Supplement: Supplementary file 6 — Source data Fig. 4 [file 44318_2025_572_MOESM6_ESM.zip › Figure 4/Figure 4N/B ACTIN 5 SEC.gel]

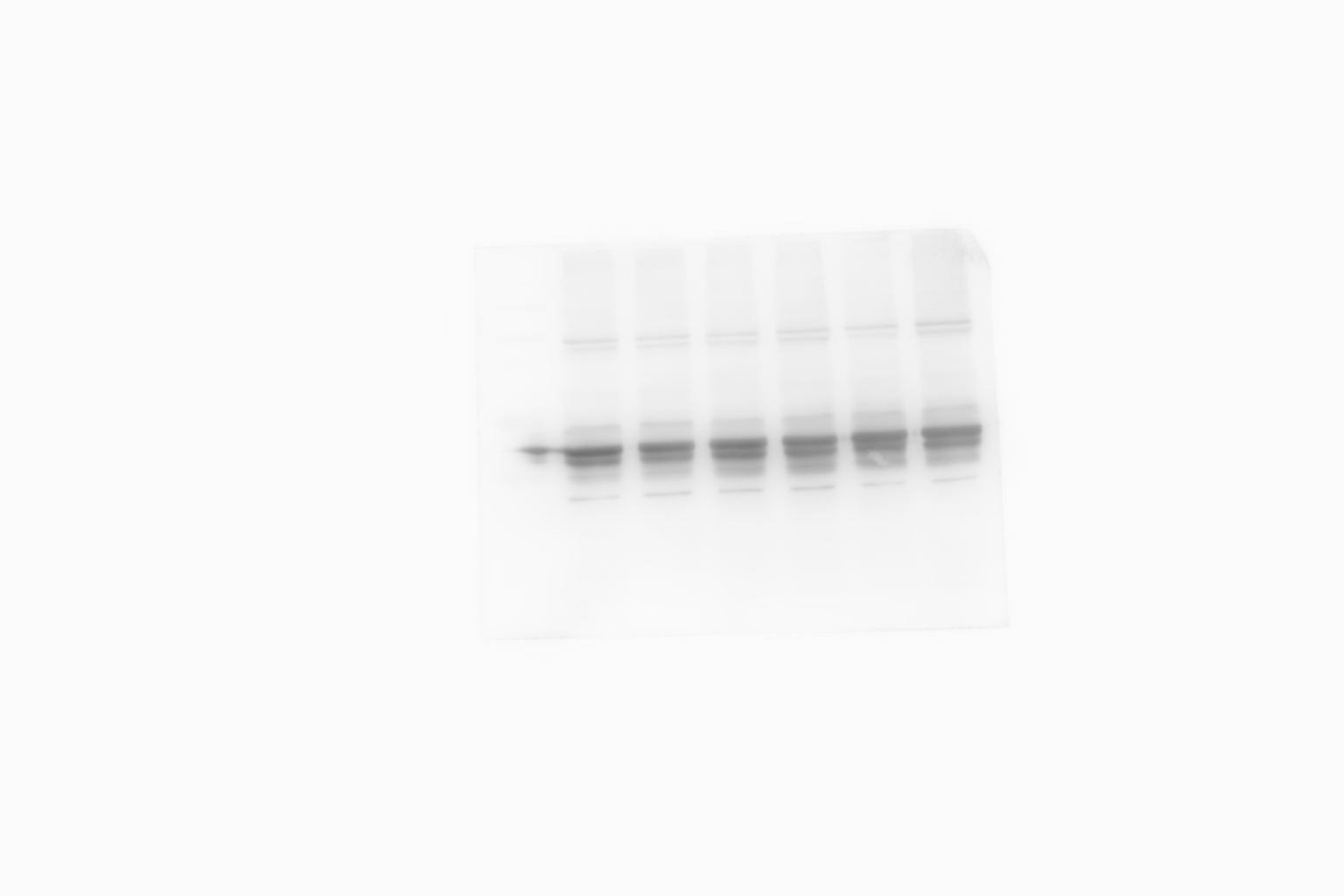

Supplement: Supplementary file 6 — Source data Fig. 4 [file 44318_2025_572_MOESM6_ESM.zip › Figure 4/Figure 4N/B ACTIN 5 SEC.gel.tif]

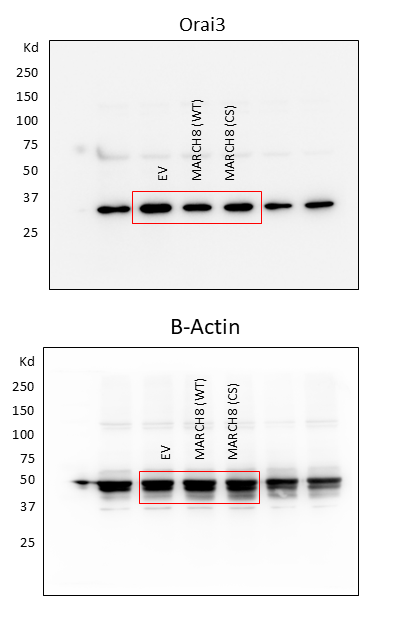

Supplement: Supplementary file 6 — Source data Fig. 4 [file 44318_2025_572_MOESM6_ESM.zip › Figure 4/Figure 4N/Figure 4N.png]

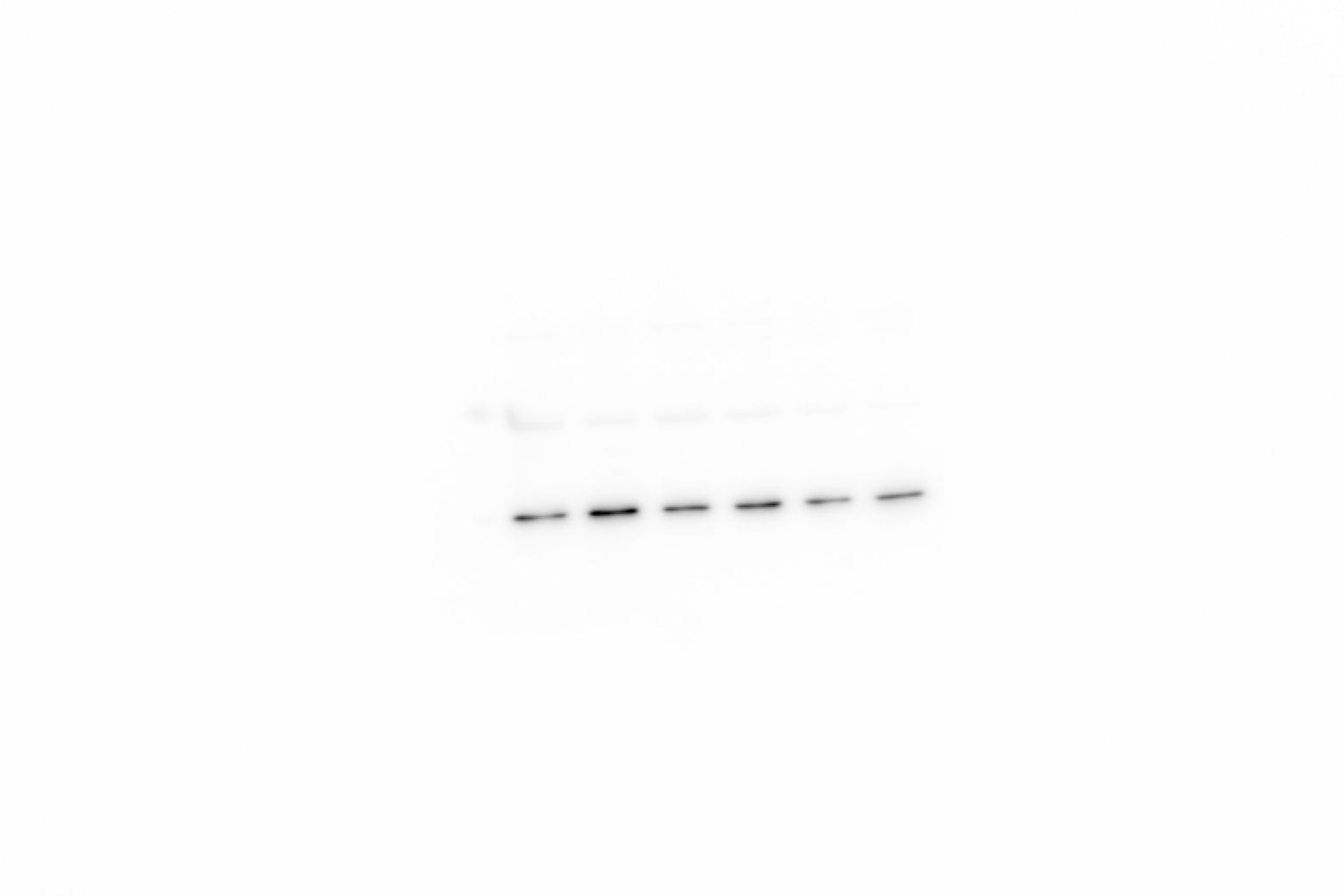

Supplement: Supplementary file 6 — Source data Fig. 4 [file 44318_2025_572_MOESM6_ESM.zip › Figure 4/Figure 4N/Orai3 4 sec.gel]

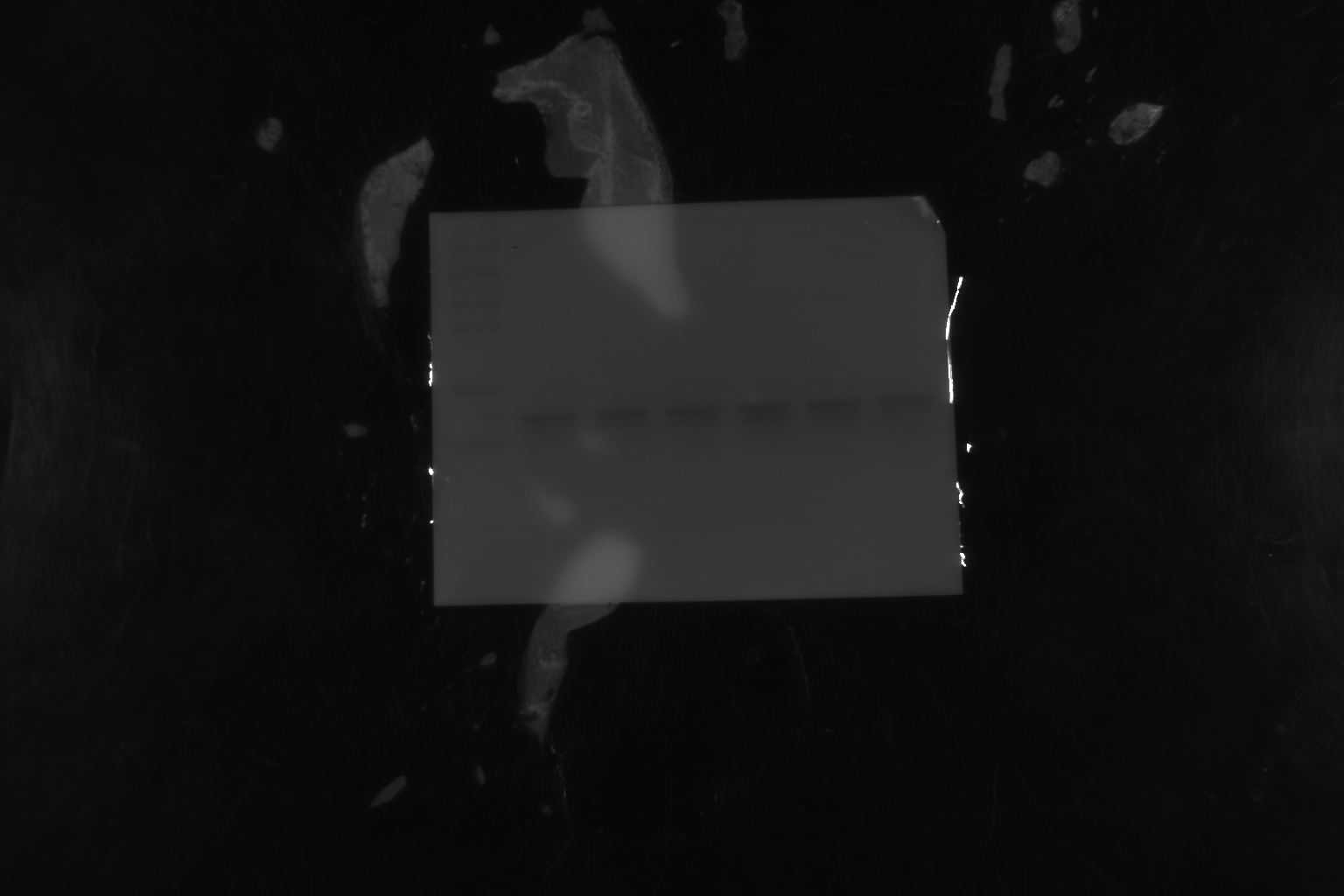

Supplement: Supplementary file 6 — Source data Fig. 4 [file 44318_2025_572_MOESM6_ESM.zip › Figure 4/Figure 4N/V_B ACTIN 2 SEC.gel]

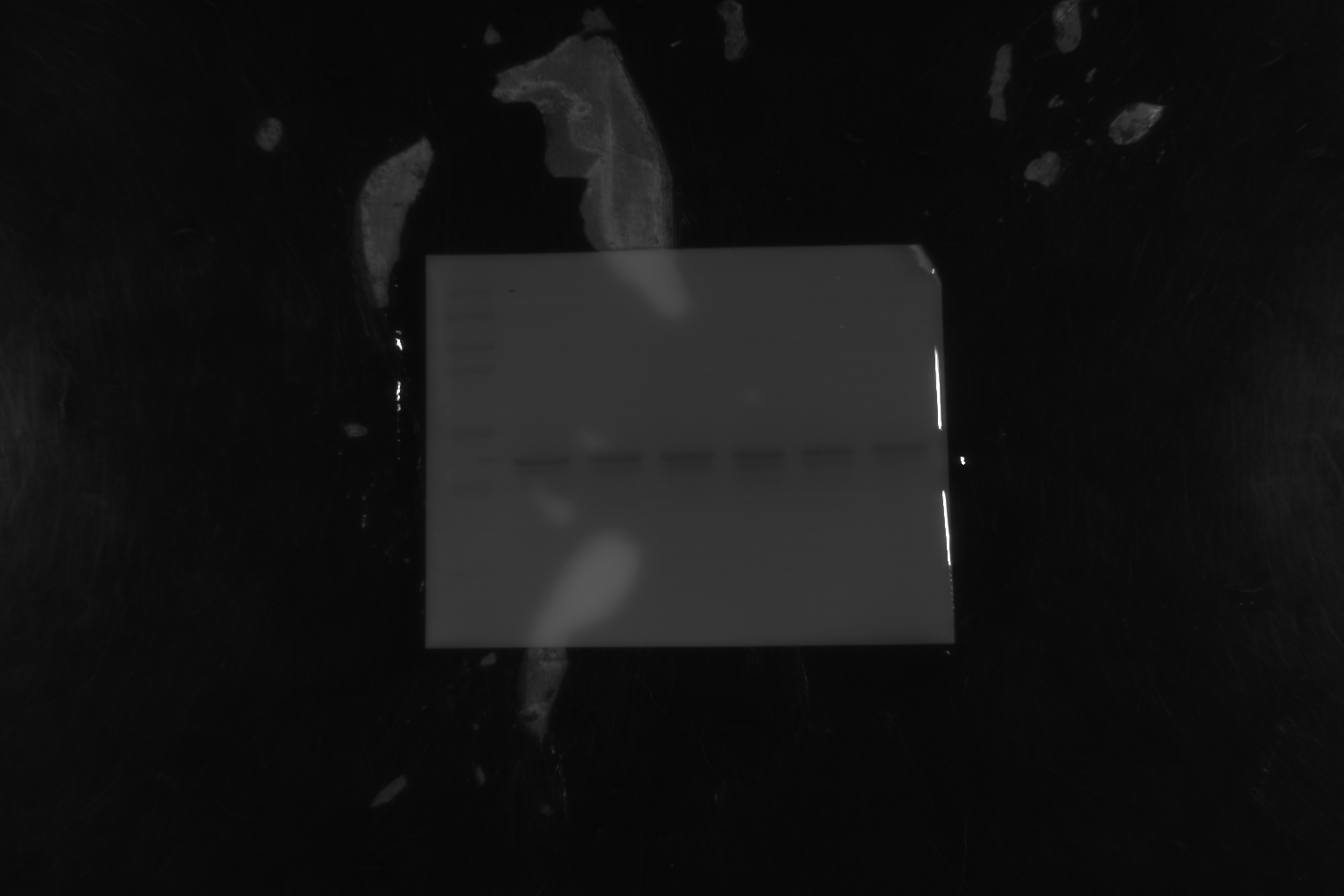

Supplement: Supplementary file 6 — Source data Fig. 4 [file 44318_2025_572_MOESM6_ESM.zip › Figure 4/Figure 4N/V_B ACTIN 3 SEC.gel]

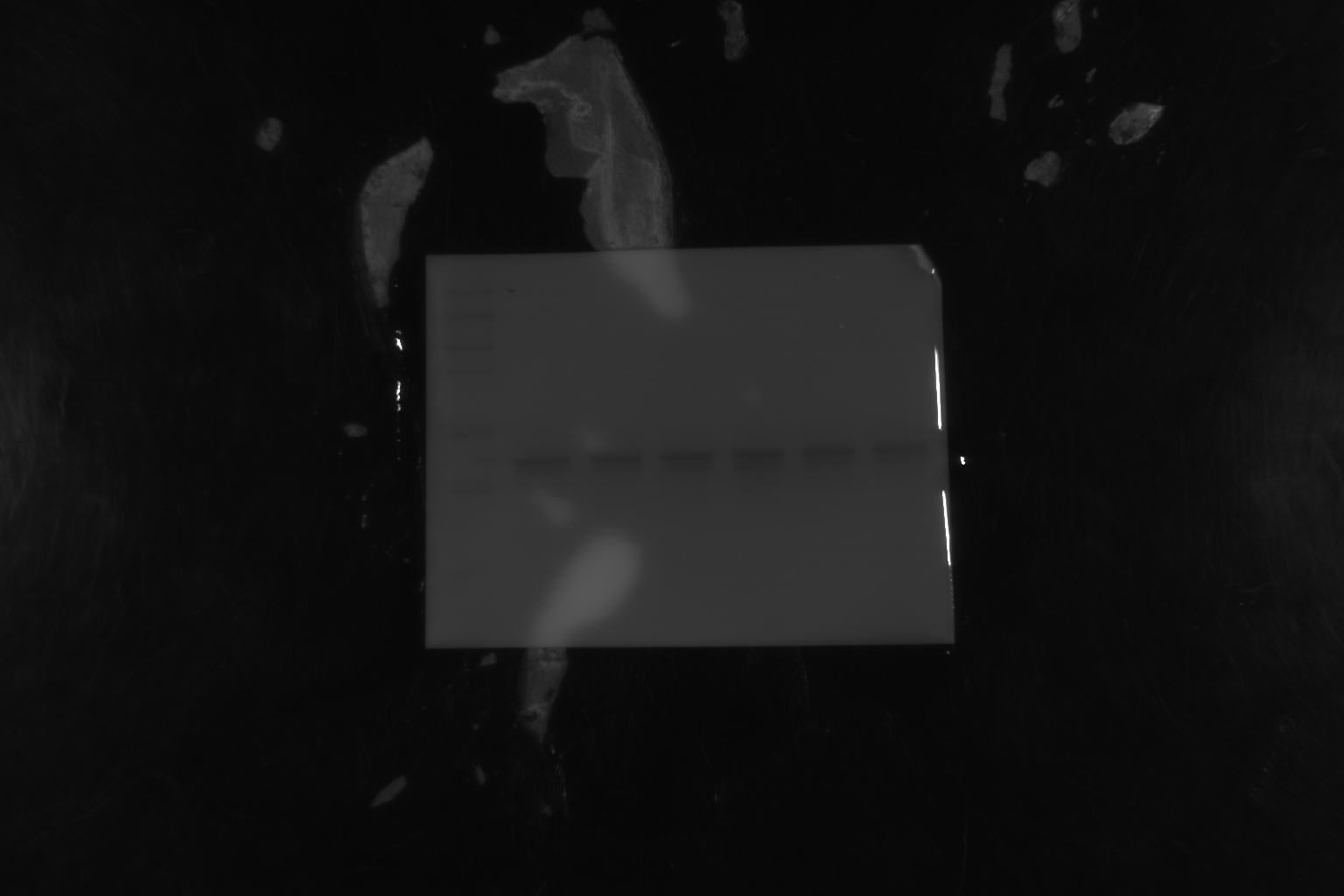

Supplement: Supplementary file 6 — Source data Fig. 4 [file 44318_2025_572_MOESM6_ESM.zip › Figure 4/Figure 4N/V_B ACTIN 3 SEC.gel.tif]

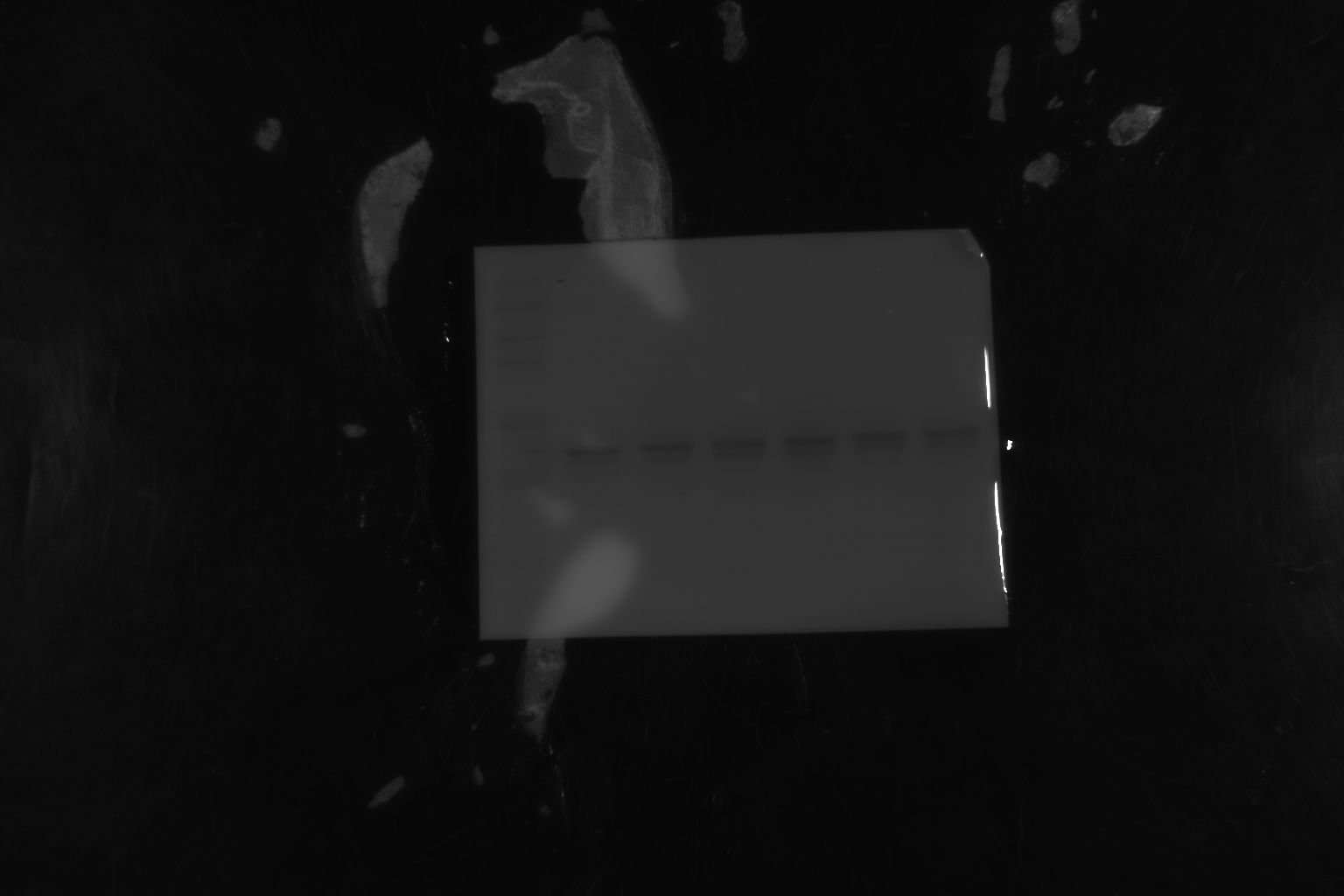

Supplement: Supplementary file 6 — Source data Fig. 4 [file 44318_2025_572_MOESM6_ESM.zip › Figure 4/Figure 4N/V_B ACTIN 5 SEC.gel]

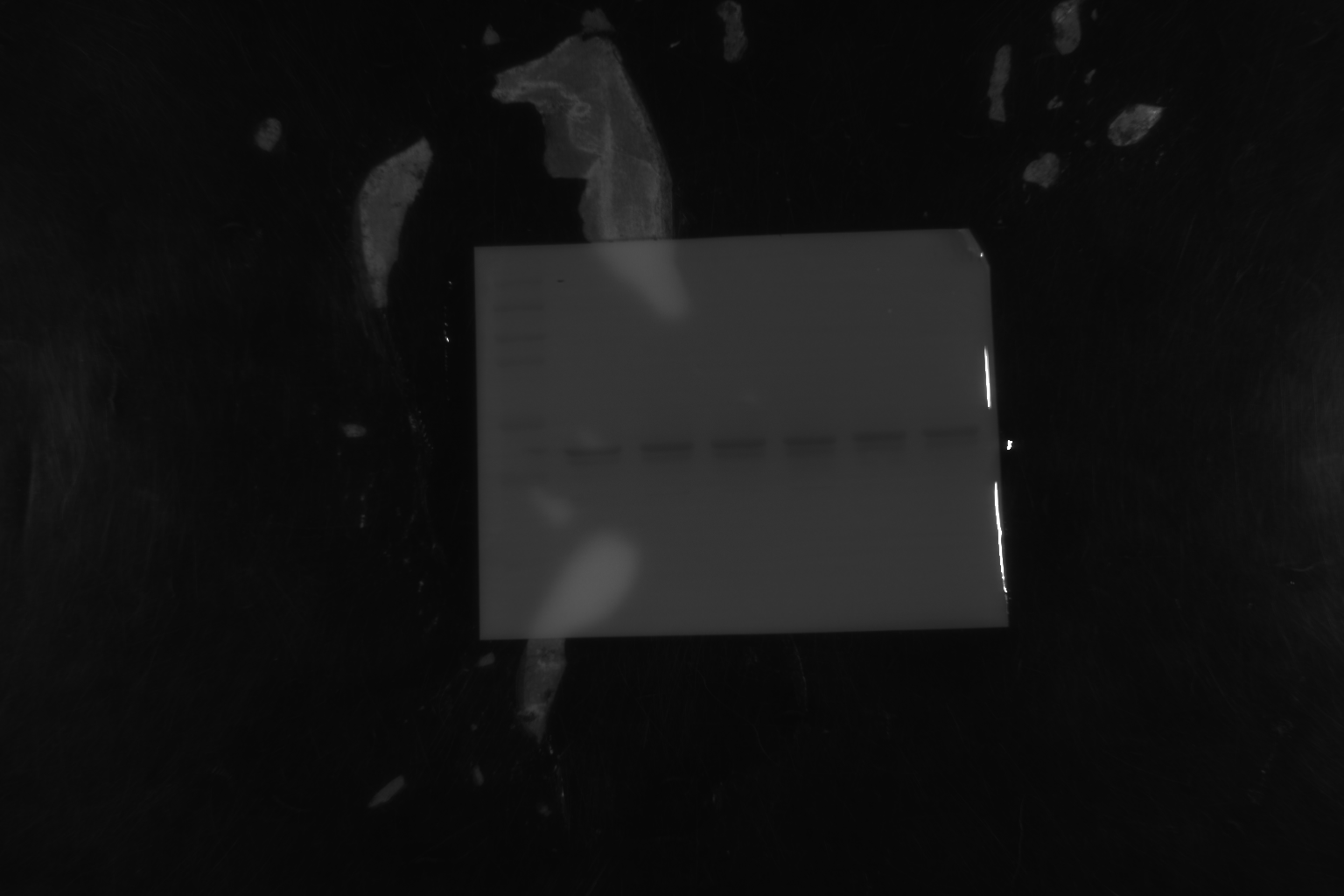

Supplement: Supplementary file 6 — Source data Fig. 4 [file 44318_2025_572_MOESM6_ESM.zip › Figure 4/Figure 4N/V_B ACTIN 5 SEC.gel.tif]

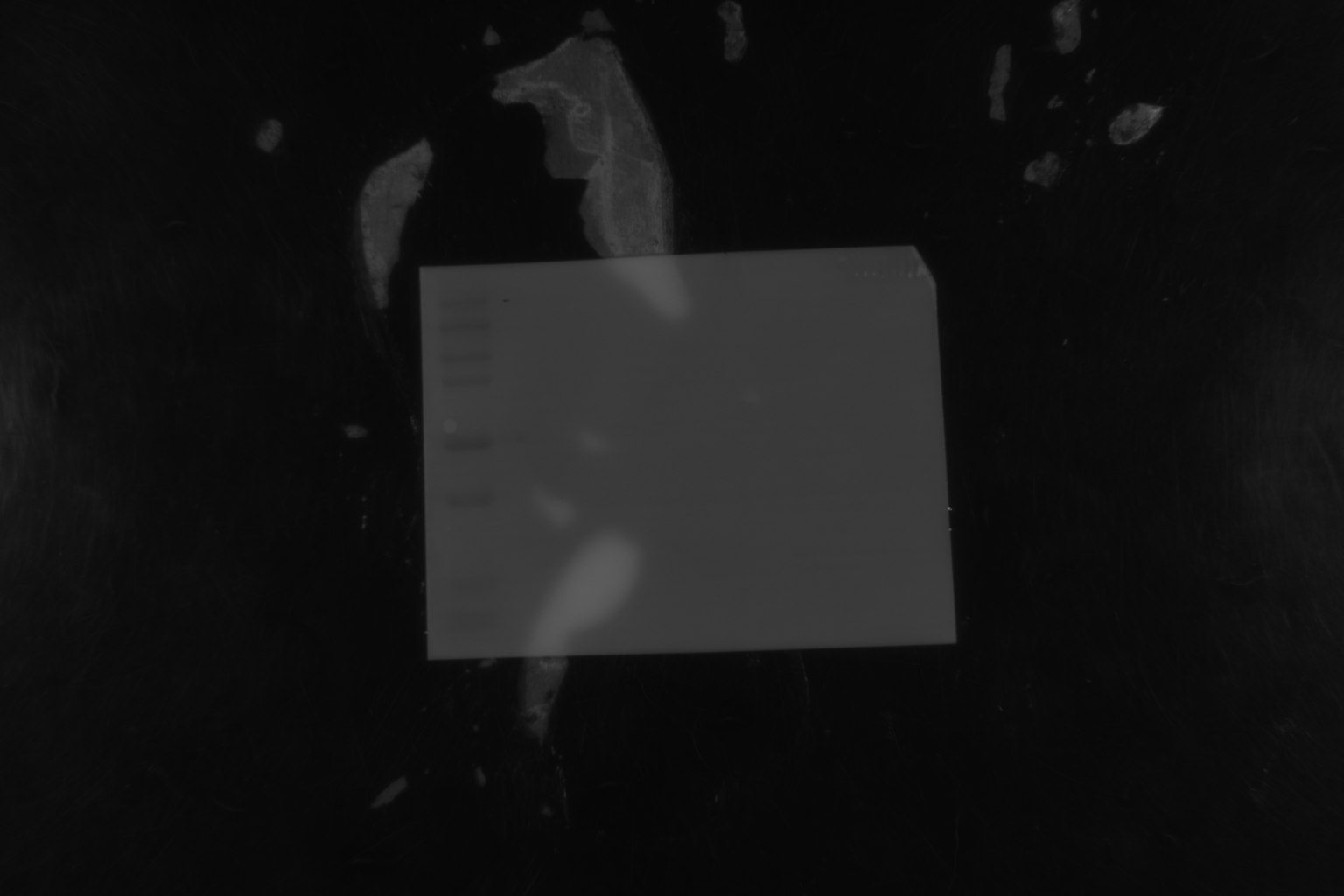

Supplement: Supplementary file 6 — Source data Fig. 4 [file 44318_2025_572_MOESM6_ESM.zip › Figure 4/Figure 4N/V_Orai3 4 sec.gel]

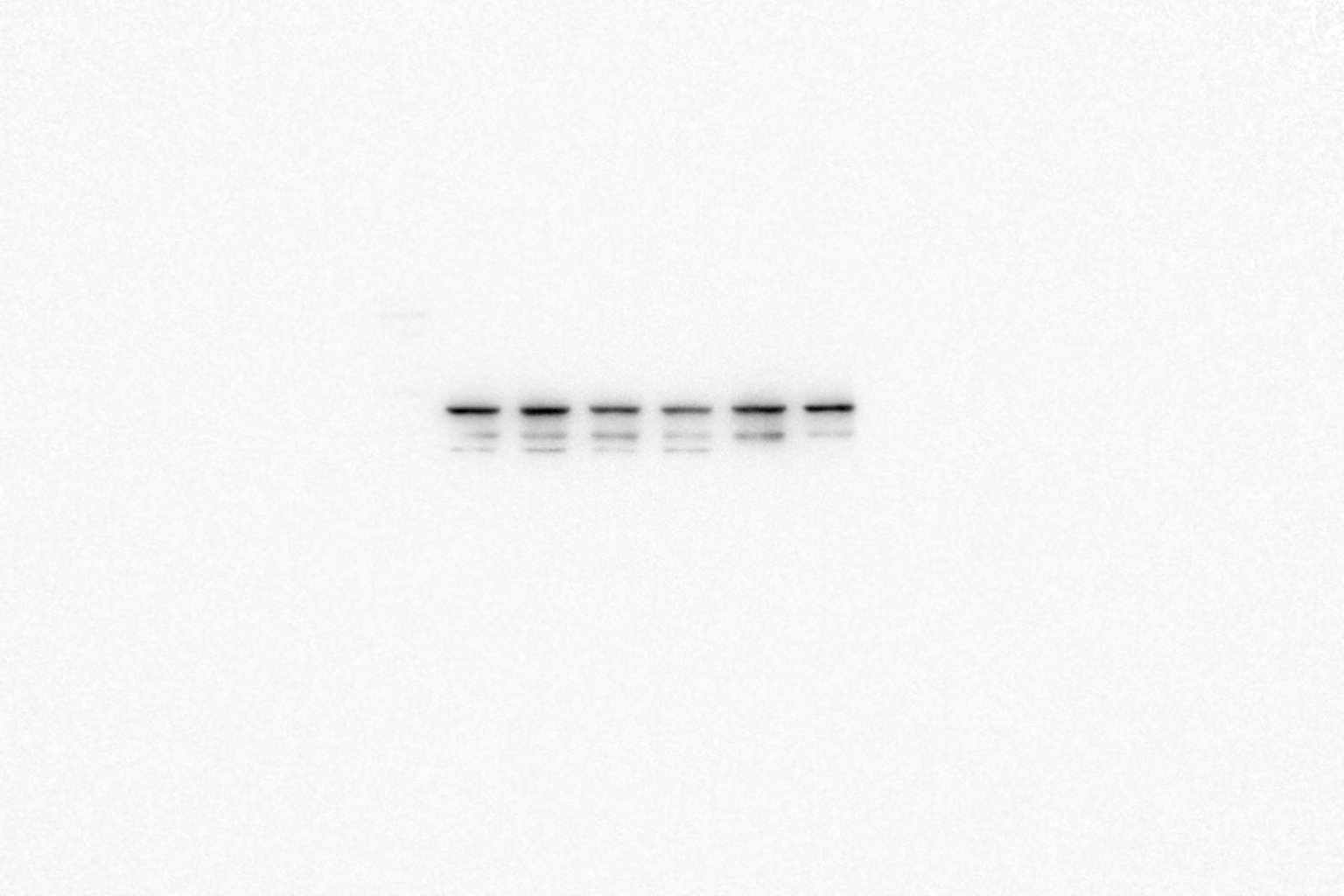

Supplement: Supplementary file 7 — Source data Fig. 5 [file 44318_2025_572_MOESM7_ESM.zip › Figure 5/Figure 5A/B Actin 1 sec.gel]

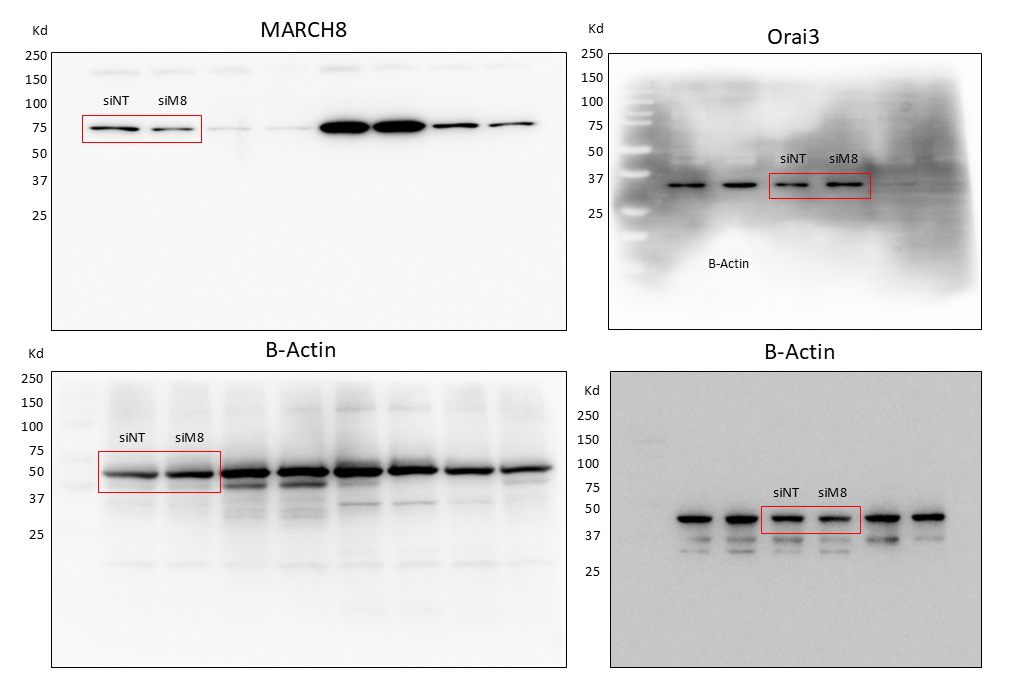

Supplement: Supplementary file 7 — Source data Fig. 5 [file 44318_2025_572_MOESM7_ESM.zip › Figure 5/Figure 5A/Figure 5A.png]

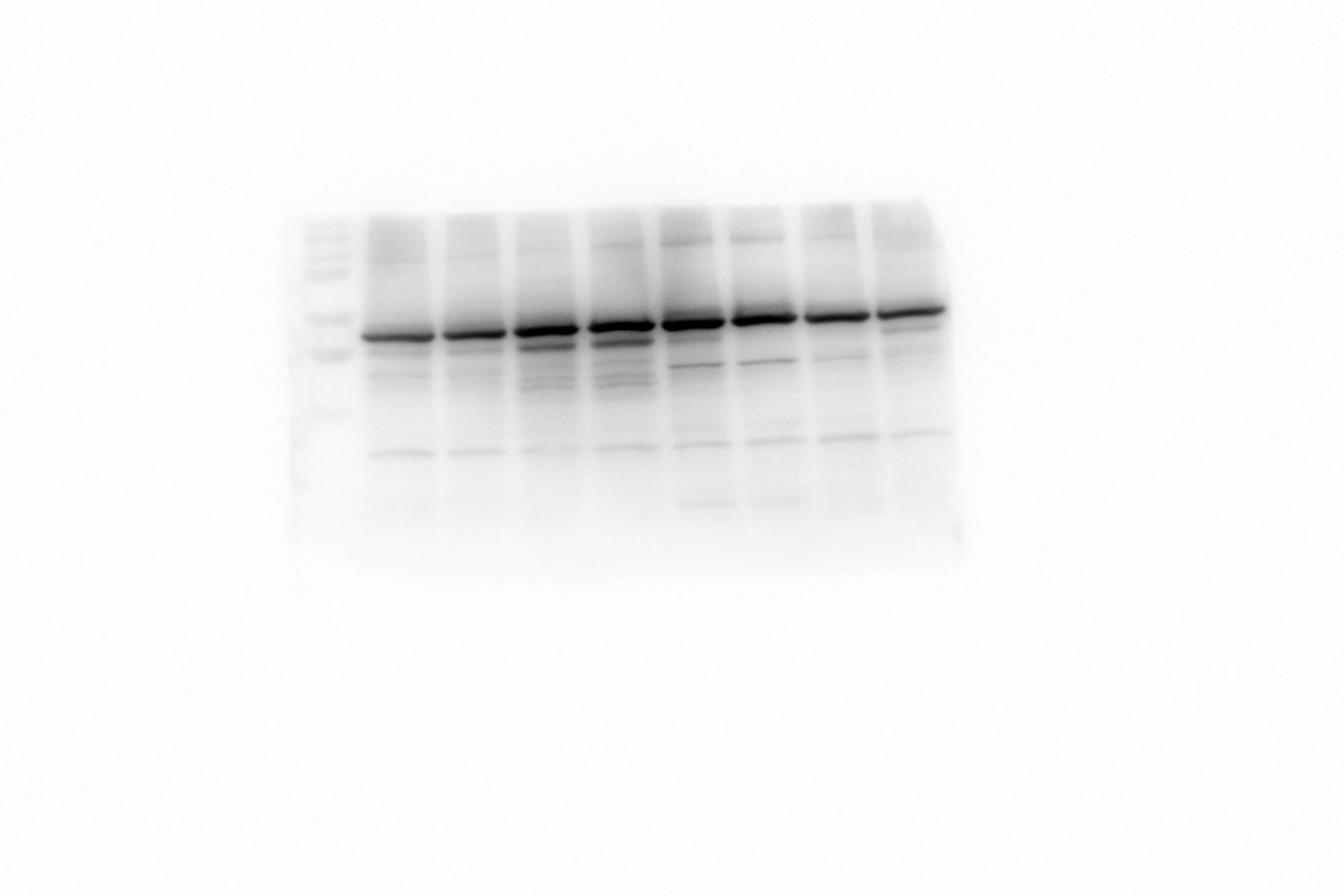

Supplement: Supplementary file 7 — Source data Fig. 5 [file 44318_2025_572_MOESM7_ESM.zip › Figure 5/Figure 5A/M8 KD B ACTIN 0.5 SEC.gel]

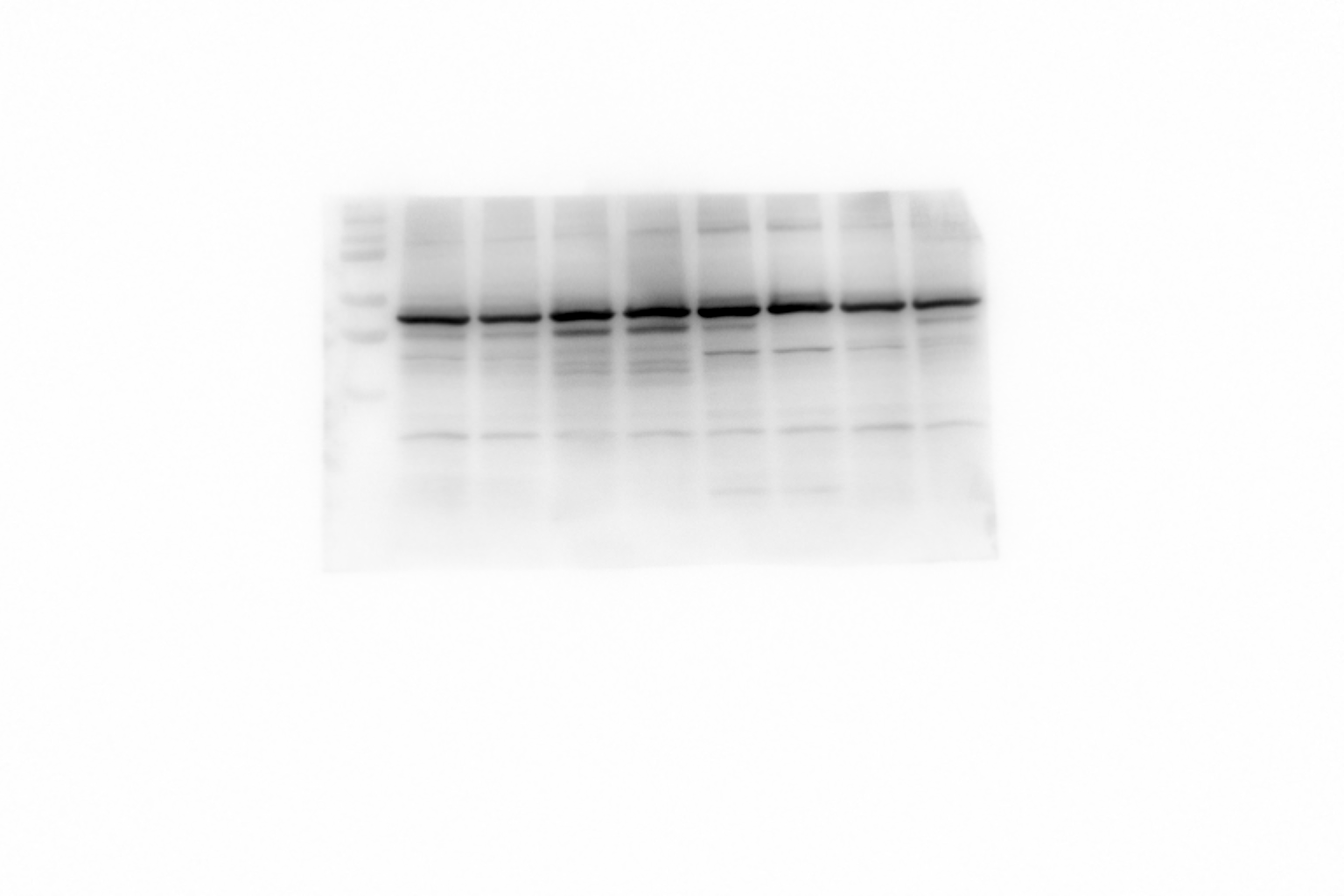

Supplement: Supplementary file 7 — Source data Fig. 5 [file 44318_2025_572_MOESM7_ESM.zip › Figure 5/Figure 5A/M8 KD B ACTIN 1 SEC.gel]

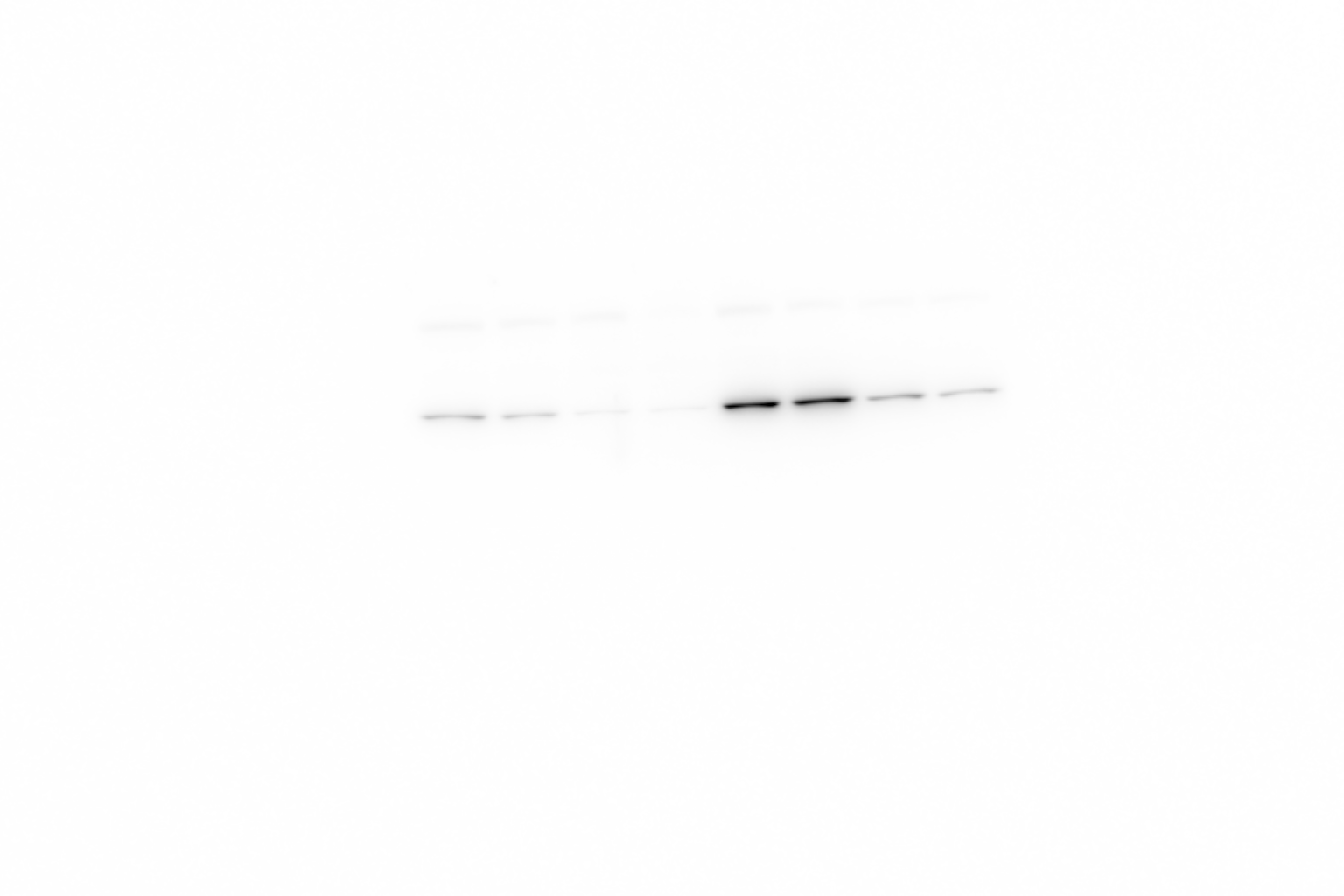

Supplement: Supplementary file 7 — Source data Fig. 5 [file 44318_2025_572_MOESM7_ESM.zip › Figure 5/Figure 5A/M8 KD MARCH 8 4 SEC.gel]

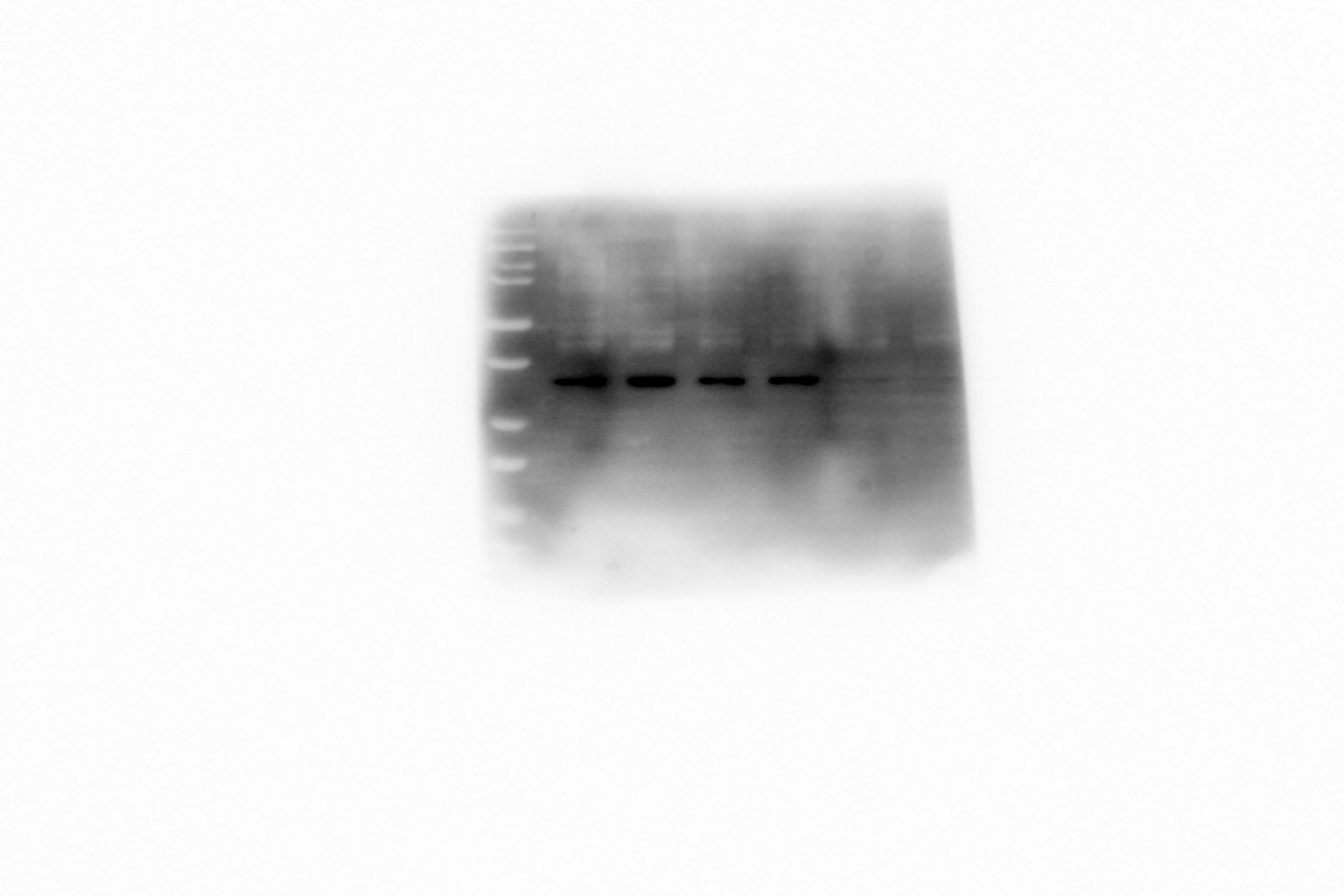

Supplement: Supplementary file 7 — Source data Fig. 5 [file 44318_2025_572_MOESM7_ESM.zip › Figure 5/Figure 5A/ORAI3 2 SEC.gel]

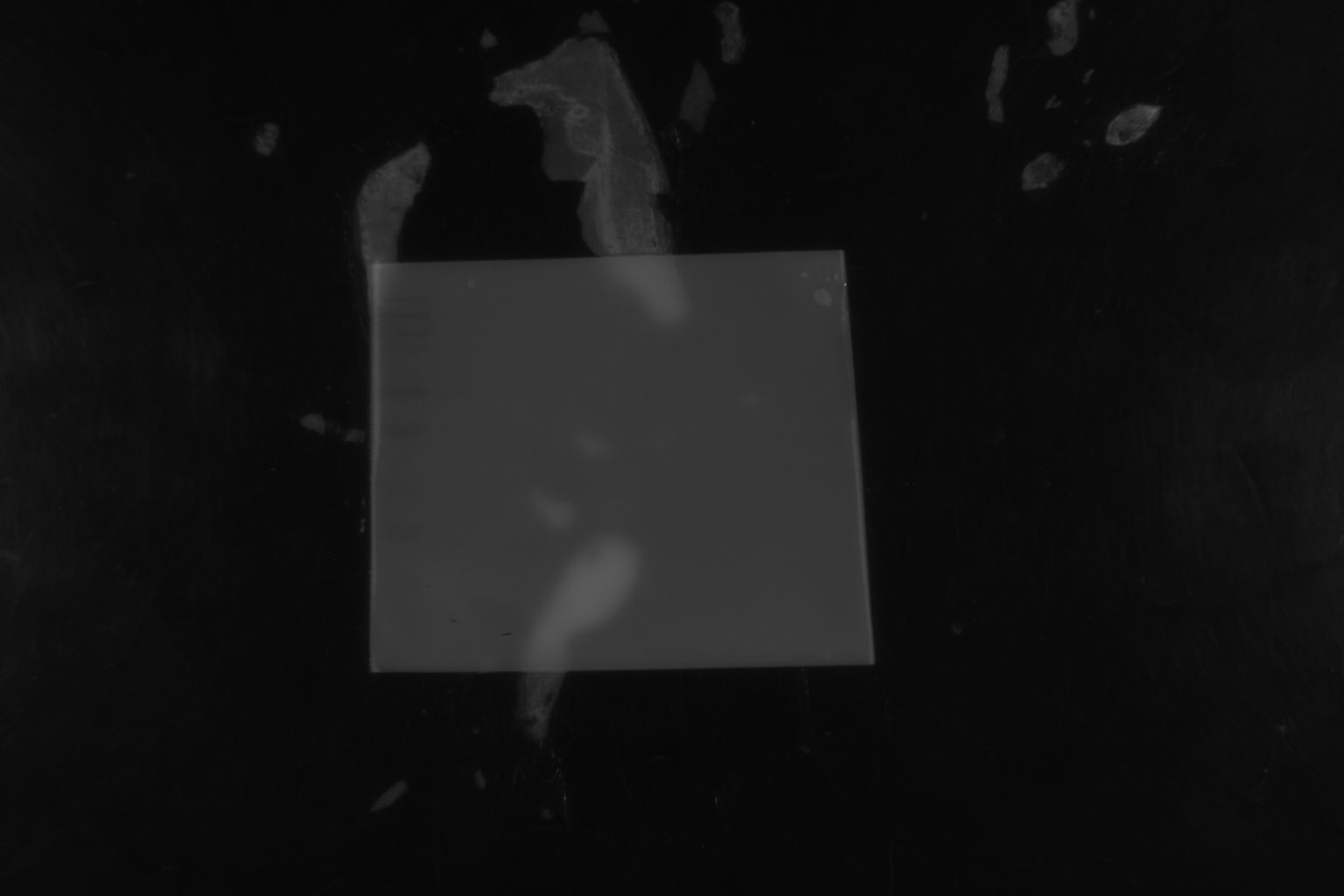

Supplement: Supplementary file 7 — Source data Fig. 5 [file 44318_2025_572_MOESM7_ESM.zip › Figure 5/Figure 5A/V_B Actin 1 sec.gel]

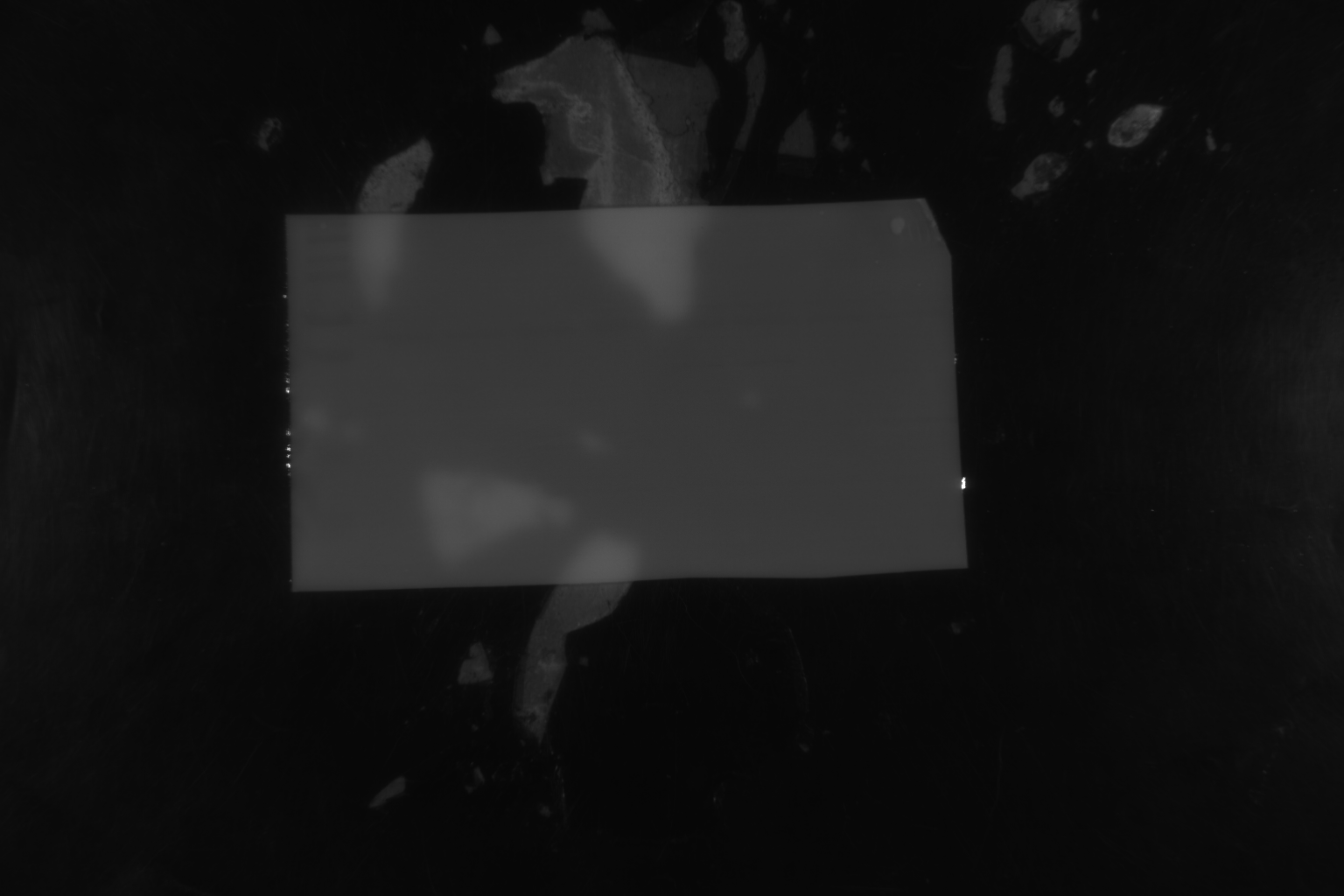

Supplement: Supplementary file 7 — Source data Fig. 5 [file 44318_2025_572_MOESM7_ESM.zip › Figure 5/Figure 5A/V_M8 KD B ACTIN 0.5 SEC.gel]

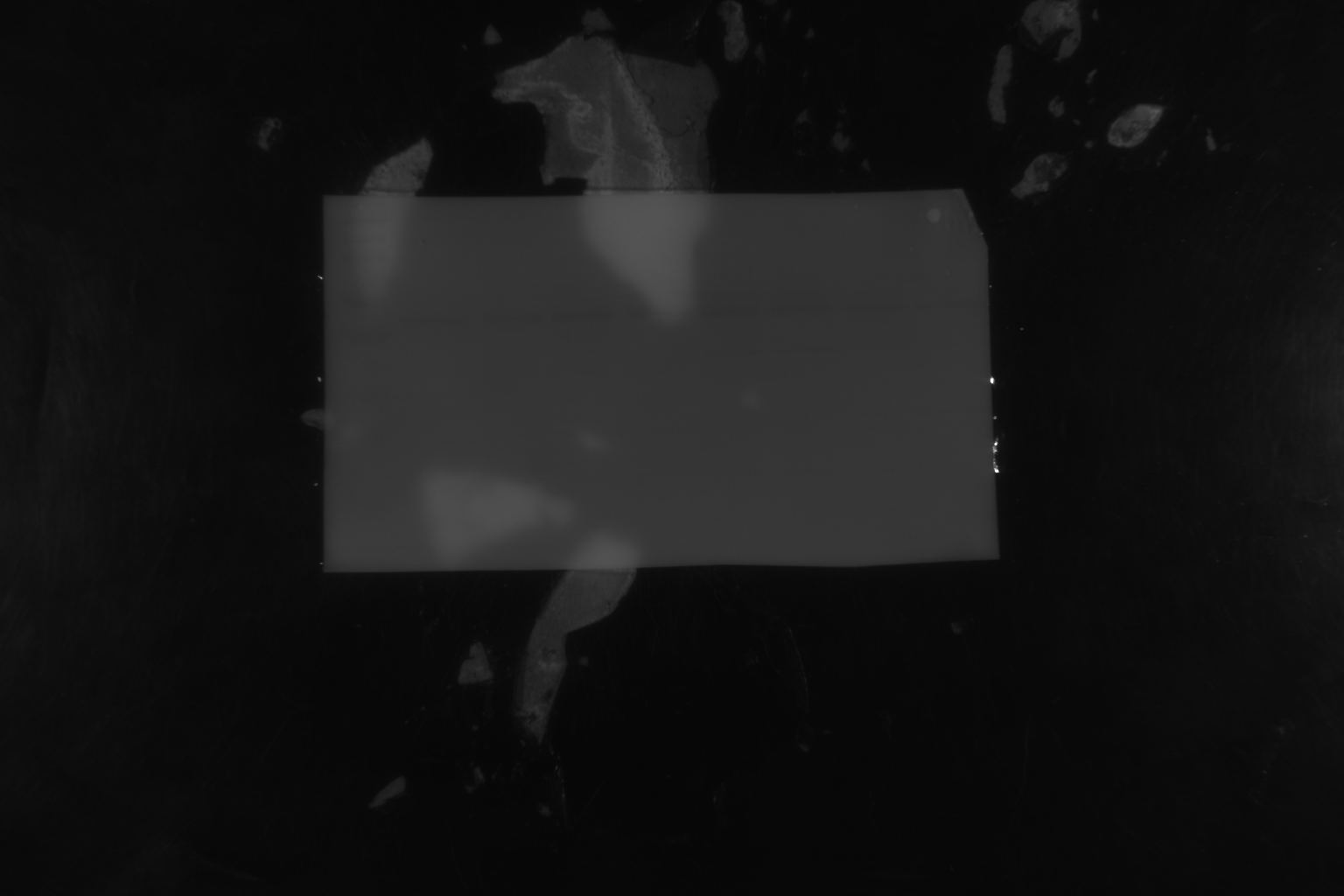

Supplement: Supplementary file 7 — Source data Fig. 5 [file 44318_2025_572_MOESM7_ESM.zip › Figure 5/Figure 5A/V_M8 KD B ACTIN 1 SEC.gel]

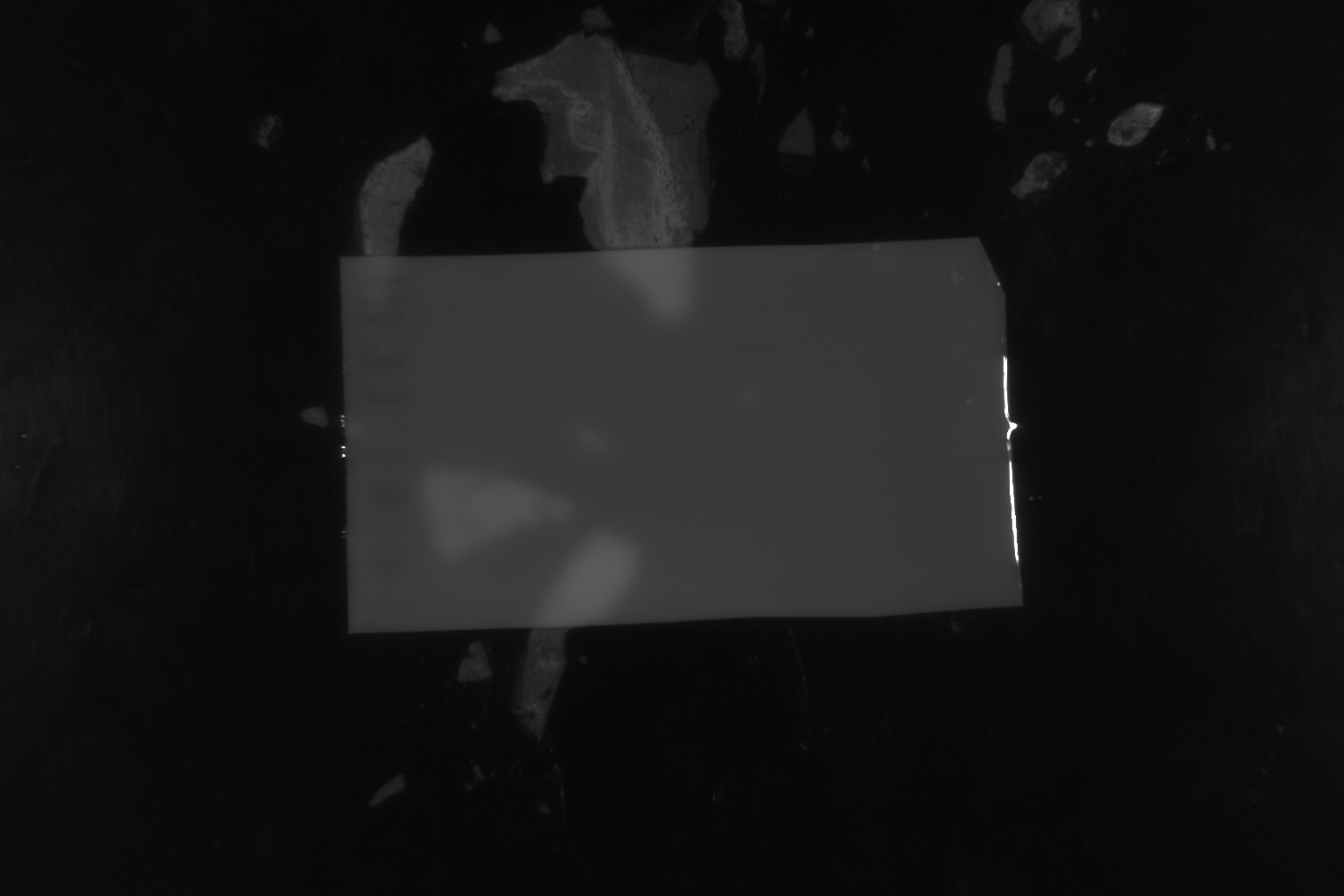

Supplement: Supplementary file 7 — Source data Fig. 5 [file 44318_2025_572_MOESM7_ESM.zip › Figure 5/Figure 5A/V_M8 KD ORAI 3 4 SEC.gel]

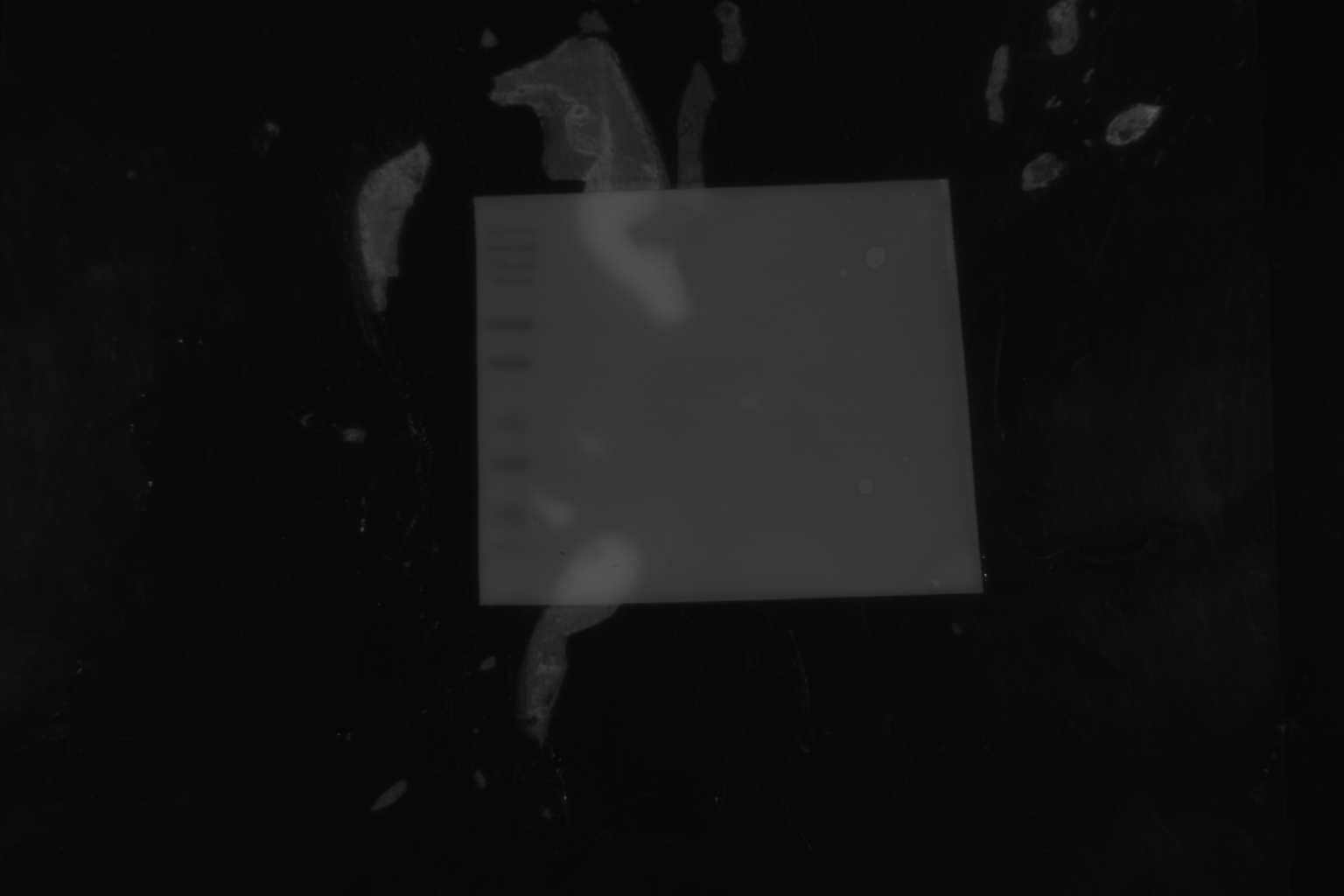

Supplement: Supplementary file 7 — Source data Fig. 5 [file 44318_2025_572_MOESM7_ESM.zip › Figure 5/Figure 5A/V_ORAI3 2 SEC.gel]

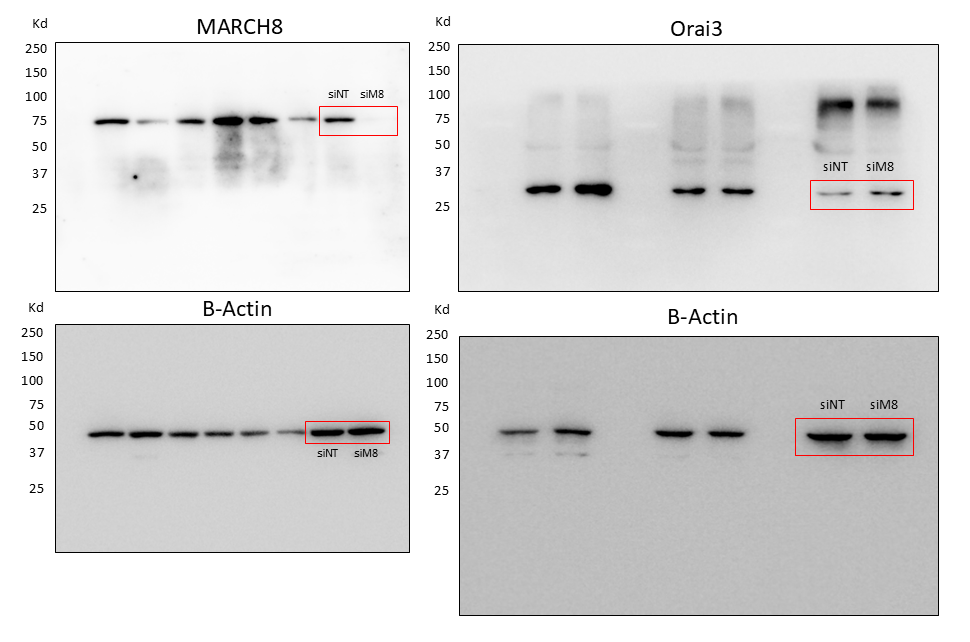

Supplement: Supplementary file 7 — Source data Fig. 5 [file 44318_2025_572_MOESM7_ESM.zip › Figure 5/Figure 5E/Figure 5E.png]

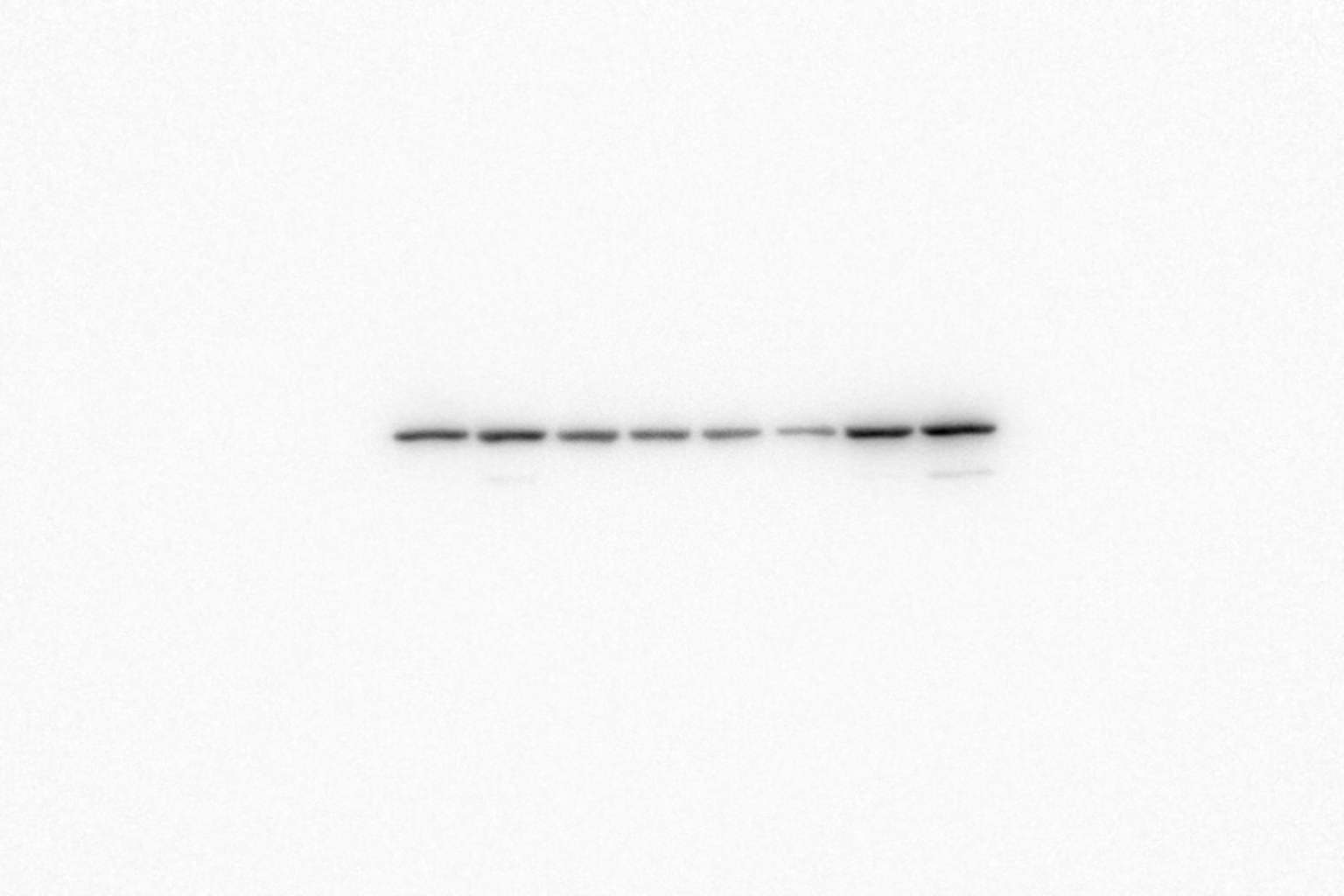

Supplement: Supplementary file 7 — Source data Fig. 5 [file 44318_2025_572_MOESM7_ESM.zip › Figure 5/Figure 5E/siM8 B ACTIN 0.5 SEC.gel]

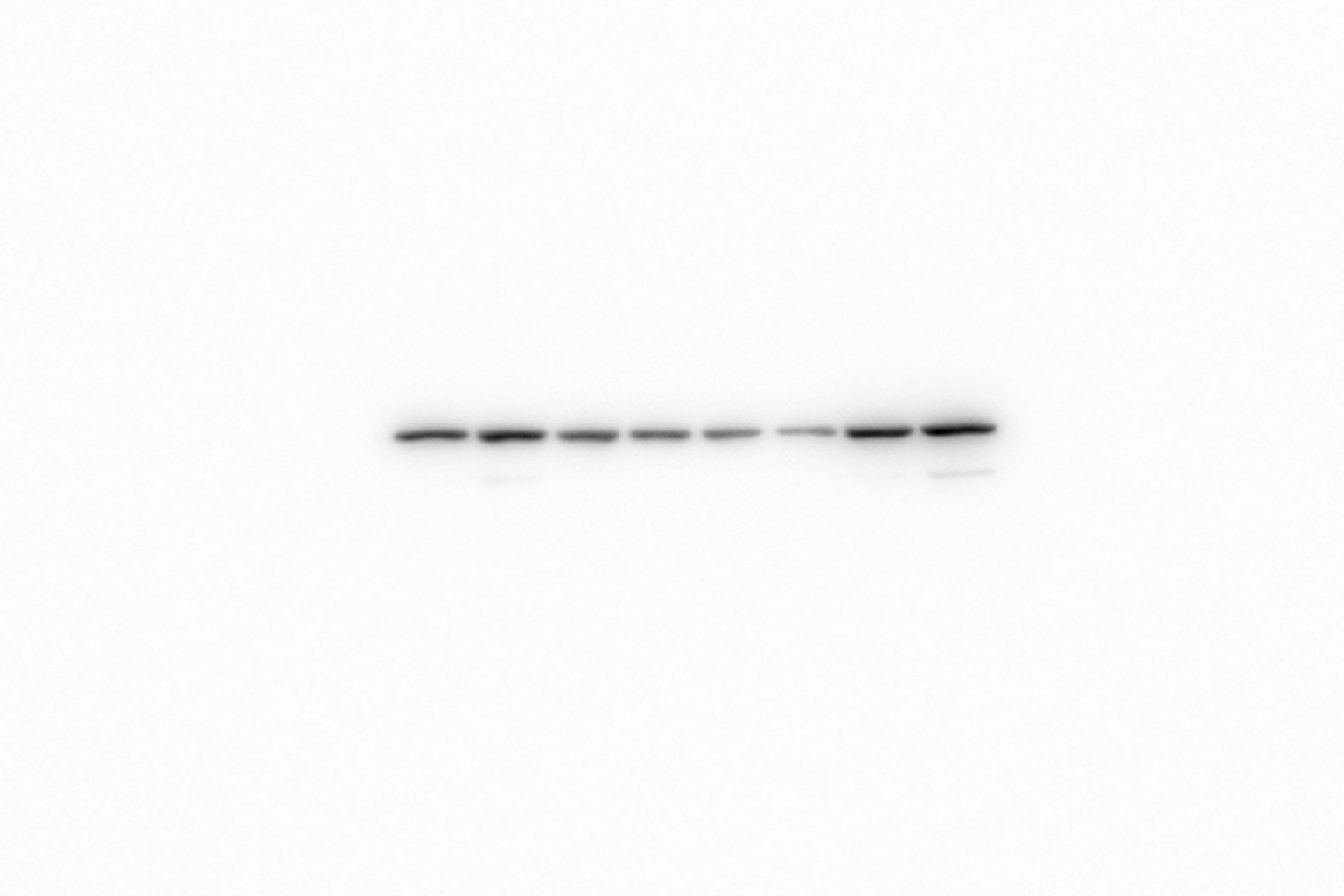

Supplement: Supplementary file 7 — Source data Fig. 5 [file 44318_2025_572_MOESM7_ESM.zip › Figure 5/Figure 5E/siM8 B ACTIN 1 SEC (2).gel]
