# Supplementary figures and images for: NFATc1 drives Orai3 transcription and proteolysis by harnessing epigenome differences in the MARCH8 promoter (part 2 of 3)
Source: EMBO J. 2025 Sep 29;44(21):6137–67. doi: 10.1038/s44318-025-00572-4 (PMC12583688; doi:10.1038/s44318-025-00572-4)

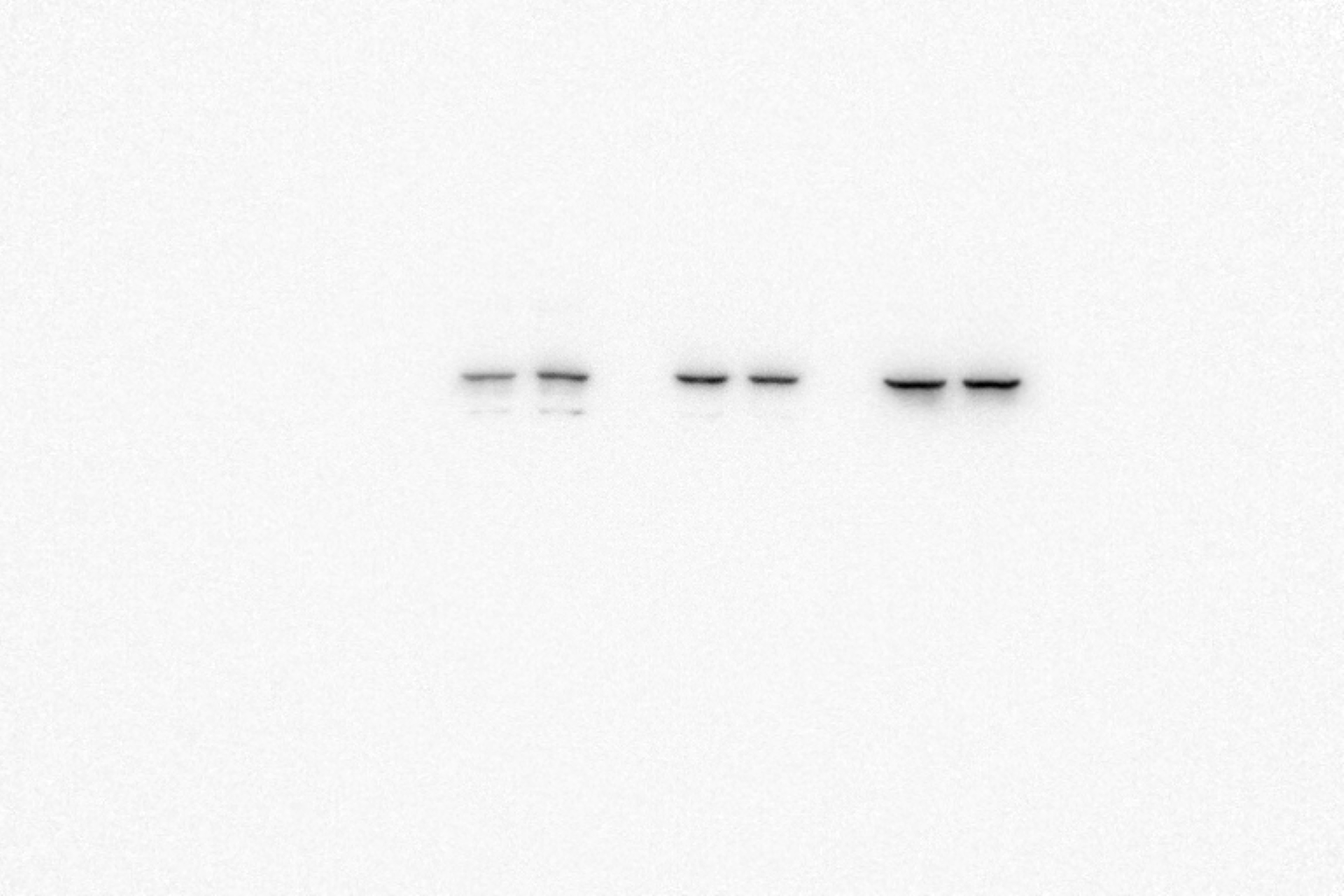

Supplement: Supplementary file 7 — Source data Fig. 5 [file 44318_2025_572_MOESM7_ESM.zip › Figure 5/Figure 5E/sim8 B ACTIN 1 SEC.gel]

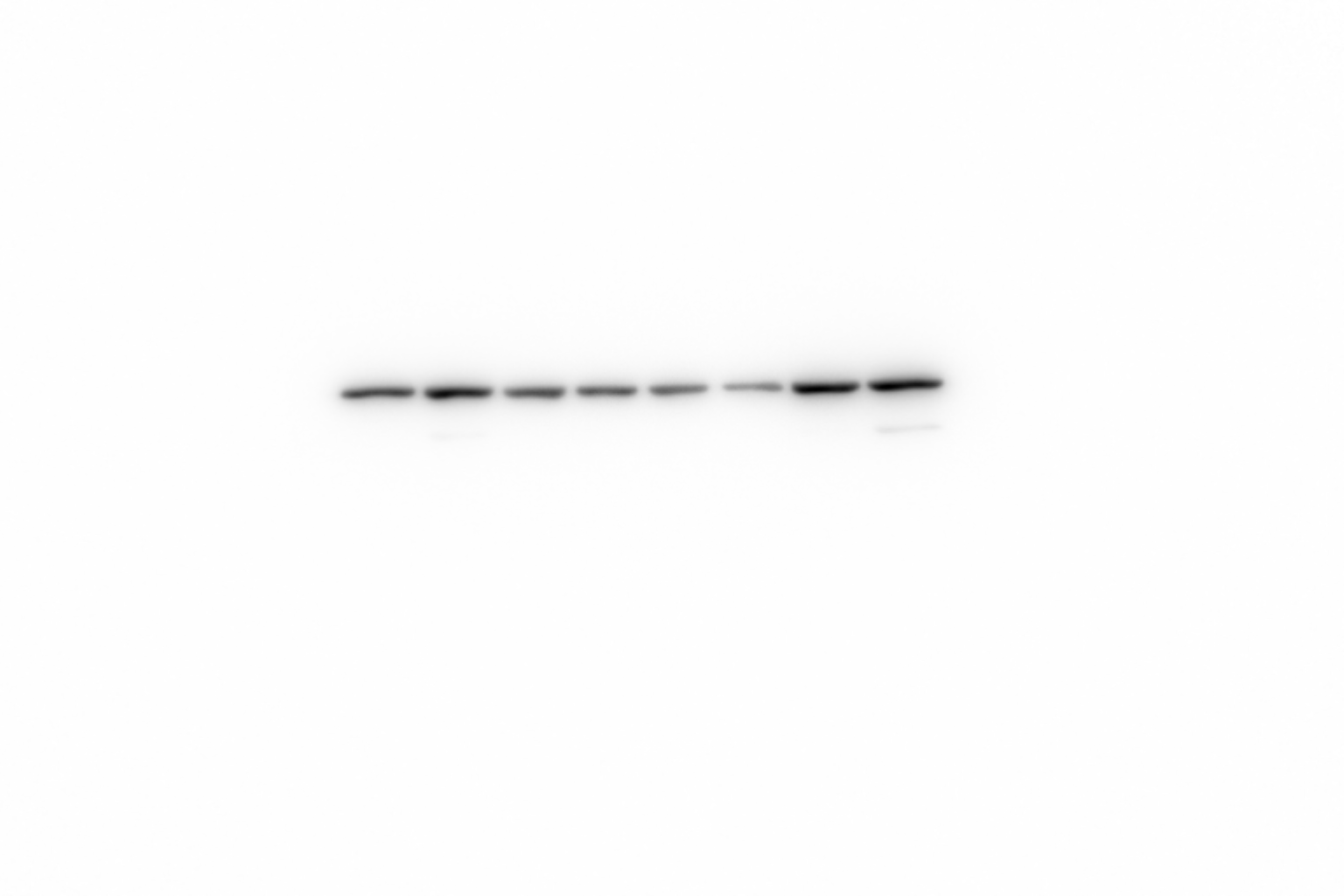

Supplement: Supplementary file 7 — Source data Fig. 5 [file 44318_2025_572_MOESM7_ESM.zip › Figure 5/Figure 5E/siM8 B ACTIN 2 SEC (2).gel]

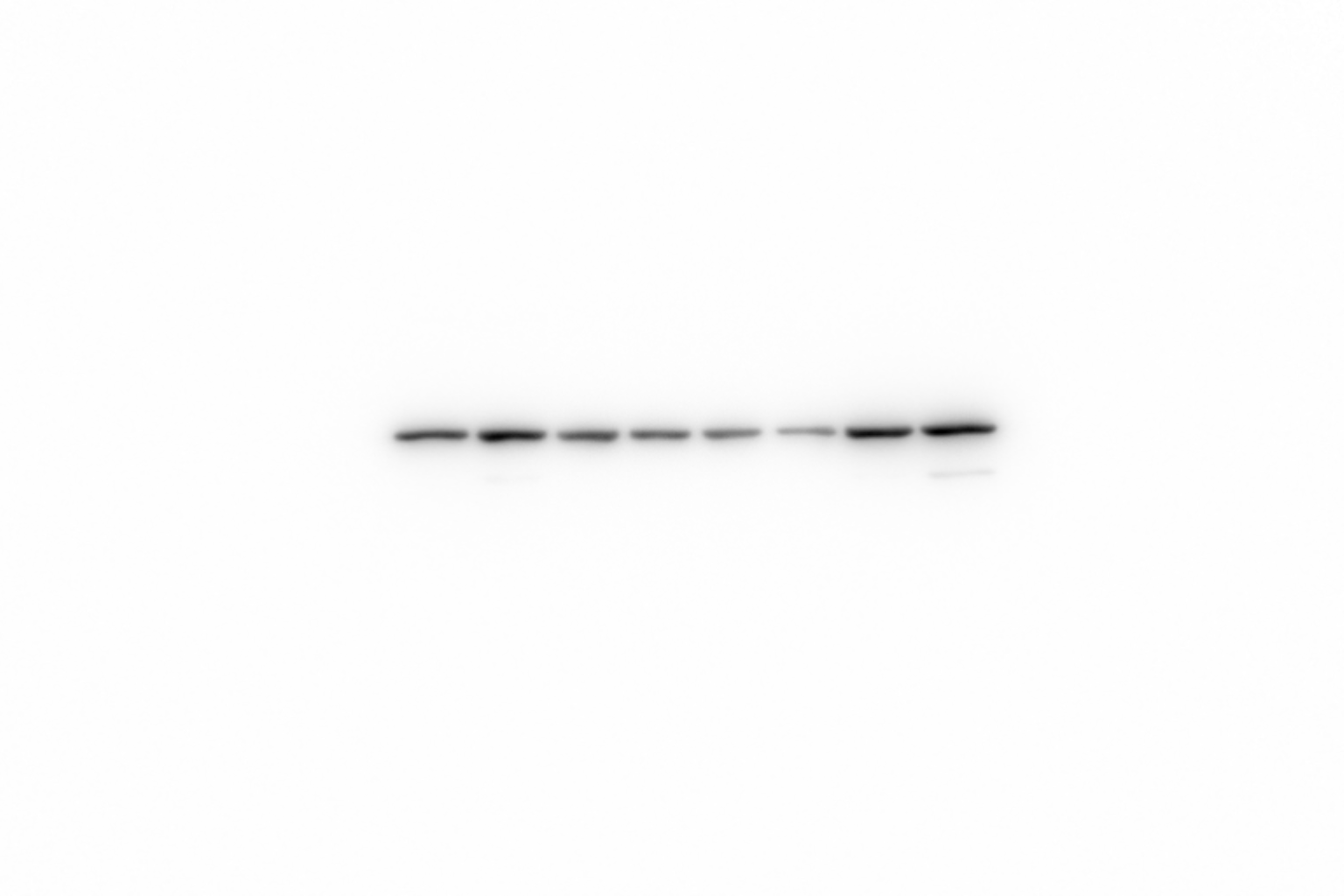

Supplement: Supplementary file 7 — Source data Fig. 5 [file 44318_2025_572_MOESM7_ESM.zip › Figure 5/Figure 5E/siM8 B ACTIN 2 SEC 2.gel]

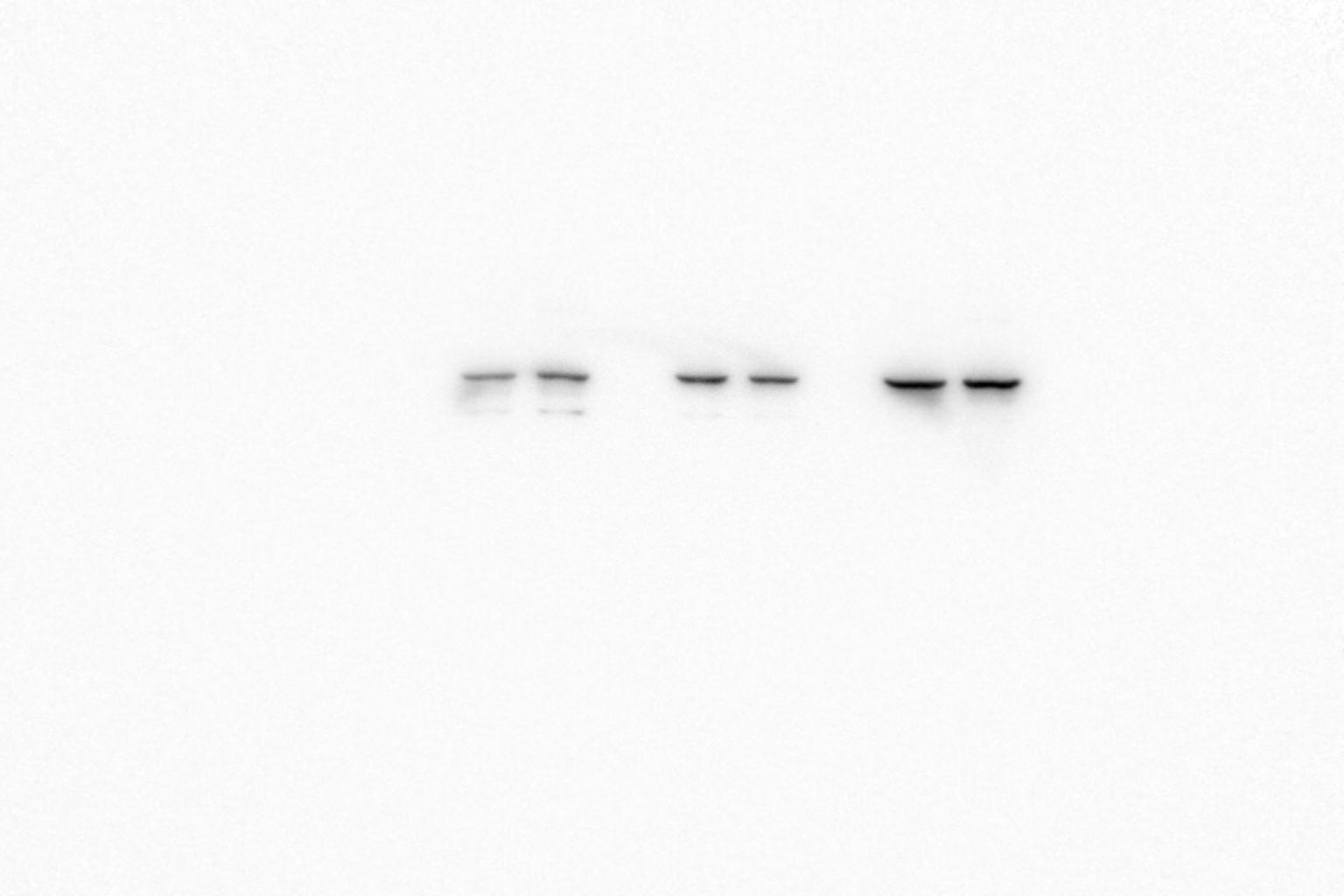

Supplement: Supplementary file 7 — Source data Fig. 5 [file 44318_2025_572_MOESM7_ESM.zip › Figure 5/Figure 5E/sim8 B ACTIN 2 SEC.gel]

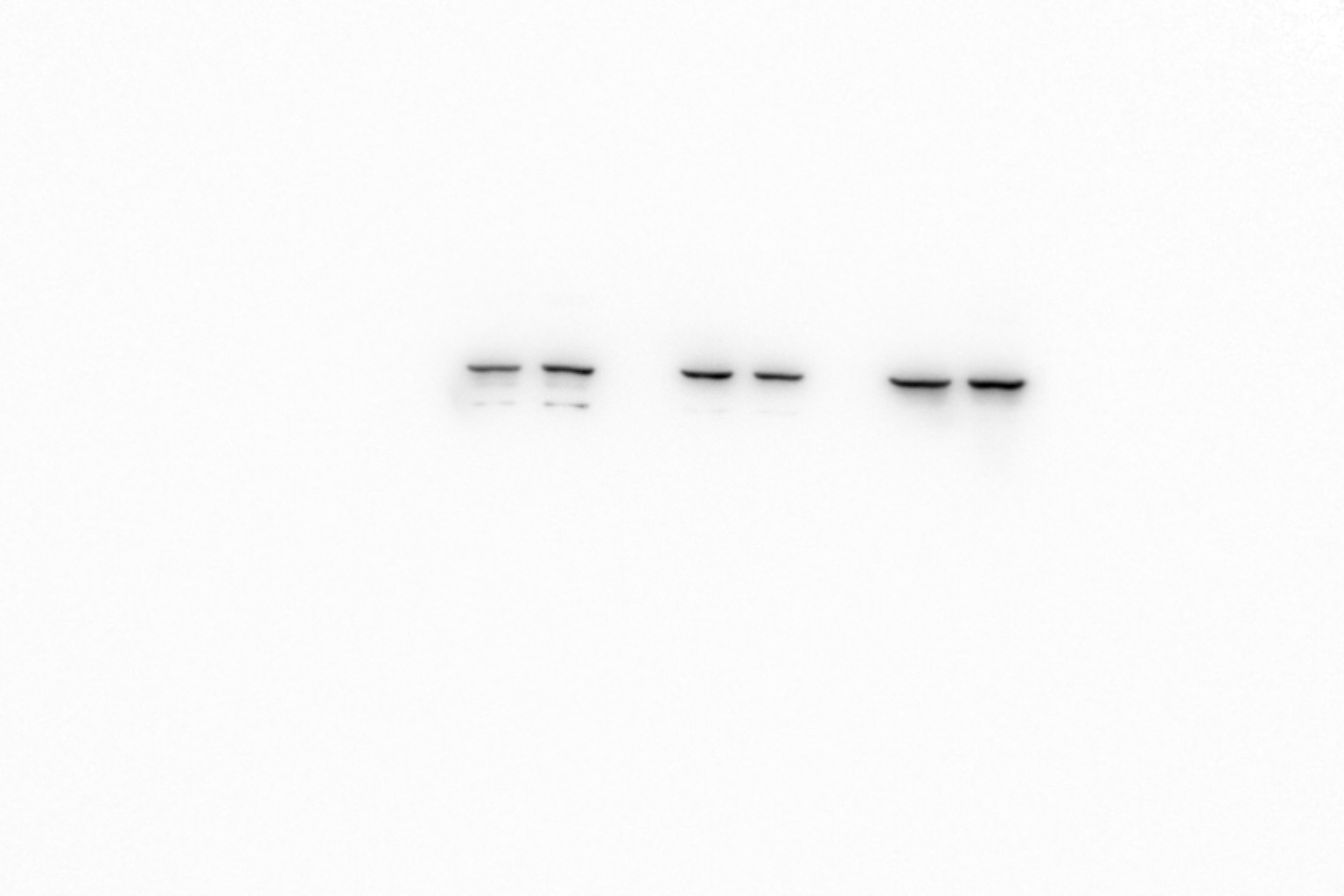

Supplement: Supplementary file 7 — Source data Fig. 5 [file 44318_2025_572_MOESM7_ESM.zip › Figure 5/Figure 5E/sim8 B ACTIN 4 SEC.gel]

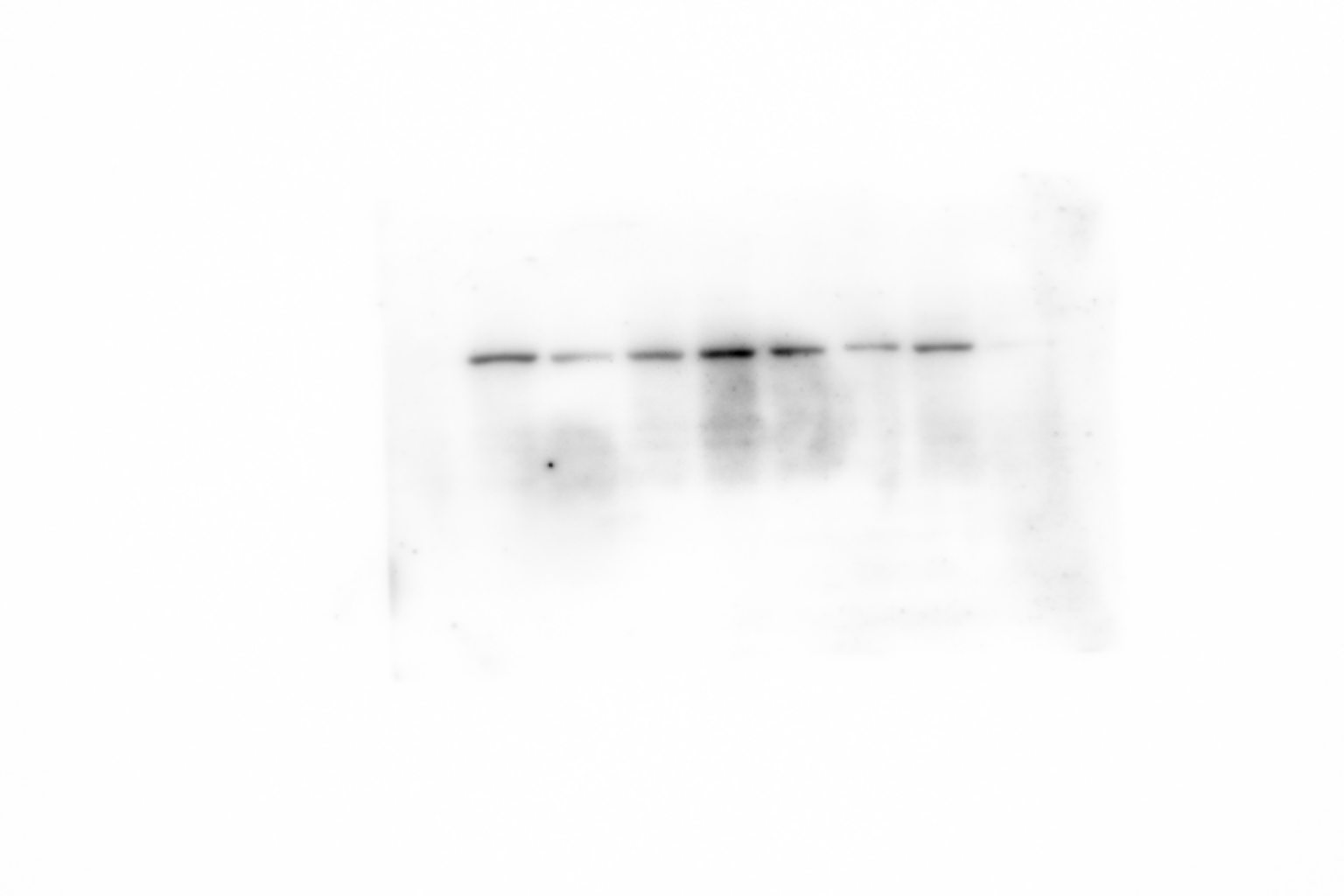

Supplement: Supplementary file 7 — Source data Fig. 5 [file 44318_2025_572_MOESM7_ESM.zip › Figure 5/Figure 5E/siM8 MARCH 8 240 SEC.gel]

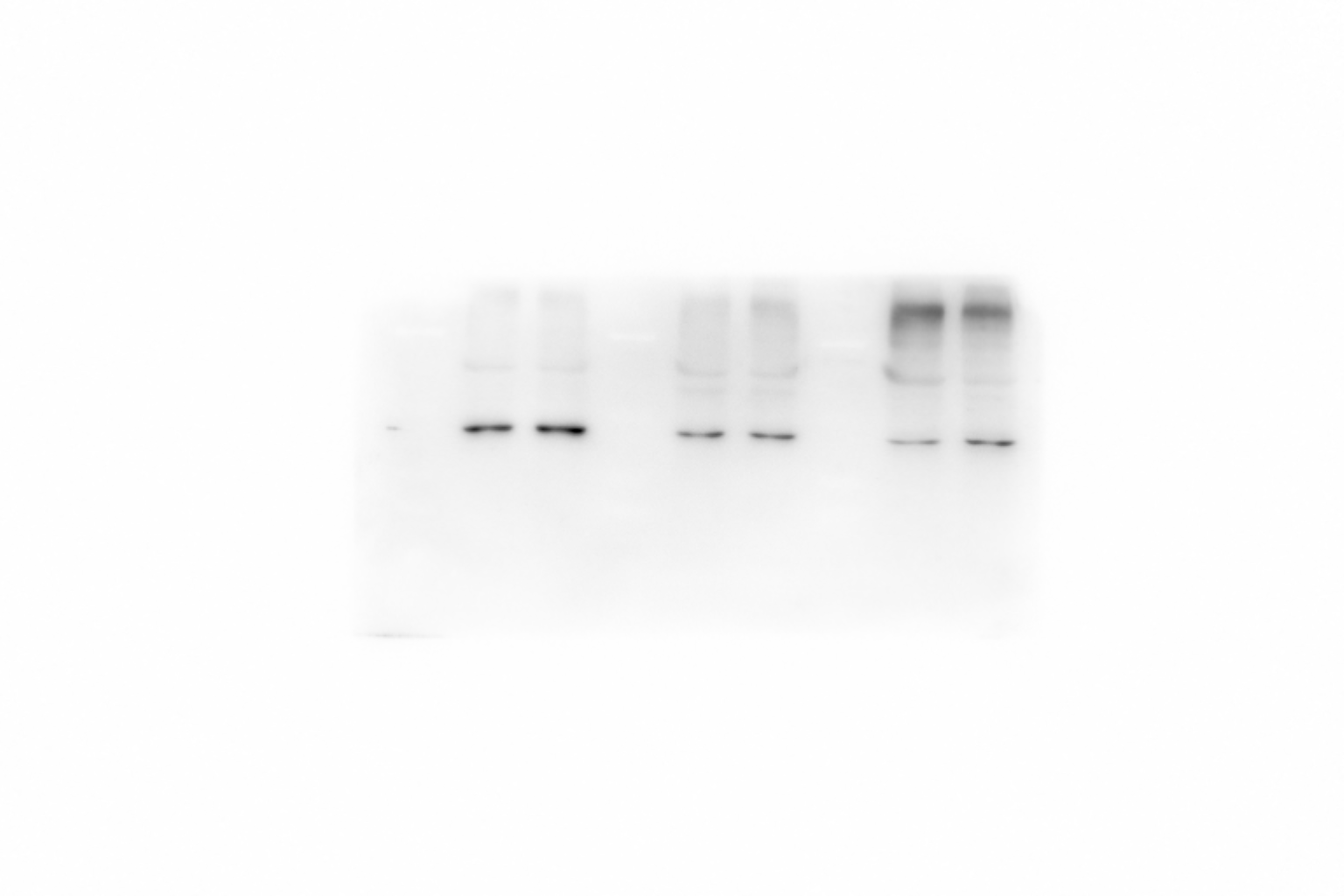

Supplement: Supplementary file 7 — Source data Fig. 5 [file 44318_2025_572_MOESM7_ESM.zip › Figure 5/Figure 5E/sim8 ORAI3 10 SEC.gel]

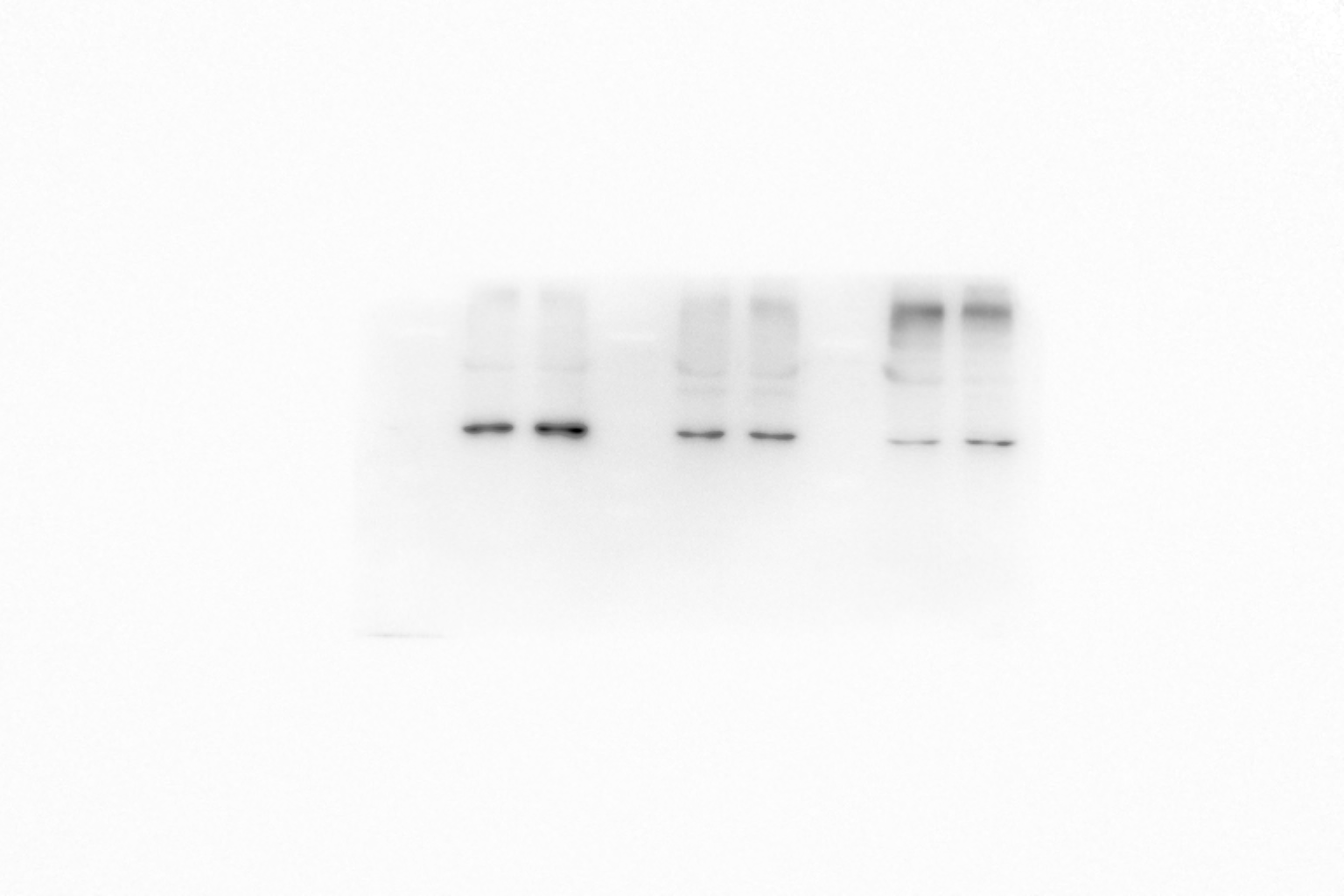

Supplement: Supplementary file 7 — Source data Fig. 5 [file 44318_2025_572_MOESM7_ESM.zip › Figure 5/Figure 5E/sim8 ORAI3 4 SEC.gel]

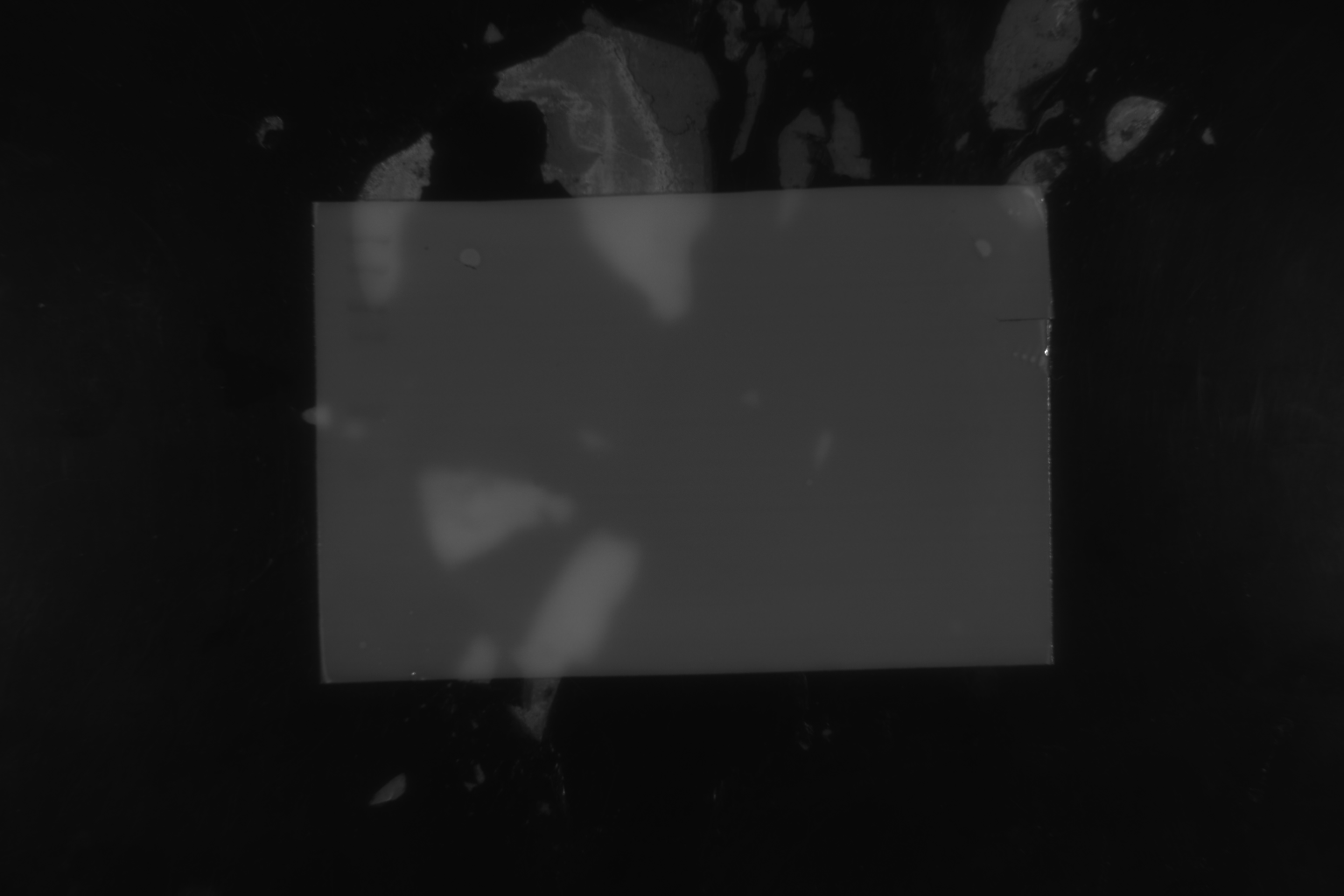

Supplement: Supplementary file 7 — Source data Fig. 5 [file 44318_2025_572_MOESM7_ESM.zip › Figure 5/Figure 5E/V_siM8 B ACTIN 0.5 SEC.gel]

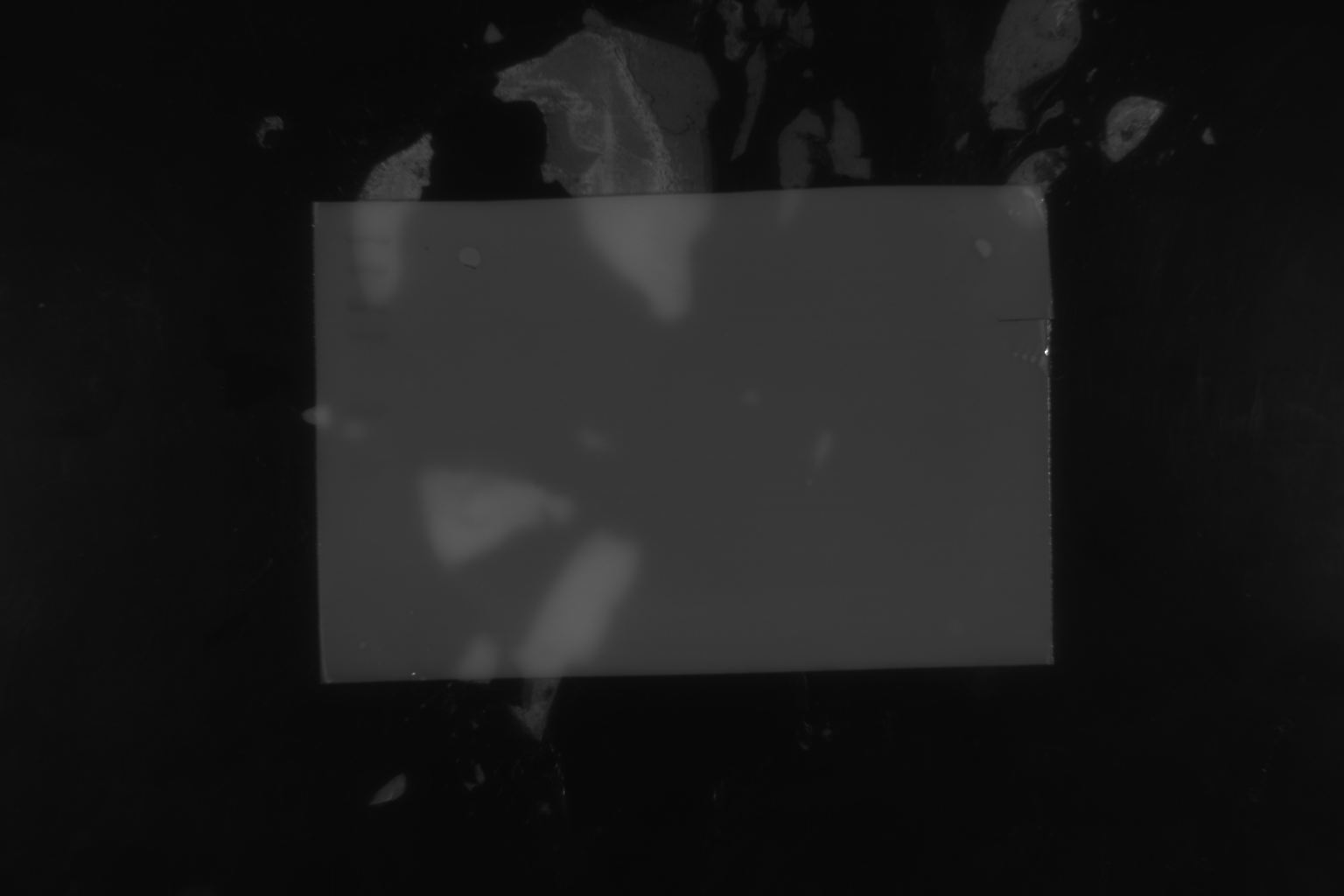

Supplement: Supplementary file 7 — Source data Fig. 5 [file 44318_2025_572_MOESM7_ESM.zip › Figure 5/Figure 5E/V_siM8 B ACTIN 1 SEC (2).gel]

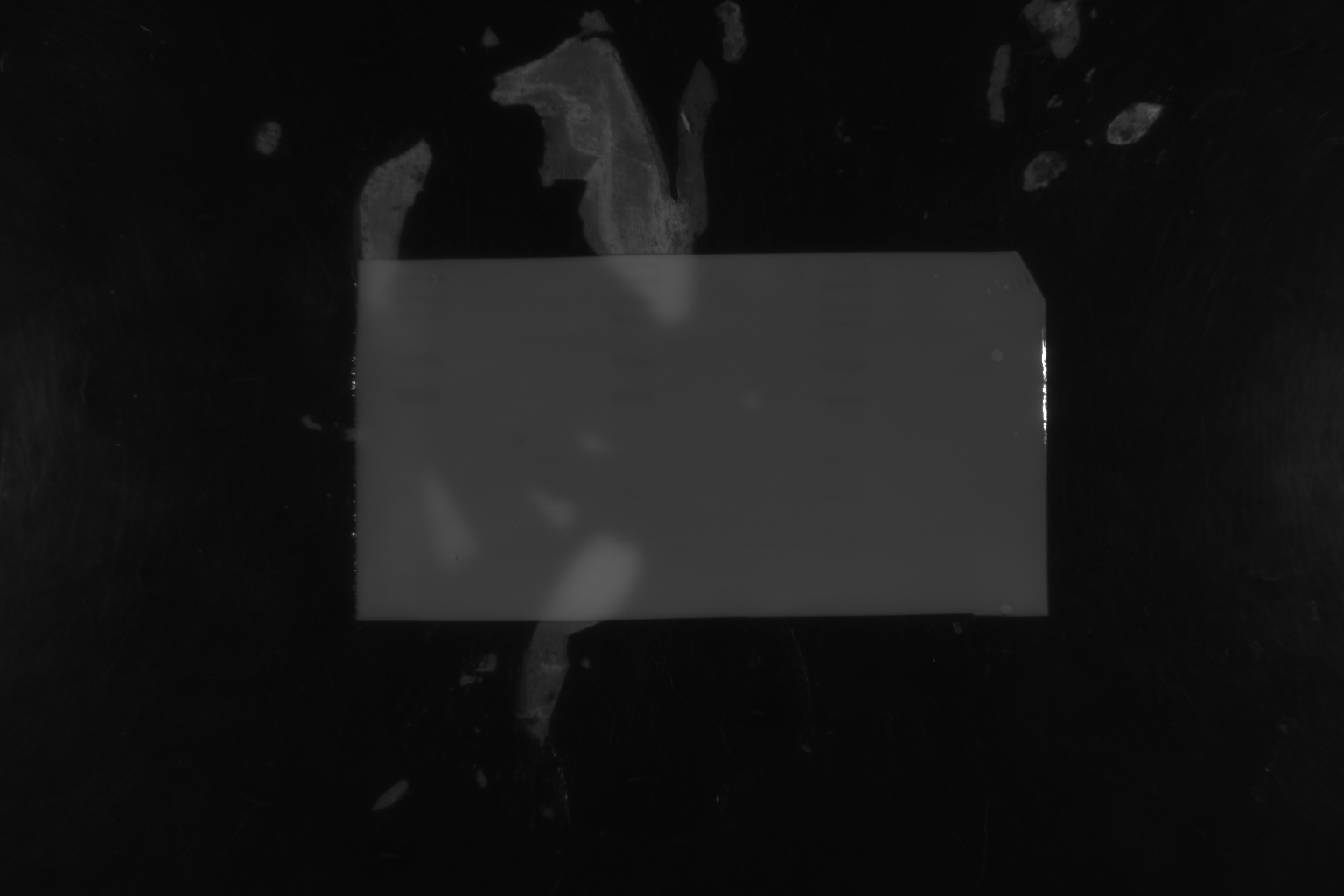

Supplement: Supplementary file 7 — Source data Fig. 5 [file 44318_2025_572_MOESM7_ESM.zip › Figure 5/Figure 5E/V_sim8 B ACTIN 1 SEC.gel]

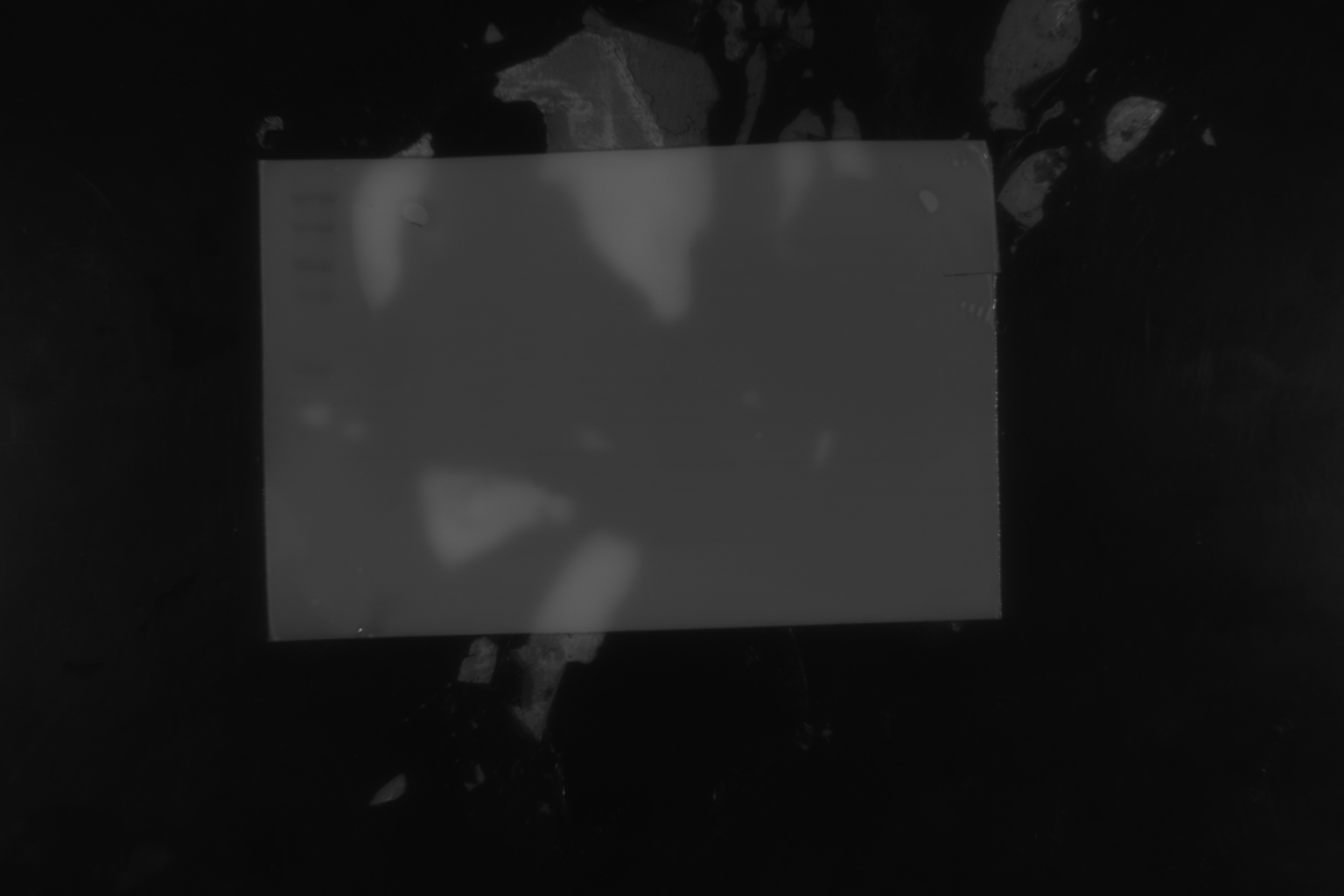

Supplement: Supplementary file 7 — Source data Fig. 5 [file 44318_2025_572_MOESM7_ESM.zip › Figure 5/Figure 5E/V_siM8 B ACTIN 2 SEC (2).gel]

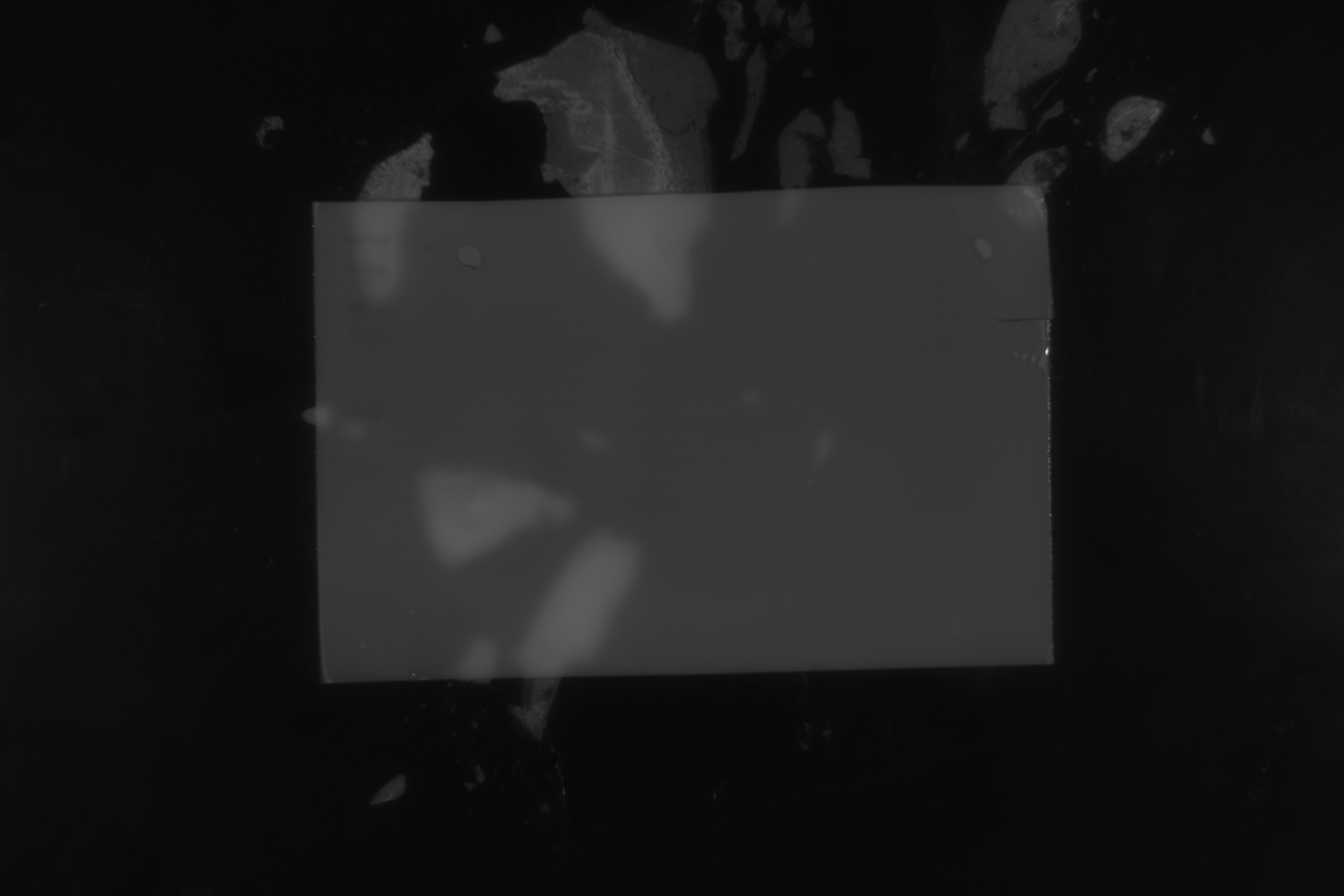

Supplement: Supplementary file 7 — Source data Fig. 5 [file 44318_2025_572_MOESM7_ESM.zip › Figure 5/Figure 5E/V_siM8 B ACTIN 2 SEC 2.gel]

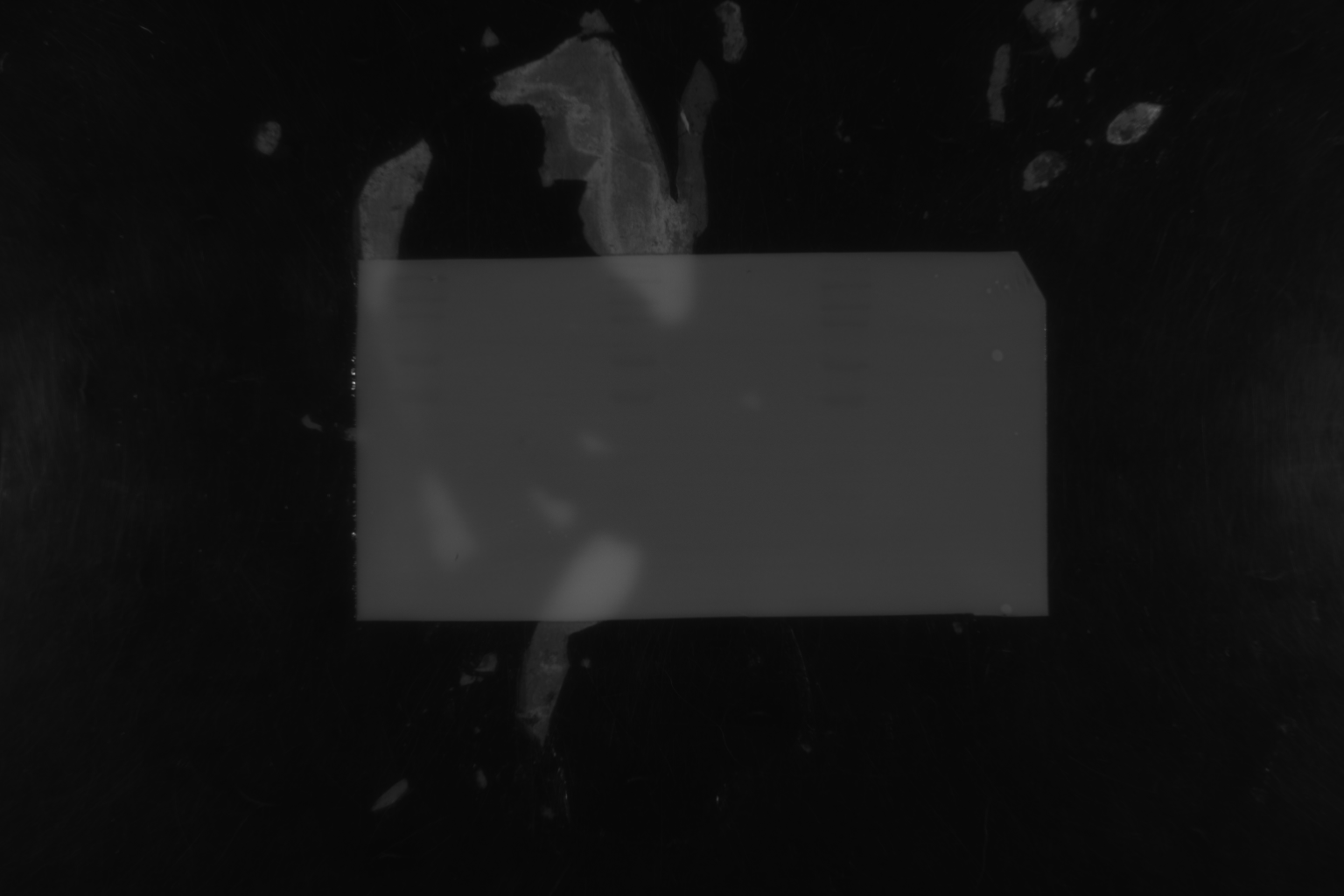

Supplement: Supplementary file 7 — Source data Fig. 5 [file 44318_2025_572_MOESM7_ESM.zip › Figure 5/Figure 5E/V_sim8 B ACTIN 2 SEC.gel]

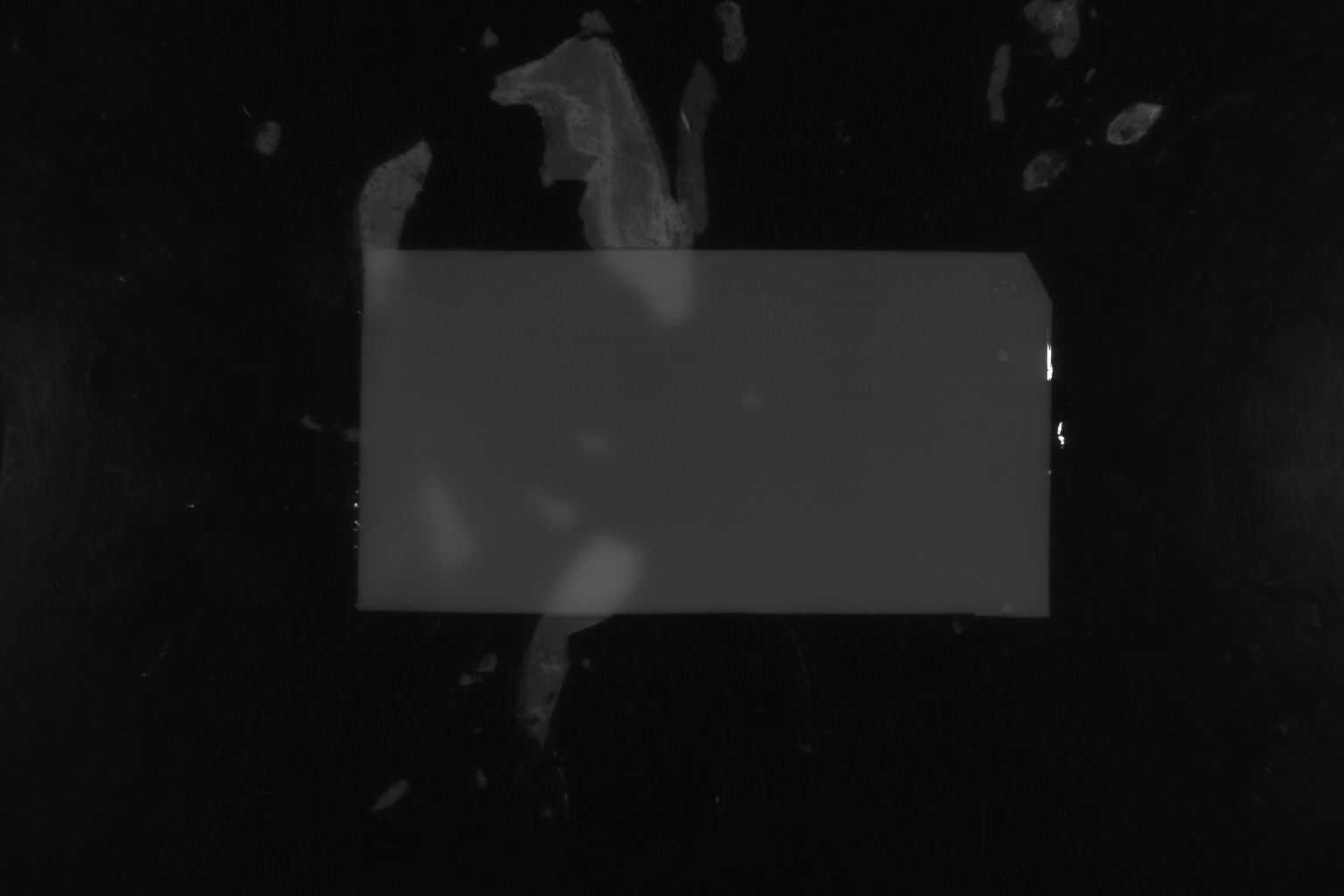

Supplement: Supplementary file 7 — Source data Fig. 5 [file 44318_2025_572_MOESM7_ESM.zip › Figure 5/Figure 5E/V_sim8 B ACTIN 4 SEC.gel]

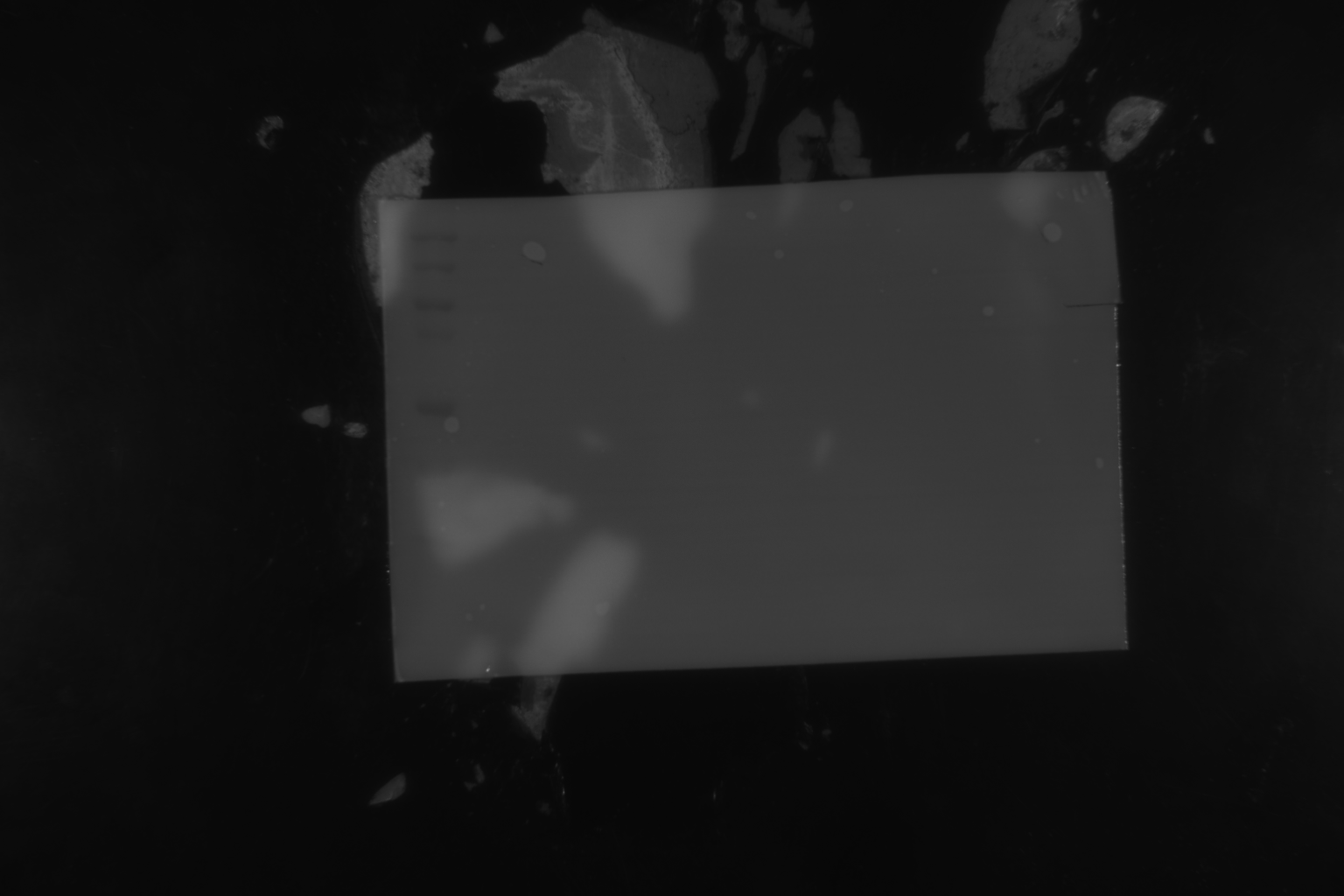

Supplement: Supplementary file 7 — Source data Fig. 5 [file 44318_2025_572_MOESM7_ESM.zip › Figure 5/Figure 5E/V_siM8 MARCH 8 240 SEC.gel]

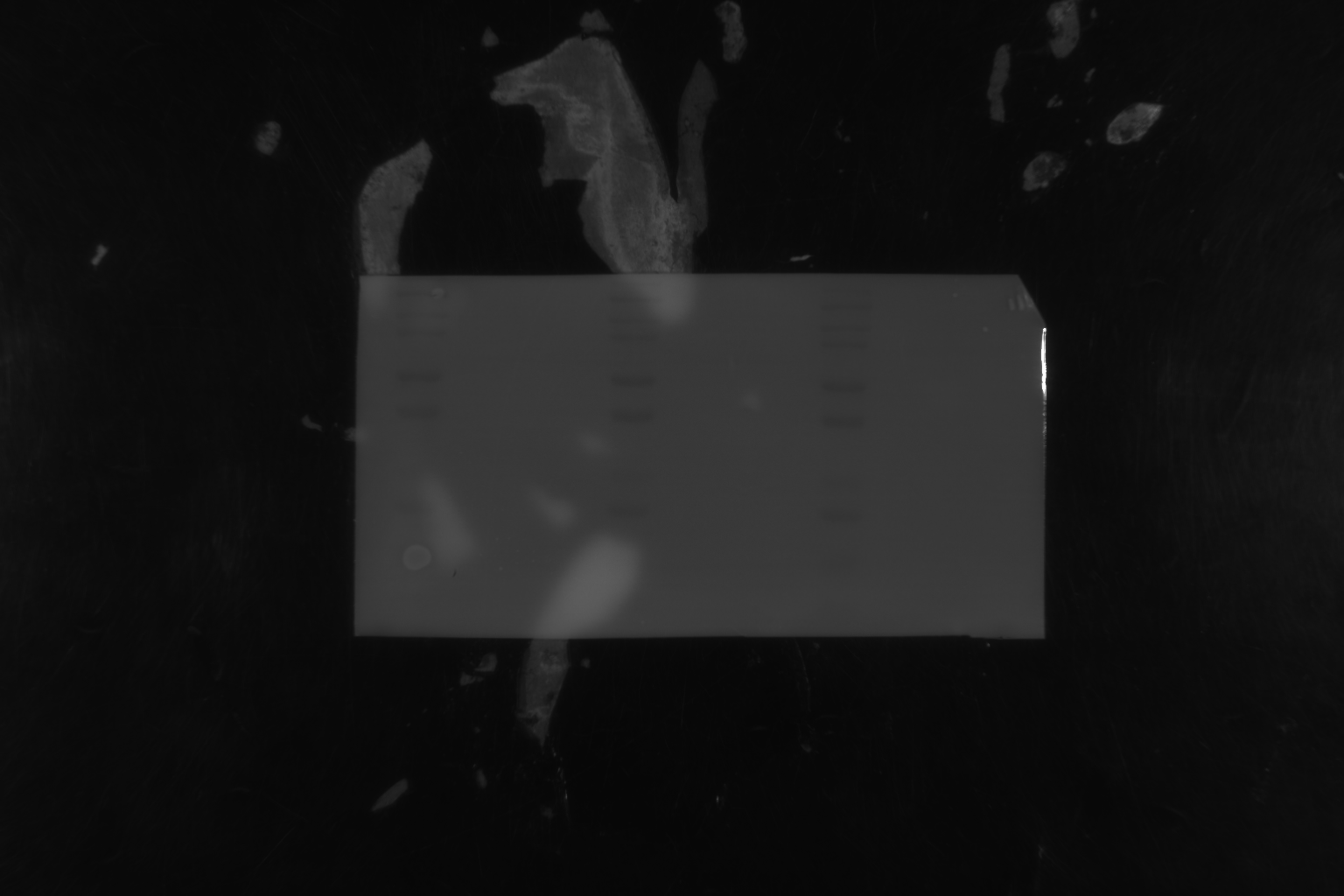

Supplement: Supplementary file 7 — Source data Fig. 5 [file 44318_2025_572_MOESM7_ESM.zip › Figure 5/Figure 5E/V_sim8 ORAI3 10 SEC.gel]

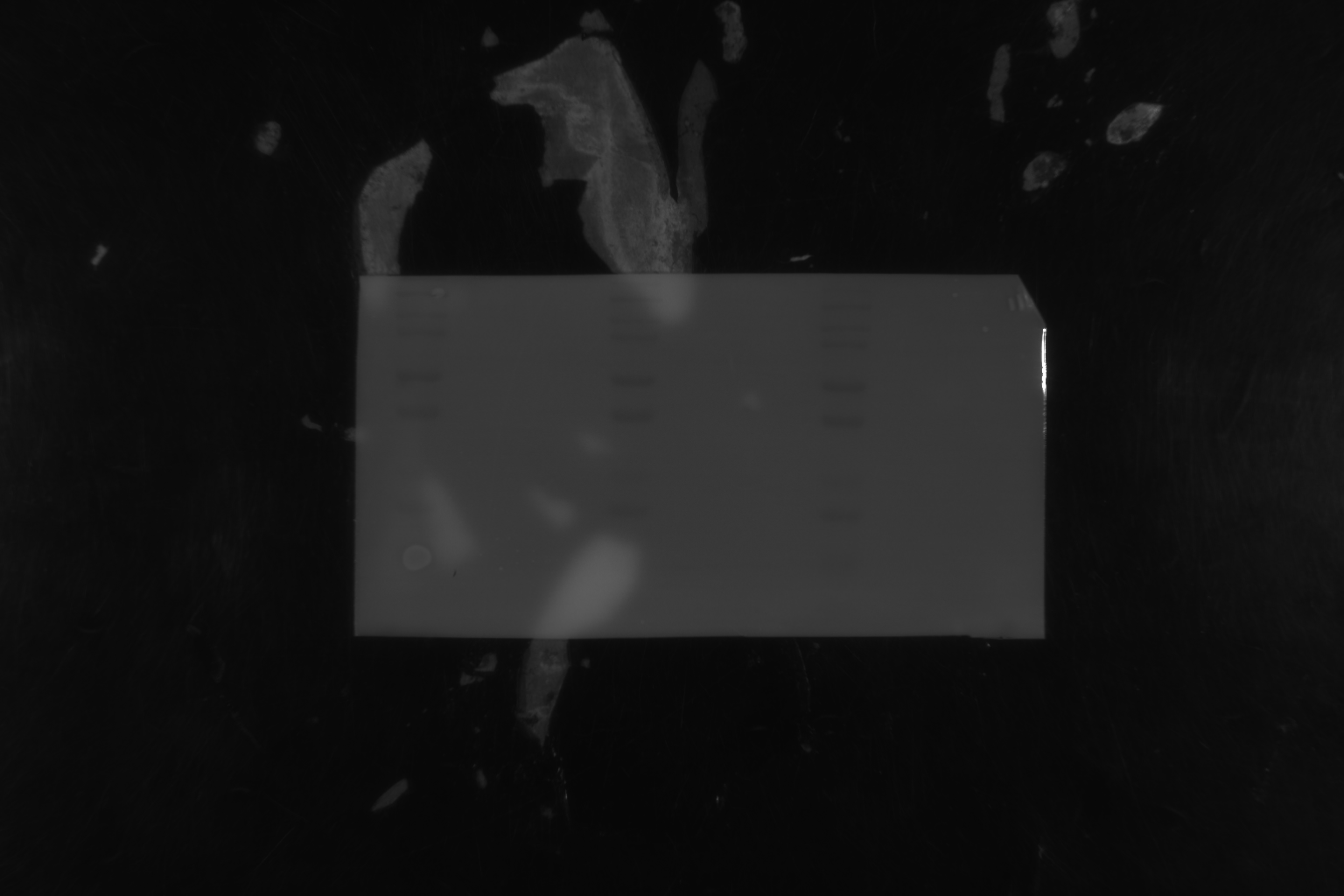

Supplement: Supplementary file 7 — Source data Fig. 5 [file 44318_2025_572_MOESM7_ESM.zip › Figure 5/Figure 5E/V_sim8 ORAI3 4 SEC.gel]

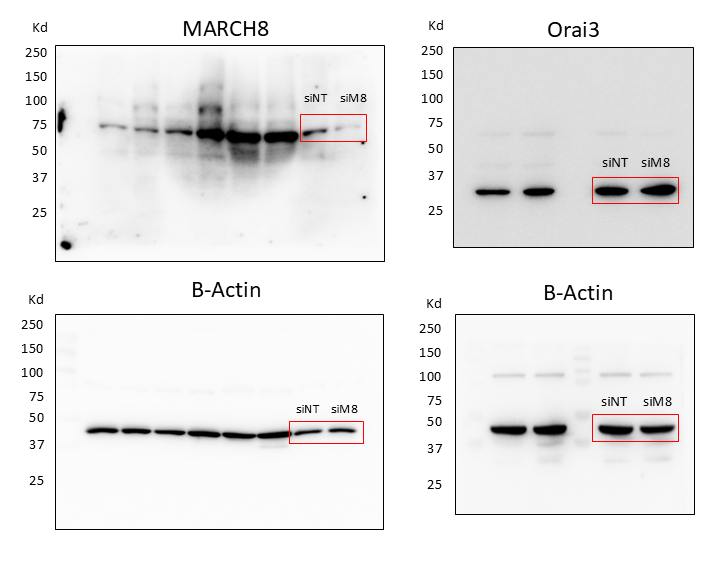

Supplement: Supplementary file 7 — Source data Fig. 5 [file 44318_2025_572_MOESM7_ESM.zip › Figure 5/Figure 5I/Figure 5I.png]

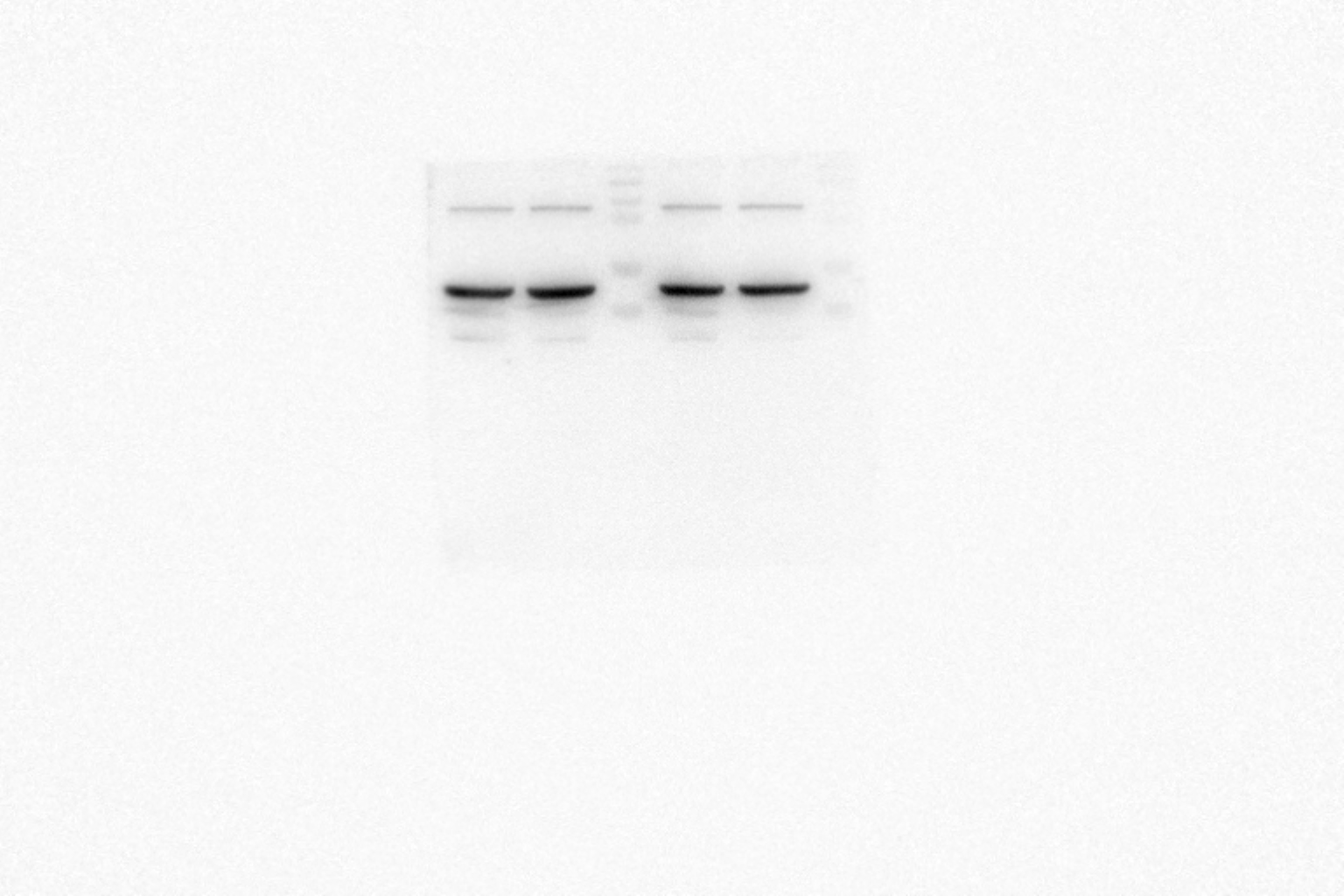

Supplement: Supplementary file 7 — Source data Fig. 5 [file 44318_2025_572_MOESM7_ESM.zip › Figure 5/Figure 5I/siM8 B ACTIN 0.125 SEC.gel]

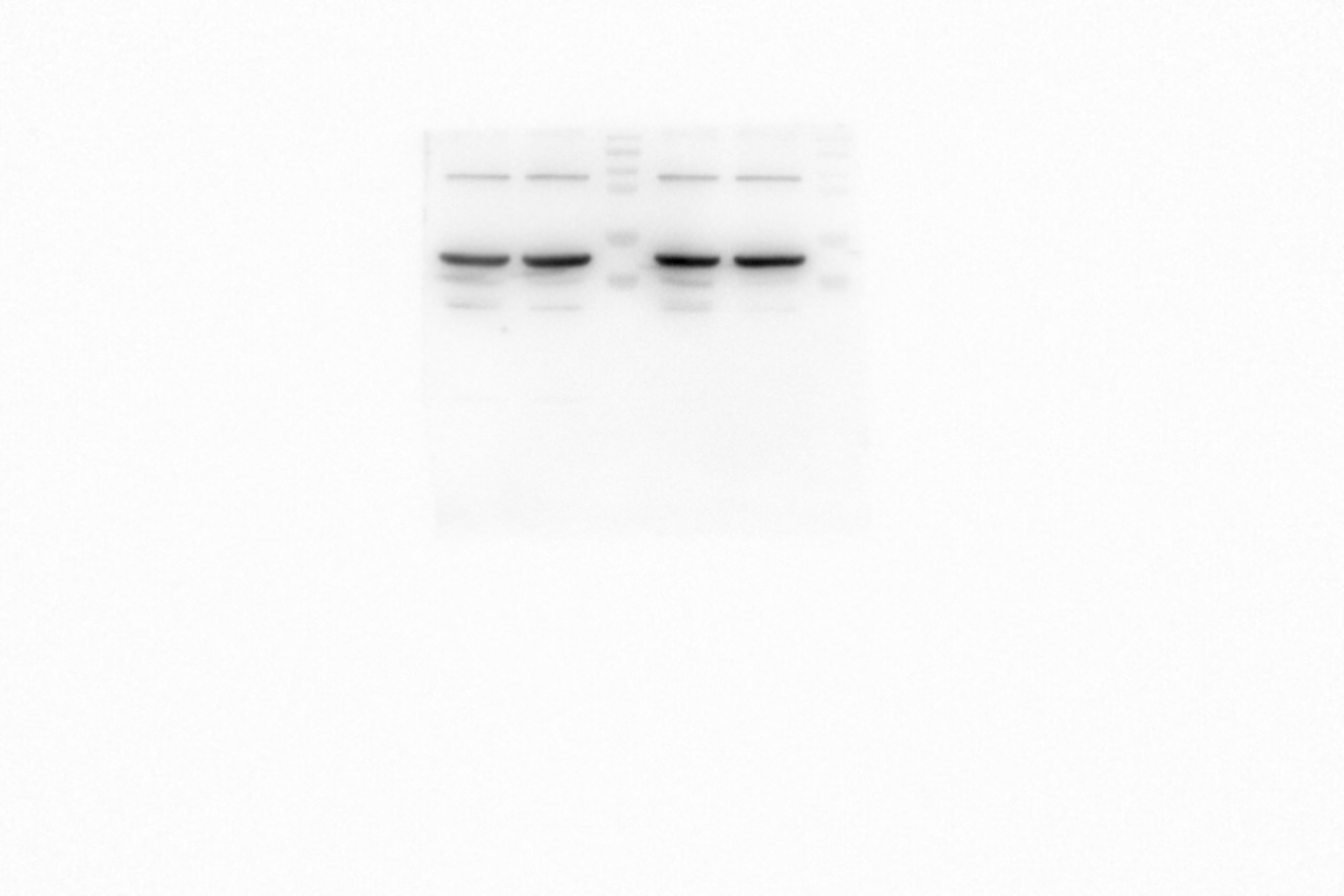

Supplement: Supplementary file 7 — Source data Fig. 5 [file 44318_2025_572_MOESM7_ESM.zip › Figure 5/Figure 5I/siM8 B ACTIN 0.25 SEC.gel]

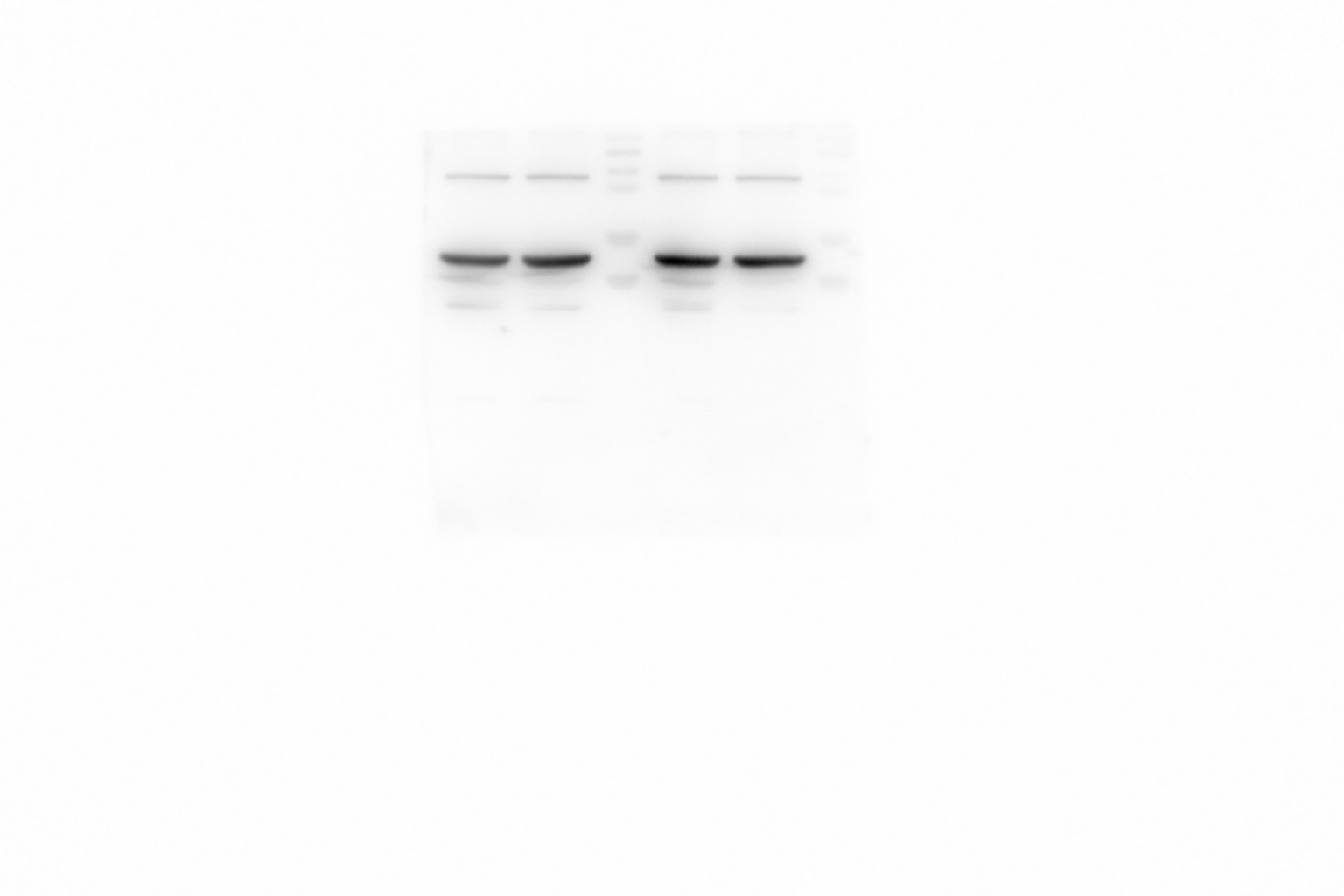

Supplement: Supplementary file 7 — Source data Fig. 5 [file 44318_2025_572_MOESM7_ESM.zip › Figure 5/Figure 5I/siM8 B ACTIN 0.5 SEC.gel]

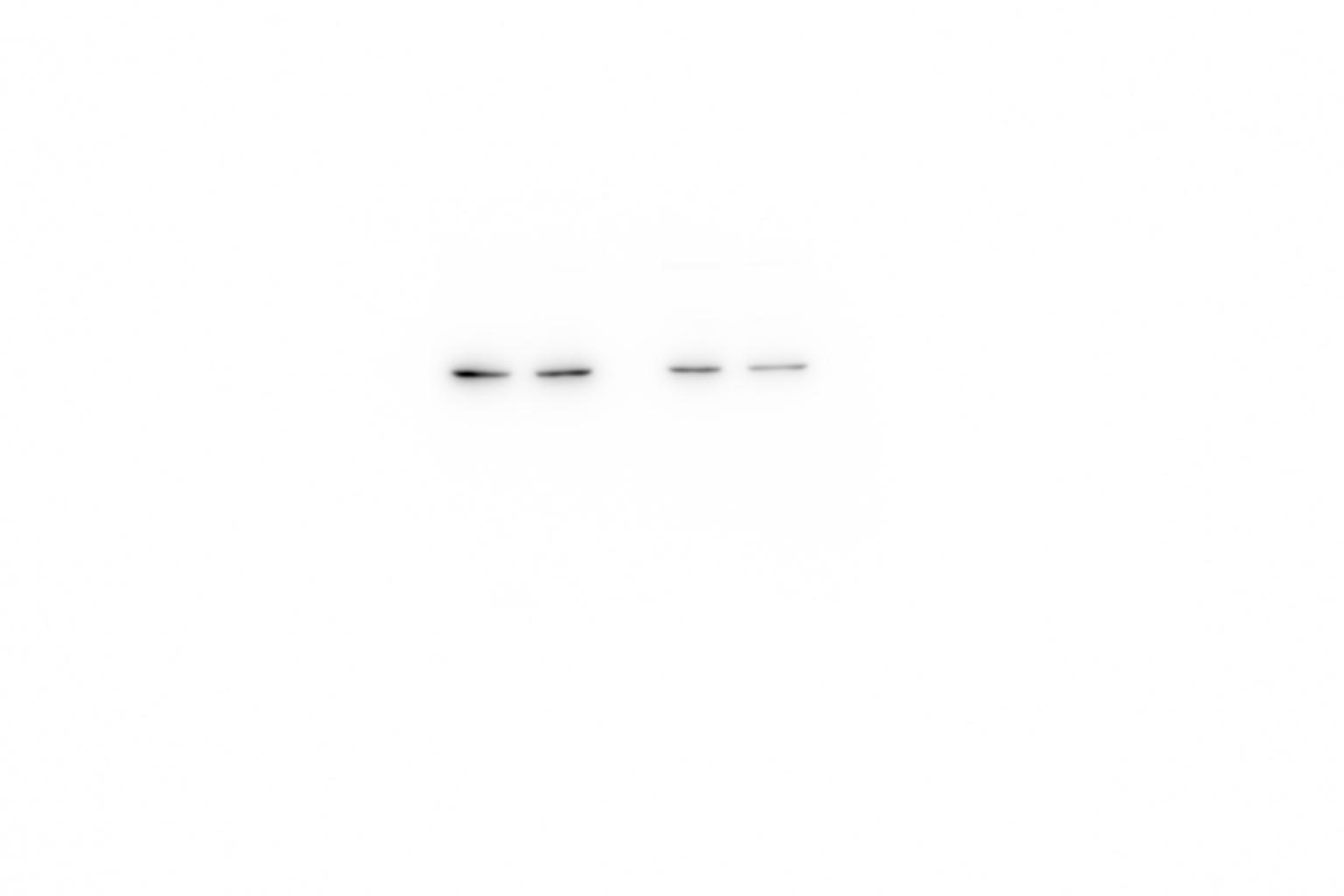

Supplement: Supplementary file 7 — Source data Fig. 5 [file 44318_2025_572_MOESM7_ESM.zip › Figure 5/Figure 5I/siM8 ORAI 3 15 SEC.gel]

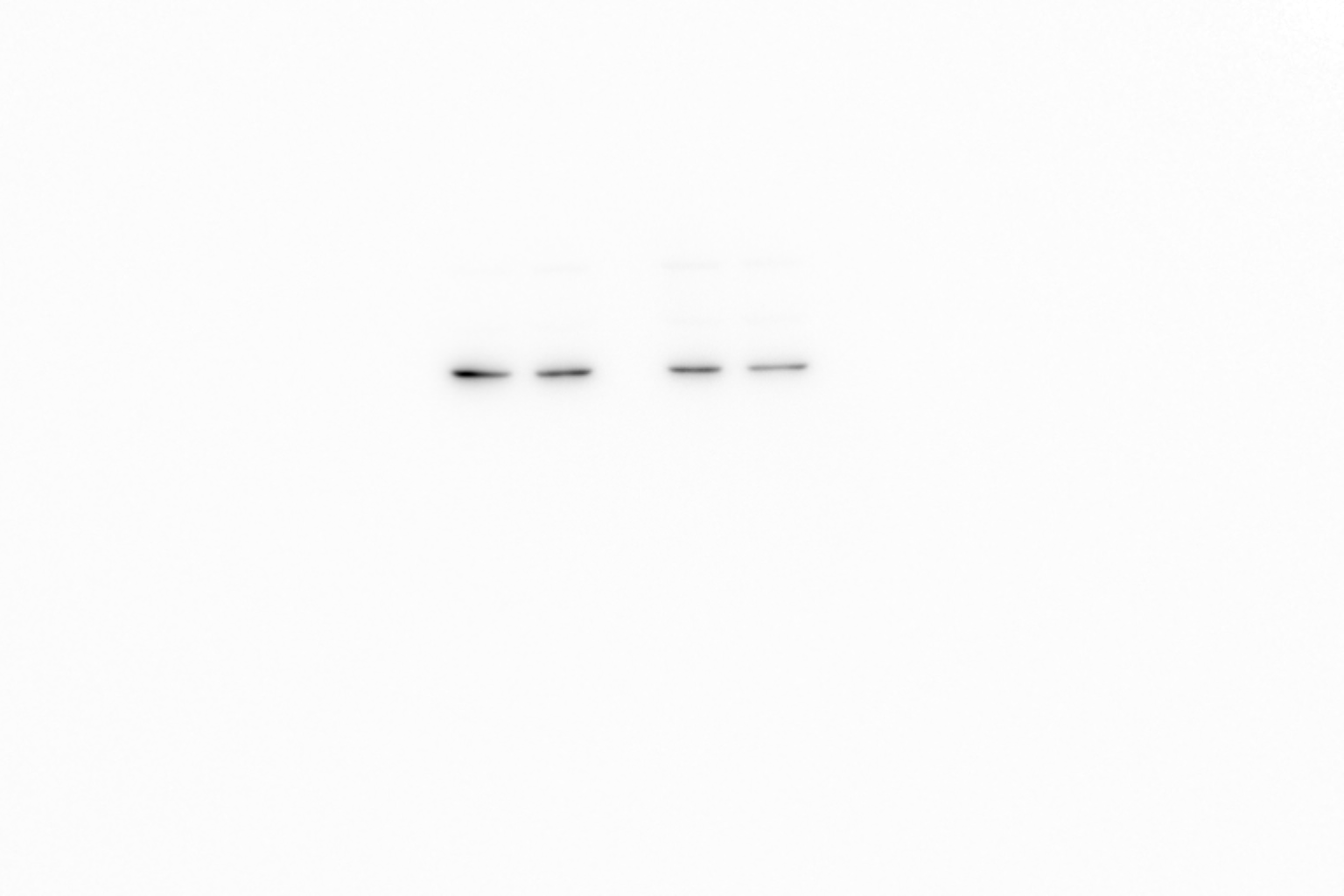

Supplement: Supplementary file 7 — Source data Fig. 5 [file 44318_2025_572_MOESM7_ESM.zip › Figure 5/Figure 5I/siM8 ORAI 3 8 SEC.gel]

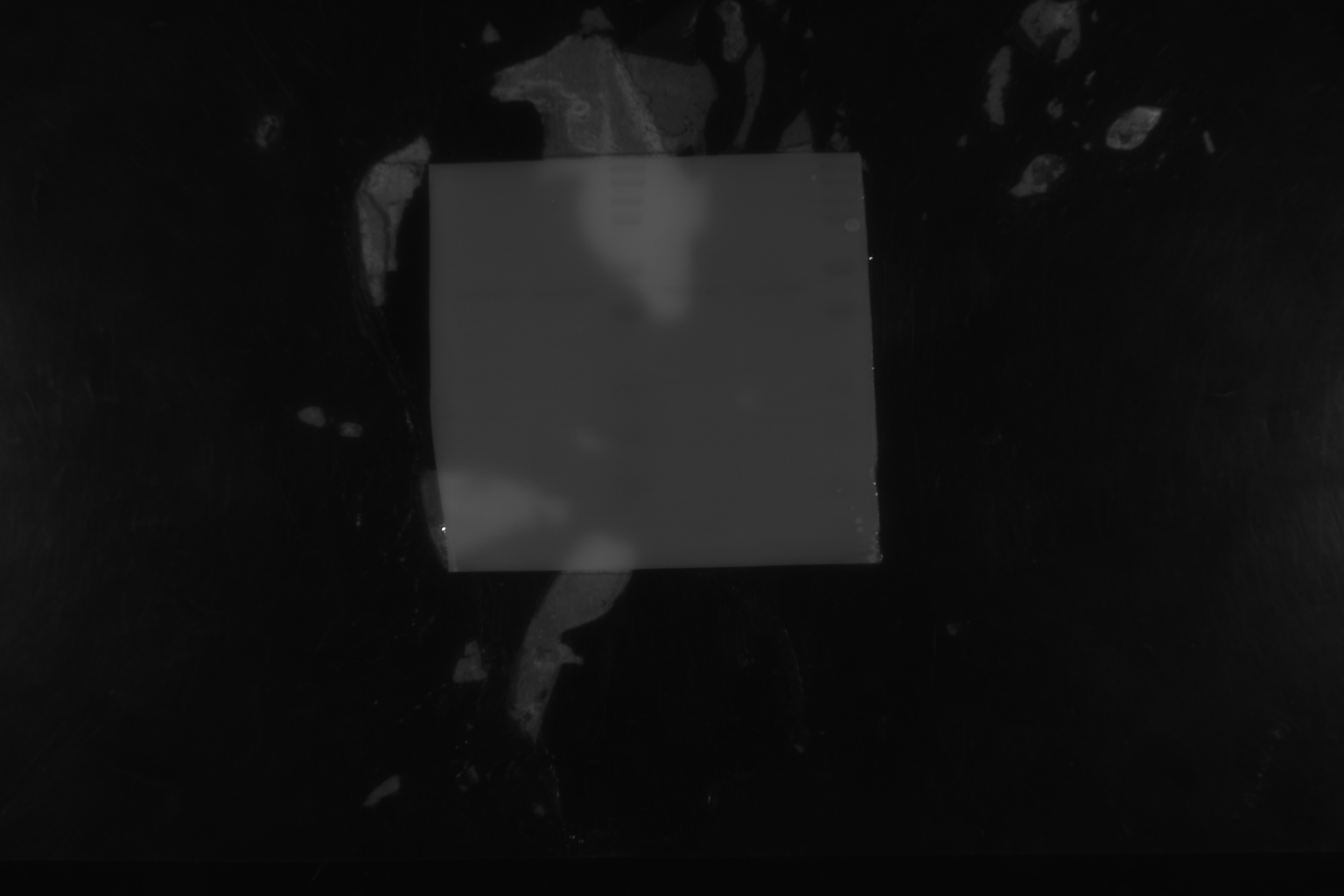

Supplement: Supplementary file 7 — Source data Fig. 5 [file 44318_2025_572_MOESM7_ESM.zip › Figure 5/Figure 5I/V_siM8 B ACTIN 0.125 SEC.gel]

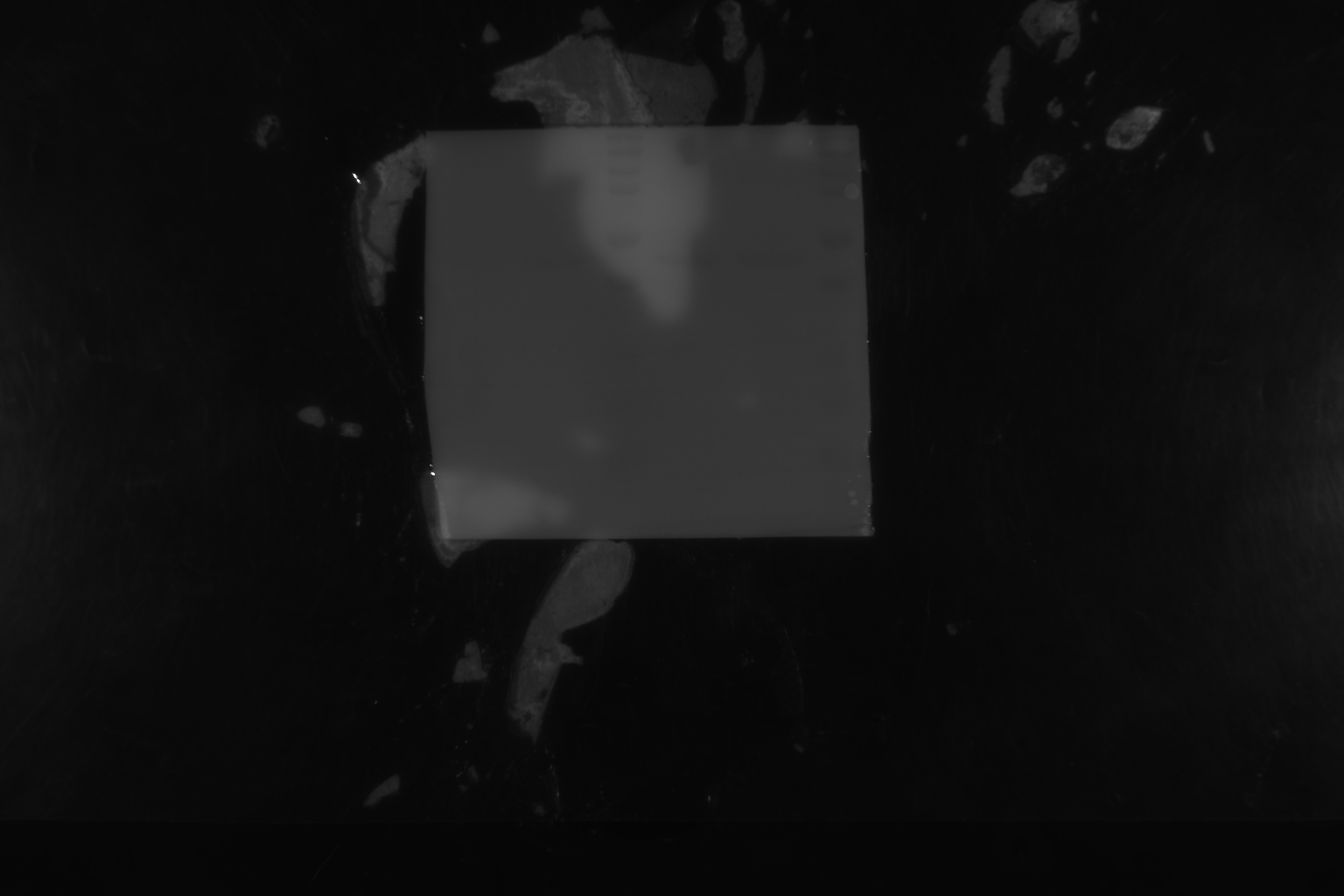

Supplement: Supplementary file 7 — Source data Fig. 5 [file 44318_2025_572_MOESM7_ESM.zip › Figure 5/Figure 5I/V_siM8 B ACTIN 0.25 SEC.gel]

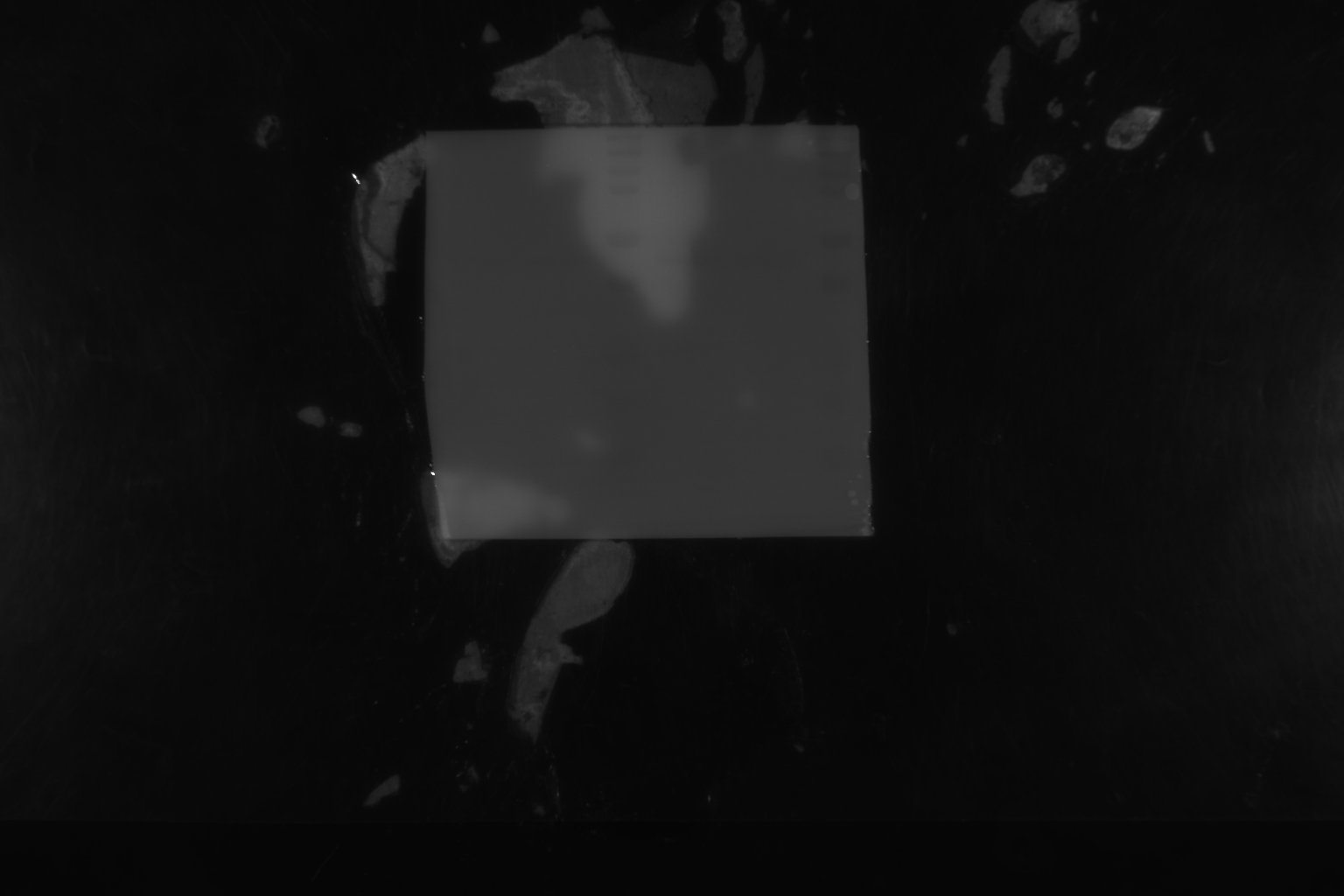

Supplement: Supplementary file 7 — Source data Fig. 5 [file 44318_2025_572_MOESM7_ESM.zip › Figure 5/Figure 5I/V_siM8 B ACTIN 0.5 SEC.gel]

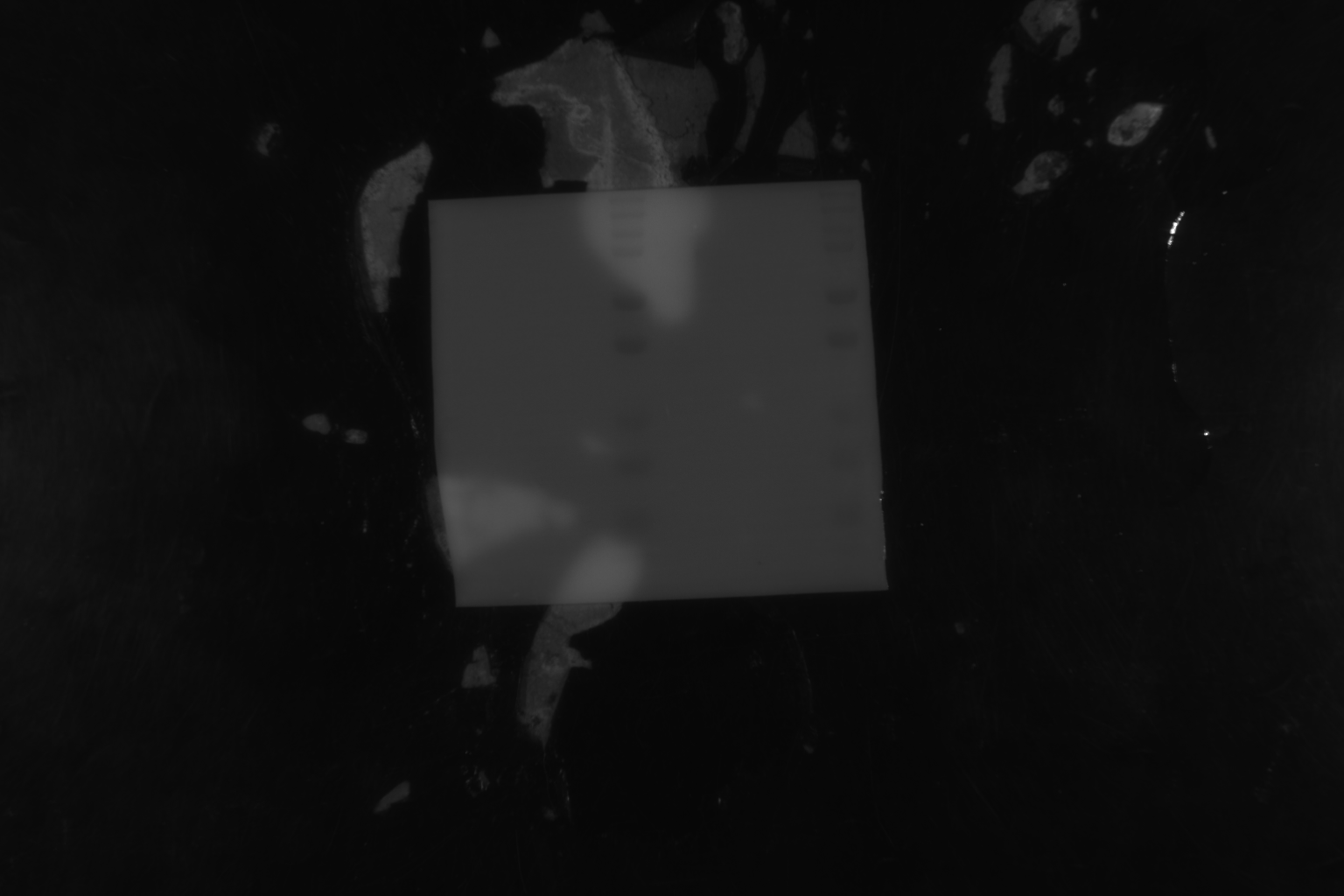

Supplement: Supplementary file 7 — Source data Fig. 5 [file 44318_2025_572_MOESM7_ESM.zip › Figure 5/Figure 5I/V_siM8 ORAI 3 15 SEC.gel]

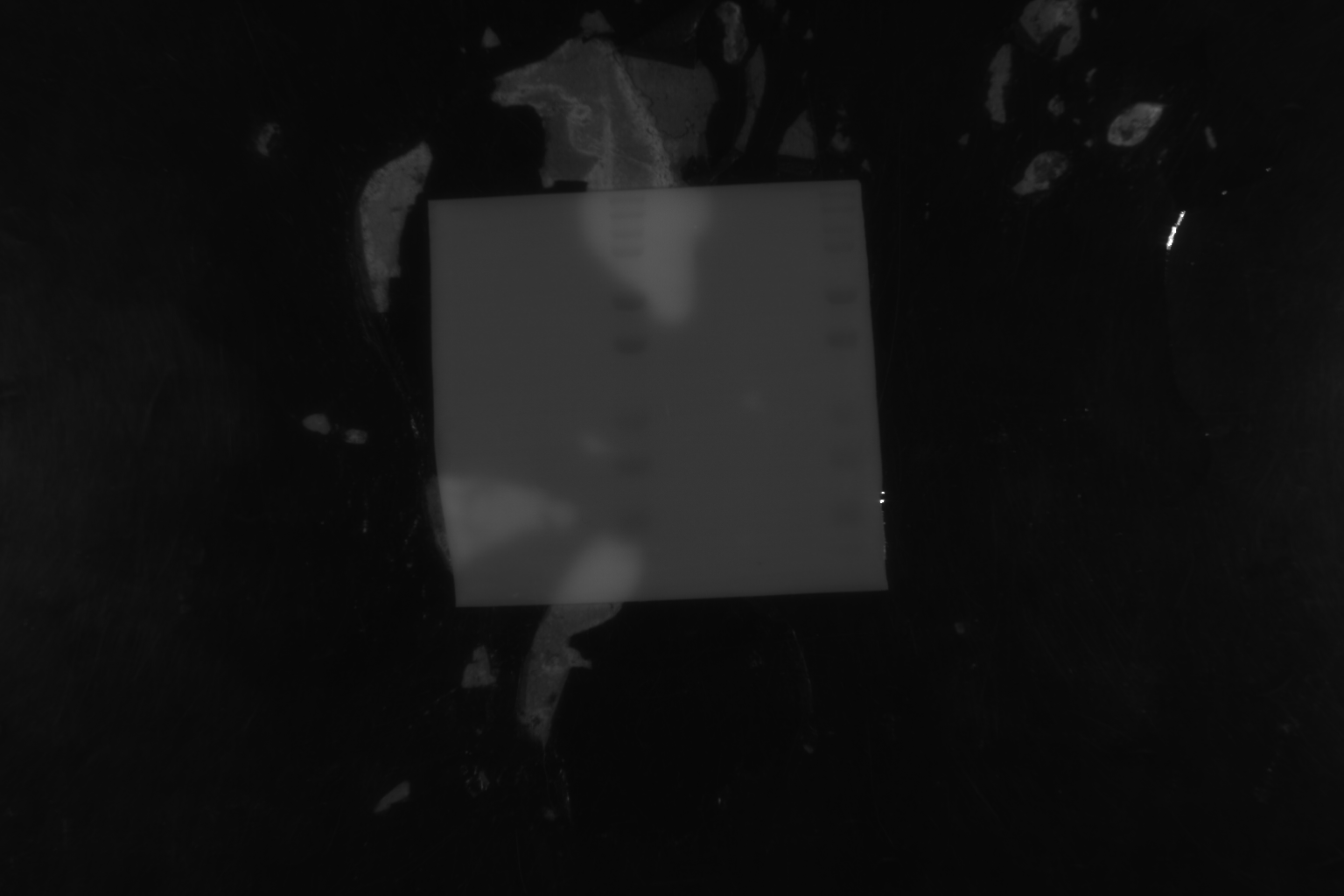

Supplement: Supplementary file 7 — Source data Fig. 5 [file 44318_2025_572_MOESM7_ESM.zip › Figure 5/Figure 5I/V_siM8 ORAI 3 8 SEC.gel]

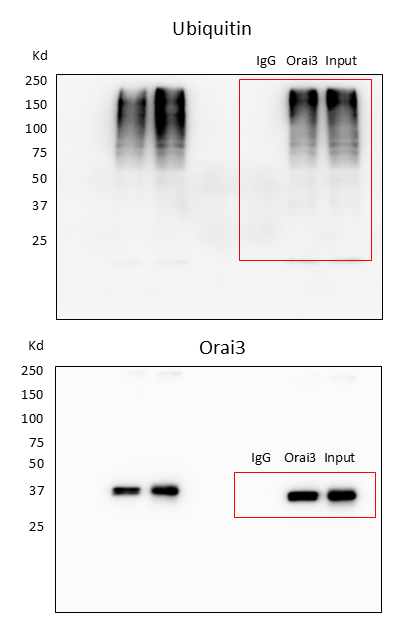

Supplement: Supplementary file 8 — Source data Fig. 6 [file 44318_2025_572_MOESM8_ESM.zip › EMBOJ-2025-121095R_Source Data for Figure 6/Figure 6/Figure 6A/Figure 6A.png]

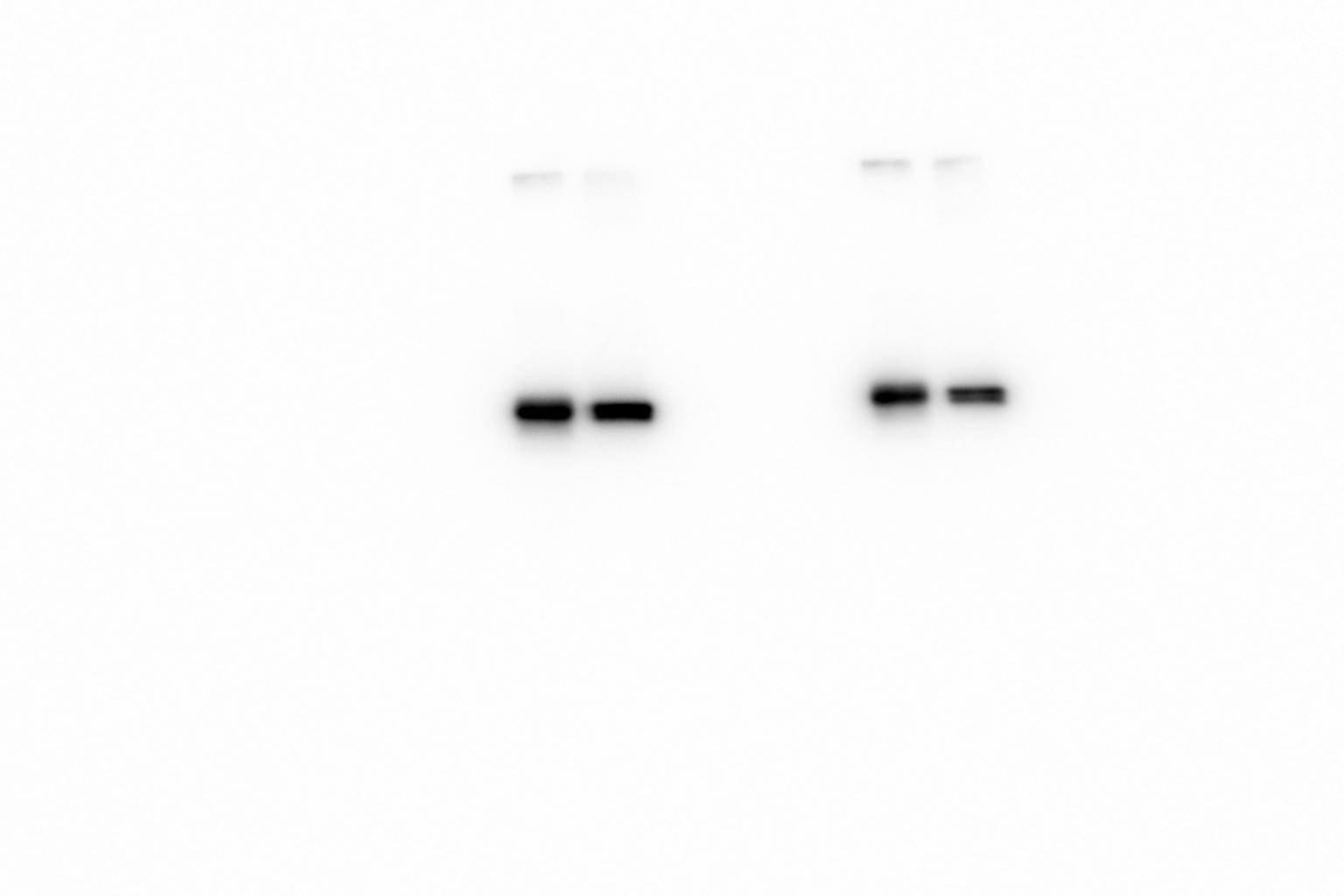

Supplement: Supplementary file 8 — Source data Fig. 6 [file 44318_2025_572_MOESM8_ESM.zip › EMBOJ-2025-121095R_Source Data for Figure 6/Figure 6/Figure 6A/IP ORAI 3.gel]

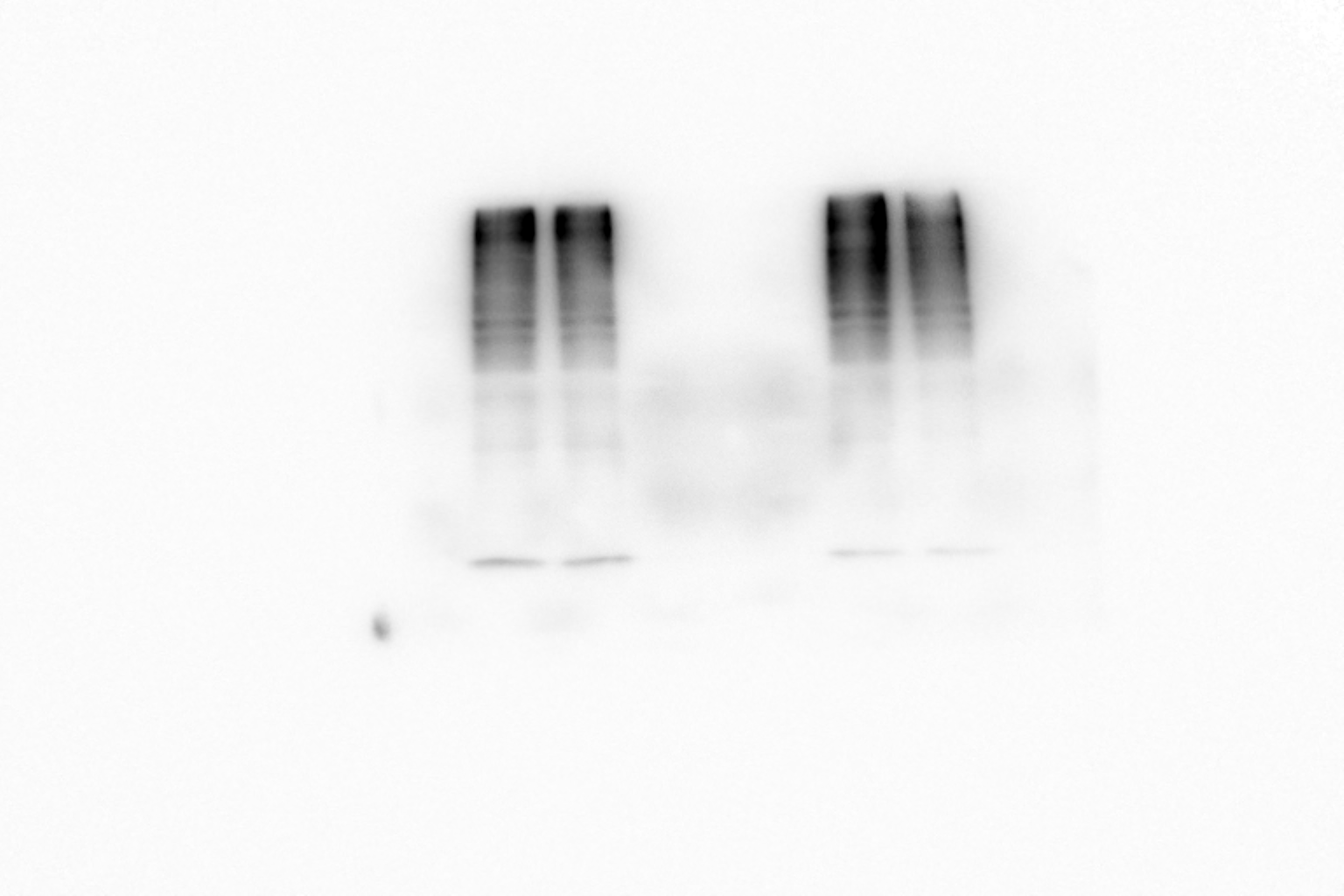

Supplement: Supplementary file 8 — Source data Fig. 6 [file 44318_2025_572_MOESM8_ESM.zip › EMBOJ-2025-121095R_Source Data for Figure 6/Figure 6/Figure 6A/IP UBIQUITIN 4 SEC.gel]

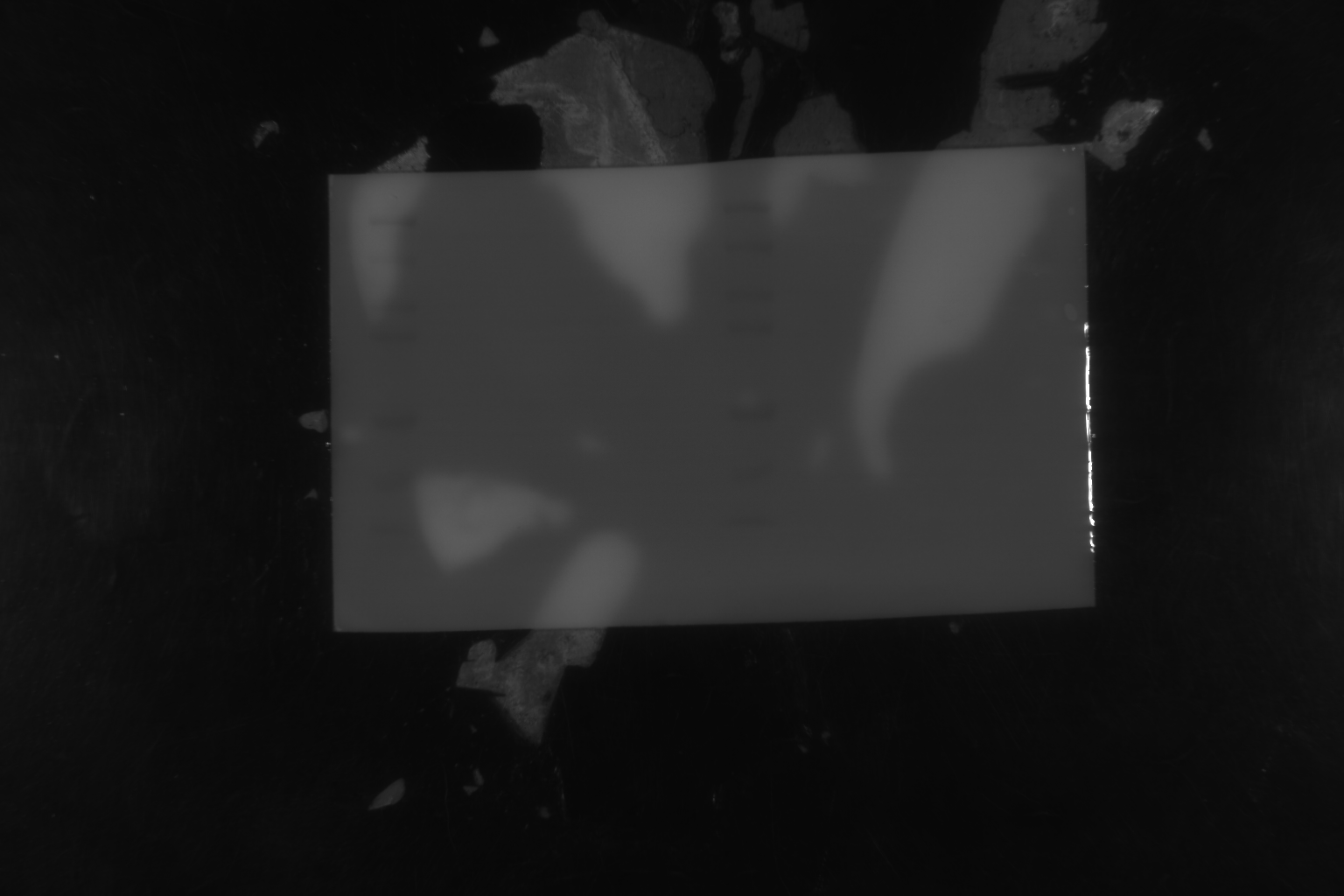

Supplement: Supplementary file 8 — Source data Fig. 6 [file 44318_2025_572_MOESM8_ESM.zip › EMBOJ-2025-121095R_Source Data for Figure 6/Figure 6/Figure 6A/V_IP ORAI 3.gel]

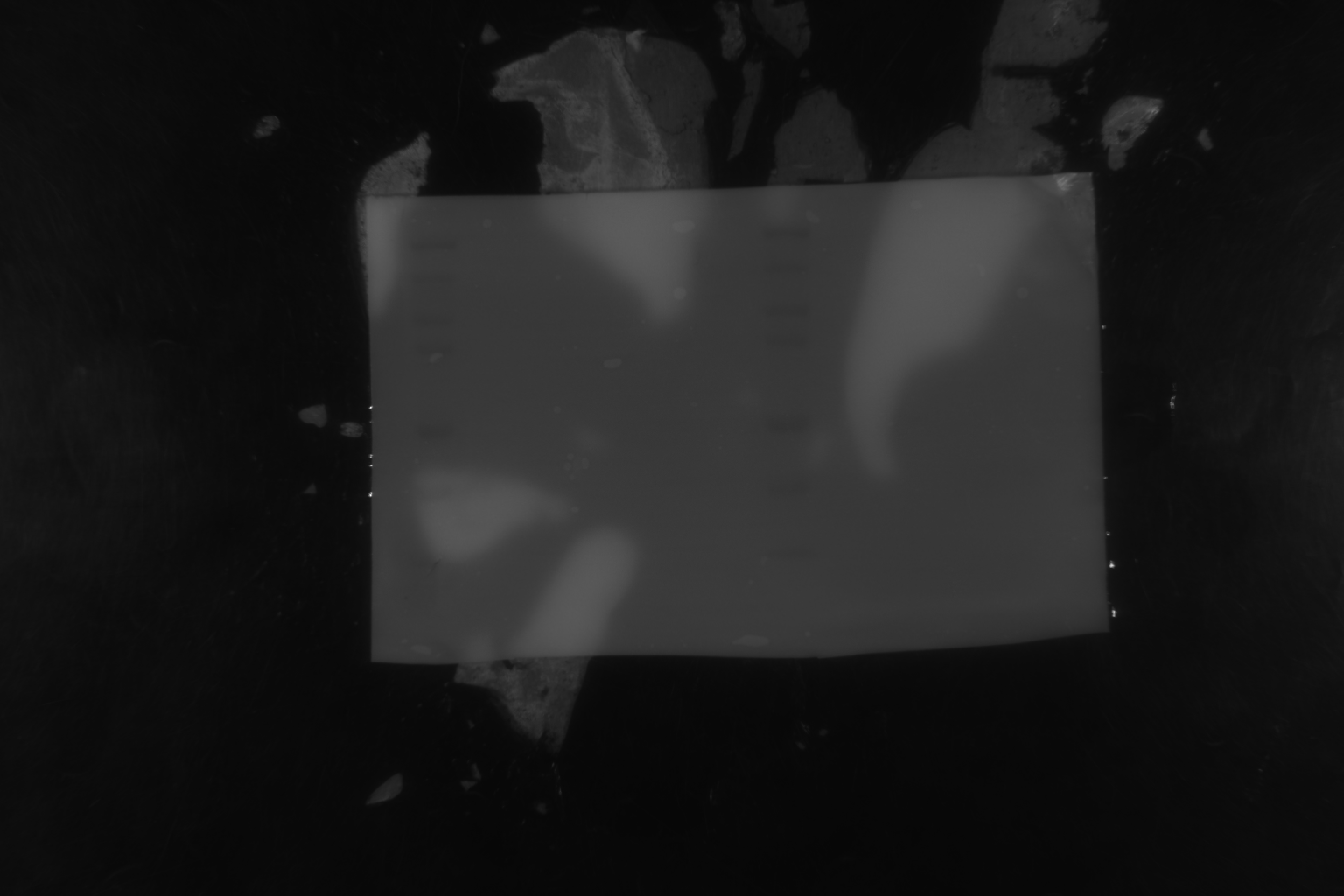

Supplement: Supplementary file 8 — Source data Fig. 6 [file 44318_2025_572_MOESM8_ESM.zip › EMBOJ-2025-121095R_Source Data for Figure 6/Figure 6/Figure 6A/V_IP UBIQUITIN 4 SEC.gel]

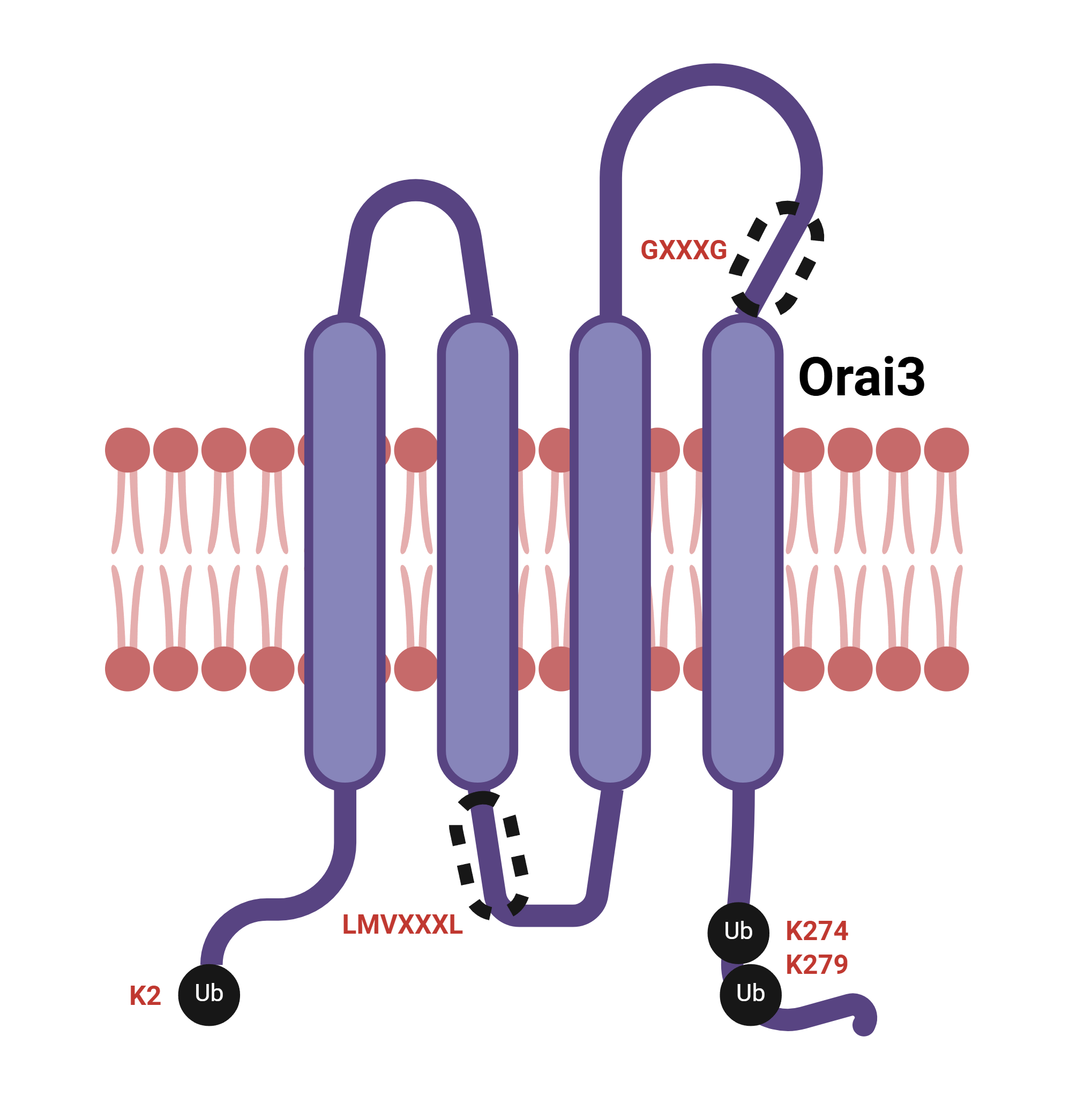

Supplement: Supplementary file 8 — Source data Fig. 6 [file 44318_2025_572_MOESM8_ESM.zip › EMBOJ-2025-121095R_Source Data for Figure 6/Figure 6/Figure 6B/Orai3 Ubiquitination and March8 Interaction.png]

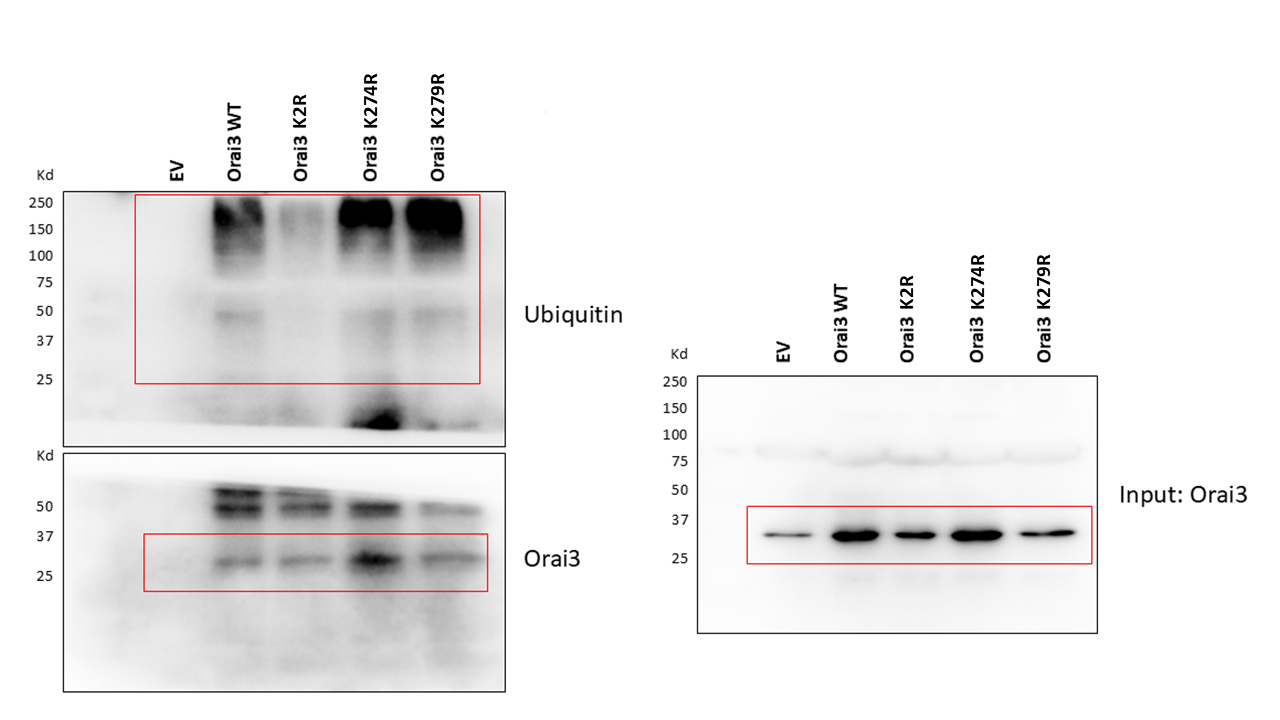

Supplement: Supplementary file 8 — Source data Fig. 6 [file 44318_2025_572_MOESM8_ESM.zip › EMBOJ-2025-121095R_Source Data for Figure 6/Figure 6/Figure 6C/Figure 6C.png]

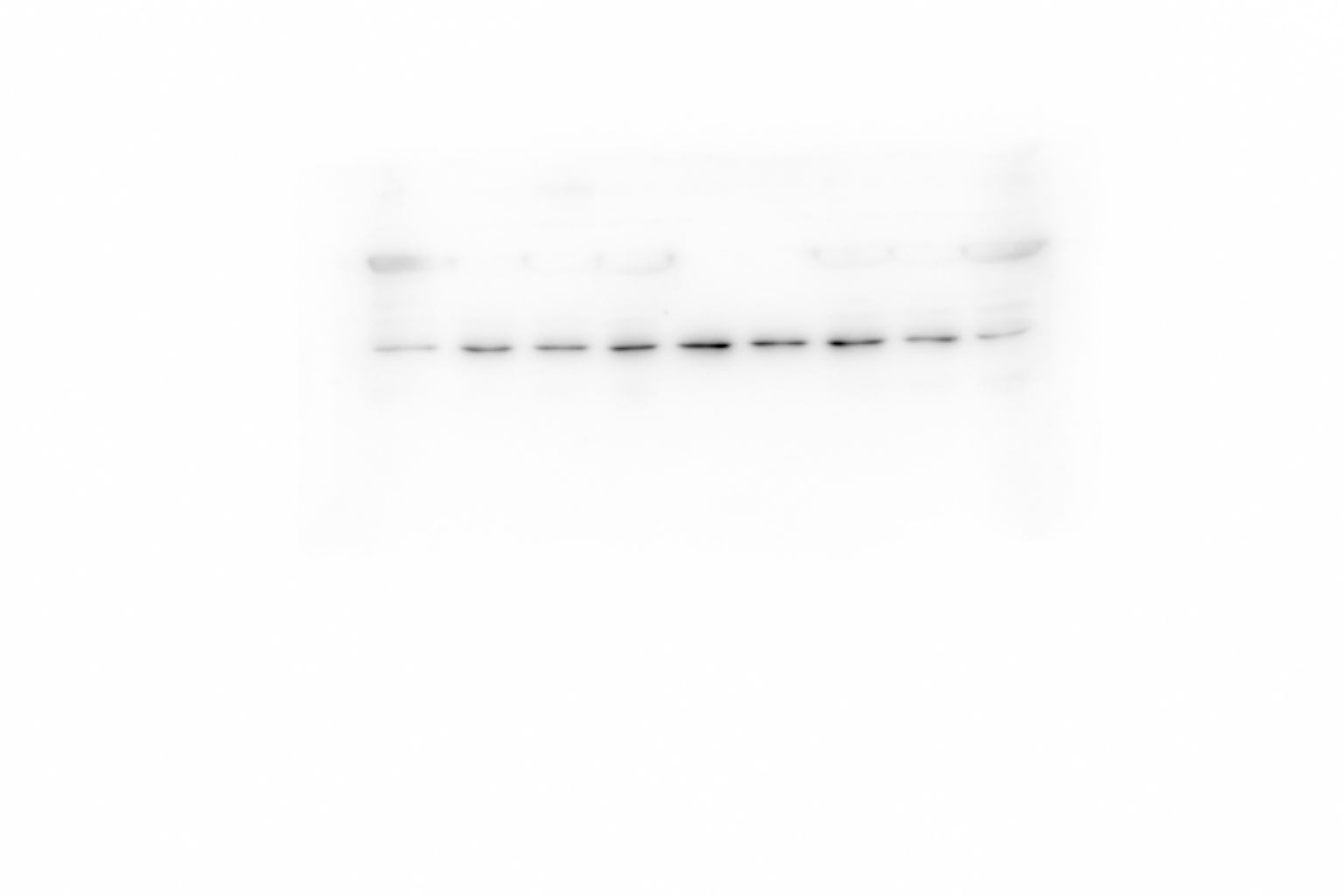

Supplement: Supplementary file 8 — Source data Fig. 6 [file 44318_2025_572_MOESM8_ESM.zip › EMBOJ-2025-121095R_Source Data for Figure 6/Figure 6/Figure 6C/Input Orai3 15 sec.gel]

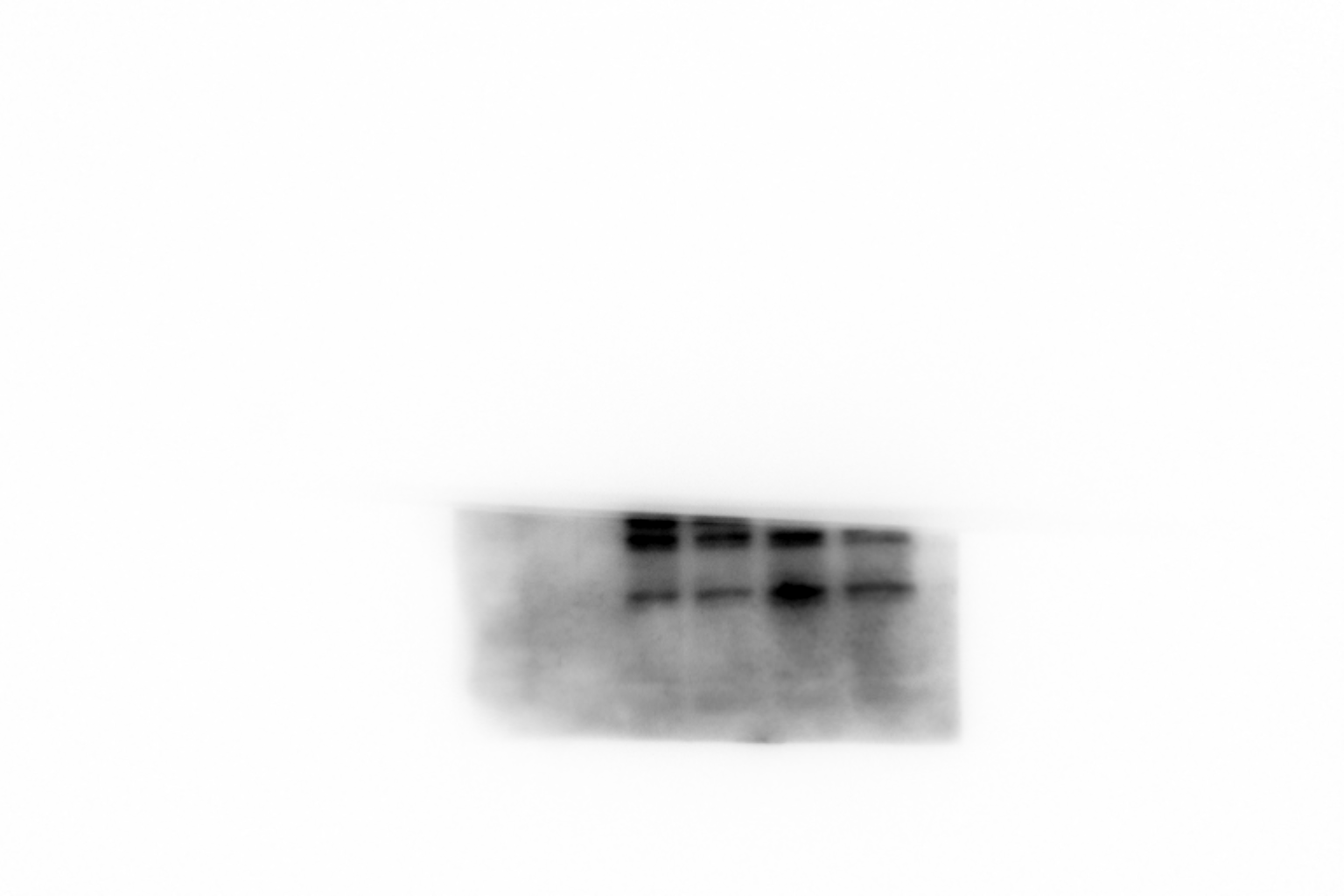

Supplement: Supplementary file 8 — Source data Fig. 6 [file 44318_2025_572_MOESM8_ESM.zip › EMBOJ-2025-121095R_Source Data for Figure 6/Figure 6/Figure 6C/Orai 3 60 sec B1.gel]

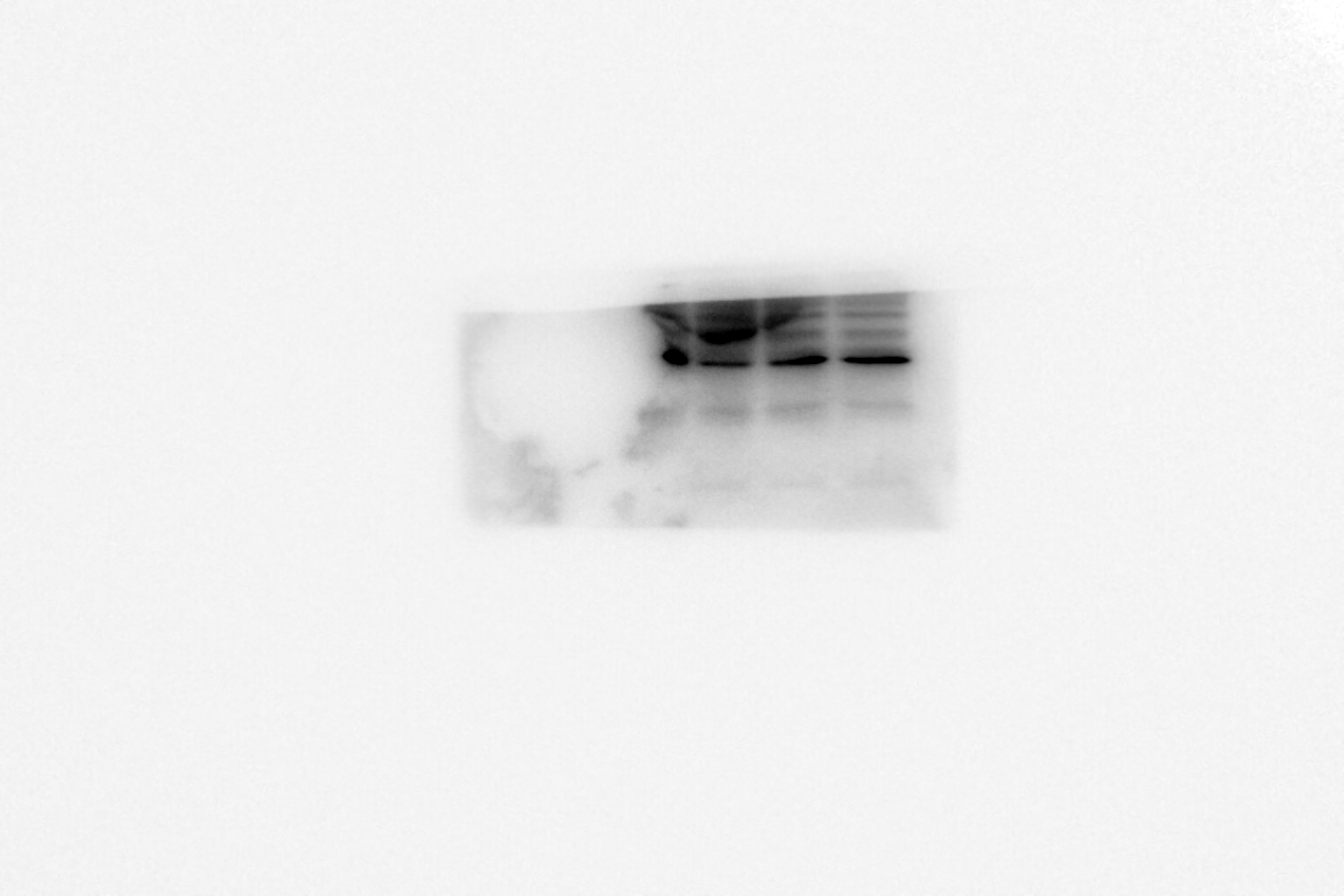

Supplement: Supplementary file 8 — Source data Fig. 6 [file 44318_2025_572_MOESM8_ESM.zip › EMBOJ-2025-121095R_Source Data for Figure 6/Figure 6/Figure 6C/Orai 3 8 sec B2.gel]

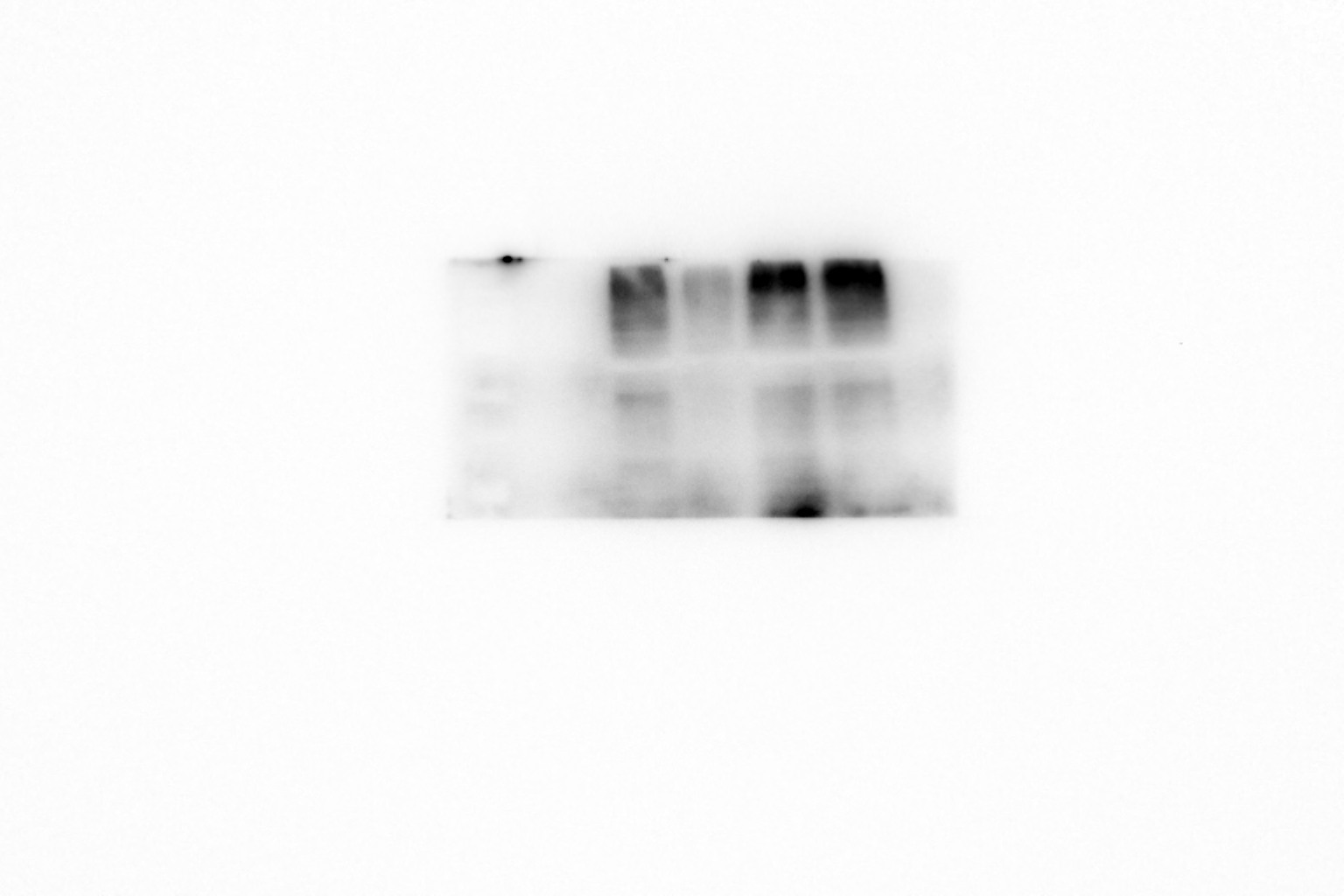

Supplement: Supplementary file 8 — Source data Fig. 6 [file 44318_2025_572_MOESM8_ESM.zip › EMBOJ-2025-121095R_Source Data for Figure 6/Figure 6/Figure 6C/UB 120 sec 2.gel]

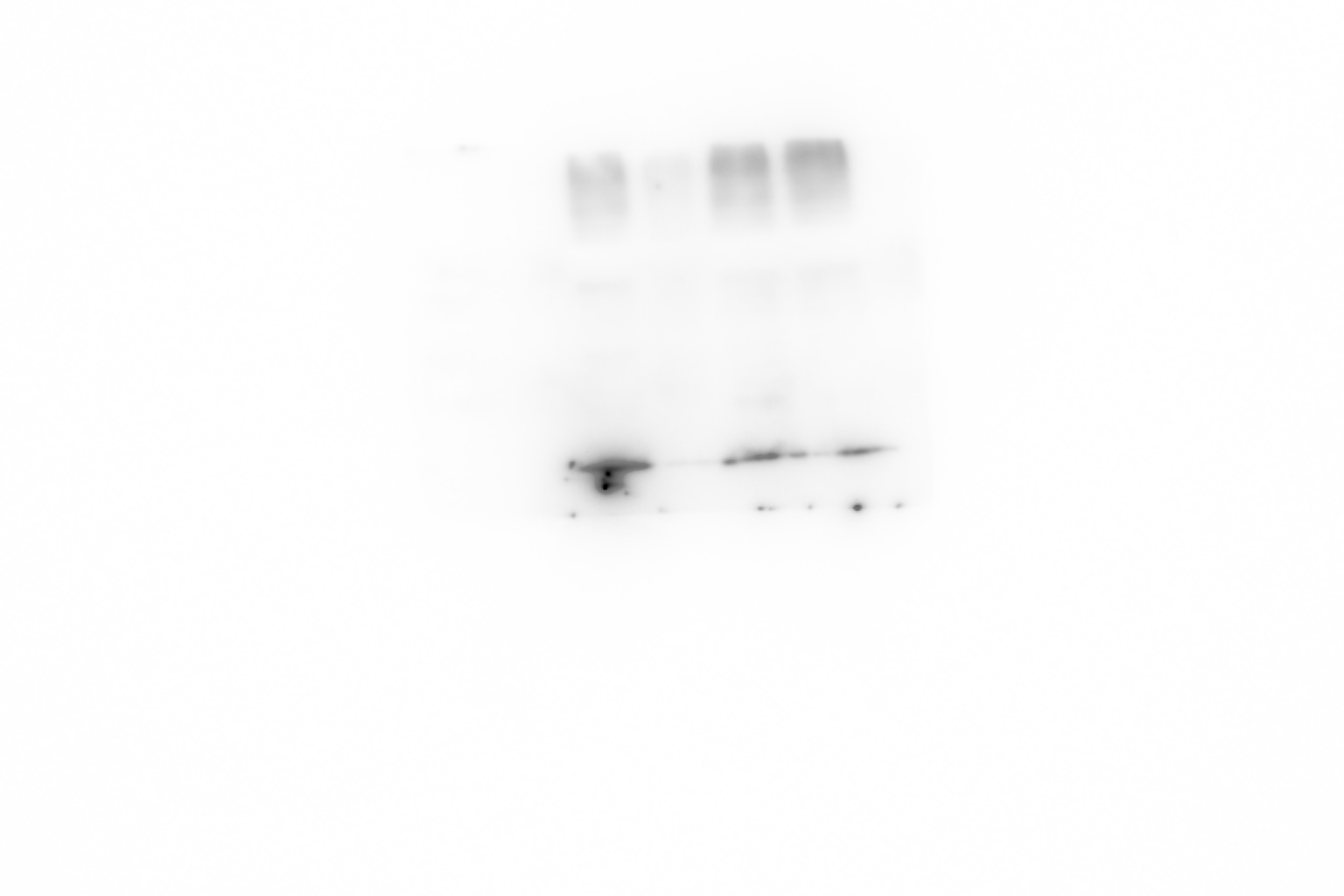

Supplement: Supplementary file 8 — Source data Fig. 6 [file 44318_2025_572_MOESM8_ESM.zip › EMBOJ-2025-121095R_Source Data for Figure 6/Figure 6/Figure 6C/UB 120 sec.gel]

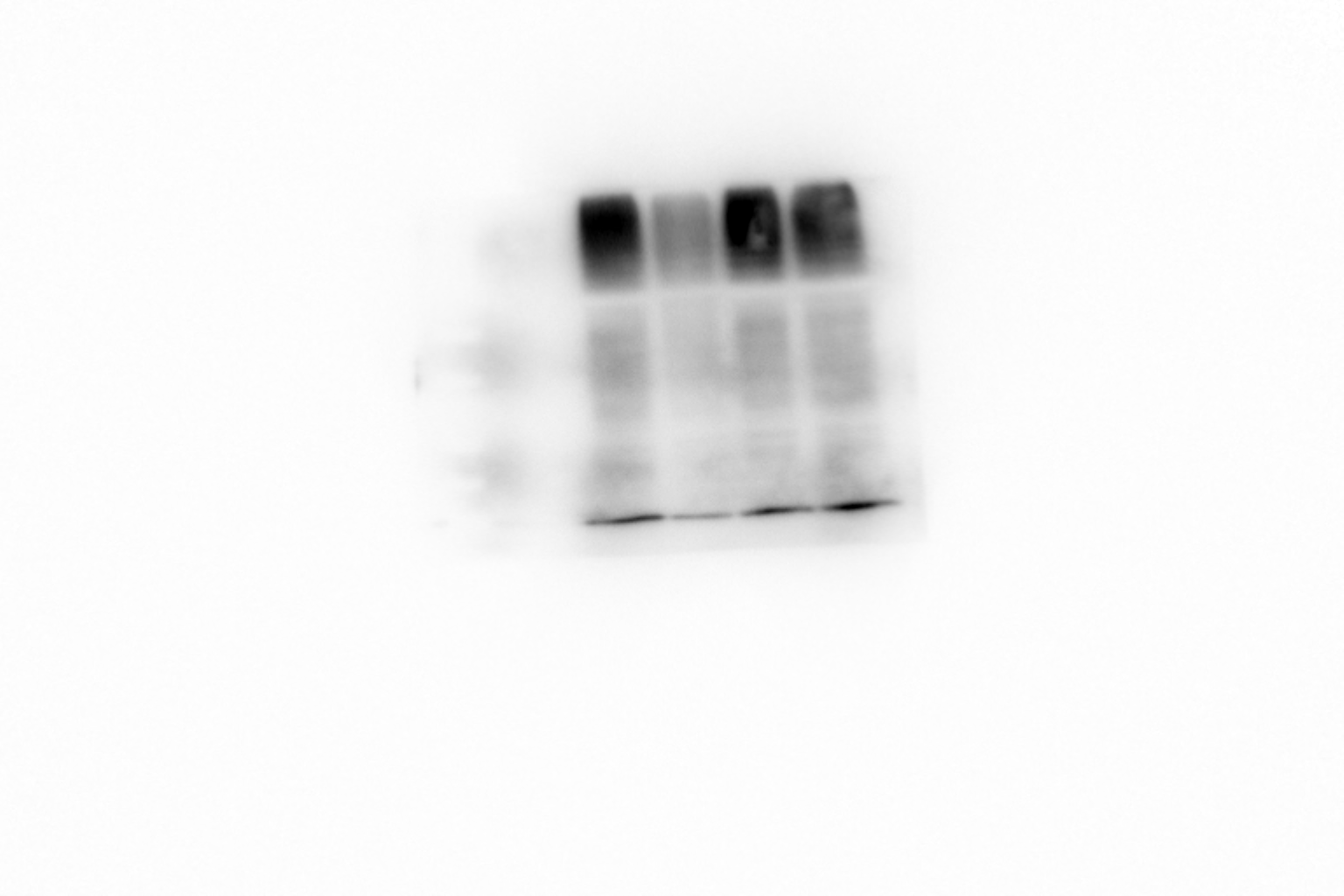

Supplement: Supplementary file 8 — Source data Fig. 6 [file 44318_2025_572_MOESM8_ESM.zip › EMBOJ-2025-121095R_Source Data for Figure 6/Figure 6/Figure 6C/UB 2 sec B2.gel]

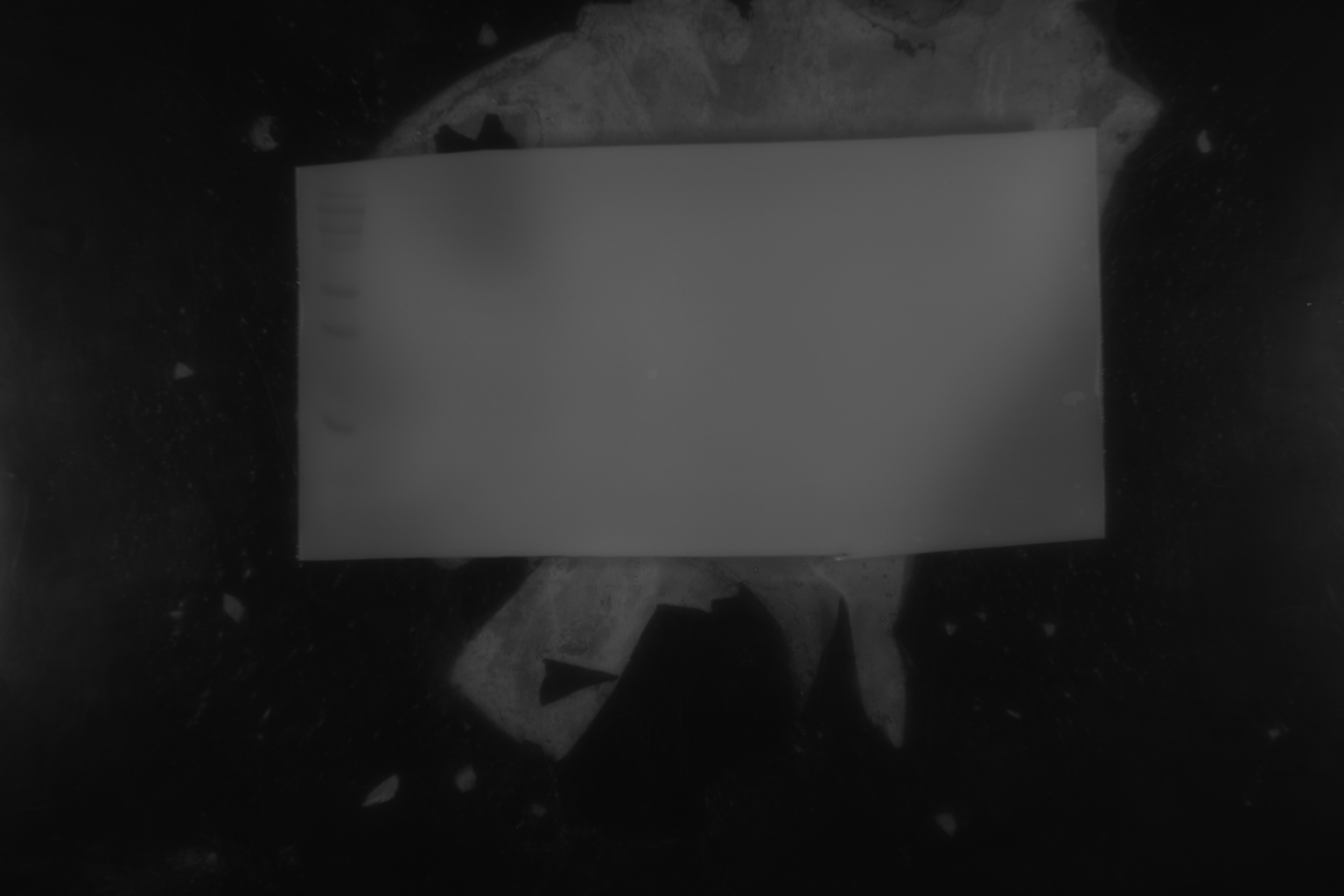

Supplement: Supplementary file 8 — Source data Fig. 6 [file 44318_2025_572_MOESM8_ESM.zip › EMBOJ-2025-121095R_Source Data for Figure 6/Figure 6/Figure 6C/V_Input Orai3 15 sec.gel]

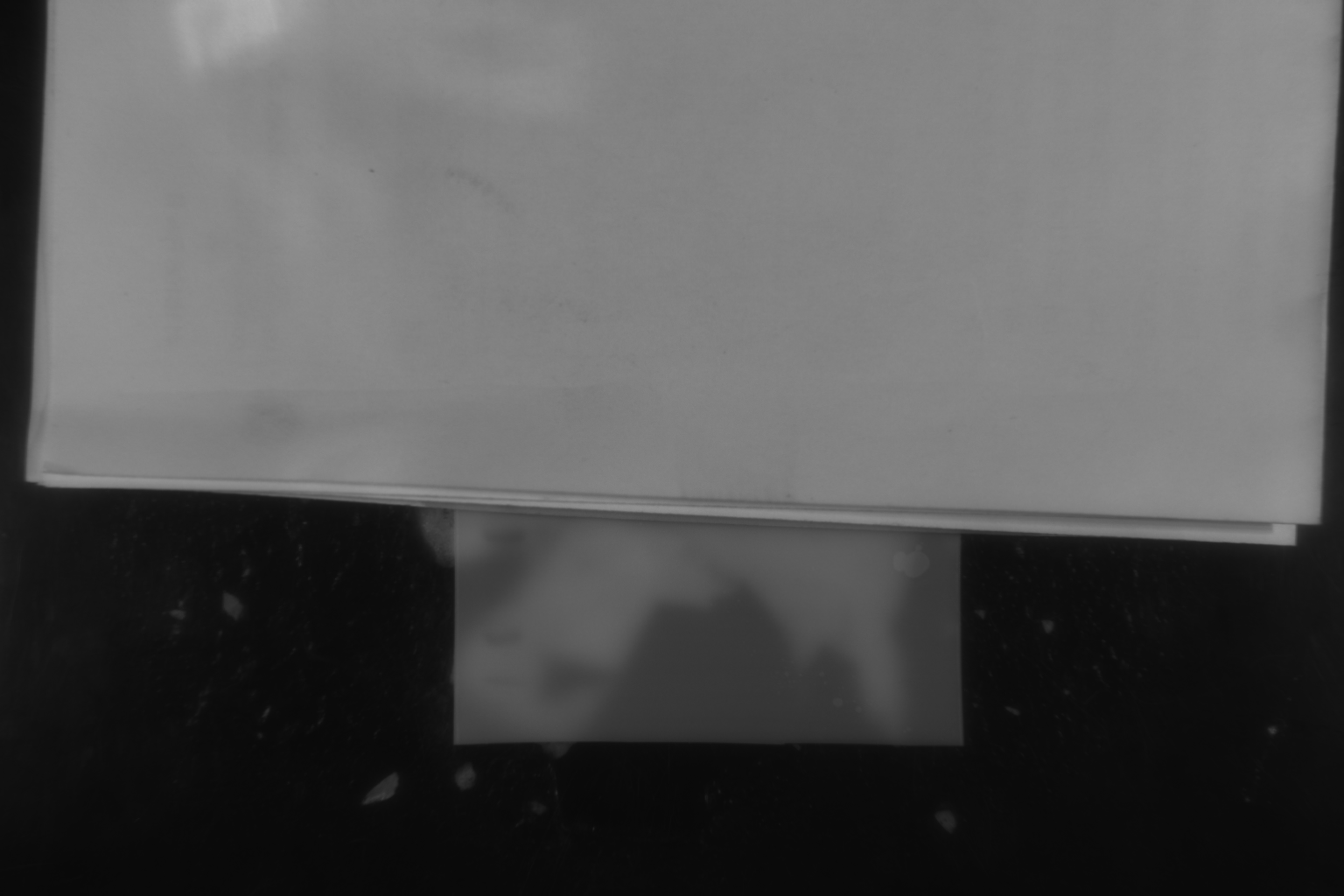

Supplement: Supplementary file 8 — Source data Fig. 6 [file 44318_2025_572_MOESM8_ESM.zip › EMBOJ-2025-121095R_Source Data for Figure 6/Figure 6/Figure 6C/V_Orai 3 60 sec B1.gel]

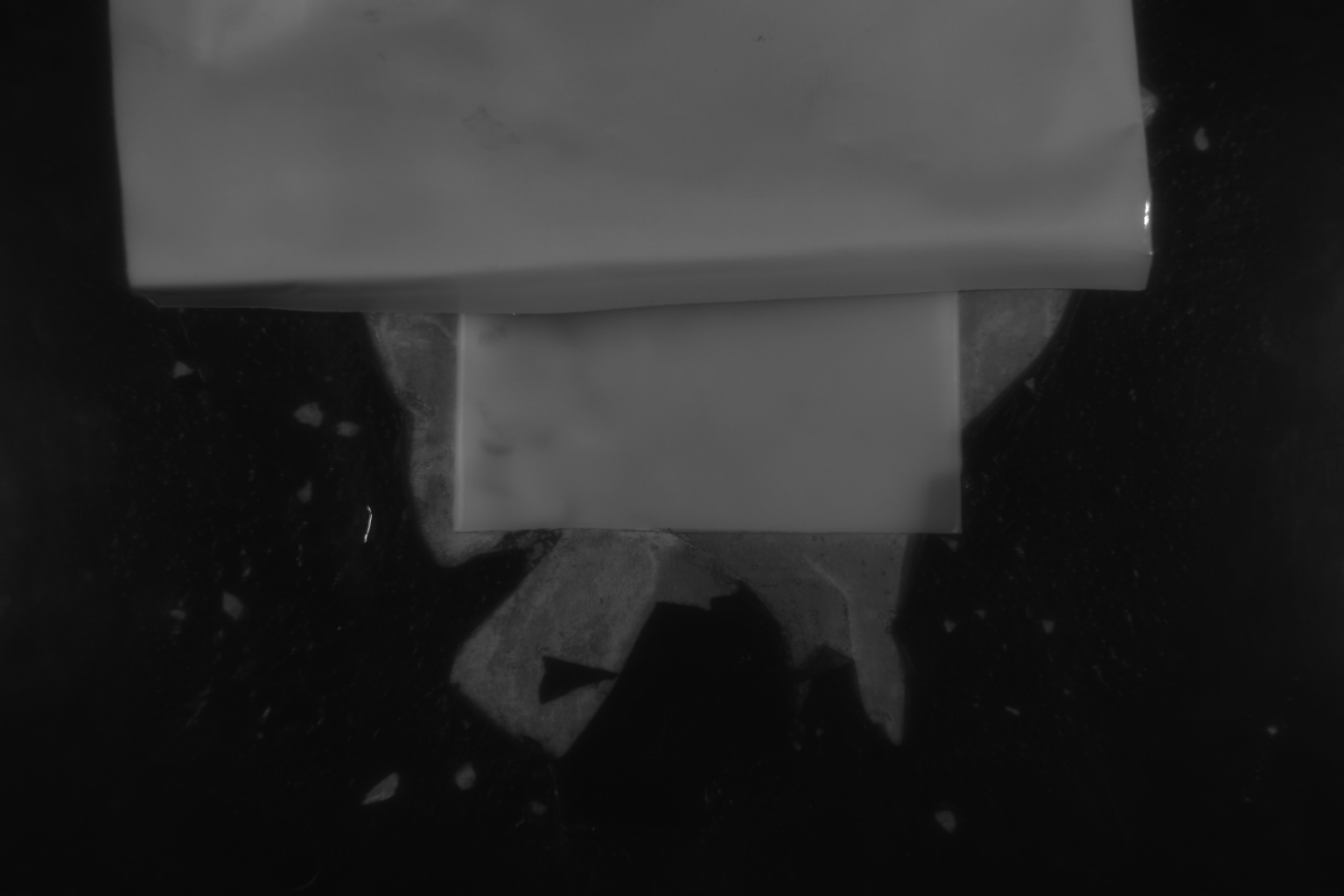

Supplement: Supplementary file 8 — Source data Fig. 6 [file 44318_2025_572_MOESM8_ESM.zip › EMBOJ-2025-121095R_Source Data for Figure 6/Figure 6/Figure 6C/V_Orai 3 8 sec B2.gel]

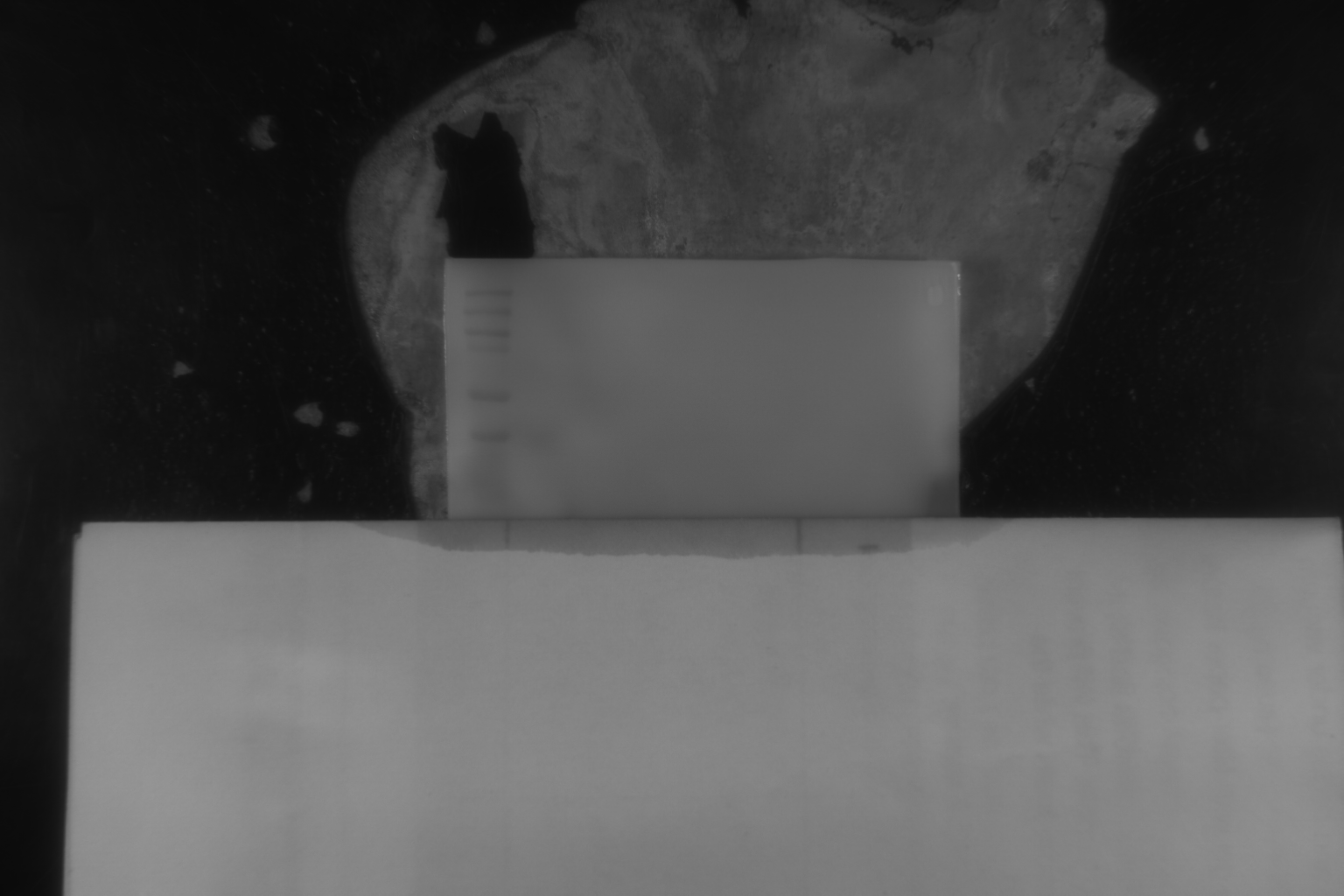

Supplement: Supplementary file 8 — Source data Fig. 6 [file 44318_2025_572_MOESM8_ESM.zip › EMBOJ-2025-121095R_Source Data for Figure 6/Figure 6/Figure 6C/V_UB 120 sec 2.gel]

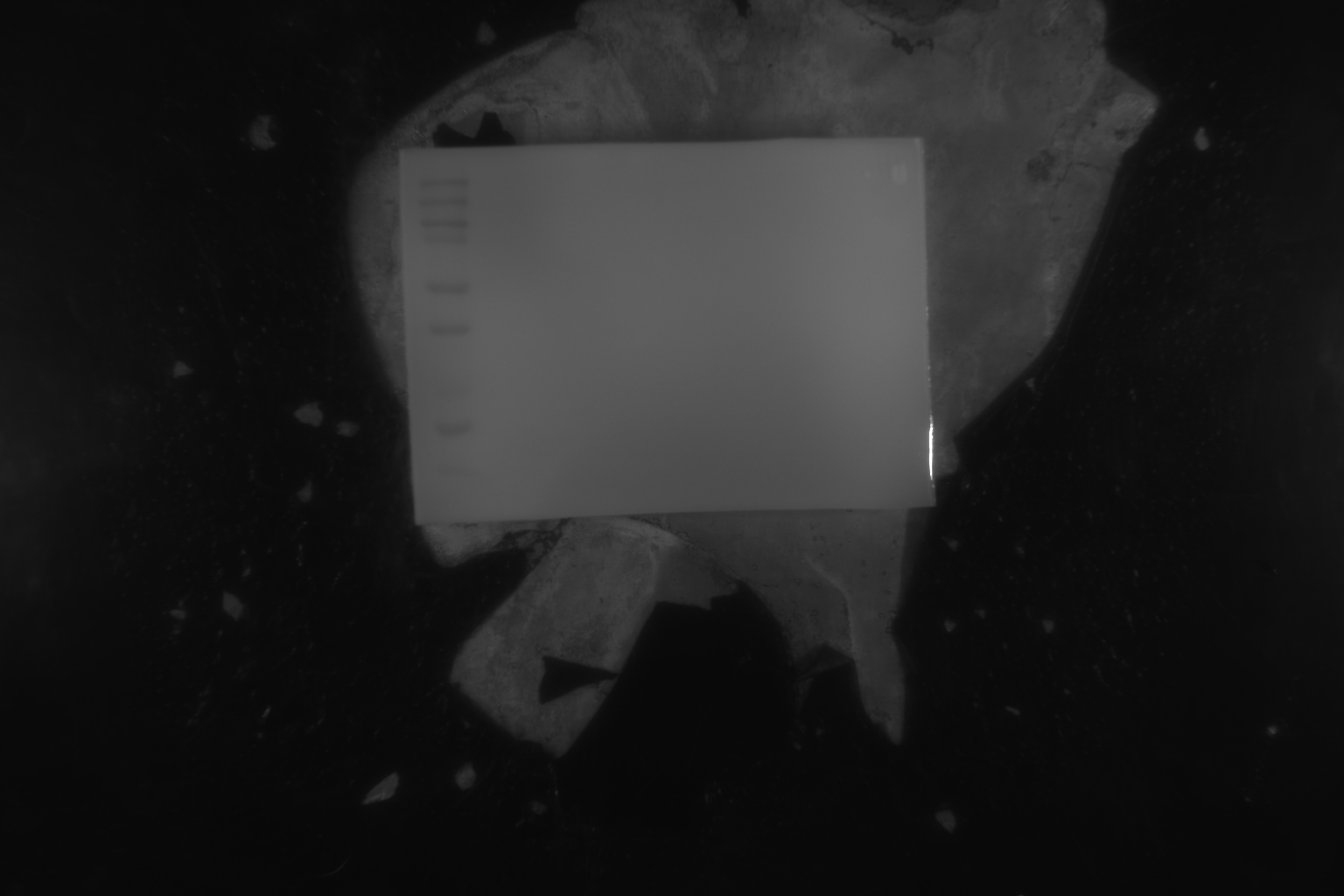

Supplement: Supplementary file 8 — Source data Fig. 6 [file 44318_2025_572_MOESM8_ESM.zip › EMBOJ-2025-121095R_Source Data for Figure 6/Figure 6/Figure 6C/V_UB 120 sec.gel]

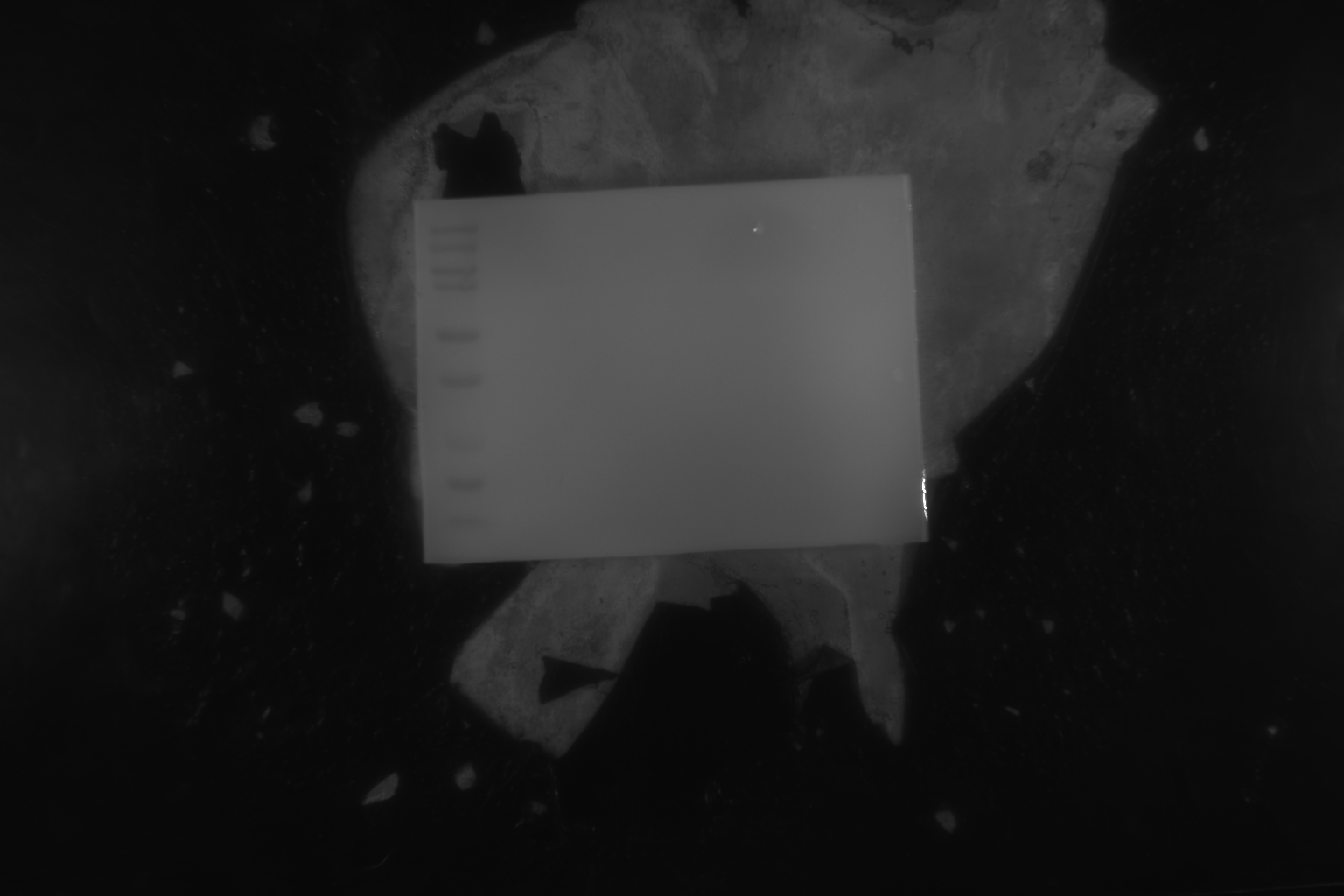

Supplement: Supplementary file 8 — Source data Fig. 6 [file 44318_2025_572_MOESM8_ESM.zip › EMBOJ-2025-121095R_Source Data for Figure 6/Figure 6/Figure 6C/V_UB 2 sec B2.gel]

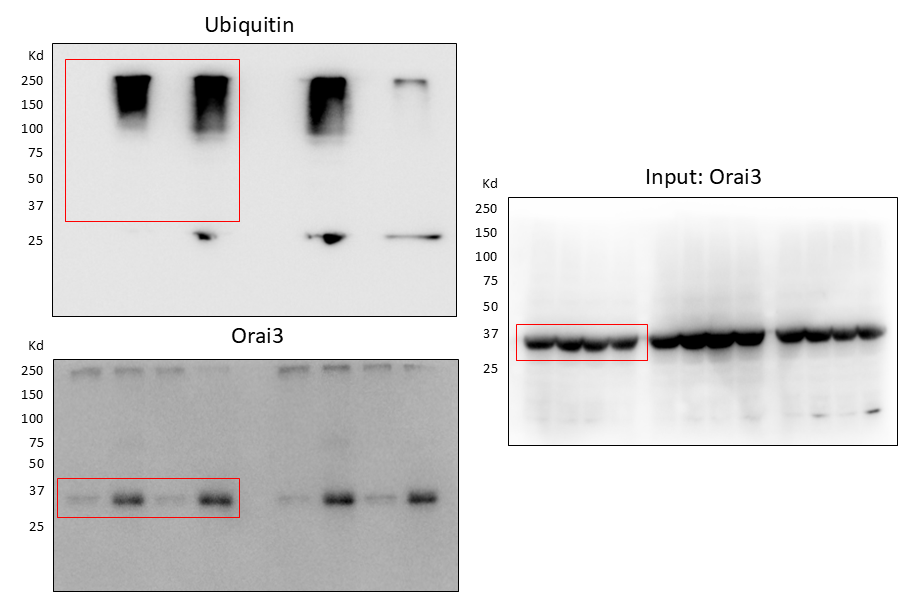

Supplement: Supplementary file 8 — Source data Fig. 6 [file 44318_2025_572_MOESM8_ESM.zip › EMBOJ-2025-121095R_Source Data for Figure 6/Figure 6/Figure 6D/Figure 6D.png]

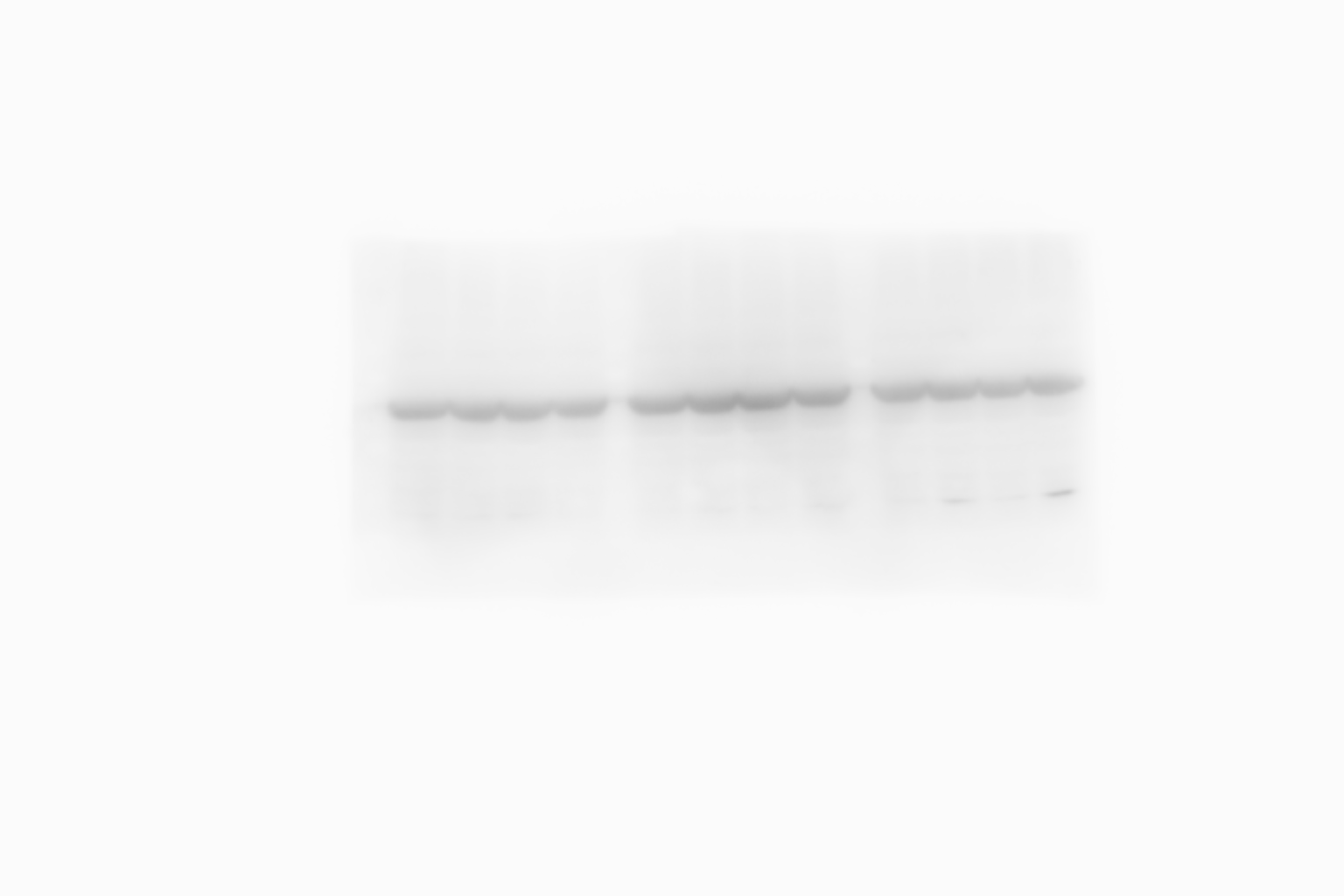

Supplement: Supplementary file 8 — Source data Fig. 6 [file 44318_2025_572_MOESM8_ESM.zip › EMBOJ-2025-121095R_Source Data for Figure 6/Figure 6/Figure 6D/INPUT ORAI 3 10 SEC.gel]

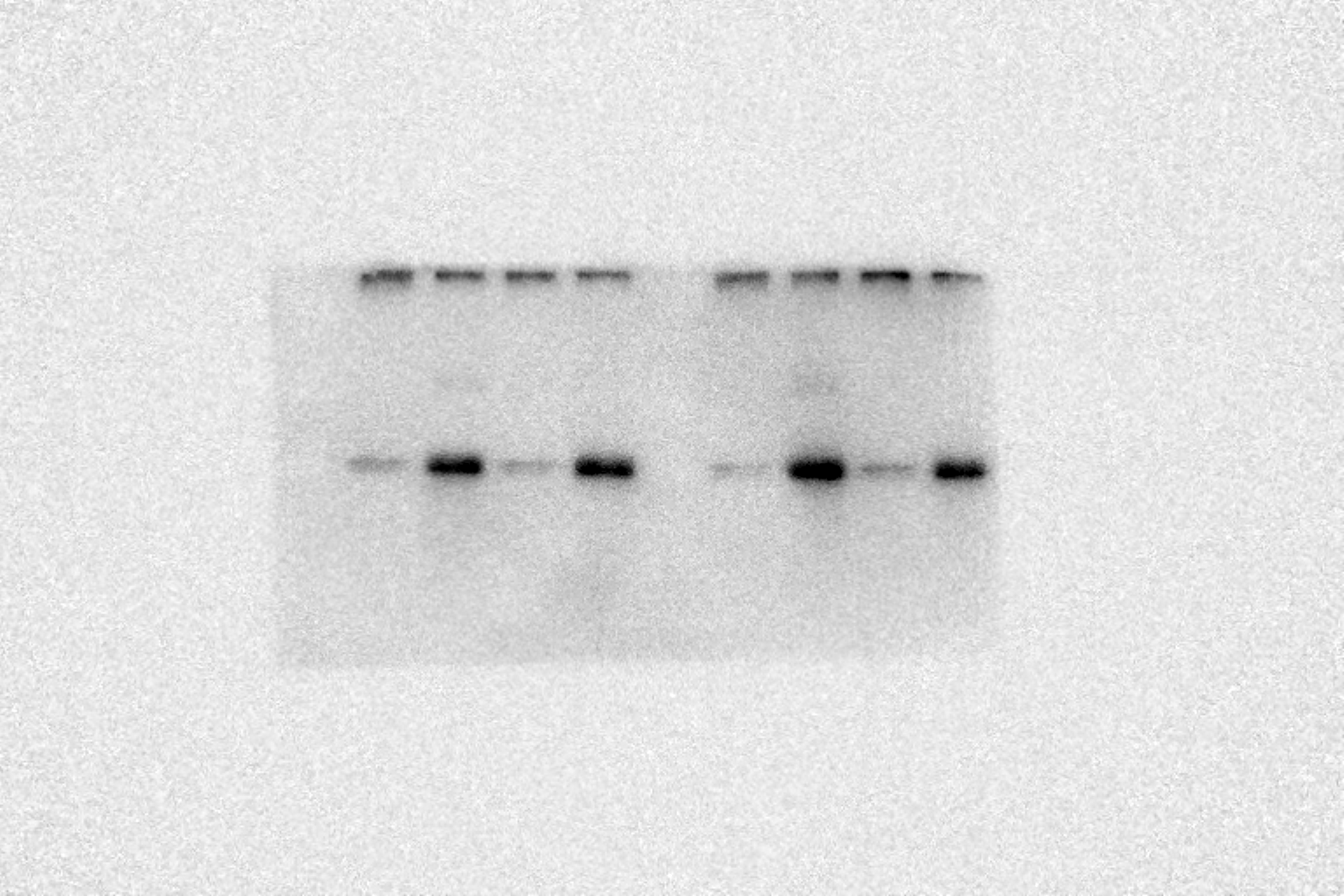

Supplement: Supplementary file 8 — Source data Fig. 6 [file 44318_2025_572_MOESM8_ESM.zip › EMBOJ-2025-121095R_Source Data for Figure 6/Figure 6/Figure 6D/ORAI 3 IP 0.5 SEC 2B.gel]

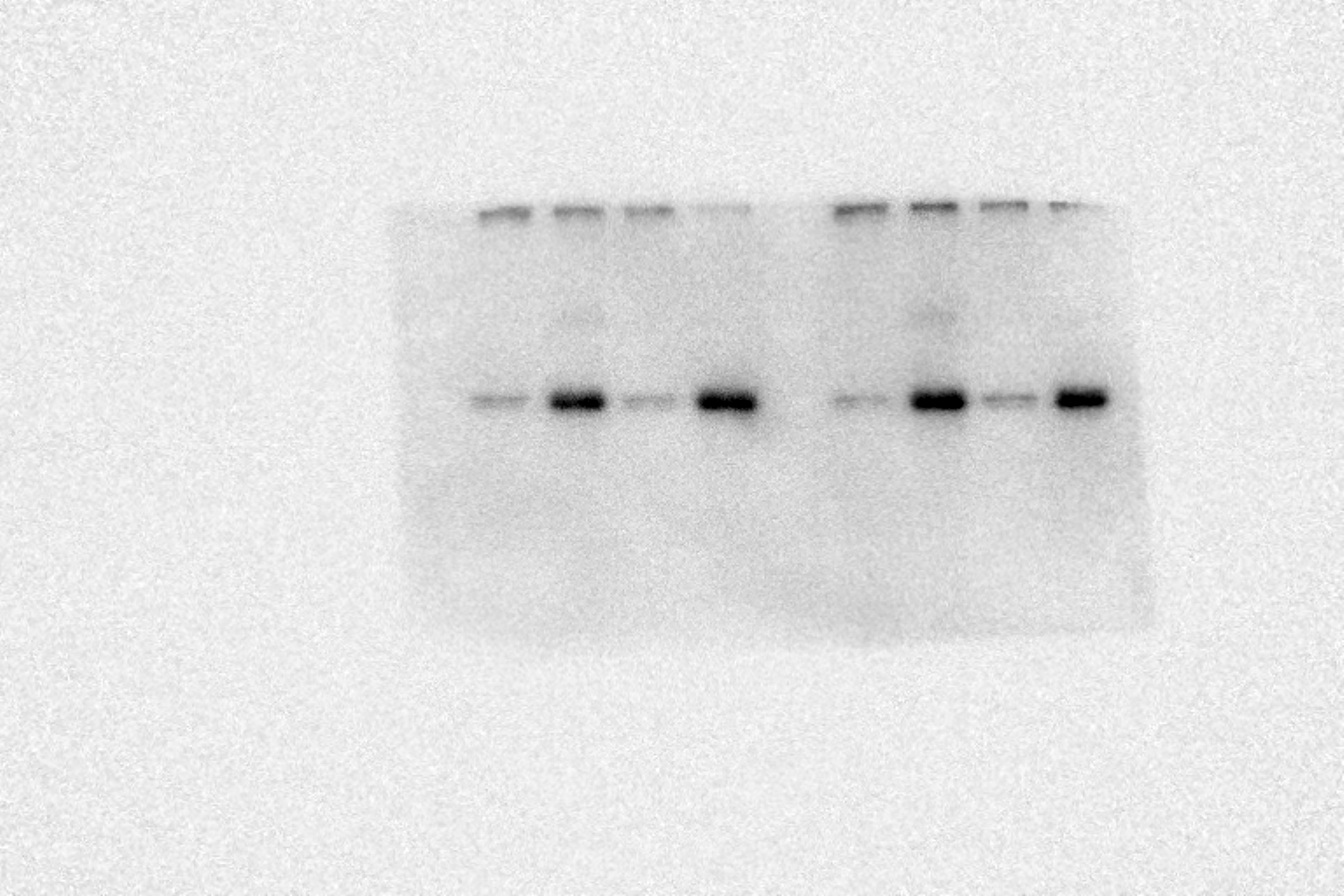

Supplement: Supplementary file 8 — Source data Fig. 6 [file 44318_2025_572_MOESM8_ESM.zip › EMBOJ-2025-121095R_Source Data for Figure 6/Figure 6/Figure 6D/ORAI 3 IP 1 SEC.gel]

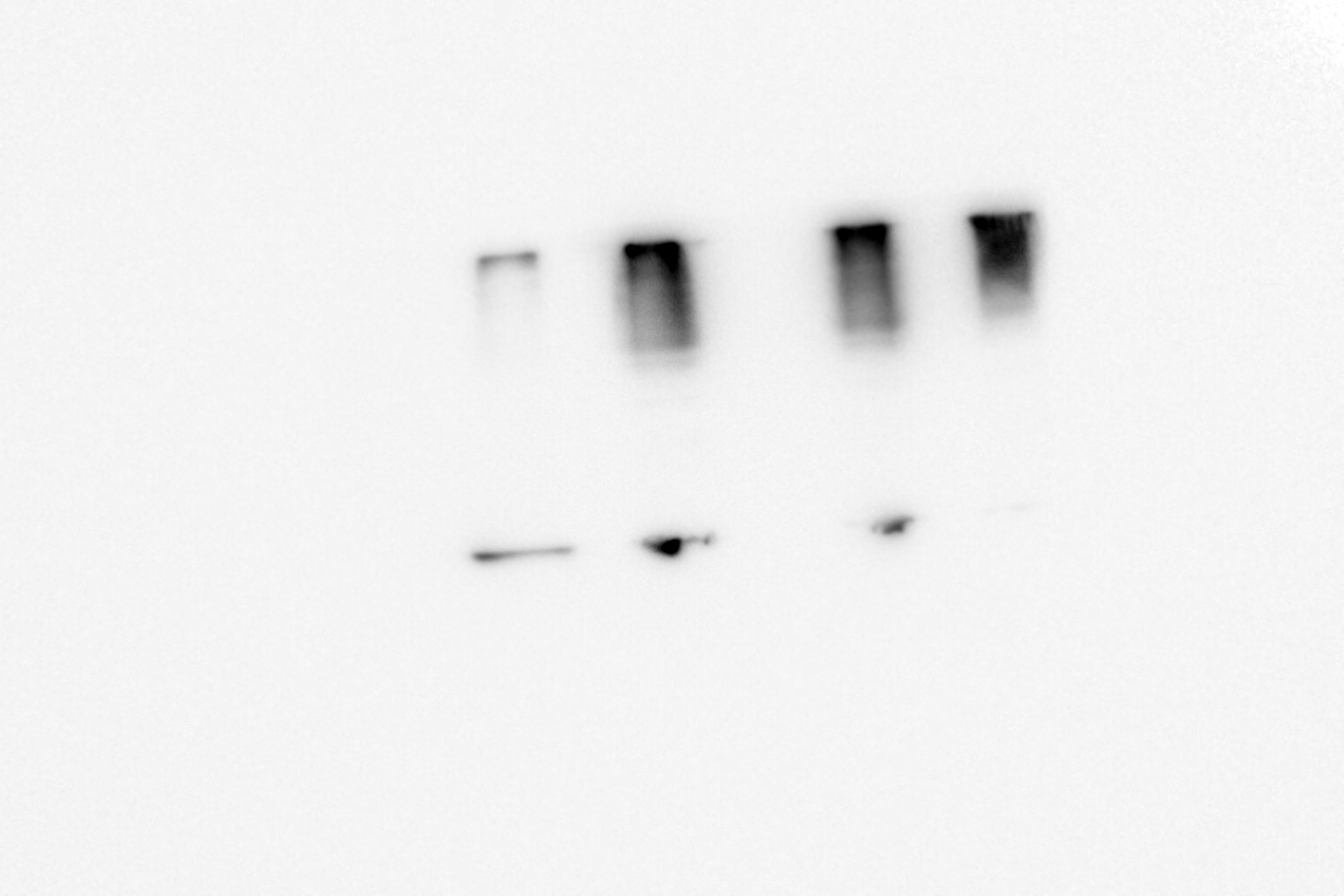

Supplement: Supplementary file 8 — Source data Fig. 6 [file 44318_2025_572_MOESM8_ESM.zip › EMBOJ-2025-121095R_Source Data for Figure 6/Figure 6/Figure 6D/Ubiqutin IP 8 SEC.gel]

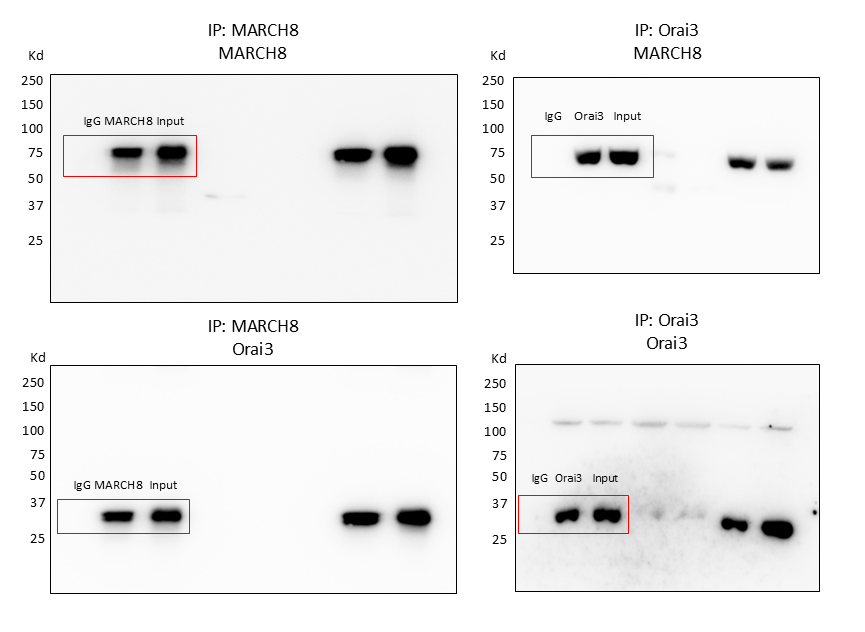

Supplement: Supplementary file 8 — Source data Fig. 6 [file 44318_2025_572_MOESM8_ESM.zip › EMBOJ-2025-121095R_Source Data for Figure 6/Figure 6/Figure 6E/Figure 6E.png]

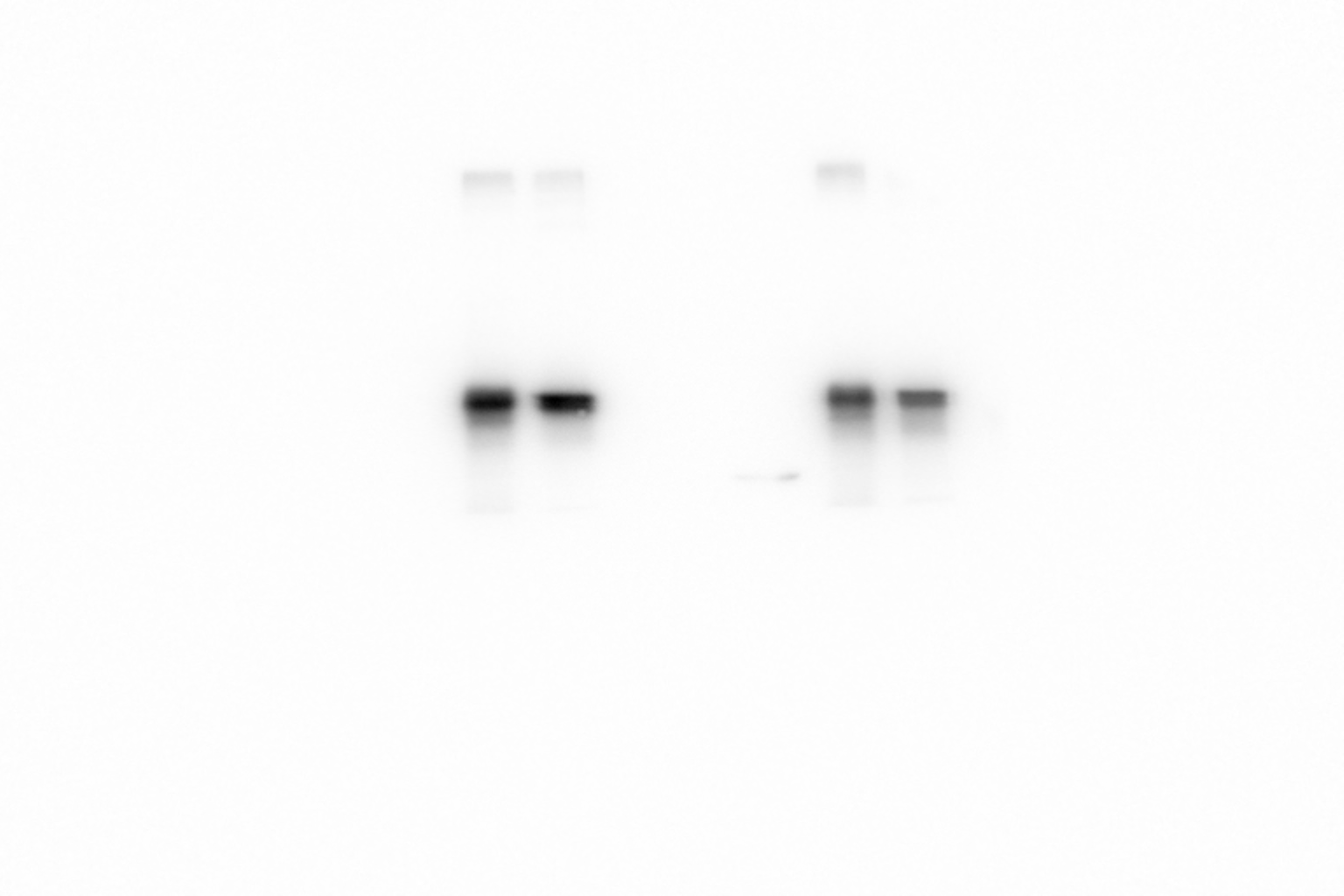

Supplement: Supplementary file 8 — Source data Fig. 6 [file 44318_2025_572_MOESM8_ESM.zip › EMBOJ-2025-121095R_Source Data for Figure 6/Figure 6/Figure 6E/MARCH 8 MARCH8 IP.gel]

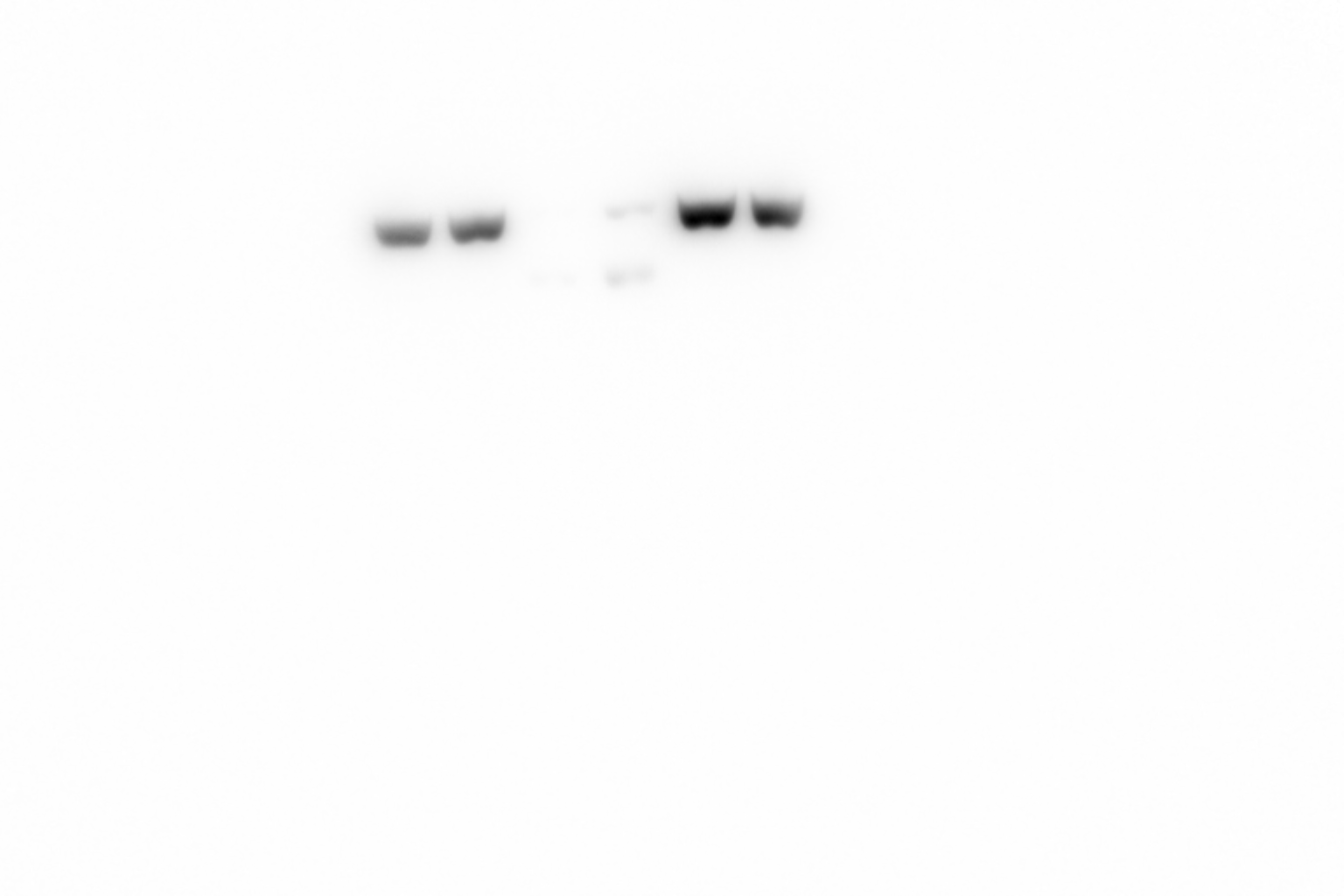

Supplement: Supplementary file 8 — Source data Fig. 6 [file 44318_2025_572_MOESM8_ESM.zip › EMBOJ-2025-121095R_Source Data for Figure 6/Figure 6/Figure 6E/MARCH8 Orai3 IP.gel]

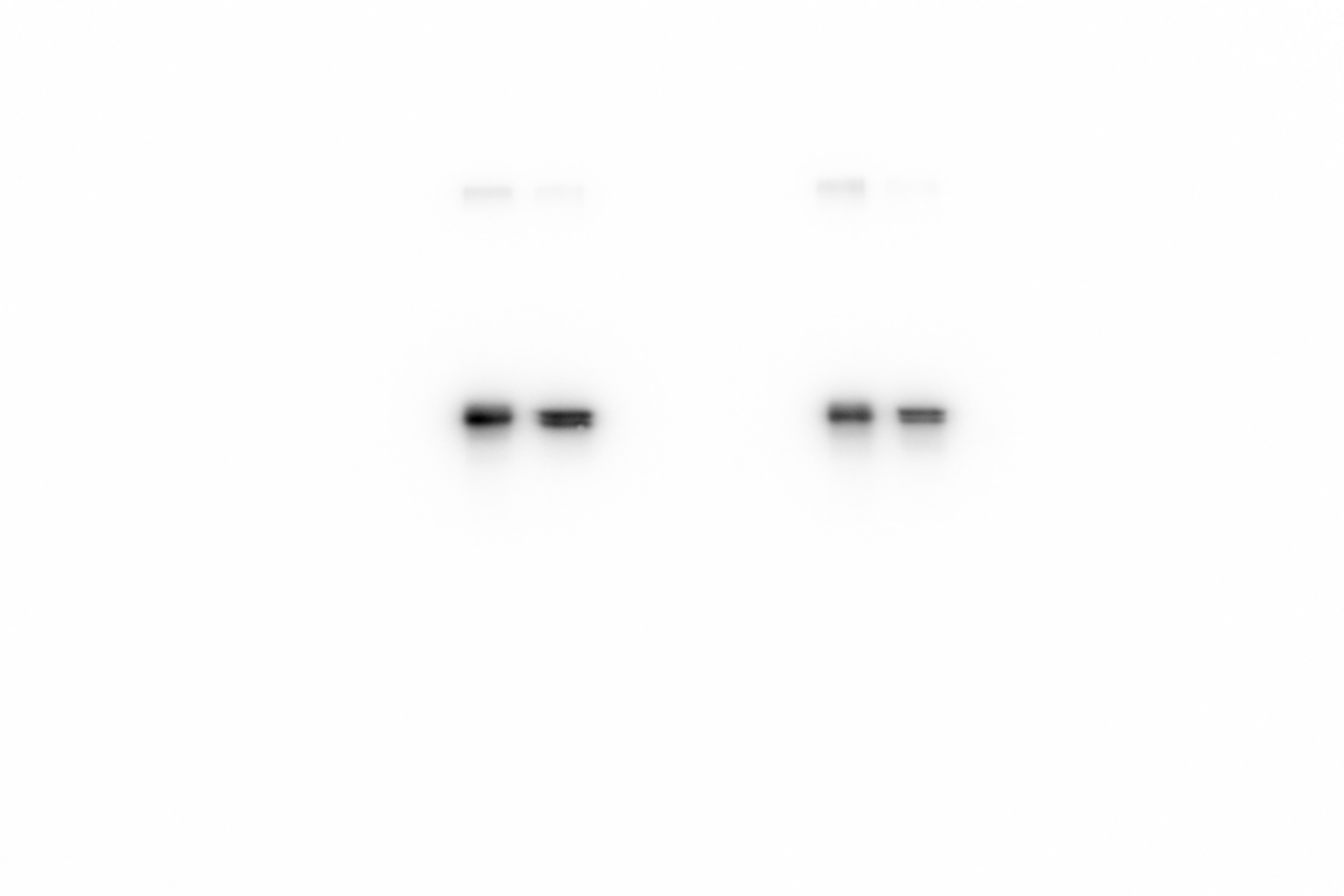

Supplement: Supplementary file 8 — Source data Fig. 6 [file 44318_2025_572_MOESM8_ESM.zip › EMBOJ-2025-121095R_Source Data for Figure 6/Figure 6/Figure 6E/ORAI 3 MARCH8 IP.gel]

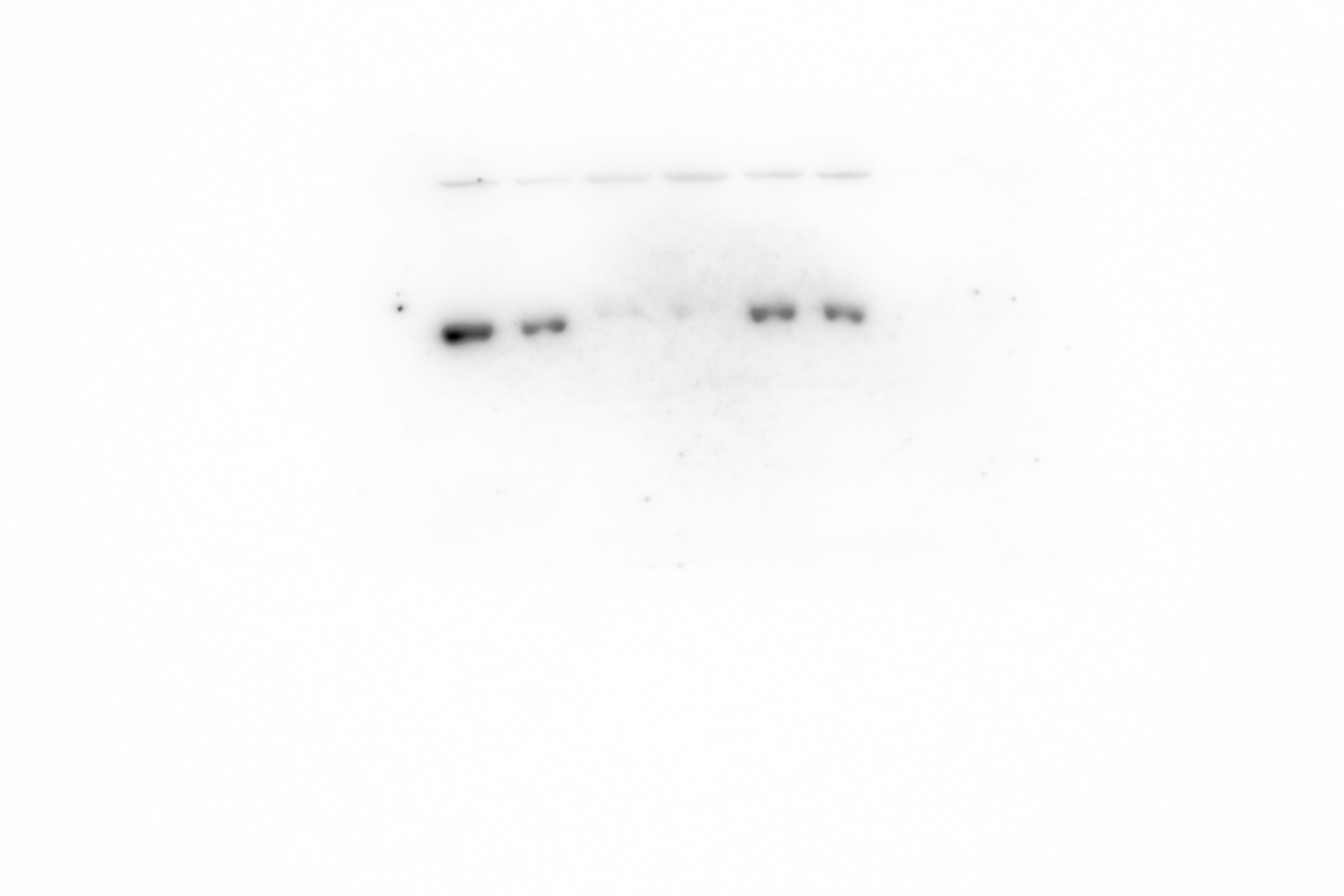

Supplement: Supplementary file 8 — Source data Fig. 6 [file 44318_2025_572_MOESM8_ESM.zip › EMBOJ-2025-121095R_Source Data for Figure 6/Figure 6/Figure 6E/Orai3 Orai3 IP.gel]

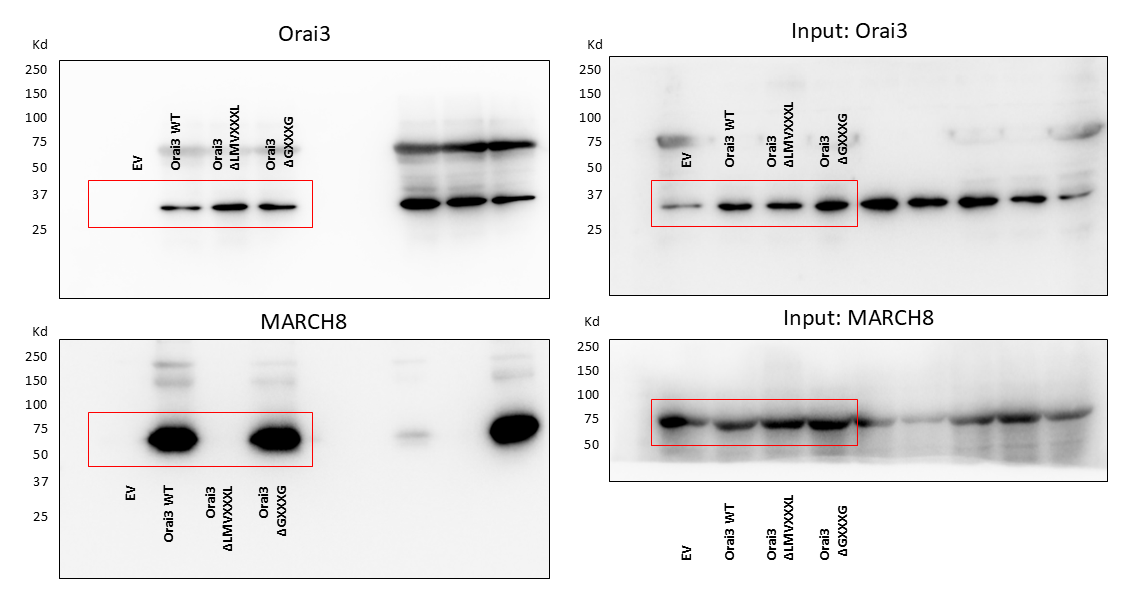

Supplement: Supplementary file 8 — Source data Fig. 6 [file 44318_2025_572_MOESM8_ESM.zip › EMBOJ-2025-121095R_Source Data for Figure 6/Figure 6/Figure 6F/Figure 6F.png]

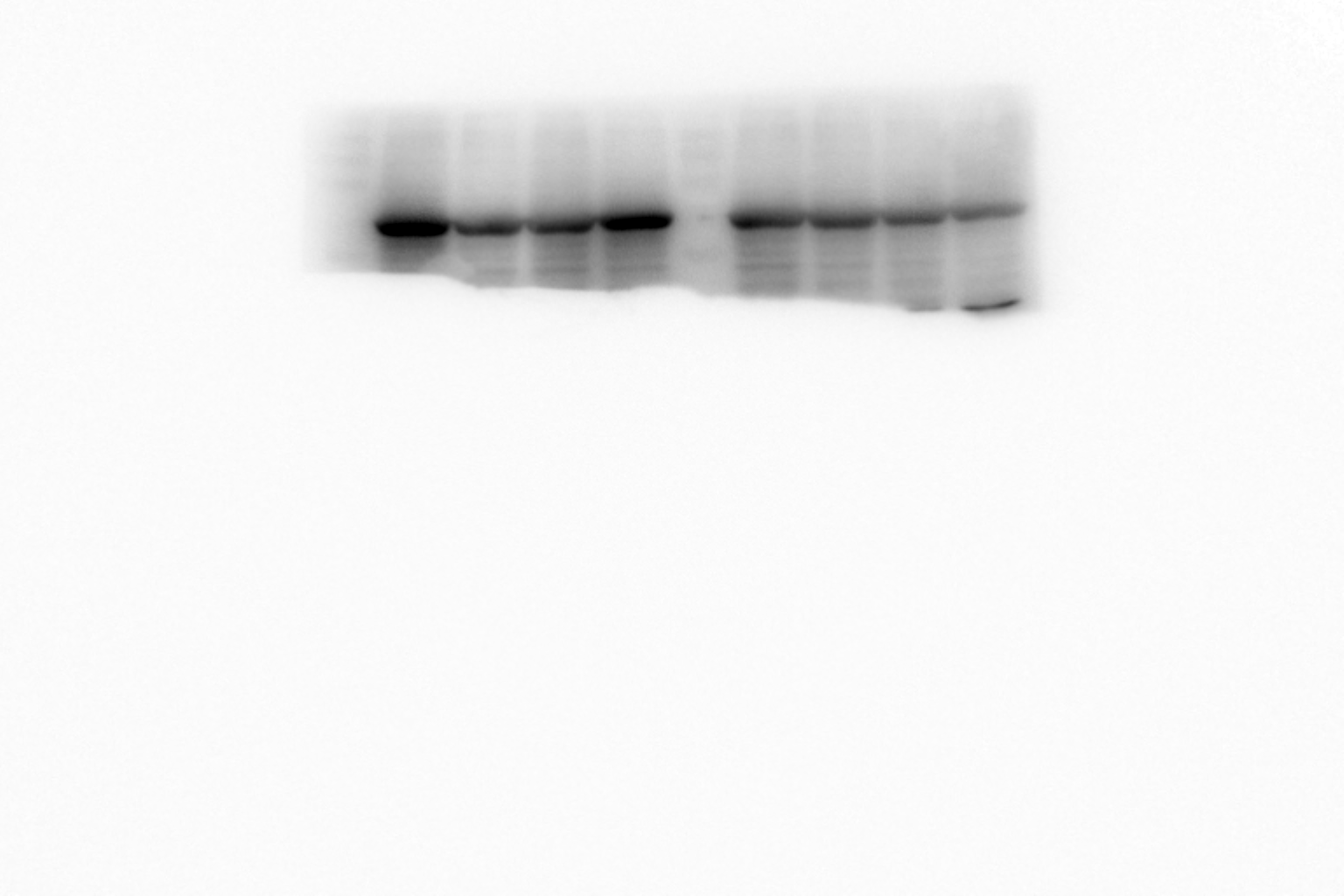

Supplement: Supplementary file 8 — Source data Fig. 6 [file 44318_2025_572_MOESM8_ESM.zip › EMBOJ-2025-121095R_Source Data for Figure 6/Figure 6/Figure 6F/Input MARCH8 4 SEC.gel]

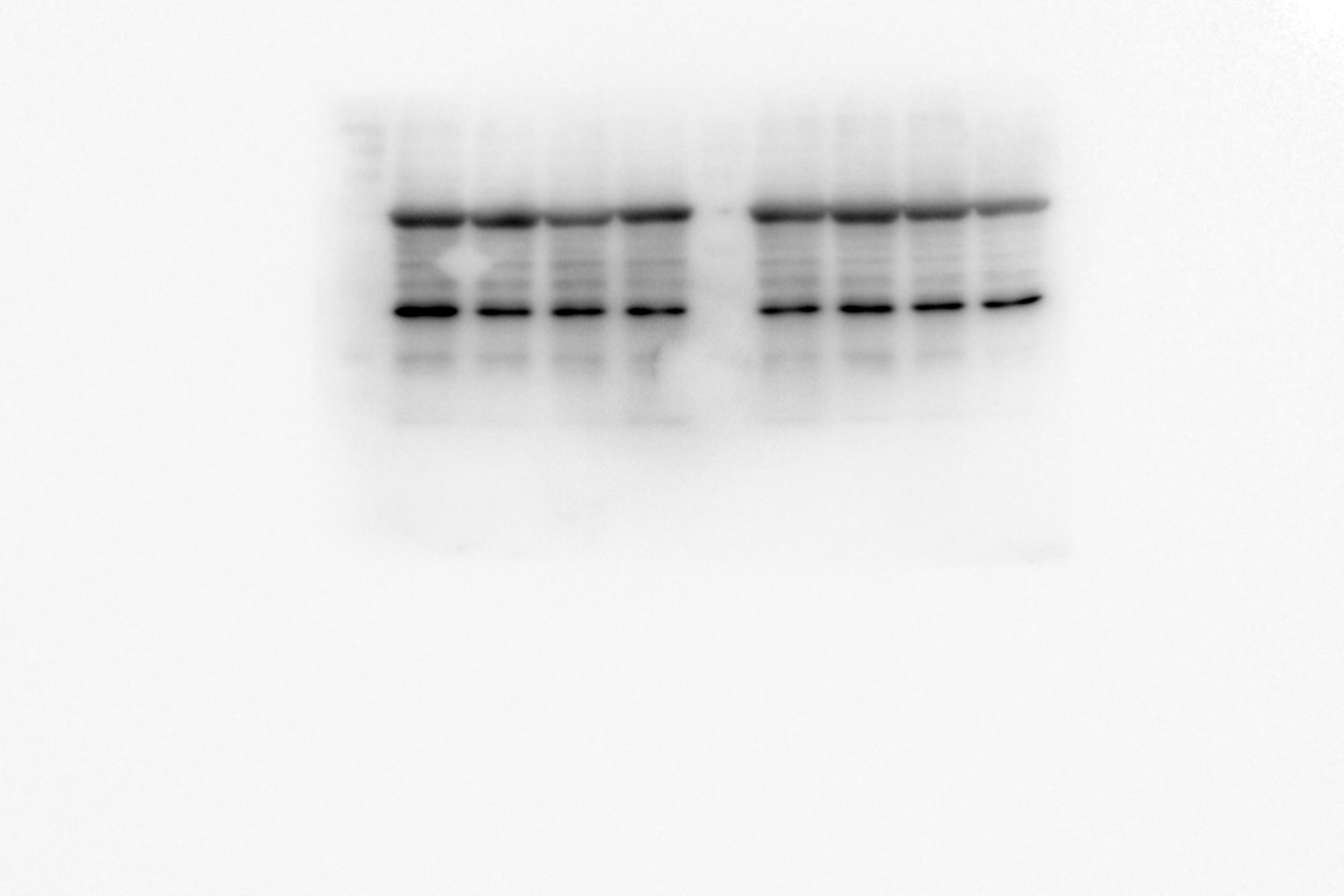

Supplement: Supplementary file 8 — Source data Fig. 6 [file 44318_2025_572_MOESM8_ESM.zip › EMBOJ-2025-121095R_Source Data for Figure 6/Figure 6/Figure 6F/Input MARCH8 8 SEC.gel]

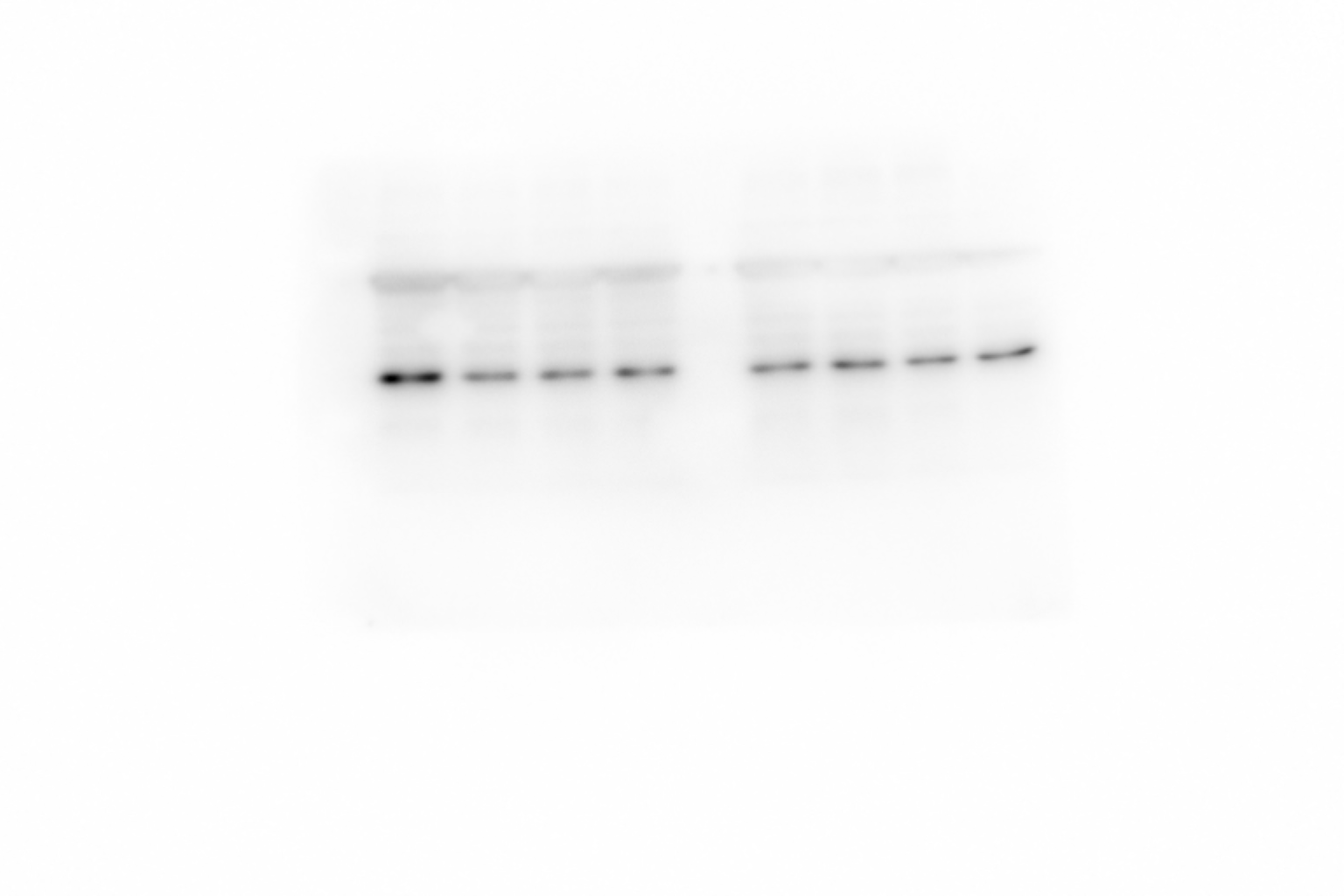

Supplement: Supplementary file 8 — Source data Fig. 6 [file 44318_2025_572_MOESM8_ESM.zip › EMBOJ-2025-121095R_Source Data for Figure 6/Figure 6/Figure 6F/Input Orai3 15 sec.gel]

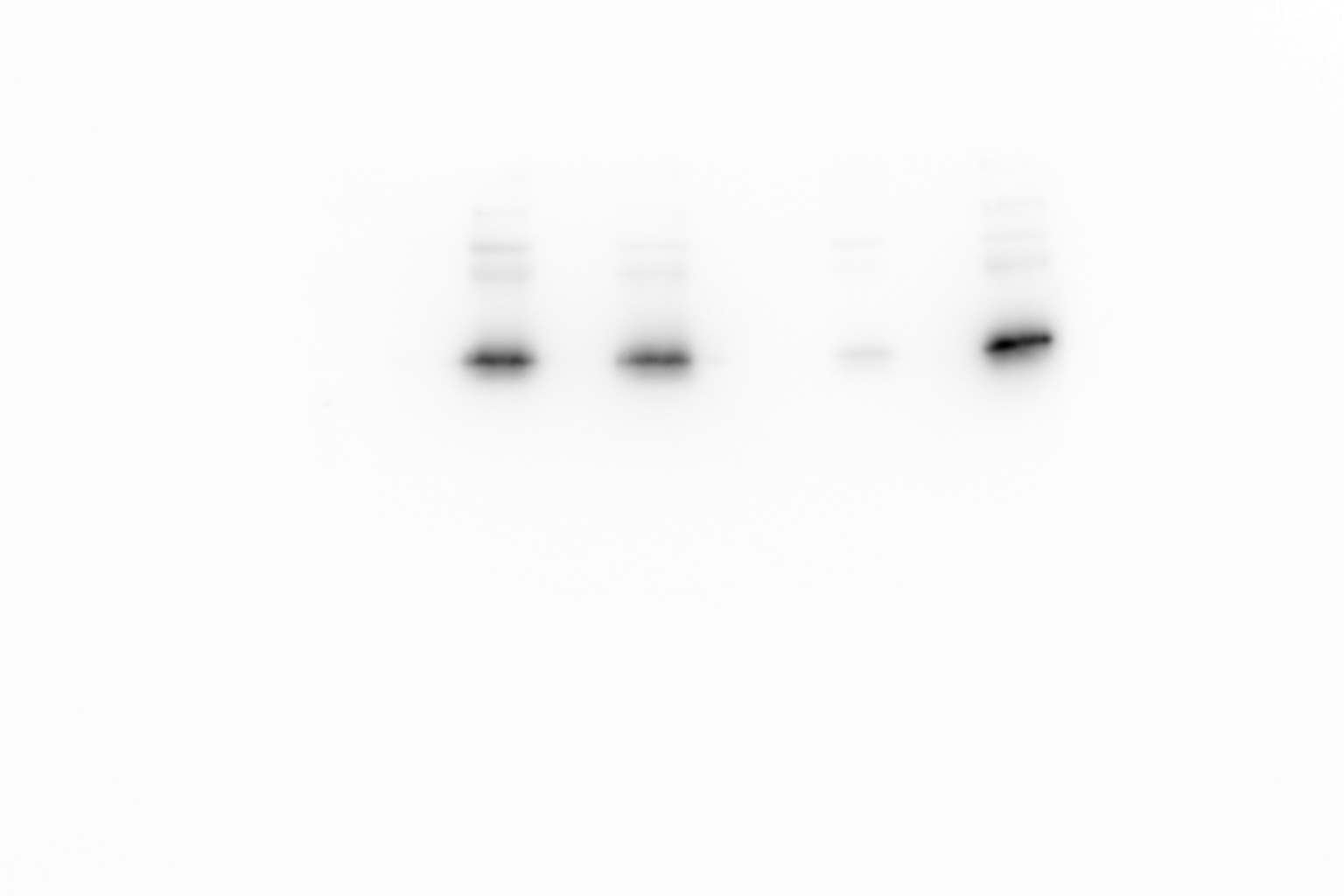

Supplement: Supplementary file 8 — Source data Fig. 6 [file 44318_2025_572_MOESM8_ESM.zip › EMBOJ-2025-121095R_Source Data for Figure 6/Figure 6/Figure 6F/MARCH 8 8 SEC.gel]

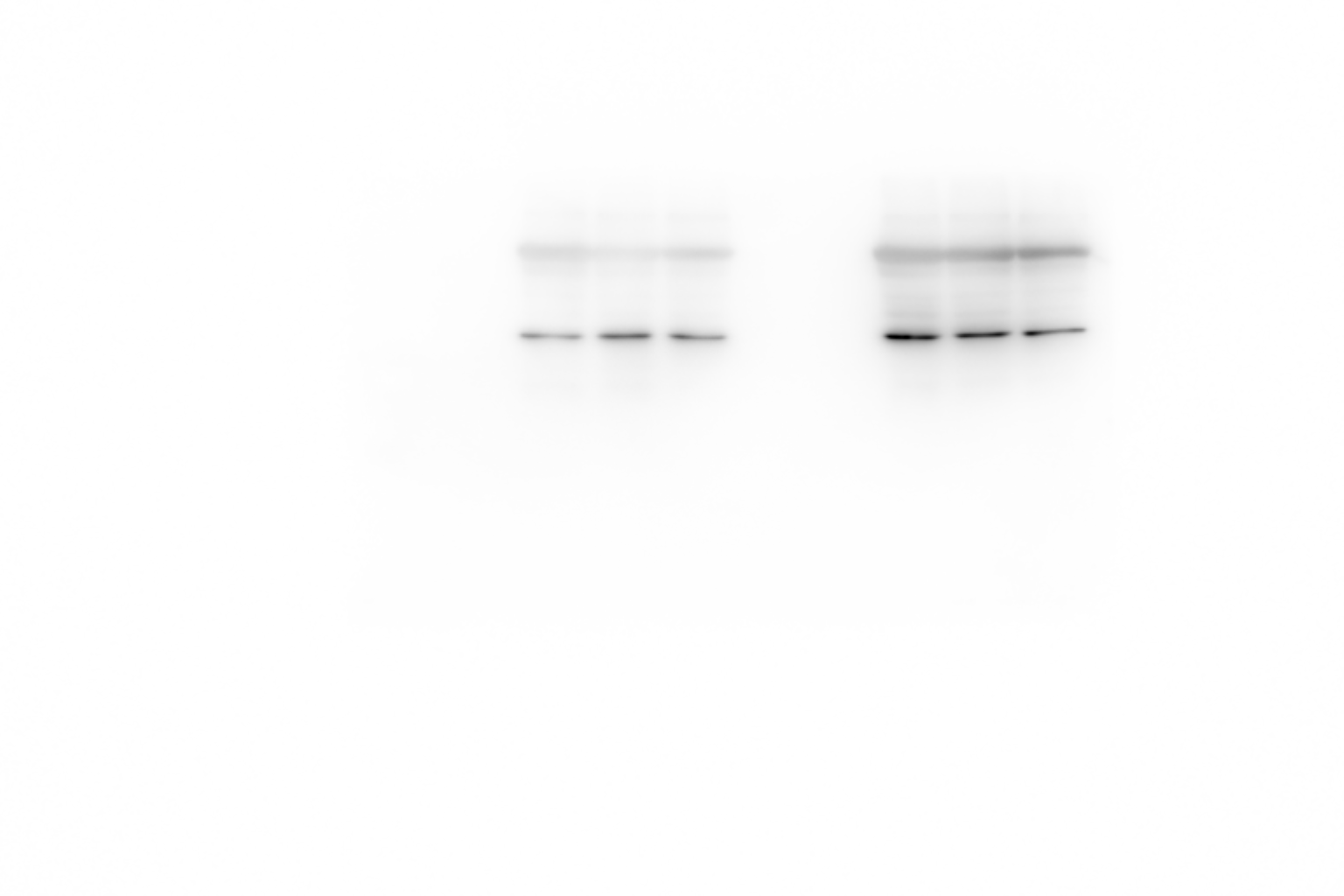

Supplement: Supplementary file 8 — Source data Fig. 6 [file 44318_2025_572_MOESM8_ESM.zip › EMBOJ-2025-121095R_Source Data for Figure 6/Figure 6/Figure 6F/Orai3 60 SEC.gel]

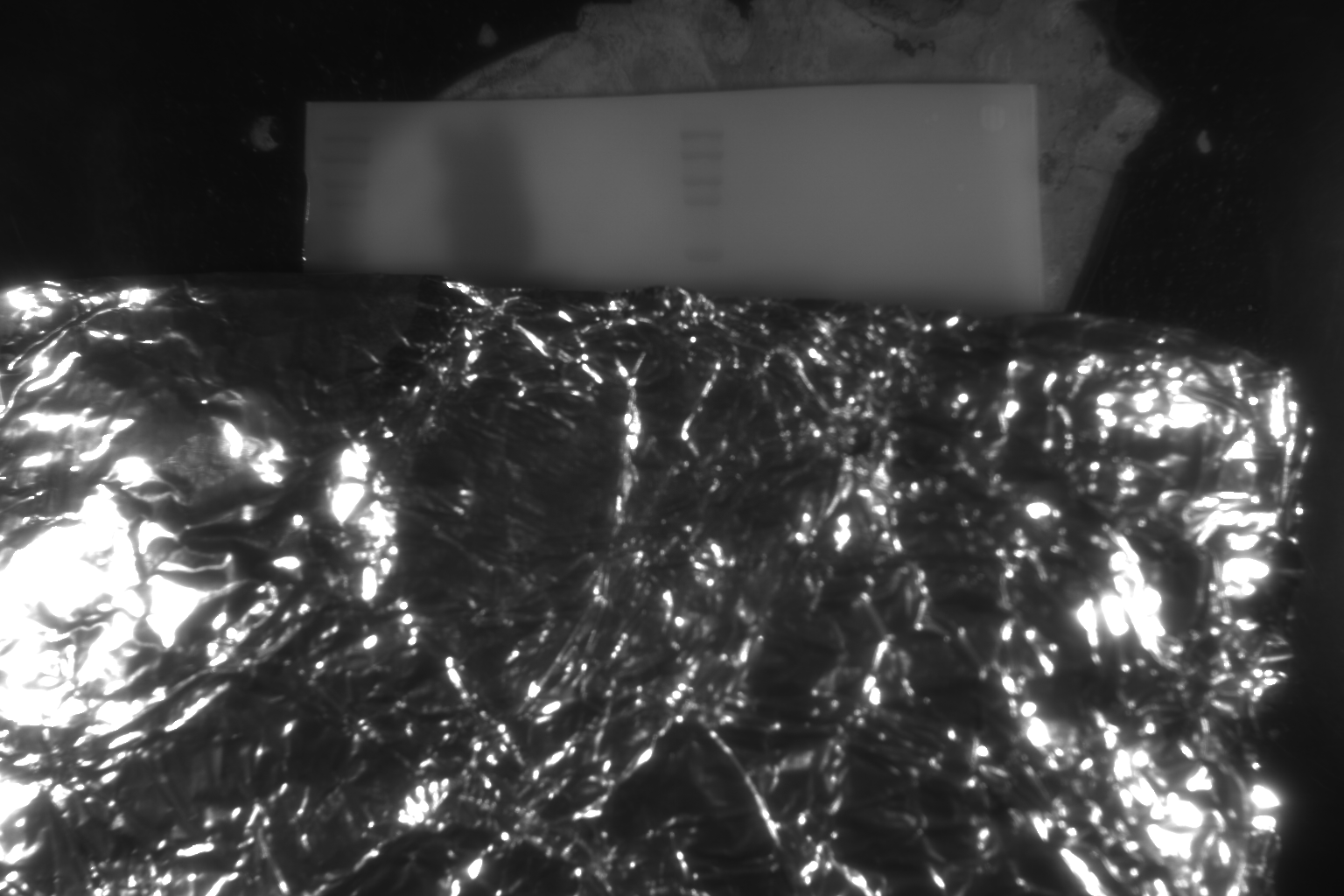

Supplement: Supplementary file 8 — Source data Fig. 6 [file 44318_2025_572_MOESM8_ESM.zip › EMBOJ-2025-121095R_Source Data for Figure 6/Figure 6/Figure 6F/V_Input MARCH8 4 SEC.gel]

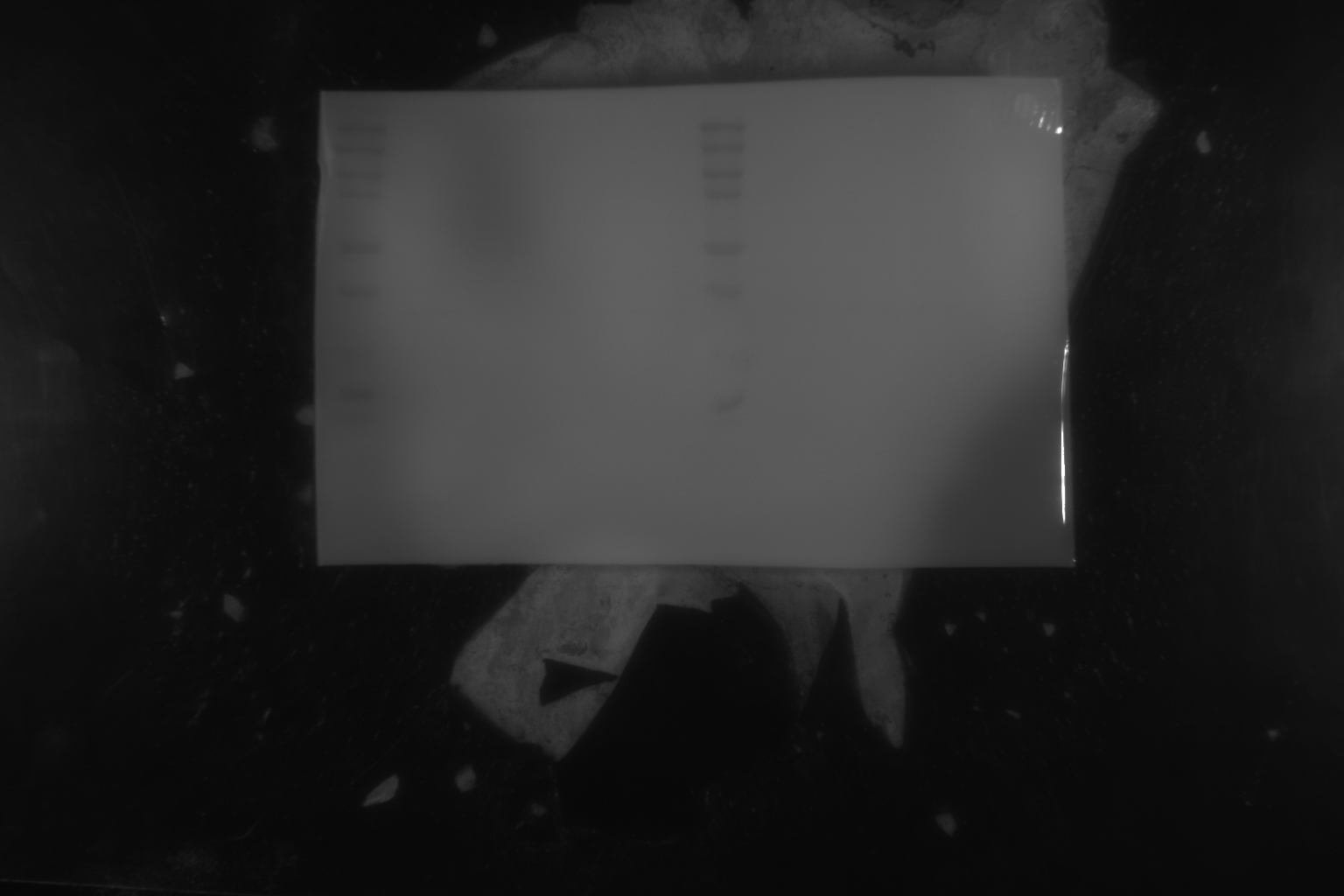

Supplement: Supplementary file 8 — Source data Fig. 6 [file 44318_2025_572_MOESM8_ESM.zip › EMBOJ-2025-121095R_Source Data for Figure 6/Figure 6/Figure 6F/V_Input MARCH8 8 SEC.gel]

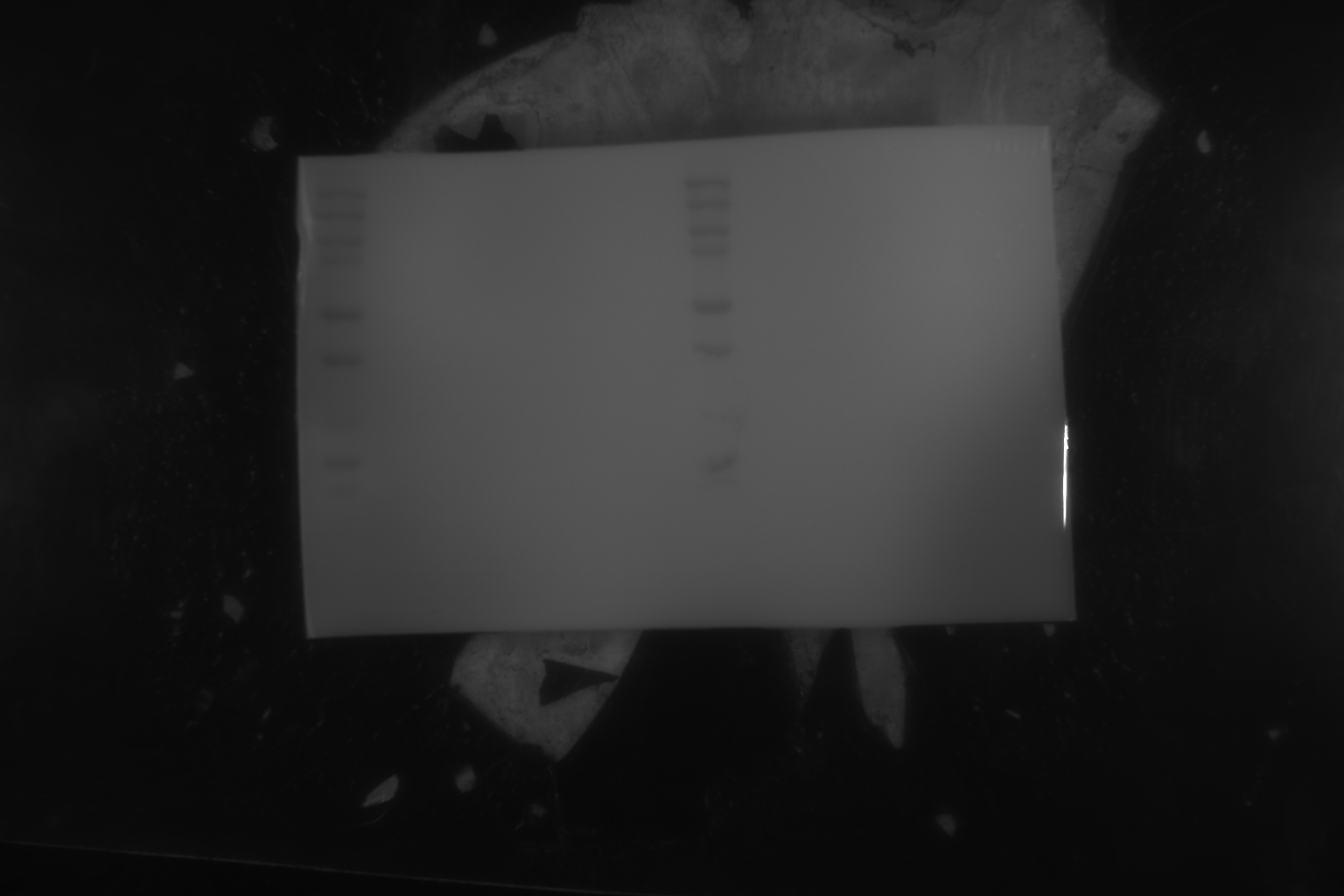

Supplement: Supplementary file 8 — Source data Fig. 6 [file 44318_2025_572_MOESM8_ESM.zip › EMBOJ-2025-121095R_Source Data for Figure 6/Figure 6/Figure 6F/V_Input Orai3 15 sec.gel]

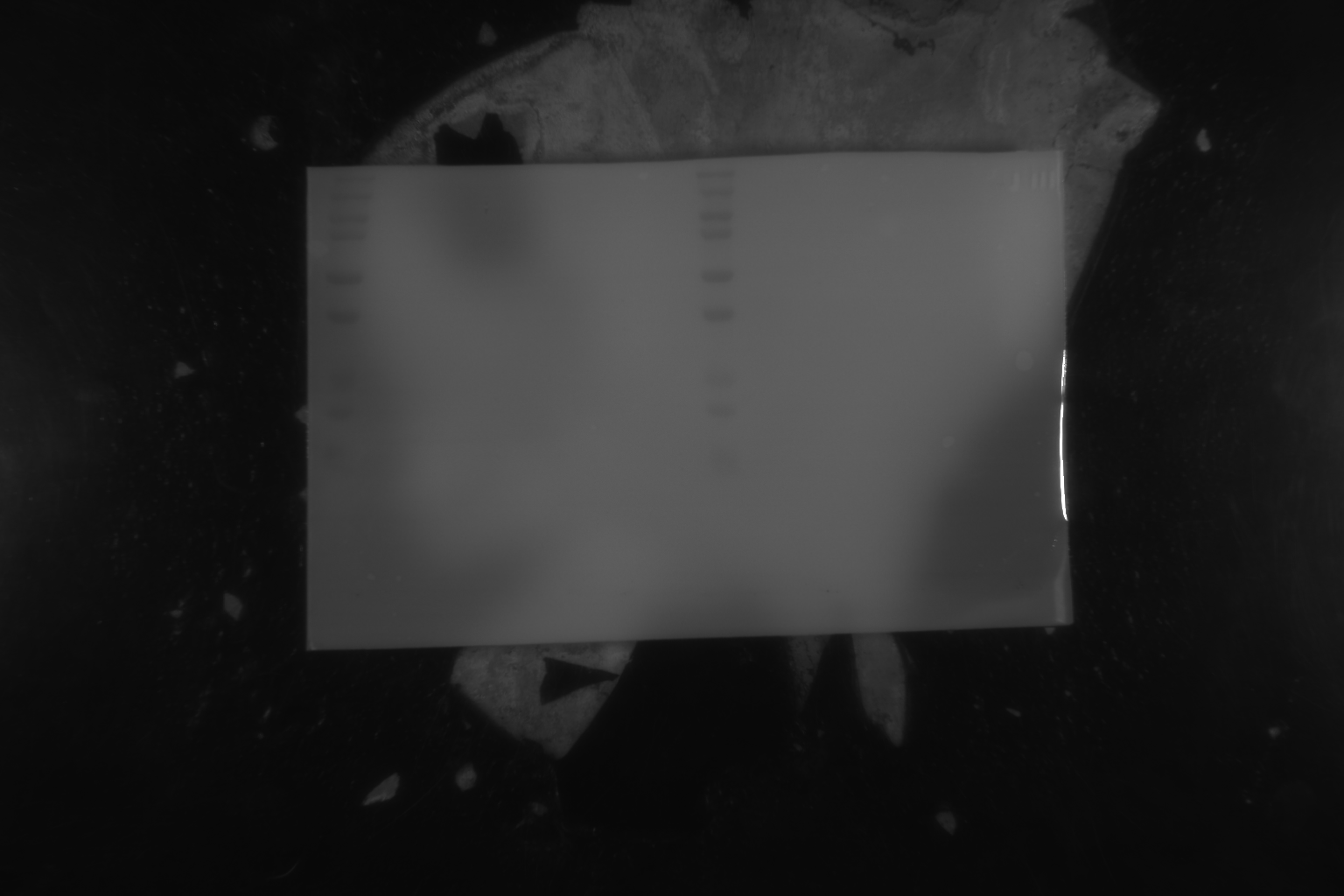

Supplement: Supplementary file 8 — Source data Fig. 6 [file 44318_2025_572_MOESM8_ESM.zip › EMBOJ-2025-121095R_Source Data for Figure 6/Figure 6/Figure 6F/V_MARCH 8 8 SEC.gel]

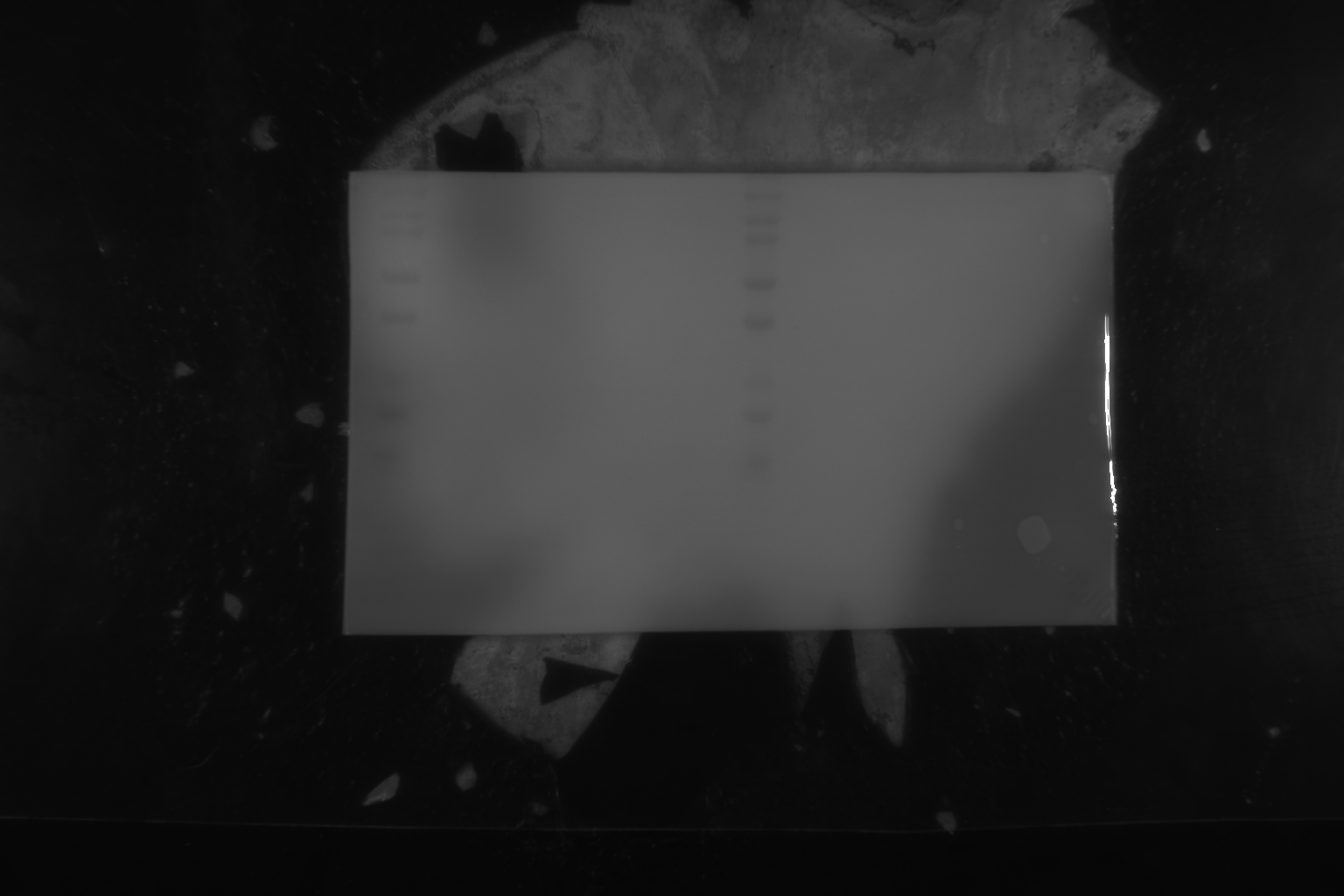

Supplement: Supplementary file 8 — Source data Fig. 6 [file 44318_2025_572_MOESM8_ESM.zip › EMBOJ-2025-121095R_Source Data for Figure 6/Figure 6/Figure 6F/V_Orai3 60 SEC.gel]

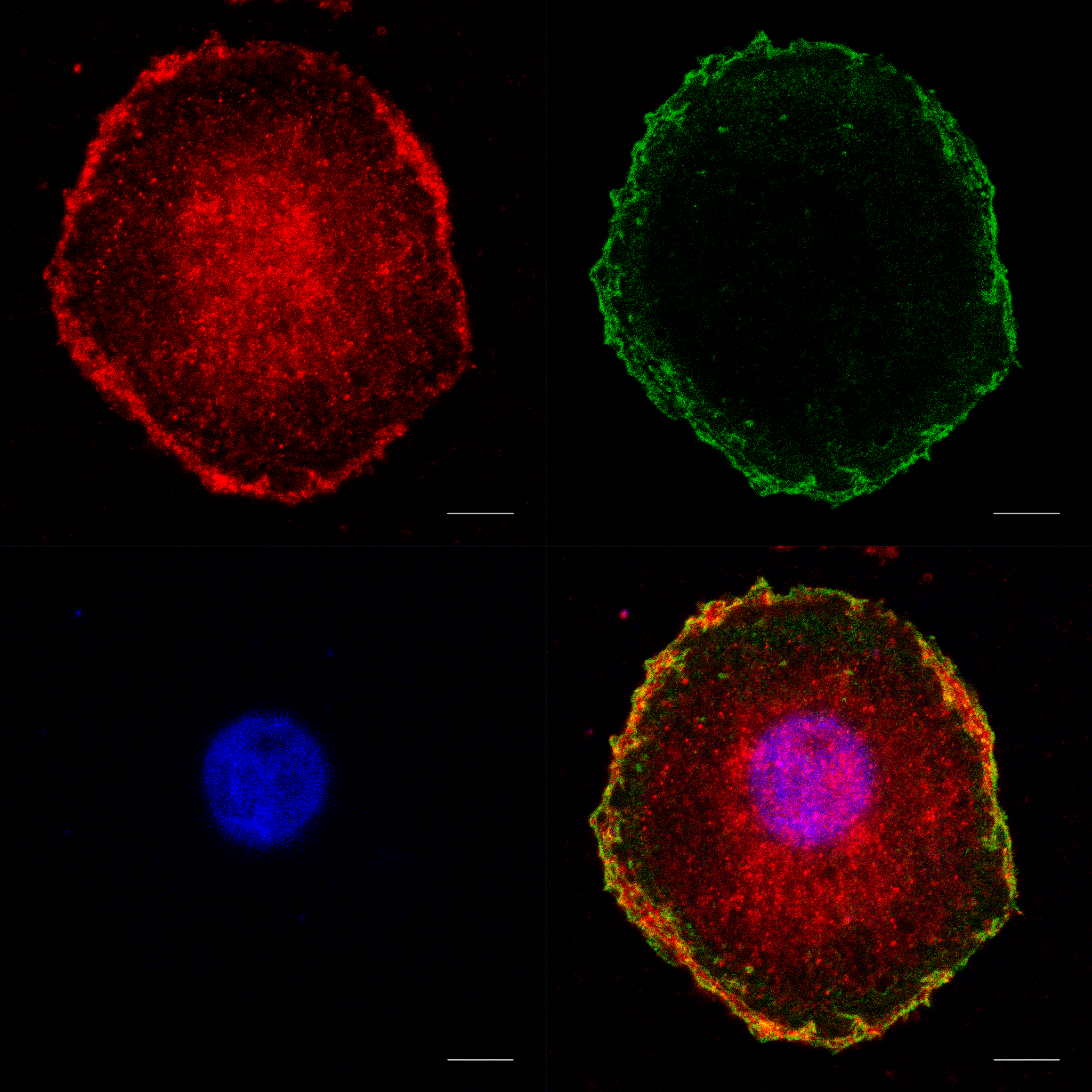

Supplement: Supplementary file 8 — Source data Fig. 6 [file 44318_2025_572_MOESM8_ESM.zip › EMBOJ-2025-121095R_Source Data for Figure 6/Figure 6/Figure 6G/Colocalization.tif]

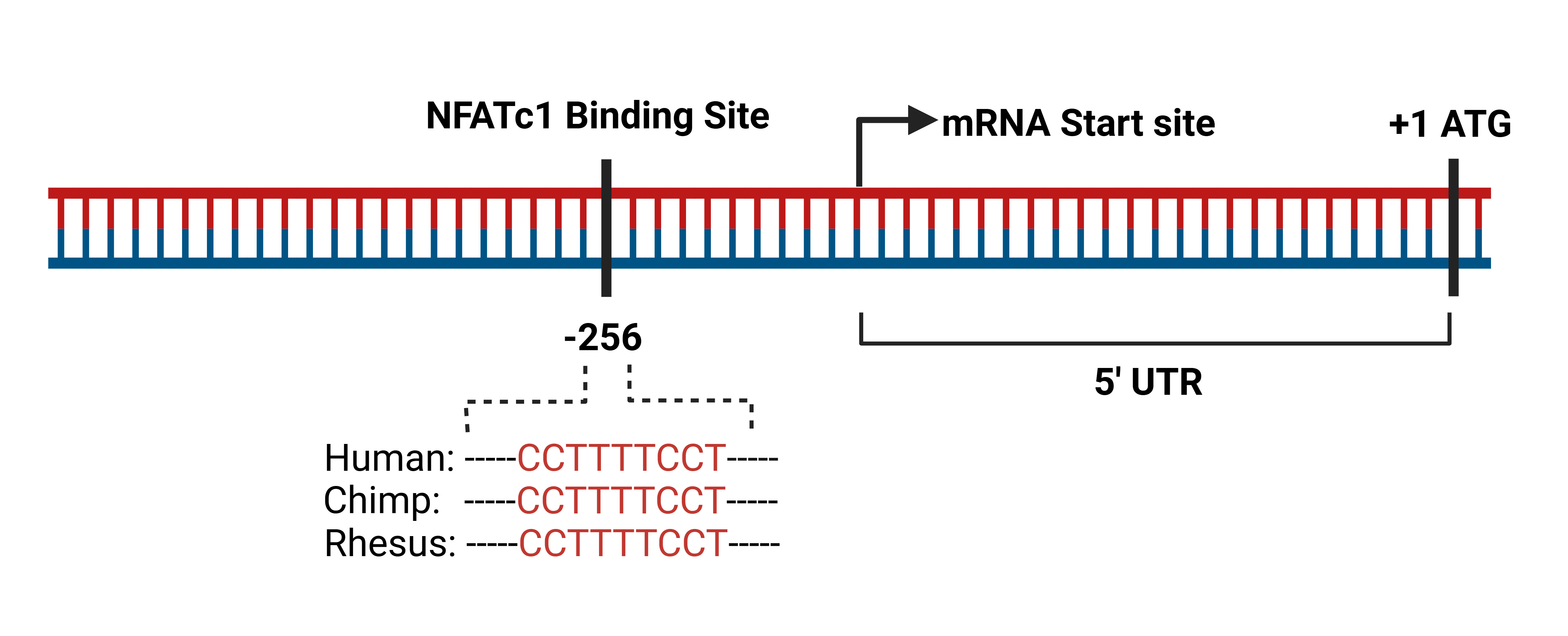

Supplement: Supplementary file 9 — Source data Fig. 7 [file 44318_2025_572_MOESM9_ESM.zip › Figure 7/Figure 7A/M8 Promoter.png]

p-value =  $4.2\text{e-}15$

R = 0.4

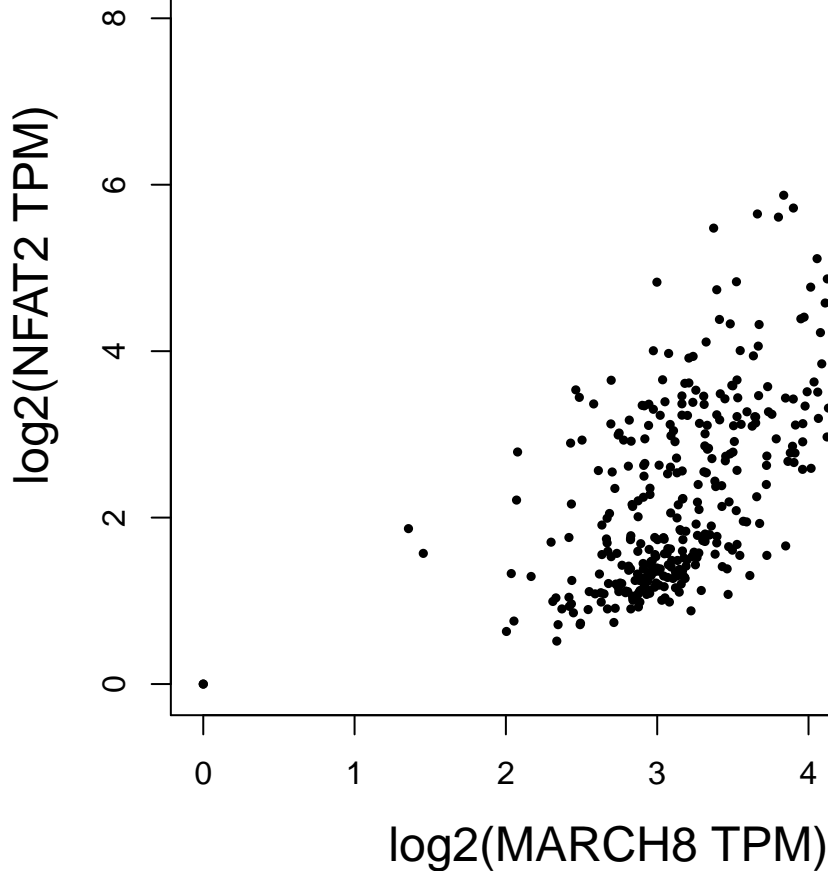

Supplement: Supplementary file 9 — Source data Fig. 7 [file 44318_2025_572_MOESM9_ESM.zip › Figure 7/Figure 7B/MARCH8 NFAT2 correlation.pdf]

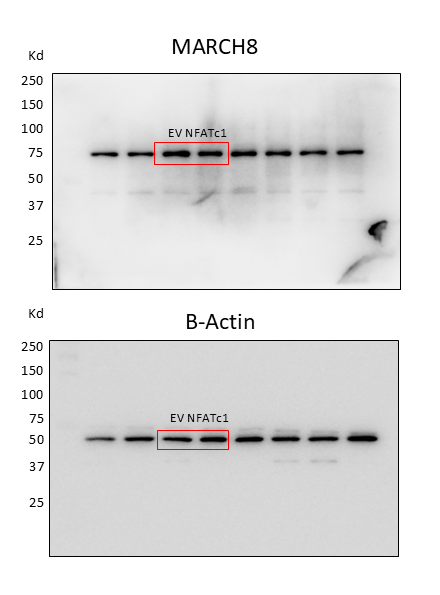

Supplement: Supplementary file 9 — Source data Fig. 7 [file 44318_2025_572_MOESM9_ESM.zip › Figure 7/Figure 7F/Figure 7F.png]

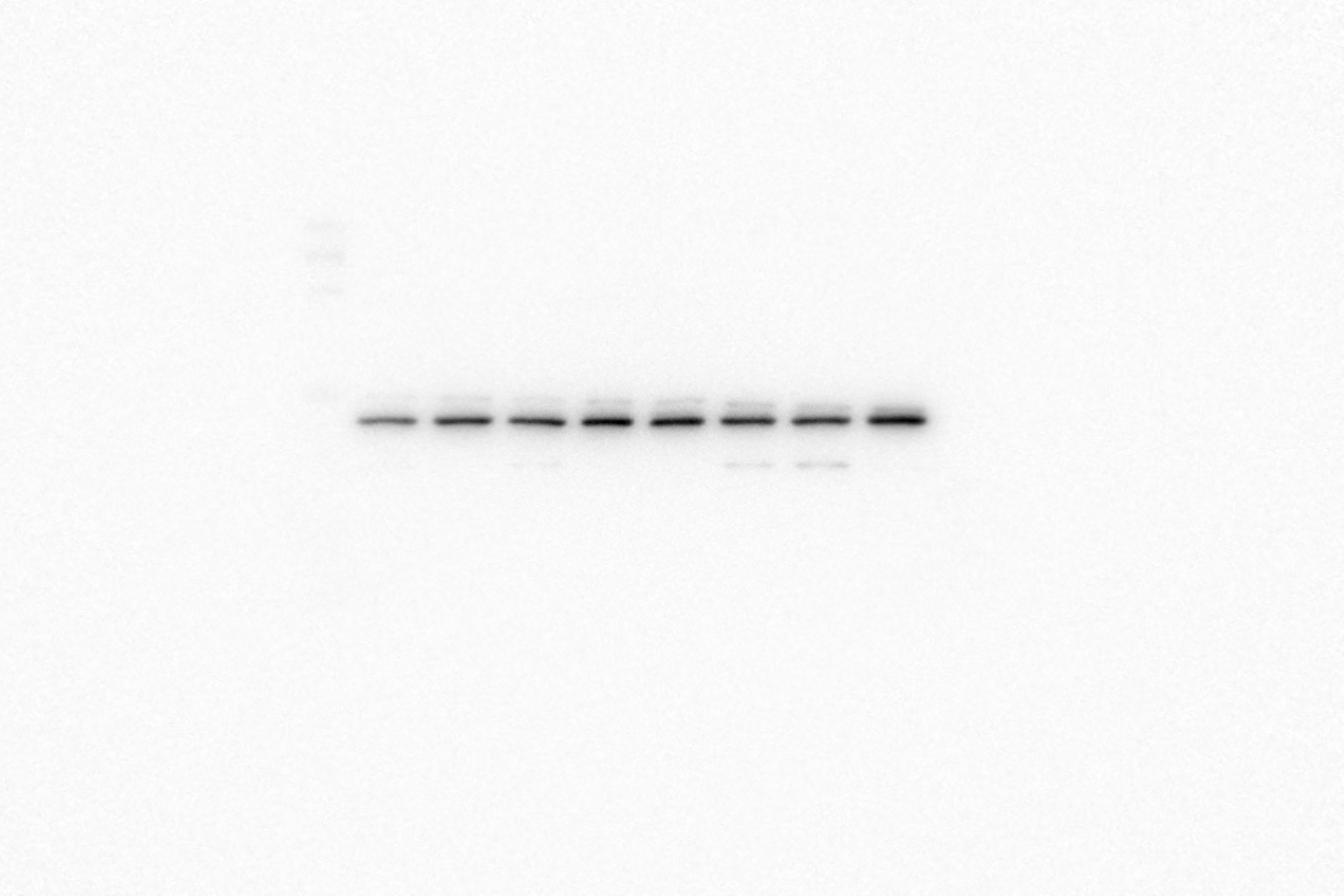

Supplement: Supplementary file 9 — Source data Fig. 7 [file 44318_2025_572_MOESM9_ESM.zip › Figure 7/Figure 7F/MIAPACA2 B ACTIN 0.5 SEC.gel]

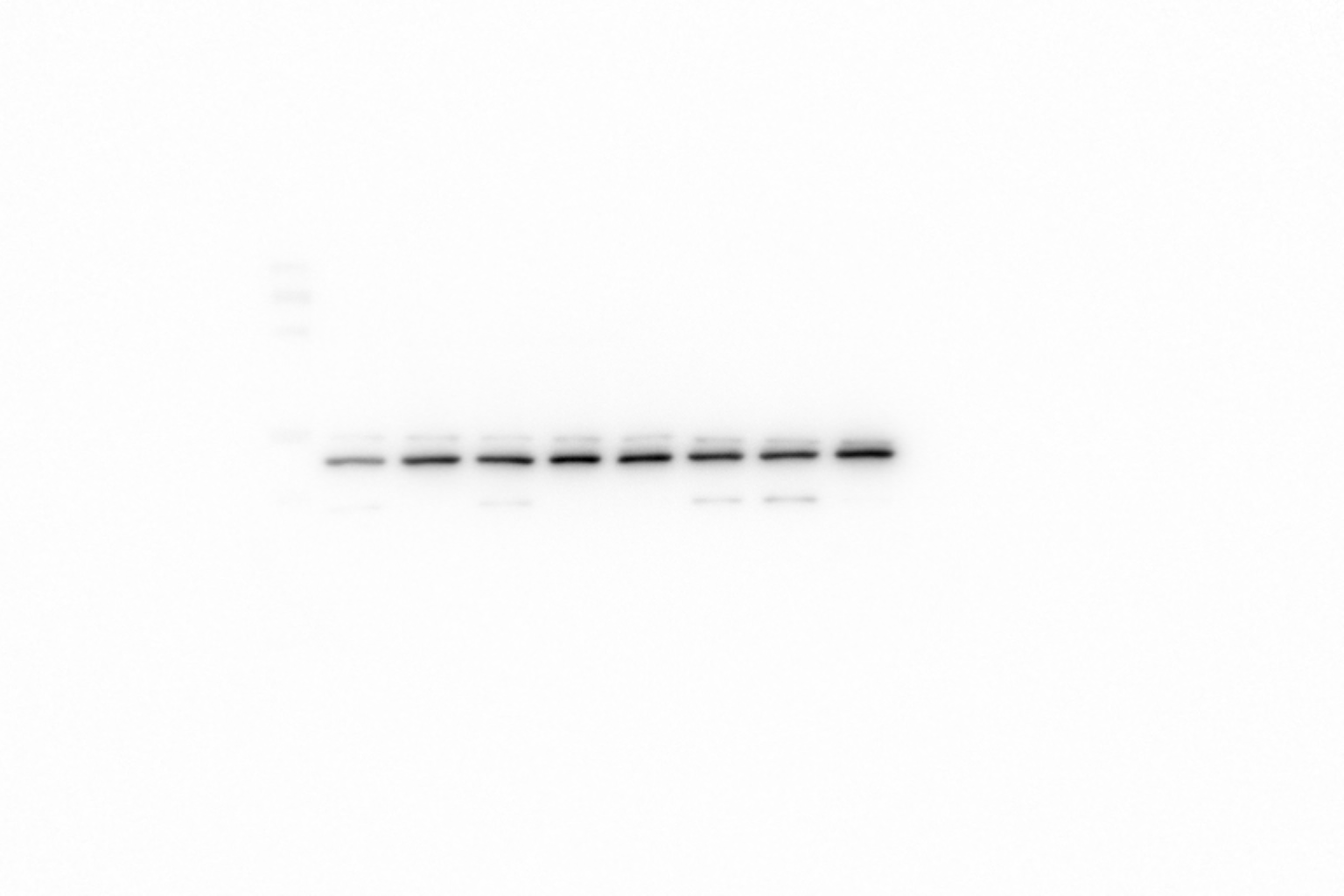

Supplement: Supplementary file 9 — Source data Fig. 7 [file 44318_2025_572_MOESM9_ESM.zip › Figure 7/Figure 7F/MIAPACA2 B ACTIN 1 SEC.gel]

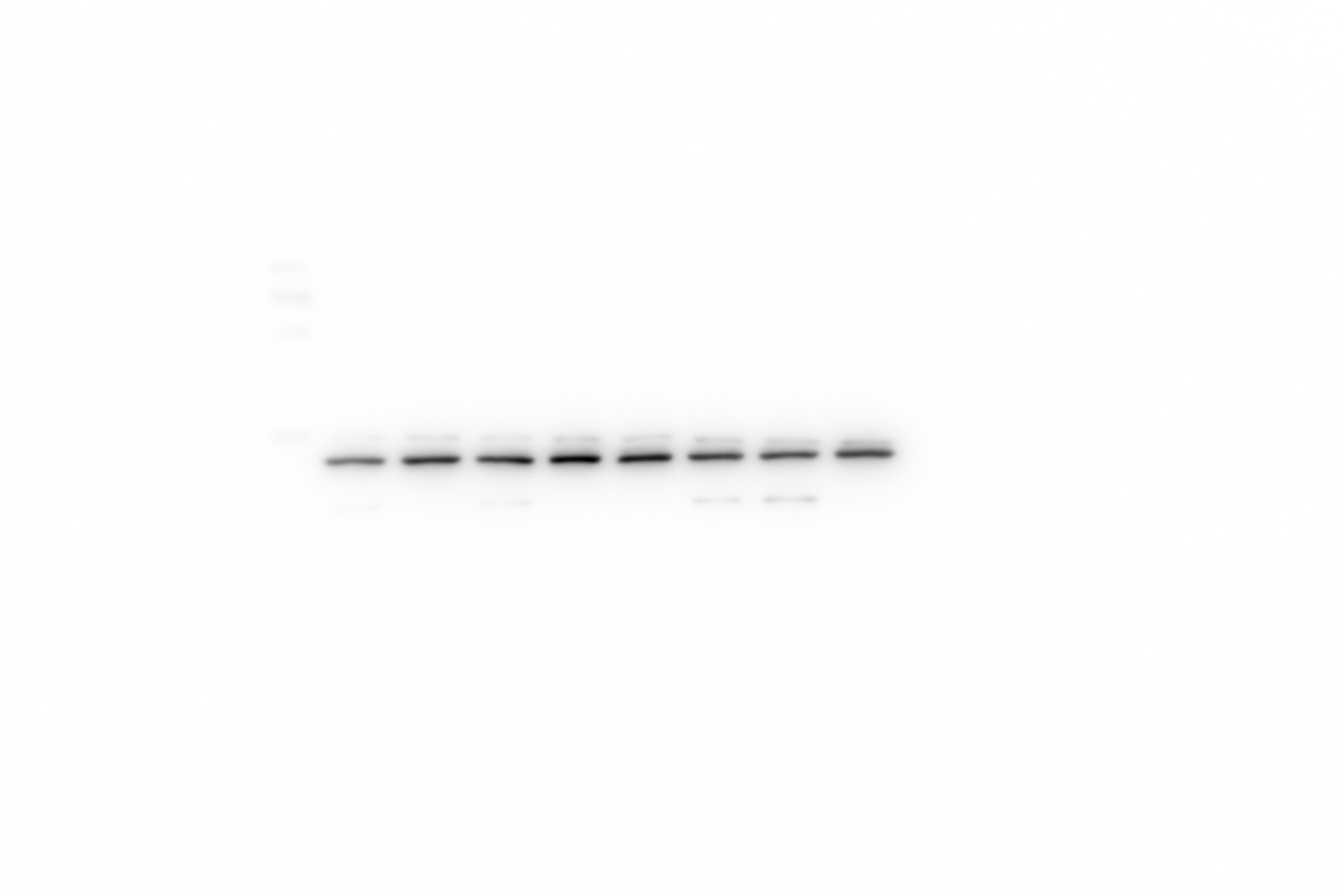

Supplement: Supplementary file 9 — Source data Fig. 7 [file 44318_2025_572_MOESM9_ESM.zip › Figure 7/Figure 7F/MIAPACA2 B ACTIN 2 SEC.gel]

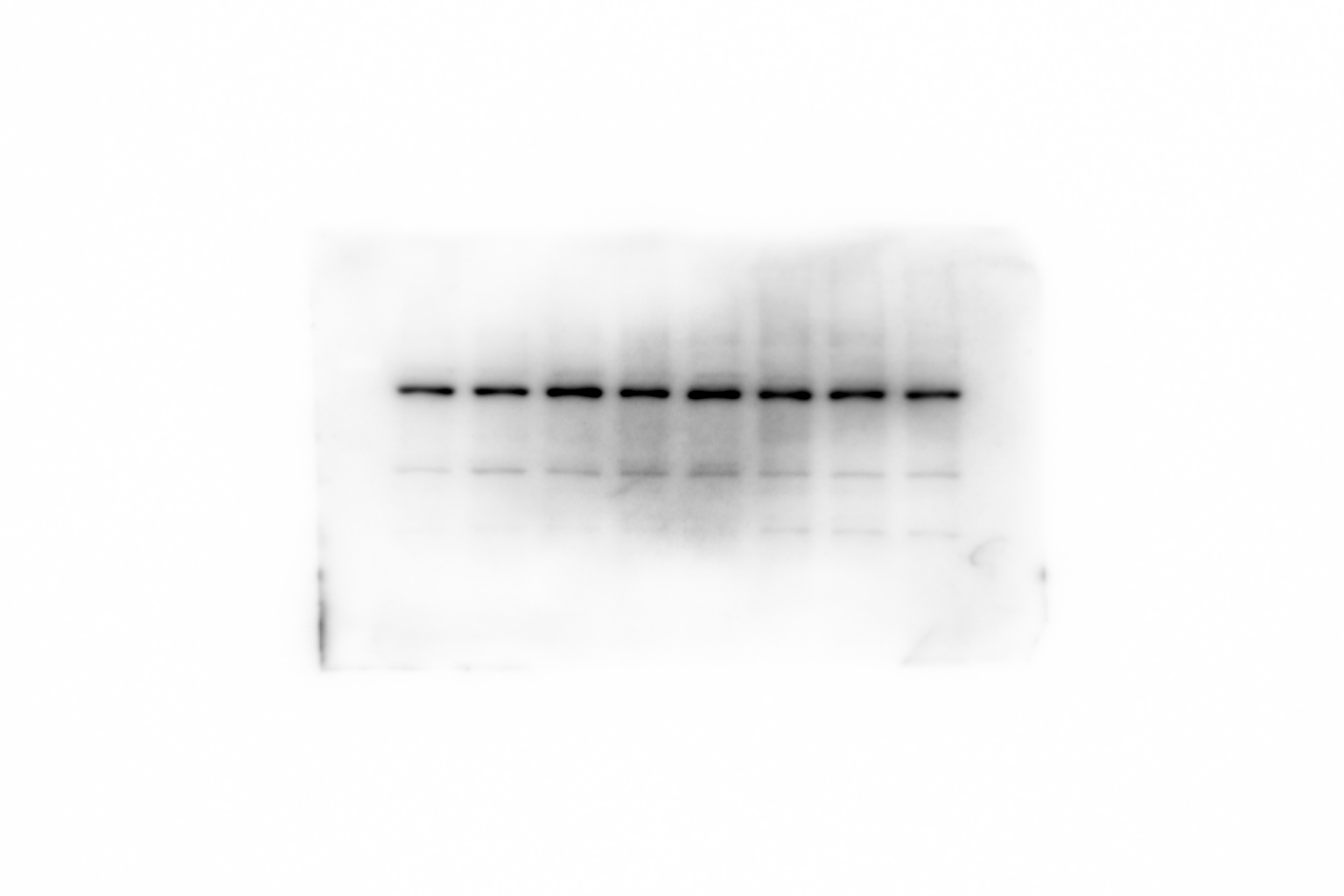

Supplement: Supplementary file 9 — Source data Fig. 7 [file 44318_2025_572_MOESM9_ESM.zip › Figure 7/Figure 7F/MIAPACA2 M8 120 SEC.gel]

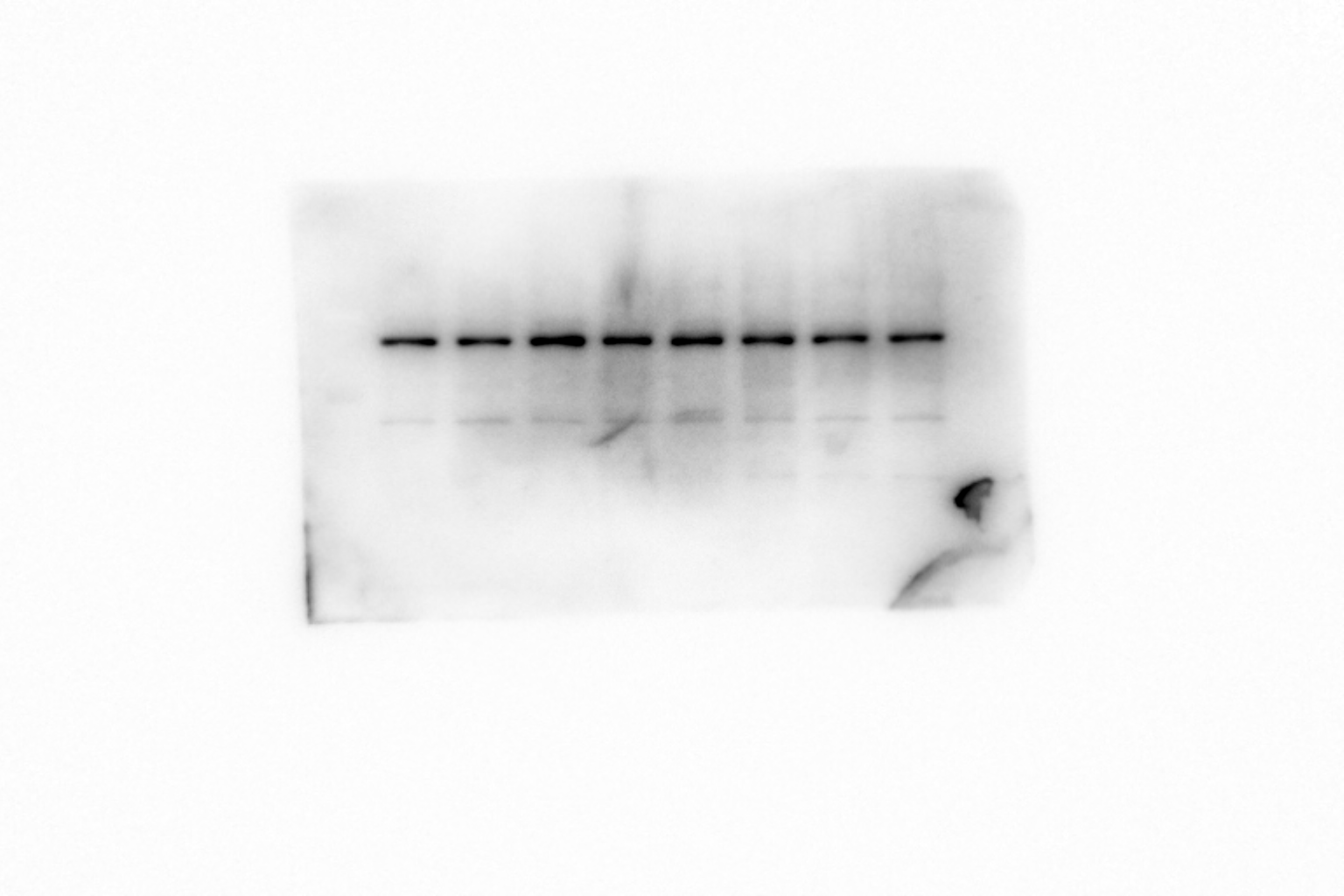

Supplement: Supplementary file 9 — Source data Fig. 7 [file 44318_2025_572_MOESM9_ESM.zip › Figure 7/Figure 7F/MIAPACA2 M8 30 SEC.gel]

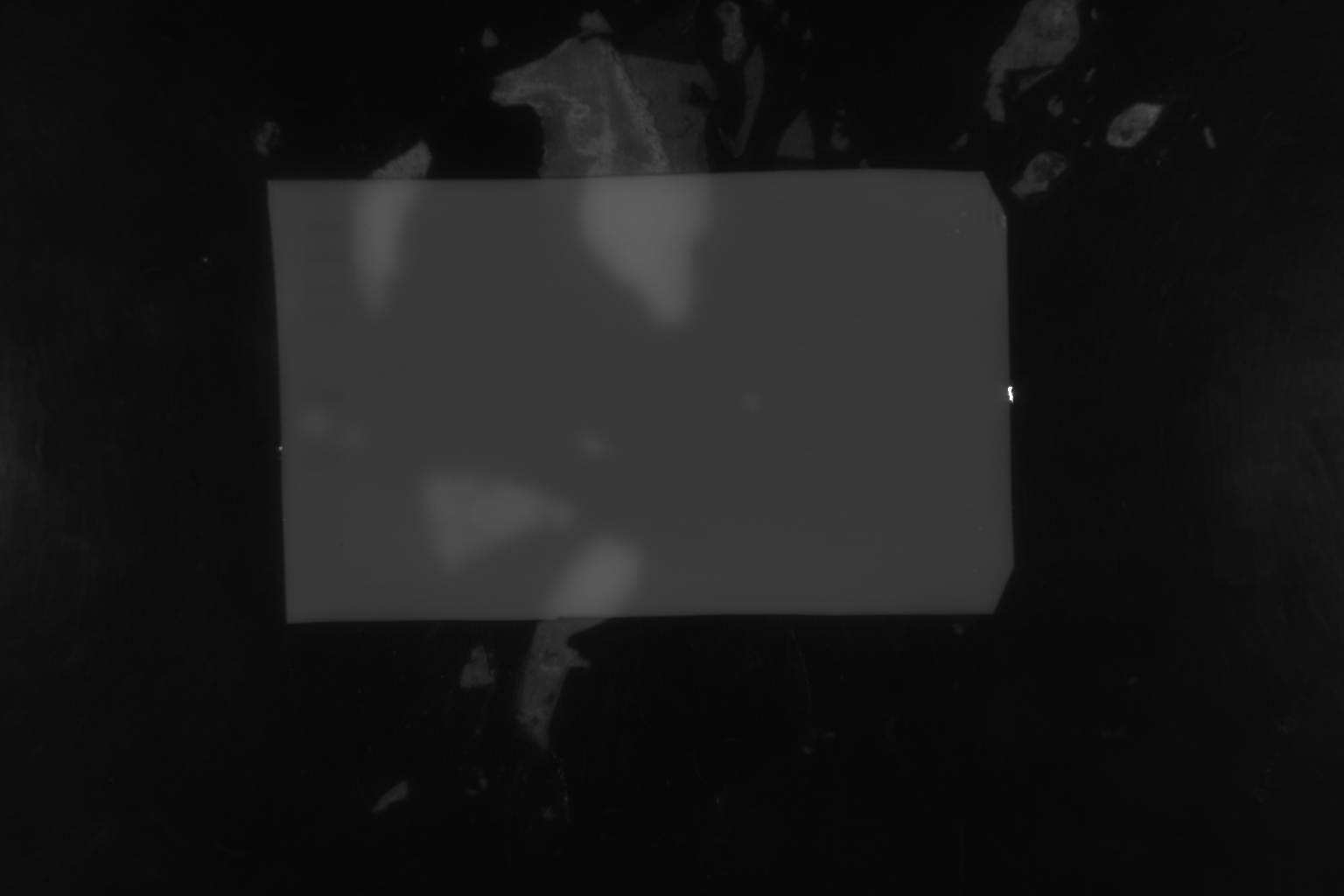

Supplement: Supplementary file 9 — Source data Fig. 7 [file 44318_2025_572_MOESM9_ESM.zip › Figure 7/Figure 7F/V_MIAPACA2 B ACTIN 0.5 SEC.gel]

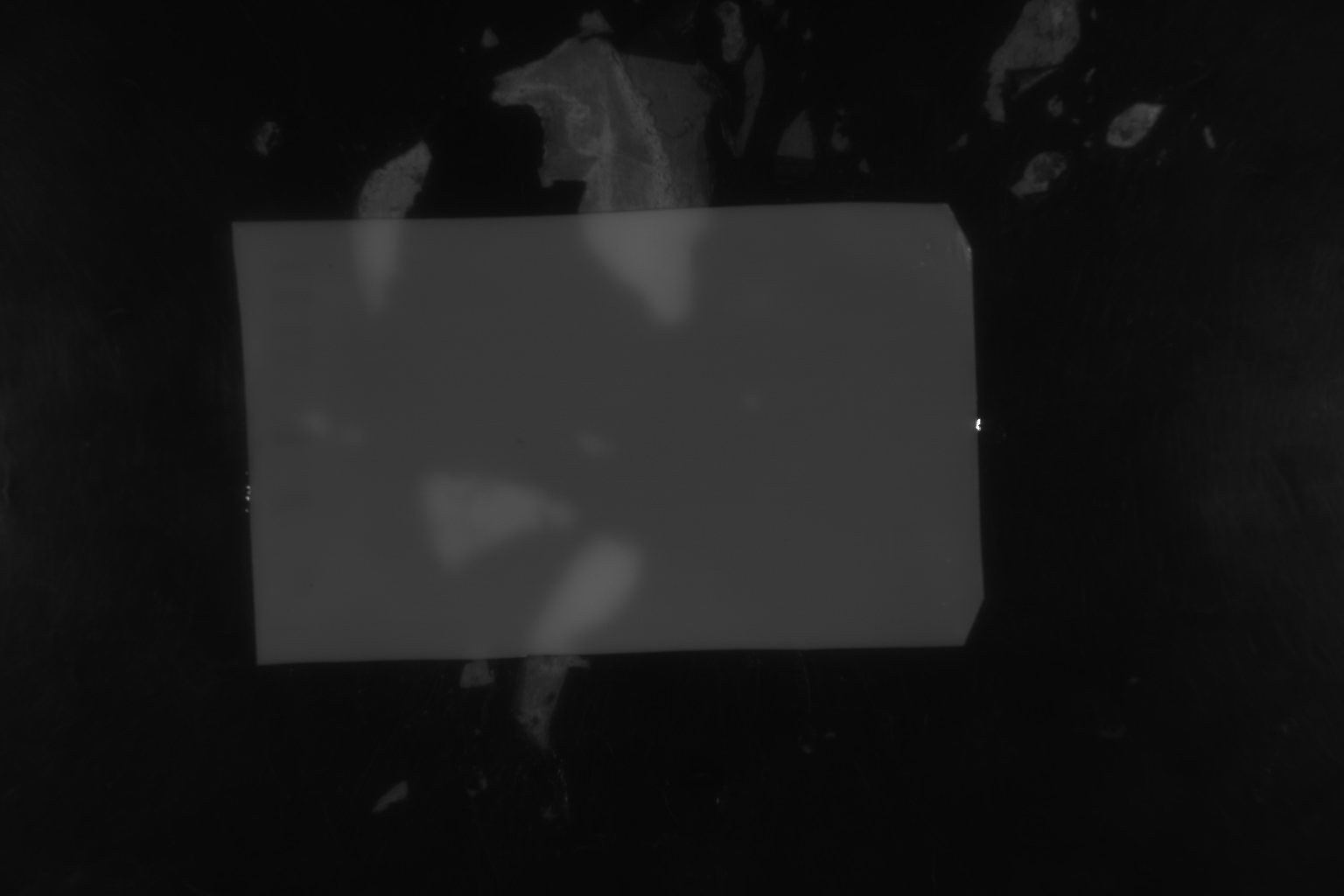

Supplement: Supplementary file 9 — Source data Fig. 7 [file 44318_2025_572_MOESM9_ESM.zip › Figure 7/Figure 7F/V_MIAPACA2 B ACTIN 1 SEC.gel]

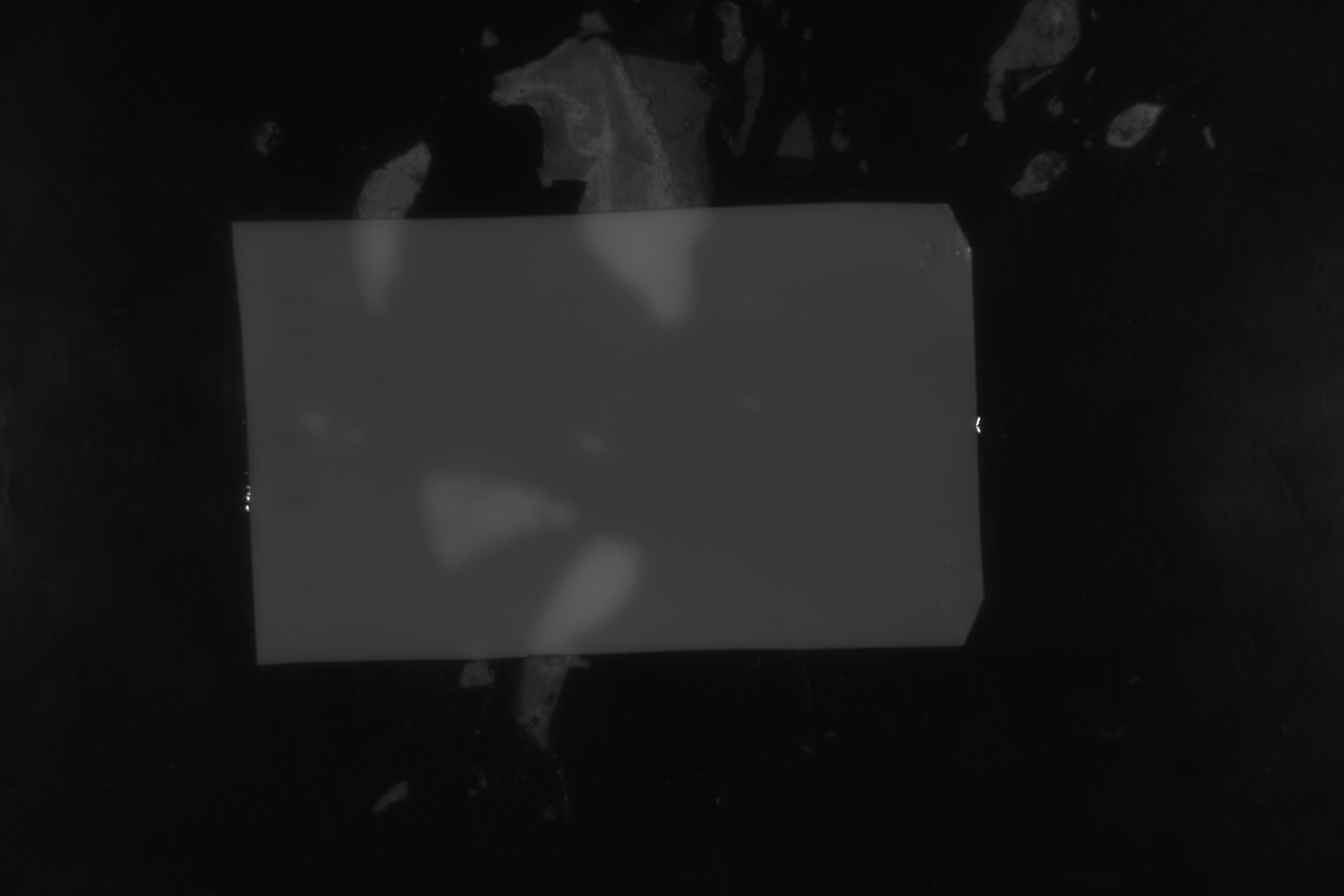

Supplement: Supplementary file 9 — Source data Fig. 7 [file 44318_2025_572_MOESM9_ESM.zip › Figure 7/Figure 7F/V_MIAPACA2 B ACTIN 2 SEC.gel]

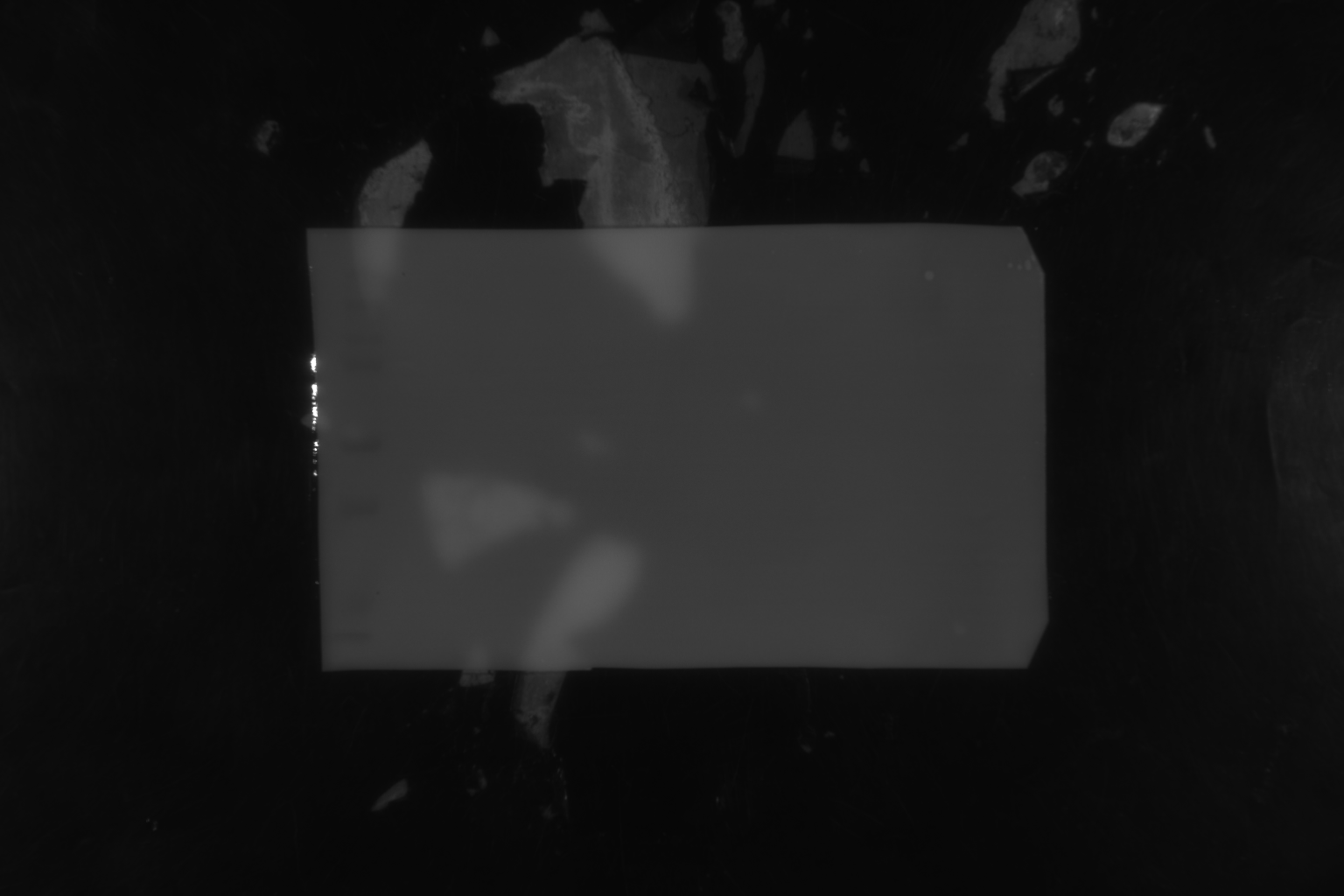

Supplement: Supplementary file 9 — Source data Fig. 7 [file 44318_2025_572_MOESM9_ESM.zip › Figure 7/Figure 7F/V_MIAPACA2 M8 120 SEC.gel]

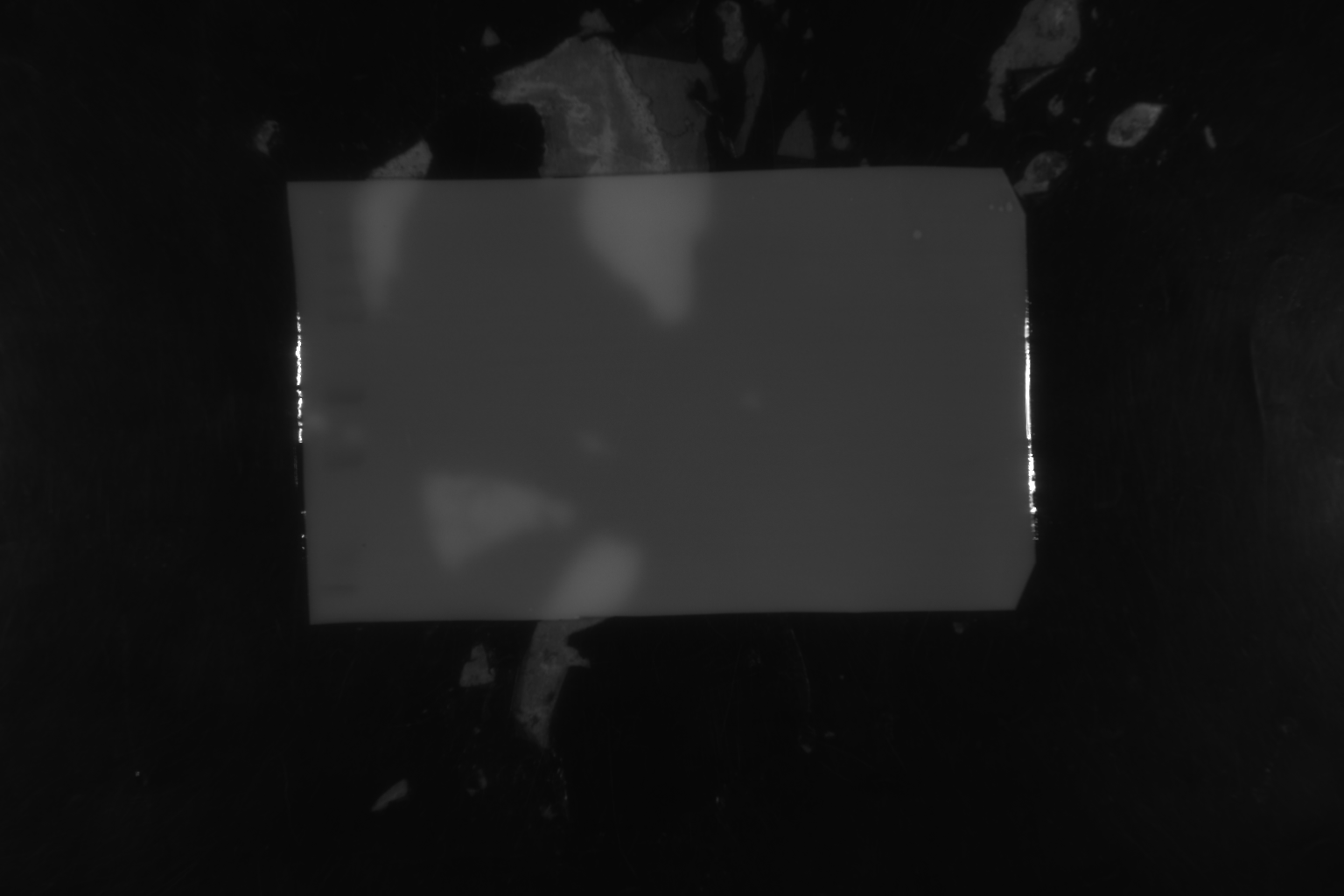

Supplement: Supplementary file 9 — Source data Fig. 7 [file 44318_2025_572_MOESM9_ESM.zip › Figure 7/Figure 7F/V_MIAPACA2 M8 30 SEC.gel]

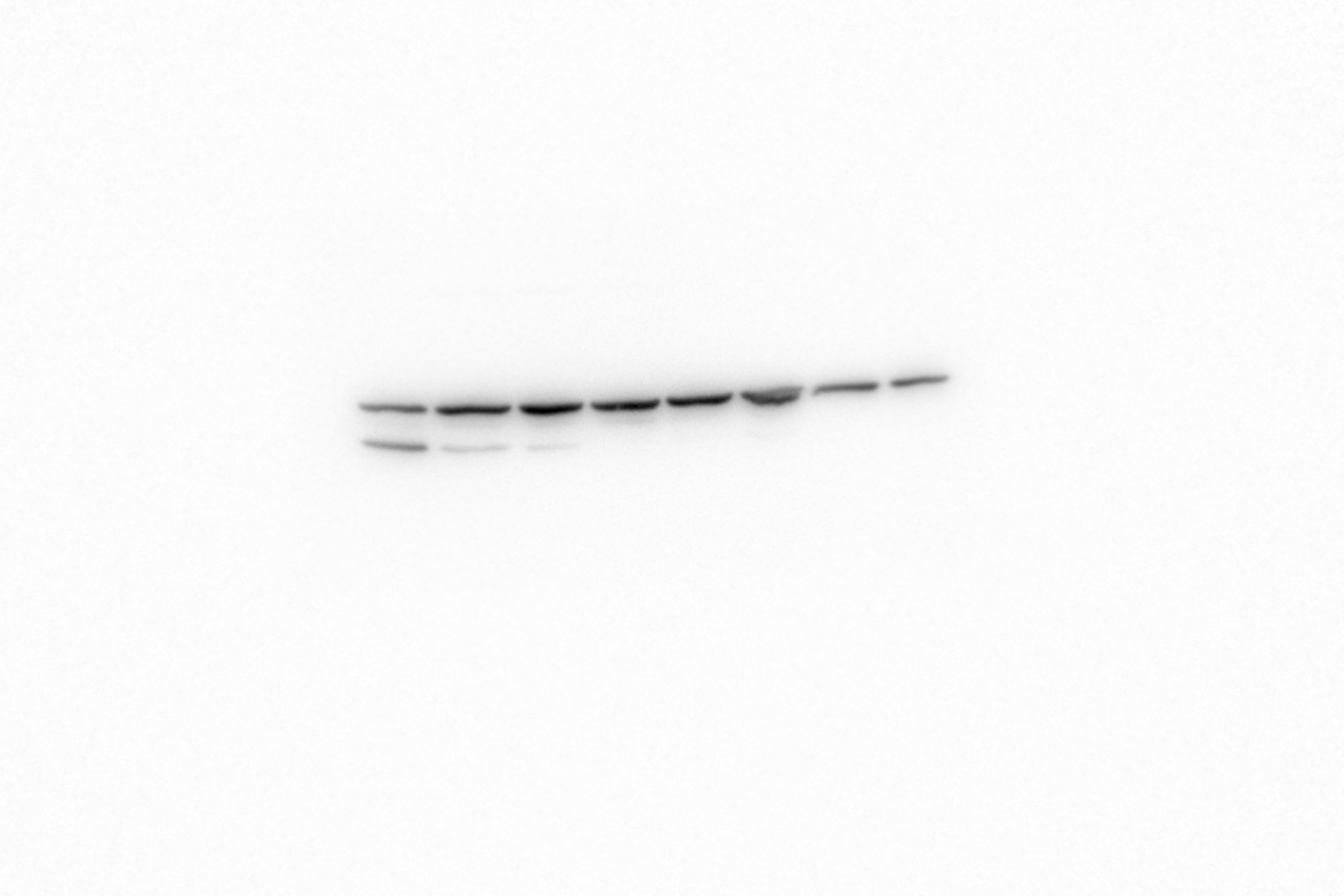

Supplement: Supplementary file 9 — Source data Fig. 7 [file 44318_2025_572_MOESM9_ESM.zip › Figure 7/Figure 7I/B ACTIN 0.5 SEC.gel]

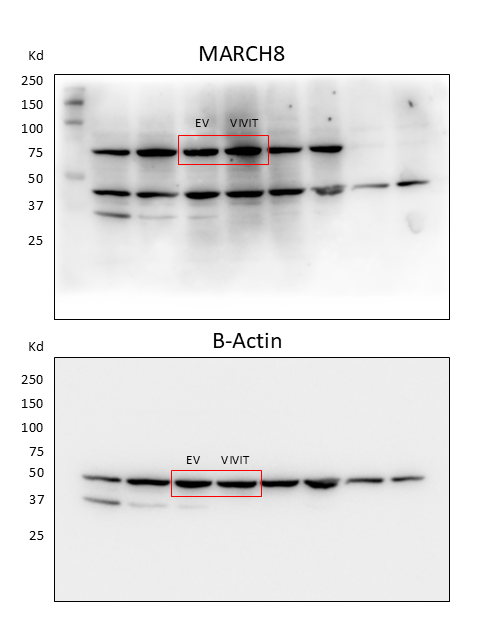

Supplement: Supplementary file 9 — Source data Fig. 7 [file 44318_2025_572_MOESM9_ESM.zip › Figure 7/Figure 7I/Figure 7I.png]

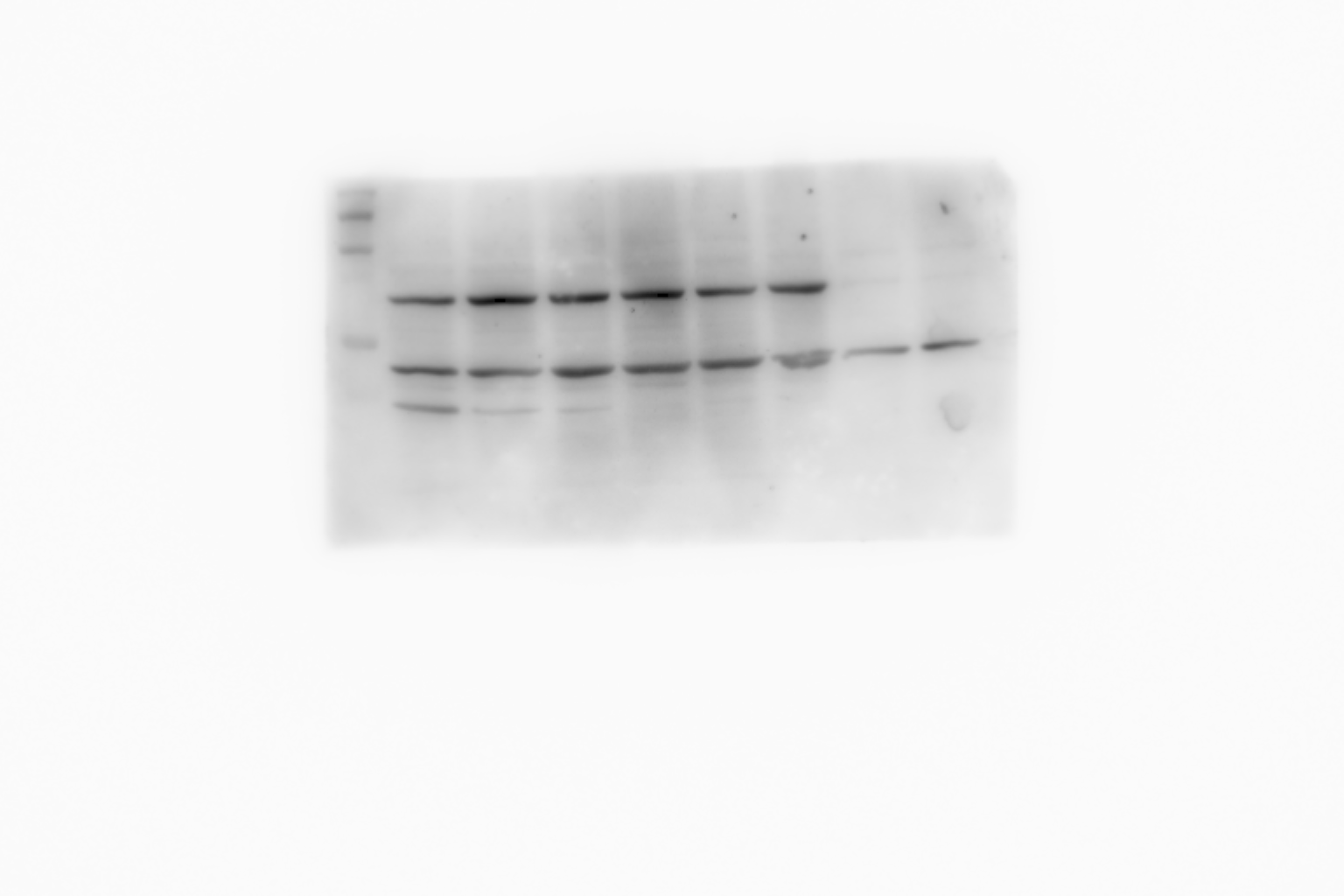

Supplement: Supplementary file 9 — Source data Fig. 7 [file 44318_2025_572_MOESM9_ESM.zip › Figure 7/Figure 7I/M8 3 MIN.gel]

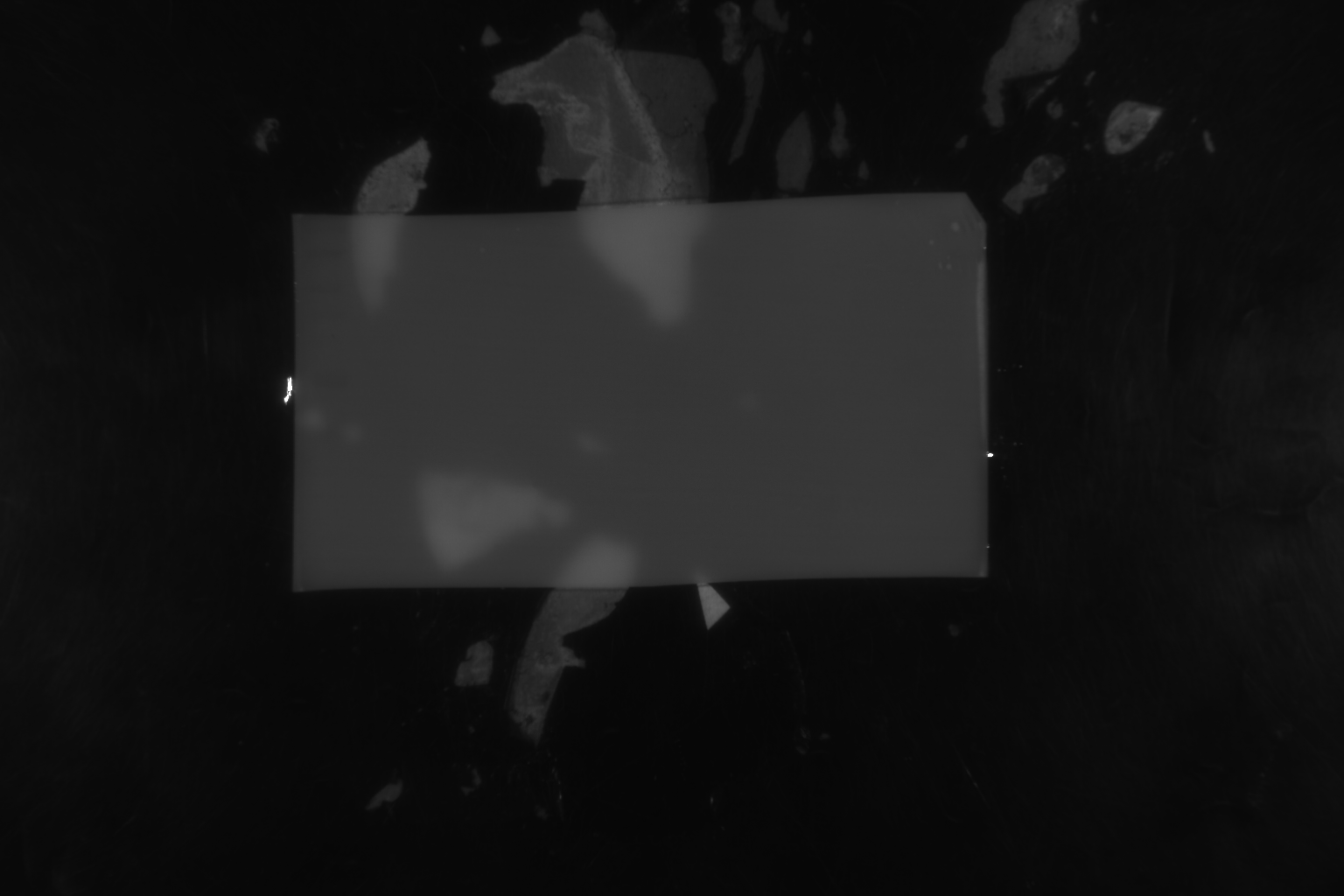

Supplement: Supplementary file 9 — Source data Fig. 7 [file 44318_2025_572_MOESM9_ESM.zip › Figure 7/Figure 7I/V_B ACTIN 0.5 SEC.gel]

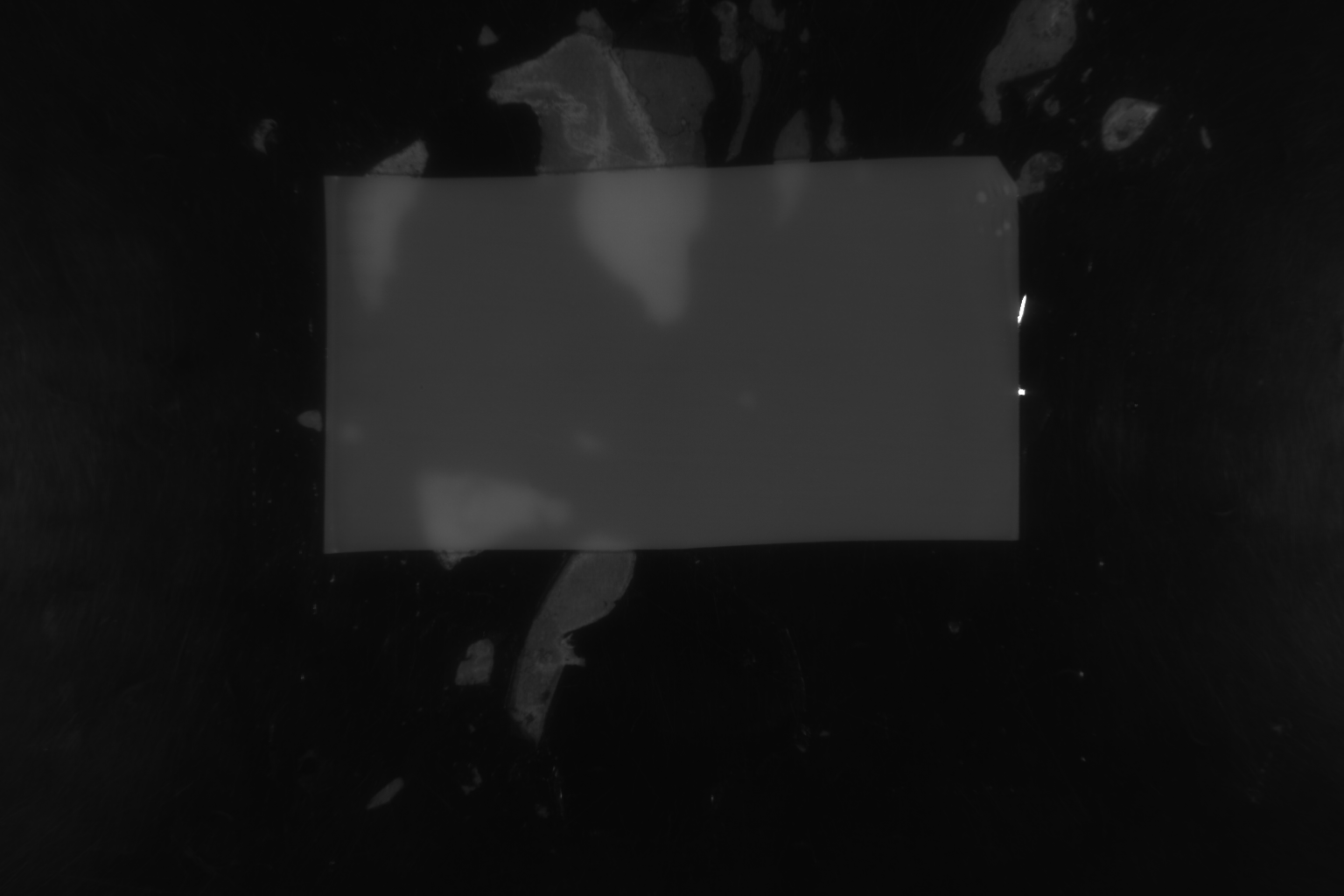

Supplement: Supplementary file 9 — Source data Fig. 7 [file 44318_2025_572_MOESM9_ESM.zip › Figure 7/Figure 7I/V_M8 3 MIN.gel]

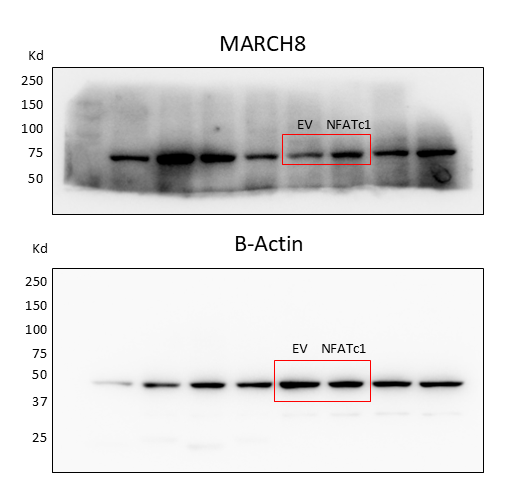

Supplement: Supplementary file 9 — Source data Fig. 7 [file 44318_2025_572_MOESM9_ESM.zip › Figure 7/Figure 7L/Figure 7L.png]

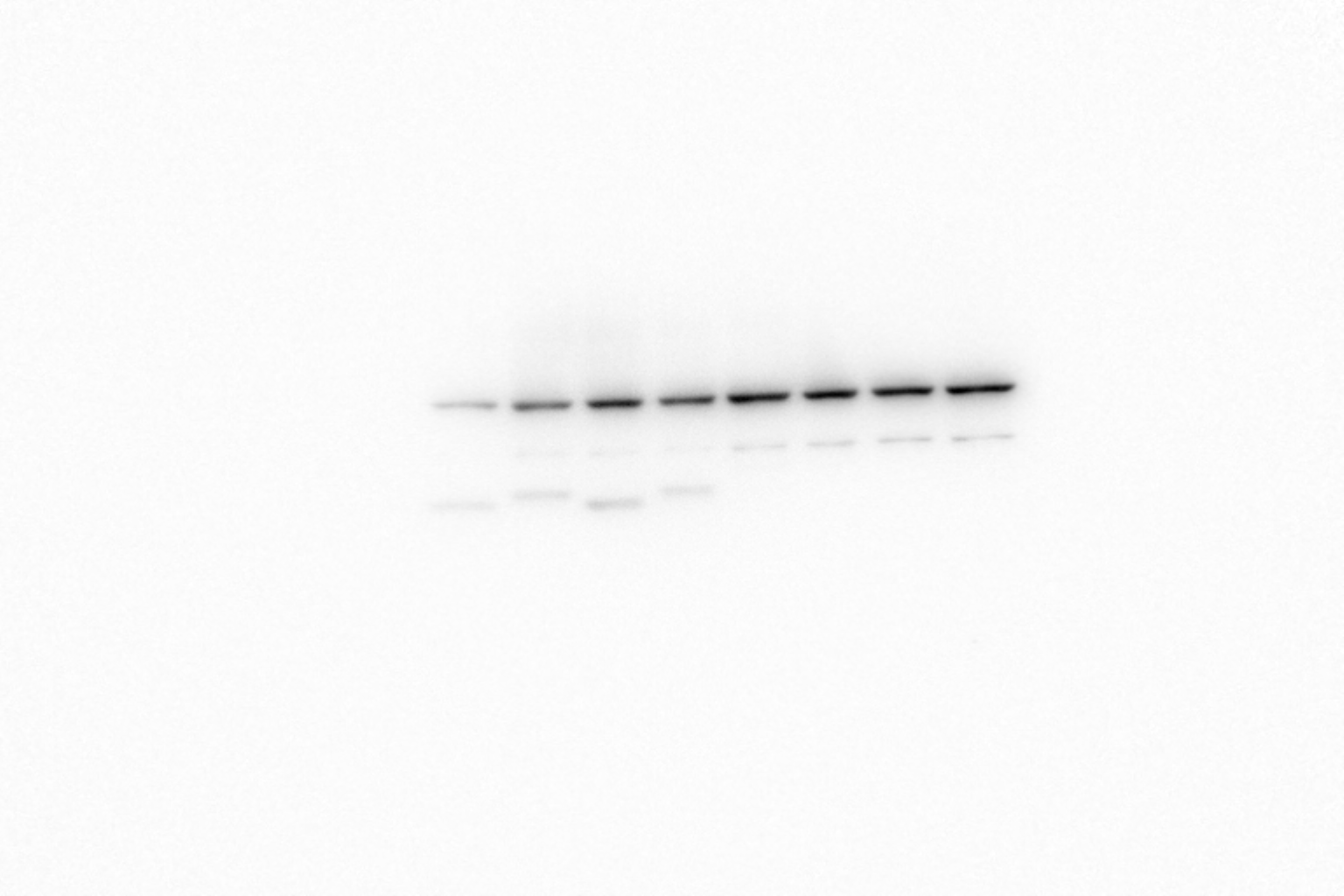

Supplement: Supplementary file 9 — Source data Fig. 7 [file 44318_2025_572_MOESM9_ESM.zip › Figure 7/Figure 7L/VIVIT B ACTIN 1 SEC.gel]

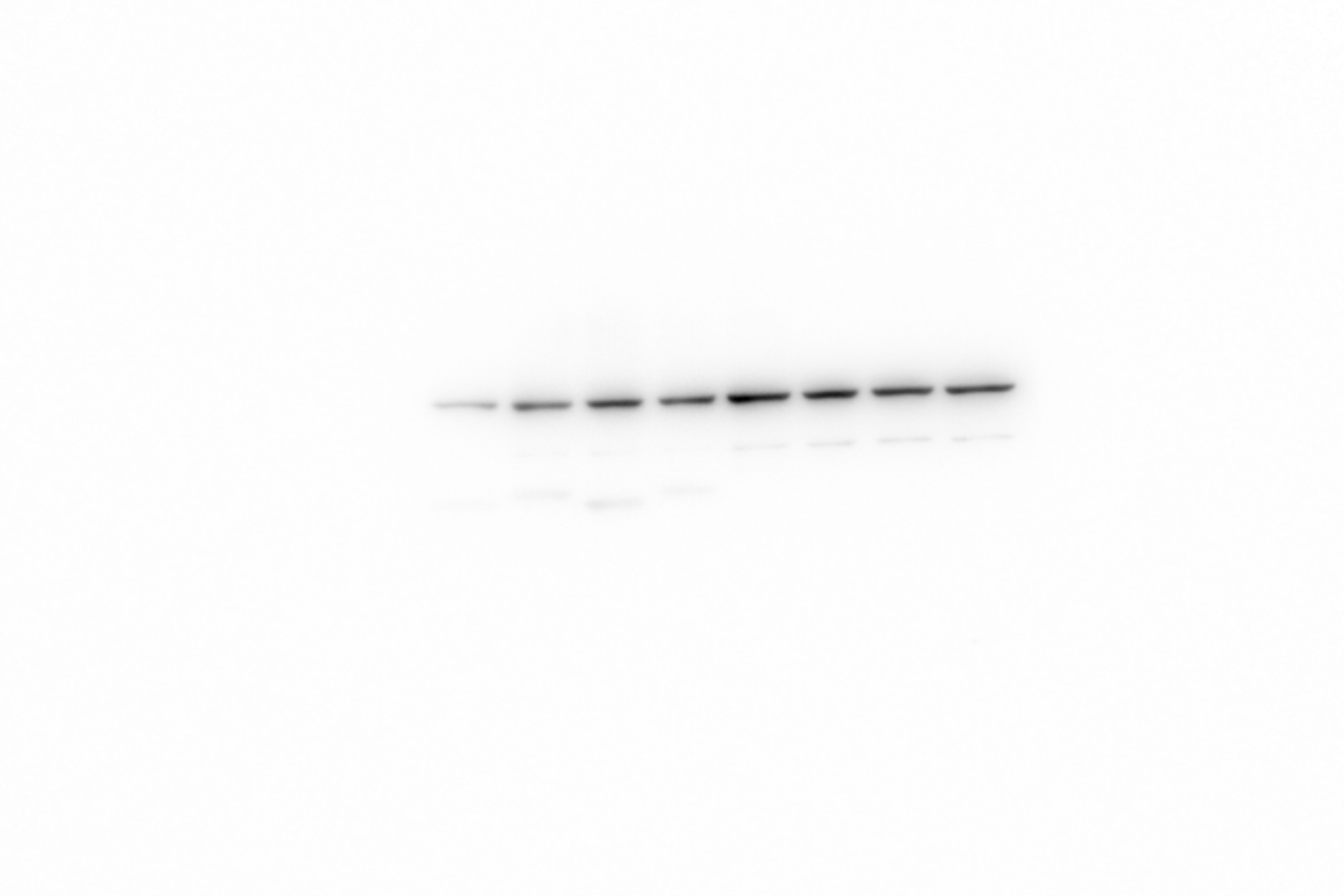

Supplement: Supplementary file 9 — Source data Fig. 7 [file 44318_2025_572_MOESM9_ESM.zip › Figure 7/Figure 7L/VIVIT B ACTIN 2 SEC.gel]

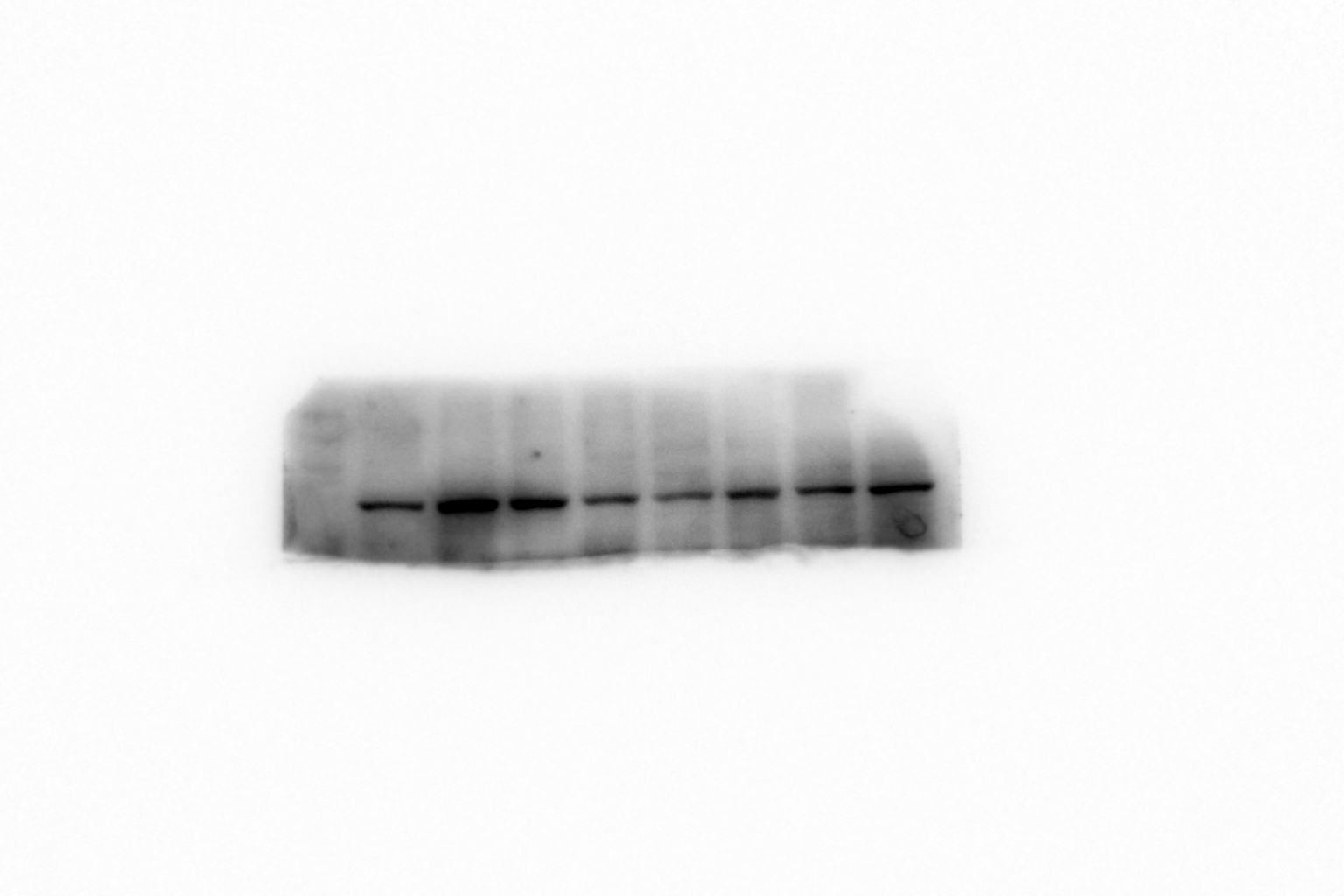

Supplement: Supplementary file 9 — Source data Fig. 7 [file 44318_2025_572_MOESM9_ESM.zip › Figure 7/Figure 7L/VIVIT M8 15 SEC.gel]

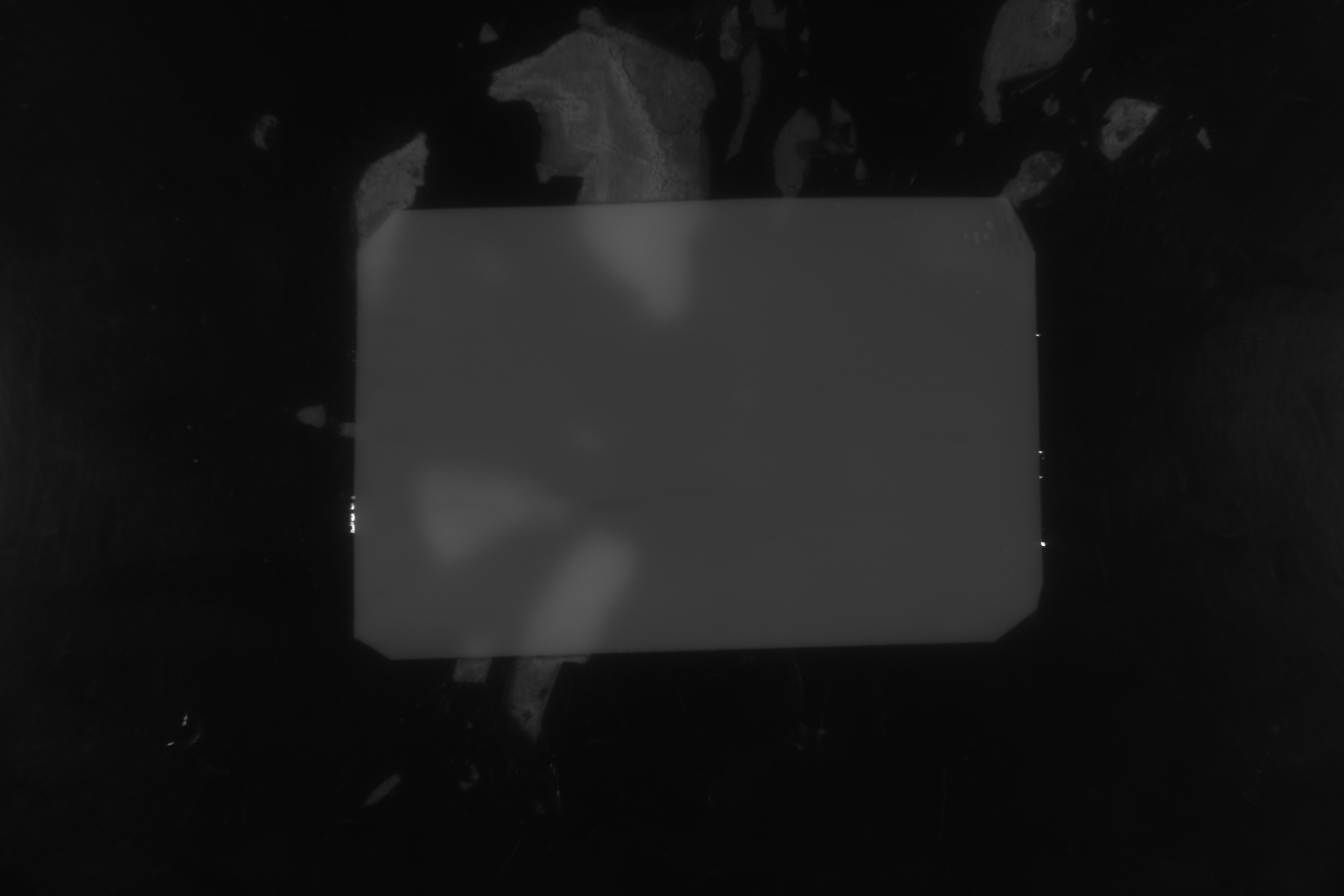

Supplement: Supplementary file 9 — Source data Fig. 7 [file 44318_2025_572_MOESM9_ESM.zip › Figure 7/Figure 7L/V_VIVIT B ACTIN 1 SEC.gel]

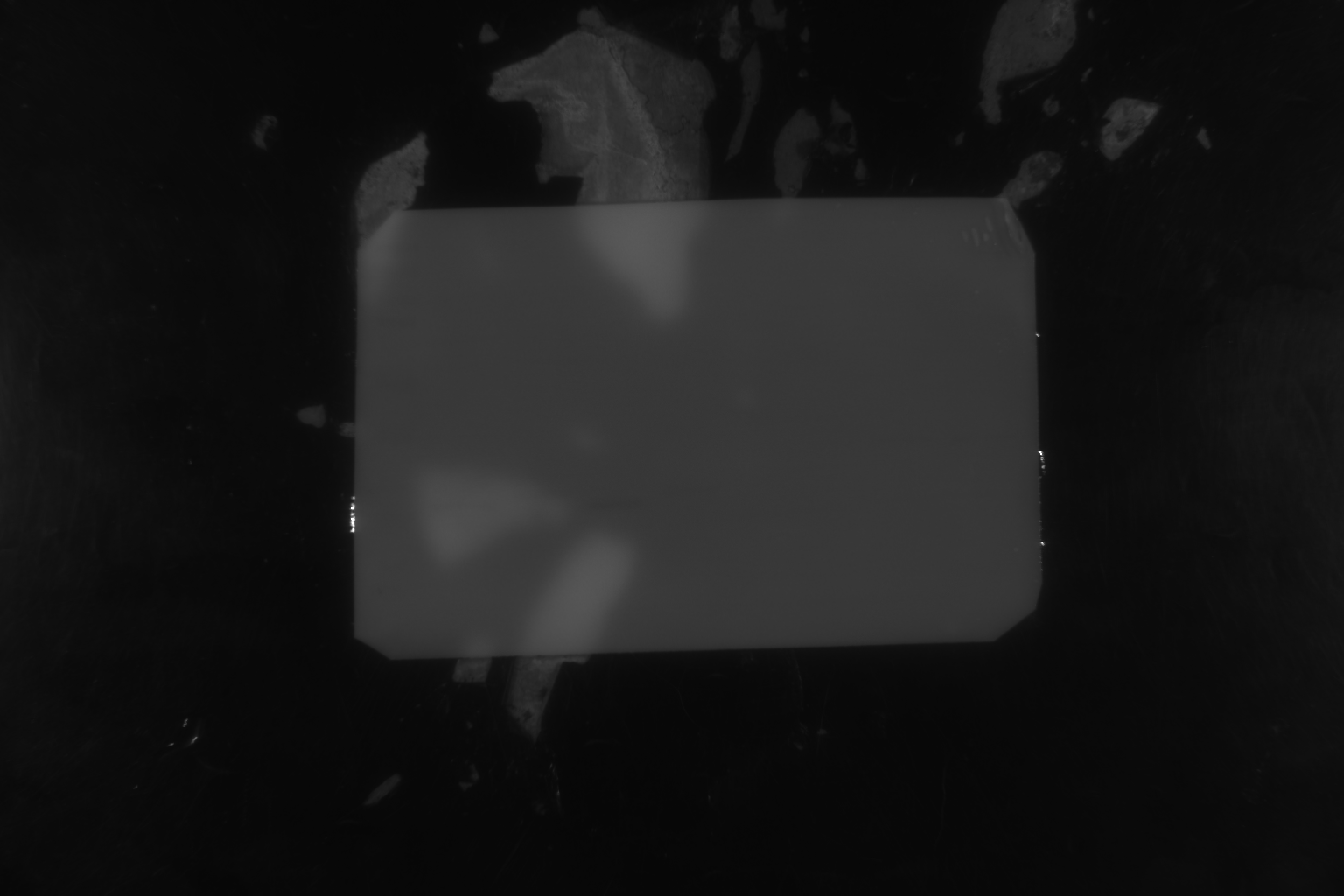

Supplement: Supplementary file 9 — Source data Fig. 7 [file 44318_2025_572_MOESM9_ESM.zip › Figure 7/Figure 7L/V_VIVIT B ACTIN 2 SEC.gel]

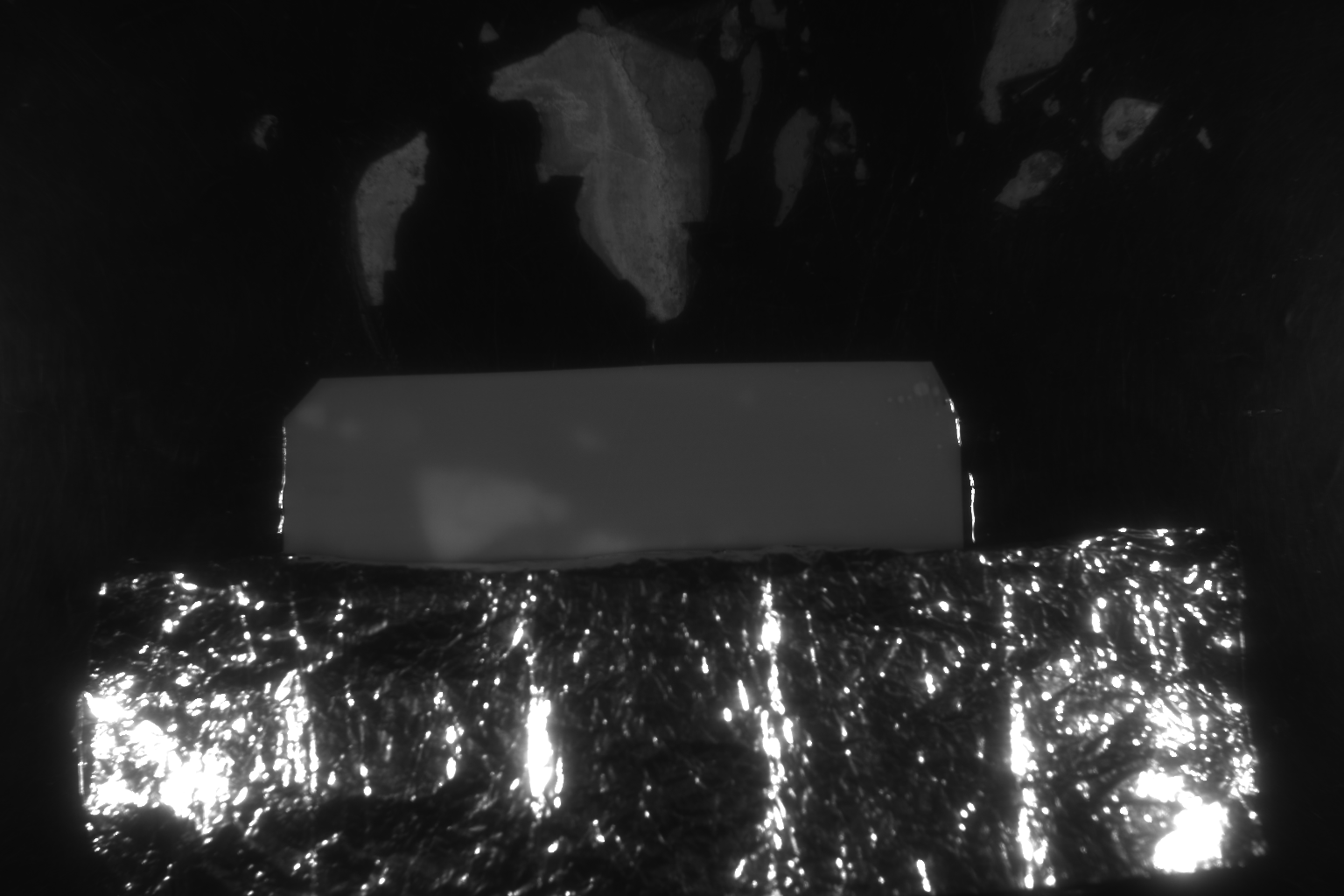

Supplement: Supplementary file 9 — Source data Fig. 7 [file 44318_2025_572_MOESM9_ESM.zip › Figure 7/Figure 7L/V_VIVIT M8 15 SEC.gel]

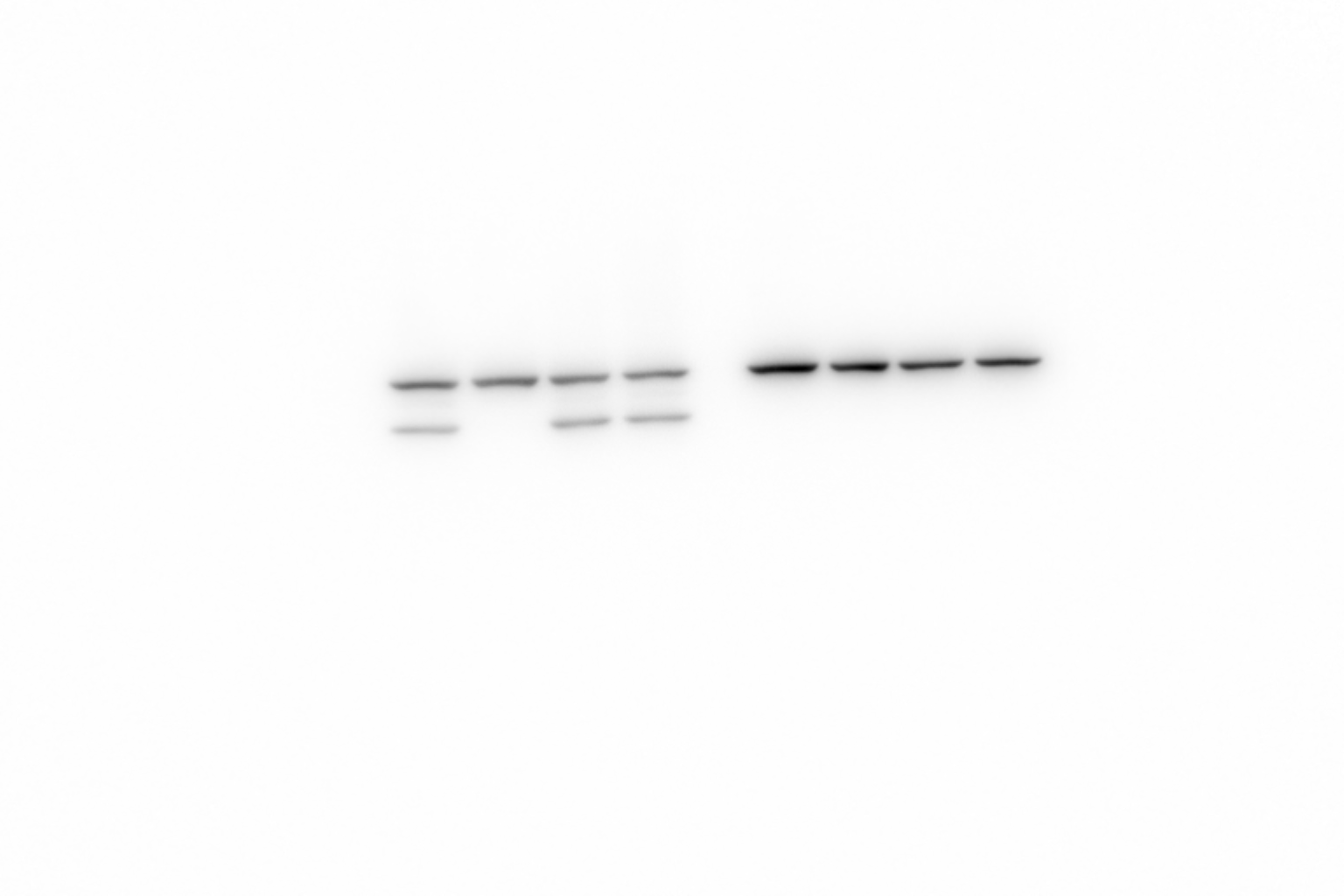

Supplement: Supplementary file 9 — Source data Fig. 7 [file 44318_2025_572_MOESM9_ESM.zip › Figure 7/Figure 7O/B ACTIN 2 SEC.gel]

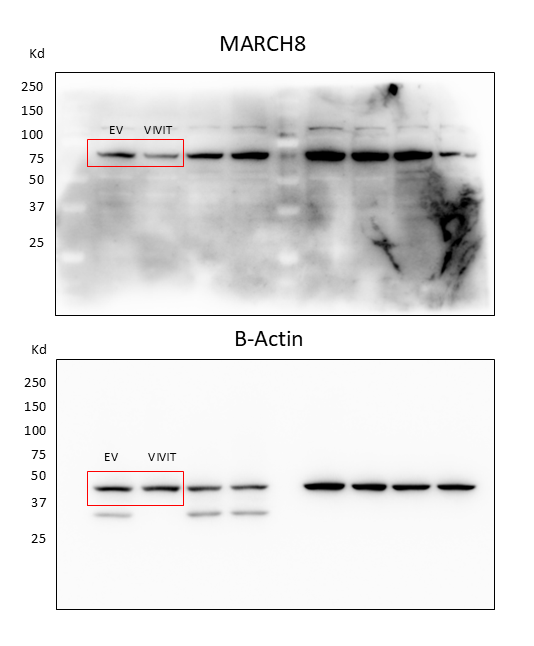

Supplement: Supplementary file 9 — Source data Fig. 7 [file 44318_2025_572_MOESM9_ESM.zip › Figure 7/Figure 7O/Figure 7O.png]

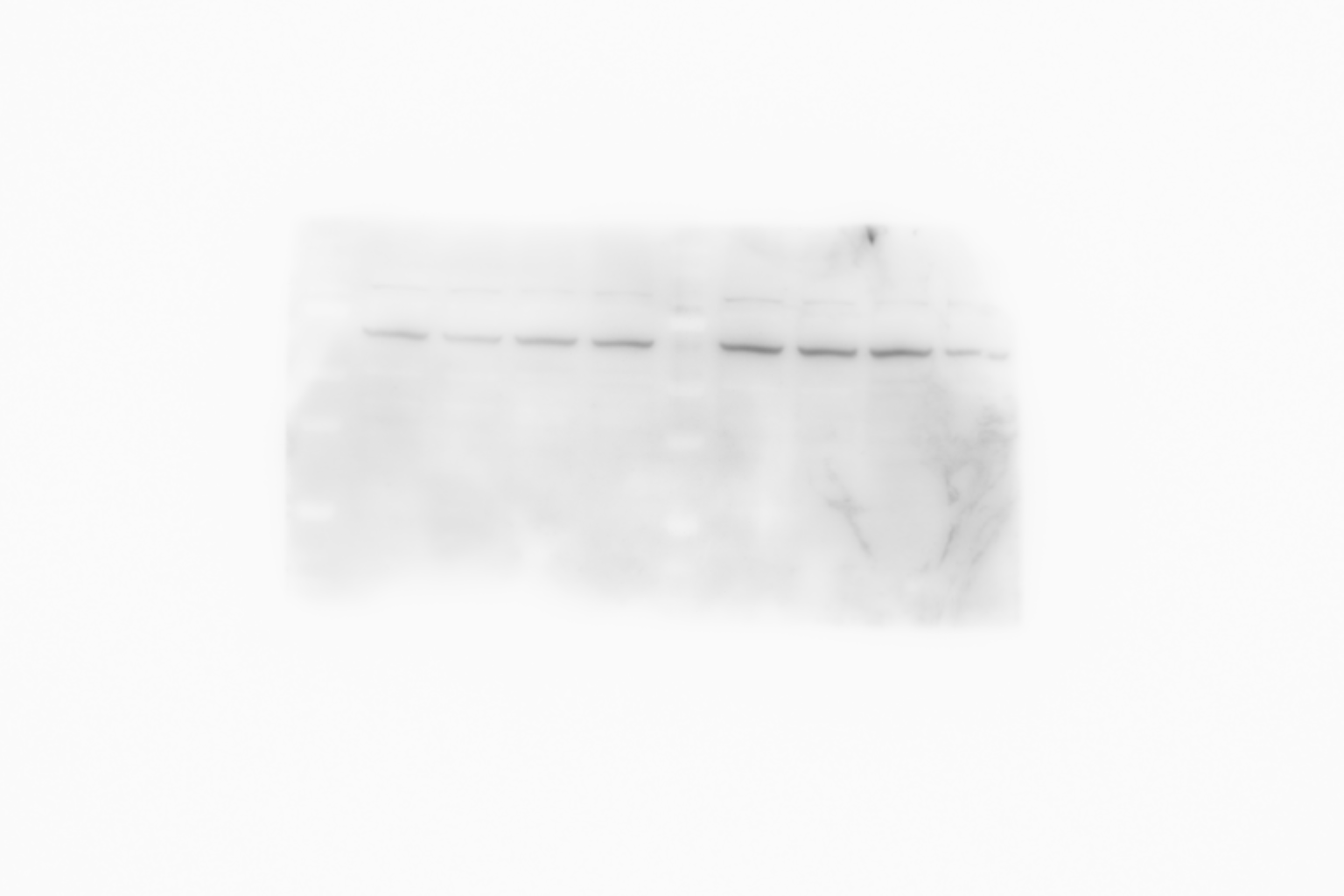

Supplement: Supplementary file 9 — Source data Fig. 7 [file 44318_2025_572_MOESM9_ESM.zip › Figure 7/Figure 7O/M8 240 SEC.gel]

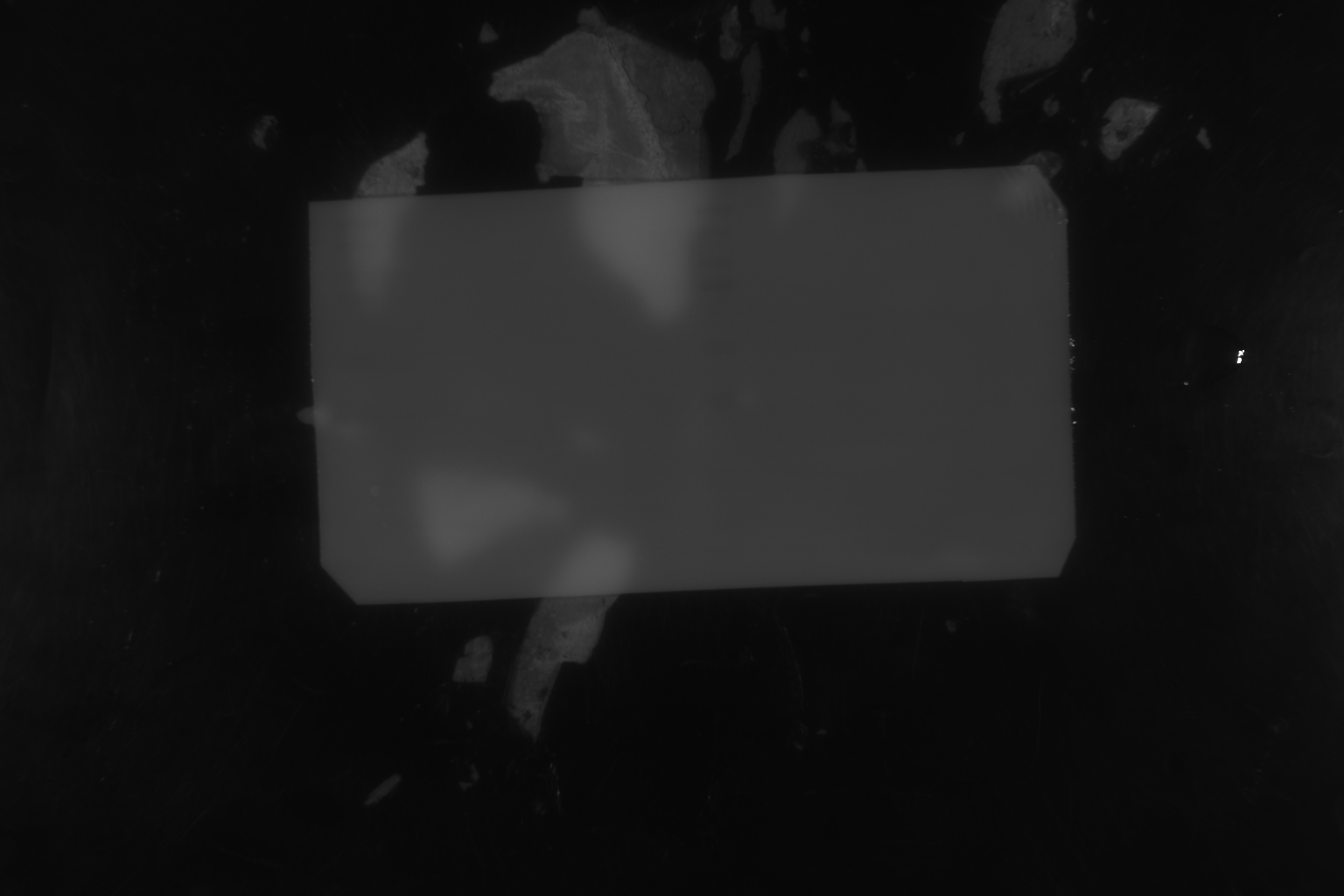

Supplement: Supplementary file 9 — Source data Fig. 7 [file 44318_2025_572_MOESM9_ESM.zip › Figure 7/Figure 7O/V_B ACTIN 2 SEC.gel]

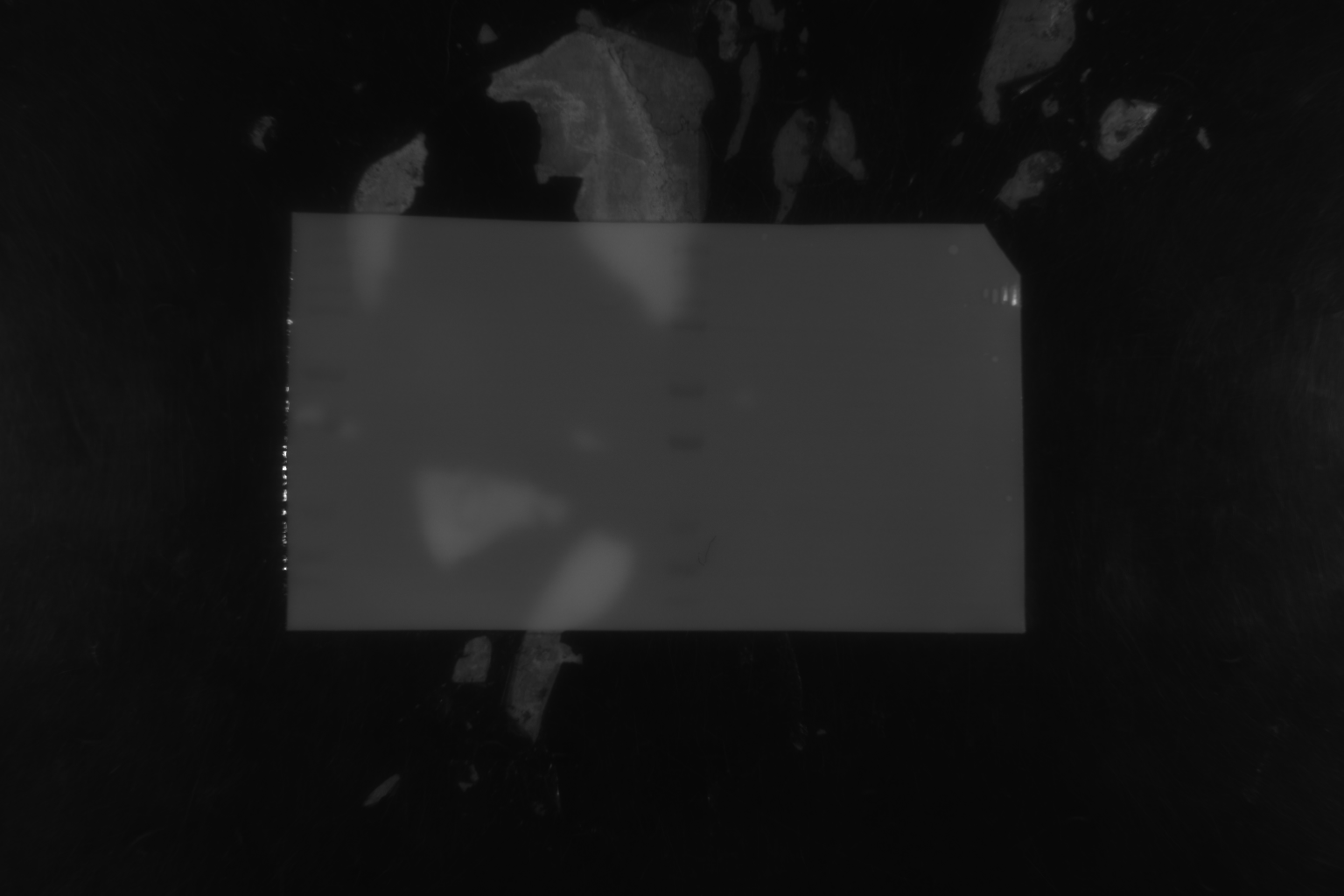

Supplement: Supplementary file 9 — Source data Fig. 7 [file 44318_2025_572_MOESM9_ESM.zip › Figure 7/Figure 7O/V_M8 240 SEC.gel]
