# Supplementary figures and images for: NFATc1 drives Orai3 transcription and proteolysis by harnessing epigenome differences in the MARCH8 promoter (part 3 of 3)
Source: EMBO J. 2025 Sep 29;44(21):6137–67. doi: 10.1038/s44318-025-00572-4 (PMC12583688; doi:10.1038/s44318-025-00572-4)

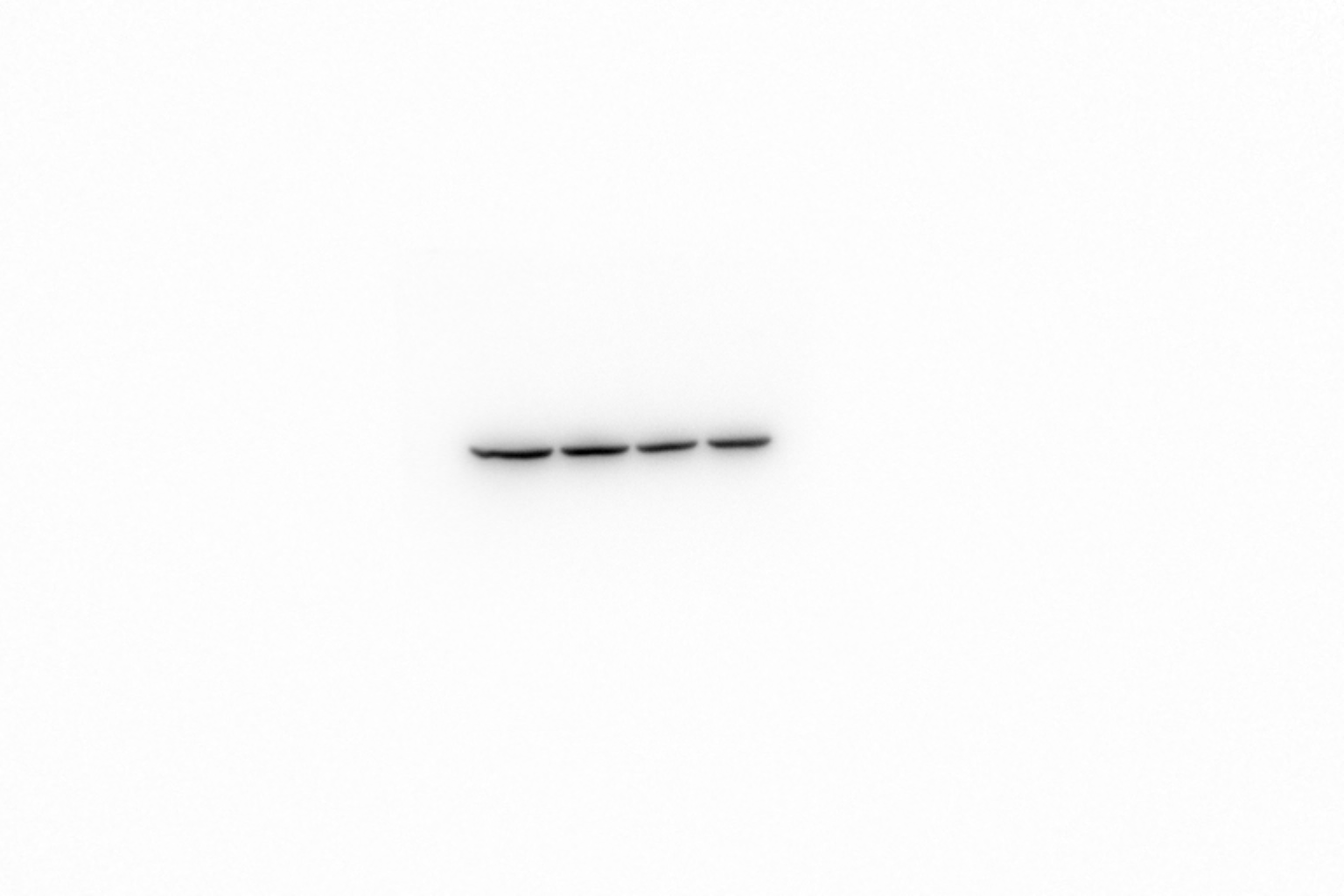

Supplement: Supplementary file 9 — Source data Fig. 7 [file 44318_2025_572_MOESM9_ESM.zip › Figure 7/Figure 7R/B ACTIN 2 SEC.gel]

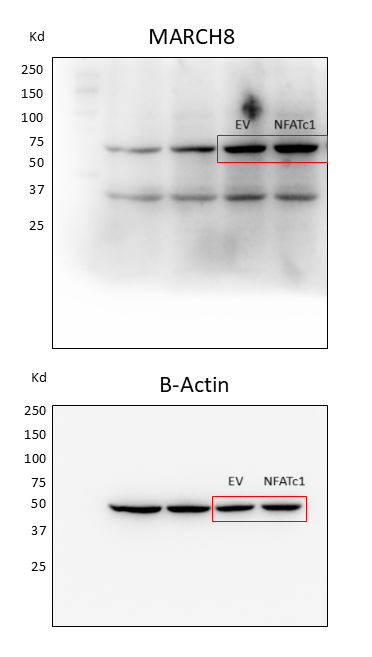

Supplement: Supplementary file 9 — Source data Fig. 7 [file 44318_2025_572_MOESM9_ESM.zip › Figure 7/Figure 7R/Figure 7R.png]

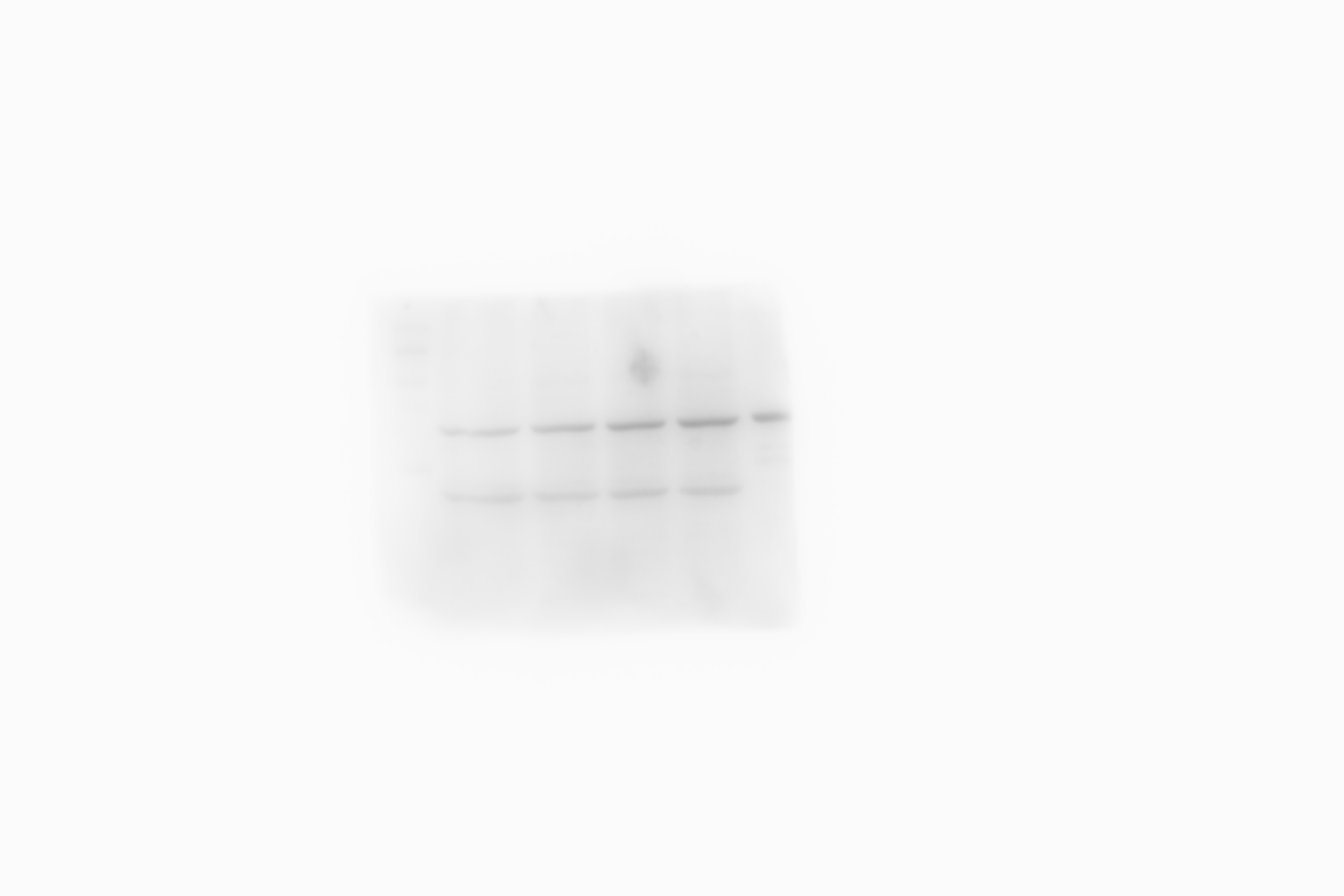

Supplement: Supplementary file 9 — Source data Fig. 7 [file 44318_2025_572_MOESM9_ESM.zip › Figure 7/Figure 7R/MARCH 8 60 SEC.gel]

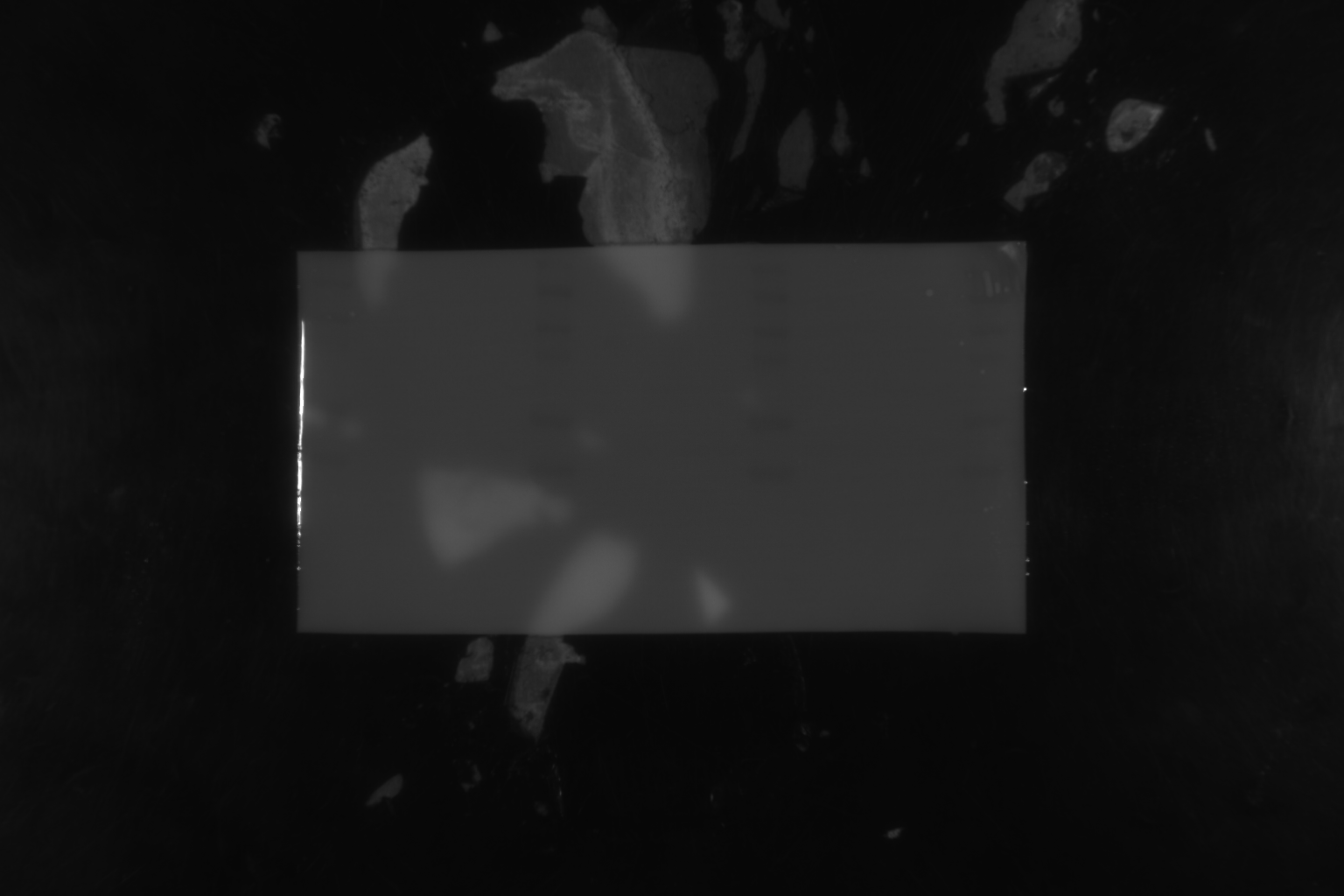

Supplement: Supplementary file 9 — Source data Fig. 7 [file 44318_2025_572_MOESM9_ESM.zip › Figure 7/Figure 7R/V_B ACTIN 3 SEC.gel]

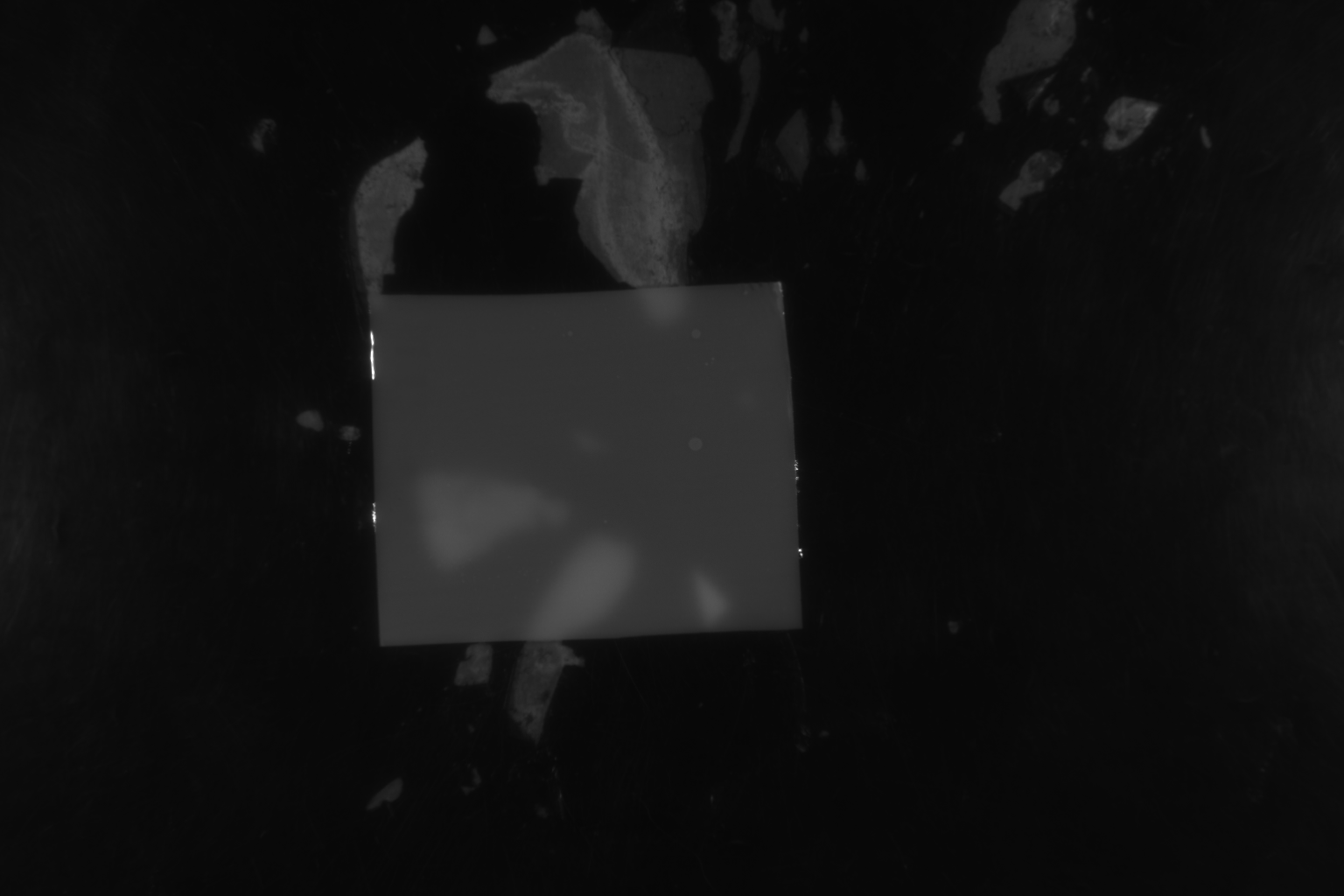

Supplement: Supplementary file 9 — Source data Fig. 7 [file 44318_2025_572_MOESM9_ESM.zip › Figure 7/Figure 7R/V_MARCH 8 60 SEC.gel]

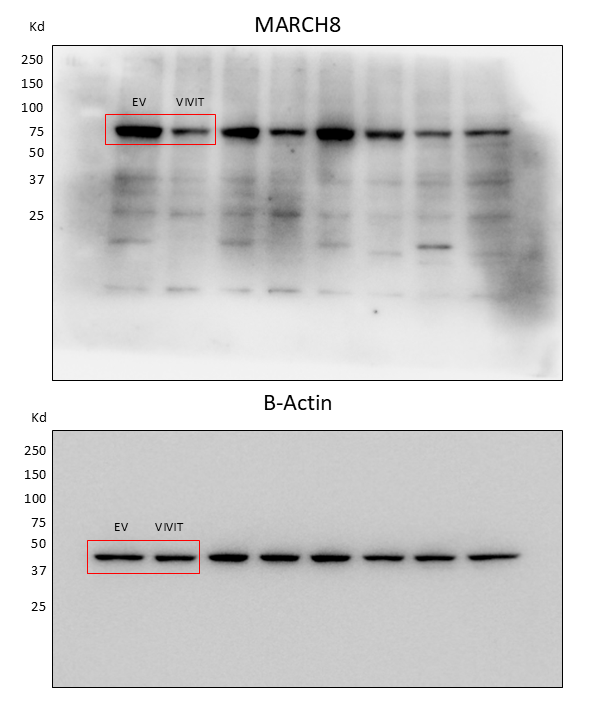

Supplement: Supplementary file 9 — Source data Fig. 7 [file 44318_2025_572_MOESM9_ESM.zip › Figure 7/Figure 7U/Figure 7U.png]

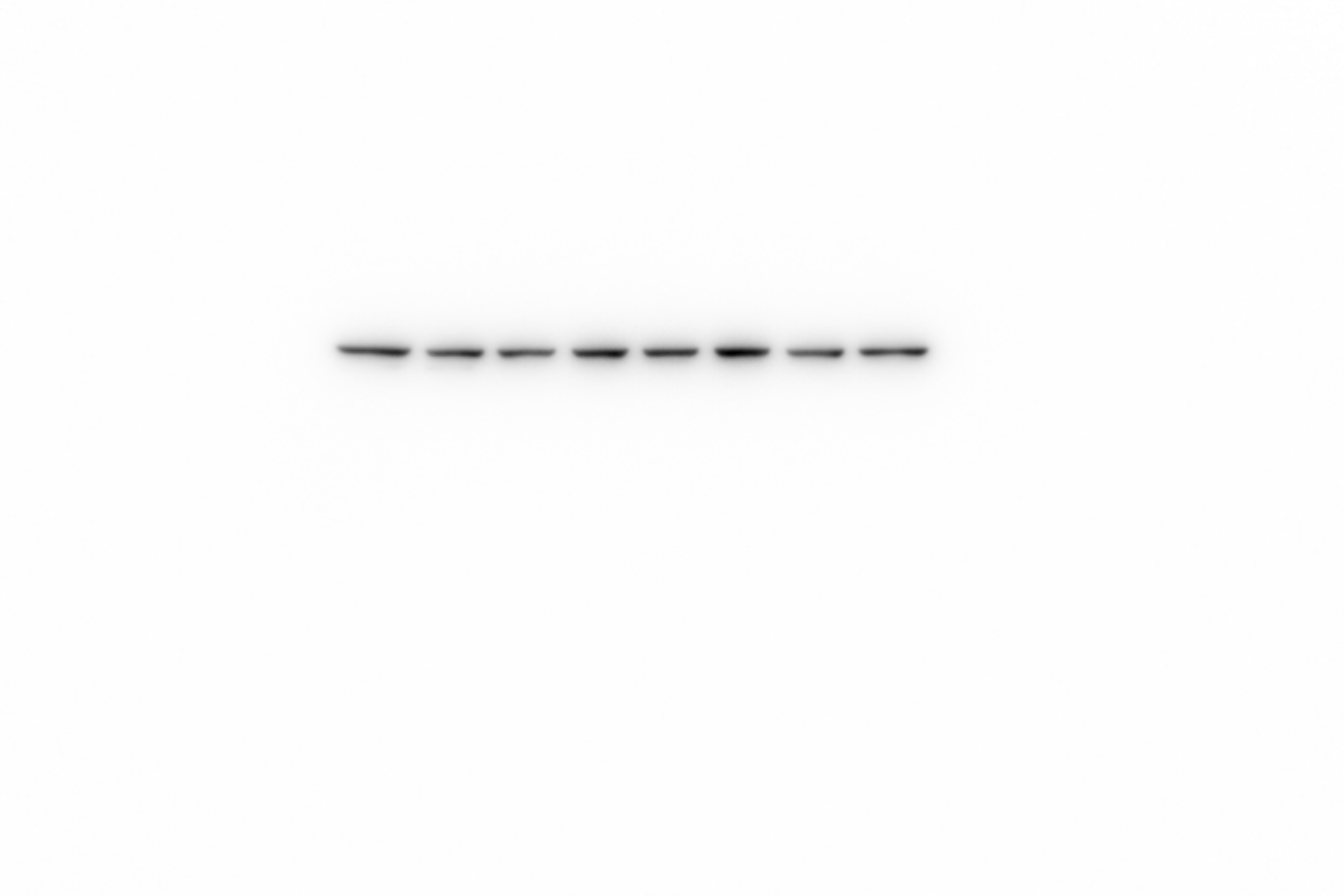

Supplement: Supplementary file 9 — Source data Fig. 7 [file 44318_2025_572_MOESM9_ESM.zip › Figure 7/Figure 7U/VIVIT B ACTIN 2 SEC.gel]

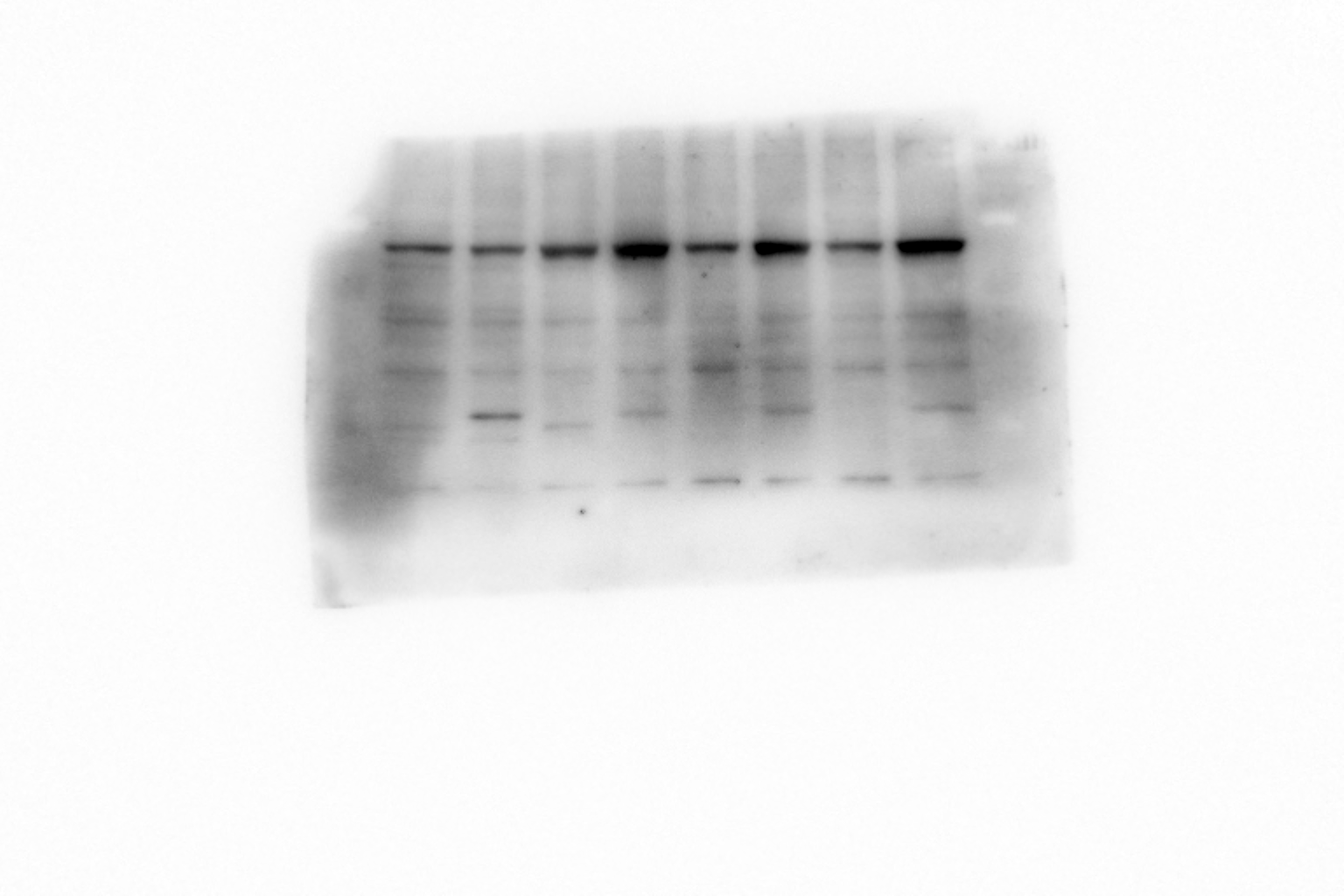

Supplement: Supplementary file 9 — Source data Fig. 7 [file 44318_2025_572_MOESM9_ESM.zip › Figure 7/Figure 7U/VIVIT M8 15 SEC.gel]

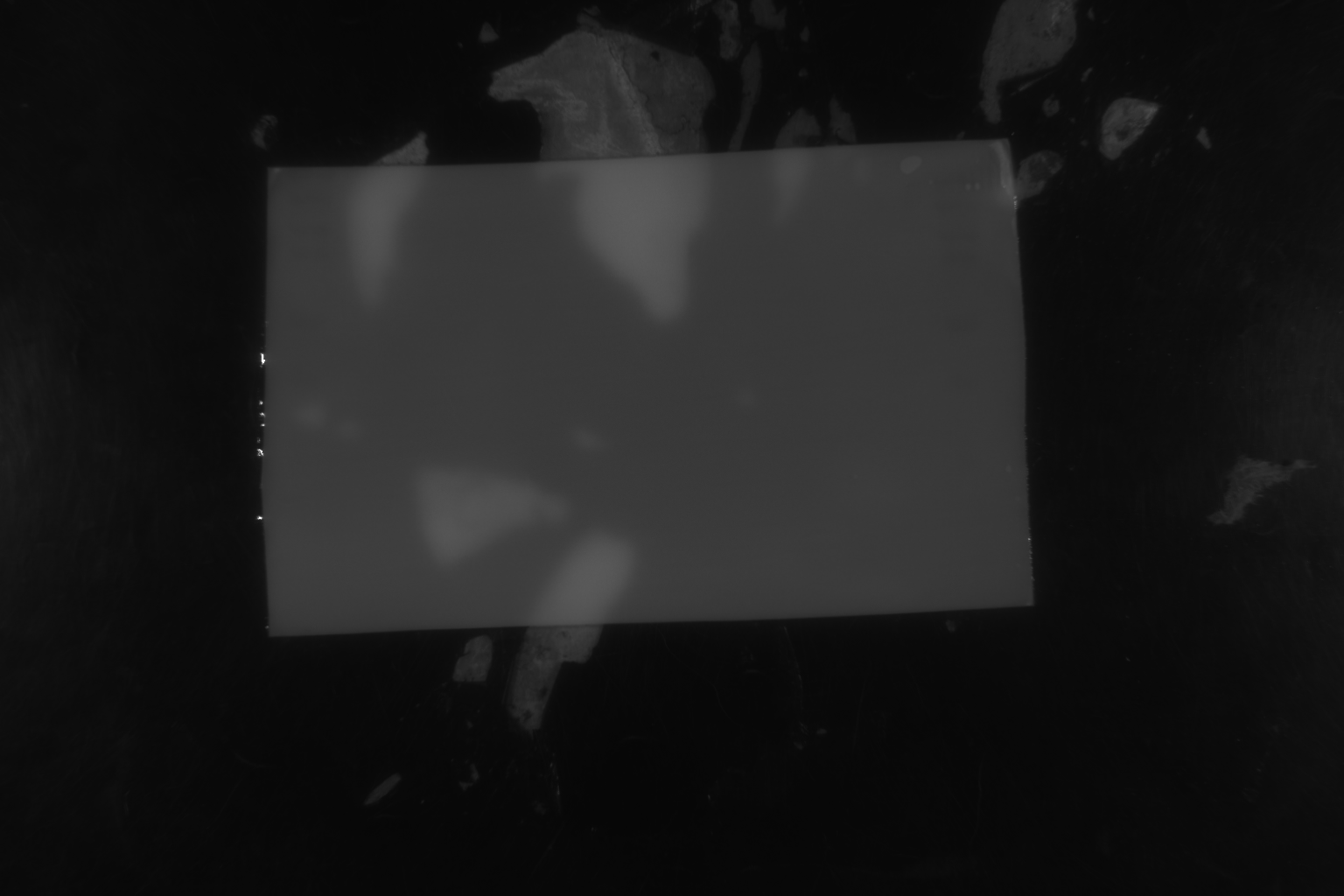

Supplement: Supplementary file 9 — Source data Fig. 7 [file 44318_2025_572_MOESM9_ESM.zip › Figure 7/Figure 7U/V_VIVIT B ACTIN 2 SEC.gel]

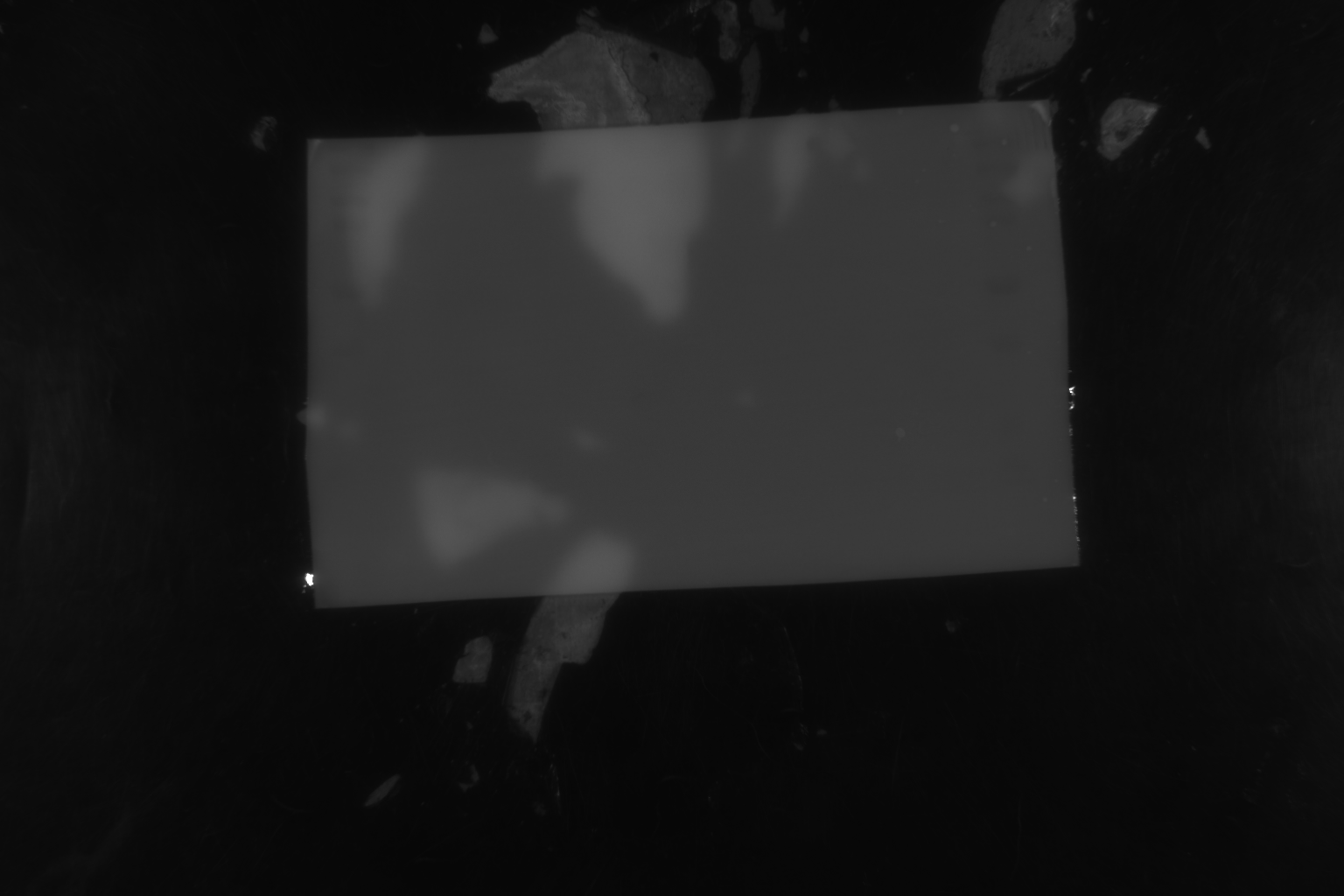

Supplement: Supplementary file 9 — Source data Fig. 7 [file 44318_2025_572_MOESM9_ESM.zip › Figure 7/Figure 7U/V_VIVIT M8 15 SEC.gel]

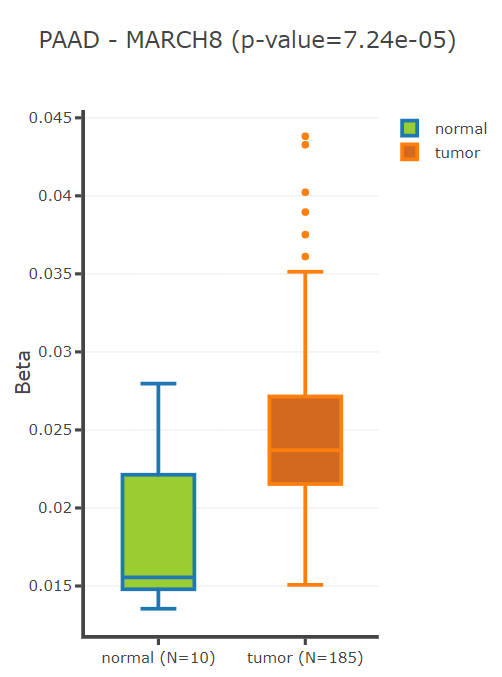

Supplement: Supplementary file 10 — Source data Fig. 8 [file 44318_2025_572_MOESM10_ESM.zip › Figure 8/Figure 8A/March 8 Methylation PAAD.png]

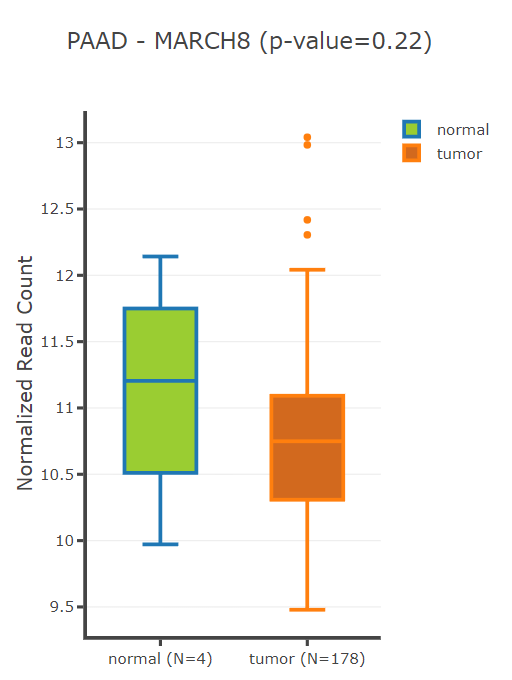

Supplement: Supplementary file 10 — Source data Fig. 8 [file 44318_2025_572_MOESM10_ESM.zip › Figure 8/Figure 8B/March 8 Expression PAAD.png]

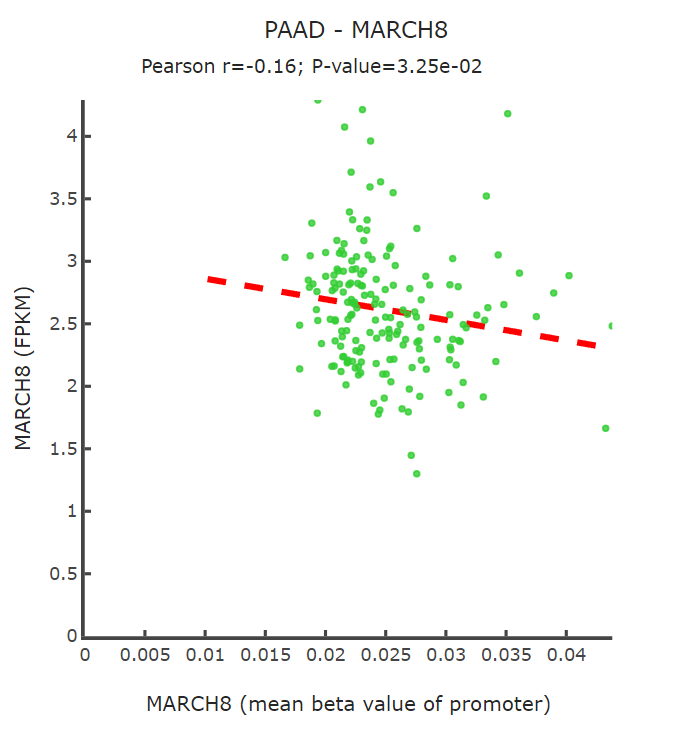

Supplement: Supplementary file 10 — Source data Fig. 8 [file 44318_2025_572_MOESM10_ESM.zip › Figure 8/Figure 8C/Pearson Coefficient .png]

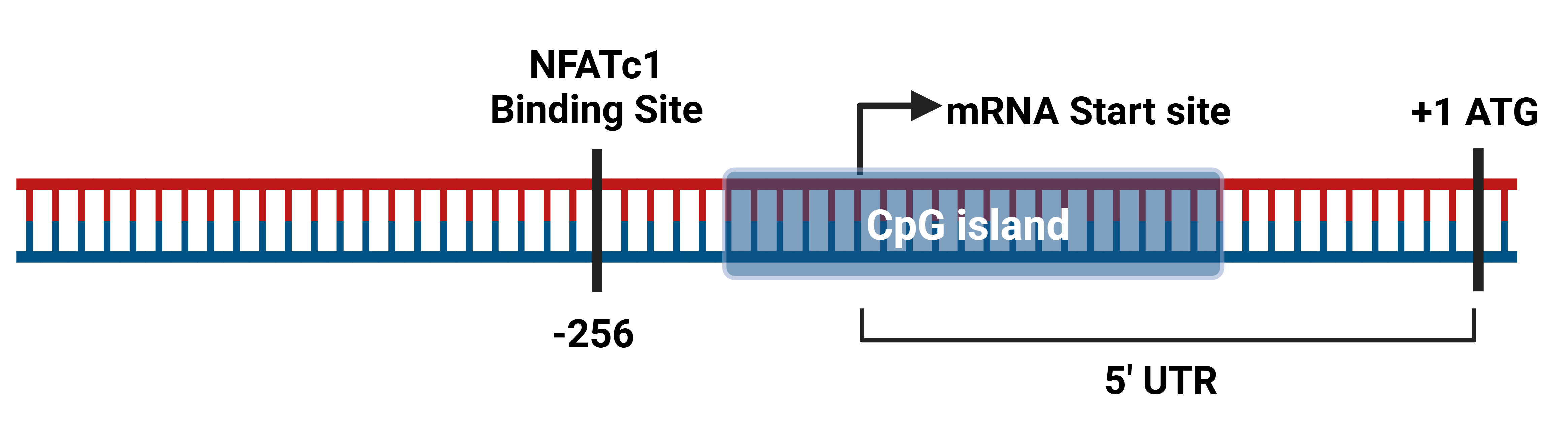

Supplement: Supplementary file 10 — Source data Fig. 8 [file 44318_2025_572_MOESM10_ESM.zip › Figure 8/Figure 8D/M8 Promoter CpG island.png]

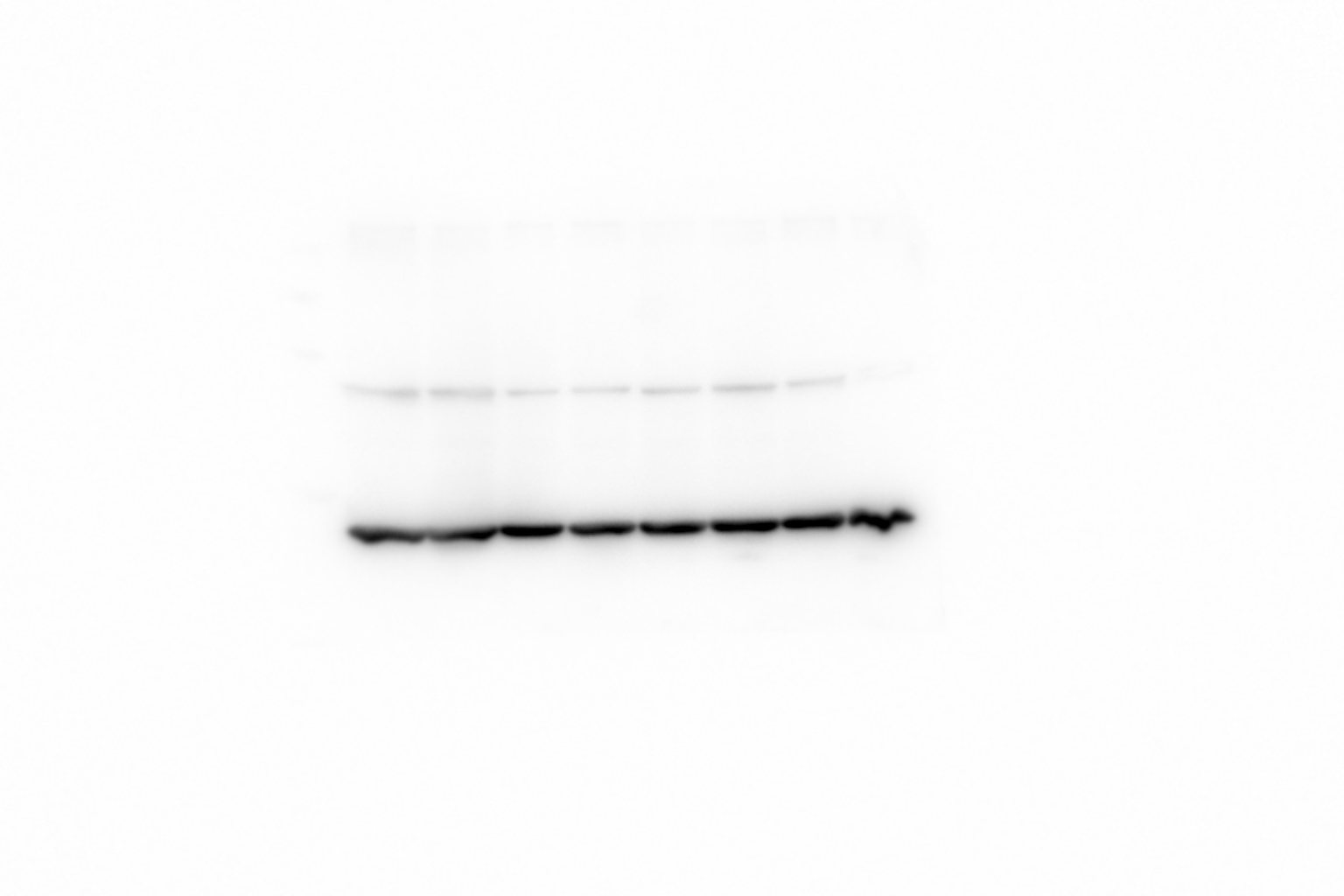

Supplement: Supplementary file 10 — Source data Fig. 8 [file 44318_2025_572_MOESM10_ESM.zip › Figure 8/Figure 8G/B ACTIN 2 1 SEC.gel]

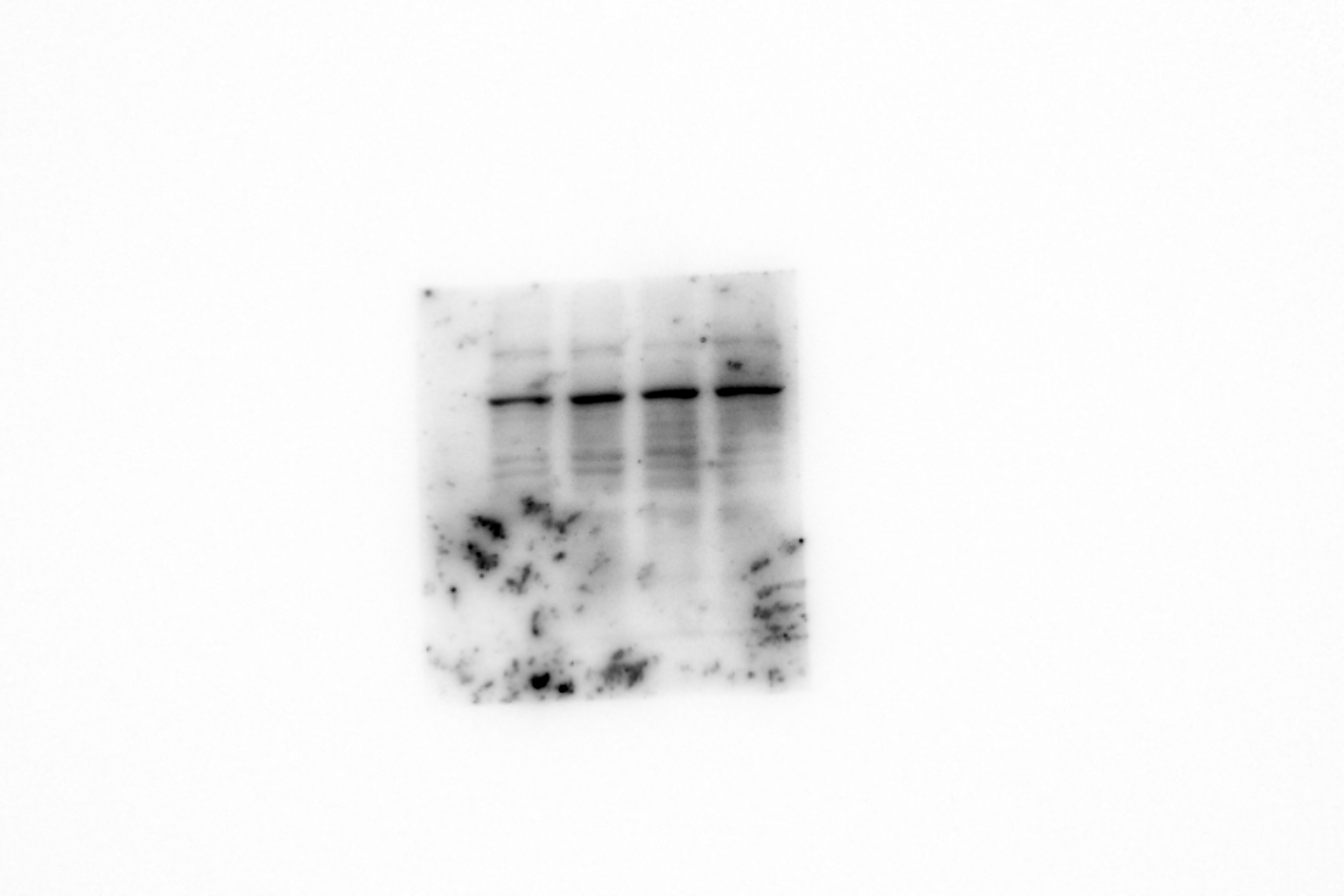

Supplement: Supplementary file 10 — Source data Fig. 8 [file 44318_2025_572_MOESM10_ESM.zip › Figure 8/Figure 8G/DAC M8 4 MIN.gel]

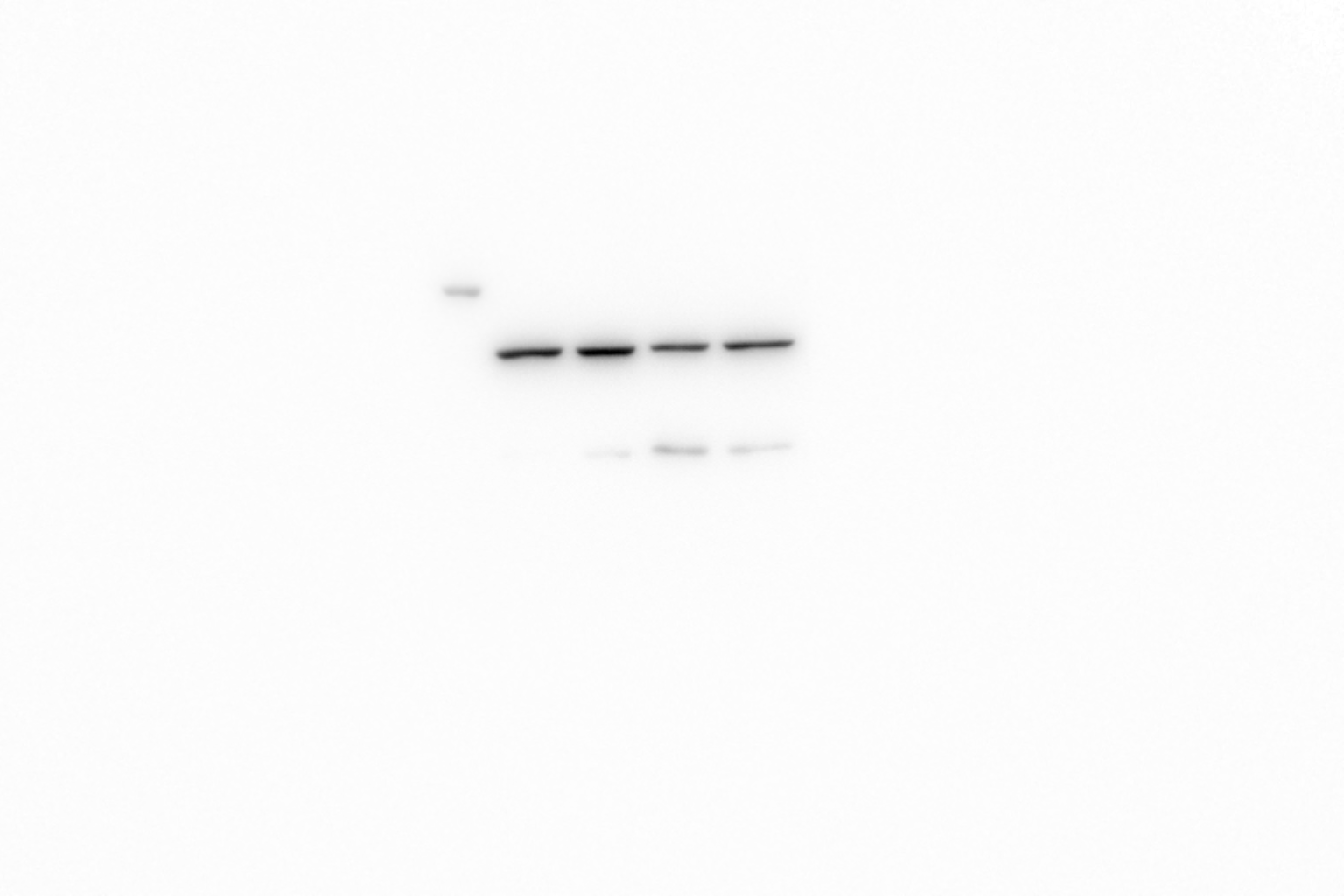

Supplement: Supplementary file 10 — Source data Fig. 8 [file 44318_2025_572_MOESM10_ESM.zip › Figure 8/Figure 8G/DAC TUB 4 SEC.gel]

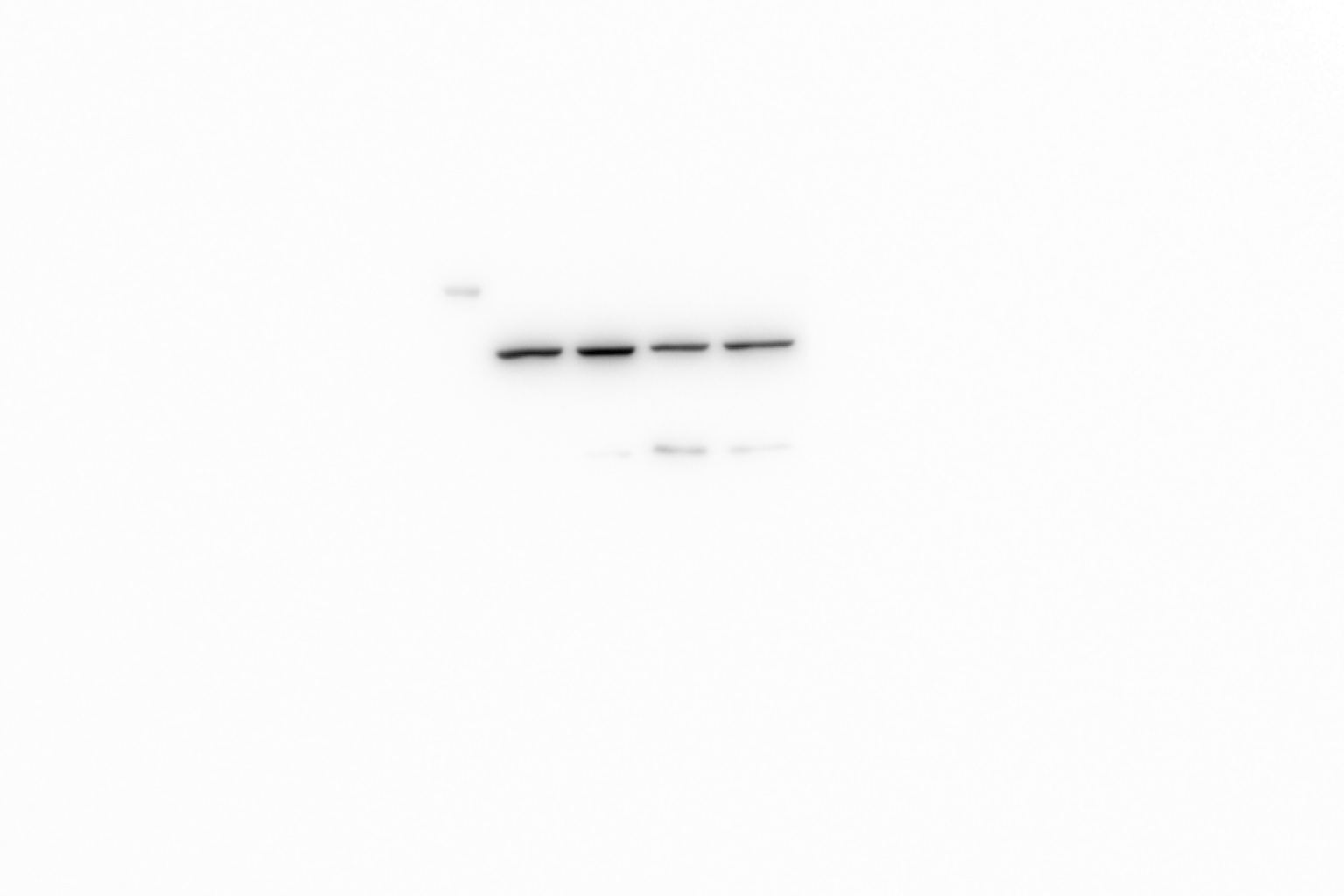

Supplement: Supplementary file 10 — Source data Fig. 8 [file 44318_2025_572_MOESM10_ESM.zip › Figure 8/Figure 8G/DAC TUB 8 SEC.gel]

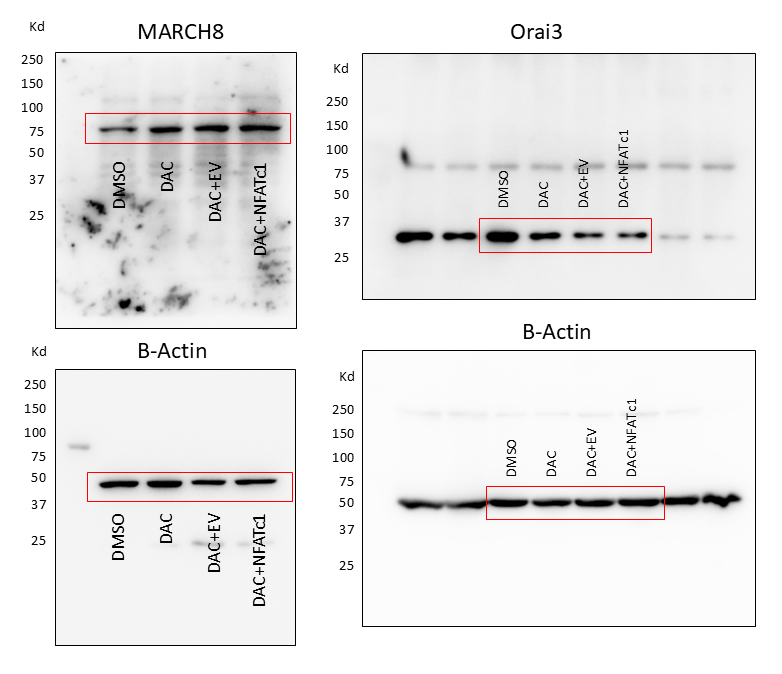

Supplement: Supplementary file 10 — Source data Fig. 8 [file 44318_2025_572_MOESM10_ESM.zip › Figure 8/Figure 8G/Figure 8G.png]

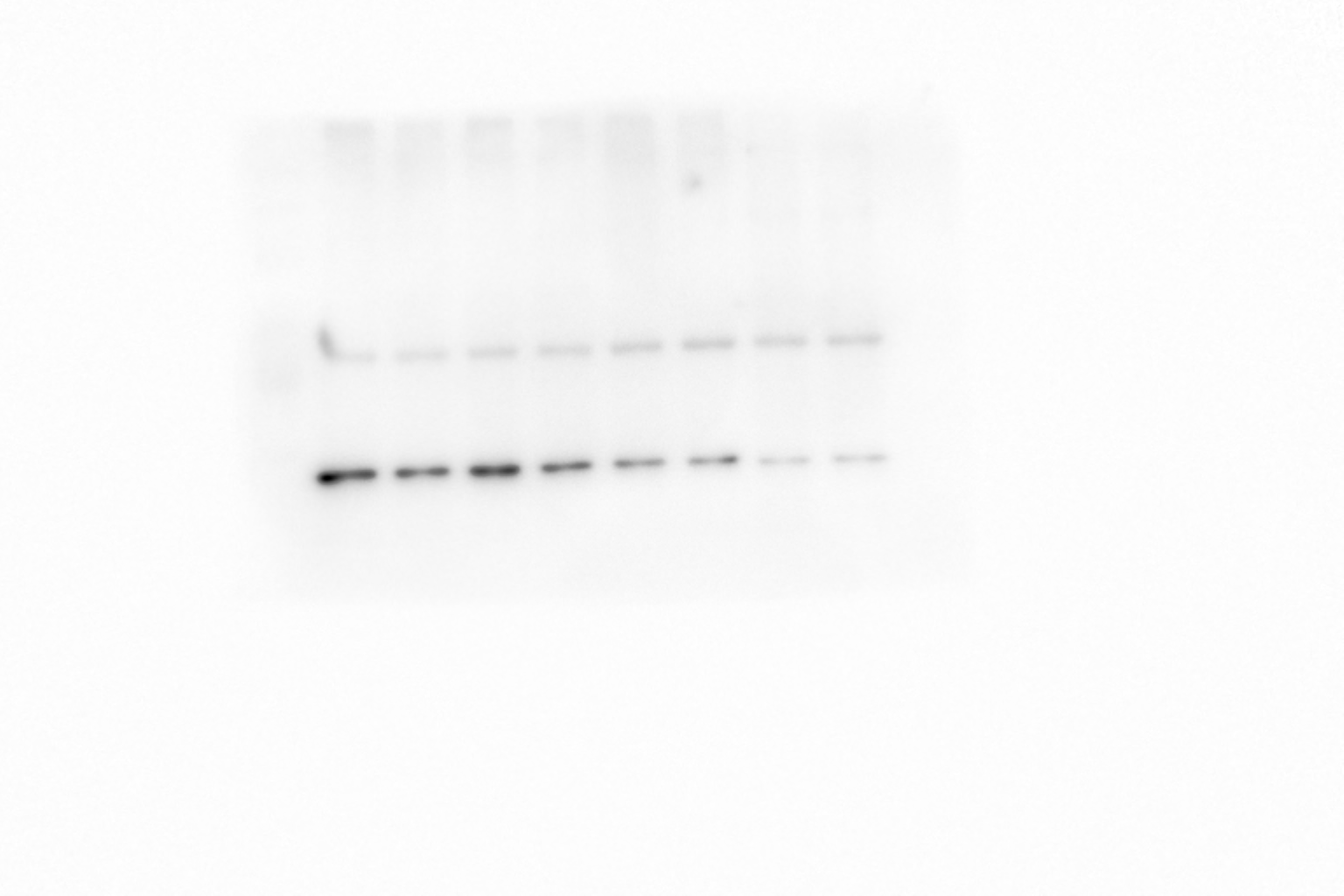

Supplement: Supplementary file 10 — Source data Fig. 8 [file 44318_2025_572_MOESM10_ESM.zip › Figure 8/Figure 8G/ORAI 3 1 SEC.gel]

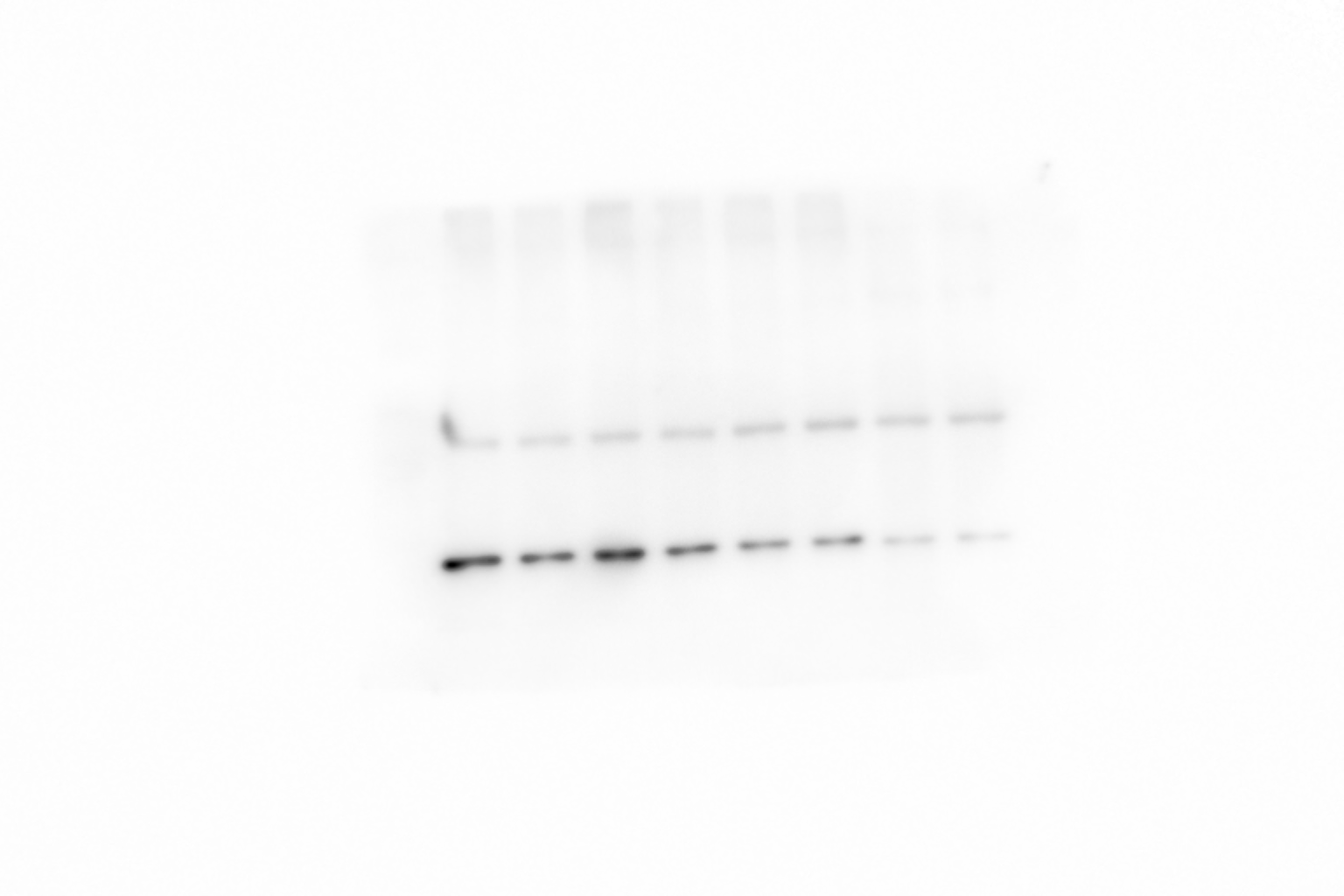

Supplement: Supplementary file 10 — Source data Fig. 8 [file 44318_2025_572_MOESM10_ESM.zip › Figure 8/Figure 8G/ORAI 3 4 SEC.gel]

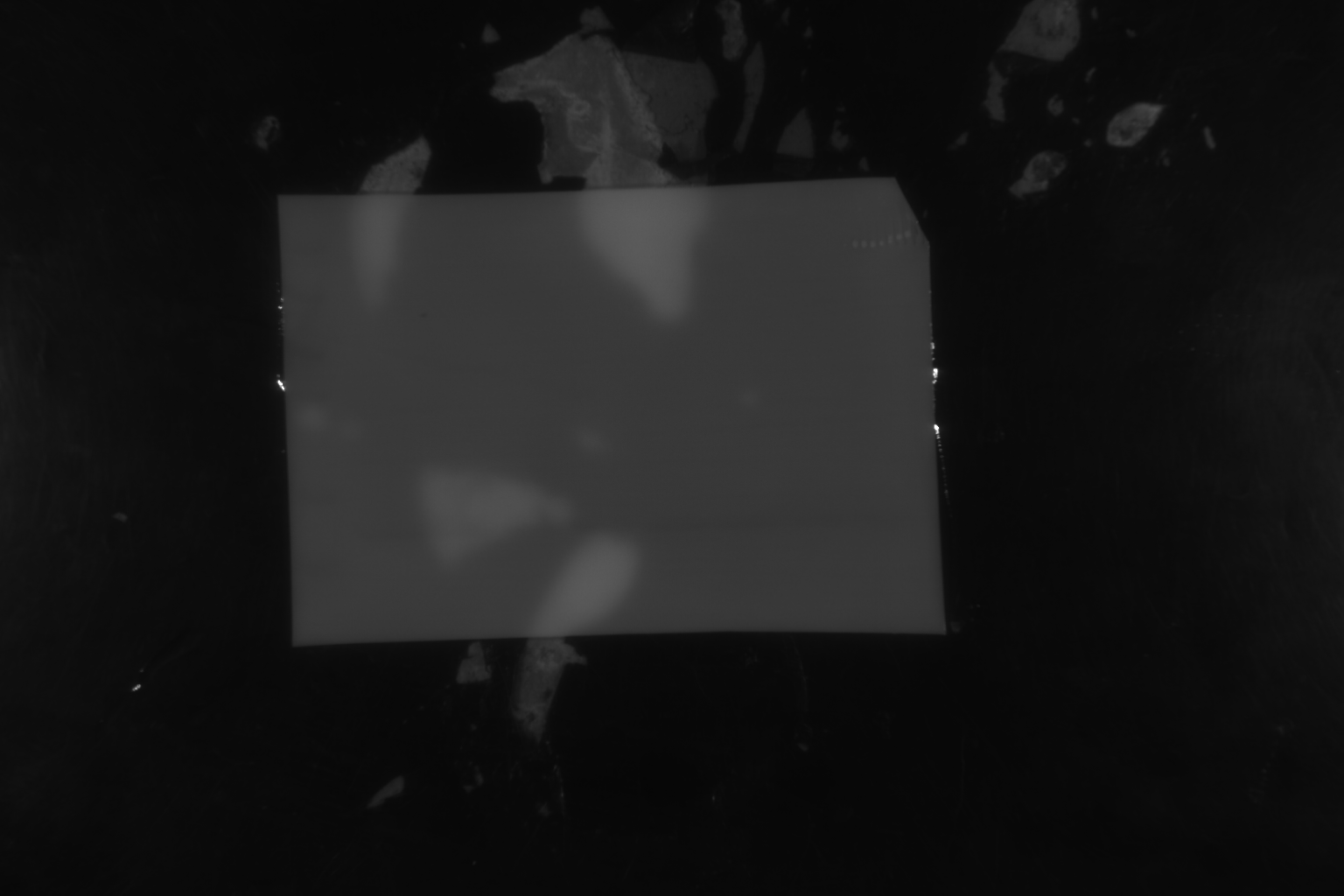

Supplement: Supplementary file 10 — Source data Fig. 8 [file 44318_2025_572_MOESM10_ESM.zip › Figure 8/Figure 8G/V_B ACTIN 2 1 SEC.gel]

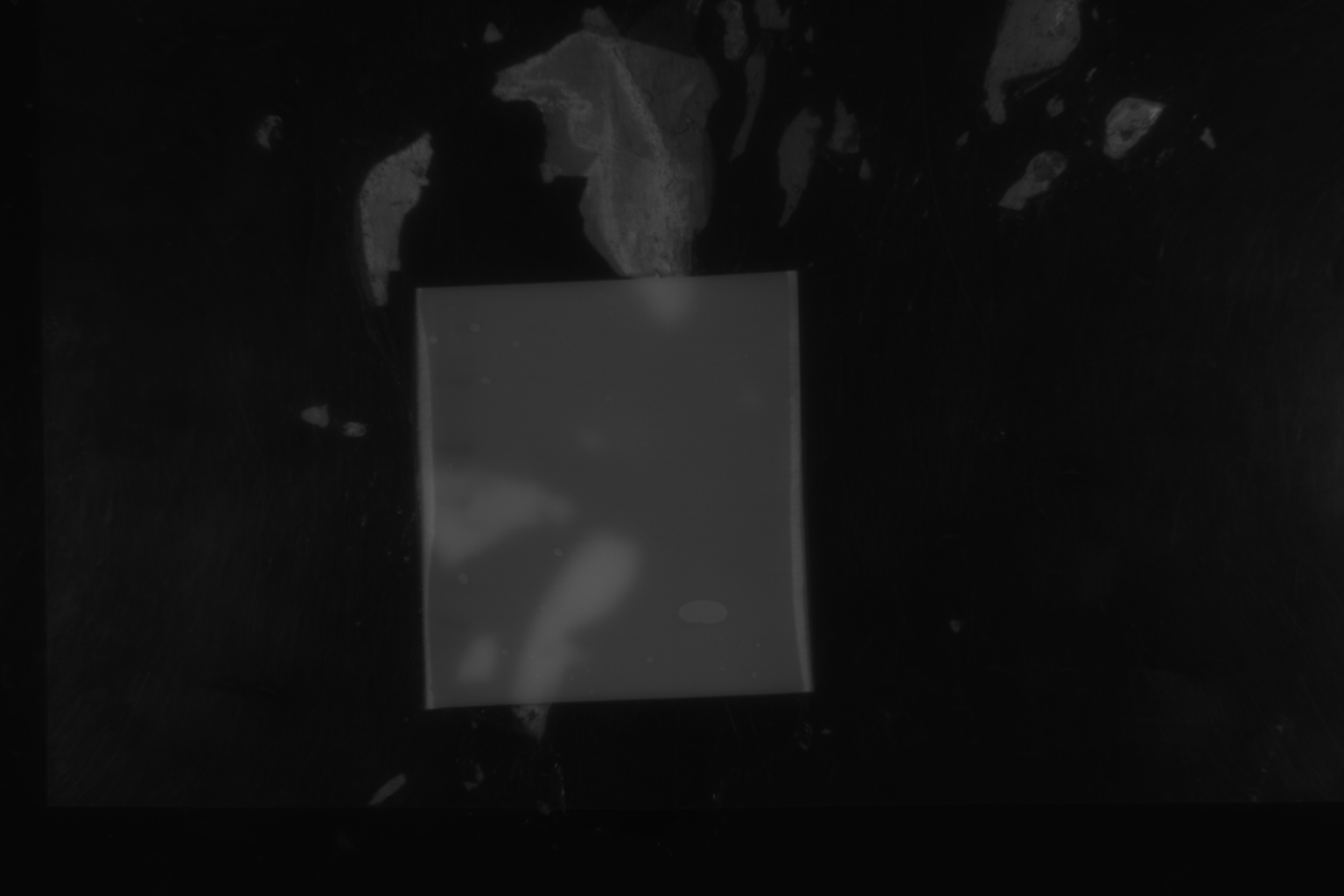

Supplement: Supplementary file 10 — Source data Fig. 8 [file 44318_2025_572_MOESM10_ESM.zip › Figure 8/Figure 8G/V_DAC M8 4 MIN.gel]

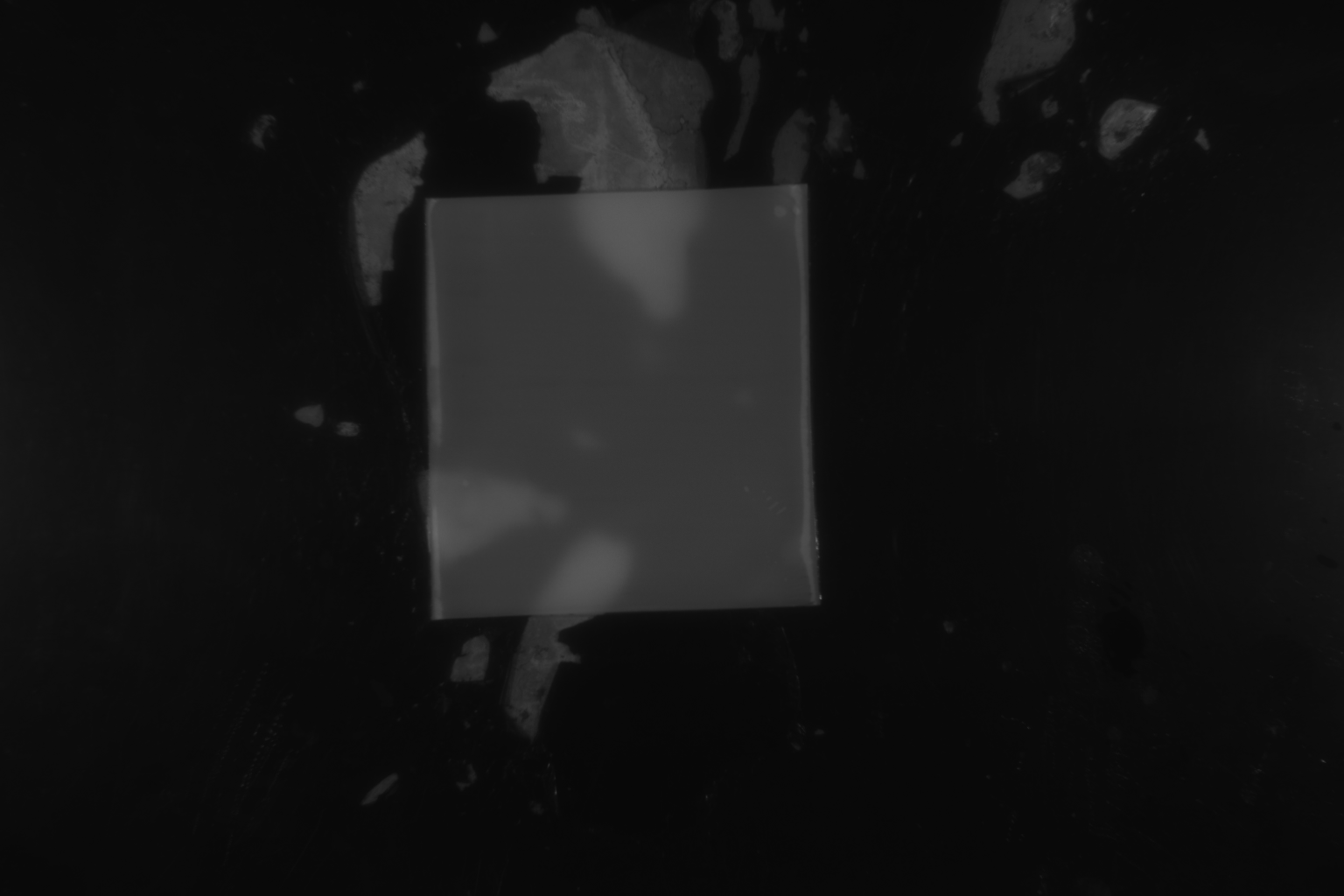

Supplement: Supplementary file 10 — Source data Fig. 8 [file 44318_2025_572_MOESM10_ESM.zip › Figure 8/Figure 8G/V_DAC TUB 4 SEC.gel]

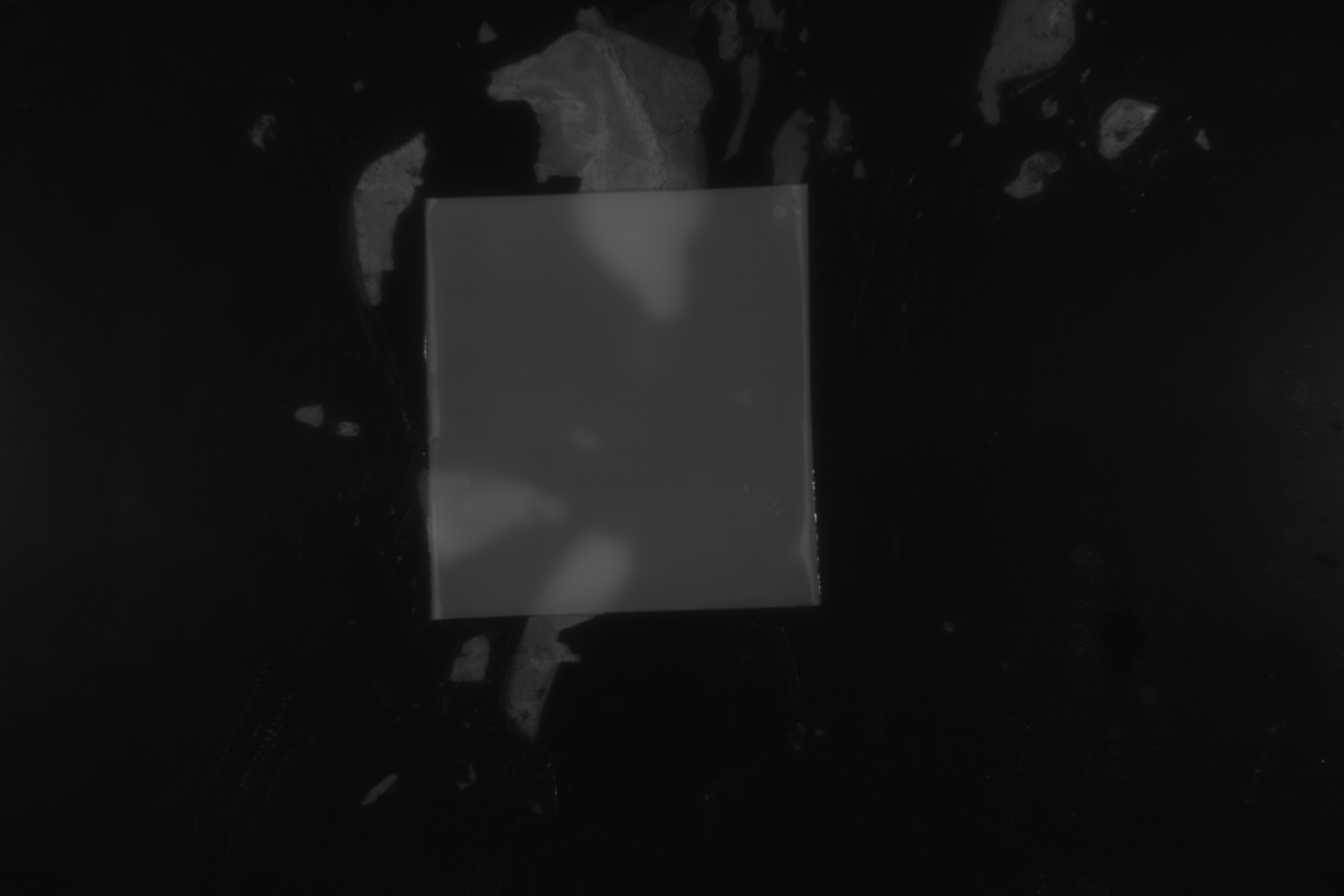

Supplement: Supplementary file 10 — Source data Fig. 8 [file 44318_2025_572_MOESM10_ESM.zip › Figure 8/Figure 8G/V_DAC TUB 8 SEC.gel]

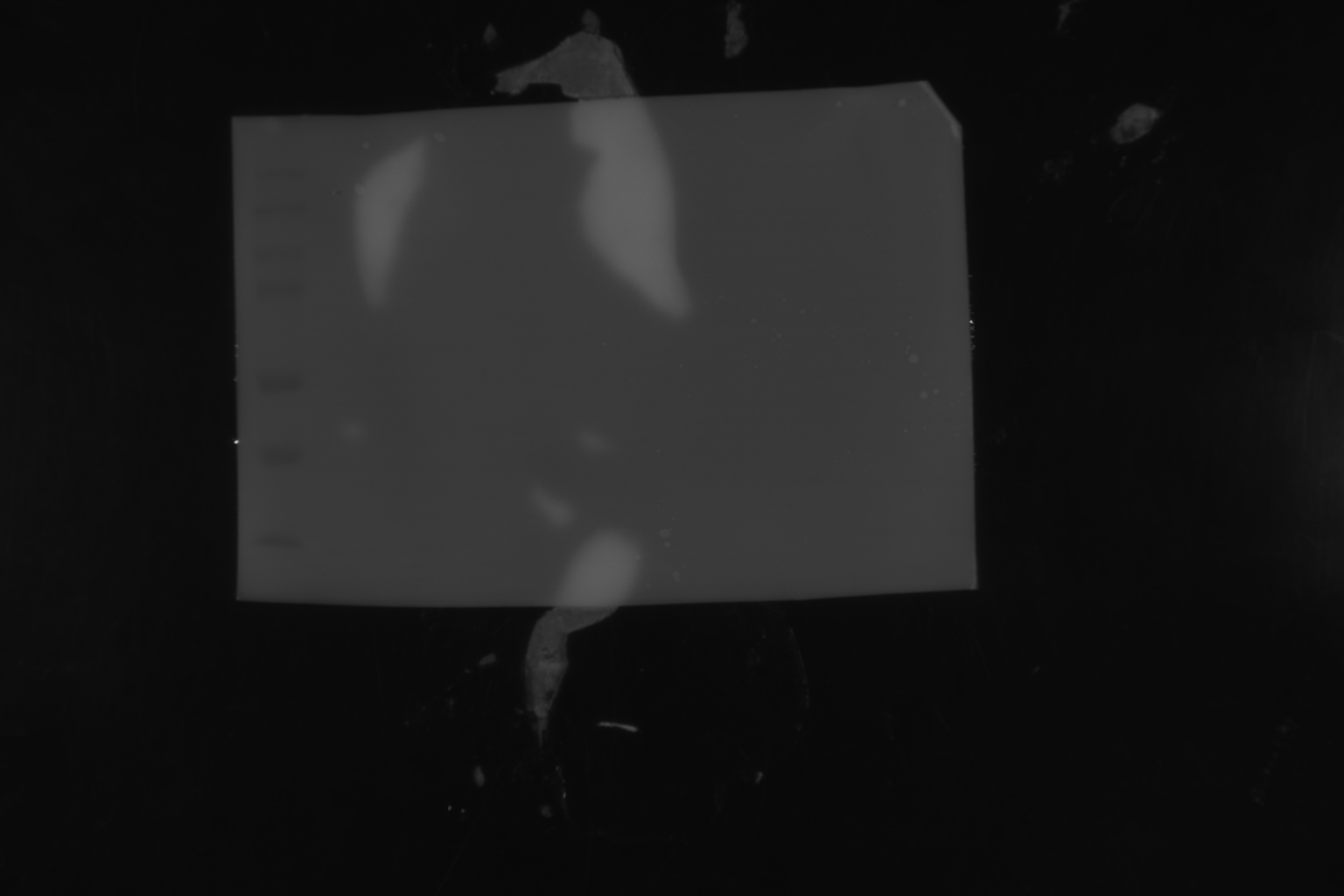

Supplement: Supplementary file 10 — Source data Fig. 8 [file 44318_2025_572_MOESM10_ESM.zip › Figure 8/Figure 8G/V_ORAI 3 1 SEC.gel]

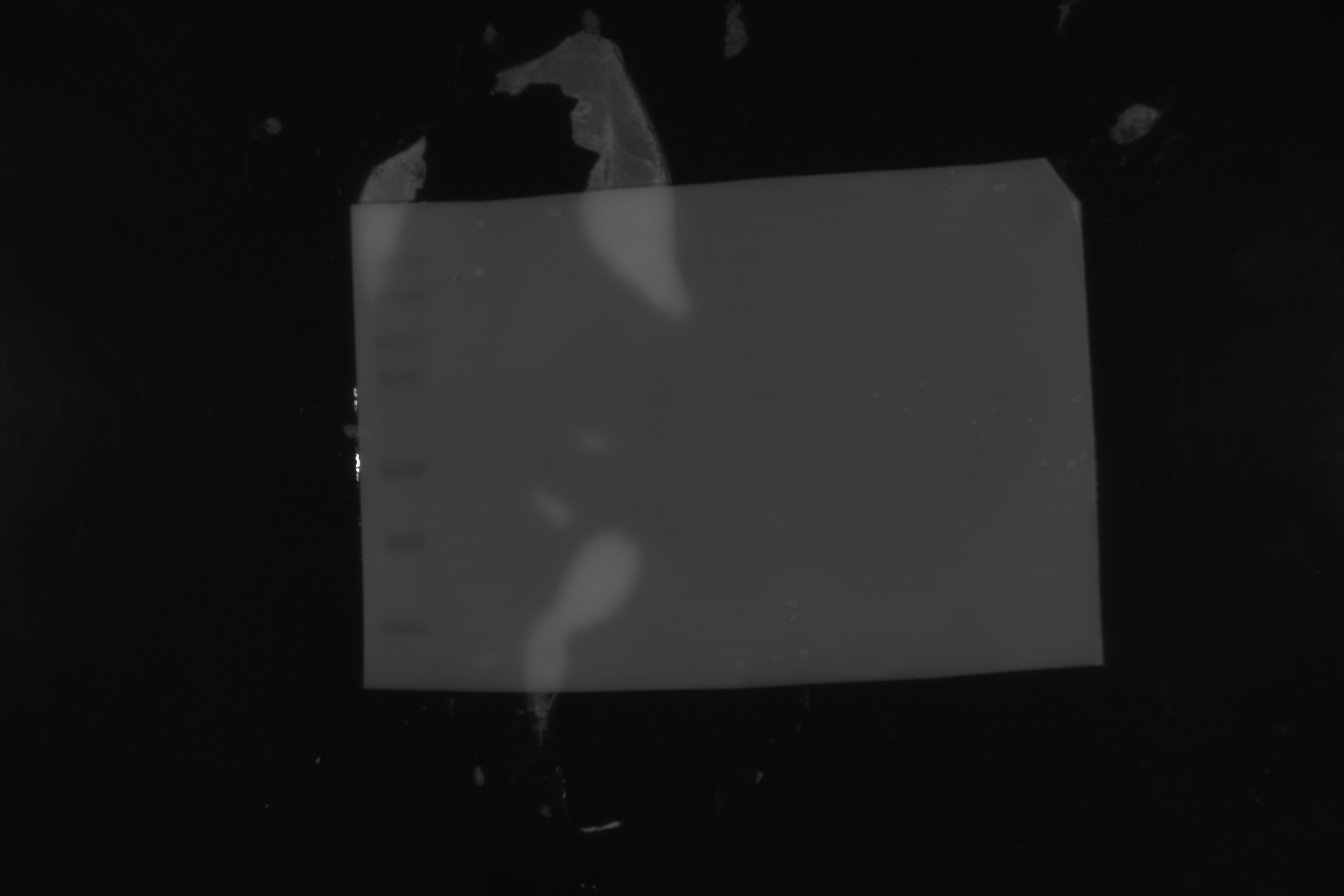

Supplement: Supplementary file 10 — Source data Fig. 8 [file 44318_2025_572_MOESM10_ESM.zip › Figure 8/Figure 8G/V_ORAI 3 4 SEC.gel]

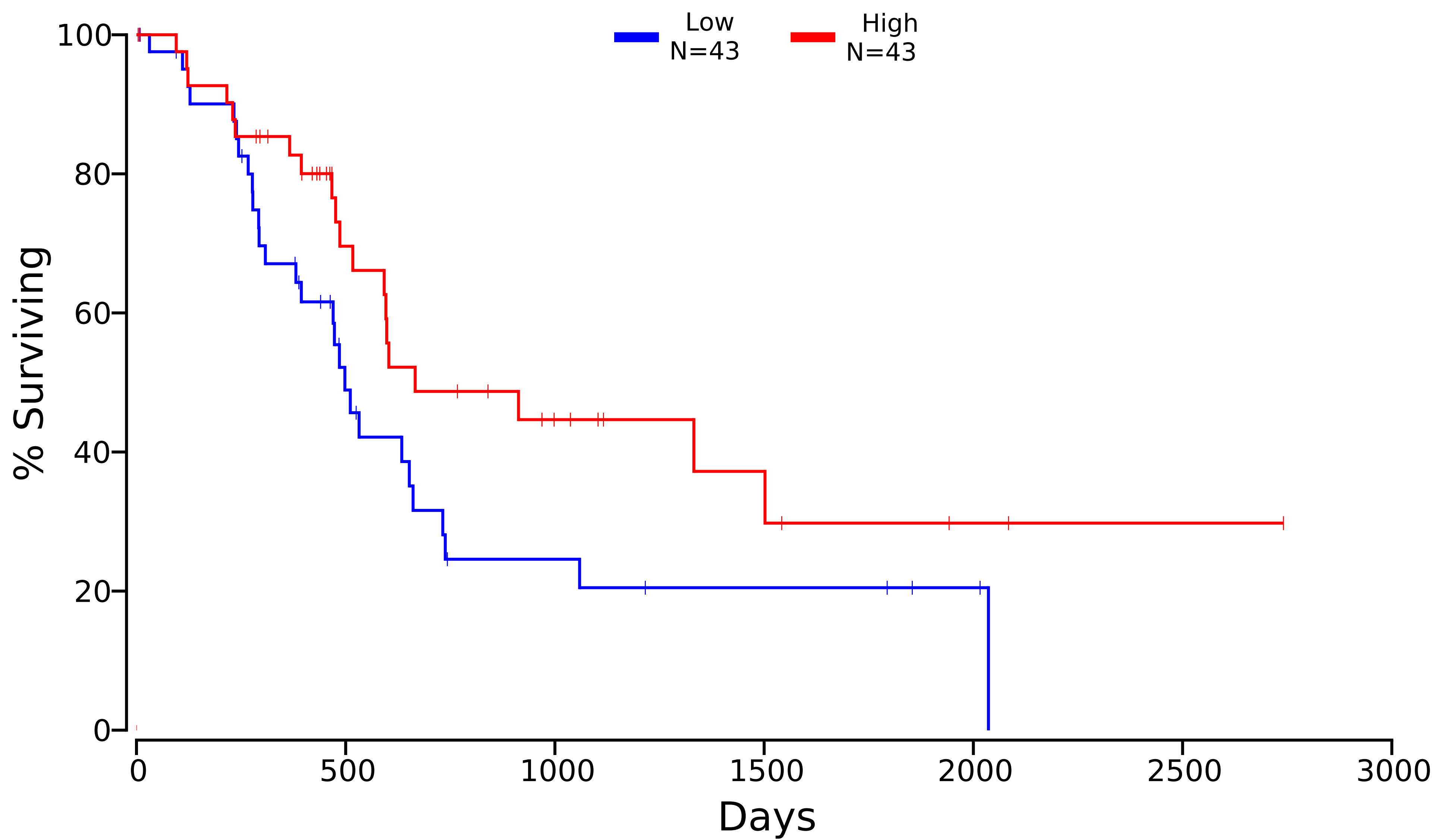

Supplement: Supplementary file 11 — Source data Fig. 9 [file 44318_2025_572_MOESM11_ESM.zip › Figure 9/Figure 9A/MARCH8 Survival.pdf]

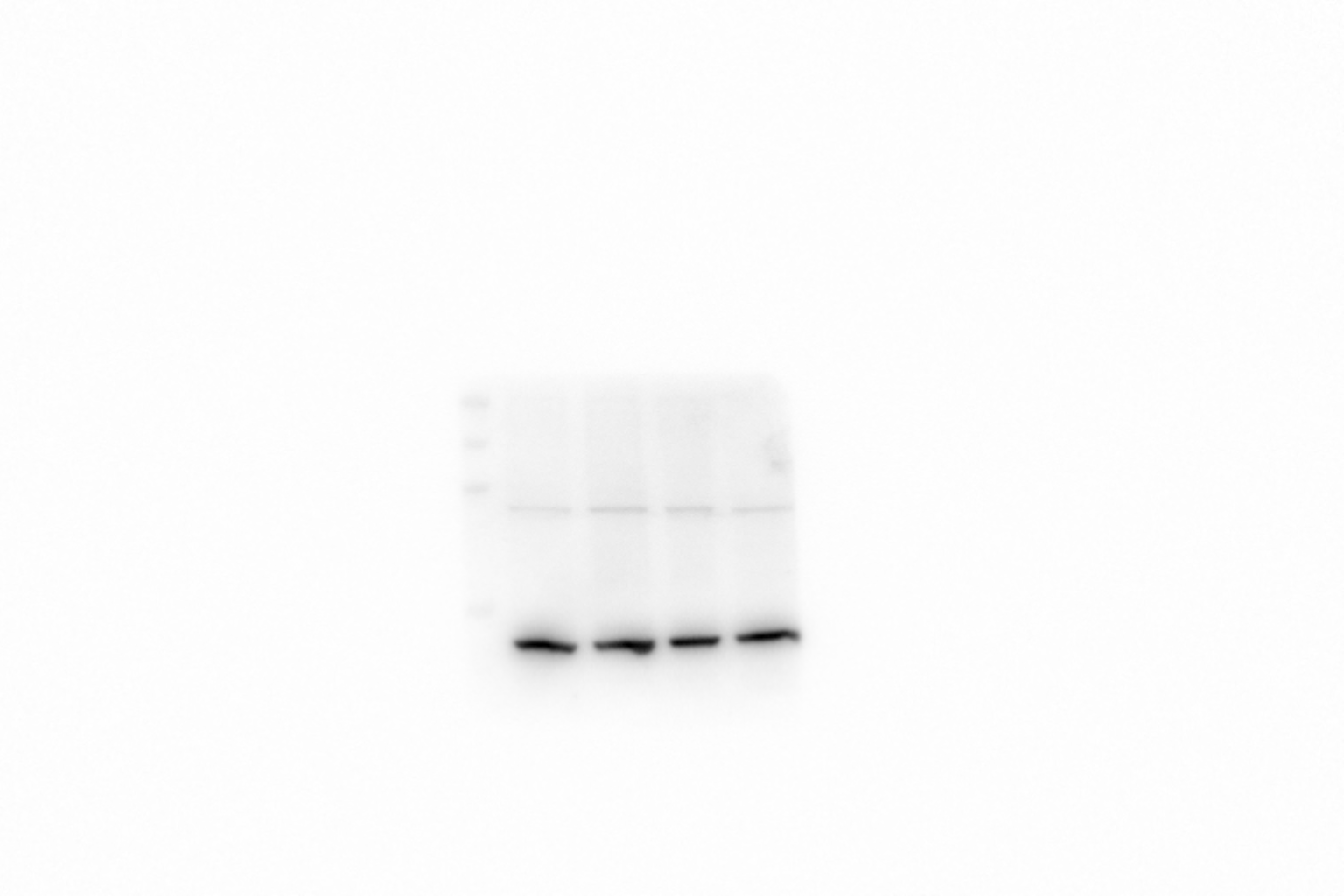

Supplement: Supplementary file 11 — Source data Fig. 9 [file 44318_2025_572_MOESM11_ESM.zip › Figure 9/Figure 9B/CFPAC1 shM8 B ACTIN.gel]

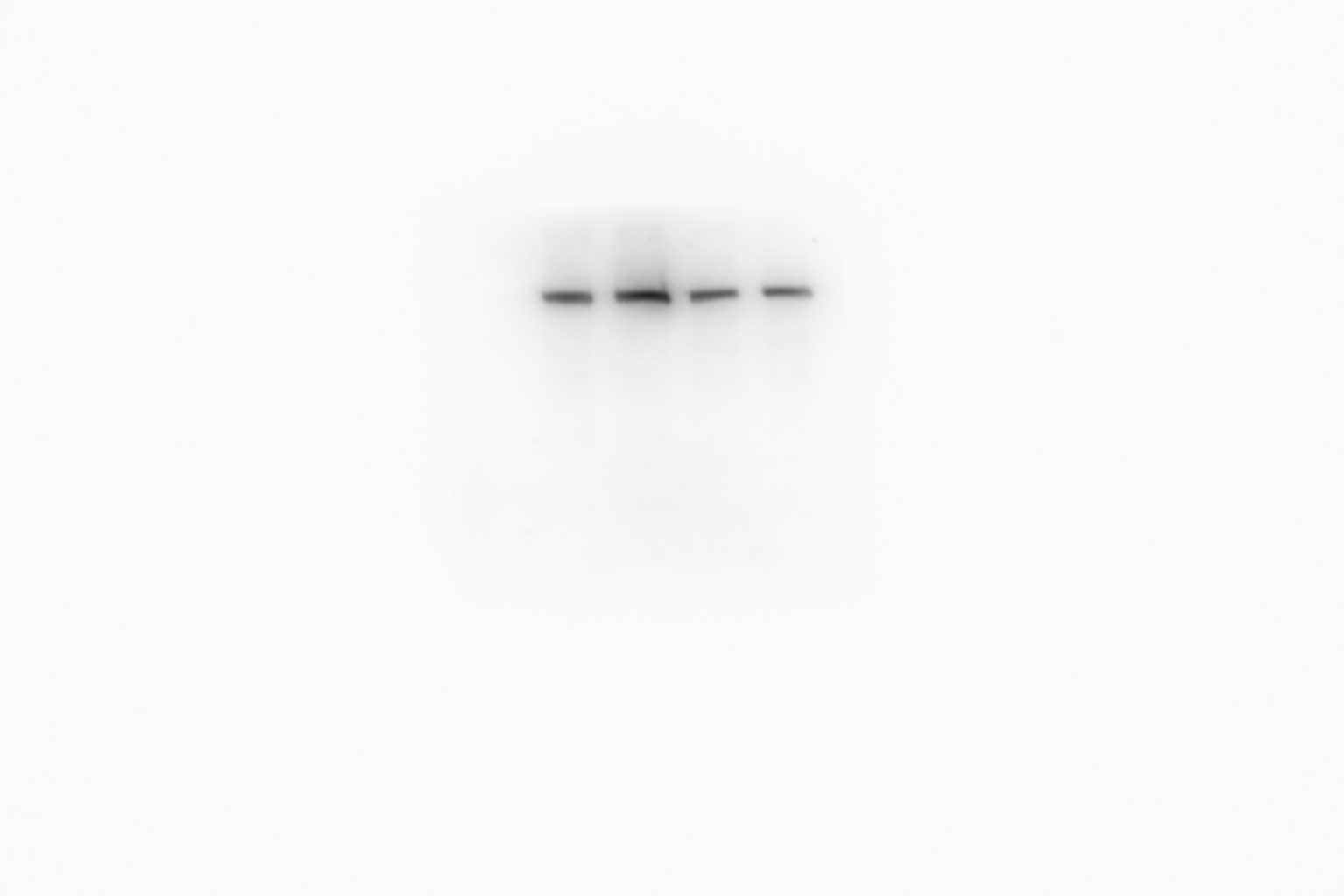

Supplement: Supplementary file 11 — Source data Fig. 9 [file 44318_2025_572_MOESM11_ESM.zip › Figure 9/Figure 9B/CFPAC1 shM8 M8.gel]

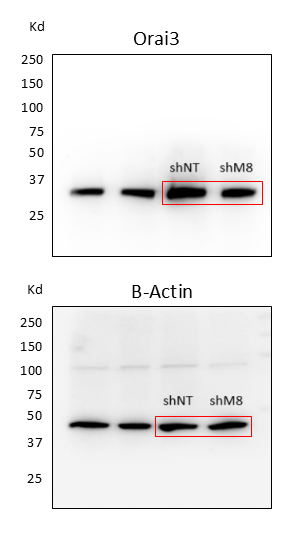

Supplement: Supplementary file 11 — Source data Fig. 9 [file 44318_2025_572_MOESM11_ESM.zip › Figure 9/Figure 9B/Figure 9B.png]

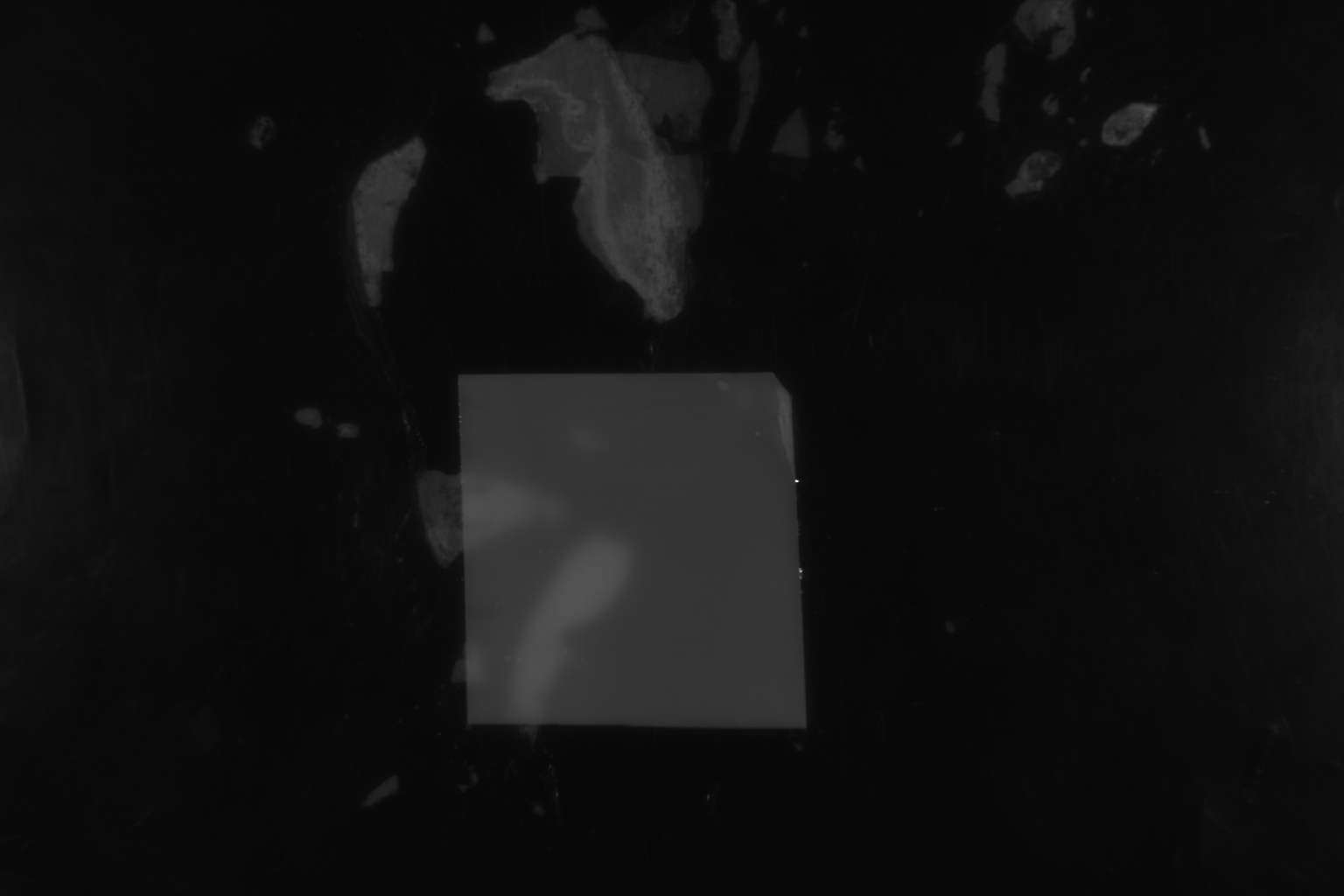

Supplement: Supplementary file 11 — Source data Fig. 9 [file 44318_2025_572_MOESM11_ESM.zip › Figure 9/Figure 9B/V_CFPAC1 shM8 B ACTIN.gel]

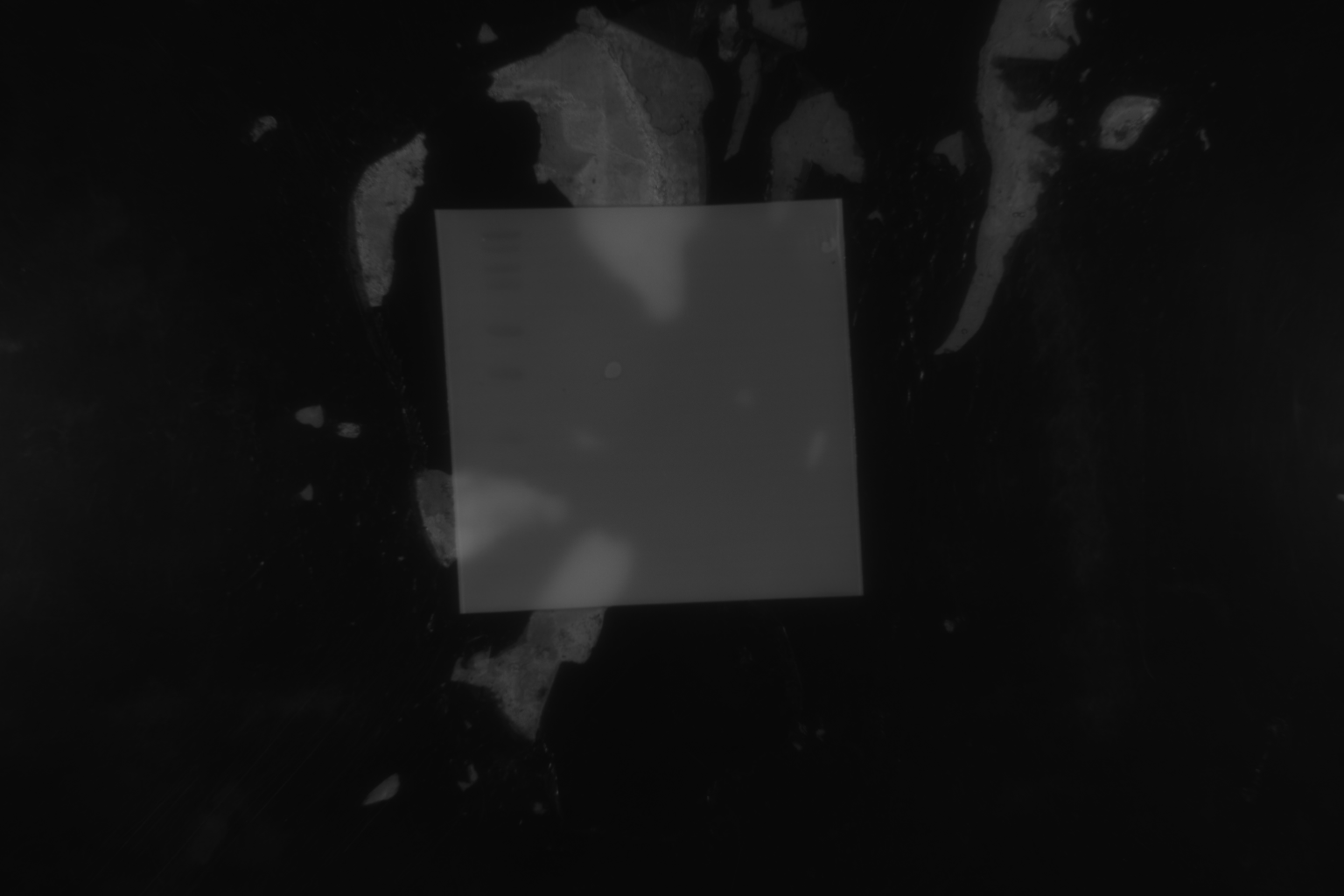

Supplement: Supplementary file 11 — Source data Fig. 9 [file 44318_2025_572_MOESM11_ESM.zip › Figure 9/Figure 9B/V_CFPAC1 shM8 M8.gel]

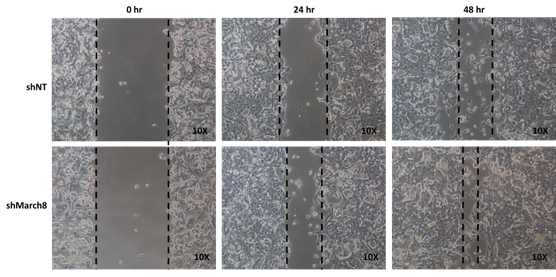

Supplement: Supplementary file 11 — Source data Fig. 9 [file 44318_2025_572_MOESM11_ESM.zip › Figure 9/Figure 9D/Figure 9D.png]

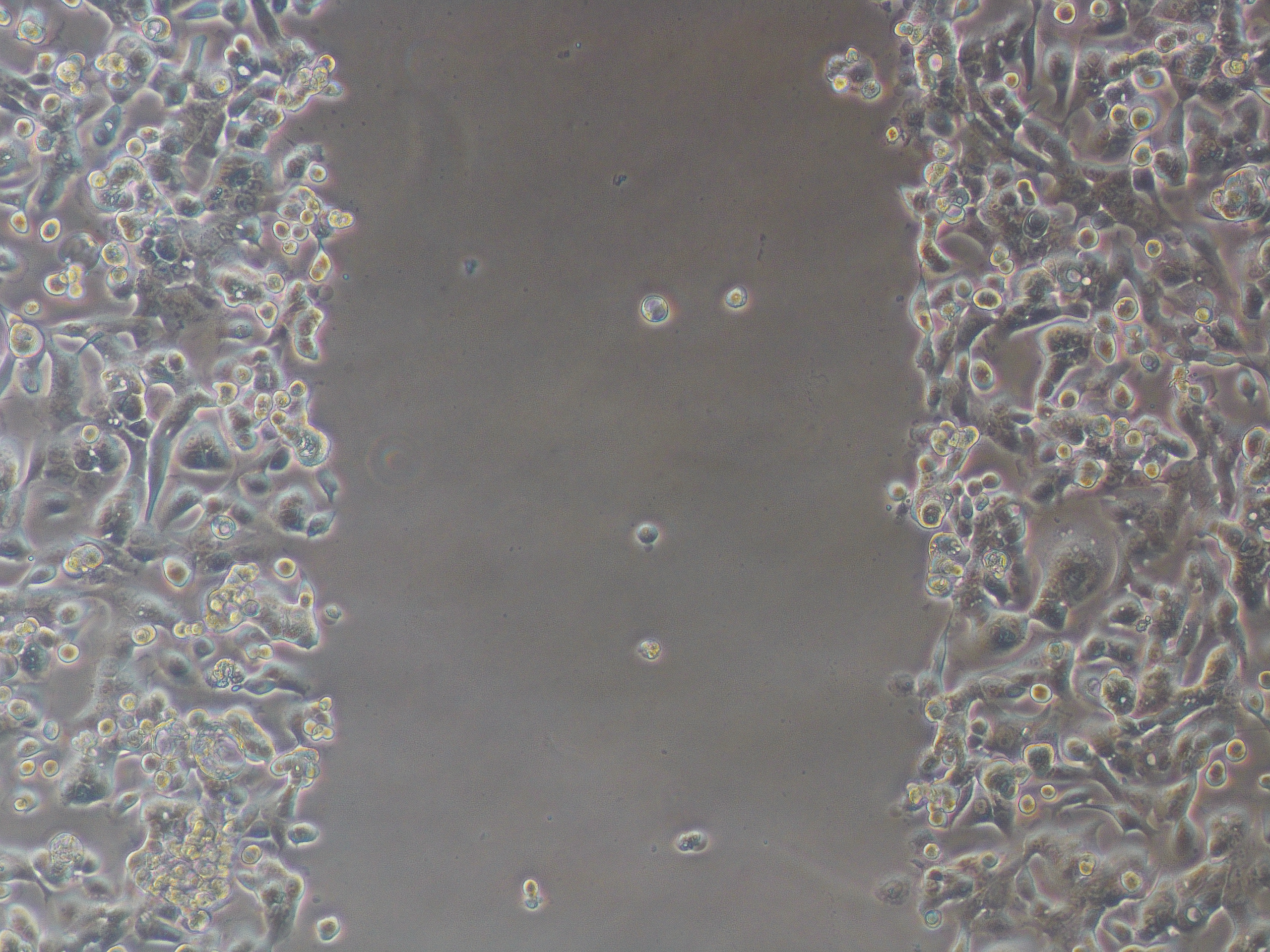

Supplement: Supplementary file 11 — Source data Fig. 9 [file 44318_2025_572_MOESM11_ESM.zip › Figure 9/Figure 9D/shMarch8 0 hr.tif]

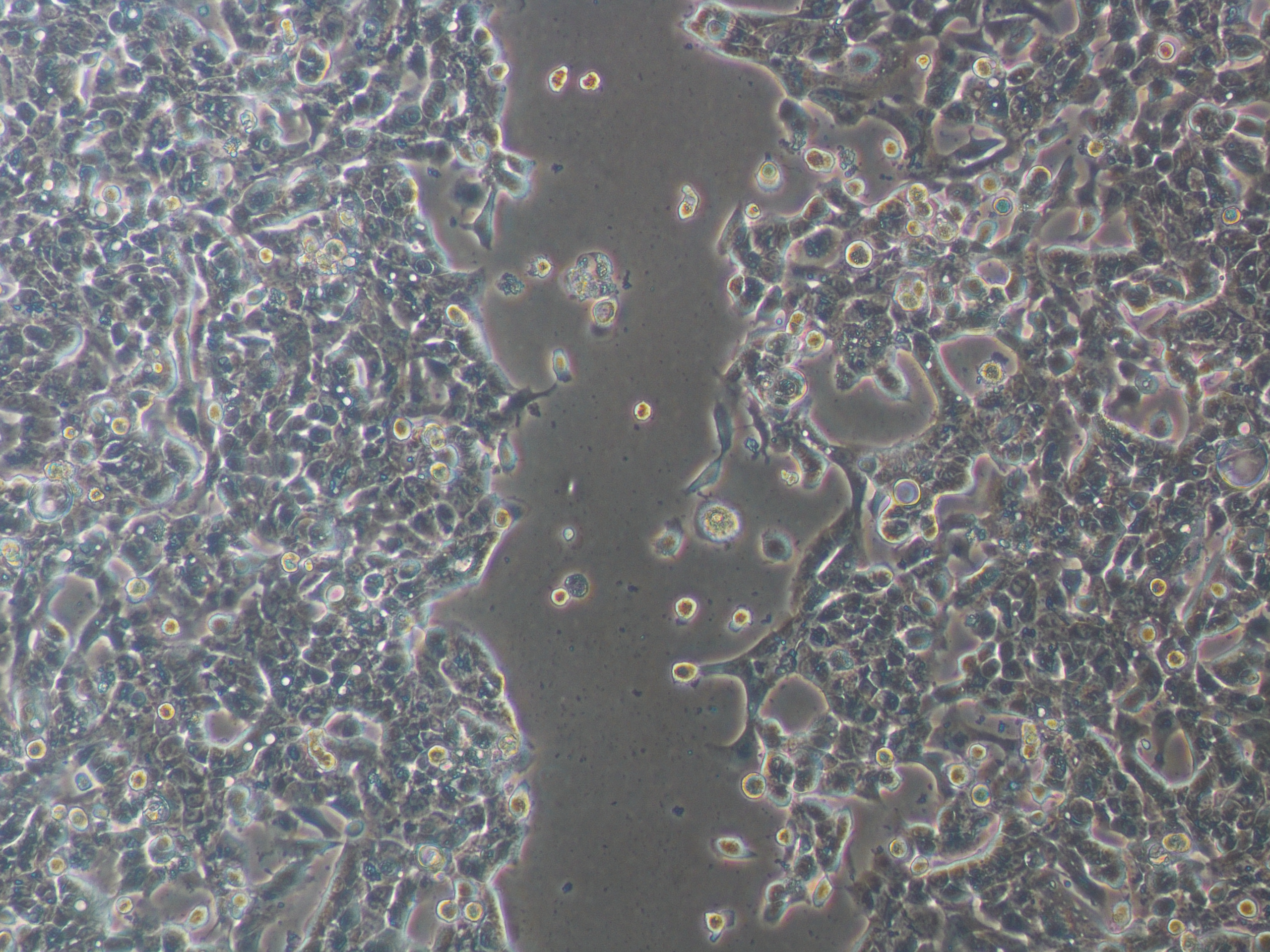

Supplement: Supplementary file 11 — Source data Fig. 9 [file 44318_2025_572_MOESM11_ESM.zip › Figure 9/Figure 9D/shMarch8 24 hr.tif]

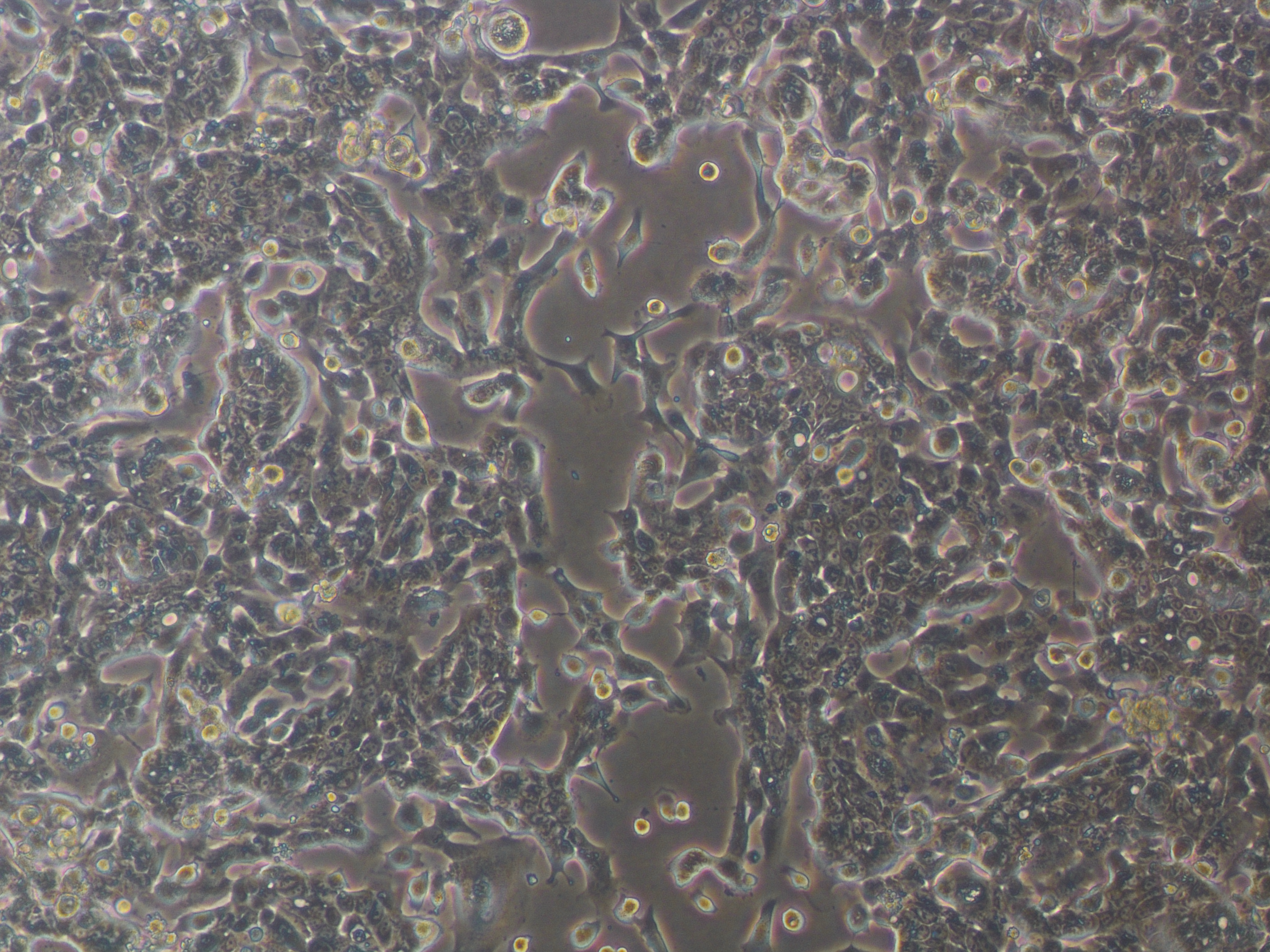

Supplement: Supplementary file 11 — Source data Fig. 9 [file 44318_2025_572_MOESM11_ESM.zip › Figure 9/Figure 9D/shMarch8 48 hr.tif]

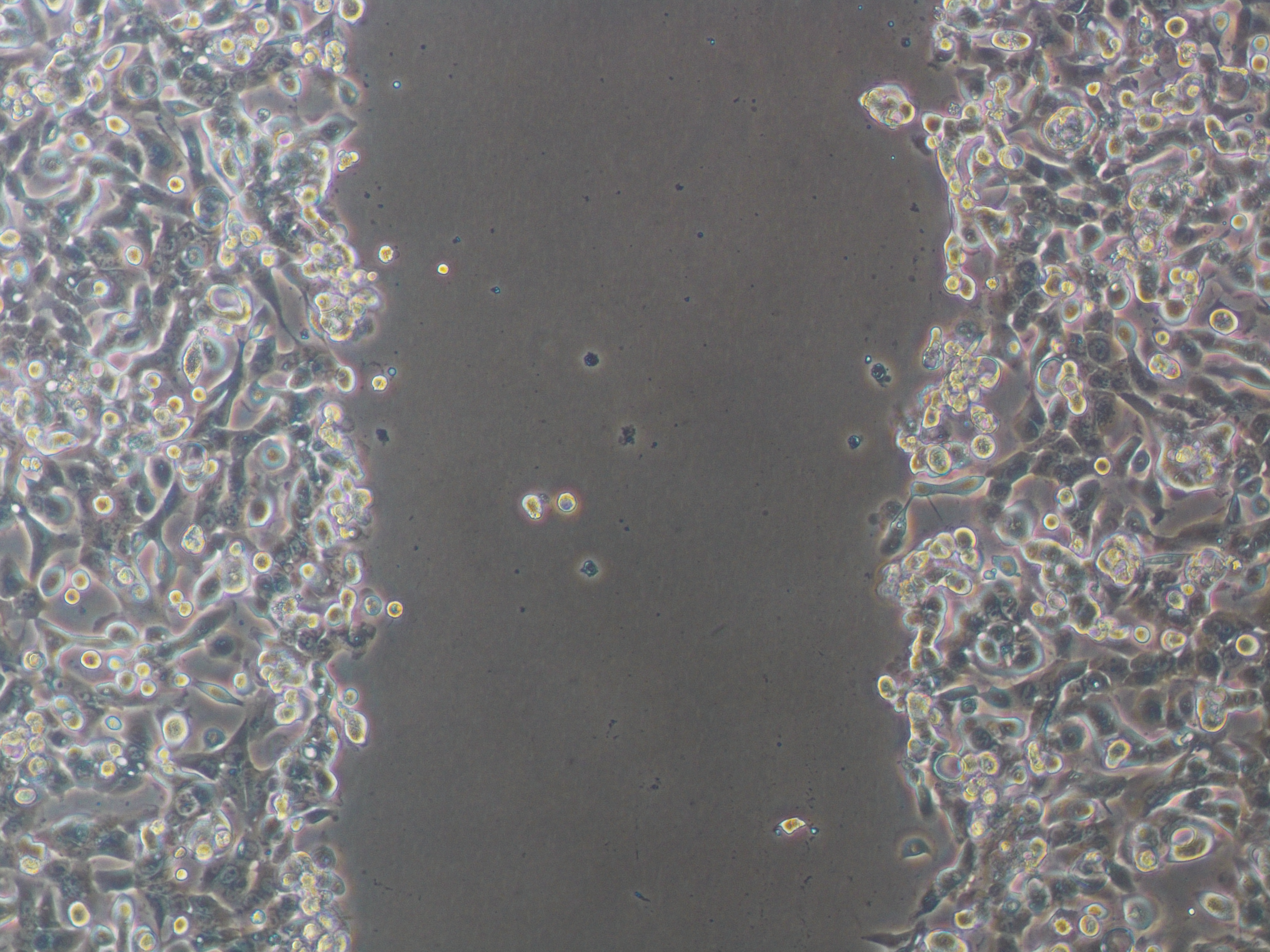

Supplement: Supplementary file 11 — Source data Fig. 9 [file 44318_2025_572_MOESM11_ESM.zip › Figure 9/Figure 9D/shNT 0 hr.tif]

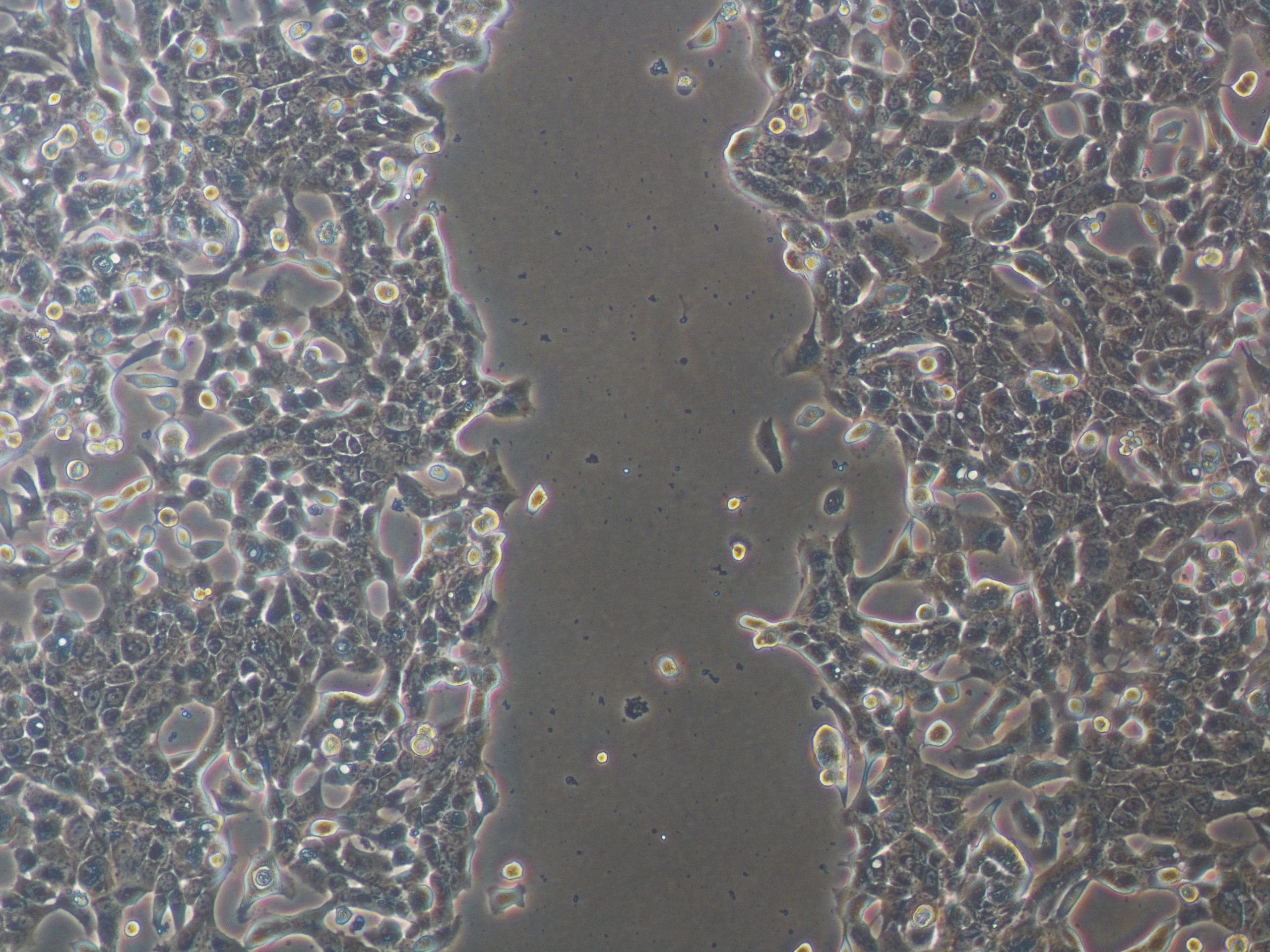

Supplement: Supplementary file 11 — Source data Fig. 9 [file 44318_2025_572_MOESM11_ESM.zip › Figure 9/Figure 9D/shNT 24 hr.tif]

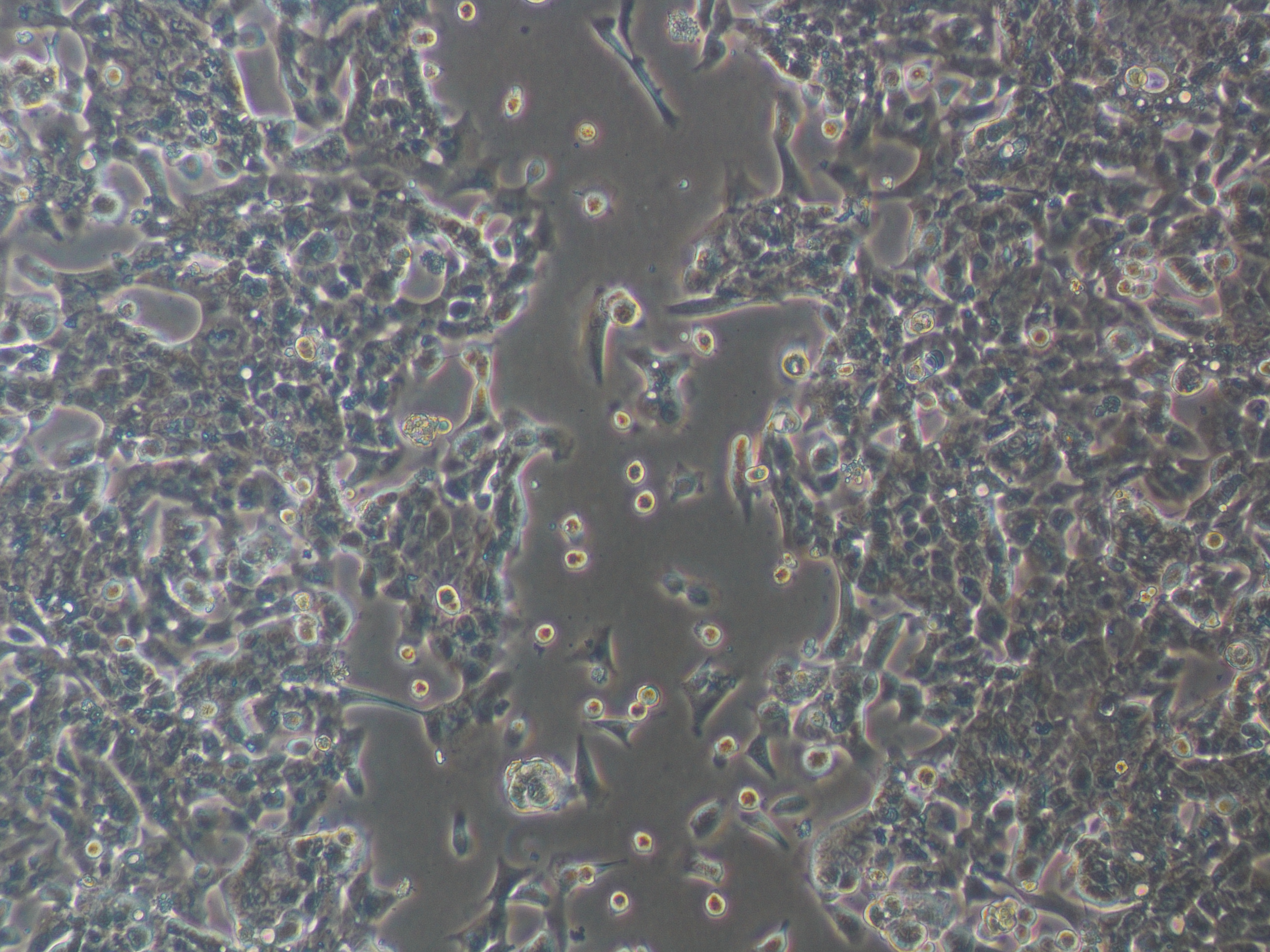

Supplement: Supplementary file 11 — Source data Fig. 9 [file 44318_2025_572_MOESM11_ESM.zip › Figure 9/Figure 9D/shNT 48 hr.tif]

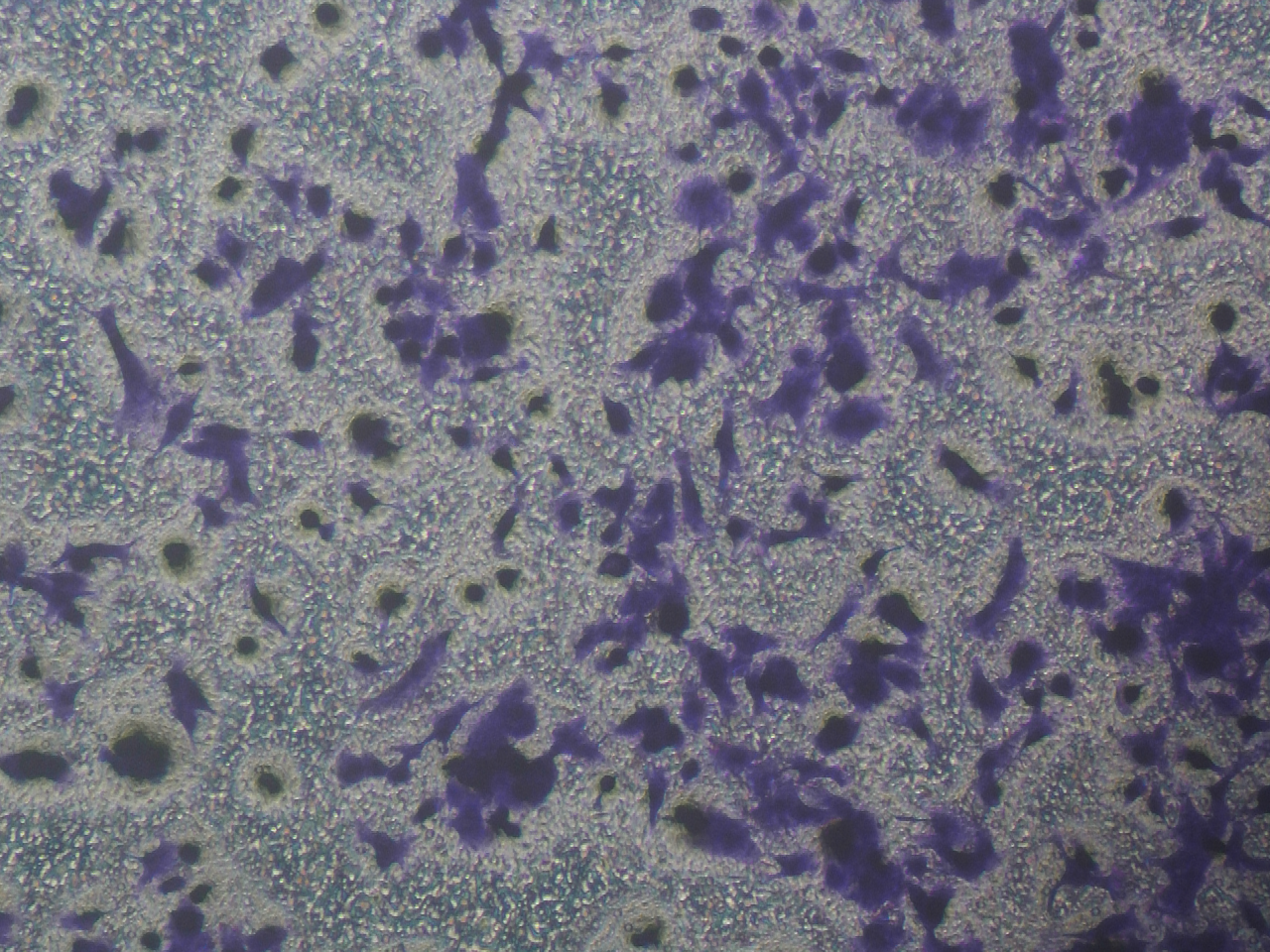

Supplement: Supplementary file 11 — Source data Fig. 9 [file 44318_2025_572_MOESM11_ESM.zip › Figure 9/Figure 9F/EV.tif]

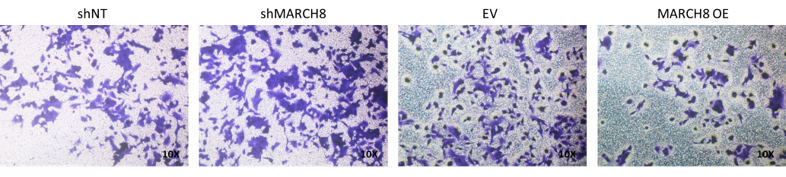

Supplement: Supplementary file 11 — Source data Fig. 9 [file 44318_2025_572_MOESM11_ESM.zip › Figure 9/Figure 9F/Figure 9F.png]

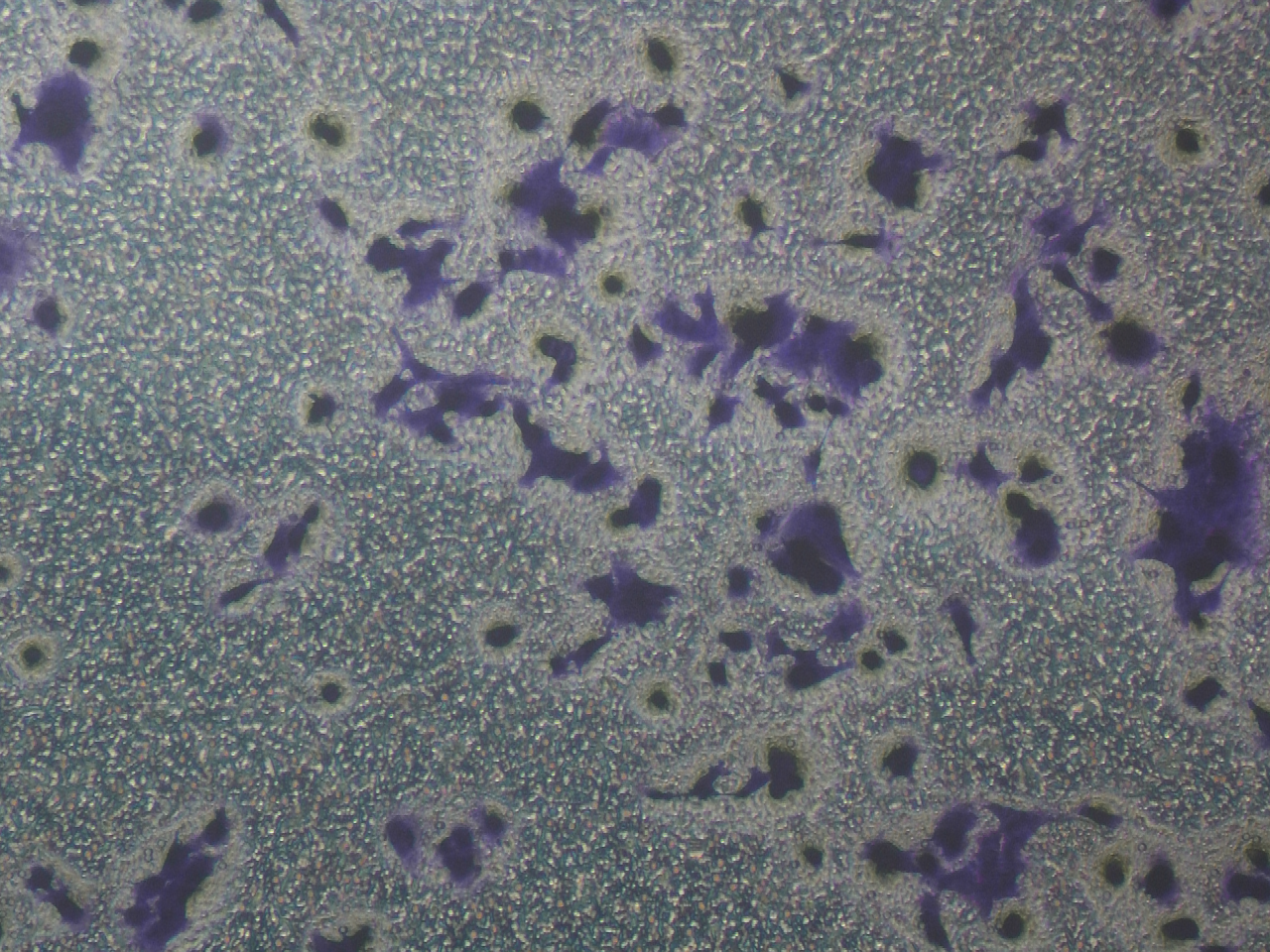

Supplement: Supplementary file 11 — Source data Fig. 9 [file 44318_2025_572_MOESM11_ESM.zip › Figure 9/Figure 9F/MARCH8 OE.tif]

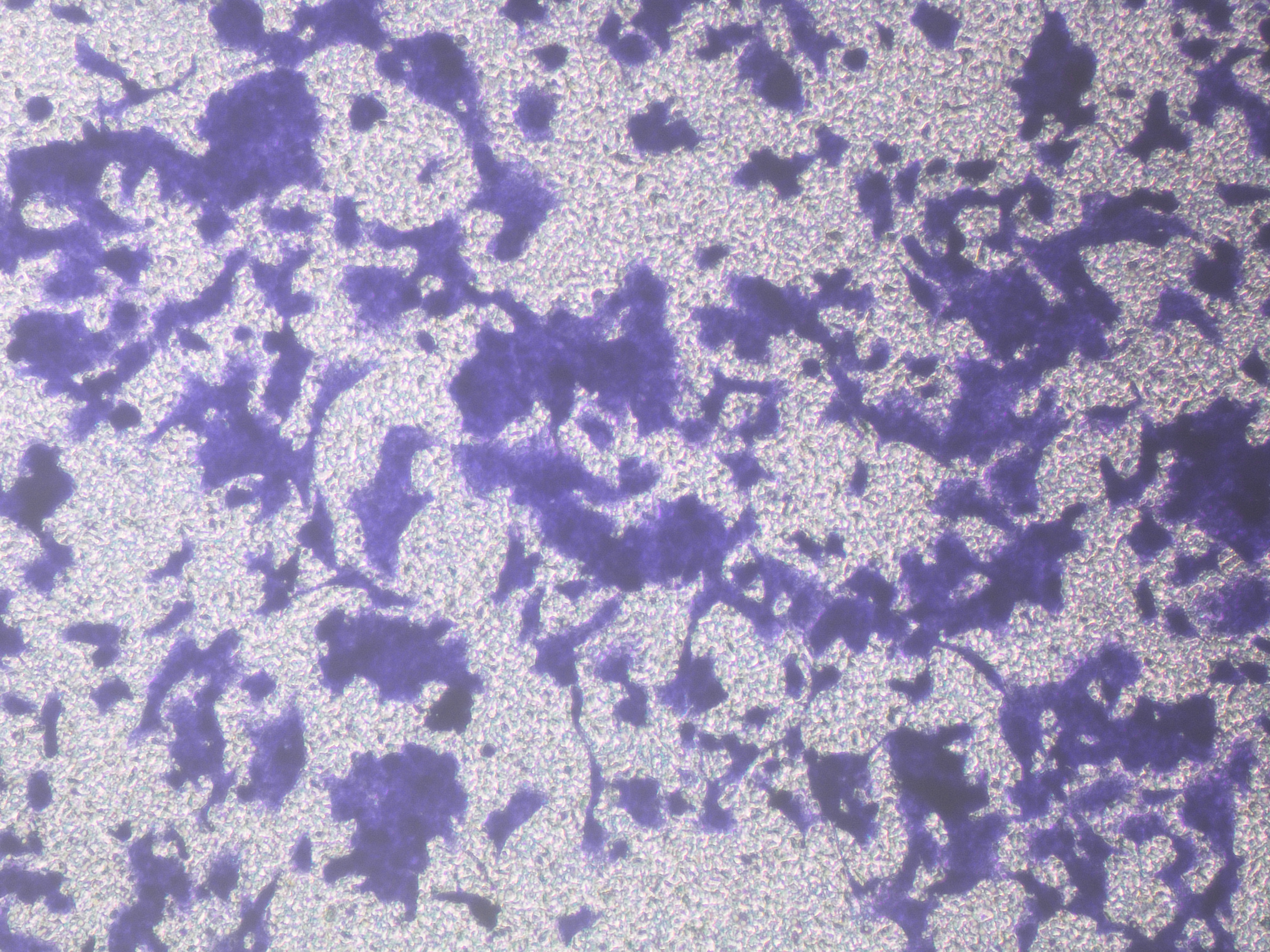

Supplement: Supplementary file 11 — Source data Fig. 9 [file 44318_2025_572_MOESM11_ESM.zip › Figure 9/Figure 9F/shM8.tif]

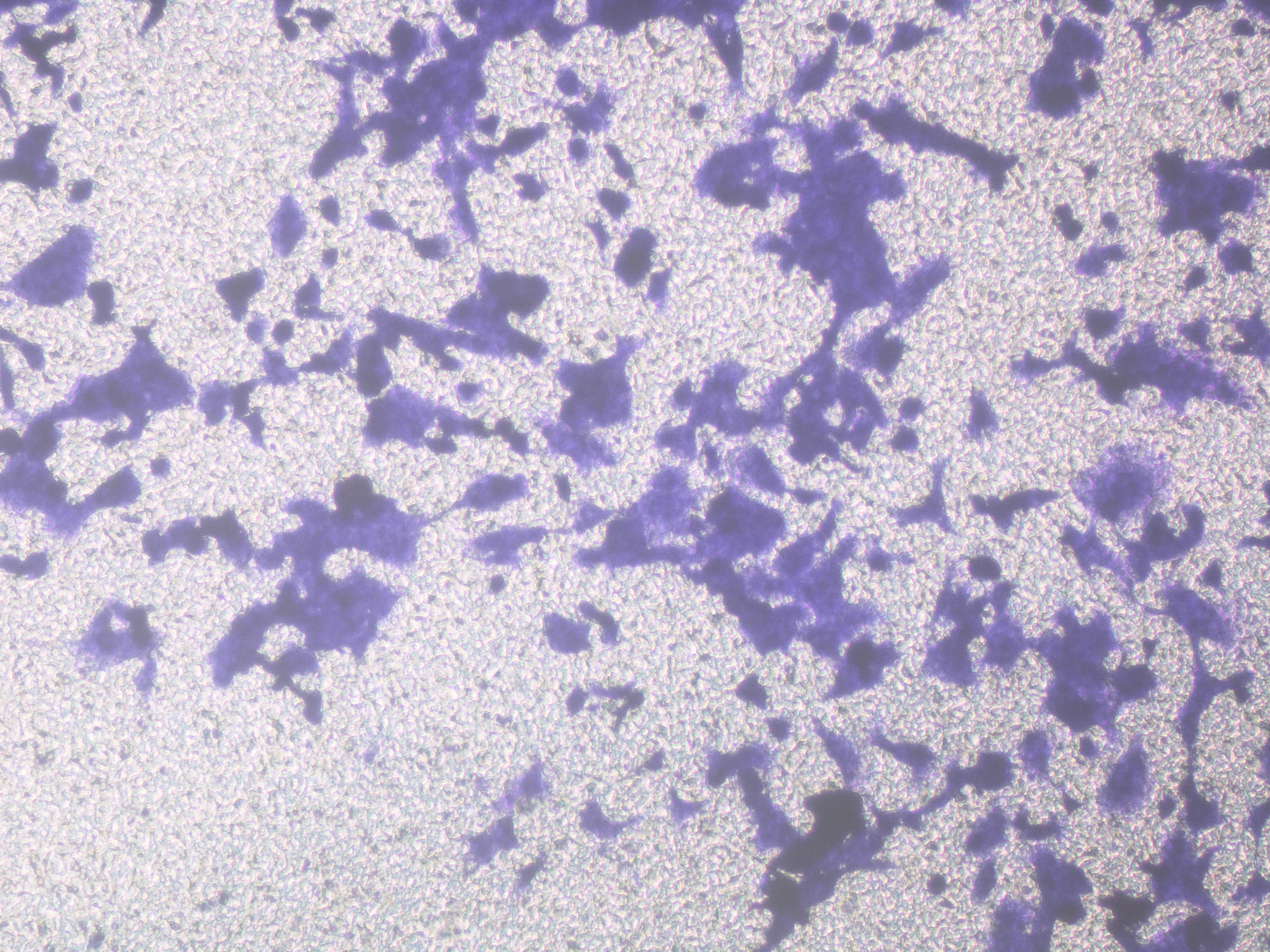

Supplement: Supplementary file 11 — Source data Fig. 9 [file 44318_2025_572_MOESM11_ESM.zip › Figure 9/Figure 9F/shNT.tif]

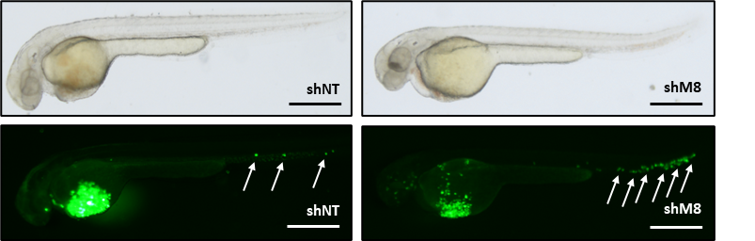

Supplement: Supplementary file 11 — Source data Fig. 9 [file 44318_2025_572_MOESM11_ESM.zip › Figure 9/Figure 9I/Figure 9I.png]

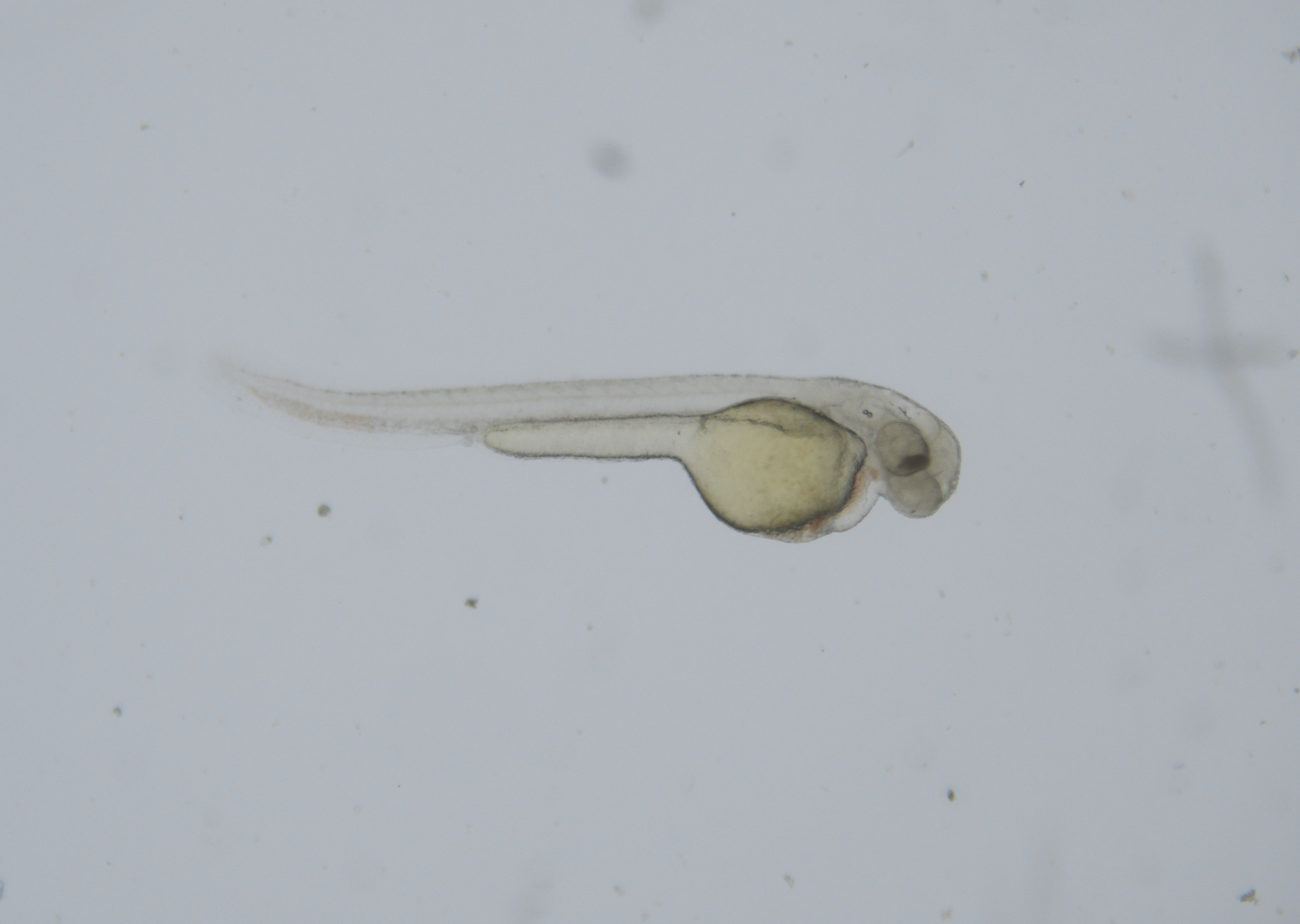

Supplement: Supplementary file 11 — Source data Fig. 9 [file 44318_2025_572_MOESM11_ESM.zip › Figure 9/Figure 9I/shM8 Bright Field.tif]

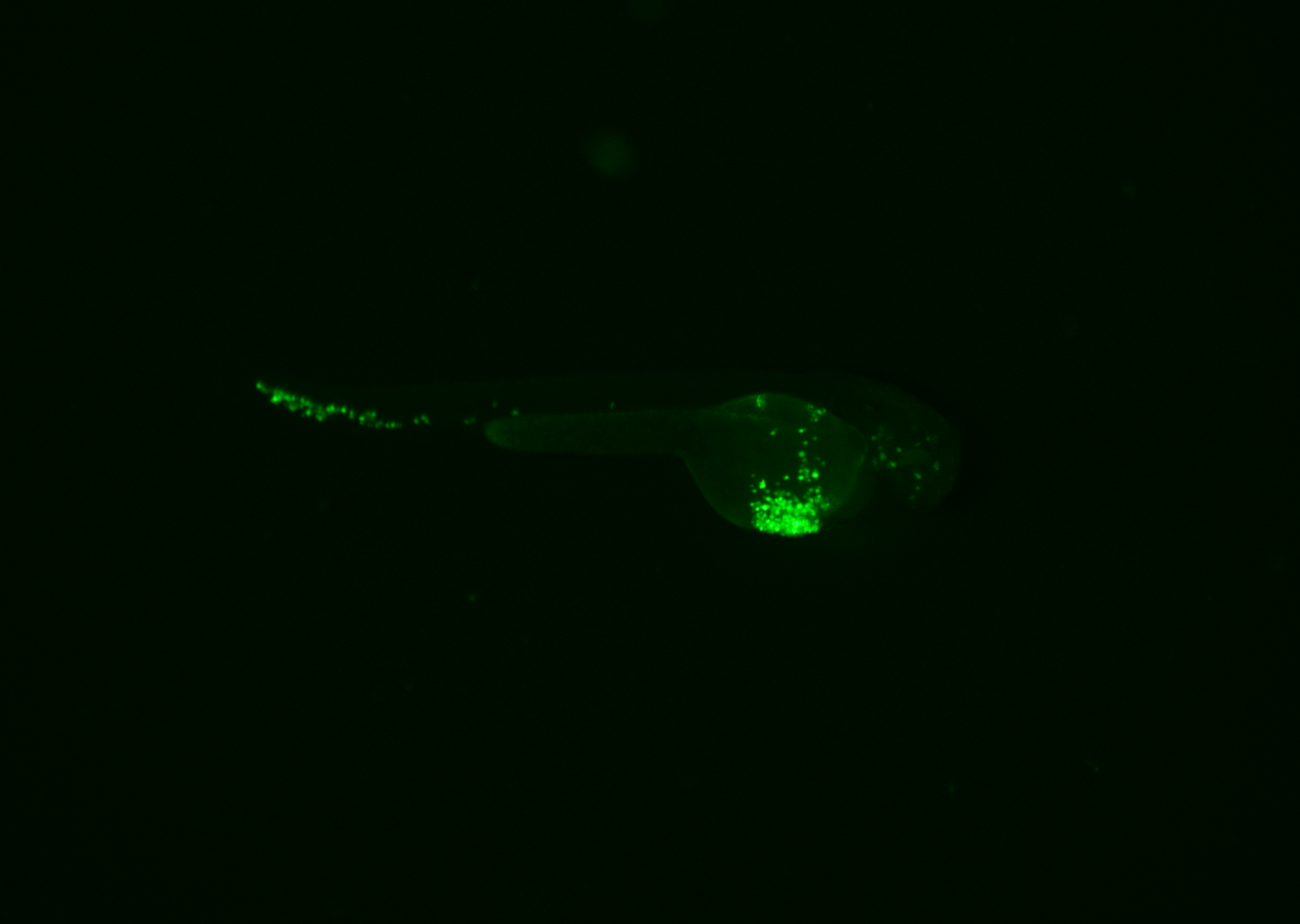

Supplement: Supplementary file 11 — Source data Fig. 9 [file 44318_2025_572_MOESM11_ESM.zip › Figure 9/Figure 9I/shM8.tif]

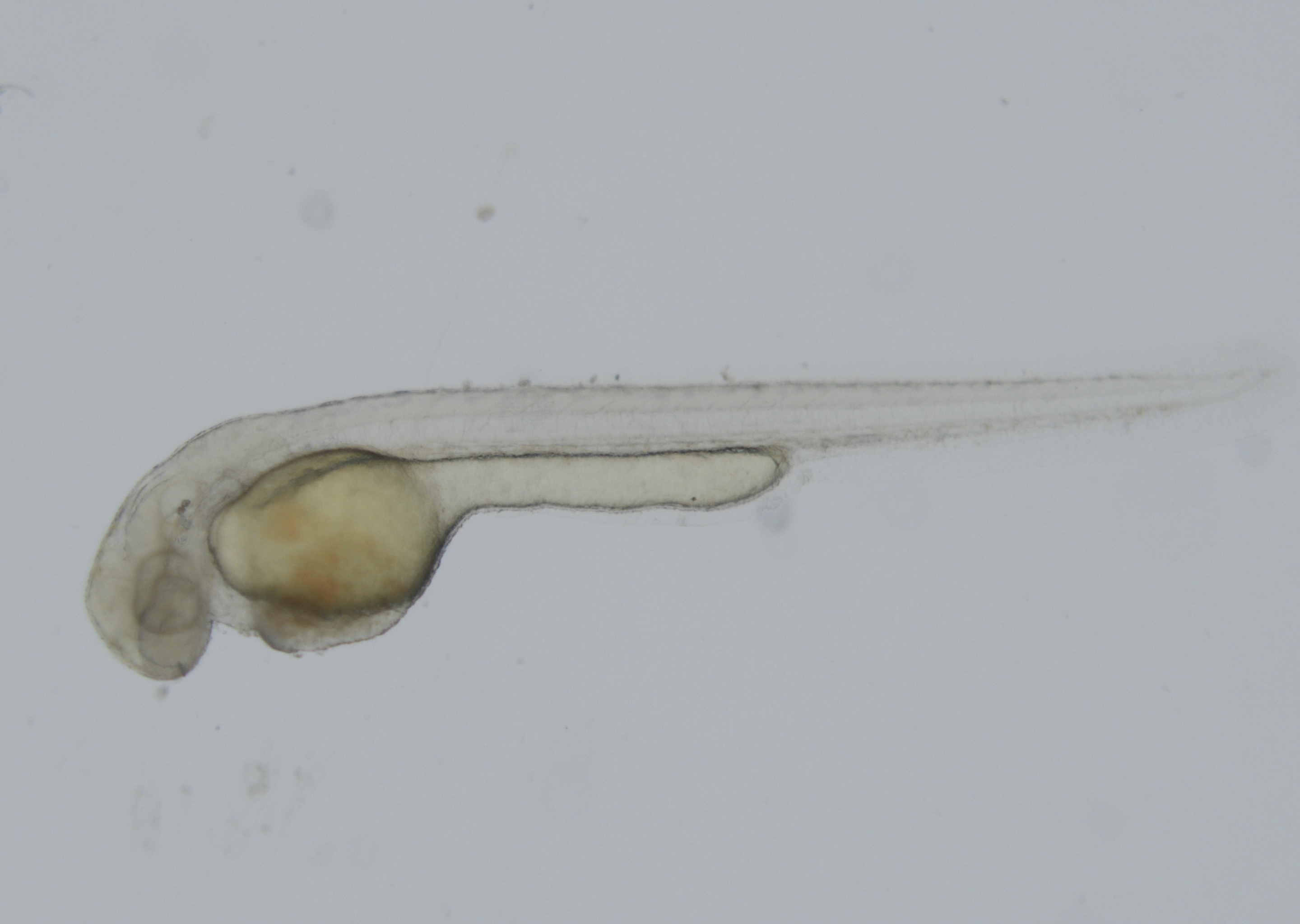

Supplement: Supplementary file 11 — Source data Fig. 9 [file 44318_2025_572_MOESM11_ESM.zip › Figure 9/Figure 9I/shNT Bright Field.tif]

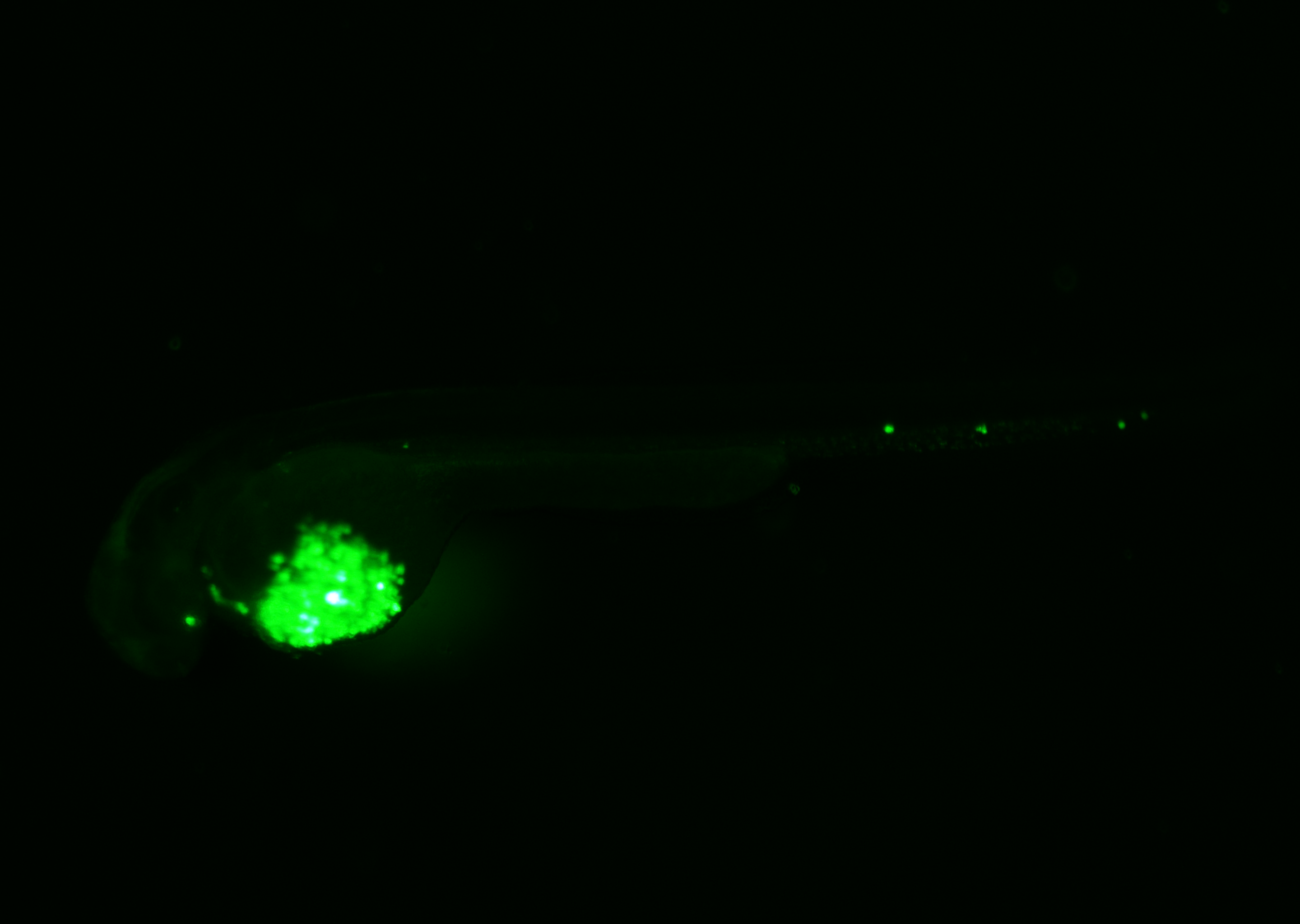

Supplement: Supplementary file 11 — Source data Fig. 9 [file 44318_2025_572_MOESM11_ESM.zip › Figure 9/Figure 9I/shNT.tif]

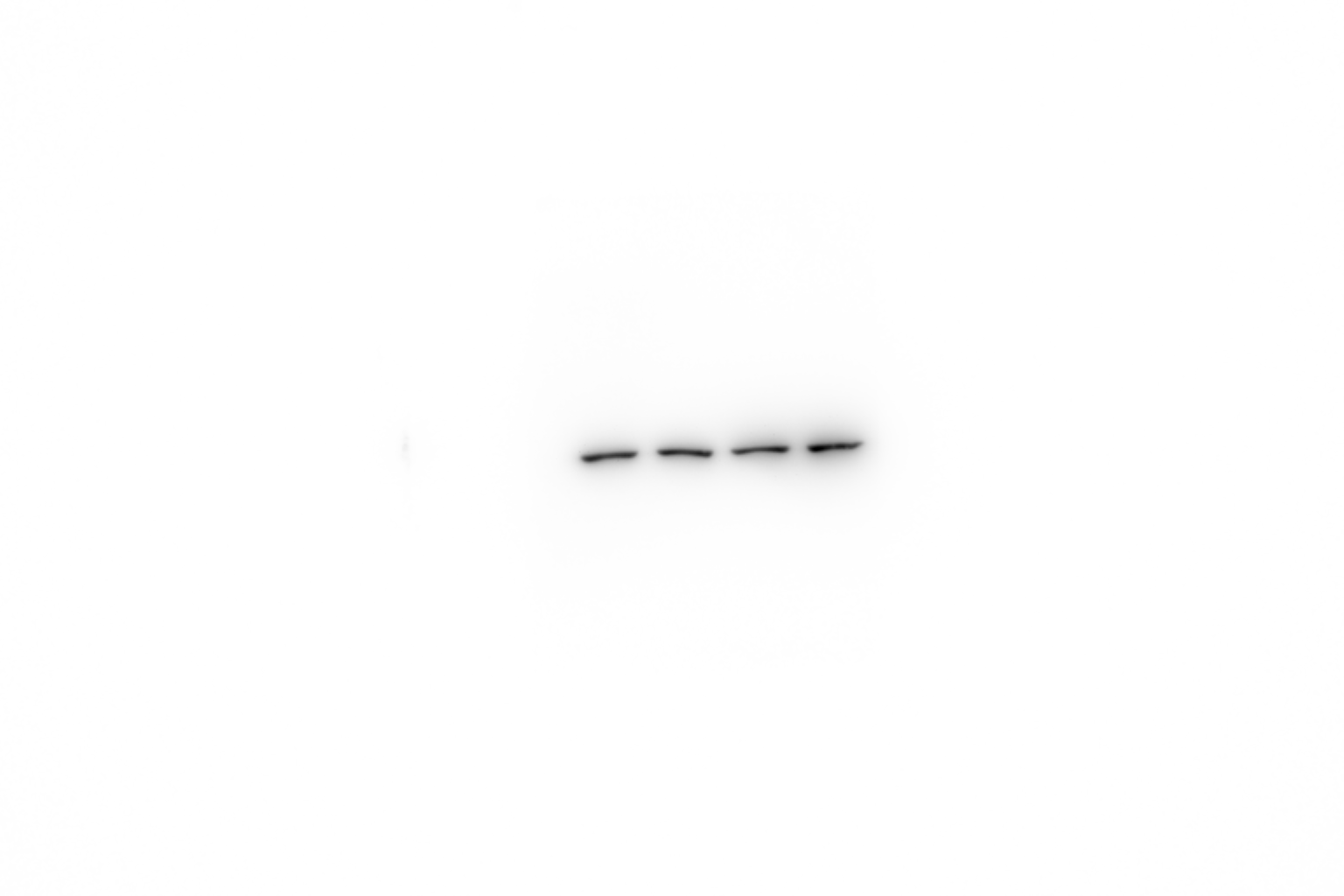

Supplement: Supplementary file 13 — Figure EV2 Source Data [file 44318_2025_572_MOESM13_ESM.zip › EV 2/EV 2J/B ACTIN 240 SEC.gel]

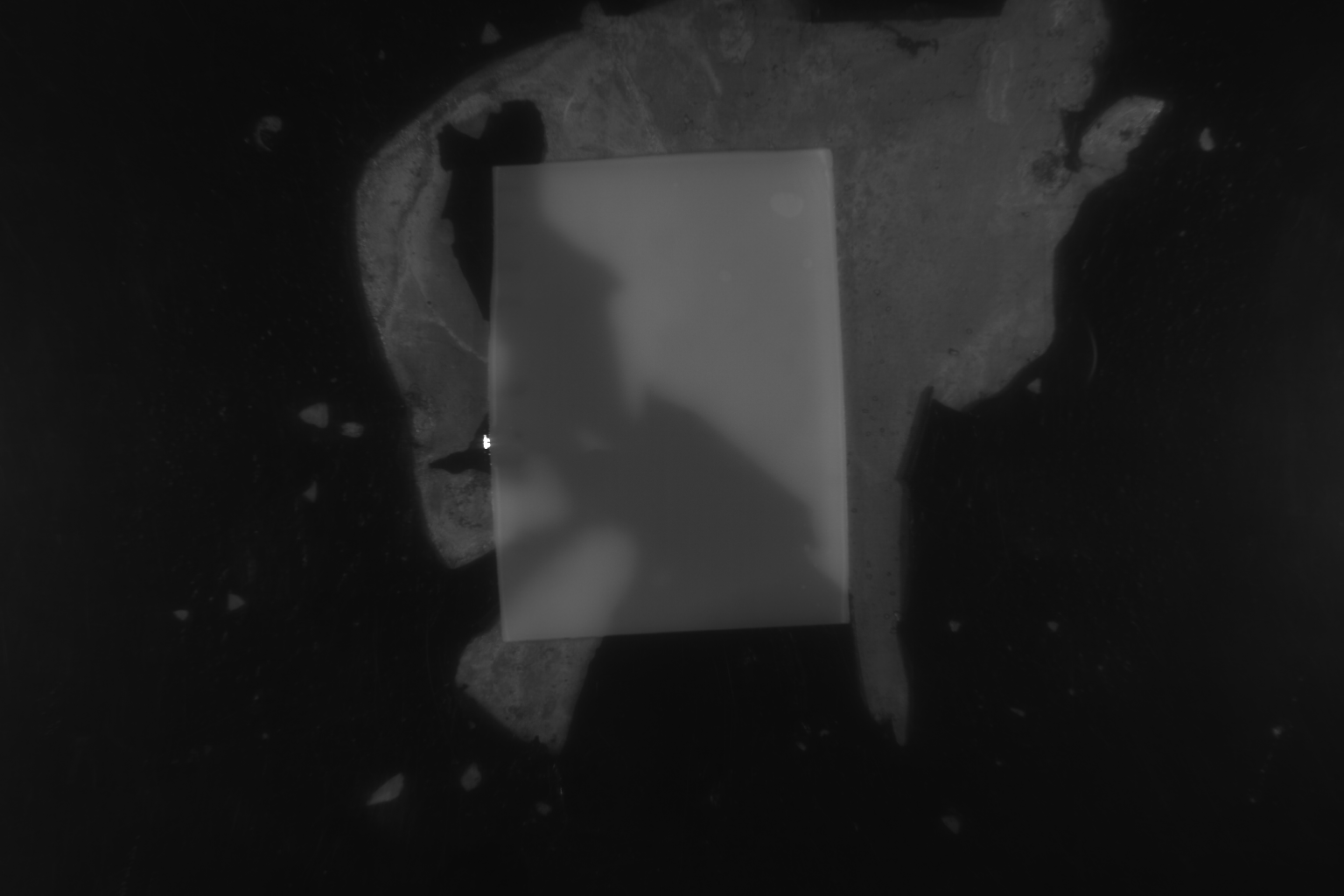

Supplement: Supplementary file 13 — Figure EV2 Source Data [file 44318_2025_572_MOESM13_ESM.zip › EV 2/EV 2J/ORAI 3 240 SEC (2).gel]

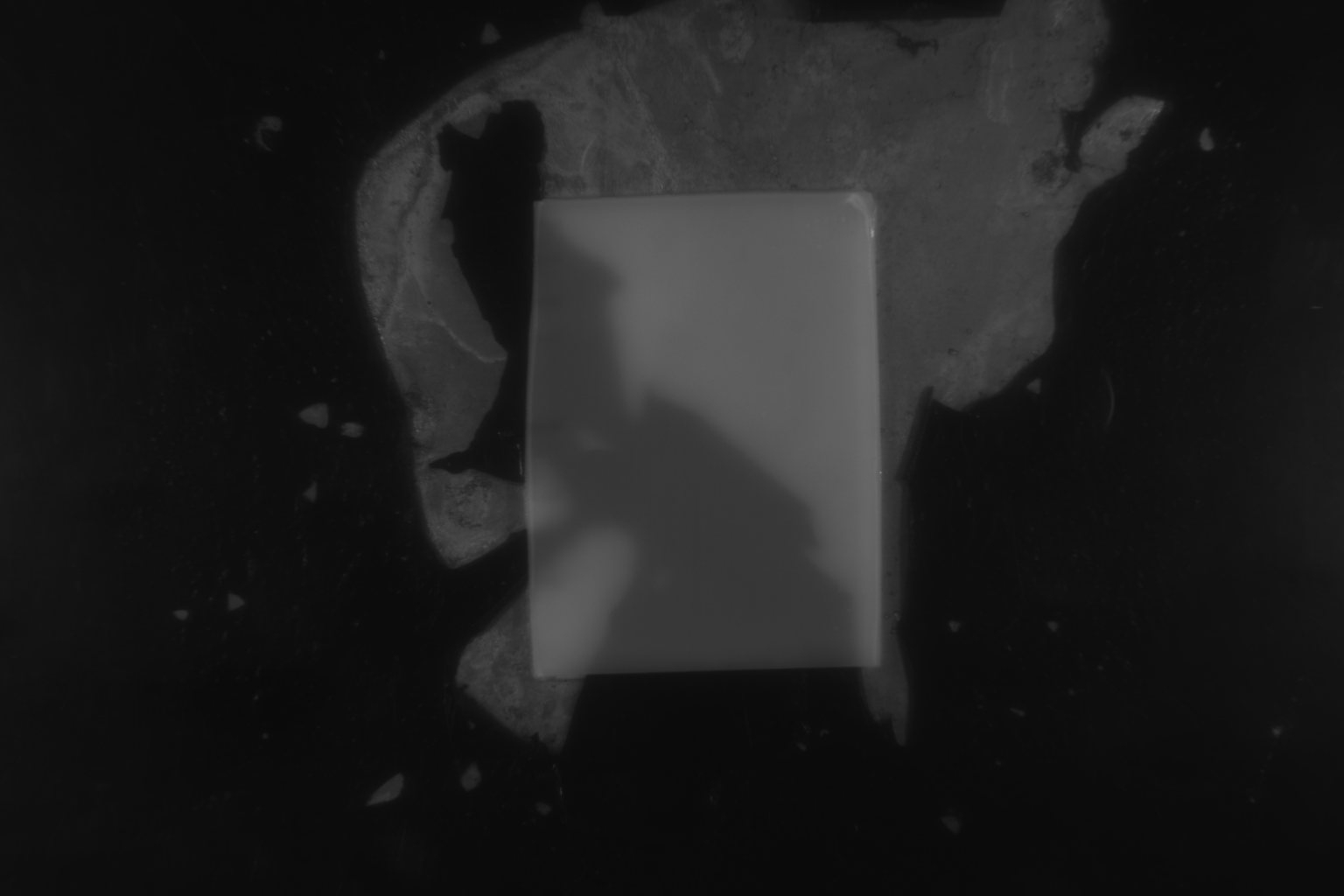

Supplement: Supplementary file 13 — Figure EV2 Source Data [file 44318_2025_572_MOESM13_ESM.zip › EV 2/EV 2J/V_B ACTIN 240 SEC.gel]

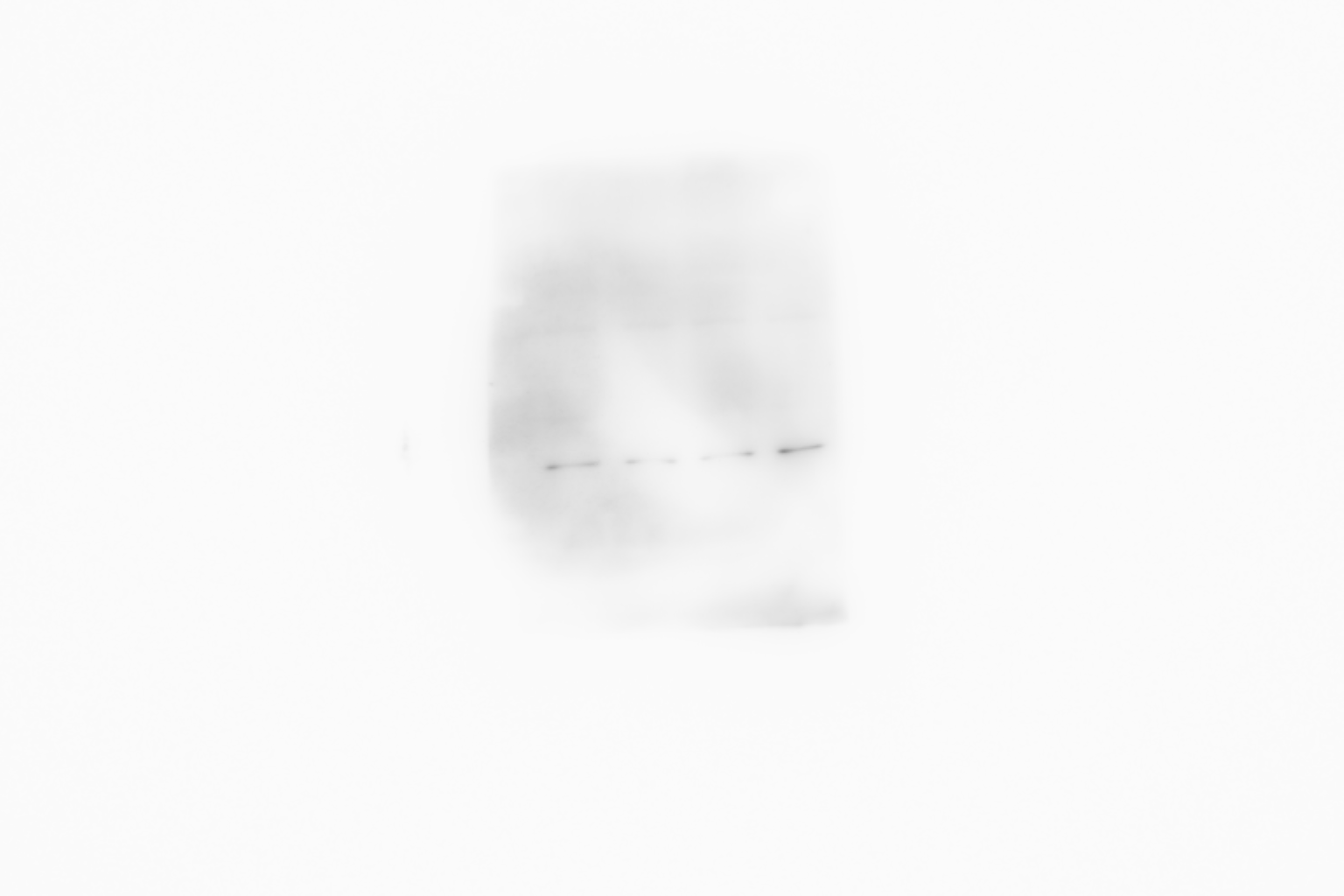

Supplement: Supplementary file 13 — Figure EV2 Source Data [file 44318_2025_572_MOESM13_ESM.zip › EV 2/EV 2J/ORAI 3 240 SEC (1).gel]

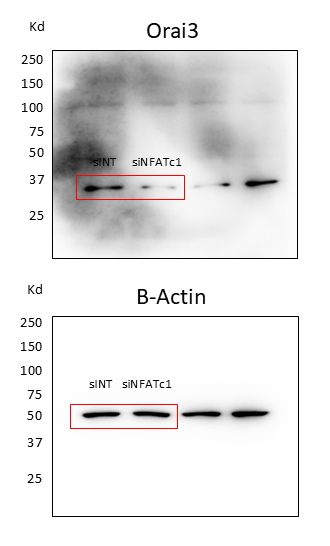

Supplement: Supplementary file 13 — Figure EV2 Source Data [file 44318_2025_572_MOESM13_ESM.zip › EV 2/EV 2J/EV 2J.png]

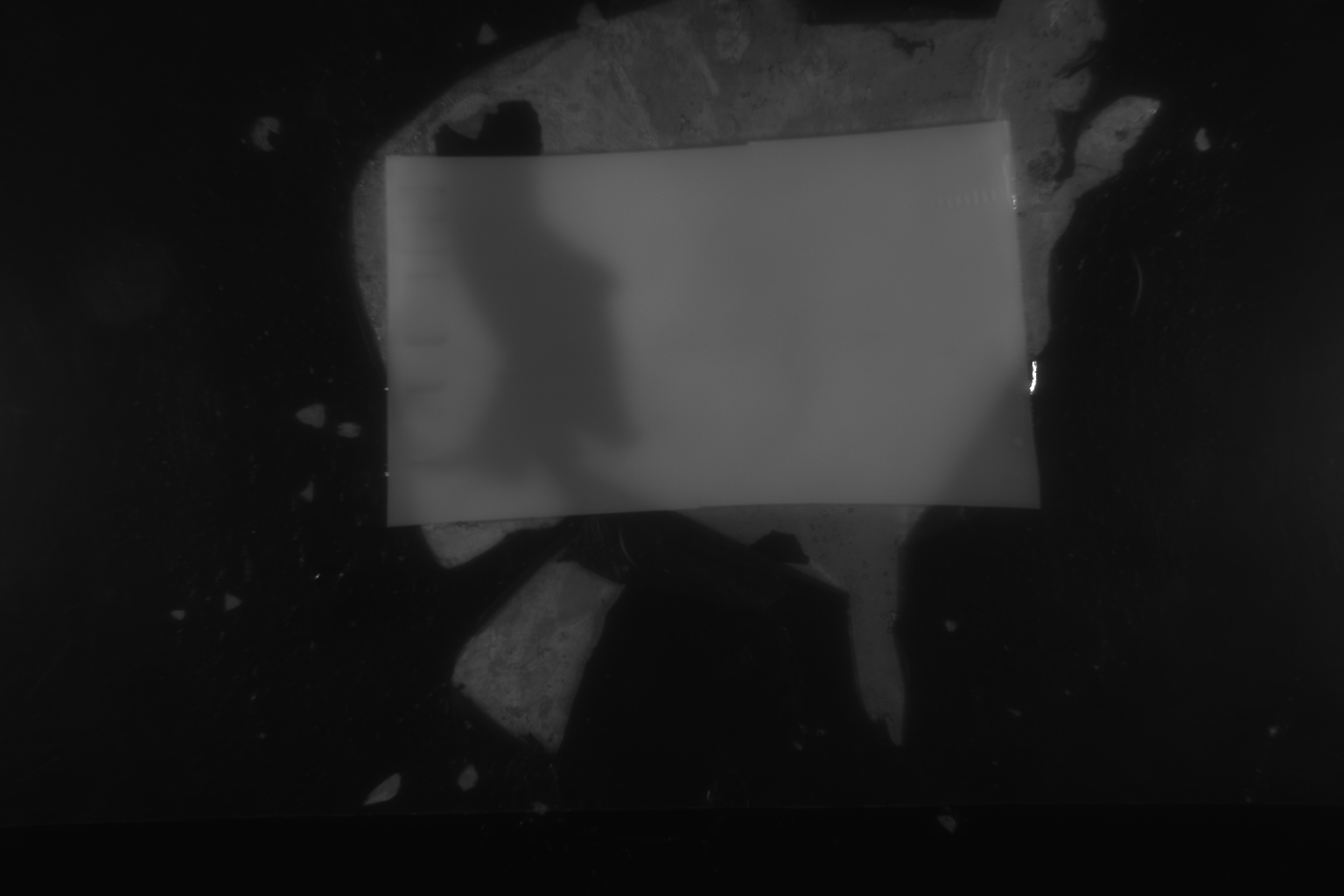

Supplement: Supplementary file 13 — Figure EV2 Source Data [file 44318_2025_572_MOESM13_ESM.zip › EV 2/EV 2T/B ACTIN 0.5 SEC (1).gel]

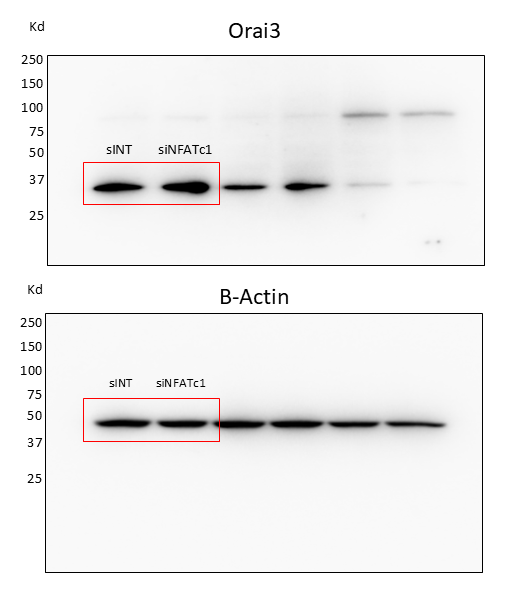

Supplement: Supplementary file 13 — Figure EV2 Source Data [file 44318_2025_572_MOESM13_ESM.zip › EV 2/EV 2T/EV 2T.png]

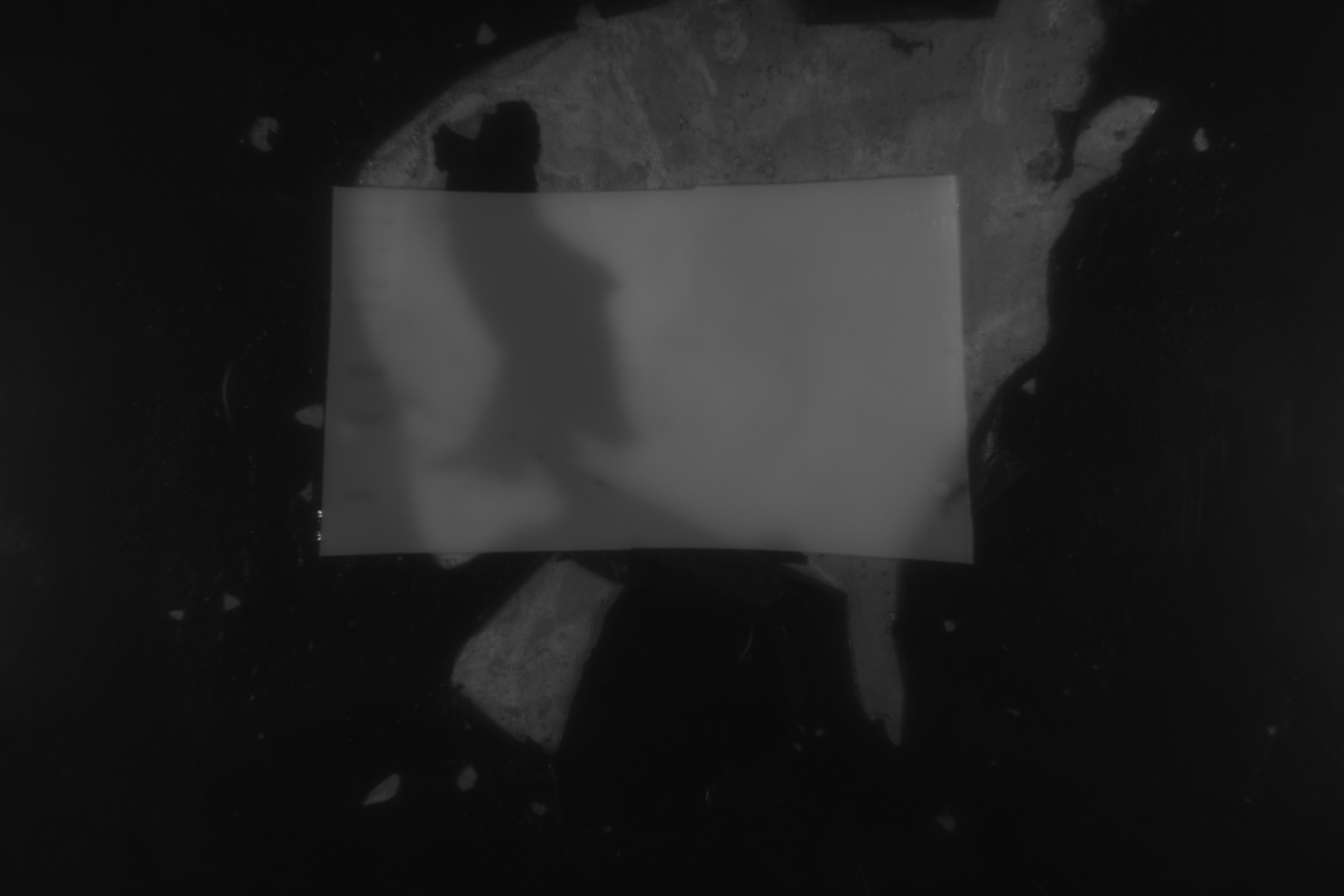

Supplement: Supplementary file 13 — Figure EV2 Source Data [file 44318_2025_572_MOESM13_ESM.zip › EV 2/EV 2T/ORAI 3 15 SEC (2).gel]

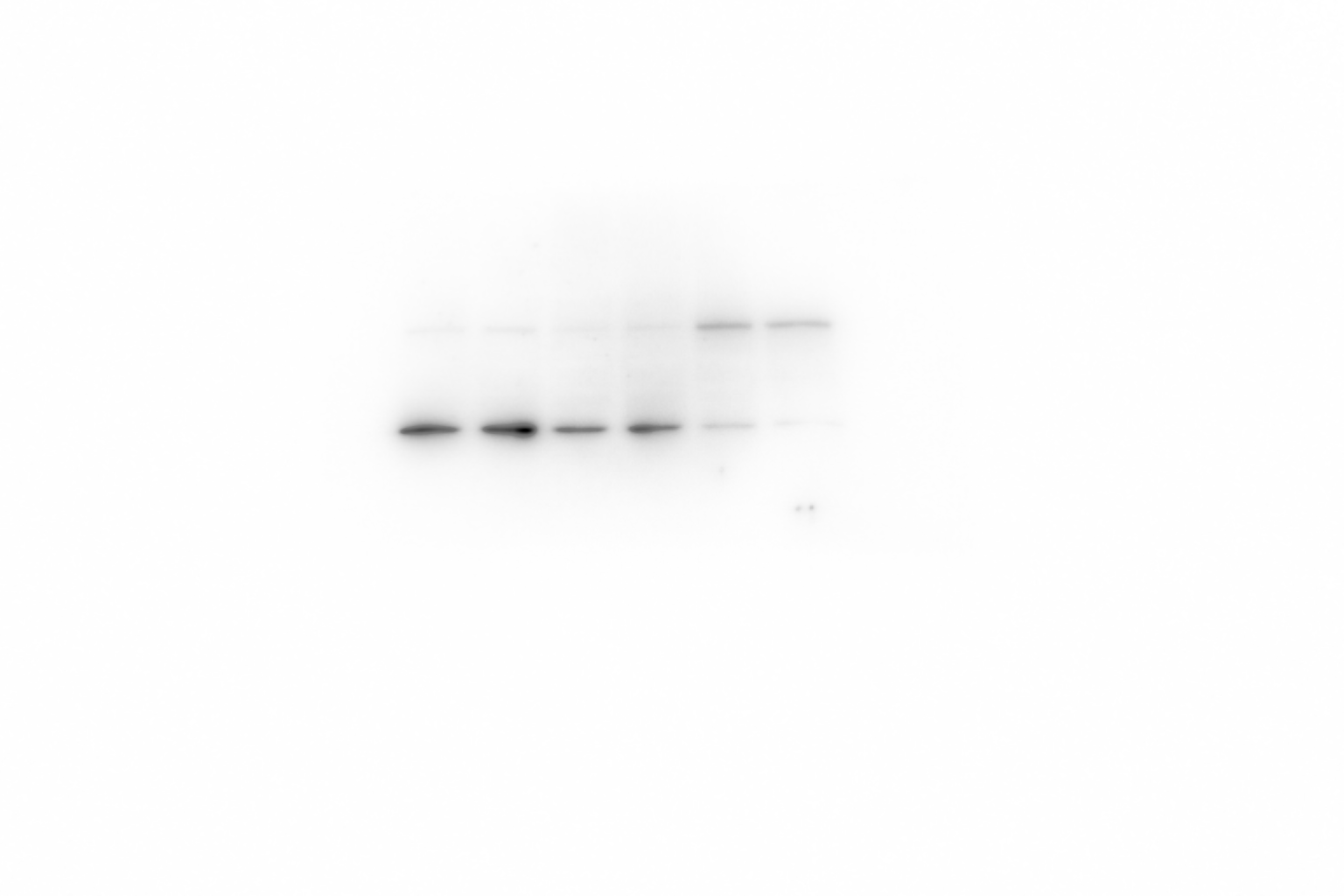

Supplement: Supplementary file 13 — Figure EV2 Source Data [file 44318_2025_572_MOESM13_ESM.zip › EV 2/EV 2T/ORAI 3 15 SEC (1).gel]

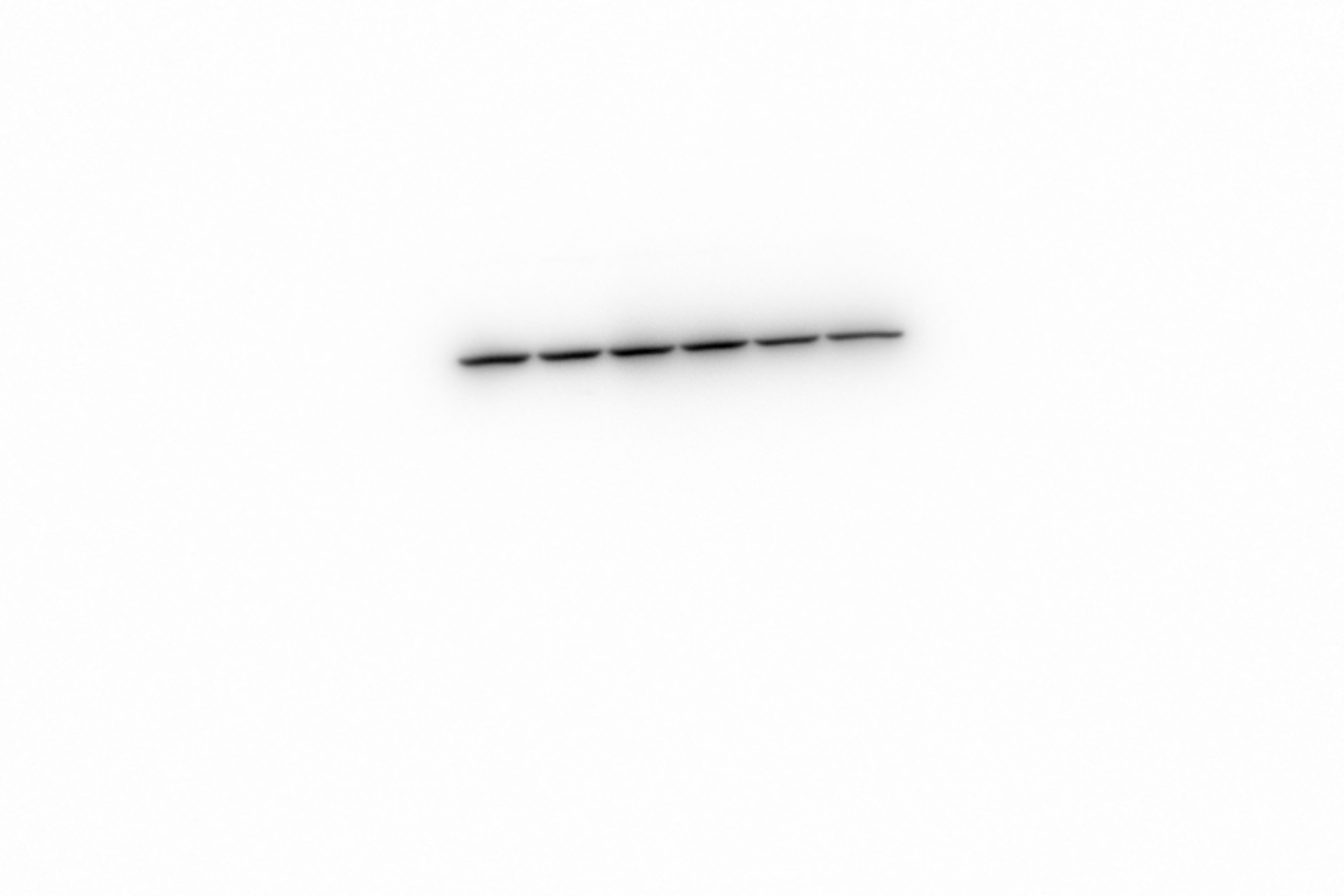

Supplement: Supplementary file 13 — Figure EV2 Source Data [file 44318_2025_572_MOESM13_ESM.zip › EV 2/EV 2T/B ACTIN 0.5 SEC (2).gel]

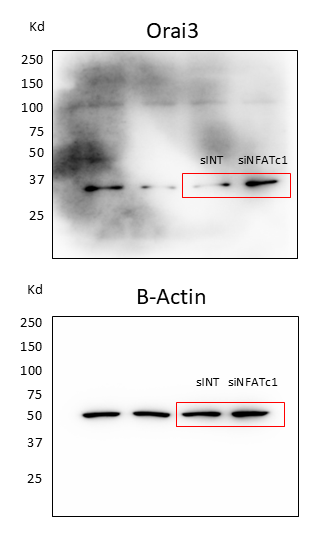

Supplement: Supplementary file 13 — Figure EV2 Source Data [file 44318_2025_572_MOESM13_ESM.zip › EV 2/EV 2O/EV 2O.png]

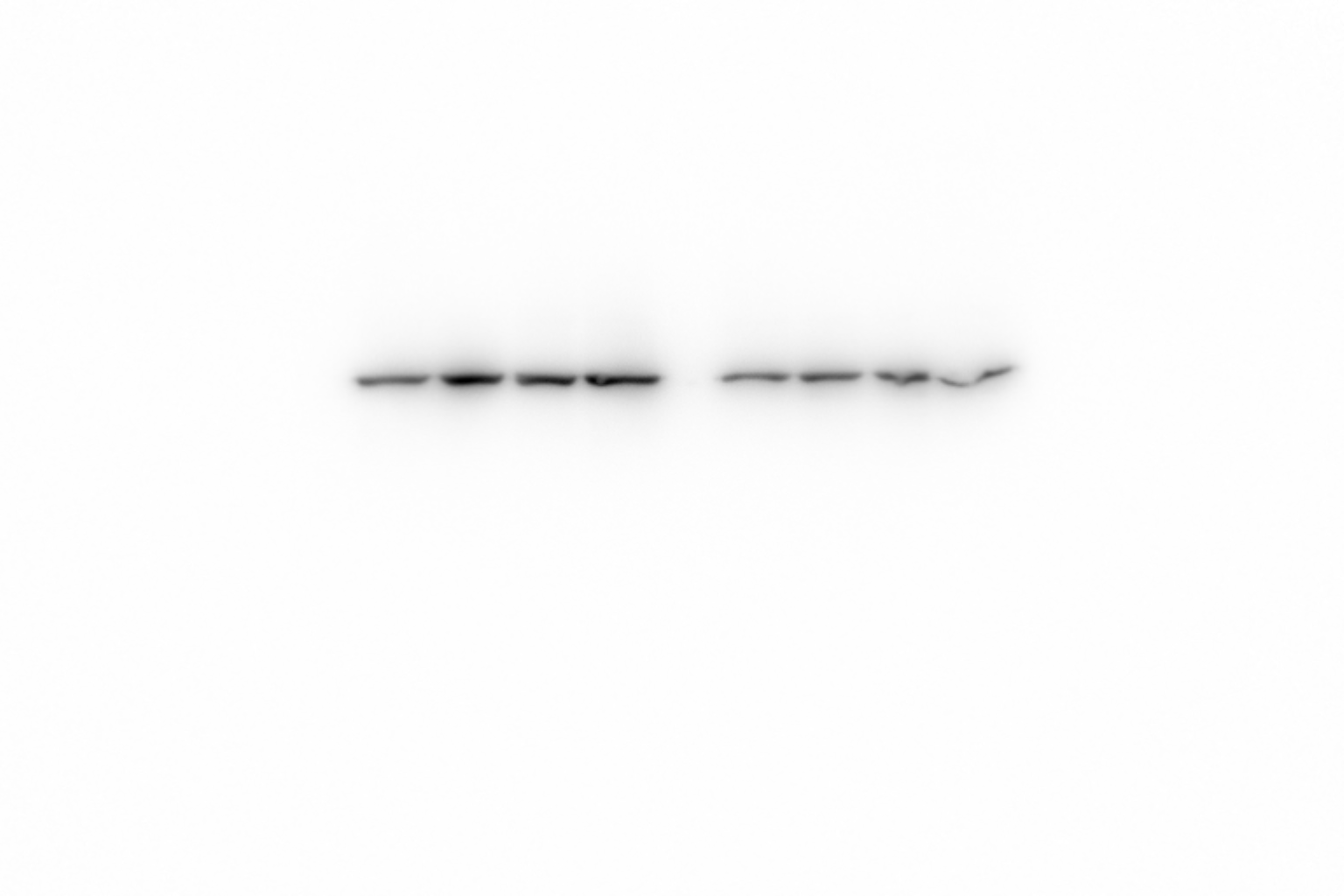

Supplement: Supplementary file 14 — Figure EV3 Source Data [file 44318_2025_572_MOESM14_ESM.zip › EV 3/EV 3N/B Actin 1 sec 2.gel]

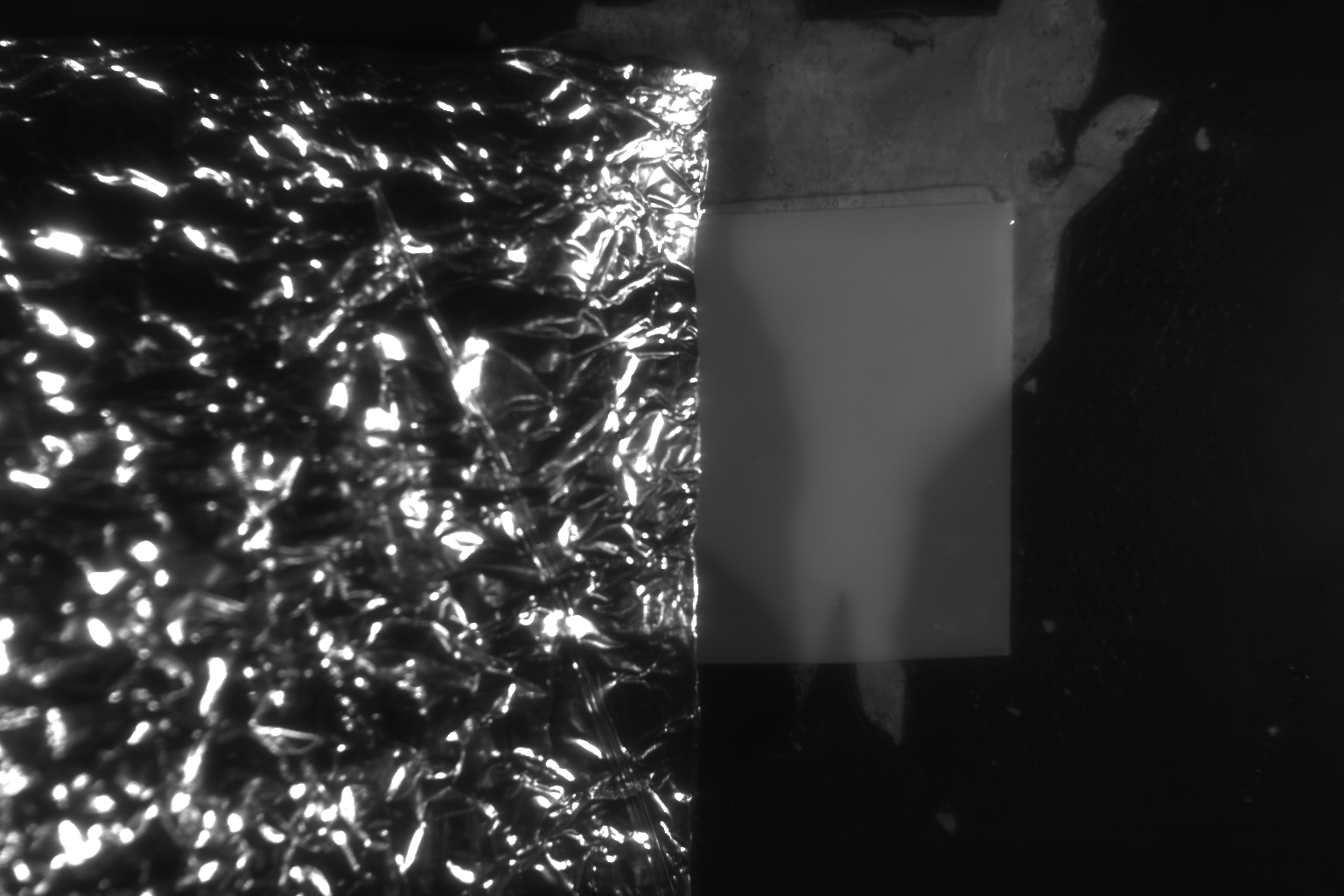

Supplement: Supplementary file 14 — Figure EV3 Source Data [file 44318_2025_572_MOESM14_ESM.zip › EV 3/EV 3N/V_ORAI3 60 SEC 3.gel]

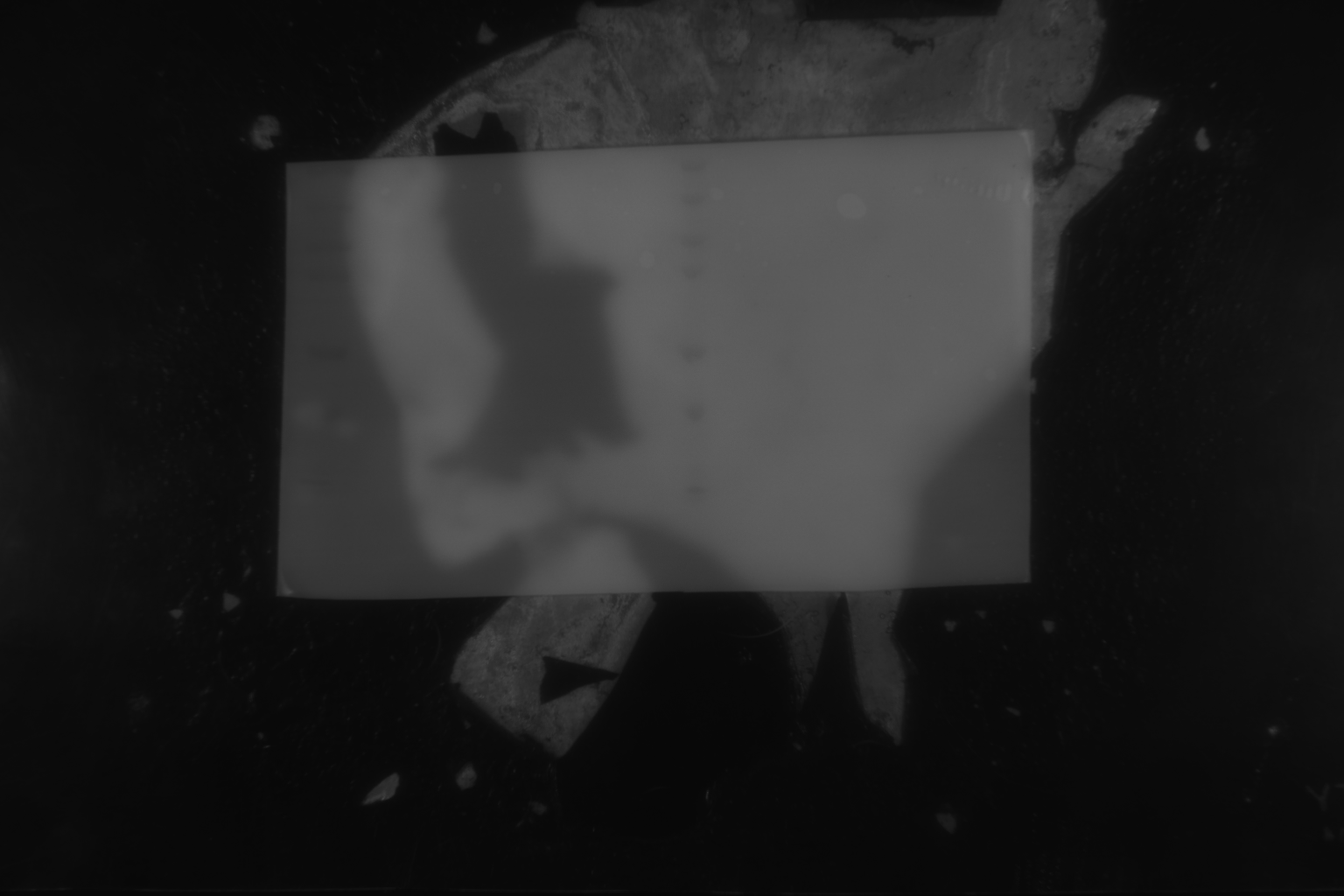

Supplement: Supplementary file 14 — Figure EV3 Source Data [file 44318_2025_572_MOESM14_ESM.zip › EV 3/EV 3N/V_B Actin 1 sec 2.gel]

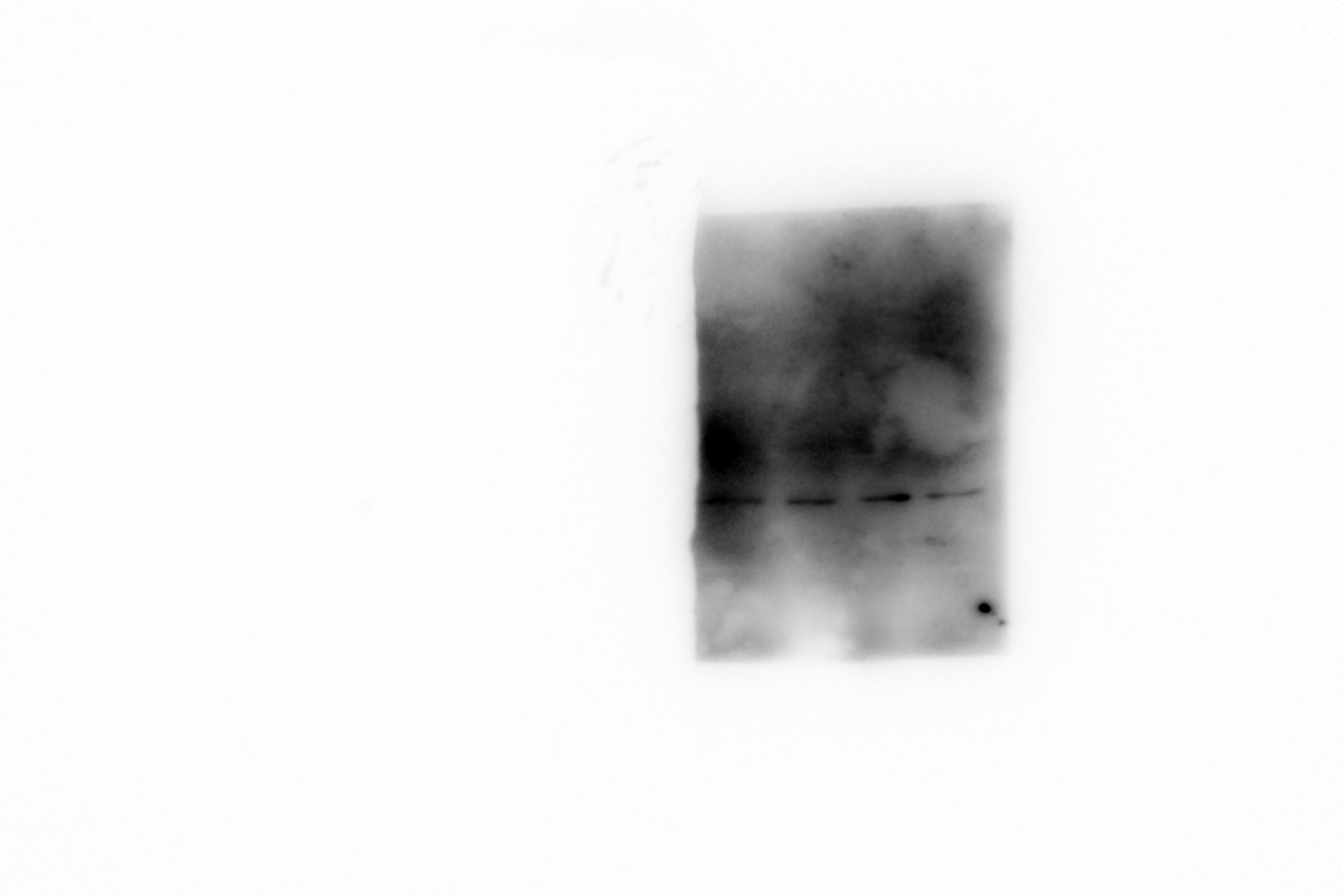

Supplement: Supplementary file 14 — Figure EV3 Source Data [file 44318_2025_572_MOESM14_ESM.zip › EV 3/EV 3N/ORAI3 60 SEC 3.gel]

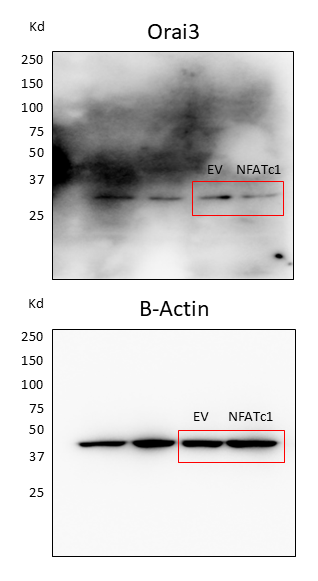

Supplement: Supplementary file 14 — Figure EV3 Source Data [file 44318_2025_572_MOESM14_ESM.zip › EV 3/EV 3N/EV 3N.png]

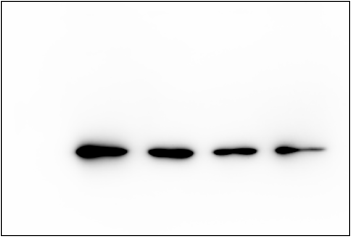

Supplement: Supplementary file 14 — Figure EV3 Source Data [file 44318_2025_572_MOESM14_ESM.zip › EV 3/EV 3H/Orai3.png]

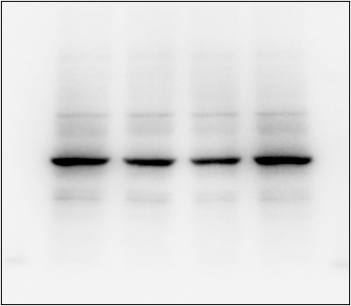

Supplement: Supplementary file 14 — Figure EV3 Source Data [file 44318_2025_572_MOESM14_ESM.zip › EV 3/EV 3H/B Actin.png]

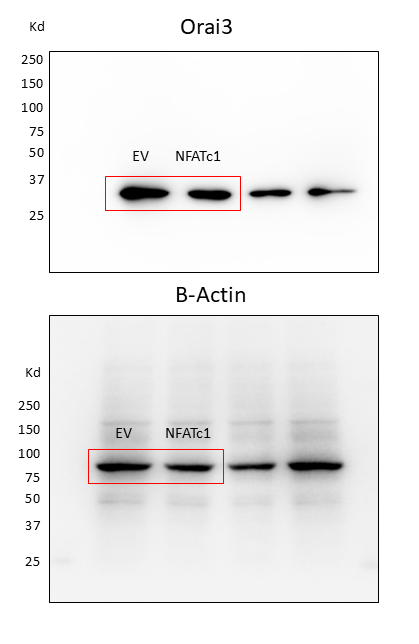

Supplement: Supplementary file 14 — Figure EV3 Source Data [file 44318_2025_572_MOESM14_ESM.zip › EV 3/EV 3H/EV 3H.png]

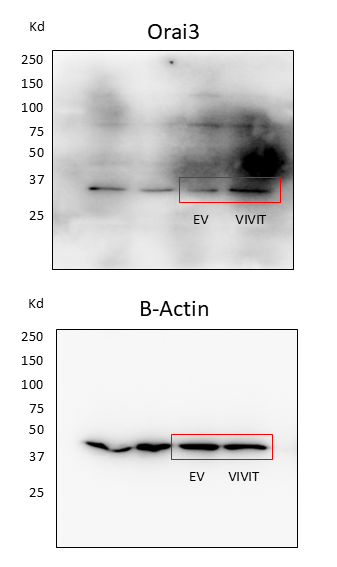

Supplement: Supplementary file 14 — Figure EV3 Source Data [file 44318_2025_572_MOESM14_ESM.zip › EV 3/EV 3Q/EV 3Q.png]

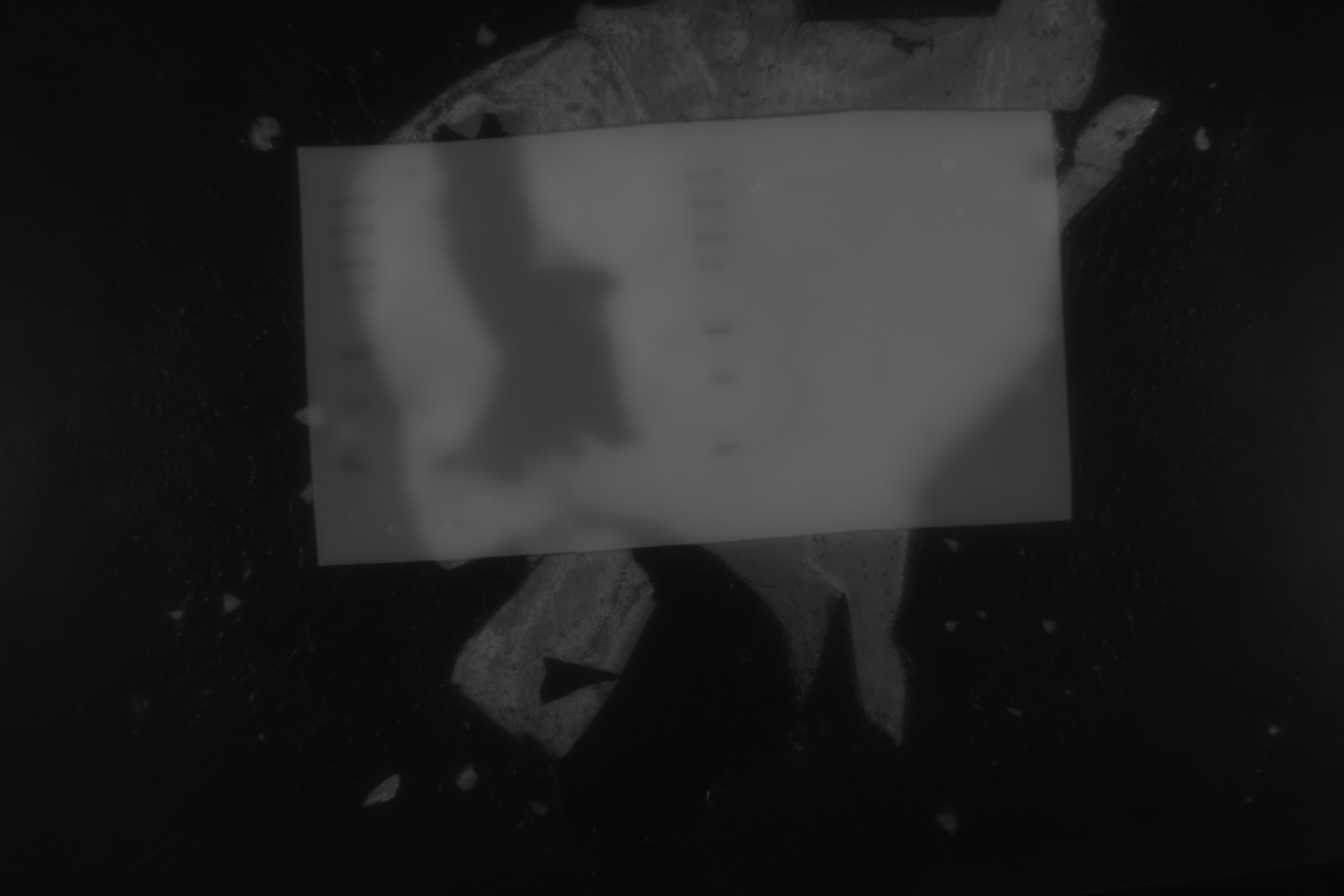

Supplement: Supplementary file 14 — Figure EV3 Source Data [file 44318_2025_572_MOESM14_ESM.zip › EV 3/EV 3K/V_ORAI3 60 SEC.gel]

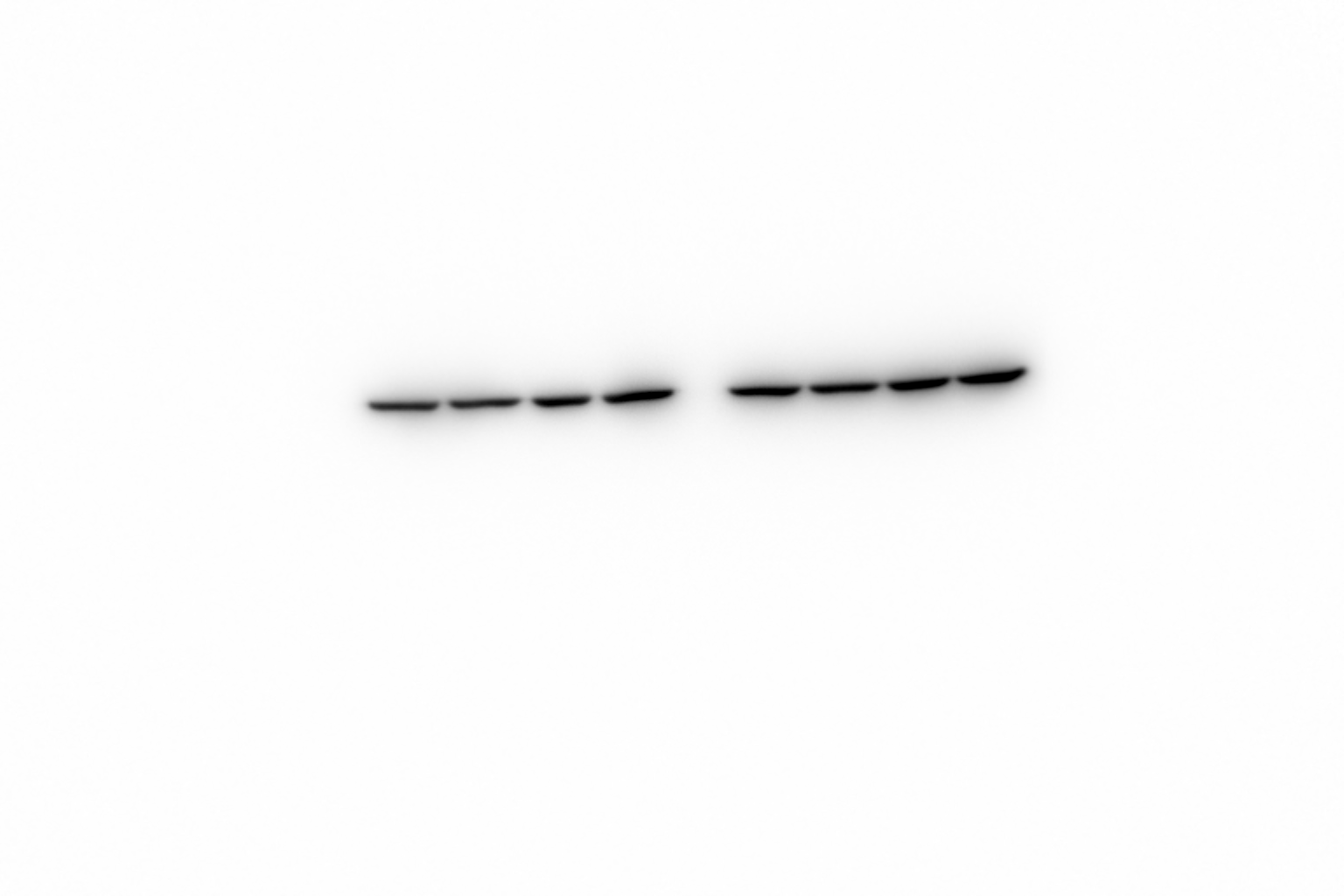

Supplement: Supplementary file 14 — Figure EV3 Source Data [file 44318_2025_572_MOESM14_ESM.zip › EV 3/EV 3K/B Actin 1 sec.gel]

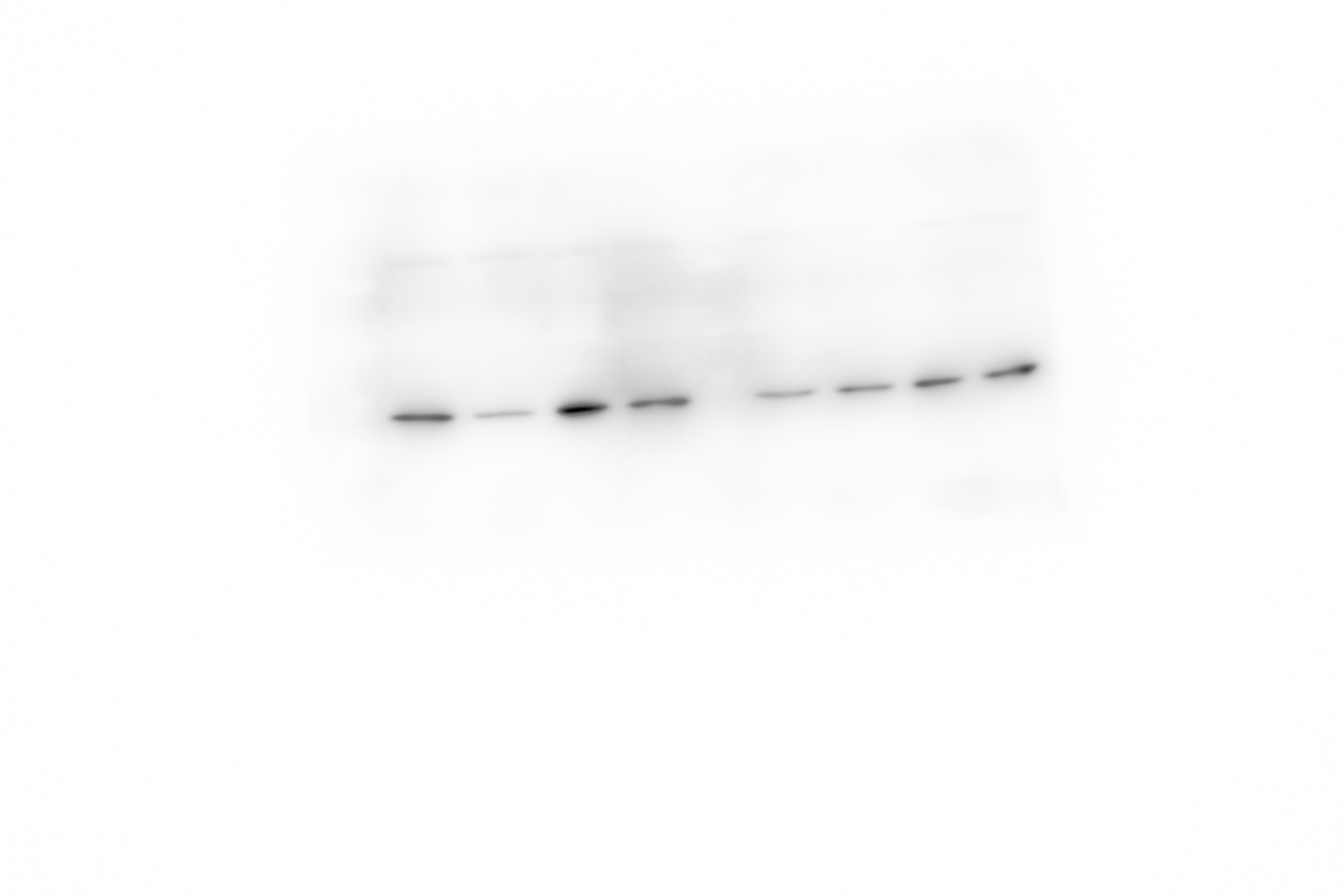

Supplement: Supplementary file 14 — Figure EV3 Source Data [file 44318_2025_572_MOESM14_ESM.zip › EV 3/EV 3K/ORAI3 60 SEC.gel]

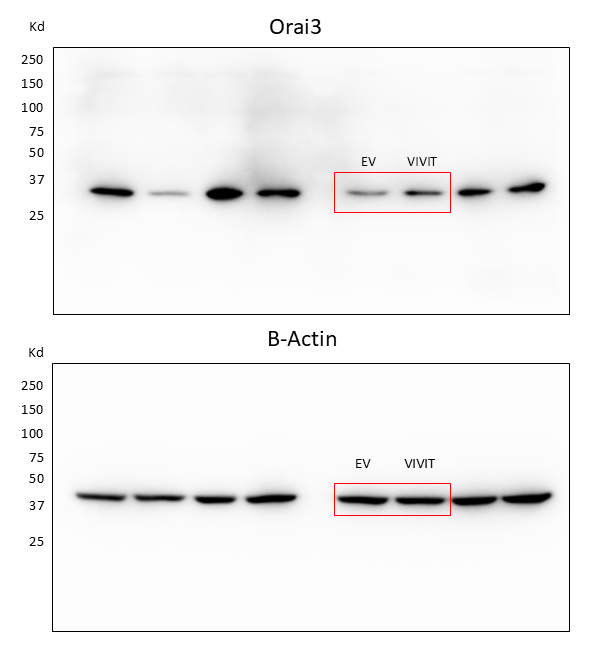

Supplement: Supplementary file 14 — Figure EV3 Source Data [file 44318_2025_572_MOESM14_ESM.zip › EV 3/EV 3K/EV 3K.png]

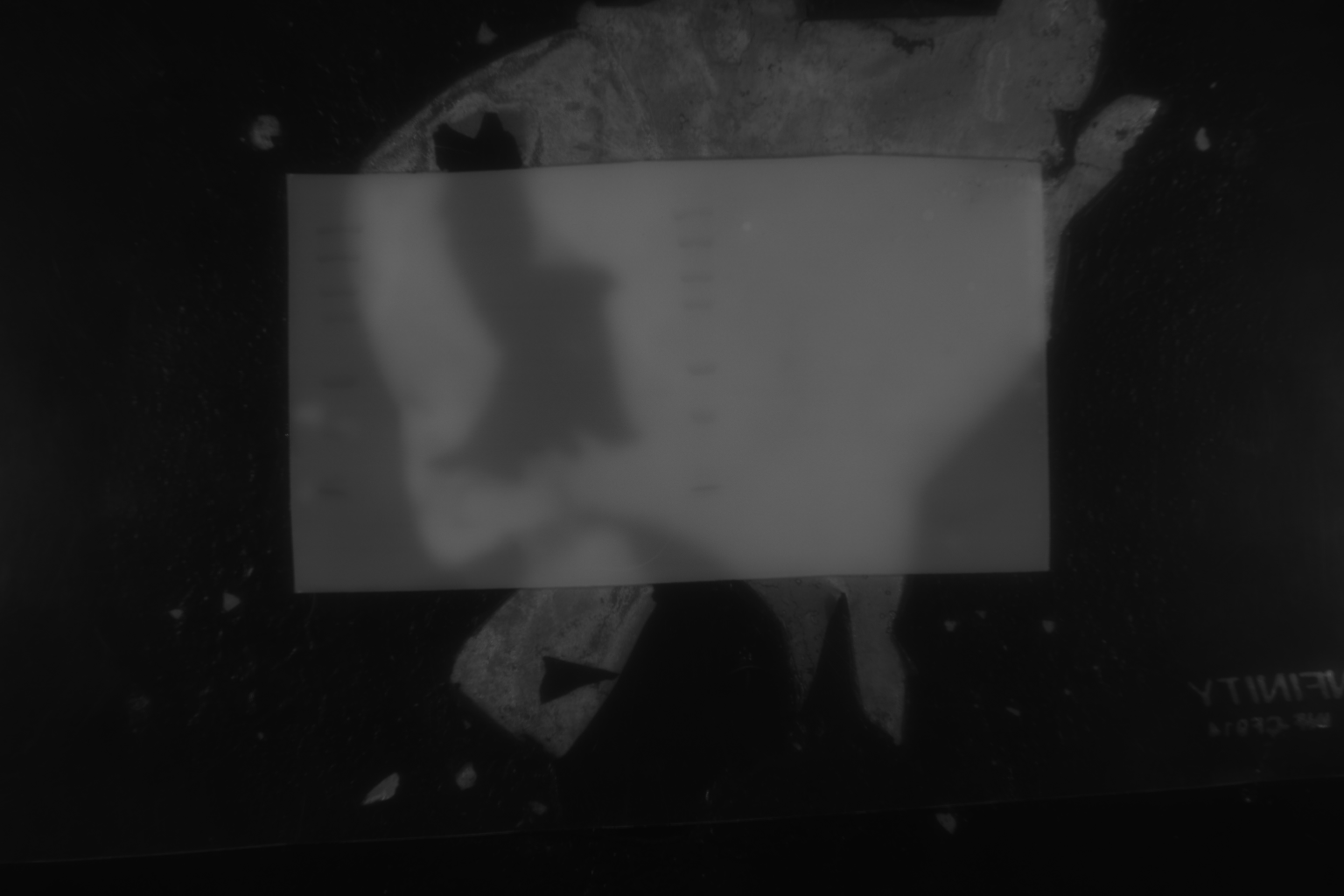

Supplement: Supplementary file 14 — Figure EV3 Source Data [file 44318_2025_572_MOESM14_ESM.zip › EV 3/EV 3K/V_B Actin 1 sec.gel]

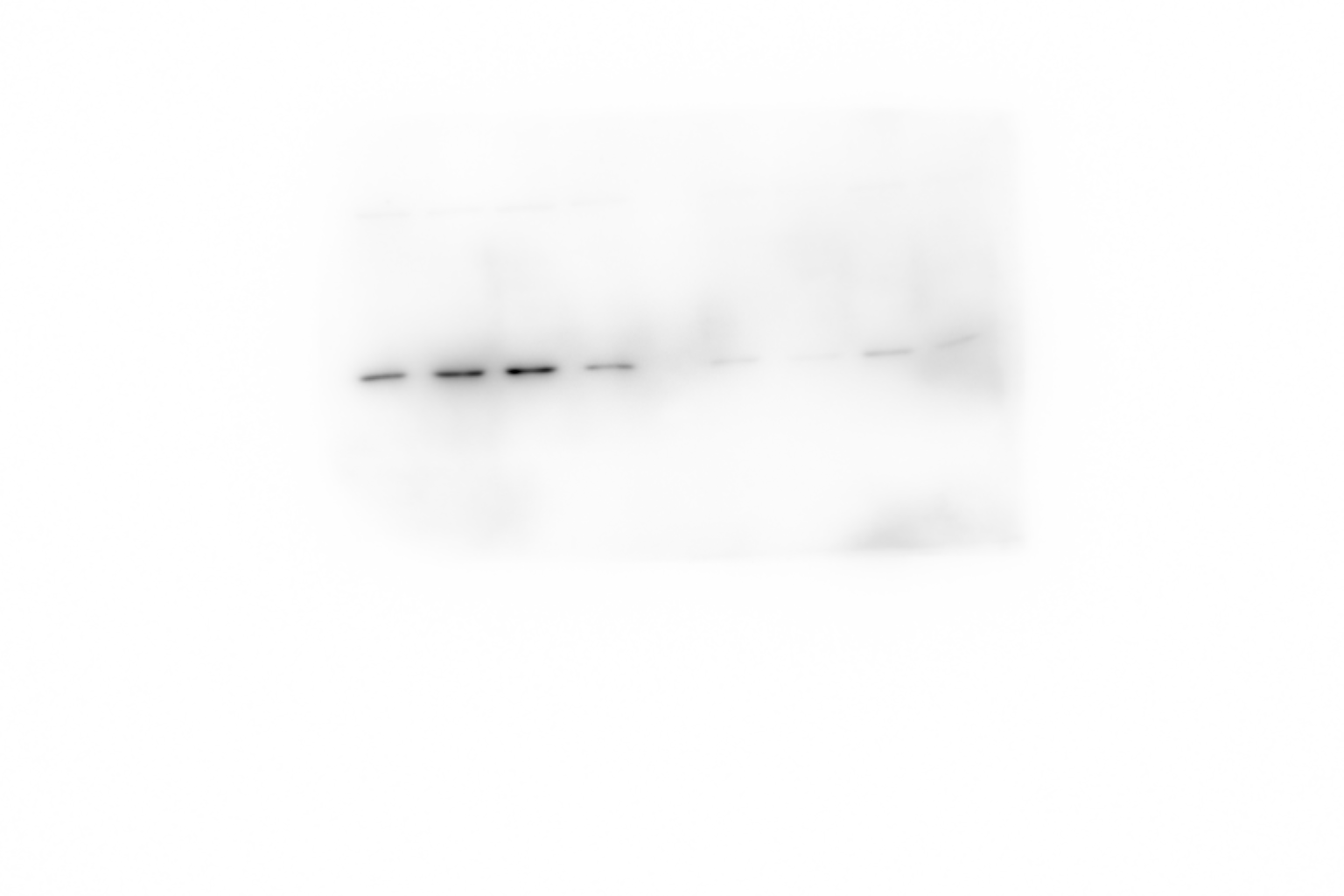

Supplement: Supplementary file 14 — Figure EV3 Source Data [file 44318_2025_572_MOESM14_ESM.zip › EV 3/EV 3E/ORAI3 30 SEC.gel]

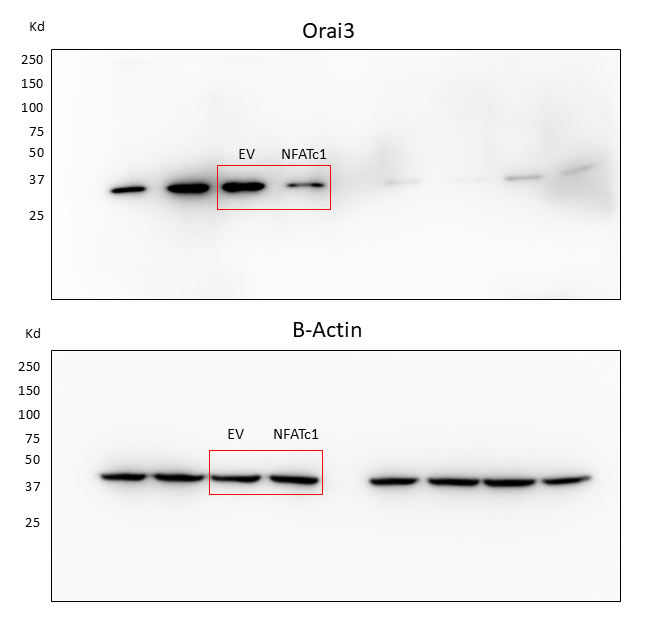

Supplement: Supplementary file 14 — Figure EV3 Source Data [file 44318_2025_572_MOESM14_ESM.zip › EV 3/EV 3E/EV 3E.png]

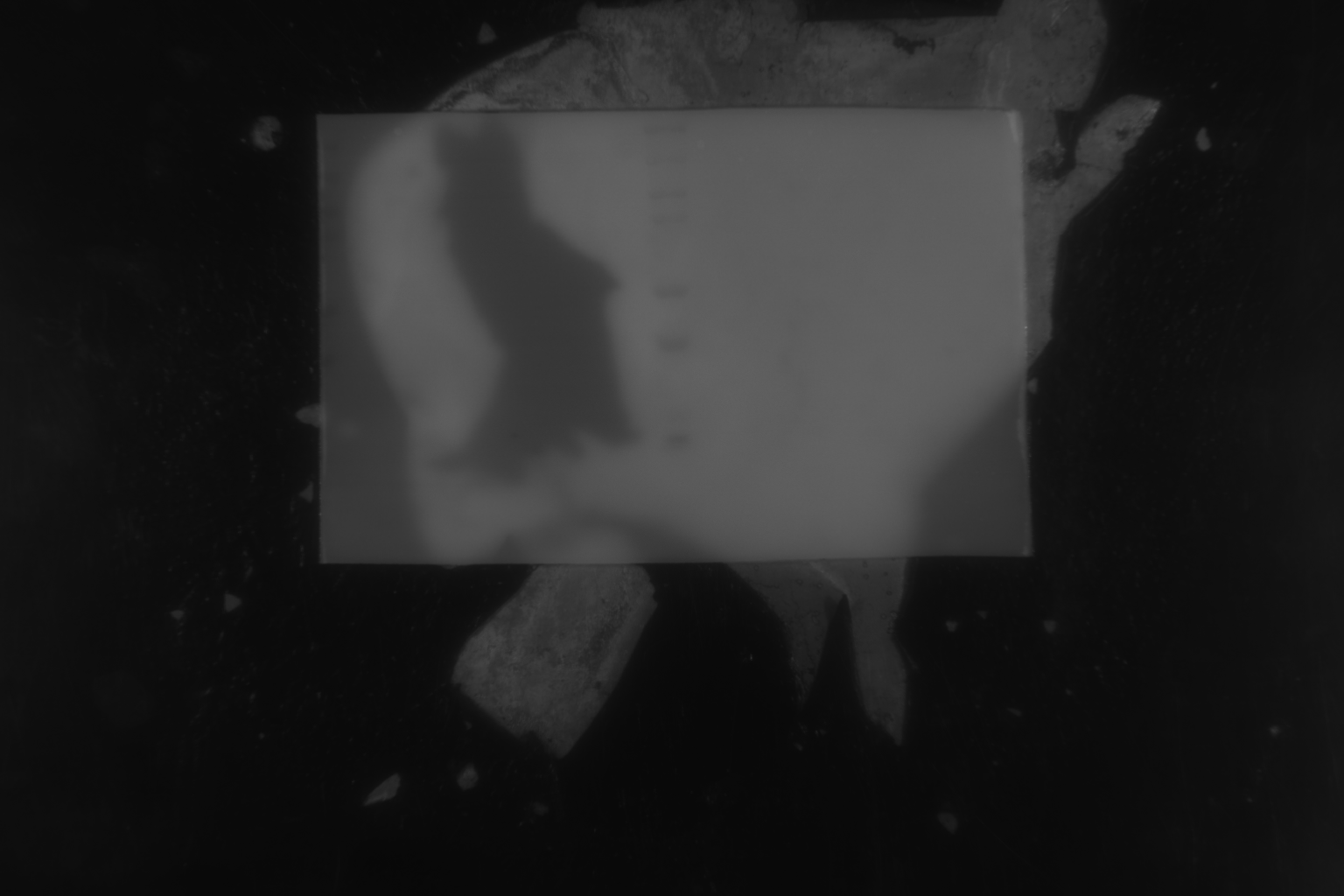

Supplement: Supplementary file 14 — Figure EV3 Source Data [file 44318_2025_572_MOESM14_ESM.zip › EV 3/EV 3E/V_ORAI3 30 SEC.gel]

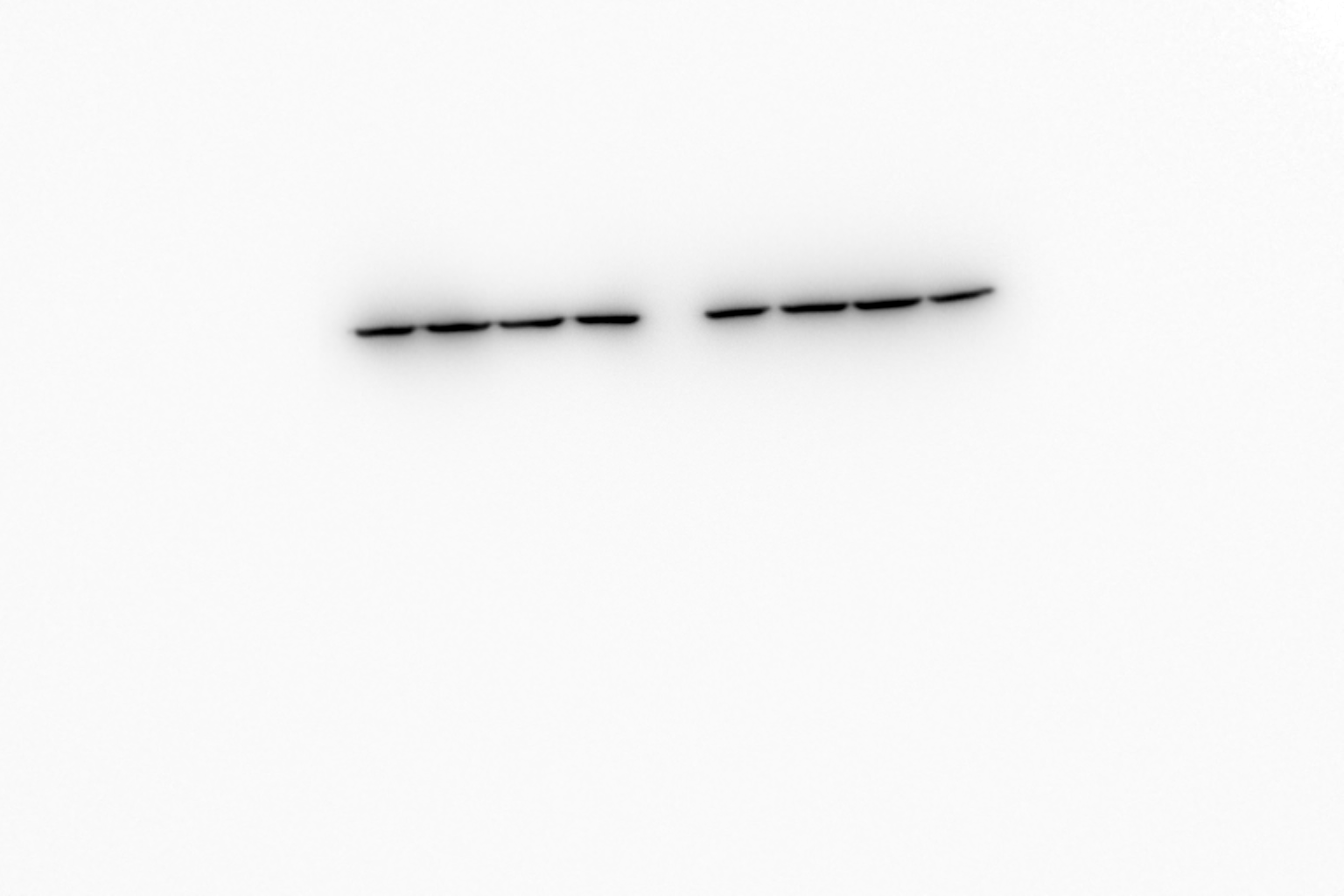

Supplement: Supplementary file 14 — Figure EV3 Source Data [file 44318_2025_572_MOESM14_ESM.zip › EV 3/EV 3E/B ACTIN 8 SEC.gel]

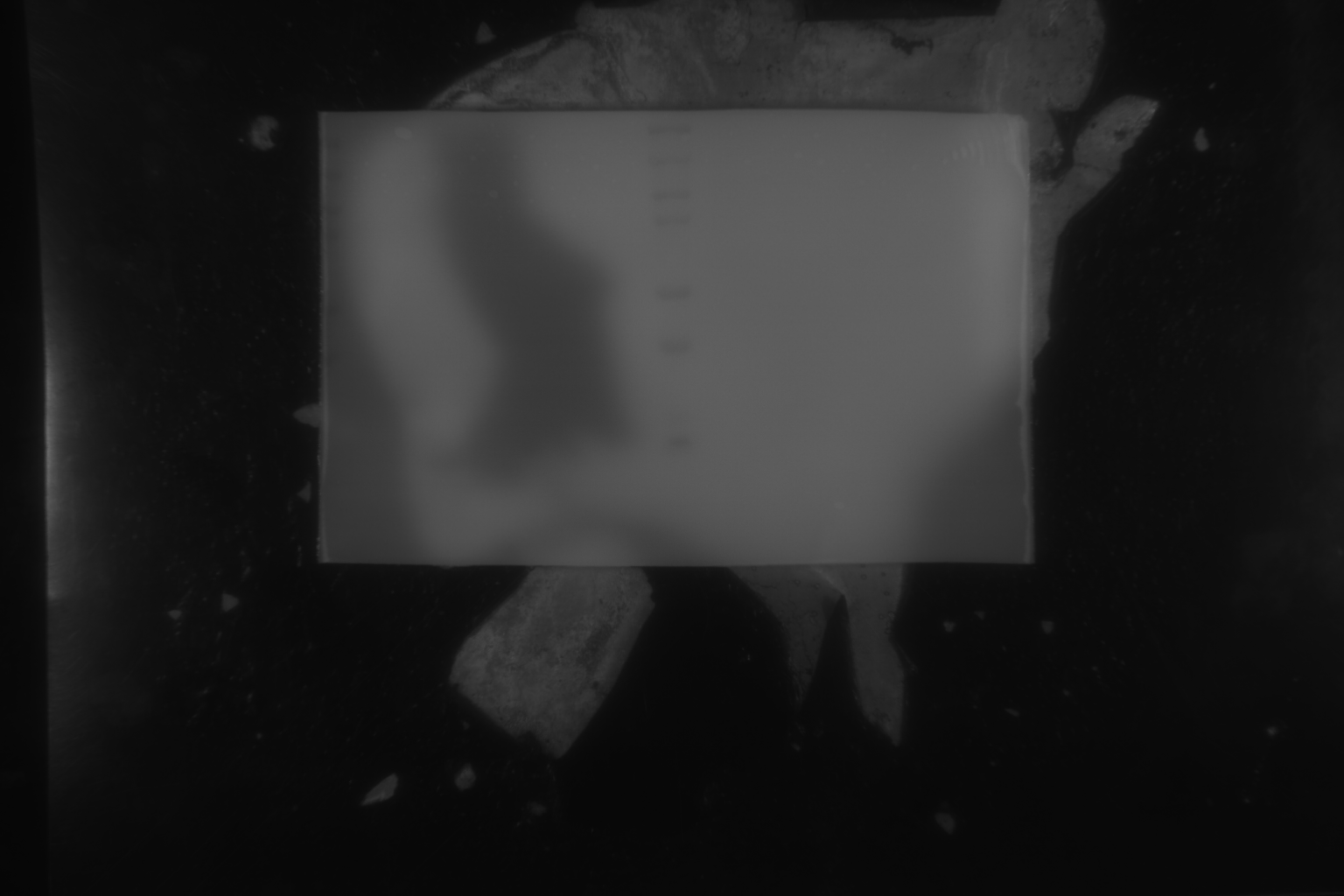

Supplement: Supplementary file 14 — Figure EV3 Source Data [file 44318_2025_572_MOESM14_ESM.zip › EV 3/EV 3E/V_B ACTIN 8 SEC.gel]

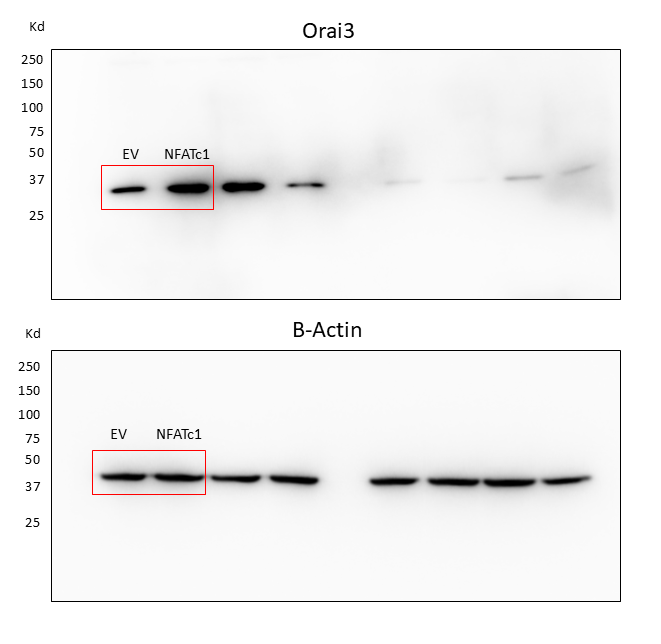

Supplement: Supplementary file 14 — Figure EV3 Source Data [file 44318_2025_572_MOESM14_ESM.zip › EV 3/EV 3B/EV 3B.png]

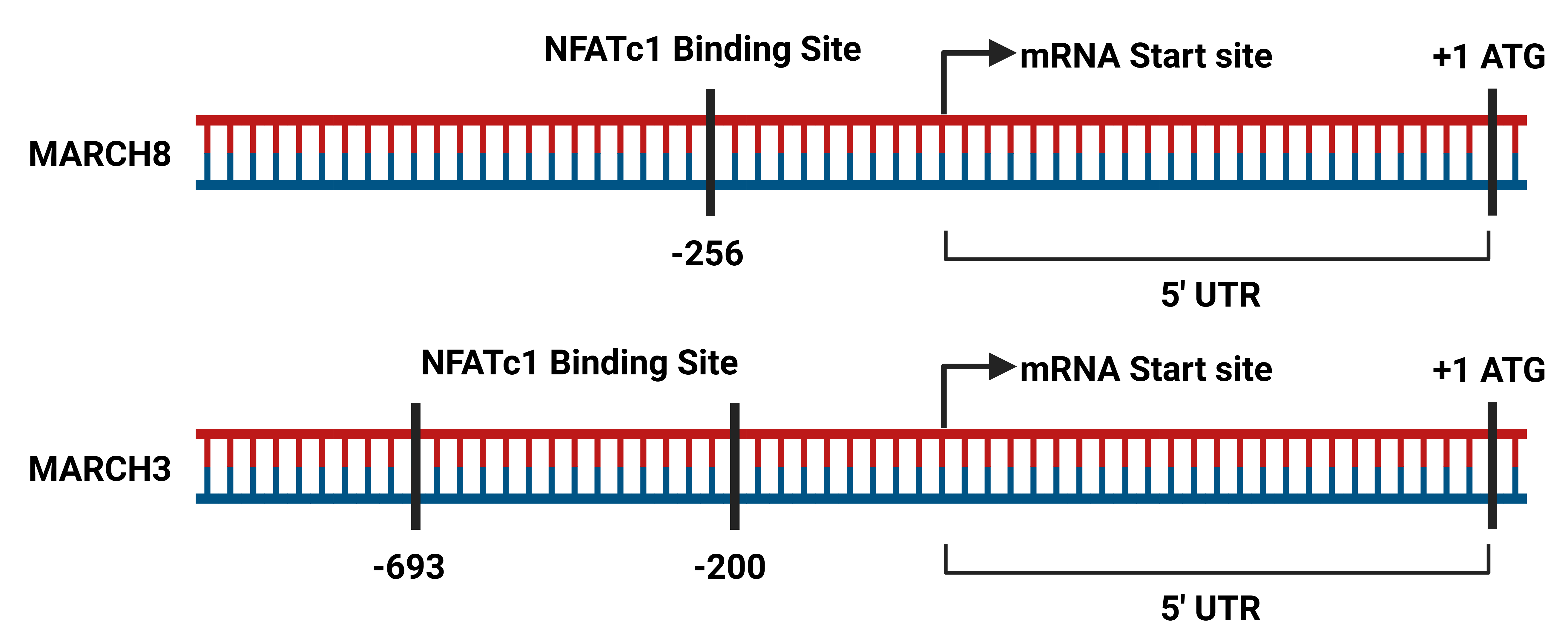

Supplement: Supplementary file 15 — Figure EV4 Source Data [file 44318_2025_572_MOESM15_ESM.zip › EV 4/EV 4A/MARCH 3 and 8 Promoter.png]

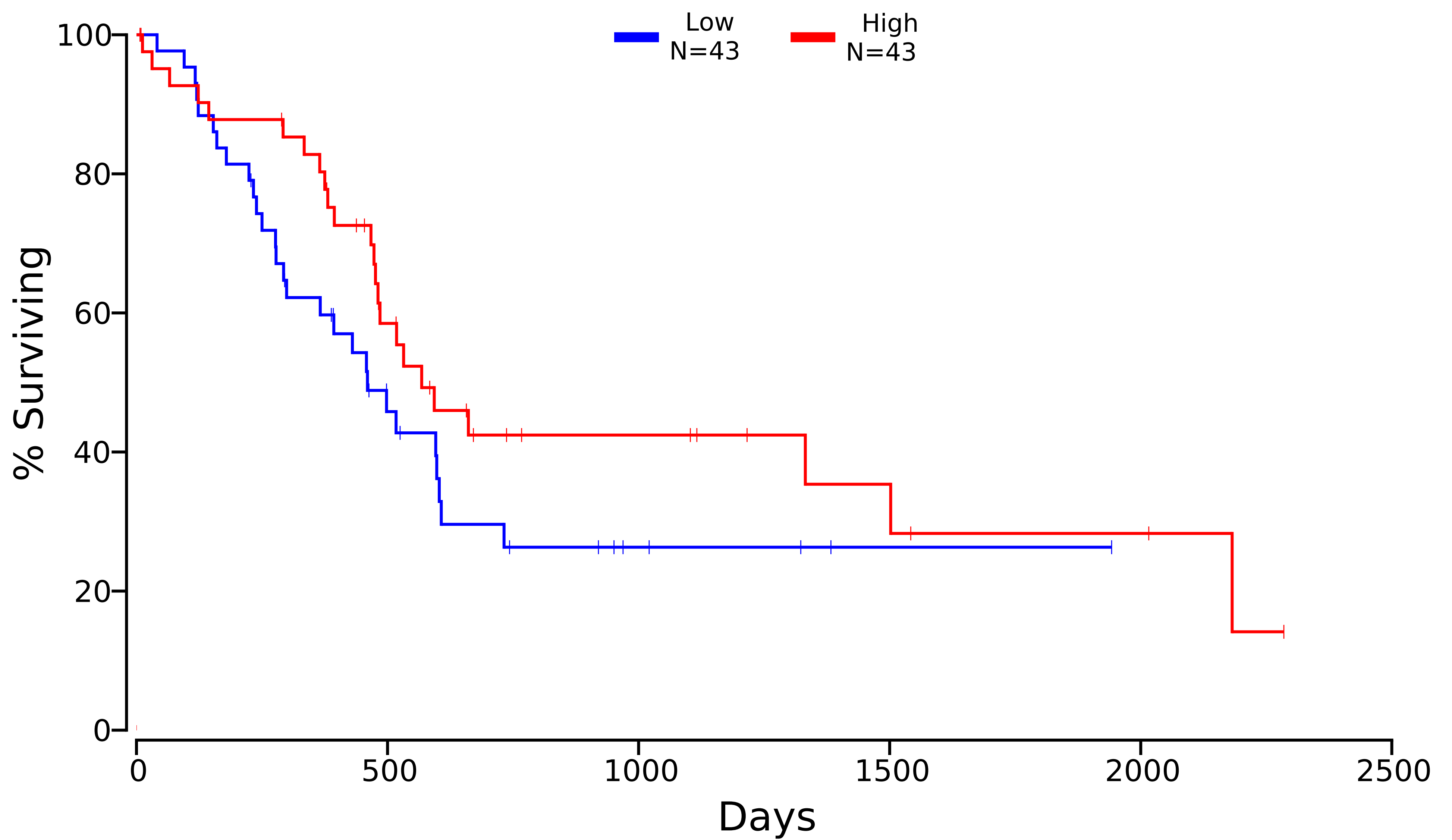

Supplement: Supplementary file 15 — Figure EV4 Source Data [file 44318_2025_572_MOESM15_ESM.zip › EV 4/EV 4D/MARCH3 Survival.pdf]

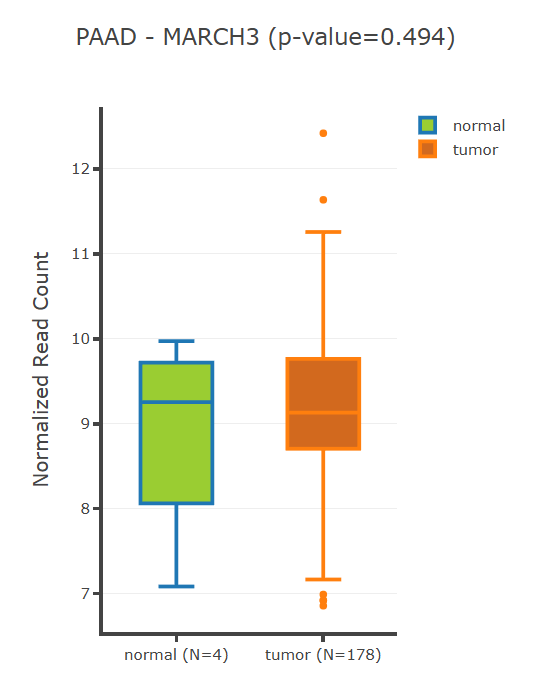

Supplement: Supplementary file 15 — Figure EV4 Source Data [file 44318_2025_572_MOESM15_ESM.zip › EV 4/EV 4B/March 3 Expression PAAD.png]
