# Supplementary material for: NFATc1 drives Orai3 transcription and proteolysis by harnessing epigenome differences in the MARCH8 promoter
Source: EMBO J. 2025 Sep 29;44(21):6137–67. doi: 10.1038/s44318-025-00572-4 (PMC12583688; doi:10.1038/s44318-025-00572-4)
Supplement: Supplementary file 17 — Expanded View Figures [file 44318_2025_572_MOESM17_ESM.pdf]

Expanded View Figures

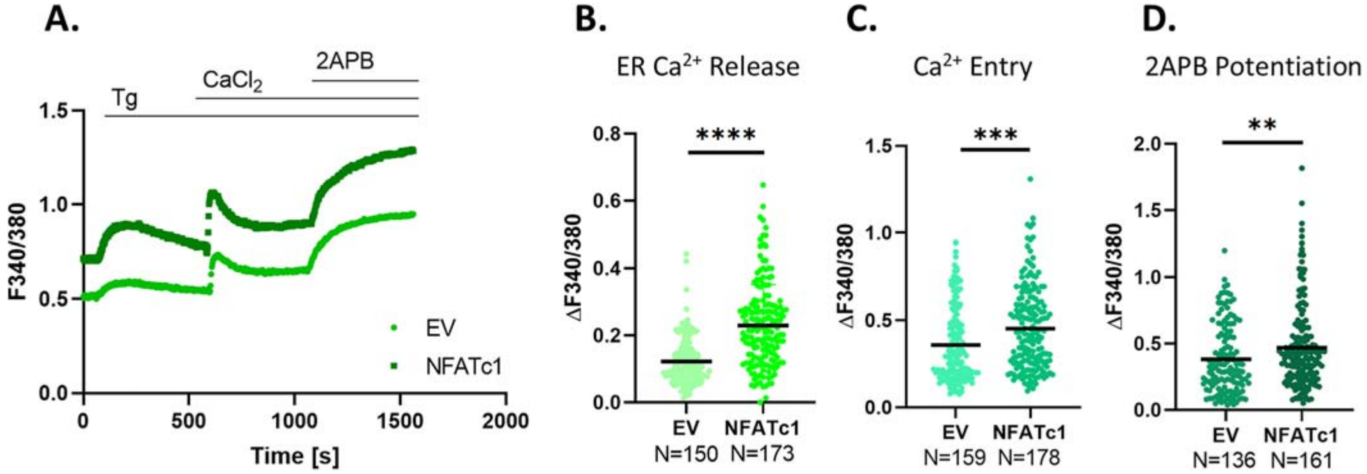

Figure EV1. Calcium imaging in MiaPaCa-2 using standard thapsigargin activated SOCE protocol.

(**A**) Representative Ca^{2+} imaging traces of empty vector control and NFATc1 overexpression in MiaPaCa-2. (**B**) Quantitation of ER Ca^{2+}release upon NFATc1 overexpression in MiaPaCa-2 compared to empty vector control where "N" denotes the number of ROIs. ****$P< 0.0001$. (**C**) Change in Ca^{2+}entry upon overexpression of NFATc1 compared to empty vector control in MiaPaCa-2 where "N" denotes the number of ROIs. ***$P = 0.0002$. (**D**) 2-APB potentiation of Orai3 in NFATc1 overexpressed and empty vector control MiaPaCa-2 where "N" denotes the number of ROIs. **$P = 0.0097$. Data presented are mean ± SEM. For statistical analysis, unpaired Student's *t* test was performed for(**B–D**) using GraphPad Prism software. Here, **$P < 0.01$; ***$P < 0.001$ and ****$P < 0.0001$. Source data are available online for this figure.

 © The Author(s)

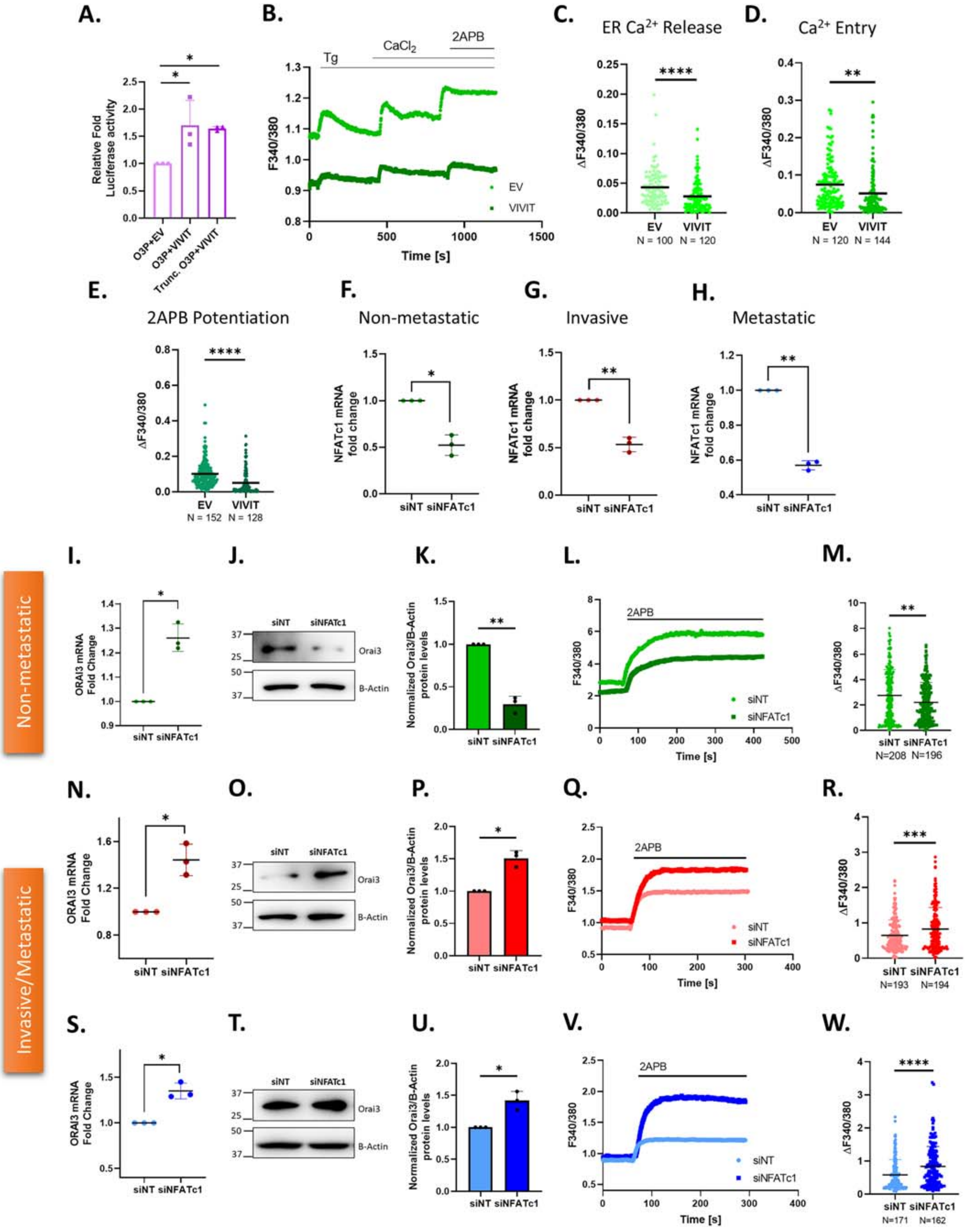
A.
Relative Fold Luciferase activity
O3P+EV
O3P+VIVIT
Trunc. O3P+VIVIT
B.
Tg
CaCl2
2APB
F340/380
Time [s]
EV
VIVIT
C.
ER Ca2+ Release
ΔF340/380
EV VIVIT
N = 100 N = 120
D.
Ca2+ Entry
ΔF340/380
EV VIVIT
N = 120 N = 144
E.
2APB Potentiation
ΔF340/380
EV VIVIT
N = 152 N = 128
F.
Non-metastatic
NFATc1 mRNA fold change
siNT siNFATc1
G.
Invasive
NFATc1 mRNA fold change
siNT siNFATc1
H.
Metastatic
NFATc1 mRNA fold change
siNT siNFATc1
Non-metastatic
I.
ORAI3 mRNA Fold Change
siNT siNFATc1
J.
siNT siNFATc1
Orai3
B-Actin
K.
Normalized Orai3/B-Actin protein levels
siNT siNFATc1
L.
2APB
F340/380
Time [s]
siNT
siNFATc1
M.
ΔF340/380
siNT siNFATc1
N=208 N=196
Invasive/Metastatic
N.
ORAI3 mRNA Fold Change
siNT siNFATc1
O.
siNT siNFATc1
Orai3
B-Actin
P.
Normalized Orai3/B-Actin protein levels
siNT siNFATc1
Q.
2APB
F340/380
Time [s]
siNT
siNFATc1
R.
ΔF340/380
siNT siNFATc1
N=193 N=194
S.
ORAI3 mRNA Fold Change
siNT siNFATc1
T.
siNT siNFATc1
Orai3
B-Actin
U.
Normalized Orai3/B-Actin protein levels
siNT siNFATc1
V.
2APB
F340/380
Time [s]
siNT
siNFATc1
W.
ΔF340/380
siNT siNFATc1
N=171 N=162

Figure EV2. siRNA mediated NFAT knockdown validates dichotomous regulation of Orai3 in non-metastatic v/s metastatic PC cells.

(**A**) Normalized luciferase activity of wild-type Orai3 promoter and truncated Orai3 promoter in PANC-1 cells upon VIVIT transfection for 48 h ($N = 3$). *$P = 0.0307$; *$P = 0.0438$. (**B**) Representative Ca^{2+} imaging trace of cells transfected with control vector pEGFP-N1 plasmid and VIVIT transfection in MiaPaCa-2. (**C**) Quantitation of ER Ca^{2+}release after VIVIT transfection in MiaPaCa-2 compared to empty vector control where "N" denotes the number of ROIs. ****$P < 0.0001$. (**D**) Change in Ca^{2+}entry upon VIVIT Transfection in MiaPaCa-2 where "N" denotes the number of ROIs. **$P = 0.0018$. (**E**) 2-APB potentiation of Orai3 in VIVIT-transfected and empty vector control MiaPaCa-2 where "N" denotes the number of ROIs. ****$P < 0.0001$. (**F**) qRT-PCR analysis showing NFATc1 knockdown validation in MiaPaCa-2 ($N = 3$). *$P = 0.0173$. (**G**) qRT-PCR analysis showing NFATc1 knockdown validation in PANC-1 ($N = 3$). **$P = 0.0088$. (**H**) qRT-PCR analysis showing NFATc1 knockdown validation in CFPAC-1 ($N = 3$). **$P = 0.0013$. (**I**) qRT-PCR analysis showing increase in Orai3 mRNA levels upon NFATc1 knockdown in MiaPaCa-2 compared to control ($N = 3$). *$P = 0.0148$. (**J**) Representative western blots showing decrease in Orai3 protein levels upon NFATc1 knockdown in MiaPaCa-2 cells compared to control. (**K**) Densitometric quantitation of Orai3 protein levels in NFATc1 knockdown MiaPaCa-2 cells compared to control ($N = 3$). **$P = 0.0060$. (**L**) Representative Ca^{2+} imaging trace of MiaPaCa-2 cells transfected with either control siNT or siNFATc1. (**M**). 2-APB potentiation of Orai3 in siNT and siNFATc1-transfected MiaPaCa-2 where "N" denotes the number of ROIs. **$P = 0.0024$. (**N**) qRT-PCR analysis showing increase in Orai3 mRNA expression upon NFATc1 knockdown in PANC-1 compared to control ($N = 3$). *$P = 0.0295$. (**O**) Representative western blots showing increase in Orai3 protein levels due to NFATc1 knockdown in PANC-1 compared to control. (**P**) Western blot densitometry of Orai3 protein levels after NFATc1 knockdown in PANC-1 cells compared to control ($N = 3$). *$P = 0.0185$. (**Q**) Representative Ca^{2+} imaging trace of PANC-1cells transfected with either control siNT or siNFATc1. (**R**) Potentiation of Orai3 by 2-APB in control siNT and siNFATc1-transfected PANC-1 where "N" denotes the number of ROIs. ***$P = 0.0009$. (**S**) qRT-PCR analysis showing increase in Orai3 mRNA expression upon NFATc1 knockdown in CFPAC-1 compared to control ($N = 3$). *$P = 0.0201$. (**T**) Representative western blots showing increase in Orai3 protein levels due to NFATc1 knockdown in CFPAC-1 compared to control. (**U**) Western blot densitometry of Orai3 protein in NFATc1 knockdown CFPAC-1 cells compared to control (N = 3). *$P = 0.0381$. (**V**) Representative Ca^{2+} imaging trace of CFPAC-1 cells transfected with either control siNT or siNFATc1. (**W**). Potentiation of Orai3 by 2-APB in siNT and siNFATc1-transfected CFPAC-1 where "N" denotes the number of ROIs. ****$P < 0.0001$. Data presented are mean ± SEM. For statistical analysis, unpaired Student's *t* test was performed for (**C**–**E**, **M**, **R**, **W**) while one-sample *t* test was performed for (**F**, **G**, **H**, **I**, **K**, **N**, **P**, **S**, **U**) using GraphPad Prism software. Here, *$P < 0.05$; **$P < 0.01$; ***$P < 0.001$ and ****$P < 0.0001$. Source data are available online for this figure.

 © The Author(s)

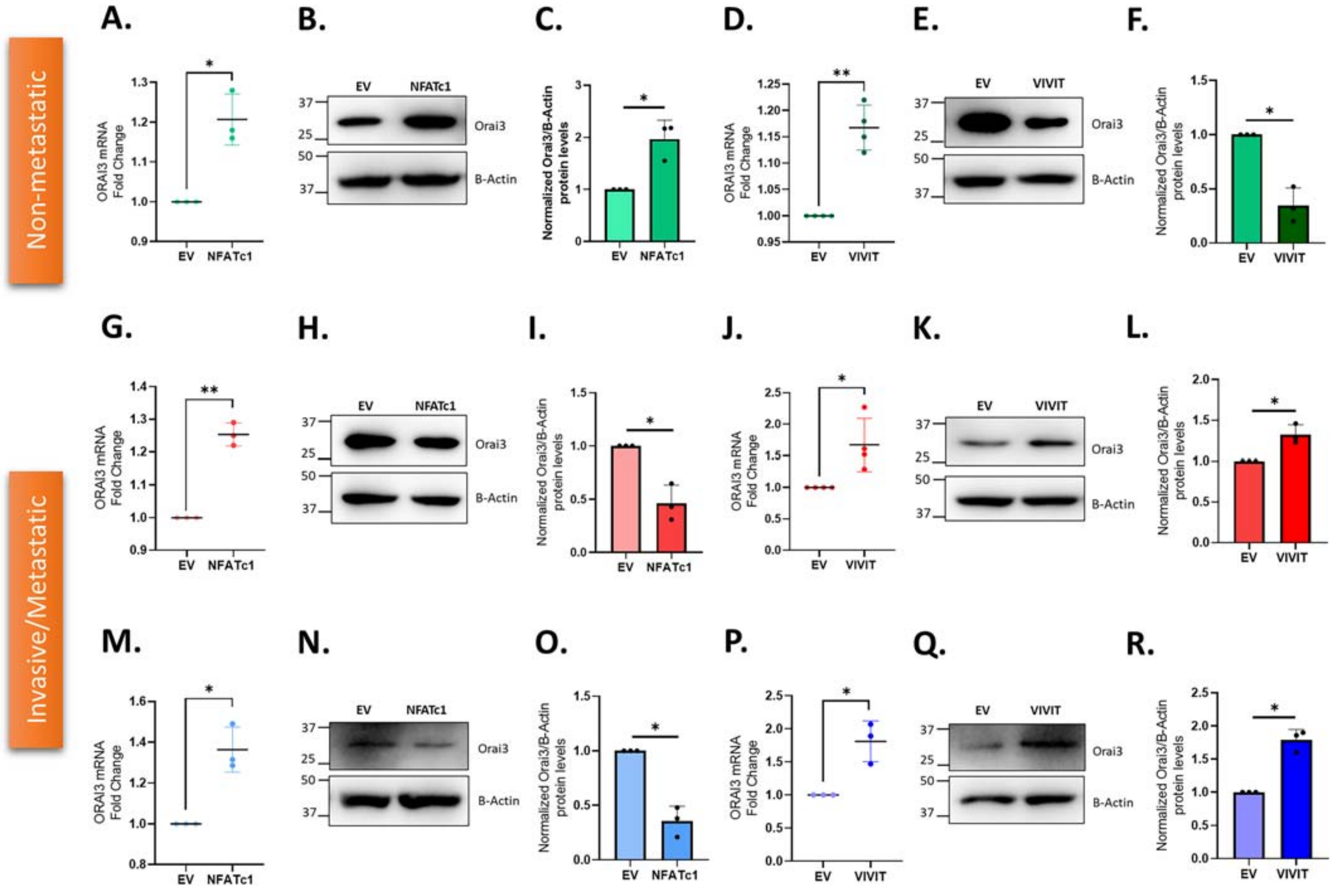

Figure EV3. NFATc1 differently regulates Orai3 expression in BxPC-3 (non-metastatic), ASPC-1 (invasive) and SW1990 (metastatic) PC cells.

(**A**) qRT-PCR analysis showing increase in Orai3 mRNA levels upon NFATc1 overexpression in BxPC-3 compared to control ($N = 3$). *$P = 0.0308$. (**B**) Representative western blots showing increase in Orai3 protein levels due to NFATc1 overexpression in BxPC-3 cells compared to control. (**C**) Densitometric quantitation of Orai3 protein levels in NFATc1 overexpressed BxPC-3 compared to control ($N = 3$). *$P = 0.0435$. (**D**) qRT-PCR analysis showing increase in Orai3 mRNA levels upon VIVIT transfection in BxPC-3 compared to control ($N = 3$). **$P = 0.0045$. (**E**) Representative western blots showing decrease in Orai3 protein levels due to VIVIT transfection in BxPC-3 cells compared to control. (**F**) Densitometric quantitation of Orai3 protein levels in VIVIT-transfected BxPC-3 compared to control ($N = 3$). *$P = 0.0198$. (**G**) qRT-PCR analysis showing increase in Orai3 mRNA levels upon NFATc1 overexpression in ASPC-1 compared to control ($N = 3$). **$P = 0.0063$. (**H**) Representative western blots showing decrease in Orai3 protein levels due to NFATc1 overexpression in ASPC-1 cells compared to control. (**I**) Densitometric quantitation of Orai3 protein levels in NFATc1 overexpressed ASPC-1 compared to control ($N = 3$). *$P = 0.0327$. (**J**) qRT-PCR analysis showing increase in Orai3 mRNA levels upon VIVIT transfection in ASPC-1 compared to control ($N = 3$). *$P = 0.0494$. (**K**) Representative western blots showing increase in Orai3 protein levels due to VIVIT transfection in ASPC-1 cells compared to control. (**L**) Densitometric quantitation of Orai3 protein levels in VIVIT-transfected ASPC-1 compared to control ($N = 3$). *$P = 0.0436$. (**M**) qRT-PCR analysis showing increase in Orai3 mRNA levels upon NFATc1 overexpression in SW1990 compared to control ($N = 3$). *$P = 0.0296$. (**N**) Representative western blots showing decrease in Orai3 protein levels due to NFATc1 overexpression in SW1990 cells compared to control. (**O**) Densitometric quantitation of Orai3 protein levels in NFATc1 overexpressed SW1990 compared to control ($N = 3$). *$P = 0.0147$. (**P**) qRT-PCR analysis showing increase in Orai3 mRNA levels upon VIVIT transfection in SW1990 compared to control ($N = 3$). *$P = 0.0449$. (**Q**) Representative western blots showing increase in Orai3 protein levels due to VIVIT transfection in SW1990 cells compared to control. (**R**) Densitometric quantitation of Orai3 protein levels in VIVIT-transfected SW1990 compared to control ($N = 3$). *$P = 0.0140$. Data presented are mean ± SEM. For statistical analysis, one-sample *t* test was performed for (**A**, **C**, **D**, **F**, **G**, **I**, **J**, **L**, **M**, **O**, **P**, **R**) using GraphPad Prism software. Here, *$P < 0.05$ and **$P < 0.01$. Source data are available online for this figure.

© The Author(s)

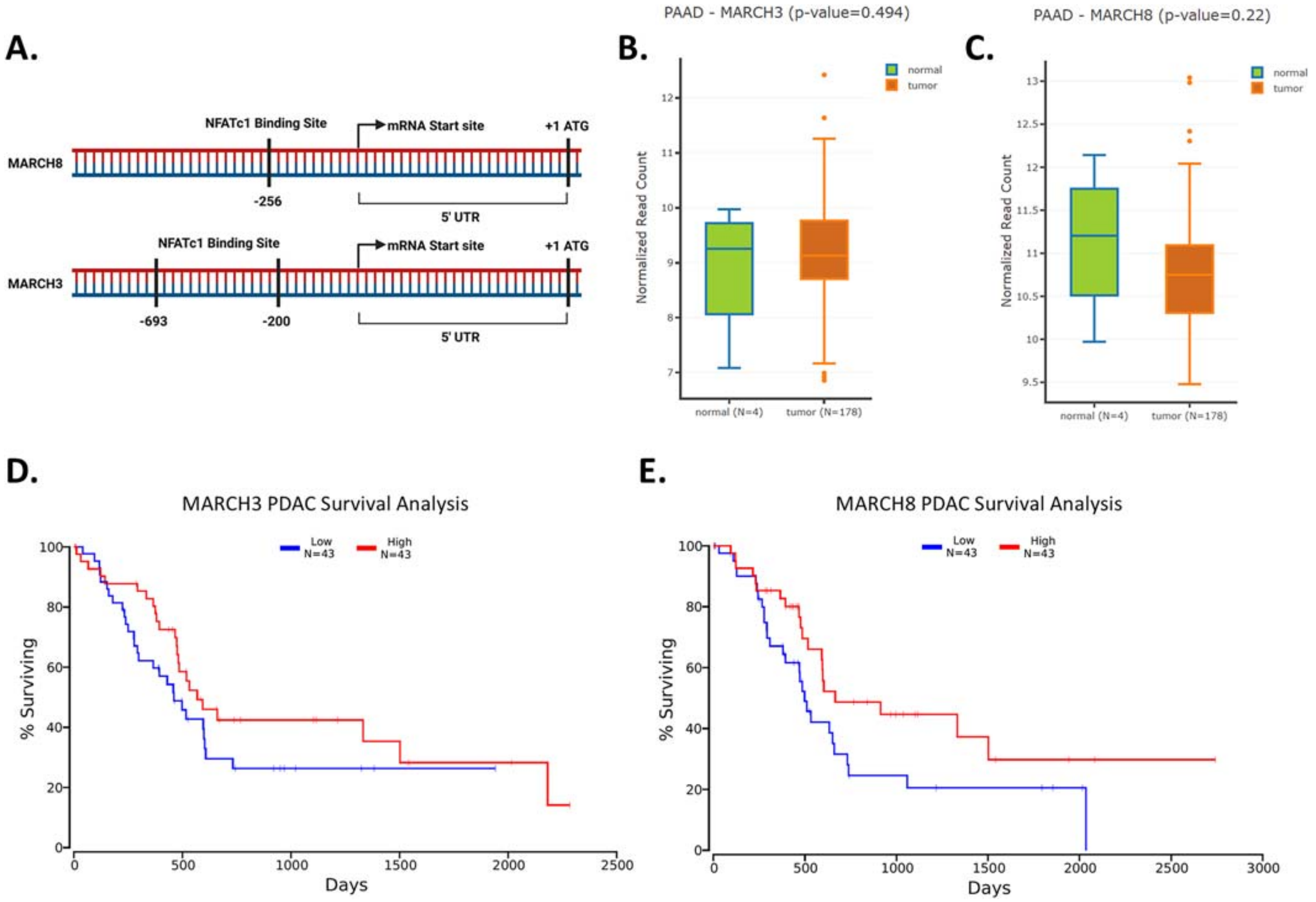

Figure EV4. Bioinformatic analysis of MARCH3 and MARCH8 E3 ubiquitin ligase.

(**A**) Identification of putative NFATc1 binding sites on the human MARCH3 and MARCH8 promoter using the EPD-Search Motif Tool at *P* value cut-off of 0.01. (**B**) MARCH3 expression levels in normal pancreatic tissues and pancreatic adenocarcinoma (PAAD) tissues analyzed by the DNMIVD database. (Normal: maxima-9.971; minima-7.082; median-9.252; Q1-8.061; Q3-9.718. Tumor: maxima-12.418; minima-6.856; median-9.129; Q1-8.704; Q3-9.762). (**C**) MARCH8 expression levels in normal pancreatic tissues and pancreatic adenocarcinoma (PAAD) tissues analyzed by the DNMIVD database. (Normal: maxima-12.14; minima-9.97; median-11.20; Q1-10.51; Q3-11.74. Tumor: maxima-13.04; minima-9.47; median-10.74; Q1-10.30; Q3-12.04). (**D**) Survival analysis of pancreatic cancer patients wherein blue trace corresponds to low MARCH3 expression ($n = 43$) and red trace corresponds to high MARCH3 expression ($n = 43$). (**E**) Survival analysis of pancreatic cancer patients wherein blue trace corresponds to low MARCH8 expression ($n = 43$) and red trace corresponds to high MARCH8 expression ($n = 43$). For statistical analysis, Mann–Whitney *U* test was performed for (**B**, **C**). Source data are available online for this figure.

 © The Author(s)

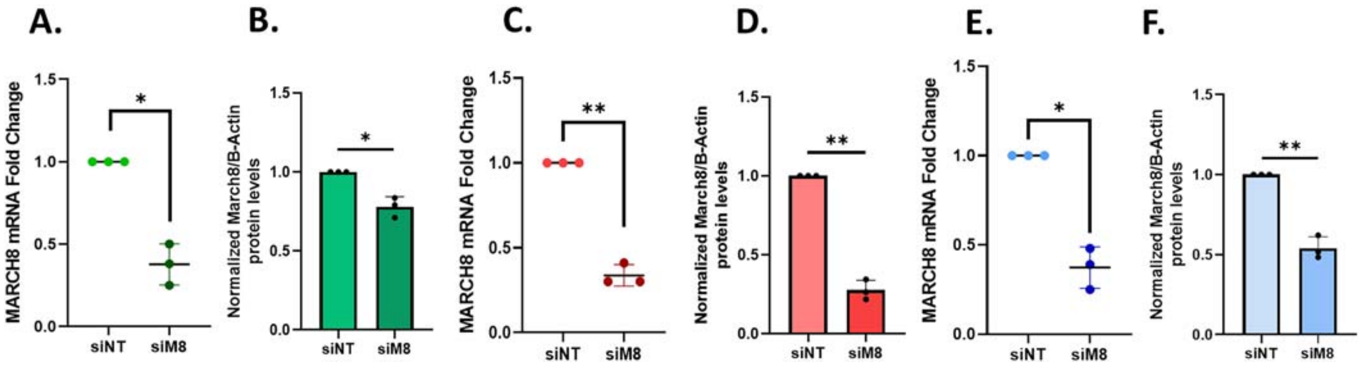

Figure EV5. siRNA mediated MARCH8 knockdown validation at RNA and protein levels in PC cells.

(**A**) qRT-PCR analysis showing decrease in MARCH8 mRNA levels upon siMARCH8 transfection in MiaPaCa-2 compared to control siNT ($N = 3$). *$P = 0.0131$. (**B**) Densitometric quantitation of MARCH8 protein levels in siMARCH8 transfection MiaPaCa-2 cells compared to siNT control ($N = 3$). *$P = 0.0266$. (**C**) qRT-PCR analysis showing decrease in MARCH8 mRNA levels upon siMARCH8 transfection in PANC-1 compared to control siNT ($N = 3$). **$P = 0.0030$. (**D**) Densitometric quantitation of MARCH8 protein levels in siMARCH8 transfection in PANC-1 cells compared to siNT control ($N = 3$). **$P = 0.0025$. (**E**) qRT-PCR analysis showing decrease in MARCH8 mRNA levels upon siMARCH8 transfection in CFPAC-1 compared to control siNT ($N = 3$). *$P = 0.0112$. (**F**) Densitometric quantitation of MARCH8 protein levels in siMARCH8 transfection in CFPAC-1 cells compared to siNT control ($N = 3$). **$P = 0.0080$. Data presented are mean ± SEM. For statistical analysis, one-sample *t* test was performed for (**A–F**) using GraphPad Prism software. Here, *$P < 0.05$ and **$P < 0.01$. Source data are available online for this figure.

© The Author(s)
